# What factors influence implementation of whole-school interventions aiming to promote student commitment to school to prevent substance use and violence? Systematic review and synthesis of process evaluations

**DOI:** 10.1186/s12889-022-14544-4

**Published:** 2022-11-22

**Authors:** Ruth Ponsford, Jane Falconer, G. J. Melendez-Torres, Chris Bonell

**Affiliations:** 1 Department of Public Health, Environments and Society, London School of Hygiene and Tropical Medicine, 15-17 Tavistock Place, WC1H 9SH London, UK; 2 Library and Archives Services, Department of Medical Statistics, London School of Hygiene and Tropical Medicine, Keppel Street, WC1E 7HT London, UK; 3 College of Medicine and Health, South Cloisters, University of Exeter, St Luke’s Campus, Heavitree Road, EX1 2LU Exeter, UK

**Keywords:** Systematic review, Whole-school interventions, School environment, Process evaluation, Substance use, Violence

## Abstract

**Background:**

Whole-school interventions go beyond classroom health education to modify the school environment to promote health. A sub-set aim to promote student commitment to school to reduce substance use and violence (outcomes associated with low commitment). It is unclear what factors influence implementation of such interventions.

**Methods:**

We conducted a systematic review including synthesis of evidence from process evaluations examining what factors affect implementation. Meta-ethnographic synthesis was informed by May’s General Theory of Implementation.

**Results:**

Sixteen reports, covering 13 studies and 10 interventions were included in our synthesis. In terms of May’s concept of ‘sense-making’, we found that school staff were more likely to understand what was required in implementing an intervention when provided with good-quality materials and support. Staff could sometimes wilfully or unintentionally misinterpret interventions. In terms of May’s concept of ‘cognitive participation’, whereby staff commit to implementation, we found that lack of intervention adaptability could in particular undermine implementation of whole-school elements. Interventions providing local data were reported as helping build staff commitment. School leaders were more likely to commit to an intervention addressing an issue they already intended to tackle. Collaborative planning groups were reported as useful in ensuring staff ‘collective action’ (May’s term for working together) to enact interventions. Collective action was also promoted by the presence of sufficient time, leadership and relationships. Implementation of whole-school interventions took time to build. Considering May’s concept of ‘reflexive monitoring’ (formal or informal review of progress), this was important in assessing and enhancing implementation. ‘Quick wins’ could help maintain collective impetus to implement further intervention activities.

**Conclusion:**

We identified novel factors influencing implementation of whole-school elements such as: local adaptability of interventions; providing local data to build commitment; interventions addressing an issue already on school leaders’ agenda; collaborative planning groups; and ‘reflexive monitoring’ as an explicit intervention component.

**Supplementary Information:**

The online version contains supplementary material available at 10.1186/s12889-022-14544-4.

## Background

Whole-school interventions go beyond merely providing classroom health education to modify the school environment to promote health [1]. A sub-set aim to promote student commitment to school to prevent outcomes such as substance use (i.e. tobacco, alcohol and other drugs) and violence. Whole-school intervention is an appropriate school health promotion strategy given that increased student commitment to school is associated with reduced substance use and violence [2, 3] and other health outcomes [4] so that promoting student commitment might prevent these risk behaviours. There is increasing academic and policy interest in such interventions, reflecting awareness that health-education lessons struggle to find a place in school timetables and have patchy results which tend to dissipate over time [5–8].

However, the effectiveness of whole-school interventions is unclear, given the lack of recent systematic reviews [1, 9]. Furthermore, although previous reviews have identified factors influencing initial delivery and sustained implementation of health interventions in schools [10, 11], these have not focused specifically on whole-school interventions. Key enablers of school interventions reported in these previous reviews are: strong institutional capacity (e.g. supportive senior management); alignment of the intervention with school ethos and priorities; positive pre-existing student and teacher attitudes; and parental support for interventions. However, whole-school interventions are more complex than the largely curriculum-based interventions examined in these previous reviews and so may be affected by different factors. Hence, a review of what factors affect the implementation of whole-school interventions is warranted in order to inform better implementation.

We conducted a systematic review synthesising evidence on whole-school interventions aiming to prevent substance use and violence. The overall review aimed to examine underlying theories of change, explore what factors affect implementation, and assess effectiveness and cost-effectiveness, and will be published shortly. This paper reports on the review and synthesis of process evaluations examining what factors affect implementation, which is a major influence on effectiveness [12]. The review was guided by the theory of human functioning and school organisation as to which interventions were included since this is the most comprehensive theory of how school environments may be modified to promote health [13, 14]. Rather than requiring included interventions to reference this theory (which might bias the review to certain geographies and disciplines), we instead required interventions to include one or more components aligning with constructs in this theory: modifying teaching to increase student engagement; enhancing student-staff relationships; revising school policies with students; encouraging students to volunteer in the community; or involving parents in school life.

The research question which our review of process evaluations aimed to examine was: what factors relating to setting, population and intervention influence the implementation of whole-school interventions aiming to prevent substance use and violence via promoting student commitment to school? Our analysis of implementation was informed by May’s General Theory of Implementation [15]. This is an appropriate framework for addressing our research question because it provides a detailed consideration of the processes involved in implementation and how these are influenced by the characteristics of the intervention and setting. Other frameworks, such as the RE-AIM framework [16], focus on implementation but not on underlying social processes or their influences. May’s theory describes implementers as engaging in processes of: ‘sense-making’ (understanding the intervention); ‘cognitive participation’ (committing to its delivery); ‘collective action’ (collaborating with others to ensure implementation); and ‘reflexive monitoring’ (assessing implementation and need for further action). The theory proposes various factors influencing the enactment of these processes: intervention ‘capability’ (the workability of and possibilities presented by the intervention); institutional ‘capacity’ (the material and cognitive resources, norms and roles present in a setting to support implementation); and ‘potential’ (individual and collective attitudes which might affect implementation). Our review also enabled an assessment of the state of evidence regarding process evaluations on this topic which might inform future work in this area.

## Methods

### Design

The overarching systematic review, from which this analysis draws evidence, followed guidelines for review conduct and reporting e.g. Centre for Reviews and Dissemination [17] and Preferred Reporting Items for Systematic Reviews and Meta-Analyses [18] (Additional File 1). The protocol was publically registered (https://www.crd.york.ac.uk/prospero/display_record.php?RecordID=154334). No protocol amendments were made.

### Identifying references

Informed by the constructs featuring in the theory of human functioning and school organisation as described above [13], we included studies of whole-school interventions (i.e. not merely classroom health education) aiming to reduce violence or substance use via: modifying teaching to increase student engagement; enhancing student-staff relationships; revising school policies with students; encouraging students to volunteer in the community; or involving parents in school life (intervention). Evaluations focused on children and young people aged 5–18 years (population) and the prevention of violence (defined as interpersonal physical, emotional or social abuse) and substance (i.e. tobacco, alcohol or other drug) use (outcomes). In this paper, we report on included process evaluations (design) which provided empirical findings on processes of intervention implementation.

Searches included terms for intervention, population and evaluation design. We originally searched 21 databases, three trial registries and 32 websites (16–27 January 2020), and updated the search (11–25 May 2021) across 14 databases, two trial registries and 32 websites (Additional File 2). Our searches deviated slightly from those in the protocol on the advice of an information scientist. Several databases that are no longer updated or which could not be accessed were dropped. A wider range of education, medical, nursing and public health databases were added to ensure the multi-disciplinary nature of this topic was adequately reflected in the references retrieved and to compensate for the dropped databases. The narrower scope of update searches was due to reduced accessibility of some sources in the context of the Covid-19 pandemic. We also searched reference lists of included studies and emailed topic experts.

### Screening references

Citations identified through searches were de-duplicated and uploaded to EPPI-Reviewer 4.0 software. Two reviewers then screened batches of the same 50 references, resolving disagreements by discussion if necessary. Our protocol was for these reviewers to double-screen the same references in batches of 50 until reaching a 90%+ agreement rate. This was achieved on the first batch where there was only one disagreement settled by discussion. Reviewers then single-screened references on title/abstract. Full reports of references not excluded at this stage were reviewed via an analogous process.

### Data extraction and quality-assessment

Two reviewers independently extracted data from included process evaluation reports on: study location, timing and duration; individual and organizational participant characteristics; study design; sampling and sample size; data collection; data analysis; findings; and interpretation. We assessed the methodological quality of process evaluations using the EPPI-Centre tool [19] addressing the (1) quality of sampling (e.g., was sampling appropriate to the questions?; were all stakeholders included?), (2) data collection (e.g., were tools validated or piloted?; was data collection comprehensive, flexible and/or sensitive to provide a rich description of processes?), (3) data analysis (e.g., was analysis systematic?; was diversity in perspective explored?), (4) the extent to which study data informed findings (e.g., were enough data presented to show how authors derived findings?; do the data presented fit the interpretation?), (5) whether the study privileged student perspectives (e.g., were students included?; was there a balance between open-ended and fixed-response options?), and (6) the breadth and depth of findings (e.g., were a range of process issues covered in the evaluation?; were the perspectives of participants fully explored in contrasting two or more perspectives and insight into a single perspective?). Informed by guidance, studies were rated as low, medium or high on the reliability of the findings, and as low, medium or high on the usefulness of the findings for addressing the research questions. Study reliability was judged high when steps were taken to ensure rigour in at least four of the above assessment criteria, medium when addressing only three and low when addressing fewer than three. To be rated ‘high’ on usefulness, studies needed to privilege student perspectives and present findings with breadth and depth. Studies rated as ‘medium’ usefulness only partially met this criterion, and studies rated ‘low’ were judged to have limited relevant findings.

### Synthesis

We synthesised process evaluation findings (including quotes from study participants) and author interpretations regarding the factors influencing implementation using meta-ethnographic synthesis methods. As with earlier reviews [20, 21], these were applied to textual reports of qualitative but also quantitative research, it not being possible to synthesise quantitative findings from process evaluations statistically because of methodological heterogeneity. In the case of findings from quantitative research, we coded author interpretations, checking whether these aligned with quantitative data. Meta-ethnographic analysis examined recurrent themes, identifying cases of ‘reciprocal translation’ (similar concepts being expressed differently in different sources) and ‘refutational synthesis’ (concepts from different sources contradicting each another). We then developed a ‘line of argument’ synthesis drawing together concepts from different sources to develop an overall analysis of factors influencing the implementation. The synthesis was not restricted to high-quality studies but poorer-quality reports were given less interpretive weight.

Synthesis involve the following steps. First, two reviewers prepared tables describing the quality, empirical focus and site/population of each study. Then the two reviewers piloted the analysis of two high-quality reports, reading and re-reading these reports and applying line-by-line codes. Next, reviewers drafted memos explaining these codes. Coding began inductively with in-vivo codes closely reflecting the words used in reports’ findings. The reviewers then grouped and organised codes, applying axial codes to identify higher-order themes. This stage of analysis was informed by May’s General Theory of Implementation as a sensitising device [15]. The two reviewers then met to compare these codes for the two studies, finalising an overall set of codes. This finalisation was facilitated by the reviewers having developed similar sets of codes. The reviewers proceeded to code the remaining studies drawing on the agreed set of codes, developing new codes as needed and writing memos to explain these. The two reviewers then met to compare their codes and memos, and agreeing a single set of overarching themes drawing on the strengths from each set of codes.

## Results

### Included reports and quality

**Fig. 1:**
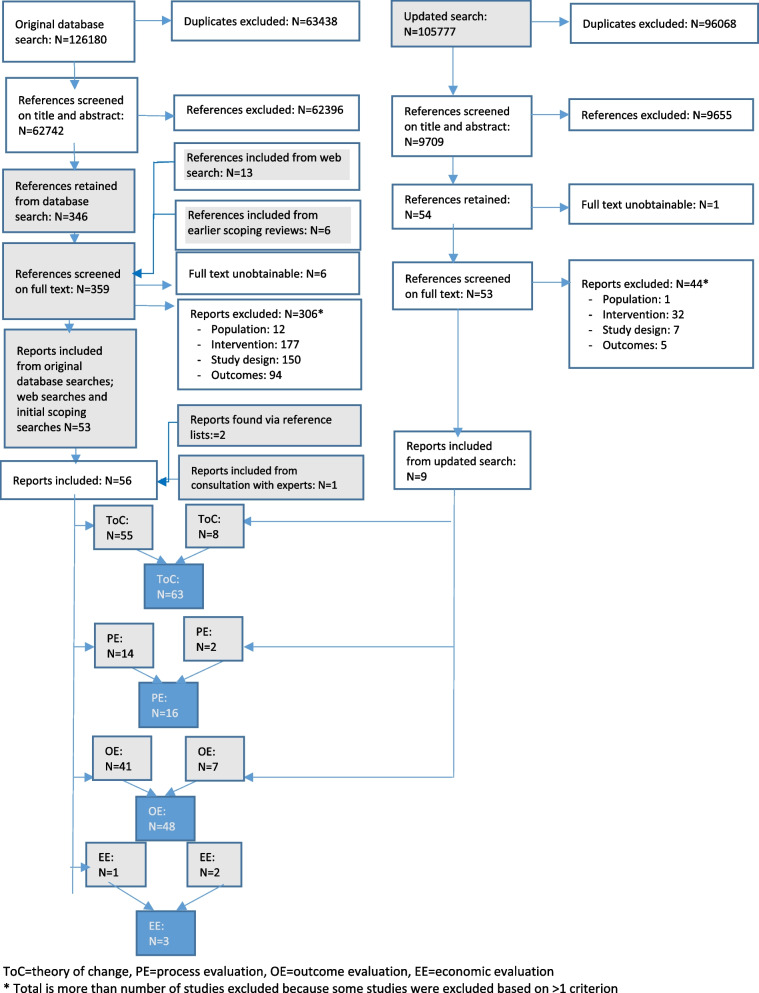
Searches and screening

The original searches retrieved 62,742 unique references and 56 eligible reports. The updated search retrieved 9,709 unique references and nine eligible reports (Fig. 1). In total, 65 reports on 27 studies of 22 interventions were included. Sixteen of these were process evaluations, covering 13 studies and 10 interventions [22–37]. Of these ten interventions, one was delivered to children approximating to English primary school age [5–10, 38], six to children of English secondary school age (age 11–18) and three to children whose ages spanned these ranges. Of the thirteen studies, five were from the USA, four from the UK three from Australia and one from Uganda. Nine reports drew on quantitative and qualitative data, five only on quantitative data and two only on qualitative data. Table 1 summarises the characteristics of process evaluations.

**Table 1:** Study characteristics

Study report and location	Intervention name, type and outcome addressed	Process evaluation design	Evaluation examined	Data collection methods	Evaluation participants and sample
**Cross** [28, 29] Australia/ Perth	Cyber Friendly Schools Student participation in policy decisions (Students sat on a decision-making group with staff) Bullying	Mixed methods	Feasibility Acceptability/ satisfaction Context	Students acting as cyberleaders were surveyed each year of the two-year intervention about their proposed activities and confidence in acting as a cyberleader. Cyberleaders also completed survey evaluations of the training activities at the end of the workshop and a brief telephone interview to measure their progress and any barriers to activity implementation at the end of each year. School project teams completed a baseline survey during the training workshop they attended and evaluations of training activities. Teaching staff completed baseline surveys prior to curriculum training and follow up surveys a year later. Project co-ordinators in each school were interviewed about their whole-school planning and activities at BL and at post tests in 2011 and 2012.	Students and school staff Cyberleaders completing surveys during training n = 87, n = 138 at post-test one, and n = 48 at post-test two. Teachers completing BL surveys n = 51. Teachers completing FU surveys at post-test 1 n = 78. Project co-ordinators completing interviews at BL n = 28. Project co-ordinator interviews at post-test 1: n = 25 and at post-test 2: n = 33.
**Bosma** [27] USA/ Minnesota	DARE Plus Student participation in policy decisions (other approaches) Drug use and violence	Quantitative	Feasibility Fidelity/ quality	Data collected included number and types of activities, numbers and names of participants, issues addressed, and leadership roles of team members. Seven forms were used to collect quantitative data on: (a) adult one-on-ones, (b) youth one-on-ones, (c) presentations at community meetings to recruit support, (d) adult action team meetings, (e) youth action team meetings, (f) adult activities, and (g) youth extracurricular activities.	Students and community members Not stated
**Cross** [28, 29] Australia/ Perth	Friendly Schools, Friendly Families Student relationships with teachers but not student participation in decisions or delivery (other approaches) Bullying	Quantitative	Coverage /reach/ accessibility Acceptability/ satisfaction	Parent questionnaire covering knowledge about bullying; self-efficacy to talk to children about bullying; parents attitudes to bullying behaviour; frequency of parent-child communication about bullying; parents perception of their ability to help their children respond to bullying; parents perception that bullying is a-priority at the child’s school. Data collected at baseline and ten months and twenty-two month post-points.	Parents 3211 parents completed the questionnaire at baseline. (1,077 Grade 2 parents, 1094 Grade 4 parents and 1,040 Grade 6 parents). 2152 parents at post-test 1 (10 months) and 56% 1784 at post-test 2 (22 months). 45% (n D 1,444) of the original cohort of parents completed surveys at baseline, post-test 1 and post-test 2.
**Bond** [24] Australia/ Victoria	Gatehouse Project Student relationships with teachers but not student participation in decisions or delivery (other approaches) Substance use, bullying	Mixed methods	Feasibility Acceptability/ satisfaction Mechanism Context	Field notes and records documenting meetings, the changes that occurred over time and the professional development provided to the school collected by the ‘critical friends’ from the Centre for Adolescent Health. Semi-structured interviews with ‘key informants’ (curriculum coordinators, student welfare, and administration) for each intervention school in the last year of implementation. At the end of each year, school background information was obtained on all schools via a structured interview with senior personnel. Audits related to school structures, policies, programs in place, strategies used to promote emotional well-being of students, and demographic information and could capture whole-school-level changes related to policies and programs.	School staff Not stated
**Knight** [31] Uganda/ Luwero district	Good Schools Toolkit Student participation in policy decisions (Students sat on a decision-making group with staff) Violence	Quantitative	Fidelity/quality Coverage/ reach/ accessibility	Cross-sectional survey at endline to measure student and staff exposure to the toolkit. Data on delivery of intervention to schools was collected routinely by intervention programme officers. All interactions with the schools— including technical support visits, group trainings and telephone calls—were systematically documented by each program officer termly. School led implementation was measured using termly ‘action plans’ routinely completed by schools. Adoption of toolkit elements by schools were tracked by an independent ‘Study Process Monitor’. Head teachers were asked a standard set of questions about Toolkit structures in each school termly, a sub-set of which were verified by direct observation.	Students and school staff 1921 students and 286 staff completed endline surveys.
**Bonell** [26, 39] UK/ south east England	Healthy School Ethos Student participation in policy decisions (Students sat on a decision-making group with staff) Substance use, violence	Mixed methods	Feasibility Coverage/ reach/ accessibility Acceptability/ satisfaction Context	Pre and post intervention survey of year-7 students conducted in private classrooms with support from two fieldworkers to examine reach. Semi-structured interviews with students and staff in intervention schools and intervention providers to examine feasibility, acceptability, awareness and contextual factors. Unstructured observations of various meetings to examine processes of participation and enable triangulation with interview accounts were also undertaken. Field notes written during observations and sometimes augmented later the same day from memory.	School staff, students and intervention providers Across all four schools, 721 (90.4 per cent) took part in the follow-up survey. Semi-structured interviews were carried out with: one head teacher per intervention school (n = 2); the external facilitator; the two trainers; and a sub-set of action team members. In one school three senior staff, one junior staff and one student involved in action groups took part (n = 5). In the other one senior staff, two junior staff and two students involved in action groups participated (n = 5). Interviews were also carried out with two other staff per school (one experienced one less experienced), four in total, who participated in the training as well as with three students in one school and five in the other who participated in other intervention activities and seventeen students in each school not participating in specific actions.
**Bonell** [25] **Fletcher** [30] UK/ south east England	Learning Together (Pilot) Student participation in policy decisions (Students sat on a decision-making group with staff) Bullying, aggression, substance use	Mixed methods	Feasibility	To examine fidelity of implementation of action groups documentary evidence was collected via intervention facilitator checklists, action group meeting minutes and school policies. To assess delivery of student needs assessment, response rates for the baseline survey were examined to ascertain if they were greater or lower than 80% of all year 8 students at the school. To examine reach of staff training and the uptake of restorative practices, evidence was collected from training provider and facilitator checklists. To examine the delivery of the student curriculum, evidence from intervention facilitators checklists was collected. Observations of training and action group meetings were carried out. Focus groups were carried out with students and school staff and semi-structured interviews with school leadership at each participating school. A subsample of action group members and all intervention providers were interviewed to explore their views on the intervention. Action group members at each intervention school were surveyed to examine their views on acceptability. Intervention reach was assessed via student survey. A teacher survey also included questions on implementation.	Students and school staff Students (n = 1114) and teachers (n = 336) took part in the follow follow-up survey. 34 action group members plus 16 other students and 4 staff took part in interviews. 112 students took part in focus groups.
**Bonell** [36] **Warren** [35] **Warren** [37] UK/ south east England	Learning Together Student participation in policy decisions (Students sat on a decision-making group with staff) Bullying, aggression, substance use	Mixed methods	Feasibility Fidelity/ quality Coverage/ reach/ accessibility Acceptability/ satisfaction Mechanism Context	n all schools, diaries were completed by trainers; researchers observed training; and staff completed a satisfaction survey for training. Semi-structured telephone interviews were also carried out with trainers. Diaries were kept by facilitators of action groups and minutes were reviewed. A survey was carried out with AG members each year of the intervention. Researchers carried out observations of action groups; semi-structured telephone interviews with AG facilitators in years one and two of the intervention; and semi-structured interviews with AG members (two per year) were also completed. A survey was carried out with staff leading curriculum implementation annually and semi-structured interviews were conducted each year with staff delivering the curriculum. In all schools a survey was also carried out termly with staff implementing restorative practice and interviews were conducted with other school staff in years 1 and 3. In six case study schools, focus groups were conducted with staff each year of the intervention and with students who were directly involved in intervention activities as well as those who were less so. Semi-structured interviews were also carried out with students involved in restoratives practice.	Students and staff Not stated.
**Mitchell** [33] USA/ Portland	Portland Peers Programme Student participation in policy decisions (Students sat on a decision-making group with staff) Substance use	Mixed methods	Feasibility Acceptability/ satisfaction	Biannual administration of the Portland Peer Helper Scale. Parent questionnaire. Initial assessment of student drug use. Interviews with project and school staff and students. Direct observation of a sample of program activities. Review of the student database of referrals for assessment.	Students, parents project and school staff Sample of research participants not described. The peer helper sample group was approximately 45% male and 55% female. The authors report that diverse ethnic groups were represented in the samples of this study p.13.
**Beets** [23] USA/ Nevada and Hawaii	Positive Action Student participation in policy decisions (Students sat on a decision-making group with staff) Substance use, violence	Quantitative (Cross sectional survey)	Fidelity/ quality Context	Year-end repeat cross sectional self-administered staff survey carried out in ten elementary schools at years 2 and 3 of the multiyear evaluation. The survey assessed: teacher perceptions of support from their administrators and connectedness with their school; their belief in their responsibility to teach social and character development concepts; their attitude towards Positive Action; the amount of the Positive Action curriculum delivered; and their usage of program-specific materials in the classroom and school wide context. School climate was assessed via of two series of questions assessing perceived administrative support and school connectedness.	Teachers In total 171 teachers in the ten elementary schools completed year-end process evaluations in year two and 191 in year three.
**Malloy** [32] USA/ Chicago	Positive Action Student participation in policy decisions (Students sat on a decision-making group with staff) Substance use, violence	Quantitative	Fidelity/ quality Context	Teacher unit implementation reports (UIRs) from first year of implementation (2004-5) administered as six time-points. Teacher work climate survey administered at baseline and after training.	Teachers Forty-six teachers who completed both the URIs (for one or more of the units) and the Teacher Work Climate Survey were included in the analysis.
**O’Hare,** [34] UK /south east England	Positive Action Student participation in policy decisions (Students sat on a decision-making group with staff) Substance use, violence	Mixed methods	Feasibility Mechanism Context	Teachers completed an implementation survey at the end of each unit of the programme for lessons and the end of every week for classroom activities used and whole-school activity to assess dose. Head teachers completed a school climate survey. Students completed a satisfaction questionnaire measuring their engagement and pupil-teacher relationships. Qualitative data was collected through classroom observations to assess fidelity (quality). Pupil focus groups and teacher and head teacher interviews were conducted to explore implementation.	Students and staff Nineteen teachers completed the implementation survey. For survey (engagement and relationships measures), 473 students moving through years 4 and 5 across fifteen schools. Twenty-five students from five schools randomly selected from the 15 took part in focus groups. One teacher and one head teacher was interviewed from each of the five randomly selected schools.
**Anyon** [22] USA	Responsive Classrooms Student Participation in Decision-making (other approaches) Violence	Mixed methods	Feasibility Fidelity/ quality Acceptability/ satisfaction Context	Observation by trained research staff of teacher implementation of RC rated on a three-point scale. Classroom practices frequency survey (CPFS) to capture teachers’ self-reported use of intervention strategies. Focus groups carried out with a range of school staff to examine factors that constrain or enable implementation.	School staff Sample included thirty school staff. Twenty-four teachers participated in classroom observations at two time-points, and 19 teachers completed the classroom practices survey. Fifteen participants completed a first round of focus groups in fall 2013, and 19 individuals participated in spring 2014.

Initial agreement over study quality was high (> 90%). Three studies were judged to be of both high reliability and high utility in addressing our research questions. [25, 26, 30, 35–37] One study was judged to be of high reliability and of medium utility in addressing our research questions. [22] One study was judged as medium reliability but low utility. [23] A further study was judged as being of low reliability but of medium utility. [24] Four studies were judged as of low quality and low utility [27, 28, 33, 34]. Three studies were rated as of high [31, 32] or medium reliability, [29] but low utility (Table 2).

**Table 2:** Quality of process evaluations

Study report	Were steps taken to minimise bias and error/increase rigour in sampling?	Were steps taken to minimise bias and error/increase rigour in data collection?	Were steps taken to minimise bias and error/increase rigour in data analysis?	Were the findings of the study grounded in/supported by data?	Was there good breadth and depth achieved in the findings?	Were the perspectives of young people privileged?	Overall reliability of findings based on the above (low, medium or high)	Overall usefulness of findings to this review low, medium or high
**Cross** [28, 29]	No None specified. Recruitment and sampling of participants for process evaluation not discussed.	No None clearly specified. Reference to use of a standardised protocol for project co-ordinator interviews.	No None specified.	No Discussion of findings is limited and does not flow clearly from methods as described. Full range of data is not discussed and there is a lack of clarity concerning where some findings are drawn from. Data tables or quotes from qualitative data are not provided to support findings.	No Surface level data on training satisfaction for cyeberleaders discussed, but full range of findings from different data sources and participants not presented. Touches on a broad range of potential implementation issues identified in student data, but these are covered in very limited depth. Qualitative data is not well reported or utilised.	No Presentation of student responses to surveys is limited and young people’s accounts from interviews are reduced to lists.	Low Limited information on methodological rigour is provided. Unclear how bias and error were controlled for in sampling, data collection and analysis. Limited data presented to support findings.	Low Some useful insights in relation to factors facilitating and impeding implementation of the intervention, but focus is mainly on cyberleader component and discussion of findings lacks depth.
**Bosma** [27]	No None described.	No None described.	No Methods of analysis are not described.	No Limited data provided to support findings. Data sources for many of the findings are not clear and limited data examples are used to support findings.	No Findings are largely limited to describing levels of participation and frequencies of events/activities implemented. No data on participant views is presented.	No Data on young people’s perspectives is not included.	Low Methods are poorly described and it is unclear from what data many of the findings and conclusions are drawn.	Low Findings on factors affecting implementation are limited.
**Cross** [28, 29]	No Self-selected sample.	Yes	Yes Appropriate statistical tests used.	Yes Detailed data tables are provided to support findings.	No Limited data from parent questionnaire examining reach and dose only.	No	Medium Sampling open to self-selection, but otherwise well conducted study.	Low Limited information on factors influencing implementation of parent component.
**Bond** [24]	No Sampling methods for individual participants unclear.	No None stated.	No None stated.	No Quotes used in places but not all findings are supported with data or linked clearly to data sources.	Yes Multiple aspects of and perspectives on implementation explored in good depth using qualitative data.	No	Low Limited information on sampling of schools and participants provided and findings not always supported by data presented. Limited information to assess quality and robustness of methods and analysis provided.	Medium Provides useful information to understand how features of the intervention (and to a lesser extent) context may facilitate/support implementation. Range of data collection tools and multiple perspectives used to explore implementation, but not data from young people included.
**Knight** [31]	Yes All students and staff invited to participate, with high student and staff response rates reported. All head teachers included in assessment of implementation.	Yes Standardised data collection tools used. All measures pilot tested before use.	Yes Triangulation (observation and teacher report) used in measurement of adoption. Appropriate statistical models and tests for reliability used.	Yes Data described in detail and full data tables provided to support conclusions.	No Range of measures, data collection methods and participants included to explore implementation, exposure/reach, but focus is on a limited set of questions on how level of implementation affected reach and outcomes, with some data provided on how reach varied by participants. In depth perspectives on implementation and factors shaping delivery or receipt are not explored.	No Student survey data on reach is included and forms part of the main analysis, but wider perspectives on implementation are not included.	High Standardised data collection tools used and triangulation with direct observation to verify findings on implementation. Survey measures piloted and were subjected to appropriate tests for reliability. Comprehensive data tables are provided to support conclusions.	Low Some useful data on how receipt (reach) varies by gender, year group and educational needs but main focus is on how level of implementation impacts reach and outcomes. Does not contribute useful information on how features of interventions, context or providers influence implementation.
**Bonell** [26, 39]	Yes Large subset of participants used for qualitative data collection, purposively selected to ensure diversity, capturing a range of roles and levels of involvement with the intervention. Student sample selected to broadly reflect student body.	Yes Questionnaire was piloted with similar age students in another school and conducted, privately in classrooms with fieldworkers. Interviews were conducted by researchers in private rooms using standardised interview guides.	Yes Interviews were recorded and transcribed in full. Two researchers coded the data (both inductively and deductively) and compared, contrasted their analysis and interpretation before coding a second time to arrive at a final set of themes. Unstructured observations of meetings were triangulated with interview accounts.	Yes Supported with appropriately detailed data tables and illustrative quotes from a range of participants.	Yes Range of aspects of implementation explored in detail via multiple participant perspectives and data sources, including in-depth qualitative data.	Yes Student accounts used to address relevant research questions.	High Steps taken to avoid bias and improve rigour in sampling, data collection and analysis. Data, including sample of appropriately illustrative quotes presented to support findings.	High Provides highly useful information drawing largely on in-depth qualitative data from a range of participants to illustrate key factors facilitating and impeding implementation.
**Bonell** [25] **Fletcher** [30]	Yes. Schools purposively selected for diversity. For qualitative data collection individuals were selected purposively to represent diversity of students and staff.	Yes Student surveys completed individually in classroom settings monitored by researchers not teachers. Staff surveys completed anonymously and confidentially. Interviews/FGs conducted by researchers in private rooms using standardised and pre-piloted interview schedules. Standardised data collection tools (checklists) also used to monitor implementation.	Yes Detailed and layered qualitative analysis methods described, drawing on well recognised methodological approaches. Data thoroughly triangulated (verified) using range of data sources. Thorough approach to qualitative analysis including double researcher coding/analysis and regular discussion and refinement of coding and interpretation. Log book of decisions was used.	Yes Findings flow logically from methods and are supported by detailed data tables, descriptions of participant responses and a number and range of quotes from different participants.	Yes Perspectives from a range of participants on a number of different areas of implementation explored using both quantitative and qualitative data, providing good breadth and depth of enquiry.	Yes Student responses considered in detail and lengthy quotes used to support interpretations.	High Well conducted study with appropriate steps taken to increase rigour in sampling, data collection and analysis. Findings follow from methods and are well supported with detailed descriptions and data.	High Range of implementation factors related to delivery and receipt considered using robust methodological approach. Provides high quality, in-depth information from a range of participants on factors influencing implementation.
**Bonell** [36] **Warren** [35] **Warren** [37]	Yes Either all relevant sample included in research activities or participants were purposively sampled for diversity.	Yes Surveys were anonymous, completed independently by students in classrooms with a researcher present and returned in envelopes sent to researchers. Interviews and FGs were conducted in private rooms with only researcher present.	Yes Multiple data sources used to triangulate data. Analysis of qualitative data carried out by two researchers using standardised coding framework and recognised methods of qualitative analysis drawn from grounded theory, such and constant comparison and examination of deviant cases.	Yes Findings are described in detail and follow logically from methods. Full data tables and appropriate, lengthy quotes from a number of participants are provided to support conclusions.	Yes Range of data collection methods and participants included across all schools providing very comprehensive picture of implementation.	Yes Survey data and qualitative data from young people is drawn upon and discussed in detail as part of the main findings.	High Well conducted study which includes broad range of measures, methods and diversity of participants, with data collected over a three-year intervention period creating a very comprehensive and reliable picture of implementation.	High Well conducted study using range of methods to capture diverse perspectives on breadth of implementation issues.
**Mitchell** [33]	No Methods of sampling not described.	No None stated. Data collection methods are poorly described.	No None stated. Data analysis methods are not described.	No Unclear from what data sources findings have been derived. Data is not presented to support findings.	No Limited detail/depth to findings on implementation and qualitative data is poorly reported.	No Interviews were carried out with students, but these are reduced to case studies written by researchers.	Low Methods are poorly described so is difficult to assess rigour and quality of study. Discussion of findings is limited and sufficient data are not provided to support conclusions.	Low Findings on implementation are limited and it is difficult to assess the rigor and quality of the study. Small amount of useful data provided on intervention acceptability and features of intervention that impeded implementation of parent component.
**Beets** [23]	No All staff invited to participate, but response rates were low in some schools and sample may be subject to self-selection bias.	Yes Surveys anonymised to promote more “truthful” answers (teachers only asked to identify year and grade level taught). Validated and pre-piloted scales used to measure key constructs.	Yes Appropriate statistical analysis and testing used accordingly.	Yes Data to support interpretations clearly presented in tables.	No Focus is on teacher survey data relating to few key concepts related to implementation.	No Study does not include student data.	Medium Appropriate steps taken to minimise bias data collection and analysis, but not sampling. Data to support findings is presented.	Low Very useful information on role of teacher beliefs and attitudes and perception of school climate in shaping implementation of curriculum and whole school materials, informed by appropriate theory. Although analysis is limited to quantitative data from teachers and small range of concepts and variables used.
**Malloy** [32]	Yes All teachers invited to participate, with 73% response rate. Data collected for the teachers who did not take part showed they were not significantly different from those that did, suggesting the sample was representative.	Yes Existing predictor variable measures were used and piloted a refined using principle component factors analysis prior to their use. Standardised data collection tools and measures used for teacher reported implementation.	Yes Although UIRs were self-report and some of the implementation data were missing, weekly implementation data were triangulated with end of term summaries and with student reported levels of engagement with the program, which supported the validity of these data. Appropriate statistical tests used.	Yes Findings follow logically from methods and full data tables are used to support conclusions.	No Limited range of concepts related to implementation explored using staff survey data.	No Focus on teachers.	High Well conducted study with bias and error in sampling, data collection and analysis accounted for. Sufficient data to support findings is provided.	Low Provides useful and reliable data on association between teacher perceived organisational climate and implementation, but sample and breadth and depth of analysis is limited, with other implementation issues relating to intervention, context or population not explored.
**O’Hare,** [34]	Yes All students and teachers in study invited to participate in survey (although response rates not reported. Unclear if sample was representative). Schools selected at random for qualitative work. Students for FGs selected at random from five selected schools. Unclear how teachers for interviews were selected.	Yes Survey measures were developed based on existing pre-tested scales. Observation, focus group and interview schedules were piloted in in each of the fifteen schools in an earlier phase and refined prior to implementation.	No Methods of data analysis not described.	No Full data tables are provided to support quantitative findings. Description and presentation of qualitative data is limited, however.	No Range of methods used to capture information on different aspects of implementation from different perspectives. Depth of data on participant perspectives limited though.	No Use of student data from FGs is limited.	Low Steps taken to reduce bias and error in sampling and data collection but methods of analysis not described and description and presentation of qualitative data is poor.	Low Although depth of data on implementation is limited, provides some useful data on student engagement and characteristics of interventions that might affect this (and consequently implementation and outcomes) as well as on feasibility for schools in terms of curriculum dose and challenges to implementing whole-school elements.
**Anyon** [22]	Yes All staff members invited to participate. Potential for self-selection bias, but researchers claim sample was representative of total population.	Yes Different instruments used to triangulate data on implementation. Standardised protocol used for qualitative data collection and previously validated instruments used for quantitative data collection.	Yes Quantitative and qualitative data triangulated. Qualitative data analysis carried out by three independent coders. Appropriate statistical tests used for quantitative data.	Yes Data presented to support quantitative findings. A number of appropriate participant quotes used in text to support qualitative findings.	Yes Mixed methods used to capture both breadth (level of implementation across all classrooms) and depth (factors shaping this). Comprehensive data collection on implementation and factors shaping this. Range of teaching staff included to capture different perspectives, but lacks data on student perspectives.	No No student data reported in study.	High Steps taken to minimise bias in all areas.	Medium Good quality, detailed information on implementation factors provided, but no student data reported.

### Synthesis of evidence on factors affecting implementation

Various themes and sub-themes were apparent in quotes from study participants and author interpretations, the structure of which is summarised in Table 3. These are presented below structured according to the constructs from the General Theory of Implementation (indicating by these being in inverted commas) with which they aligned. References indicate which studies informed which themes.

**Table 3:** Coding structure

Process Construct from General Theory of Implementation	Influence construct from General Theory of Implementation refined to encompass school factors	Influence Sub-construct (where relevant)
Sense-making	Intervention capability to be made sense of – good materials and support	
	School capacity to make sense of an intervention – rooted in existing priorities and capacities	
Cognitive participation	Intervention capability for local tailoring and adding value	Particularly for whole-school components where these could jeopardise other work
	Intervention capability for using data to build commitment	But can undermine as well as build commitment
	Intervention capability in terms of student participation	
	Staff potential for commitment based on perceived need	
	Staff potential for commitment based on existing strategies and values	Cherry-picking components most aligning with potential
Collective action	Intervention capability as workable	
	Planning groups as a key element of intervention capability	Planning groups and participative decisions as a potential source of deviation
	Synergy between intervention components as a key element of intervention capability	
	School capacity to support collective action	Time resources
		Leadership resources
		Staff/school relational and culture resources
Reflexive monitoring	Intervention capability for reflexive monitoring	
	Collective reflexive monitoring to refine implementation	
	Reflexive monitoring reinforcing implementation	

#### Sense-making

‘Sense-making’ was a recurrent theme in studies i.e. a process of staff coming to understand the intervention which contributed to the enactment of interventions. Sense-making was reported to accrue over time and pervade all, not just the initial, stages of implementation [22, 24, 26, 30, 35]. Various factors were reported to affect how school staff and students understood intervention resources.

##### Intervention capability’ to be made sense of

A sub-theme suggested that sense-making could be facilitated by an intervention’s ‘capability’ (i.e. workability). This could be in terms of providing good-quality materials and/or ongoing support in the form of training, external facilitation or coaching [22, 24–26, 30, 33–36]. Materials and resources that included tangible, contextually relevant examples were reported as enabling providers to understand how intervention activities might occur in their setting, for example as reported by one head-teacher in an evaluation of Learning Together rated as of high reliability and usefulness [25]:

“The one thing schools need is a model, of how it’s going to work in the school, in a real-life school, so that they can almost touch it, taste it, feel it, and then start implementing it in their own schools”. (p.39)

Two studies reported that staff were sometimes initially confused by intervention materials or external providers [25, 26]. In the study of the Healthy School Ethos intervention rated as of highly reliability and usefulness [26], an initial presentation by an external facilitator was reported to have caused staff and students to misunderstand the aims of a whole-school intervention.

##### School ‘capacity’ to make sense of an intervention

Another sub-theme apparent in one UK study was how staff’s making sense of an intervention could be influenced by their existing priorities and the school’s institutional ‘capacity’ in terms of the resources present to support implementation [22, 26, 35, 36]. Those leading implementation in one school were said to have creatively reinterpreted Learning Together, an anti-bullying intervention, as an intervention aiming to maintain the emotional health of pressurised students in an academically selective school [35]. This evaluation further reported that, in another school, the lead reinterpreted the staff-student action group as being a site for students to learn the skills needed to avoid or respond to bullying (rather than, as intended, to coordinate intervention activities). This occurred in the context of the lead’s imprecise grasp of the intervention and inability to involve other staff.

#### Cognitive participation

The notion of ‘cognitive participation’ also recurred as a theme across studies, presented as a process of staff commiting to implement an intervention. Various factors concerning the intervention and the school were identified as influencing the extent to which school agents felt able to commit to enact intervention activities. Like sense-making, cognitive participation was a process that was built across all stages of implementation [22]. Several factors affected how cognitive participation developed.

##### Intervention ‘capability’ for local tailoring and adding value

A key sub-theme apparent in several studies was that school staff assessed intervention ‘capability (workability) in terms of ease of integration with existing practices [22, 24–27, 36]. Interventions that could be locally tailored or build on existing work were more likely to secure staff’s cognitive participation. An evaluation of low reliability and usefulness of the Drug Abuse Resistance Education (DARE) Plus intervention [27] described how the assessment phase of the intervention was essential to tailor the intervention and develop commitment:

”The assessment phase of the organizing process is critical to its long-term success. It is invaluable to take the required time to get to know the community before attempting to launch an action team.” (p.17)

Another report describes how school staff bought-in to use of restorative practice as an approach to discipline because this was viewed as providing a means of building on existing work and developing a consistent approach to discipline [25].

Interventions not viewed as being capable of local tailoring often failed to engender staff commitment, as reported by evaluations of the Responsive Classrooms and Positive Action interventions, respectively of high reliability and medium usefulness and low reliability and usefulness [22, 34].

A sub-theme apparent was that this lack of intervention capability for tailoring or adding value was particularly undermining for whole-school elements [22, 34]. Head-teachers and other school leaders could withhold commitment when they felt that whole-school actions might jeopardise their wider strategies. This could be the case, for example, where interventions required changes to school rewards or discipline policies that school leaders thought might weaken the school’s ability to pass school inspections or attract parents to send their children to the school. As an evaluation of Positive Action [34] reported:

”Reluctance to change whole-school policy may be exacerbated by circumstances such as an upcoming [government] inspection: ’It was hard to make a whole-school change to sanction and reward policy, so whole-school activity was harder to implement. [The government inspectorate] was coming and it would have been too big a change.’” (p.34)

##### Intervention ‘capability’ for using data to build commitment

Another sub-theme was that the provision of local data as part of the intervention could improve its ‘capability’ (workability) and build staff commitment [24–27, 30, 35, 36]. The evaluation of the Learning Together intervention suggested that providing such data could make it harder for staff to dismiss the need for intervention [25, 35, 36]. A staff-member on a pastoral team commented [35]:

“I remember when [facilitator] came to present to [senior leadership team] and said how terrible our data was… it was like a tumbleweed moment; it was so funny. I mean... it wasn’t funny in a good way, but... but it was a realistic... realisation for everyone if you know what I mean... Because we all knew it was like that, but we didn’t realise how much the children didn’t actually like us.” (p. 990)

However, in an example of refutational synthesis, several studies identified that the provision of data could sometimes undermine staff commitment when staff interpreted the data as a criticism of their work to date or where data did not indicate positive trends after implementing an intervention [26, 30, 36, 37].

##### Intervention ‘capability’ in terms of student participation

A sub-theme from several UK evaluations of interventions aiming to encourage student participation in decisions was that students were more likely to commit to an intervention where this offered an opportunity for them to express their views [25, 26, 34–37].

##### Staff ‘potential’ for commitment based on perceived need

Staff commitment to interventions was influenced by staff’s ‘potential’: whether staff were attitudinally ready for such an intervention. A key sub-theme was that interventions should offer school leaders something they already knew they needed [22–24, 34, 36]. This might be a way of responding to government policies, pressures from parents or inspection requirements. Or it might address internal imperatives, such as school leaders’ existing strategies for school change. This theme was particularly clear in the UK studies of both Healthy School Ethos [26] and Learning Together [25, 30, 35, 36]. The pilot evaluation of Learning Together [30], for example, reported:

“head teachers and their [management teams] consistently reported that it was important to address aggressive behaviours in order to recruit and retain ‘the best’ parents and students. [Managers] also suggested that this project was prioritised as it was seen as likely to impress the national school inspectorate… due to its focus on student voice and behaviour.” (p.328)

Interventions aiming to achieve whole-school change were more likely to get school leaders’ commitment when there was already a recognised need for change, for example because of poor inspection results [25, 30]. Reciprocally translating with this concept, it was apparent that in schools where leaders perceived no such urgent imperative for change, genuine school commitment was less likely.

##### Staff ‘potential’ for commitment based on existing strategies and values

A related sub-theme was that school staff had more attitudinal ‘potential’ for commitment to a whole-school intervention when their existing strategies and values made this seem attractive [22, 23, 25, 30, 32, 36]. New head-teachers were reported as particularly likely to commit to interventions involving whole-school change because these aligned with their desire to make their mark and change schools [30]. Reciprocally translating with this concept of school leaders’ ‘potential’ was teachers’ ‘potential’ [23, 25]. For example, teachers with a prior commitment to social and character education within their classes were more likely to implement curricula addressing this according to a study of Positive Action of medium reliability and low usefulness [23].

In cases where the values or priorities did not align, staff commitment appeared less likely [22, 23, 32]. For example, where staff or students perceived restorative practice to be a softer option, they were reportedly unlikely to commit to enacting it. As a study of Responsive Classrooms [22] reported:

“In contrast, some middle school staff members’ beliefs about the value of punitive responses to problem behavior were incompatible with the core tenets of the intervention, which emphasized inclusion and opportunities to learn: ’When you steal, there are real consequences; there’s jail or fines…’ These staff members believed that zero-tolerance policies, which use punishment as an extrinsic motivator for behavior change, were more effective than RC approaches.” (p.84)

A sub-theme concerned the possibility of schools committing to implementing only those intervention components aligning with their existing strategies and values, rejecting components that they regarded as deviating from these [26, 36].

#### Collective action

Evaluations also examined processes by which those in schools engaged in ‘collective action’ (working together) to divide up responsibilities for delivering interventions. A number of factors were identified as influences on such processes.

##### Intervention ‘capability’ as workable

A key sub-theme was the importance of interventions being locally workable for staff enacting interventions as planned [22, 26, 36]. For example, curriculum materials which did not fit into the school curriculum or which did not provide staff with clear lesson plans tended to be adapted before they were delivered, or were not delivered at all [24, 35, 36].

An important aspect of workability was the extent to which guidance materials spelt out how delivery should proceed. For example, materials underpinning a restorative practice interventions needed to specify which staff-members were responsible and whether the intervention was intended to complement or replace punitive discipline [22].

Some interventions were not collectively enacted as had been planned. For example, an evaluation of Responsive Classrooms [22] found that a new approach to discipline failed to work within the reality of schools:

”[P]articipants reported that a key RC strategy, Logical Consequences, in which a response to student misbehavior is tied to the specific incident and creates an opportunity for learning, was too unwieldy to implement in a way that students could anticipate and incorporate: ’I totally agree with the theory behind logical consequences where you want the consequences that match the behavior and that’s, like, respectful to the child and respectful to the teacher. But it’s hard because it’s different every time… It’s not a system where they know, like, oh, if I do this I know what’s going to happen.’” (p.85)

##### Planning groups as a key element of intervention ‘capability’

An important sub-theme was that interventions which included planning groups, consisting of staff and sometimes students, parents or other community-members, were more workable in ensuring collective action. This was apparent from reports of the Gatehouse Project (of low reliability and medium usefulness), Learning Together interventions (of high reliability and usefulness) [24–26, 35–37] and other interventions [23, 24, 27, 32–34]. Diverse participation in such groups could support implementation by ensuring that the decisions made by the group were pragmatic and by achieving wider commitment across the school.

Such groups were reported to be particularly facilitative of whole-school approaches [24, 26, 35, 37]. These groups could also help ensure that intervention activities added up to a coordinated process of integrated school transformation, rather than merely a disparate set of initiatives.

##### Synergy between intervention components as a key element of intervention ‘capability’

A further sub-theme was that some interventions were more workable because they had better synergies between intervention components than others [22, 24–26, 30, 35–37]. Some intervention activities created the informational and relational resources needed to enable agents to enact other actions. The evaluation of the Gatehouse Project [24] reported for example:

“It is clear from our work that these elements - the adolescent health team, the school social climate profile, and the critical friend - do not work in isolation. The profile provides local data that are essential for identifying risk and protective factors relevant to the particular school community. The adolescent health team ensures that the responses to the profile are owned and implemented by the whole-school community. The critical friend provides expertise, impetus, motivation, and links to external resources.” (p. 380)

As described above, data on student needs being provided as part of an intervention could encourage others to implement intervention activities or lead to school staff producing or sharing other data [25, 30].

One area of synergy was where training components provided staff with the skills they needed to deliver other intervention elements. This could be valuable in ensuring staff accumulated and consolidated their skills [22].

Other reports focused on lack of intervention-component synergy as an inhibitor of collective action. For example, some evaluations reported that there was a noticeable lack of effective interaction between curriculum and whole-school components. In some cases, classroom curriculum activities were enacted but whole-school changes were incompletely delivered [34]. In other case, whole-school elements which aimed to build on existing school achievements were enacted but curriculum elements were not delivered with fidelity because these were judged unworkable [35, 36].

##### School ‘capacity’ to support collective action

The extent to which agents in schools could come together to collectively enact interventions also depended on school ‘capacity’ (i.e. the resources available to these agents). The lack of space in school timetables, and the lack of non-contact time within which school staff could plan intervention activities was frequently reported by evaluations [22, 25, 27, 28, 33, 34, 36]. For example, the evaluation of low reliability and usefulness of the Cyber Friendly Schools intervention reported [28]:

“Many teachers reported not being able to find sufficient time in their teaching curriculum to complete the eight learning activities.” (p. 104)

In the evaluations of low reliability and usefulness of the DARE Plus intervention and the PPP intervention [27, 33], whole-school elements were described as the most challenging and time-consuming to organise.

Staff struggled to marshal time and other resources when they were expected to deliver a new intervention alongside other initiatives. These situations diffused the resources available for any one intervention and eroded agents’ ability to commit the time needed to support effective decision-making and delivery. The evaluation of Responsive Classrooms for example reported [22]:

”A school leader noted that ‘It’s not one new thing; it’s always five new things that we’re working on. I think the attention span is tested.’” (p.84)

Another resource factor in determining whether interventions were collectively enacted with fidelity was whether those charged with leading the intervention possessed leadership resources, such as a budget, the ability to direct other staff or the ability to modify policies or systems [22, 25–27, 30, 36, 37, 39]. Schools that gave intervention leadership roles to powerful staff consistently achieved better implementation according to several evaluations. Power and authority could be formal or informal, the latter reflecting individuals or groups having a long track-record at the school, strong relationships and an informal ability to persuade people to make things happen [35, 36]. An evaluation of Learning Together [35] for example reported:

”In another school, despite there being no senior leaders on the group, the lead had worked for a long time at the school and was well respected and liked by both students and staff. Thus, it was possible to galvanise action without the formal involvement of senior leaders in some cases.” (p.989)

Where leadership commitment to intervention activities was limited or inconsistent, there may thus have been less collective vision and impetus for implementation, as reported in the evaluation of the Responsive Classrooms intervention [22]. Lack of senior level support could also affect the drawing down of material and cognitive resources to support intervention activities [35, 36]. For example, some decisions made by action groups were stalled or rejected by other agents within the school system, such as head-teachers or school-leadership teams [35, 36].

Interventions could also be better implemented in schools characterised by strong connections between staff or with strong cultures of innovation [22–24, 32, 36]. In schools with strong connections, those agents leading interventions could draw on existing relational resources such as mutual support, observation and learning to support enactment, rather than attempting to develop this from a low baseline. An evaluation of Positive Action [32] of high reliability and low usefulness reported:

“Stronger affiliation among teachers likely led to more opportunities to share ideas about PA materials and observe other teachers as they carried out PA activities outside of the classroom. This may have influenced teachers’ use of these supplementary program components, with higher levels of use by teachers who had perceptions of high engagement and support among teachers in their schools.” (p.1091)

An evaluation of the Gatehouse Project [24] similarly reported the importance of networks connecting staff in enabling collective action.

A culture of teacher autonomy, as reported in the evaluation of Friendly Schools [22], could undermine collective action, because it was difficult for those leading an intervention to encourage the consistent enactment of new practices which deviated from locally understood norms and expectations of staff roles. Similarly, the evaluation of the Responsive Classrooms [22] intervention reported:

“School staff observed that [Responsive Classrooms], a schoolwide intervention, ran counter to the school’s culture of individuality. For example, one teacher noted: ’One… characteristic of [the school is]… there’s a lot of autonomy in terms of how teachers run their classrooms… it’s a little bit of territorial, like… I know what I’m doing and I have my way of doing it so I don’t need to participate necessarily in a whole-school anything.’” (p.84)

A staff culture of innovation could also support collective implementation. Such cultures could encourage staff to take the time to identify who would implement the intervention and then enact this with fidelity [23, 34].

#### Reflexive monitoring

Whole-school interventions took time to build. ‘Reflexive monitoring’ (whereby staff assessed the success of implementation through formal or informal processes) was important in determining the extent to which implementation built or dissipated over time.

##### Intervention ‘capability’ for reflexive monitoring

Reflexive monitoring worked well when interventions included this as an explicit component [24, 26, 30, 36] increasing their ‘capability (workability). Studies indicated that interventions were particularly successful when they included an action group that reviewed data, identified priorities, oversaw delivery and reflected on the results. This enabled members to reflexively monitor what was being enacted and with what consequences. Evaluations suggested that this gave participants the permission and resources to try different things, persist with what was perceived as working and refine or reject what was perceived to go less well. This approach allowed staff to abandon activities viewed as unsuccessful without rejecting the intervention overall. For example, an evaluation of the Gatehouse Project [24] reported:

”This common purpose gave permission for teachers to try new strategies such as substantially restructuring student and teacher teams. For example, in one school, teachers worked together to reorganize classes into small groups of four or five learners and teachers into teaching teams to promote a collaborative and an academic environment.” (p.375)

As part of processes of reflexive monitoring, ‘quick wins’ evidencing positive outcomes could also can help maintain and further build coalitions and commitment, and collective impetus to implement further intervention activities [27].

As well as groups, ongoing support from training, facilitation or coaching could also support reflexive monitoring by providing an opportunity for reflection and/or an outsider perspective. The importance of an external facilitator was, for example, described as follows in an evaluation of the Gatehouse Project [24]:

“The support that [critical friend] provided in the staff room, in staff meetings, has been invaluable. We wouldn’t be where we are now, because I’d never recognized the value of having a person who is not a practicing teacher in the school at the moment… the way that you’ve been able to involve yourself in the discussion and the activities that are going on and come through with some very well-made points at crucial times, but in small groups and large groups.” (p. 377)

##### ‘Collective reflexive monitoring’ to refine implementation

Reflexive monitoring could be a collective action oriented towards refining how an intervention was implemented [24, 33, 35]. For example, in the case of two interventions, over time staff in some schools opted to recruit fewer disengaged or disadvantaged students to participate in intervention activities [33, 36].

When external facilitators were removed in the Learning Together intervention, this resulted in the overall fidelity of implementation declining but some intervention components becoming mainstreamed so that their ‘form’ was modified at the same time as their ‘function’ became integrated within school policies and systems, as one evaluation [35] evaluation reported:

”Most interviewees suggested that external facilitation was not necessary in the final year, but a few suggested this was a significant loss: ’The absence of [facilitator] has been incredibly significant because she… was able to tie it in all the time to the agenda. And was a touchstone I suppose really for that. And then… so that… I think that was a loss’. (Senior leadership team member…)” (p.991)

##### Reflexive monitoring reinforcing implementation

Reflexive monitoring could reinforce the conditions necessary for further implementation [24, 26, 33, 36]. Staff and students recognised through processes of reflexive monitoring that interventions had diverse consequences for different parts of school systems, many of which were unanticipated. For example, an evaluation of the Gatehouse Project [24] reported:

“not only has the work of the adolescent health team facilitated reviews of organizational structure, but it has also contributed to a substantial shift in the perceptions of what is the core business of schools. [As one staff member reported:] ’But just really reinforcing the ideas of the positiveness and feeling secure at school, and certainly encouraging staff, that irrespective of what subject they teach, they can have an influence. And it’s a bit like planting a seed…’. There was also evidence of changing professional identity - teachers shifted their position from being a teacher of a subject or program to placing the young person and learning at the center of practice.” (p.379)

Similarly, involving students in decision-making or being surveyed about their needs could transform staff and student attitudes by suggesting that the school was becoming a more participative institution [26].

## Discussion

### Summary of key findings

We examined what factors relating to setting, population and intervention influence the implementation of whole-school interventions aiming to prevent substance use and violence via increasing student commitment. We used the General Theory of Implementation [15] as a framework to inform our analysis. This aligned strongly with our findings and provided us with a basis to summarise the school, population and intervention-related factors that influenced implementation. It enabled us to draw out which points in the process of implementation were affected by particular factors.

In terms of ‘sense-making’, evaluations suggested that school staff were more likely to understand what was required when provided with good-quality materials and ongoing support, a point also reported by previous reviews [10, 11]. A novel finding was that school staff could sometimes wilfully or unintentionally misinterpret intervention aims.

In terms of ‘cognitive participation’, evaluations suggested that various factors influenced whether school staff were prepared to commit to enacting intervention activities. As identified in previous reviews [10, 11], staff assessed interventions in terms of their ease of integration with existing practices. A novel finding from our review was that intervention lack of local adaptability was particularly undermining for whole-school elements, such as proposed changes to school policies or discipline systems. Interventions providing local data was reported as helping build commitment. The factors affecting implement are influenced by the intervention theory of change. For whole-school interventions aiming to build student commitment to school, it is important that interventions are tailorable to school cultures and structures.

As identified in previous reviews of school-based interventions [10, 11], staff commitment to deliver an intervention was also reported to be affected by the school capacity. A novel finding from our review was that school leaders were more likely to commit to a whole-school intervention when this addressed an issue they were already interested in tackling, for example, providing a way to respond to a new government policy or inspection requirements. Schools were also more likely to commit when there was already a recognition of the need for change, for example because of poor exam results or a new head wanting to transform a school. Again, it appears that this factor is a more important influence on the implementation of interventions aiming to transform whole-school cultures and systems than classroom education interventions.

In terms of ‘collective action’, as found in previous reviews [10, 11], interventions needed to be locally workable if staff were to work together to deliver them: fitting with school timetables and providing staff and students with clear guidance. A novel finding which again appears to be specific to interventions aiming to transform whole-school organisation was that planning groups (consisting of staff and possibly also students, parents or other community-members) were reported as particularly successful in ensuring collective action to enact interventions. Diverse participation in such groups could support implementation by ensuring decisions were pragmatic and by maintaining commitment. Studies also reported that these groups could also help ensure that intervention activities added up to a coordinated process of integrated school transformation, rather than merely a disparate set of initiatives.

Another novel finding, which may be particularly important regarding multi-component whole-school interventions, was that better synergies between intervention components appeared to facilitate implementation. Synergy appeared to occur where the consequences of enacting one intervention activity (e.g. training or provision of local data) helped provide the conditions required for the implementation of other components.

The extent to which staff in schools could come together to collectively enact interventions also depended on local ‘capacity’ (available resources). As identified in previous reviews of implementing school health interventions [10, 11], time was in chronic short supply in many schools, which undermined staff’s ability to implement interventions. A novel finding from our review was that another key resource was whether those charged with leading whole-school interventions were empowered to do this. Such leadership resources could include the appropriate budget, the ability to direct other staff or the ability to modify policies or systems. Another key resource was whether school systems possessed strong connections between staff and cultures of innovation. Conversely, a culture of teacher autonomy could undermine collective action with regard to whole-school work.

In terms of ‘reflexive monitoring’ (review of implementation), an important new finding specific to implementation of whole-school interventions was that it took time to achieve whole-school change as school staff and students gradually came to define their roles in intervention processes and develop the commitment and cognitive capacity they needed to effectively facilitate implementation. Reflexive monitoring, whether through formal or informal processes, was therefore critically important in determining the extent to which implementation built or dissipated over time. Interventions were particularly successful when they included an action group that oversaw delivery and reflected on the results. These processes enabled members to reflexively monitor what was being enacted and with what consequences. This could give participants the permission and resources to try different things, persist with what was perceived as working and refine or reject what was perceived to go less well. ‘Quick wins’ evidencing positive outcomes could help maintain collective impetus to implement further intervention activities. Ongoing support from training, facilitation or coaching could also support reflexive monitoring by providing an opportunity for reflection and/or an outsider perspective.

### Limitations

The original review searches involved multiple sources and methods, and aimed to maximise sensitivity. However, the updated searches were necessarily narrower because of the limits imposed by the Covid-19 pandemic. However, the sources that yielded all of the included study reports found as a result of the original electronic searches were included in the updated searches so we think it unlikely that any studies were missed because of this reduced scope. We synthesised qualitative research on implementation processes and thus any assessments of implementation are based on the assessments of those interviewed. It did not aim to synthesise quantitative evidence on implementation fidelity. Our process evaluation synthesis was limited by the size and quality of eligible reports. Study quality was mixed with a minority assessed as of high reliability and usefulness. We acknowledge that studies in which some of the authors of this review were involved were assessed as relatively high quality. Rather than this reflecting bias in our assessments, we think this reflects the fact that our design of the evaluations in question was informed by the quality assessment criteria used in this review [19]. Our review was not able to distinguish factors influencing implementation in primary versus secondary schools because studies were conducted in a diversity of school systems with different age bands.

## Conclusion

Whole-school interventions to prevent substance use and violence are feasible to implement in schools. Because good implementation is so critical to intervention effectiveness [12], interventions need to be designed so that they can be delivered with strong fidelity and so achieve significant public-health benefits. Our review suggests that interventions should be optimised by designing them to be maximally implementable, for example by providing good guidance, data on local needs and developing collaborative coordinating bodies. However, there is a lack of evidence from low-income countries and we cannot be confident that our findings apply to such settings. Future evaluations of whole-school interventions need to occur across different settings and should include process evaluations to examine intervention acceptability and fidelity as well as factors affecting this. This should contribute to intervention refinements and inform assessments of potential intervention transferability to other settings and populations. The wider review of which this review of implementation is one element will report on the effectiveness of such interventions in preventing substance use and violence. The quality of process evaluations of the sort reviewed here could be improved by evaluators referring to quality-assessment tools such as those used in this review [19].

## Supplementary Information

**Additional file 1.** PRISMA checklist.

**Additional file 2: Appendix 1.** Full search terms and strategies: 2020 search.

## Data Availability

The data are all available in the public realm. All research materials are available on request.

## Abbreviations

DARE: Drug Abuse Resistance Education
UK: United Kingdom
USA: United States of America
