# Supplementary material for: What factors influence implementation of whole-school interventions aiming to promote student commitment to school to prevent substance use and violence? Systematic review and synthesis of process evaluations
Source: BMC Public Health. 2022 Nov 22;22:2148. doi: 10.1186/s12889-022-14544-4 (PMC9682645; doi:10.1186/s12889-022-14544-4)
Supplement: Supplementary file 2 — Additional file 2: Appendix 1. Full search terms and strategies: 2020 search. [file 12889_2022_14544_MOESM2_ESM.docx]

**Appendix 1: Full search terms and strategies: 2020 search**

This provides full details of search strings used for bibliographic databases and trials registers, with dates and number of references returned and notes explaining any unusual search techniques or syntax. The EndNote X9 import order is provided, as the deduplication technique keeps the first uploaded copy of the reference by default.

In all searches, numbers in parentheses at the end of each row show the number of hits retrieved.

## OvidSP Medline

| Database name | Medline |
| --- | --- |
| Database platform | OvidSP |
| Dates of database coverage | Ovid MEDLINE ALL 1946 to January 14, 2020 |
| Date searched | 16/01/2020 |
| Searched by | JF |
| Number of results | 8646 |
| EndNote import order | 1 |
| Number of results once duplicates removed | 8007 |
| Search strategy notes | This is the search that all other database searches are based on.  Search lines ending in a ‘/’ are subject heading searches. Search lines ending in ‘/*ab*’ are subject headings with subheadings added. The subheading is listed in square brackets at the end of the line. Search lines beginning ‘exp’ are exploded subject heading searches. Search lines ending in .ti,ab,kf. search in the title, abstract and author keywords only. Search lines ending in .jw. search in the journal title only. or/*x-y* combines search sets in the range *x-y* with Boolean operator OR. * is used for truncation of words. # is used for a compulsory wildcard. ? is used for an optional wildcard. adj*n* searches for terms within *n* words or each other. Words in square brackets [ ] are comments and are not included in the search terms. |

1. child/ (1655207)
2. adolescent/ (1984969)
3. Minors/ (2552)
4. Students/ (56190)
5. School Teachers/ (1166)
6. exp Parents/ (108859)
7. child*.ti,ab,kf. (1390425)
8. boy?.ti,ab,kf. (141695)
9. girl?.ti,ab,kf. (144346)
10. (schoolage or (school adj1 age)).ti,ab,kf. (13320)
11. schoolchild*.ti,ab,kf. (13629)
12. youngster?.ti,ab,kf. (2479)
13. minor?.ti,ab,kf. (222311)
14. (pupil* or student*).ti,ab,kf. not (pupil/ [removes items about eyes] or exp Students, Health Occupations/) (239994)
15. prepubescen*.ti,ab,kf. (984)
16. pubescent*.ti,ab,kf. (840)
17. adolescent*.ti,ab,kf. (248192)
18. juvenil*.ti,ab,kf. (81133)
19. underage*.ti,ab,kf. (1200)
20. (preteen* or pre-teen*).ti,ab,kf. (473)
21. (teen or teens).ti,ab,kf. (10506)
22. teenage*.ti,ab,kf. (20921)
23. (youth or youths).ti,ab,kf. (75360)
24. young person*.ti,ab,kf. (3407)
25. young people*.ti,ab,kf. (27110)
26. (kid or kids).ti,ab,kf. (7577)
27. (transition adj4 adult*).ti,ab,kf. (4263)
28. emerging adult*.ti,ab,kf. (2387)
29. young adult*.ti,ab,kf. (92072)
30. parent?.ti,ab,kf. (263293)
31. mother?.ti,ab,kf. (210197)
32. father?.ti,ab,kf. (39584)
33. guardian?.ti,ab,kf. (6714)
34. teacher?.ti,ab,kf. (42958)
35. child*.jw. (152768)
36. adolescen*.jw. (43811)
37. youth*.jw. (3546)
38. school*.jw. (17580)
39. (("5" or "6" or "7" or "8" or "9" or "10" or "11" or "12" or "13" or "14" or "15" or "16" or "17" or "18") adj (year* old or year* of age)).ti,ab,kf. (216660)
40. ((five or six or seven or eight or nine or ten or eleven or twelve or thirteen or fourteen or fifteen or sixteen or seventeen or eighteen) adj (year* old or year* of age)).ti,ab,kf. (18566)
41. (age* adj ("5" or "6" or "7" or "8" or "9" or "10" or "11" or "12" or "13" or "14" or "15" or "16" or "17" or "18") adj year*).ti,ab,kf. (43073)
42. (age* adj (five or six or seven or eight or nine or ten or eleven or twelve or thirteen or fourteen or fifteen or sixteen or seventeen or eighteen) adj year*).ti,ab,kf. (908)
43. ((primary or secondary or year) adj1 ("1" or "2" or "3" or "4" or "5" or "6" or "7" or "8" or "9" or "10" or "11" or "12" or "13")).ti,ab,kf. (596764)
44. (grade? adj1 (first or second or third or fourth or fifth or sixth or seventh or eighth or ninth or tenth or eleventh or twelfth)).ti,ab,kf. (10647)
45. or/1-44 [POPULATION] (4486751)
46. schools/og [Organization & Administration] (1533)
47. School Health Services/og [Organization & Administration] (3677)
48. (school? adj5 (based or level or wide or led or ethos or environment or organi?ation* or climate or toolkit? or approach* or action group? or practice? or whole or health* or leader* or cultur* or manag* or governance or system? or communit*)).ti,ab,kf. (58044)
49. schoolwide.ti,ab,kf. (95)
50. restor* justice.ti,ab,kf. (136)
51. Schools/ (36711)
52. school?.ti,ab,kf,jw. (272029)
53. or/51-52 (276848)
54. "peer-to-peer".ti,ab,kf. (1242)
55. (peer adj2 (led or leader* or participat* or mediat* or helper? or resistan* or wide or action? or influen*)).ti,ab,kf. (3830)
56. (relationship* adj2 (led or leader* or participat* or mediat* or helper? or resistan* or wide or action? or influen*)).ti,ab,kf. (14525)
57. Community Networks/ (6788)
58. Community Participation/ (16843)
59. ((community or communities) adj2 (led or leader* or participat* or mediat* or helper? or resistan* or wide or action? or influen* or relationship*)).ti,ab,kf. (20747)
60. (pupil? adj2 (led or leader* or participat* or mediat* or helper? or resistan* or wide or action? or influen* or relationship*)).ti,ab,kf. not pupil/ [removes items about eyes] (265)
61. ((child or children) adj2 (led or leader* or participat* or mediat* or helper? or resistan* or wide or action? or influen*)).ti,ab,kf. (12891)
62. ((young people or young person) adj2 (led or leader* or participat* or mediat* or helper? or resistan* or wide or action? or influen*)).ti,ab,kf. (279)
63. (teen* adj2 (led or leader* or participat* or mediat* or helper? or resistan* or wide or action? or influen*)).ti,ab,kf. (377)
64. (adolescent* adj2 (led or leader* or participat* or mediat* or helper? or resistan* or wide or action? or influen*)).ti,ab,kf. (4404)
65. (youth? adj2 (led or leader* or participat* or mediat* or helper? or resistan* or wide or action? or influen*)).ti,ab,kf. (1835)
66. (student? adj2 (led or leader* or participat* or mediat* or helper? or resistan* or wide or action? or influen* or relationship*)).ti,ab,kf. (13001)
67. (teach* adj2 (led or leader* or participat* or mediat* or helper? or resistan* or wide or action? or influen* or relationship*)).ti,ab,kf. (3217)
68. (curricul* adj2 (led or leader* or participat* or mediat* or helper? or resistan* or wide or action? or influen* or relationship*)).ti,ab,kf. (724)
69. (administrat* adj2 (led or leader* or participat* or mediat* or helper? or resistan* or wide or action? or influen* or relationship*)).ti,ab,kf. (4873)
70. ((personnel or staff) adj2 (led or leader* or participat* or mediat* or helper? or resistan* or wide or action? or influen* or relationship*)).ti,ab,kf. (4164)
71. (volunteer* adj2 (led or leader* or participat* or mediat* or helper? or resistan* or wide or action? or influen* or relationship*)).ti,ab,kf. (5663)
72. ((advocacy or advocate) adj2 (led or leader* or participat* or mediat* or helper? or resistan* or wide or action? or influen* or relationship*)).ti,ab,kf. (706)
73. (parent* adj2 (led or leader* or participat* or mediat* or helper? or resistan* or wide or action? or influen*)).ti,ab,kf. (9466)
74. Social Environment/ (42305)
75. (environment* adj2 (led or leader* or participat* or mediat* or helper? or resistan* or wide or action? or influen* or relationship*)).ti,ab,kf. (27216)
76. (socio-ecolog* adj2 (led or leader* or participat* or mediat* or helper? or resistan* or wide or action? or influen* or relationship*)).ti,ab,kf. (33)
77. social support/ (68775)
78. social learning/ (373)
79. (learn* adj2 (led or leader* or participat* or mediat* or helper? or resistan* or wide or action? or influen* or relationship*)).ti,ab,kf. (6486)
80. ((policy or policies) adj2 (led or leader* or participat* or mediat* or helper? or resistan* or wide or action? or influen* or relationship*)).ti,ab,kf. (5478)
81. or/54-80 (280269)
82. 53 and 81 (24534)
83. or/46-50,82 [SCHOOL-WIDE INTERVENTION] (76066)
84. Smoking Prevention/ (17855)
85. exp Tobacco Smoking/pc [Prevention & Control] (119)
86. Vaping/pc [Prevention & Control] (11)
87. Marijuana Smoking/pc [Prevention & Control] (263)
88. smoking cessation/ (27807)
89. "tobacco use cessation"/ (1132)
90. smoking reduction/ (43)
91. ((smoking or smoked or smoke or smoker) adj5 (reduc* or decreas* or increas* or lower* or fewer or improv* or enhance* or extend* or develop* or prevalen* or daily or weekly or current or rate or rates or behavio?r* or knowledge or prevent* or attitude* or avoid* or perceive* or percept* or promot* or program* or change*)).ti,ab,kf. (78201)
92. (tobacco adj5 (reduc* or decreas* or increas* or lower* or fewer or improv* or enhance* or extend* or develop* or prevalen* or daily or weekly or current or rate or rates or behavio?r* or knowledge or prevent* or attitude* or avoid* or perceive* or percept* or promot* or program* or change*)).ti,ab,kf. (25194)
93. (cigarette? adj5 (reduc* or decreas* or increas* or lower* or fewer or improv* or enhance* or extend* or develop* or prevalen* or daily or weekly or current or rate or rates or behavio?r* or knowledge or prevent* or attitude* or avoid* or perceive* or percept* or promot* or program* or change*)).ti,ab,kf. (20899)
94. (marijuana adj5 (reduc* or decreas* or increas* or lower* or fewer or improv* or enhance* or extend* or develop* or prevalen* or daily or weekly or current or rate or rates or behavio?r* or knowledge or prevent* or attitude* or avoid* or perceive* or percept* or promot* or program* or change*)).ti,ab,kf. (3501)
95. (cannabis adj5 (reduc* or decreas* or increas* or lower* or fewer or improv* or enhance* or extend* or develop* or prevalen* or daily or weekly or current or rate or rates or behavio?r* or knowledge or prevent* or attitude* or avoid* or perceive* or percept* or promot* or program* or change*)).ti,ab,kf. (4524)
96. (solvent? adj5 (reduc* or decreas* or increas* or lower* or fewer or improv* or enhance* or extend* or develop* or prevalen* or daily or weekly or current or rate or rates or behavio?r* or knowledge or prevent* or attitude* or avoid* or perceive* or percept* or promot* or program* or change*)).ti,ab,kf. (18959)
97. exp Substance-Related Disorders/pc [Prevention & Control] (22138)
98. Alcohol Drinking/pc [Prevention & Control] (4358)
99. Binge Drinking/pc [Prevention & Control] (186)
100. Underage Drinking/pc [Prevention & Control] (146)
101. (alcohol adj5 (reduc* or decreas* or increas* or lower* or fewer or improv* or enhance* or extend* or develop* or prevalen* or daily or weekly or current or rate or rates or behavio?r* or knowledge or prevent* or attitude* or avoid* or perceive* or percept* or promot* or program* or change*)).ti,ab,kf. (62670)
102. (binge drink* adj5 (reduc* or decreas* or increas* or lower* or fewer or improv* or enhance* or extend* or develop* or prevalen* or daily or weekly or current or rate or rates or behavio?r* or knowledge or prevent* or attitude* or avoid* or perceive* or percept* or promot* or program* or change*)).ti,ab,kf. (1904)
103. (underage drink* adj5 (reduc* or decreas* or increas* or lower* or fewer or improv* or enhance* or extend* or develop* or prevalen* or daily or weekly or current or rate or rates or behavio?r* or knowledge or prevent* or attitude* or avoid* or perceive* or percept* or promot* or program* or change*)).ti,ab,kf. (226)
104. (substance? adj5 (reduc* or decreas* or increas* or lower* or fewer or improv* or enhance* or extend* or develop* or prevalen* or daily or weekly or current or rate or rates or behavio?r* or knowledge or prevent* or attitude* or avoid* or perceive* or percept* or promot* or program* or change*)).ti,ab,kf. (45271)
105. (drug? adj5 (reduc* or decreas* or increas* or lower* or fewer or improv* or enhance* or extend* or develop* or prevalen* or daily or weekly or current or rate or rates or behavio?r* or knowledge or prevent* or attitude* or avoid* or perceive* or percept* or promot* or program* or change*)).ti,ab,kf. (343301)
106. exp Academic Performance/ (1591)
107. (education* attain* adj5 (reduc* or decreas* or increas* or lower* or fewer or improv* or enhance* or extend* or develop* or prevalen* or daily or weekly or current or rate or rates or behavio?r* or knowledge or prevent* or attitude* or avoid* or perceive* or percept* or promot* or program* or change*)).ti,ab,kf. (2326)
108. (academic attain* adj5 (reduc* or decreas* or increas* or lower* or fewer or improv* or enhance* or extend* or develop* or prevalen* or daily or weekly or current or rate or rates or behavio?r* or knowledge or prevent* or attitude* or avoid* or perceive* or percept* or promot* or program* or change*)).ti,ab,kf. (59)
109. (student* attain* adj5 (reduc* or decreas* or increas* or lower* or fewer or improv* or enhance* or extend* or develop* or prevalen* or daily or weekly or current or rate or rates or behavio?r* or knowledge or prevent* or attitude* or avoid* or perceive* or percept* or promot* or program* or change*)).ti,ab,kf. (21)
110. (education* achieve* adj5 (reduc* or decreas* or increas* or lower* or fewer or improv* or enhance* or extend* or develop* or prevalen* or daily or weekly or current or rate or rates or behavio?r* or knowledge or prevent* or attitude* or avoid* or perceive* or percept* or promot* or program* or change*)).ti,ab,kf. (384)
111. (academic achieve* adj5 (reduc* or decreas* or increas* or lower* or fewer or improv* or enhance* or extend* or develop* or prevalen* or daily or weekly or current or rate or rates or behavio?r* or knowledge or prevent* or attitude* or avoid* or perceive* or percept* or promot* or program* or change*)).ti,ab,kf. (1481)
112. (student* achieve* adj5 (reduc* or decreas* or increas* or lower* or fewer or improv* or enhance* or extend* or develop* or prevalen* or daily or weekly or current or rate or rates or behavio?r* or knowledge or prevent* or attitude* or avoid* or perceive* or percept* or promot* or program* or change*)).ti,ab,kf. (226)
113. (education* perform* adj5 (reduc* or decreas* or increas* or lower* or fewer or improv* or enhance* or extend* or develop* or prevalen* or daily or weekly or current or rate or rates or behavio?r* or knowledge or prevent* or attitude* or avoid* or perceive* or percept* or promot* or program* or change*)).ti,ab,kf. (102)
114. (academic perform* adj5 (reduc* or decreas* or increas* or lower* or fewer or improv* or enhance* or extend* or develop* or prevalen* or daily or weekly or current or rate or rates or behavio?r* or knowledge or prevent* or attitude* or avoid* or perceive* or percept* or promot* or program* or change*)).ti,ab,kf. (1774)
115. (student* perform* adj5 (reduc* or decreas* or increas* or lower* or fewer or improv* or enhance* or extend* or develop* or prevalen* or daily or weekly or current or rate or rates or behavio?r* or knowledge or prevent* or attitude* or avoid* or perceive* or percept* or promot* or program* or change*)).ti,ab,kf. (941)
116. (education* outcome? adj5 (reduc* or decreas* or increas* or lower* or fewer or improv* or enhance* or extend* or develop* or prevalen* or daily or weekly or current or rate or rates or behavio?r* or knowledge or prevent* or attitude* or avoid* or perceive* or percept* or promot* or program* or change*)).ti,ab,kf. (594)
117. (academic outcome? adj5 (reduc* or decreas* or increas* or lower* or fewer or improv* or enhance* or extend* or develop* or prevalen* or daily or weekly or current or rate or rates or behavio?r* or knowledge or prevent* or attitude* or avoid* or perceive* or percept* or promot* or program* or change*)).ti,ab,kf. (287)
118. (student* outcome? adj5 (reduc* or decreas* or increas* or lower* or fewer or improv* or enhance* or extend* or develop* or prevalen* or daily or weekly or current or rate or rates or behavio?r* or knowledge or prevent* or attitude* or avoid* or perceive* or percept* or promot* or program* or change*)).ti,ab,kf. (203)
119. (education* commitment adj5 (reduc* or decreas* or increas* or lower* or fewer or improv* or enhance* or extend* or develop* or prevalen* or daily or weekly or current or rate or rates or behavio?r* or knowledge or prevent* or attitude* or avoid* or perceive* or percept* or promot* or program* or change*)).ti,ab,kf. (8)
120. (academic commitment adj5 (reduc* or decreas* or increas* or lower* or fewer or improv* or enhance* or extend* or develop* or prevalen* or daily or weekly or current or rate or rates or behavio?r* or knowledge or prevent* or attitude* or avoid* or perceive* or percept* or promot* or program* or change*)).ti,ab,kf. (6)
121. (student* commitment adj5 (reduc* or decreas* or increas* or lower* or fewer or improv* or enhance* or extend* or develop* or prevalen* or daily or weekly or current or rate or rates or behavio?r* or knowledge or prevent* or attitude* or avoid* or perceive* or percept* or promot* or program* or change*)).ti,ab,kf. (15)
122. (education* engage* adj5 (reduc* or decreas* or increas* or lower* or fewer or improv* or enhance* or extend* or develop* or prevalen* or daily or weekly or current or rate or rates or behavio?r* or knowledge or prevent* or attitude* or avoid* or perceive* or percept* or promot* or program* or change*)).ti,ab,kf. (12)
123. (academic engage* adj5 (reduc* or decreas* or increas* or lower* or fewer or improv* or enhance* or extend* or develop* or prevalen* or daily or weekly or current or rate or rates or behavio?r* or knowledge or prevent* or attitude* or avoid* or perceive* or percept* or promot* or program* or change*)).ti,ab,kf. (82)
124. (student* engage* adj5 (reduc* or decreas* or increas* or lower* or fewer or improv* or enhance* or extend* or develop* or prevalen* or daily or weekly or current or rate or rates or behavio?r* or knowledge or prevent* or attitude* or avoid* or perceive* or percept* or promot* or program* or change*)).ti,ab,kf. (515)
125. (dropout adj5 (reduc* or decreas* or increas* or lower* or fewer or improv* or enhance* or extend* or develop* or prevalen* or daily or weekly or current or rate or rates or behavio?r* or knowledge or prevent* or attitude* or avoid* or perceive* or percept* or promot* or program* or change*)).ti,ab,kf. (4247)
126. (absentee* adj5 (reduc* or decreas* or increas* or lower* or fewer or improv* or enhance* or extend* or develop* or prevalen* or daily or weekly or current or rate or rates or behavio?r* or knowledge or prevent* or attitude* or avoid* or perceive* or percept* or promot* or program* or change*)).ti,ab,kf. (2035)
127. Violence/pc [Prevention & Control] (5930)
128. Aggression/pc [Prevention & Control] (16)
129. Physical Abuse/pc [Prevention & Control] (67)
130. Gender-Based Violence/pc [Prevention & Control] (45)
131. Gun Violence/pc [Prevention & Control] (51)
132. exp Bullying/pc [Prevention & Control] (368)
133. ((violent or violence) adj5 (reduc* or decreas* or increas* or lower* or fewer or improv* or enhance* or extend* or develop* or prevalen* or daily or weekly or current or rate or rates or behavio?r* or knowledge or prevent* or attitude* or avoid* or perceive* or percept* or promot* or program* or change*)).ti,ab,kf. (19561)
134. ((aggression or aggressive) adj5 (reduc* or decreas* or increas* or lower* or fewer or improv* or enhance* or extend* or develop* or prevalen* or daily or weekly or current or rate or rates or behavio?r* or knowledge or prevent* or attitude* or avoid* or perceive* or percept* or promot* or program* or change*)).ti,ab,kf. (44488)
135. ((hostile or hostility) adj5 (reduc* or decreas* or increas* or lower* or fewer or improv* or enhance* or extend* or develop* or prevalen* or daily or weekly or current or rate or rates or behavio?r* or knowledge or prevent* or attitude* or avoid* or perceive* or percept* or promot* or program* or change*)).ti,ab,kf. (2712)
136. (assault* adj5 (reduc* or decreas* or increas* or lower* or fewer or improv* or enhance* or extend* or develop* or prevalen* or daily or weekly or current or rate or rates or behavio?r* or knowledge or prevent* or attitude* or avoid* or perceive* or percept* or promot* or program* or change*)).ti,ab,kf. (2659)
137. (crime adj5 (reduc* or decreas* or increas* or lower* or fewer or improv* or enhance* or extend* or develop* or prevalen* or daily or weekly or current or rate or rates or behavio?r* or knowledge or prevent* or attitude* or avoid* or perceive* or percept* or promot* or program* or change*)).ti,ab,kf. (3161)
138. ((abuse? or abusive) adj5 (reduc* or decreas* or increas* or lower* or fewer or improv* or enhance* or extend* or develop* or prevalen* or daily or weekly or current or rate or rates or behavio?r* or knowledge or prevent* or attitude* or avoid* or perceive* or percept* or promot* or program* or change*)).ti,ab,kf. (27598)
139. (misbehav* adj5 (reduc* or decreas* or increas* or lower* or fewer or improv* or enhance* or extend* or develop* or prevalen* or daily or weekly or current or rate or rates or behavio?r* or knowledge or prevent* or attitude* or avoid* or perceive* or percept* or promot* or program* or change*)).ti,ab,kf. (139)
140. (threat? adj5 (reduc* or decreas* or increas* or lower* or fewer or improv* or enhance* or extend* or develop* or prevalen* or daily or weekly or current or rate or rates or behavio?r* or knowledge or prevent* or attitude* or avoid* or perceive* or percept* or promot* or program* or change*)).ti,ab,kf. (12884)
141. (conduct adj5 (reduc* or decreas* or increas* or lower* or fewer or improv* or enhance* or extend* or develop* or prevalen* or daily or weekly or current or rate or rates or behavio?r* or knowledge or prevent* or attitude* or avoid* or perceive* or percept* or promot* or program* or change*)).ti,ab,kf. (8142)
142. (delinquen* adj5 (reduc* or decreas* or increas* or lower* or fewer or improv* or enhance* or extend* or develop* or prevalen* or daily or weekly or current or rate or rates or behavio?r* or knowledge or prevent* or attitude* or avoid* or perceive* or percept* or promot* or program* or change*)).ti,ab,kf. (2863)
143. (disrupt* behavio?r* adj5 (reduc* or decreas* or increas* or lower* or fewer or improv* or enhance* or extend* or develop* or prevalen* or daily or weekly or current or rate or rates or behavio?r* or knowledge or prevent* or attitude* or avoid* or perceive* or percept* or promot* or program* or change*)).ti,ab,kf. (3685)
144. (problem* behavio?r* adj5 (reduc* or decreas* or increas* or lower* or fewer or improv* or enhance* or extend* or develop* or prevalen* or daily or weekly or current or rate or rates or behavio?r* or knowledge or prevent* or attitude* or avoid* or perceive* or percept* or promot* or program* or change*)).ti,ab,kf. (5752)
145. (volatile behavio?r* adj5 (reduc* or decreas* or increas* or lower* or fewer or improv* or enhance* or extend* or develop* or prevalen* or daily or weekly or current or rate or rates or behavio?r* or knowledge or prevent* or attitude* or avoid* or perceive* or percept* or promot* or program* or change*)).ti,ab,kf. (22)
146. ((antisocial or anti-social) adj5 (reduc* or decreas* or increas* or lower* or fewer or improv* or enhance* or extend* or develop* or prevalen* or daily or weekly or current or rate or rates or behavio?r* or knowledge or prevent* or attitude* or avoid* or perceive* or percept* or promot* or program* or change*)).ti,ab,kf. (5199)
147. (perpetrat* adj5 (reduc* or decreas* or increas* or lower* or fewer or improv* or enhance* or extend* or develop* or prevalen* or daily or weekly or current or rate or rates or behavio?r* or knowledge or prevent* or attitude* or avoid* or perceive* or percept* or promot* or program* or change*)).ti,ab,kf. (1800)
148. (bully* adj5 (reduc* or decreas* or increas* or lower* or fewer or improv* or enhance* or extend* or develop* or prevalen* or daily or weekly or current or rate or rates or behavio?r* or knowledge or prevent* or attitude* or avoid* or perceive* or percept* or promot* or program* or change*)).ti,ab,kf. (2448)
149. (victimi* adj5 (reduc* or decreas* or increas* or lower* or fewer or improv* or enhance* or extend* or develop* or prevalen* or daily or weekly or current or rate or rates or behavio?r* or knowledge or prevent* or attitude* or avoid* or perceive* or percept* or promot* or program* or change*)).ti,ab,kf. (3271)
150. (cyberbully* adj5 (reduc* or decreas* or increas* or lower* or fewer or improv* or enhance* or extend* or develop* or prevalen* or daily or weekly or current or rate or rates or behavio?r* or knowledge or prevent* or attitude* or avoid* or perceive* or percept* or promot* or program* or change*)).ti,ab,kf. (299)
151. (conflict* adj5 (reduc* or decreas* or increas* or lower* or fewer or improv* or enhance* or extend* or develop* or prevalen* or daily or weekly or current or rate or rates or behavio?r* or knowledge or prevent* or attitude* or avoid* or perceive* or percept* or promot* or program* or change*)).ti,ab,kf. (16652)
152. (positive behavio?r* adj5 (reduc* or decreas* or increas* or lower* or fewer or improv* or enhance* or extend* or develop* or prevalen* or daily or weekly or current or rate or rates or behavio?r* or knowledge or prevent* or attitude* or avoid* or perceive* or percept* or promot* or program* or change*)).ti,ab,kf. (1535)
153. (improv* behavio?r* adj5 (reduc* or decreas* or increas* or lower* or fewer or improv* or enhance* or extend* or develop* or prevalen* or daily or weekly or current or rate or rates or behavio?r* or knowledge or prevent* or attitude* or avoid* or perceive* or percept* or promot* or program* or change*)).ti,ab,kf. (2184)
154. (social behavio?r* adj5 (reduc* or decreas* or increas* or lower* or fewer or improv* or enhance* or extend* or develop* or prevalen* or daily or weekly or current or rate or rates or behavio?r* or knowledge or prevent* or attitude* or avoid* or perceive* or percept* or promot* or program* or change*)).ti,ab,kf. (15003)
155. ((social-emotion* or socio-emotion*) adj5 (reduc* or decreas* or increas* or lower* or fewer or improv* or enhance* or extend* or develop* or prevalen* or daily or weekly or current or rate or rates or behavio?r* or knowledge or prevent* or attitude* or avoid* or perceive* or percept* or promot* or program* or change*)).ti,ab,kf. (1940)
156. (social-charact* adj5 (reduc* or decreas* or increas* or lower* or fewer or improv* or enhance* or extend* or develop* or prevalen* or daily or weekly or current or rate or rates or behavio?r* or knowledge or prevent* or attitude* or avoid* or perceive* or percept* or promot* or program* or change*)).ti,ab,kf. (238)
157. (social-inclusion adj5 (reduc* or decreas* or increas* or lower* or fewer or improv* or enhance* or extend* or develop* or prevalen* or daily or weekly or current or rate or rates or behavio?r* or knowledge or prevent* or attitude* or avoid* or perceive* or percept* or promot* or program* or change*)).ti,ab,kf. (392)
158. (social develop* adj5 (reduc* or decreas* or increas* or lower* or fewer or improv* or enhance* or extend* or develop* or prevalen* or daily or weekly or current or rate or rates or behavio?r* or knowledge or prevent* or attitude* or avoid* or perceive* or percept* or promot* or program* or change*)).ti,ab,kf. (4336)
159. (social competen* adj5 (reduc* or decreas* or increas* or lower* or fewer or improv* or enhance* or extend* or develop* or prevalen* or daily or weekly or current or rate or rates or behavio?r* or knowledge or prevent* or attitude* or avoid* or perceive* or percept* or promot* or program* or change*)).ti,ab,kf. (1197)
160. (emotion* develop* adj5 (reduc* or decreas* or increas* or lower* or fewer or improv* or enhance* or extend* or develop* or prevalen* or daily or weekly or current or rate or rates or behavio?r* or knowledge or prevent* or attitude* or avoid* or perceive* or percept* or promot* or program* or change*)).ti,ab,kf. (1674)
161. (conduct adj5 (reduc* or decreas* or increas* or lower* or fewer or improv* or enhance* or extend* or develop* or prevalen* or daily or weekly or current or rate or rates or behavio?r* or knowledge or prevent* or attitude* or avoid* or perceive* or percept* or promot* or program* or change*)).ti,ab,kf. (8142)
162. (respect adj5 (reduc* or decreas* or increas* or lower* or fewer or improv* or enhance* or extend* or develop* or prevalen* or daily or weekly or current or rate or rates or behavio?r* or knowledge or prevent* or attitude* or avoid* or perceive* or percept* or promot* or program* or change*)).ti,ab,kf. (37971)
163. ((safety or safe) adj5 (reduc* or decreas* or increas* or lower* or fewer or improv* or enhance* or extend* or develop* or prevalen* or daily or weekly or current or rate or rates or behavio?r* or knowledge or prevent* or attitude* or avoid* or perceive* or percept* or promot* or program* or change*)).ti,ab,kf. (118094)
164. or/84-163 [BEHAVIOUR CHANGE] (887343)
165. exp Program Evaluation/ (73976)
166. Evaluation Studies/ (247878)
167. Validation Studies/ (97924)
168. exp Clinical Trial/ (849209)
169. prevent*.ti,ab,kf. (1393162)
170. program*.ti,ab,kf. (857378)
171. intervent*.ti,ab,kf. (963446)
172. restorative approach*.ti,ab,kf. (220)
173. control school?.ti,ab,kf. (855)
174. (trial or trials).ti,ab,kf. (994535)
175. or/165-174 [QUANTITATIVE STUDY] (4308598)
176. 45 and 83 and 164 and 175 (8646)
177. limit 176 to medline (7455) [MEDLINE records]
178. 176 not 177 (1191) [MEDLINE IN PROCESS and OTHERS records]

## ProQuest Applied Social Sciences Index & Abstract (ASSIA)

| Database name | Applied Social Sciences Index and Abstracts (ASSIA) |
| --- | --- |
| Database platform | ProQuest |
| Dates of database coverage | Complete database to date searched. |
| Date searched | 23/01/2020 |
| Searched by | JF |
| Number of results | 7627 |
| EndNote import order | 15 |
| Number of results once duplicates removed | 3444 |
| Search strategy notes | Due to limitations with the search interface, this search only includes terms for Concept 1 (population) and any school-wide intervention.  * is used for truncation. ? is used for a mandatory wildcard. Search strings starting with TI search the title only. Search strings starting with AB search the abstract only. NEAR/*n* search for terms within *n* words of each other, in any order. PRE/*n* search for terms within *n* words of each other in the order entered. |

(ti((school* NEAR/5 (based OR level OR wide OR led OR ethos OR environment OR organi?ation* OR climate OR toolkit OR approach OR action PRE/0 group OR practice OR whole OR health* OR leader OR cultur* OR manag* OR governance OR system* OR communit*)) OR schoolwide OR restor* PRE/0 justice) OR ab((school* NEAR/5 (based OR level OR wide OR led OR ethos OR environment OR organi?ation* OR climate OR toolkit OR approach OR action PRE/0 group OR practice OR whole OR health* OR leader OR cultur* OR manag* OR governance OR system* OR communit*)) OR schoolwide OR restor* PRE/0 justice) OR ((ti("peer-to-peer" OR ((peer OR pupil OR child OR children OR young PRE/0 people OR young PRE/0 person OR teen* OR adolescent* OR youth* OR student OR parent OR teach* OR curricul* OR administrat* OR personnel OR staff OR volunteer OR learn* OR advocacy OR advocate OR environment OR socio-ecolog* OR social PRE/0 support OR policy OR policies) NEAR/2 (led OR leader OR participat* OR mediat* OR helper* OR resistan* OR wide OR action* OR influen*))) OR ab("peer-to-peer" OR ((peer OR pupil OR child OR children OR young PRE/0 people OR young PRE/0 person OR teen* OR adolescent* OR youth* OR student OR parent OR teach* OR curricul* OR administrat* OR personnel OR staff OR volunteer OR learn* OR advocacy OR advocate OR environment OR socio-ecolog* OR social PRE/0 support OR policy OR policies) NEAR/2 (led OR leader OR participat* OR mediat* OR helper* OR resistan* OR wide OR action* OR influen*)))) AND noft(school*))) AND (ti(prevent* OR program* OR intervent* OR restorative PRE/0 approach* OR control PRE/0 school* OR trial OR trials) OR ab(prevent* OR program* OR intervent* OR restorative PRE/0 approach* OR control PRE/0 school* OR trial OR trials)) AND (ti(child OR boy OR girl OR schoolage OR school PRE/0 age OR schoolchild OR youngster OR minor OR pupil OR student OR prepubescent OR pubescent OR adolescent OR juvenile OR underage OR preteen OR pre-teen OR teen OR teens OR teenage* OR youth OR youths OR young PRE/0 person OR young PRE/0 people OR (transition NEAR/4 adult) OR emerging adult OR young adult OR parent OR mother OR father OR guardian OR teacher) OR ab(child OR boy OR girl OR schoolage OR school PRE/0 age OR schoolchild OR youngster OR minor OR pupil OR student OR prepubescent OR pubescent OR adolescent OR juvenile OR underage OR preteen OR pre-teen OR teen OR teens OR teenage* OR youth OR youths OR young PRE/0 person OR young PRE/0 people OR (transition NEAR/4 adult) OR emerging adult OR young adult OR parent OR mother OR father OR guardian OR teacher))

## ProQuest Australian Educational Index

| Database name | Australian Educational Index |
| --- | --- |
| Database platform | ProQuest |
| Dates of database coverage | Complete database to date searched. |
| Date searched | 23/01/2020 |
| Searched by | JF |
| Number of results | 4738 |
| EndNote import order | 16 |
| Number of results once duplicates removed | 4414 |
| Search strategy notes | Due to limitations with the search interface, this search only includes terms for Concept 1 (population) and any school-wide intervention.  * is used for truncation. ? is used for a mandatory wildcard. Search strings starting with TI search the title only. Search strings starting with AB search the abstract only. NEAR/*n* search for terms within *n* words of each other, in any order. PRE/*n* search for terms within *n* words of each other in the order entered. |

(ti((school* NEAR/5 (based OR level OR wide OR led OR ethos OR environment OR organi?ation* OR climate OR toolkit OR approach OR action PRE/0 group OR practice OR whole OR health* OR leader OR cultur* OR manag* OR governance OR system* OR communit*)) OR schoolwide OR restor* PRE/0 justice) OR ab((school* NEAR/5 (based OR level OR wide OR led OR ethos OR environment OR organi?ation* OR climate OR toolkit OR approach OR action PRE/0 group OR practice OR whole OR health* OR leader OR cultur* OR manag* OR governance OR system* OR communit*)) OR schoolwide OR restor* PRE/0 justice) OR ((ti("peer-to-peer" OR ((peer OR pupil OR child OR children OR young PRE/0 people OR young PRE/0 person OR teen* OR adolescent* OR youth* OR student OR parent OR teach* OR curricul* OR administrat* OR personnel OR staff OR volunteer OR learn* OR advocacy OR advocate OR environment OR socio-ecolog* OR social PRE/0 support OR policy OR policies) NEAR/2 (led OR leader OR participat* OR mediat* OR helper* OR resistan* OR wide OR action* OR influen*))) OR ab("peer-to-peer" OR ((peer OR pupil OR child OR children OR young PRE/0 people OR young PRE/0 person OR teen* OR adolescent* OR youth* OR student OR parent OR teach* OR curricul* OR administrat* OR personnel OR staff OR volunteer OR learn* OR advocacy OR advocate OR environment OR socio-ecolog* OR social PRE/0 support OR policy OR policies) NEAR/2 (led OR leader OR participat* OR mediat* OR helper* OR resistan* OR wide OR action* OR influen*)))) AND noft(school*))) AND (ti(prevent* OR program* OR intervent* OR restorative PRE/0 approach* OR control PRE/0 school* OR trial OR trials) OR ab(prevent* OR program* OR intervent* OR restorative PRE/0 approach* OR control PRE/0 school* OR trial OR trials)) AND (ti(child OR boy OR girl OR schoolage OR school PRE/0 age OR schoolchild OR youngster OR minor OR pupil OR student OR prepubescent OR pubescent OR adolescent OR juvenile OR underage OR preteen OR pre-teen OR teen OR teens OR teenage* OR youth OR youths OR young PRE/0 person OR young PRE/0 people OR (transition NEAR/4 adult) OR emerging adult OR young adult OR parent OR mother OR father OR guardian OR teacher) OR ab(child OR boy OR girl OR schoolage OR school PRE/0 age OR schoolchild OR youngster OR minor OR pupil OR student OR prepubescent OR pubescent OR adolescent OR juvenile OR underage OR preteen OR pre-teen OR teen OR teens OR teenage* OR youth OR youths OR young PRE/0 person OR young PRE/0 people OR (transition NEAR/4 adult) OR emerging adult OR young adult OR parent OR mother OR father OR guardian OR teacher))

## EBSCO British Education Index

| Database name | British Education Index |
| --- | --- |
| Database platform | EBSCO |
| Dates of database coverage | Complete database to date searched. |
| Date searched | 16/01/2020 |
| Searched by | JF |
| Number of results | 440 |
| EndNote import order | 9 |
| Number of results once duplicates removed | 199 |
| Search strategy notes | * is used for truncation. # is used for an optional wildcard. Search strings starting with TI search the title only. Search strings starting with AB search the abstract only. Search strings starting with SO search the journal title only. N/*n* search for terms within *n* words of each other, in any order. |

| Search number | Query | Results |
| --- | --- | --- |
| S1 | (TI child*) or (AB child*) | 30,737 |
| S2 | (TI boy#) or (AB boy#) | 2,303 |
| S3 | (TI girl#) or (AB girl#) | 2,760 |
| S4 | (TI (schoolage or (school N1 age))) or (AB (schoolage or (school N1 age))) | 428 |
| S5 | (TI schoolchild*) or (AB schoolchild*) | 279 |
| S6 | (TI youngster#) OR (AB youngster#) | 91 |
| S7 | (TI minor#) OR (AB minor#) | 234 |
| S8 | (TI pupil*) OR (AB pupil*) | 6,440 |
| S9 | ((TI student*) OR (AB student*)) NOT ((TI "medical student*") or (AB "medical student*")) | 47,126 |
| S10 | (TI prepubescen*) OR (AB prepubescen*) | 0 |
| S11 | (TI pubescent*) or (AB pubescent*) | 7 |
| S12 | (TI adolescent*) OR (AB adolescent*) | 3,224 |
| S13 | (TI juvenil*) OR (AB juvenil*) | 131 |
| S14 | (TI underage*) or (AB underage*) | 12 |
| S15 | (TI (preteen* or pre-teen*)) OR (AB (preteen* or pre-teen*)) | 18 |
| S16 | (TI (teen or teens)) OR (AB (teen or teens)) | 146 |
| S17 | (TI teenage*) OR (AB teenage*) | 599 |
| S18 | (TI (youth or youths)) OR (AB (youth or youths)) | 2,971 |
| S19 | (TI "young person*") OR (AB "young person*") | 143 |
| S20 | (TI "young people*") OR (AB "young people*") | 3,949 |
| S21 | (TI (kid or kids)) OR (AB (kid or kids)) | 274 |
| S22 | (TI (transition n4 adult*)) OR (AB (transition N4 adult*)) | 133 |
| S23 | (TI "emerging adult*") OR (AB "emerging adult*") | 27 |
| S24 | (TI "young adult*") OR (AB "young adult*") | 527 |
| S25 | (TI parent#) OR (AB parent#) | 6,126 |
| S26 | (TI mother#) OR (AB mother#) | 1,830 |
| S27 | (TI father#) or (AB father#) | 497 |
| S28 | (TI guardian#) OR (AB guardian#) | 82 |
| S29 | (TI teacher#) OR (AB teacher#) | 38,277 |
| S30 | (SO child*) | 10,878 |
| S31 | (SO adolescen*) | 168 |
| S32 | (SO youth*) | 218 |
| S33 | (SO school*) | 14,595 |
| S34 | (TI ("5" or "6" or "7" or "8" or "9" or "10" or "11" or "12" or "13" or "14" or "15" or "16" or "17" or "18") N1 ("year* old" or "year* of age")) OR (AB ("5" or "6" or "7" or "8" or "9" or "10" or "11" or "12" or "13" or "14" or "15" or "16" or "17" or "18") N1 ("year* old" or "year* of age")) | 375 |
| S35 | (TI (five or six or seven or eight or nine or ten or eleven or twelve or thirteen or fourteen or fifteen or sixteen or seventeen or eighteen) N1 ("year* old" or "year* of age")) OR (AB (five or six or seven or eight or nine or ten or eleven or twelve or thirteen or fourteen or fifteen or sixteen or seventeen or eighteen) N1 ("year* old" or "year* of age")) | 946 |
| S36 | (TI (age* N1 ("5" or "6" or "7" or "8" or "9" or "10" or "11" or "12" or "13" or "14" or "15" or "16" or "17" or "18") N1 year*)) OR (AB (age* N1 ("5" or "6" or "7" or "8" or "9" or "10" or "11" or "12" or "13" or "14" or "15" or "16" or "17" or "18") N1 year*)) | 233 |
| S37 | (TI (age* N1 (five or six or seven or eight or nine or ten or eleven or twelve or thirteen or fourteen or fifteen or sixteen or seventeen or eighteen) N1 year*)) OR (AB (age* N1 (five or six or seven or eight or nine or ten or eleven or twelve or thirteen or fourteen or fifteen or sixteen or seventeen or eighteen) N1 year*)) | 308 |
| S38 | (TI (primary or secondary or year) N1 ("1" or "2" or "3" or "4" or "5" or "6" or "7" or "8" or "9" or "10" or "11" or "12" or "13")) OR (AB (primary or secondary or year) N1 ("1" or "2" or "3" or "4" or "5" or "6" or "7" or "8" or "9" or "10" or "11" or "12" or "13")) | 2,754 |
| S39 | (TI grade# N1 (first or second or third or fourth or fifth or sixth or seventh or eighth or ninth or tenth or eleventh or twelfth)) OR (AB grade# N1 (first or second or third or fourth or fifth or sixth or seventh or eighth or ninth or tenth or eleventh or twelfth)) | 1,555 |
| S40 | S1 OR S2 OR S3 OR S4 OR S5 OR S6 OR S7 OR S8 OR S9 OR S10 OR S11 OR S12 OR S13 OR S14 OR S15 OR S16 OR S17 OR S18 OR S19 OR S20 OR S21 OR S22 OR S23 OR S24 OR S25 OR S26 OR S27 OR S28 OR S29 OR S30 OR S31 OR S32 OR S33 OR S34 OR S35 OR S36 OR S37 OR S38 OR S39 | 125,989 |
| S41 | (TI (school# N5 (based or level or wide or led or ethos or environment or organi#ation* or climate or toolkit# or approach* or "action group#" or practice# or whole or health* or leader* or cultur* or manag* or governance or system# or communit*))) OR (AB (school# N5 (based or level or wide or led or ethos or environment or organi#ation* or climate or toolkit# or approach* or "action group#" or practice# or whole or health* or leader* or cultur* or manag* or governance or system# or communit*))) | 13,447 |
| S42 | (TI schoolwide) or (AB schoolwide) | 35 |
| S43 | (TI "restor* justice") OR (AB "restor* justice") | 18 |
| S44 | (TI school#) or (AB school#) | 51,075 |
| S45 | (TI "peer-to-peer") OR (AB "peer-to-peer") | 109 |
| S46 | (TI (peer N2 (led or leader* or participat* or mediat* or helper# or resistan* or wide or action# or influen*))) OR (AB (peer N2 (led or leader* or participat* or mediat* or helper# or resistan* or wide or action# or influen*))) | 335 |
| S47 | (TI (relationship* N2 (led or leader* or participat* or mediat* or helper# or resistan* or wide or action# or influen*))) OR (AB (relationship* N2 (led or leader* or participat* or mediat* or helper# or resistan* or wide or action# or influen*))) | 468 |
| S48 | (TI ((community or communities) N2 (led or leader* or participat* or mediat* or helper# or resistan* or wide or action# or influen* or relationship*))) OR (AB ((community or communities) N2 (led or leader* or participat* or mediat* or helper# or resistan* or wide or action# or influen* or relationship*))) | 686 |
| S49 | ((TI (pupil# N2 (led or leader* or participat* or mediat* or helper# or resistan* or wide or action# or influen* or relationship*)) OR (AB (pupil# N2 (led or leader* or participat* or mediat* or helper# or resistan* or wide or action# or influen* or relationship*))) | 277 |
| S50 | (TI ((child or children) N2 (led or leader* or participat* or mediat* or helper# or resistan* or wide or action# or influen*))) OR (AB (child or children*) N2 (led or leader* or participat* or mediat* or helper# or resistan* or wide or action# or influen*))) | 1,123 |
| S51 | (TI (("young people" or "young person") N2 (led or leader* or participat* or mediat* or helper# or resistan* or wide or action# or influen*))) OR (AB (("young people" or "young person") N2 (led or leader* or participat* or mediat* or helper# or resistan* or wide or action# or influen*))) | 114 |
| S52 | (TI ((teen or teens or teenage*) N2 (led or leader* or participat* or mediat* or helper# or resistan* or wide or action# or influen*))) OR (AB ((teen or teens or teenage*) N2 (led or leader* or participat* or mediat* or helper# or resistan* or wide or action# or influen*))) | 24 |
| S53 | (TI (adolescen* N2 (led or leader* or participat* or mediat* or helper# or resistan* or wide or action# or influen*))) OR (AB (adolescen* N2 (led or leader* or participat* or mediat* or helper# or resistan* or wide or action# or influen*))) | 113 |
| S54 | (TI (youth# N2 (led or leader* or participat* or mediat* or helper# or resistan* or wide or action# or influen*))) OR (AB (youth# N2 (led or leader* or participat* or mediat* or helper# or resistan* or wide or action# or influen*))) | 209 |
| S55 | (TI (student# N2 (led or leader* or participat* or mediat* or helper# or resistan* or wide or action# or influen* or relationship*))) OR (AB (student# N2 (led or leader* or participat* or mediat* or helper# or resistan* or wide or action# or influen* or relationship*))) | 3,989 |
| S56 | (TI (teach* N2 (led or leader* or participat* or mediat* or helper# or resistan* or wide or action# or influen* or relationship*))) OR (AB (teach* N2 (led or leader* or participat* or mediat* or helper# or resistan* or wide or action# or influen* or relationship*))) | 3,936 |
| S57 | (TI (curricul* N2 (led or leader* or participat* or mediat* or helper# or resistan* or wide or action# or influen* or relationship*))) OR (AB (curricul* N2 (led or leader* or participat* or mediat* or helper# or resistan* or wide or action# or influen* or relationship*))) | 425 |
| S58 | (TI (administrat* N2 (led or leader* or participat* or mediat* or helper# or resistan* or wide or action# or influen* or relationship*))) OR (AB (administrat* N2 (led or leader* or participat* or mediat* or helper# or resistan* or wide or action# or influen* or relationship*))) | 137 |
| S59 | (TI ((personnel or staff) N2 (led or leader* or participat* or mediat* or helper# or resistan* or wide or action# or influen* or relationship*))) OR (AB ((personnel or staff) N2 (led or leader* or participat* or mediat* or helper# or resistan* or wide or action# or influen* or relationship*))) | 231 |
| S60 | (TI (volunteer* N2 (led or leader* or participat* or mediat* or helper# or resistan* or wide or action# or influen* or relationship*))) OR (AB (volulnteer* N2 (led or leader* or participat* or mediat* or helper# or resistan* or wide or action# or influen* or relationship*))) | 8 |
| S61 | (TI ((advocacy or advocate) N2 (led or leader* or participat* or mediat* or helper# or resistan* or wide or action# or influen* or relationship*))) OR (AB ((advocacy or advocate) N2 (led or leader* or participat* or mediat* or helper# or resistan* or wide or action# or influen* or relationship*))) | 61 |
| S62 | (TI (parent* N2 (led or leader* or participat* or mediat* or helper# or resistan* or wide or action# or influen*))) OR (AB (parent* N2 (led or leader* or participat* or mediat* or helper# or resistan* or wide or action# or influen*))) | 523 |
| S63 | (TI (environment* N2 (led or leader* or participat* or mediat* or helper# or resistan* or wide or action# or influen* or relationship*))) OR (AB (environment* N2 (led or leader* or participat* or mediat* or helper# or resistan* or wide or action# or influen* or relationship*))) | 531 |
| S64 | (TI ("socio-ecolog*" N2 (led or leader* or participat* or mediat* or helper# or resistan* or wide or action# or influen* or relationship*))) OR (AB ("socio-ecolog*" N2 (led or leader* or participat* or mediat* or helper# or resistan* or wide or action# or influen* or relationship*))) | 3 |
| S65 | (TI (learn* N2 (led or leader* or participat* or mediat* or helper# or resistan* or wide or action# or influen* or relationship*))) OR (AB (learn* N2 (led or leader* or participat* or mediat* or helper# or resistan* or wide or action# or influen* or relationship*))) | 2,699 |
| S66 | (TI ((policy or policies) N2 (led or leader* or participat* or mediat* or helper# or resistan* or wide or action# or influen* or relationship*))) OR (AB ((policy or policies) N2 (led or leader* or participat* or mediat* or helper# or resistan* or wide or action# or influen* or relationship*))) | 666 |
| S67 | S45 OR S46 OR S47 OR S48 OR S49 OR S50 OR S51 OR S52 OR S53 OR S54 OR S55 OR S56 OR S57 OR S58 OR S59 OR S60 OR S61 OR S62 OR S63 OR S64 OR S65 OR S66 | 13,747 |
| S68 | S44 AND S67 | 4,843 |
| S69 | S41 OR S42 OR S43 OR S68 | 16,175 |
| S70 | (TI ((smoking or smoked or smoke or smoker) N5 (reduc* or decreas* or increas* or lower* or fewer or improv* or enhance* or extend* or develop* or prevalen* or daily or weekly or current or rate or rates or behavio#r* or knowledge or prevent* or attitude* or avoid* or perceive* or percept* or promot* or program* or change*))) OR (AB ((smoking or smoked or smoke or smoker) N5 (reduc* or decreas* or increas* or lower* or fewer or improv* or enhance* or extend* or develop* or prevalen* or daily or weekly or current or rate or rates or behavio#r* or knowledge or prevent* or attitude* or avoid* or perceive* or percept* or promot* or program* or change*))) | 209 |
| S71 | (TI (tobacco N5 (reduc* or decreas* or increas* or lower* or fewer or improv* or enhance* or extend* or develop* or prevalen* or daily or weekly or current or rate or rates or behavio#r* or knowledge or prevent* or attitude* or avoid* or perceive* or percept* or promot* or program* or change*)) OR (AB (tobacco N5 (reduc* or decreas* or increas* or lower* or fewer or improv* or enhance* or extend* or develop* or prevalen* or daily or weekly or current or rate or rates or behavio#r* or knowledge or prevent* or attitude* or avoid* or perceive* or percept* or promot* or program* or change*)) | 83 |
| S72 | (TI (cigarette# N5 (reduc* or decreas* or increas* or lower* or fewer or improv* or enhance* or extend* or develop* or prevalen* or daily or weekly or current or rate or rates or behavio#r* or knowledge or prevent* or attitude* or avoid* or perceive* or percept* or promot* or program* or change*)) OR (AB (cigarette# N5 (reduc* or decreas* or increas* or lower* or fewer or improv* or enhance* or extend* or develop* or prevalen* or daily or weekly or current or rate or rates or behavio#r* or knowledge or prevent* or attitude* or avoid* or perceive* or percept* or promot* or program* or change*)) | 40 |
| S73 | (TI (marijuana N5 (reduc* or decreas* or increas* or lower* or fewer or improv* or enhance* or extend* or develop* or prevalen* or daily or weekly or current or rate or rates or behavio#r* or knowledge or prevent* or attitude* or avoid* or perceive* or percept* or promot* or program* or change*)) OR (AB (marijuana N5 (reduc* or decreas* or increas* or lower* or fewer or improv* or enhance* or extend* or develop* or prevalen* or daily or weekly or current or rate or rates or behavio#r* or knowledge or prevent* or attitude* or avoid* or perceive* or percept* or promot* or program* or change*)) | 16 |
| S74 | (TI (cannabis N5 (reduc* or decreas* or increas* or lower* or fewer or improv* or enhance* or extend* or develop* or prevalen* or daily or weekly or current or rate or rates or behavio#r* or knowledge or prevent* or attitude* or avoid* or perceive* or percept* or promot* or program* or change*)) OR (AB (cannabis N5 (reduc* or decreas* or increas* or lower* or fewer or improv* or enhance* or extend* or develop* or prevalen* or daily or weekly or current or rate or rates or behavio#r* or knowledge or prevent* or attitude* or avoid* or perceive* or percept* or promot* or program* or change*)) | 23 |
| S75 | (TI (solvent# N5 (reduc* or decreas* or increas* or lower* or fewer or improv* or enhance* or extend* or develop* or prevalen* or daily or weekly or current or rate or rates or behavio#r* or knowledge or prevent* or attitude* or avoid* or perceive* or percept* or promot* or program* or change*)) OR (AB (solvent# N5 (reduc* or decreas* or increas* or lower* or fewer or improv* or enhance* or extend* or develop* or prevalen* or daily or weekly or current or rate or rates or behavio#r* or knowledge or prevent* or attitude* or avoid* or perceive* or percept* or promot* or program* or change*)) | 4 |
| S76 | (TI (alcohol N5 (reduc* or decreas* or increas* or lower* or fewer or improv* or enhance* or extend* or develop* or prevalen* or daily or weekly or current or rate or rates or behavio#r* or knowledge or prevent* or attitude* or avoid* or perceive* or percept* or promot* or program* or change*)) OR (AB (alcohol N5 (reduc* or decreas* or increas* or lower* or fewer or improv* or enhance* or extend* or develop* or prevalen* or daily or weekly or current or rate or rates or behavio#r* or knowledge or prevent* or attitude* or avoid* or perceive* or percept* or promot* or program* or change*)) | 196 |
| S77 | (TI ("binge drink*" N5 (reduc* or decreas* or increas* or lower* or fewer or improv* or enhance* or extend* or develop* or prevalen* or daily or weekly or current or rate or rates or behavio#r* or knowledge or prevent* or attitude* or avoid* or perceive* or percept* or promot* or program* or change*)) OR (AB ("binge drink*" N5 (reduc* or decreas* or increas* or lower* or fewer or improv* or enhance* or extend* or develop* or prevalen* or daily or weekly or current or rate or rates or behavio#r* or knowledge or prevent* or attitude* or avoid* or perceive* or percept* or promot* or program* or change*)) | 14 |
| S78 | (TI ("underage drink*" N5 (reduc* or decreas* or increas* or lower* or fewer or improv* or enhance* or extend* or develop* or prevalen* or daily or weekly or current or rate or rates or behavio#r* or knowledge or prevent* or attitude* or avoid* or perceive* or percept* or promot* or program* or change*)) OR (AB ("underage drink*" N5 (reduc* or decreas* or increas* or lower* or fewer or improv* or enhance* or extend* or develop* or prevalen* or daily or weekly or current or rate or rates or behavio#r* or knowledge or prevent* or attitude* or avoid* or perceive* or percept* or promot* or program* or change*)) | 1,139 |
| S79 | (TI ("underage drink*" N5 (reduc* or decreas* or increas* or lower* or fewer or improv* or enhance* or extend* or develop* or prevalen* or daily or weekly or current or rate or rates or behavio#r* or knowledge or prevent* or attitude* or avoid* or perceive* or percept* or promot* or program* or change*)) OR (AB ("underage drink*" N5 (reduc* or decreas* or increas* or lower* or fewer or improv* or enhance* or extend* or develop* or prevalen* or daily or weekly or current or rate or rates or behavio#r* or knowledge or prevent* or attitude* or avoid* or perceive* or percept* or promot* or program* or change*)) | 1,139 |
| S80 | (TI (substance# N5 (reduc* or decreas* or increas* or lower* or fewer or improv* or enhance* or extend* or develop* or prevalen* or daily or weekly or current or rate or rates or behavio#r* or knowledge or prevent* or attitude* or avoid* or perceive* or percept* or promot* or program* or change*)) OR (AB (substance# N5 (reduc* or decreas* or increas* or lower* or fewer or improv* or enhance* or extend* or develop* or prevalen* or daily or weekly or current or rate or rates or behavio#r* or knowledge or prevent* or attitude* or avoid* or perceive* or percept* or promot* or program* or change*)) | 120 |
| S81 | (TI (drug# N5 (reduc* or decreas* or increas* or lower* or fewer or improv* or enhance* or extend* or develop* or prevalen* or daily or weekly or current or rate or rates or behavio#r* or knowledge or prevent* or attitude* or avoid* or perceive* or percept* or promot* or program* or change*)) OR (AB (drug# N5 (reduc* or decreas* or increas* or lower* or fewer or improv* or enhance* or extend* or develop* or prevalen* or daily or weekly or current or rate or rates or behavio#r* or knowledge or prevent* or attitude* or avoid* or perceive* or percept* or promot* or program* or change*)) | 263 |
| S82 | (TI ("education* attain*" N5 (reduc* or decreas* or increas* or lower* or fewer or improv* or enhance* or extend* or develop* or prevalen* or daily or weekly or current or rate or rates or behavio#r* or knowledge or prevent* or attitude* or avoid* or perceive* or percept* or promot* or program* or change*)) OR (AB ("education* attain*" N5 (reduc* or decreas* or increas* or lower* or fewer or improv* or enhance* or extend* or develop* or prevalen* or daily or weekly or current or rate or rates or behavio#r* or knowledge or prevent* or attitude* or avoid* or perceive* or percept* or promot* or program* or change*)) | 89 |
| S83 | (TI ("academic attain*" N5 (reduc* or decreas* or increas* or lower* or fewer or improv* or enhance* or extend* or develop* or prevalen* or daily or weekly or current or rate or rates or behavio#r* or knowledge or prevent* or attitude* or avoid* or perceive* or percept* or promot* or program* or change*)) OR (AB ("academic attain*" N5 (reduc* or decreas* or increas* or lower* or fewer or improv* or enhance* or extend* or develop* or prevalen* or daily or weekly or current or rate or rates or behavio#r* or knowledge or prevent* or attitude* or avoid* or perceive* or percept* or promot* or program* or change*)) | 27 |
| S84 | (TI ("student* attain*" N5 (reduc* or decreas* or increas* or lower* or fewer or improv* or enhance* or extend* or develop* or prevalen* or daily or weekly or current or rate or rates or behavio#r* or knowledge or prevent* or attitude* or avoid* or perceive* or percept* or promot* or program* or change*)) OR (AB ("student* attain*" N5 (reduc* or decreas* or increas* or lower* or fewer or improv* or enhance* or extend* or develop* or prevalen* or daily or weekly or current or rate or rates or behavio#r* or knowledge or prevent* or attitude* or avoid* or perceive* or percept* or promot* or program* or change*)) | 20 |
| S85 | (TI ("education* achieve*" N5 (reduc* or decreas* or increas* or lower* or fewer or improv* or enhance* or extend* or develop* or prevalen* or daily or weekly or current or rate or rates or behavio#r* or knowledge or prevent* or attitude* or avoid* or perceive* or percept* or promot* or program* or change*)) OR (AB ("education* achieve*" N5 (reduc* or decreas* or increas* or lower* or fewer or improv* or enhance* or extend* or develop* or prevalen* or daily or weekly or current or rate or rates or behavio#r* or knowledge or prevent* or attitude* or avoid* or perceive* or percept* or promot* or program* or change*)) | 46 |
| S86 | (TI ("academic achieve*" N5 (reduc* or decreas* or increas* or lower* or fewer or improv* or enhance* or extend* or develop* or prevalen* or daily or weekly or current or rate or rates or behavio#r* or knowledge or prevent* or attitude* or avoid* or perceive* or percept* or promot* or program* or change*)) OR (AB ("academic achieve*" N5 (reduc* or decreas* or increas* or lower* or fewer or improv* or enhance* or extend* or develop* or prevalen* or daily or weekly or current or rate or rates or behavio#r* or knowledge or prevent* or attitude* or avoid* or perceive* or percept* or promot* or program* or change*)) | 259 |
| S87 | (TI ("student* achieve*" N5 (reduc* or decreas* or increas* or lower* or fewer or improv* or enhance* or extend* or develop* or prevalen* or daily or weekly or current or rate or rates or behavio#r* or knowledge or prevent* or attitude* or avoid* or perceive* or percept* or promot* or program* or change*)) OR (AB ("student* achieve*" N5 (reduc* or decreas* or increas* or lower* or fewer or improv* or enhance* or extend* or develop* or prevalen* or daily or weekly or current or rate or rates or behavio#r* or knowledge or prevent* or attitude* or avoid* or perceive* or percept* or promot* or program* or change*)) | 238 |
| S88 | (TI ("education* perform*" N5 (reduc* or decreas* or increas* or lower* or fewer or improv* or enhance* or extend* or develop* or prevalen* or daily or weekly or current or rate or rates or behavio#r* or knowledge or prevent* or attitude* or avoid* or perceive* or percept* or promot* or program* or change*)) OR (AB ("education* perform*" N5 (reduc* or decreas* or increas* or lower* or fewer or improv* or enhance* or extend* or develop* or prevalen* or daily or weekly or current or rate or rates or behavio#r* or knowledge or prevent* or attitude* or avoid* or perceive* or percept* or promot* or program* or change*)) | 27 |
| S89 | (TI ("academic perform*" N5 (reduc* or decreas* or increas* or lower* or fewer or improv* or enhance* or extend* or develop* or prevalen* or daily or weekly or current or rate or rates or behavio#r* or knowledge or prevent* or attitude* or avoid* or perceive* or percept* or promot* or program* or change*)) OR (AB ("academic perform*" N5 (reduc* or decreas* or increas* or lower* or fewer or improv* or enhance* or extend* or develop* or prevalen* or daily or weekly or current or rate or rates or behavio#r* or knowledge or prevent* or attitude* or avoid* or perceive* or percept* or promot* or program* or change*)) | 255 |
| S90 | (TI ("student* perform*" N5 (reduc* or decreas* or increas* or lower* or fewer or improv* or enhance* or extend* or develop* or prevalen* or daily or weekly or current or rate or rates or behavio#r* or knowledge or prevent* or attitude* or avoid* or perceive* or percept* or promot* or program* or change*)) OR (AB ("student* perform*" N5 (reduc* or decreas* or increas* or lower* or fewer or improv* or enhance* or extend* or develop* or prevalen* or daily or weekly or current or rate or rates or behavio#r* or knowledge or prevent* or attitude* or avoid* or perceive* or percept* or promot* or program* or change*)) | 240 |
| S91 | (TI ("education* outcome#" N5 (reduc* or decreas* or increas* or lower* or fewer or improv* or enhance* or extend* or develop* or prevalen* or daily or weekly or current or rate or rates or behavio#r* or knowledge or prevent* or attitude* or avoid* or perceive* or percept* or promot* or program* or change*)) OR (AB ("education* outcome#" N5 (reduc* or decreas* or increas* or lower* or fewer or improv* or enhance* or extend* or develop* or prevalen* or daily or weekly or current or rate or rates or behavio#r* or knowledge or prevent* or attitude* or avoid* or perceive* or percept* or promot* or program* or change*)) | 111 |
| S92 | (TI ("academic outcome#" N5 (reduc* or decreas* or increas* or lower* or fewer or improv* or enhance* or extend* or develop* or prevalen* or daily or weekly or current or rate or rates or behavio#r* or knowledge or prevent* or attitude* or avoid* or perceive* or percept* or promot* or program* or change*)) OR (AB ("academic outcome#" N5 (reduc* or decreas* or increas* or lower* or fewer or improv* or enhance* or extend* or develop* or prevalen* or daily or weekly or current or rate or rates or behavio#r* or knowledge or prevent* or attitude* or avoid* or perceive* or percept* or promot* or program* or change*)) | 70 |
| S93 | (TI ("student* outcome#" N5 (reduc* or decreas* or increas* or lower* or fewer or improv* or enhance* or extend* or develop* or prevalen* or daily or weekly or current or rate or rates or behavio#r* or knowledge or prevent* or attitude* or avoid* or perceive* or percept* or promot* or program* or change*)) OR (AB ("student* outcome#" N5 (reduc* or decreas* or increas* or lower* or fewer or improv* or enhance* or extend* or develop* or prevalen* or daily or weekly or current or rate or rates or behavio#r* or knowledge or prevent* or attitude* or avoid* or perceive* or percept* or promot* or program* or change*)) | 126 |
| S94 | (TI ("education* commitment" N5 (reduc* or decreas* or increas* or lower* or fewer or improv* or enhance* or extend* or develop* or prevalen* or daily or weekly or current or rate or rates or behavio#r* or knowledge or prevent* or attitude* or avoid* or perceive* or percept* or promot* or program* or change*)) OR (AB ("education* commitment" N5 (reduc* or decreas* or increas* or lower* or fewer or improv* or enhance* or extend* or develop* or prevalen* or daily or weekly or current or rate or rates or behavio#r* or knowledge or prevent* or attitude* or avoid* or perceive* or percept* or promot* or program* or change*)) | 1,280 |
| S95 | (TI ("education* commitment" N5 (reduc* or decreas* or increas* or lower* or fewer or improv* or enhance* or extend* or develop* or prevalen* or daily or weekly or current or rate or rates or behavio#r* or knowledge or prevent* or attitude* or avoid* or perceive* or percept* or promot* or program* or change*)) OR (AB ("education* commitment" N5 (reduc* or decreas* or increas* or lower* or fewer or improv* or enhance* or extend* or develop* or prevalen* or daily or weekly or current or rate or rates or behavio#r* or knowledge or prevent* or attitude* or avoid* or perceive* or percept* or promot* or program* or change*)) | 1,280 |
| S96 | (TI ("academic commitment" N5 (reduc* or decreas* or increas* or lower* or fewer or improv* or enhance* or extend* or develop* or prevalen* or daily or weekly or current or rate or rates or behavio#r* or knowledge or prevent* or attitude* or avoid* or perceive* or percept* or promot* or program* or change*)) OR (AB ("academic commitment" N5 (reduc* or decreas* or increas* or lower* or fewer or improv* or enhance* or extend* or develop* or prevalen* or daily or weekly or current or rate or rates or behavio#r* or knowledge or prevent* or attitude* or avoid* or perceive* or percept* or promot* or program* or change*)) | 1,280 |
| S97 | (TI ("academic commitment" N5 (reduc* or decreas* or increas* or lower* or fewer or improv* or enhance* or extend* or develop* or prevalen* or daily or weekly or current or rate or rates or behavio#r* or knowledge or prevent* or attitude* or avoid* or perceive* or percept* or promot* or program* or change*)) OR (AB ("academic commitment" N5 (reduc* or decreas* or increas* or lower* or fewer or improv* or enhance* or extend* or develop* or prevalen* or daily or weekly or current or rate or rates or behavio#r* or knowledge or prevent* or attitude* or avoid* or perceive* or percept* or promot* or program* or change*)) | 1,280 |
| S98 | (TI ("student* commitment" N5 (reduc* or decreas* or increas* or lower* or fewer or improv* or enhance* or extend* or develop* or prevalen* or daily or weekly or current or rate or rates or behavio#r* or knowledge or prevent* or attitude* or avoid* or perceive* or percept* or promot* or program* or change*)) OR (AB ("student* commitment" N5 (reduc* or decreas* or increas* or lower* or fewer or improv* or enhance* or extend* or develop* or prevalen* or daily or weekly or current or rate or rates or behavio#r* or knowledge or prevent* or attitude* or avoid* or perceive* or percept* or promot* or program* or change*)) | 5 |
| S99 | (TI ("education* engage*" N5 (reduc* or decreas* or increas* or lower* or fewer or improv* or enhance* or extend* or develop* or prevalen* or daily or weekly or current or rate or rates or behavio#r* or knowledge or prevent* or attitude* or avoid* or perceive* or percept* or promot* or program* or change*)) OR (AB ("education* engage*" N5 (reduc* or decreas* or increas* or lower* or fewer or improv* or enhance* or extend* or develop* or prevalen* or daily or weekly or current or rate or rates or behavio#r* or knowledge or prevent* or attitude* or avoid* or perceive* or percept* or promot* or program* or change*)) | 5 |
| S100 | (TI ("academic engage*" N5 (reduc* or decreas* or increas* or lower* or fewer or improv* or enhance* or extend* or develop* or prevalen* or daily or weekly or current or rate or rates or behavio#r* or knowledge or prevent* or attitude* or avoid* or perceive* or percept* or promot* or program* or change*)) OR (AB ("academic engage*" N5 (reduc* or decreas* or increas* or lower* or fewer or improv* or enhance* or extend* or develop* or prevalen* or daily or weekly or current or rate or rates or behavio#r* or knowledge or prevent* or attitude* or avoid* or perceive* or percept* or promot* or program* or change*)) | 29 |
| S101 | (TI ("student* engage*" N5 (reduc* or decreas* or increas* or lower* or fewer or improv* or enhance* or extend* or develop* or prevalen* or daily or weekly or current or rate or rates or behavio#r* or knowledge or prevent* or attitude* or avoid* or perceive* or percept* or promot* or program* or change*)) OR (AB ("student* engage*" N5 (reduc* or decreas* or increas* or lower* or fewer or improv* or enhance* or extend* or develop* or prevalen* or daily or weekly or current or rate or rates or behavio#r* or knowledge or prevent* or attitude* or avoid* or perceive* or percept* or promot* or program* or change*)) | 396 |
| S102 | (TI (dropout N5 (reduc* or decreas* or increas* or lower* or fewer or improv* or enhance* or extend* or develop* or prevalen* or daily or weekly or current or rate or rates or behavio#r* or knowledge or prevent* or attitude* or avoid* or perceive* or percept* or promot* or program* or change*)) OR (AB (dropout N5 (reduc* or decreas* or increas* or lower* or fewer or improv* or enhance* or extend* or develop* or prevalen* or daily or weekly or current or rate or rates or behavio#r* or knowledge or prevent* or attitude* or avoid* or perceive* or percept* or promot* or program* or change*)) | 161 |
| S103 | (TI (absentee* N5 (reduc* or decreas* or increas* or lower* or fewer or improv* or enhance* or extend* or develop* or prevalen* or daily or weekly or current or rate or rates or behavio#r* or knowledge or prevent* or attitude* or avoid* or perceive* or percept* or promot* or program* or change*)) OR (AB (absentee* N5 (reduc* or decreas* or increas* or lower* or fewer or improv* or enhance* or extend* or develop* or prevalen* or daily or weekly or current or rate or rates or behavio#r* or knowledge or prevent* or attitude* or avoid* or perceive* or percept* or promot* or program* or change*)) | 19 |
| S104 | (TI ((violent or violence) N5 (reduc* or decreas* or increas* or lower* or fewer or improv* or enhance* or extend* or develop* or prevalen* or daily or weekly or current or rate or rates or behavio#r* or knowledge or prevent* or attitude* or avoid* or perceive* or percept* or promot* or program* or change*))) OR (AB ((violent or violence) N5 (reduc* or decreas* or increas* or lower* or fewer or improv* or enhance* or extend* or develop* or prevalen* or daily or weekly or current or rate or rates or behavio#r* or knowledge or prevent* or attitude* or avoid* or perceive* or percept* or promot* or program* or change*))) | 211 |
| S105 | (TI ((aggression or aggressive) N5 (reduc* or decreas* or increas* or lower* or fewer or improv* or enhance* or extend* or develop* or prevalen* or daily or weekly or current or rate or rates or behavio#r* or knowledge or prevent* or attitude* or avoid* or perceive* or percept* or promot* or program* or change*))) OR (AB ((aggression or aggressive) N5 (reduc* or decreas* or increas* or lower* or fewer or improv* or enhance* or extend* or develop* or prevalen* or daily or weekly or current or rate or rates or behavio#r* or knowledge or prevent* or attitude* or avoid* or perceive* or percept* or promot* or program* or change*))) | 237 |
| S106 | (TI ((hostile or hostility) N5 (reduc* or decreas* or increas* or lower* or fewer or improv* or enhance* or extend* or develop* or prevalen* or daily or weekly or current or rate or rates or behavio#r* or knowledge or prevent* or attitude* or avoid* or perceive* or percept* or promot* or program* or change*))) OR (AB ((hostile or hostility) N5 (reduc* or decreas* or increas* or lower* or fewer or improv* or enhance* or extend* or develop* or prevalen* or daily or weekly or current or rate or rates or behavio#r* or knowledge or prevent* or attitude* or avoid* or perceive* or percept* or promot* or program* or change*))) | 27 |
| S107 | (TI (assault* N5 (reduc* or decreas* or increas* or lower* or fewer or improv* or enhance* or extend* or develop* or prevalen* or daily or weekly or current or rate or rates or behavio#r* or knowledge or prevent* or attitude* or avoid* or perceive* or percept* or promot* or program* or change*)) OR (AB (assault* N5 (reduc* or decreas* or increas* or lower* or fewer or improv* or enhance* or extend* or develop* or prevalen* or daily or weekly or current or rate or rates or behavio#r* or knowledge or prevent* or attitude* or avoid* or perceive* or percept* or promot* or program* or change*)) | 14 |
| S108 | (TI (crime N5 (reduc* or decreas* or increas* or lower* or fewer or improv* or enhance* or extend* or develop* or prevalen* or daily or weekly or current or rate or rates or behavio#r* or knowledge or prevent* or attitude* or avoid* or perceive* or percept* or promot* or program* or change*)) OR (AB (crime N5 (reduc* or decreas* or increas* or lower* or fewer or improv* or enhance* or extend* or develop* or prevalen* or daily or weekly or current or rate or rates or behavio#r* or knowledge or prevent* or attitude* or avoid* or perceive* or percept* or promot* or program* or change*)) | 44 |
| S109 | (TI ((abuse# or abusive) N5 (reduc* or decreas* or increas* or lower* or fewer or improv* or enhance* or extend* or develop* or prevalen* or daily or weekly or current or rate or rates or behavio#r* or knowledge or prevent* or attitude* or avoid* or perceive* or percept* or promot* or program* or change*))) OR (AB ((abuse# or abusive) N5 (reduc* or decreas* or increas* or lower* or fewer or improv* or enhance* or extend* or develop* or prevalen* or daily or weekly or current or rate or rates or behavio#r* or knowledge or prevent* or attitude* or avoid* or perceive* or percept* or promot* or program* or change*))) | 189 |
| S110 | (TI (misbehav* N5 (reduc* or decreas* or increas* or lower* or fewer or improv* or enhance* or extend* or develop* or prevalen* or daily or weekly or current or rate or rates or behavio#r* or knowledge or prevent* or attitude* or avoid* or perceive* or percept* or promot* or program* or change*)) OR (AB (misbehav* N5 (reduc* or decreas* or increas* or lower* or fewer or improv* or enhance* or extend* or develop* or prevalen* or daily or weekly or current or rate or rates or behavio#r* or knowledge or prevent* or attitude* or avoid* or perceive* or percept* or promot* or program* or change*)) | 24 |
| S111 | (TI (threat# N5 (reduc* or decreas* or increas* or lower* or fewer or improv* or enhance* or extend* or develop* or prevalen* or daily or weekly or current or rate or rates or behavio#r* or knowledge or prevent* or attitude* or avoid* or perceive* or percept* or promot* or program* or change*)) OR (AB (threat# N5 (reduc* or decreas* or increas* or lower* or fewer or improv* or enhance* or extend* or develop* or prevalen* or daily or weekly or current or rate or rates or behavio#r* or knowledge or prevent* or attitude* or avoid* or perceive* or percept* or promot* or program* or change*)) | 136 |
| S112 | (TI (conduct N5 (reduc* or decreas* or increas* or lower* or fewer or improv* or enhance* or extend* or develop* or prevalen* or daily or weekly or current or rate or rates or behavio#r* or knowledge or prevent* or attitude* or avoid* or perceive* or percept* or promot* or program* or change*)) OR (AB (conduct N5 (reduc* or decreas* or increas* or lower* or fewer or improv* or enhance* or extend* or develop* or prevalen* or daily or weekly or current or rate or rates or behavio#r* or knowledge or prevent* or attitude* or avoid* or perceive* or percept* or promot* or program* or change*)) | 143 |
| S113 | (TI (delinquen* N5 (reduc* or decreas* or increas* or lower* or fewer or improv* or enhance* or extend* or develop* or prevalen* or daily or weekly or current or rate or rates or behavio#r* or knowledge or prevent* or attitude* or avoid* or perceive* or percept* or promot* or program* or change*)) OR (AB (delinquen* N5 (reduc* or decreas* or increas* or lower* or fewer or improv* or enhance* or extend* or develop* or prevalen* or daily or weekly or current or rate or rates or behavio#r* or knowledge or prevent* or attitude* or avoid* or perceive* or percept* or promot* or program* or change*)) | 40 |
| S114 | (TI ("disrupt* behavio#r*" N5 (reduc* or decreas* or increas* or lower* or fewer or improv* or enhance* or extend* or develop* or prevalen* or daily or weekly or current or rate or rates or behavio#r* or knowledge or prevent* or attitude* or avoid* or perceive* or percept* or promot* or program* or change*)) OR (AB ("disrupt* behavio#r*" N5 (reduc* or decreas* or increas* or lower* or fewer or improv* or enhance* or extend* or develop* or prevalen* or daily or weekly or current or rate or rates or behavio#r* or knowledge or prevent* or attitude* or avoid* or perceive* or percept* or promot* or program* or change*)) | 102 |
| S115 | (TI ("problem* behavio#r*" N5 (reduc* or decreas* or increas* or lower* or fewer or improv* or enhance* or extend* or develop* or prevalen* or daily or weekly or current or rate or rates or behavio#r* or knowledge or prevent* or attitude* or avoid* or perceive* or percept* or promot* or program* or change*)) OR (AB ("problem* behavio#r*" N5 (reduc* or decreas* or increas* or lower* or fewer or improv* or enhance* or extend* or develop* or prevalen* or daily or weekly or current or rate or rates or behavio#r* or knowledge or prevent* or attitude* or avoid* or perceive* or percept* or promot* or program* or change*)) | 184 |
| S116 | (TI ("volatile behavio#r*" N5 (reduc* or decreas* or increas* or lower* or fewer or improv* or enhance* or extend* or develop* or prevalen* or daily or weekly or current or rate or rates or behavio#r* or knowledge or prevent* or attitude* or avoid* or perceive* or percept* or promot* or program* or change*)) OR (AB ("volatile behavio#r*" N5 (reduc* or decreas* or increas* or lower* or fewer or improv* or enhance* or extend* or develop* or prevalen* or daily or weekly or current or rate or rates or behavio#r* or knowledge or prevent* or attitude* or avoid* or perceive* or percept* or promot* or program* or change*)) | 1,273 |
| S117 | (TI ("volatile behavio#r*" N5 (reduc* or decreas* or increas* or lower* or fewer or improv* or enhance* or extend* or develop* or prevalen* or daily or weekly or current or rate or rates or behavio#r* or knowledge or prevent* or attitude* or avoid* or perceive* or percept* or promot* or program* or change*)) OR (AB ("volatile behavio#r*" N5 (reduc* or decreas* or increas* or lower* or fewer or improv* or enhance* or extend* or develop* or prevalen* or daily or weekly or current or rate or rates or behavio#r* or knowledge or prevent* or attitude* or avoid* or perceive* or percept* or promot* or program* or change*)) | 1,273 |
| S118 | (TI ((antisocial or "anti-social") N5 (reduc* or decreas* or increas* or lower* or fewer or improv* or enhance* or extend* or develop* or prevalen* or daily or weekly or current or rate or rates or behavio#r* or knowledge or prevent* or attitude* or avoid* or perceive* or percept* or promot* or program* or change*))) OR (AB ((antisocial or "anti-social") N5 (reduc* or decreas* or increas* or lower* or fewer or improv* or enhance* or extend* or develop* or prevalen* or daily or weekly or current or rate or rates or behavio#r* or knowledge or prevent* or attitude* or avoid* or perceive* or percept* or promot* or program* or change*))) | 88 |
| S119 | (TI (perpetrat* N5 (reduc* or decreas* or increas* or lower* or fewer or improv* or enhance* or extend* or develop* or prevalen* or daily or weekly or current or rate or rates or behavio#r* or knowledge or prevent* or attitude* or avoid* or perceive* or percept* or promot* or program* or change*)) OR (AB (perpetrat* N5 (reduc* or decreas* or increas* or lower* or fewer or improv* or enhance* or extend* or develop* or prevalen* or daily or weekly or current or rate or rates or behavio#r* or knowledge or prevent* or attitude* or avoid* or perceive* or percept* or promot* or program* or change*)) | 23 |
| S120 | (TI (bully* N5 (reduc* or decreas* or increas* or lower* or fewer or improv* or enhance* or extend* or develop* or prevalen* or daily or weekly or current or rate or rates or behavio#r* or knowledge or prevent* or attitude* or avoid* or perceive* or percept* or promot* or program* or change*)) OR (AB (bully* N5 (reduc* or decreas* or increas* or lower* or fewer or improv* or enhance* or extend* or develop* or prevalen* or daily or weekly or current or rate or rates or behavio#r* or knowledge or prevent* or attitude* or avoid* or perceive* or percept* or promot* or program* or change*)) | 304 |
| S121 | (TI (victim* N5 (reduc* or decreas* or increas* or lower* or fewer or improv* or enhance* or extend* or develop* or prevalen* or daily or weekly or current or rate or rates or behavio#r* or knowledge or prevent* or attitude* or avoid* or perceive* or percept* or promot* or program* or change*)) OR (AB (victim* N5 (reduc* or decreas* or increas* or lower* or fewer or improv* or enhance* or extend* or develop* or prevalen* or daily or weekly or current or rate or rates or behavio#r* or knowledge or prevent* or attitude* or avoid* or perceive* or percept* or promot* or program* or change*)) | 161 |
| S122 | (TI (cyberbully* N5 (reduc* or decreas* or increas* or lower* or fewer or improv* or enhance* or extend* or develop* or prevalen* or daily or weekly or current or rate or rates or behavio#r* or knowledge or prevent* or attitude* or avoid* or perceive* or percept* or promot* or program* or change*)) OR (AB (cyberbully* N5 (reduc* or decreas* or increas* or lower* or fewer or improv* or enhance* or extend* or develop* or prevalen* or daily or weekly or current or rate or rates or behavio#r* or knowledge or prevent* or attitude* or avoid* or perceive* or percept* or promot* or program* or change*)) | 42 |
| S123 | (TI (conflict* N5 (reduc* or decreas* or increas* or lower* or fewer or improv* or enhance* or extend* or develop* or prevalen* or daily or weekly or current or rate or rates or behavio#r* or knowledge or prevent* or attitude* or avoid* or perceive* or percept* or promot* or program* or change*)) OR (AB (conflict* N5 (reduc* or decreas* or increas* or lower* or fewer or improv* or enhance* or extend* or develop* or prevalen* or daily or weekly or current or rate or rates or behavio#r* or knowledge or prevent* or attitude* or avoid* or perceive* or percept* or promot* or program* or change*)) | 397 |
| S124 | (TI ("positive behavio#r*" N5 (reduc* or decreas* or increas* or lower* or fewer or improv* or enhance* or extend* or develop* or prevalen* or daily or weekly or current or rate or rates or behavio#r* or knowledge or prevent* or attitude* or avoid* or perceive* or percept* or promot* or program* or change*)) OR (AB ("positive behavio#r*" N5 (reduc* or decreas* or increas* or lower* or fewer or improv* or enhance* or extend* or develop* or prevalen* or daily or weekly or current or rate or rates or behavio#r* or knowledge or prevent* or attitude* or avoid* or perceive* or percept* or promot* or program* or change*)) | 97 |
| S125 | (TI ("improv* behavio#r*" N5 (reduc* or decreas* or increas* or lower* or fewer or improv* or enhance* or extend* or develop* or prevalen* or daily or weekly or current or rate or rates or behavio#r* or knowledge or prevent* or attitude* or avoid* or perceive* or percept* or promot* or program* or change*)) OR (AB ("improv* behavio#r*" N5 (reduc* or decreas* or increas* or lower* or fewer or improv* or enhance* or extend* or develop* or prevalen* or daily or weekly or current or rate or rates or behavio#r* or knowledge or prevent* or attitude* or avoid* or perceive* or percept* or promot* or program* or change*)) | 20 |
| S126 | (TI ("social behavio#r*" N5 (reduc* or decreas* or increas* or lower* or fewer or improv* or enhance* or extend* or develop* or prevalen* or daily or weekly or current or rate or rates or behavio#r* or knowledge or prevent* or attitude* or avoid* or perceive* or percept* or promot* or program* or change*)) OR (AB ("social behavio#r*" N5 (reduc* or decreas* or increas* or lower* or fewer or improv* or enhance* or extend* or develop* or prevalen* or daily or weekly or current or rate or rates or behavio#r* or knowledge or prevent* or attitude* or avoid* or perceive* or percept* or promot* or program* or change*)) | 191 |
| S127 | (TI (("social-emotion*" or "socio-emotion*") N5 (reduc* or decreas* or increas* or lower* or fewer or improv* or enhance* or extend* or develop* or prevalen* or daily or weekly or current or rate or rates or behavio#r* or knowledge or prevent* or attitude* or avoid* or perceive* or percept* or promot* or program* or change*))) OR (AB (("social-emotion*" or "socio-emotion*") N5 (reduc* or decreas* or increas* or lower* or fewer or improv* or enhance* or extend* or develop* or prevalen* or daily or weekly or current or rate or rates or behavio#r* or knowledge or prevent* or attitude* or avoid* or perceive* or percept* or promot* or program* or change*))) | 299 |
| S128 | (TI ("social-charact*" N5 (reduc* or decreas* or increas* or lower* or fewer or improv* or enhance* or extend* or develop* or prevalen* or daily or weekly or current or rate or rates or behavio#r* or knowledge or prevent* or attitude* or avoid* or perceive* or percept* or promot* or program* or change*)) OR (AB ("social-charact*" N5 (reduc* or decreas* or increas* or lower* or fewer or improv* or enhance* or extend* or develop* or prevalen* or daily or weekly or current or rate or rates or behavio#r* or knowledge or prevent* or attitude* or avoid* or perceive* or percept* or promot* or program* or change*)) | 6 |
| S129 | (TI ("social-inclusion" N5 (reduc* or decreas* or increas* or lower* or fewer or improv* or enhance* or extend* or develop* or prevalen* or daily or weekly or current or rate or rates or behavio#r* or knowledge or prevent* or attitude* or avoid* or perceive* or percept* or promot* or program* or change*)) OR (AB ("social-inclusion" N5 (reduc* or decreas* or increas* or lower* or fewer or improv* or enhance* or extend* or develop* or prevalen* or daily or weekly or current or rate or rates or behavio#r* or knowledge or prevent* or attitude* or avoid* or perceive* or percept* or promot* or program* or change*)) | 88 |
| S130 | (TI ("social develop*" N5 (reduc* or decreas* or increas* or lower* or fewer or improv* or enhance* or extend* or develop* or prevalen* or daily or weekly or current or rate or rates or behavio#r* or knowledge or prevent* or attitude* or avoid* or perceive* or percept* or promot* or program* or change*)) OR (AB ("social develop*" N5 (reduc* or decreas* or increas* or lower* or fewer or improv* or enhance* or extend* or develop* or prevalen* or daily or weekly or current or rate or rates or behavio#r* or knowledge or prevent* or attitude* or avoid* or perceive* or percept* or promot* or program* or change*)) | 215 |
| S131 | (TI ("social competen*" N5 (reduc* or decreas* or increas* or lower* or fewer or improv* or enhance* or extend* or develop* or prevalen* or daily or weekly or current or rate or rates or behavio#r* or knowledge or prevent* or attitude* or avoid* or perceive* or percept* or promot* or program* or change*)) OR (AB ("social competen*" N5 (reduc* or decreas* or increas* or lower* or fewer or improv* or enhance* or extend* or develop* or prevalen* or daily or weekly or current or rate or rates or behavio#r* or knowledge or prevent* or attitude* or avoid* or perceive* or percept* or promot* or program* or change*)) | 57 |
| S132 | (TI ("emotion* develop*" N5 (reduc* or decreas* or increas* or lower* or fewer or improv* or enhance* or extend* or develop* or prevalen* or daily or weekly or current or rate or rates or behavio#r* or knowledge or prevent* or attitude* or avoid* or perceive* or percept* or promot* or program* or change*)) OR (AB ("emotion* develop*" N5 (reduc* or decreas* or increas* or lower* or fewer or improv* or enhance* or extend* or develop* or prevalen* or daily or weekly or current or rate or rates or behavio#r* or knowledge or prevent* or attitude* or avoid* or perceive* or percept* or promot* or program* or change*)) | 141 |
| S133 | (TI (conduct N5 (reduc* or decreas* or increas* or lower* or fewer or improv* or enhance* or extend* or develop* or prevalen* or daily or weekly or current or rate or rates or behavio#r* or knowledge or prevent* or attitude* or avoid* or perceive* or percept* or promot* or program* or change*)) OR (AB (conduct N5 (reduc* or decreas* or increas* or lower* or fewer or improv* or enhance* or extend* or develop* or prevalen* or daily or weekly or current or rate or rates or behavio#r* or knowledge or prevent* or attitude* or avoid* or perceive* or percept* or promot* or program* or change*)) | 143 |
| S134 | (TI (respect N5 (reduc* or decreas* or increas* or lower* or fewer or improv* or enhance* or extend* or develop* or prevalen* or daily or weekly or current or rate or rates or behavio#r* or knowledge or prevent* or attitude* or avoid* or perceive* or percept* or promot* or program* or change*)) OR (AB (respect N5 (reduc* or decreas* or increas* or lower* or fewer or improv* or enhance* or extend* or develop* or prevalen* or daily or weekly or current or rate or rates or behavio#r* or knowledge or prevent* or attitude* or avoid* or perceive* or percept* or promot* or program* or change*)) | 381 |
| S135 | (TI ((safety or safe) N5 (reduc* or decreas* or increas* or lower* or fewer or improv* or enhance* or extend* or develop* or prevalen* or daily or weekly or current or rate or rates or behavio#r* or knowledge or prevent* or attitude* or avoid* or perceive* or percept* or promot* or program* or change*))) OR (AB ((safety or safe) N5 (reduc* or decreas* or increas* or lower* or fewer or improv* or enhance* or extend* or develop* or prevalen* or daily or weekly or current or rate or rates or behavio#r* or knowledge or prevent* or attitude* or avoid* or perceive* or percept* or promot* or program* or change*))) | 316 |
| S136 | S70 OR S71 OR S72 OR S73 OR S74 OR S75 OR S76 OR S77 OR S78 OR S79 OR S80 OR S81 OR S82 OR S83 OR S84 OR S85 OR S86 OR S87 OR S88 OR S89 OR S90 OR S91 OR S92 OR S93 OR S94 OR S95 OR S96 OR S97 OR S98 OR S99 OR S100 OR S101 OR S102 OR S103 OR S104 OR S105 OR S106 OR S107 OR S108 OR S109 OR S110 OR S111 OR S112 OR S113 OR S114 OR S115 OR S116 OR S117 OR S118 OR S119 OR S120 OR S121 OR S122 OR S123 OR S124 OR S125 OR S126 OR S127 OR S128 OR S129 OR S130 OR S131 OR S132 OR S133 OR S134 OR S135 | 6,240 |
| S137 | (TI prevent*) OR (AB prevent*) | 2,176 |
| S138 | (TI program*) OR (AB program*) | 17,947 |
| S139 | (TI intervent*) OR (AB intervent*) | 6,812 |
| S140 | (TI "restorative approach*") OR (AB "restorative approach*") | 9 |
| S141 | (TI "control school#") OR (AB "control school#") | 29 |
| S142 | (TI (trial or trials)) OR (AB (trial or trials)) | 1,174 |
| S143 | S137 OR S138 OR S139 OR S140 OR S141 OR S142 | 25,098 |
| S144 | S40 AND S69 AND S136 AND S143 | 440 |

## EBSCO CINAHL Plus

| Database name | CINAHL Plus |
| --- | --- |
| Database platform | EBSCO |
| Dates of database coverage | Complete database to date searched. |
| Date searched | 16/01/2020 |
| Searched by | JF |
| Number of results | 6011 |
| EndNote import order | 8 |
| Number of results once duplicates removed | 1728 |
| Search strategy notes | * is used for truncation. # is used for an optional wildcard. Search strings starting with TI search the title only. Search strings starting with AB search the abstract only. Search strings starting with SO search the journal title only. N/*n* search for terms within *n* words of each other, in any order. |

| Search number | Query | Results |
| --- | --- | --- |
| S1 | (MH "Child") | 415,620 |
| S2 | (MH "Adolescence") | 479,274 |
| S3 | (MH "Minors (Legal)") | 643 |
| S4 | (MH "Students, High School") OR (MH "Students, Middle School") OR (MH "Students") | 26,646 |
| S5 | (MH "Teachers") | 10,710 |
| S6 | (MH "Parents+") | 86,439 |
| S7 | (TI child*) or (AB child*) | 432,743 |
| S8 | (TI boy#) or (AB boy#) | 33,127 |
| S9 | (TI girl#) or (AB girl#) | 36,706 |
| S10 | (TI (schoolage or (school N1 age))) or (AB (schoolage or (school N1 age))) | 5,771 |
| S11 | (TI schoolchild*) or (AB schoolchild*) | 3,468 |
| S12 | (TI youngster#) OR (AB youngster#) | 777 |
| S13 | (TI minor#) OR (AB minor#) | 27,261 |
| S14 | ((TI pupil*) OR (AB pupil*)) NOT (MH "Pupil") | 4,099 |
| S15 | ((TI student*) OR (AB student*)) NOT (MH "Students, Health Occupations+") | 109,618 |
| S16 | (TI prepubescen*) OR (AB prepubescen*) | 246 |
| S17 | (TI pubescent*) or (AB pubescent*) | 157 |
| S18 | (TI adolescent*) OR (AB adolescent*) | 112,736 |
| S19 | (TI juvenil*) OR (AB juvenil*) | 9,046 |
| S20 | (TI underage*) or (AB underage*) | 759 |
| S21 | (TI (preteen* or pre-teen*)) OR (AB (preteen* or pre-teen*)) | 277 |
| S22 | (TI (teen or teens)) OR (AB (teen or teens)) | 8,137 |
| S23 | (TI teenage*) OR (AB teenage*) | 9,295 |
| S24 | (TI (youth or youths)) OR (AB (youth or youths)) | 43,559 |
| S25 | (TI "young person*") OR (AB "young person*") | 1,422 |
| S26 | (TI "young people*") OR (AB "young people*") | 17,295 |
| S27 | (TI (kid or kids)) OR (AB (kid or kids)) | 8,547 |
| S28 | (TI (transition n4 adult*)) OR (AB (transition N4 adult*)) | 2,762 |
| S29 | (TI "emerging adult*") OR (AB "emerging adult*") | 1,710 |
| S30 | (TI "young adult*") OR (AB "young adult*") | 30,891 |
| S31 | (TI parent#) OR (AB parent#) | 91,744 |
| S32 | (TI mother#) OR (AB mother#) | 67,743 |
| S33 | (TI father#) or (AB father#) | 13,383 |
| S34 | (TI guardian#) OR (AB guardian#) | 2,499 |
| S35 | (TI teacher#) OR (AB teacher#) | 24,015 |
| S36 | (SO child*) | 100,638 |
| S37 | (SO adolescen*) | 38,466 |
| S38 | (SO youth*) | 7,328 |
| S39 | (SO school*) | 16,812 |
| S40 | (TI ("5" or "6" or "7" or "8" or "9" or "10" or "11" or "12" or "13" or "14" or "15" or "16" or "17" or "18") N1 ("year* old" or "year* of age")) OR (AB ("5" or "6" or "7" or "8" or "9" or "10" or "11" or "12" or "13" or "14" or "15" or "16" or "17" or "18") N1 ("year* old" or "year* of age")) | 24,381 |
| S41 | (TI (five or six or seven or eight or nine or ten or eleven or twelve or thirteen or fourteen or fifteen or sixteen or seventeen or eighteen) N1 ("year* old" or "year* of age")) OR (AB (five or six or seven or eight or nine or ten or eleven or twelve or thirteen or fourteen or fifteen or sixteen or seventeen or eighteen) N1 ("year* old" or "year* of age")) | 27,032 |
| S42 | (TI (age* N1 ("5" or "6" or "7" or "8" or "9" or "10" or "11" or "12" or "13" or "14" or "15" or "16" or "17" or "18") N1 year*)) OR (AB (age* N1 ("5" or "6" or "7" or "8" or "9" or "10" or "11" or "12" or "13" or "14" or "15" or "16" or "17" or "18") N1 year*)) | 30,315 |
| S43 | (TI (primary or secondary or year) N1 ("1" or "2" or "3" or "4" or "5" or "6" or "7" or "8" or "9" or "10" or "11" or "12" or "13")) OR (AB (primary or secondary or year) N1 ("1" or "2" or "3" or "4" or "5" or "6" or "7" or "8" or "9" or "10" or "11" or "12" or "13")) | 186,827 |
| S44 | (TI grade# N1 (first or second or third or fourth or fifth or sixth or seventh or eighth or ninth or tenth or eleventh or twelfth)) OR (AB grade# N1 (first or second or third or fourth or fifth or sixth or seventh or eighth or ninth or tenth or eleventh or twelfth)) | 7,097 |
| S45 | S1 OR S2 OR S3 OR S4 OR S5 OR S6 OR S7 OR S8 OR S9 OR S10 OR S11 OR S12 OR S13 OR S14 OR S15 OR S16 OR S17 OR S18 OR S19 OR S20 OR S21 OR S22 OR S23 OR S24 OR S25 OR S26 OR S27 OR S28 OR S29 OR S30 OR S31 OR S32 OR S33 OR S34 OR S35 OR S36 OR S37 OR S38 OR S39 OR S40 OR S41 OR S42 OR S43 OR S44 | 1,259,777 |
| S46 | (MH "Schools, Secondary/AM/OG") OR (MH "Schools, Middle/AM/OG") OR (MH "Schools, Elementary/AM/OG") OR (MH "Schools/AM/OG") | 449 |
| S47 | (MH "School Health Services+/AM/OG") | 883 |
| S48 | (TI (school# N5 (based or level or wide or led or ethos or environment or organi#ation* or climate or toolkit# or approach* or "action group#" or practice# or whole or health* or leader* or cultur* or manag* or governance or system# or communit*))) OR (AB (school# N5 (based or level or wide or led or ethos or environment or organi#ation* or climate or toolkit# or approach* or "action group#" or practice# or whole or health* or leader* or cultur* or manag* or governance or system# or communit*))) | 36,970 |
| S49 | (TI schoolwide) or (AB schoolwide) | 130 |
| S50 | (TI "restor* justice") OR (AB "restor* justice") | 150 |
| S51 | (MH "Schools") OR (MH "Schools, Elementary") OR (MH "Schools, Middle") OR (MH "Schools, Secondary") | 21,494 |
| S52 | (TI school#) or (AB school#) | 120,896 |
| S53 | S51 OR S52 | 125,953 |
| S54 | (TI "peer-to-peer") OR (AB "peer-to-peer") | 790 |
| S55 | (TI (peer N2 (led or leader* or participat* or mediat* or helper# or resistan* or wide or action# or influen*))) OR (AB (peer N2 (led or leader* or participat* or mediat* or helper# or resistan* or wide or action# or influen*))) | 3,262 |
| S56 | (TI (relationship* N2 (led or leader* or participat* or mediat* or helper# or resistan* or wide or action# or influen*))) OR (AB (relationship* N2 (led or leader* or participat* or mediat* or helper# or resistan* or wide or action# or influen*))) | 10,707 |
| S57 | (MH "Community Networks") | 2,348 |
| S58 | (TI ((community or communities) N2 (led or leader* or participat* or mediat* or helper# or resistan* or wide or action# or influen* or relationship*))) OR (AB ((community or communities) N2 (led or leader* or participat* or mediat* or helper# or resistan* or wide or action# or influen* or relationship*))) | 12,801 |
| S59 | ((TI (pupil# N2 (led or leader* or participat* or mediat* or helper# or resistan* or wide or action# or influen* or relationship*)) OR (AB (pupil# N2 (led or leader* or participat* or mediat* or helper# or resistan* or wide or action# or influen* or relationship*))) NOT (MH "Pupil") | 142 |
| S60 | (TI ((child or children) N2 (led or leader* or participat* or mediat* or helper# or resistan* or wide or action# or influen*))) OR (AB (child or children*) N2 (led or leader* or participat* or mediat* or helper# or resistan* or wide or action# or influen*))) | 11,081 |
| S61 | (TI (("young people" or "young person") N2 (led or leader* or participat* or mediat* or helper# or resistan* or wide or action# or influen*))) OR (AB (("young people" or "young person") N2 (led or leader* or participat* or mediat* or helper# or resistan* or wide or action# or influen*))) | 318 |
| S62 | (TI ((teen or teens or teenage*) N2 (led or leader* or participat* or mediat* or helper# or resistan* or wide or action# or influen*))) OR (AB ((teen or teens or teenage*) N2 (led or leader* or participat* or mediat* or helper# or resistan* or wide or action# or influen*))) | 362 |
| S63 | (TI (adolescen* N2 (led or leader* or participat* or mediat* or helper# or resistan* or wide or action# or influen*))) OR (AB (adolescen* N2 (led or leader* or participat* or mediat* or helper# or resistan* or wide or action# or influen*))) | 3,945 |
| S64 | (TI (youth# N2 (led or leader* or participat* or mediat* or helper# or resistan* or wide or action# or influen*))) OR (AB (youth# N2 (led or leader* or participat* or mediat* or helper# or resistan* or wide or action# or influen*))) | 1,731 |
| S65 | (TI (student# N2 (led or leader* or participat* or mediat* or helper# or resistan* or wide or action# or influen* or relationship*))) OR (AB (student# N2 (led or leader* or participat* or mediat* or helper# or resistan* or wide or action# or influen* or relationship*))) NOT (MH "Students, Health Occupations+") | 6,958 |
| S66 | (TI (teach* N2 (led or leader* or participat* or mediat* or helper# or resistan* or wide or action# or influen* or relationship*))) OR (AB (teach* N2 (led or leader* or participat* or mediat* or helper# or resistan* or wide or action# or influen* or relationship*))) | 2,771 |
| S67 | (TI (curricul* N2 (led or leader* or participat* or mediat* or helper# or resistan* or wide or action# or influen* or relationship*))) OR (AB (curricul* N2 (led or leader* or participat* or mediat* or helper# or resistan* or wide or action# or influen* or relationship*))) | 747 |
| S68 | (TI (administrat* N2 (led or leader* or participat* or mediat* or helper# or resistan* or wide or action# or influen* or relationship*))) OR (AB (administrat* N2 (led or leader* or participat* or mediat* or helper# or resistan* or wide or action# or influen* or relationship*))) | 2,133 |
| S69 | (TI ((personnel or staff) N2 (led or leader* or participat* or mediat* or helper# or resistan* or wide or action# or influen* or relationship*))) OR (AB ((personnel or staff) N2 (led or leader* or participat* or mediat* or helper# or resistan* or wide or action# or influen* or relationship*))) | 4,729 |
| S70 | (TI (volunteer* N2 (led or leader* or participat* or mediat* or helper# or resistan* or wide or action# or influen* or relationship*))) OR (AB (volulnteer* N2 (led or leader* or participat* or mediat* or helper# or resistan* or wide or action# or influen* or relationship*))) | 170 |
| S71 | (TI ((advocacy or advocate) N2 (led or leader* or participat* or mediat* or helper# or resistan* or wide or action# or influen* or relationship*))) OR (AB ((advocacy or advocate) N2 (led or leader* or participat* or mediat* or helper# or resistan* or wide or action# or influen* or relationship*))) | 1,184 |
| S72 | (TI (parent* N2 (led or leader* or participat* or mediat* or helper# or resistan* or wide or action# or influen*))) OR (AB (parent* N2 (led or leader* or participat* or mediat* or helper# or resistan* or wide or action# or influen*))) | 6,638 |
| S73 | (MH "Social Environment") | 11,191 |
| S74 | (TI (environment* N2 (led or leader* or participat* or mediat* or helper# or resistan* or wide or action# or influen* or relationship*))) OR (AB (environment* N2 (led or leader* or participat* or mediat* or helper# or resistan* or wide or action# or influen* or relationship*))) | 8,069 |
| S75 | (TI ("socio-ecolog*" N2 (led or leader* or participat* or mediat* or helper# or resistan* or wide or action# or influen* or relationship*))) OR (AB ("socio-ecolog*" N2 (led or leader* or participat* or mediat* or helper# or resistan* or wide or action# or influen* or relationship*))) | 23 |
| S76 | (TI (learn* N2 (led or leader* or participat* or mediat* or helper# or resistan* or wide or action# or influen* or relationship*))) OR (AB (learn* N2 (led or leader* or participat* or mediat* or helper# or resistan* or wide or action# or influen* or relationship*))) | 4,692 |
| S77 | (MH "School Policies") | 1,803 |
| S78 | (TI ((policy or policies) N2 (led or leader* or participat* or mediat* or helper# or resistan* or wide or action# or influen* or relationship*))) OR (AB ((policy or policies) N2 (led or leader* or participat* or mediat* or helper# or resistan* or wide or action# or influen* or relationship*))) | 4,872 |
| S79 | S54 OR S55 OR S56 OR S57 OR S58 OR S59 OR S60 OR S61 OR S62 OR S63 OR S64 OR S65 OR S66 OR S67 OR S68 OR S69 OR S70 OR S71 OR S72 OR S73 OR S74 OR S75 OR S76 OR S77 OR S78 | 92,291 |
| S80 | S53 AND S79 | 13,417 |
| S81 | S46 OR S47 OR S48 OR S49 OR S50 OR S80 | 45,395 |
| S82 | (MH "Smoking Cessation Programs") | 2,184 |
| S83 | (MH "Smoking Cessation") | 19,058 |
| S84 | (MH "Smoking/PC") | 7,559 |
| S85 | (TI ((smoking or smoked or smoke or smoker) N5 (reduc* or decreas* or increas* or lower* or fewer or improv* or enhance* or extend* or develop* or prevalen* or daily or weekly or current or rate or rates or behavio#r* or knowledge or prevent* or attitude* or avoid* or perceive* or percept* or promot* or program* or change*))) OR (AB ((smoking or smoked or smoke or smoker) N5 (reduc* or decreas* or increas* or lower* or fewer or improv* or enhance* or extend* or develop* or prevalen* or daily or weekly or current or rate or rates or behavio#r* or knowledge or prevent* or attitude* or avoid* or perceive* or percept* or promot* or program* or change*))) | 33,456 |
| S86 | (TI (tobacco N5 (reduc* or decreas* or increas* or lower* or fewer or improv* or enhance* or extend* or develop* or prevalen* or daily or weekly or current or rate or rates or behavio#r* or knowledge or prevent* or attitude* or avoid* or perceive* or percept* or promot* or program* or change*)) OR (AB (tobacco N5 (reduc* or decreas* or increas* or lower* or fewer or improv* or enhance* or extend* or develop* or prevalen* or daily or weekly or current or rate or rates or behavio#r* or knowledge or prevent* or attitude* or avoid* or perceive* or percept* or promot* or program* or change*)) | 10,492 |
| S87 | (TI (cigarette# N5 (reduc* or decreas* or increas* or lower* or fewer or improv* or enhance* or extend* or develop* or prevalen* or daily or weekly or current or rate or rates or behavio#r* or knowledge or prevent* or attitude* or avoid* or perceive* or percept* or promot* or program* or change*)) OR (AB (cigarette# N5 (reduc* or decreas* or increas* or lower* or fewer or improv* or enhance* or extend* or develop* or prevalen* or daily or weekly or current or rate or rates or behavio#r* or knowledge or prevent* or attitude* or avoid* or perceive* or percept* or promot* or program* or change*)) | 7,957 |
| S88 | (TI (marijuana N5 (reduc* or decreas* or increas* or lower* or fewer or improv* or enhance* or extend* or develop* or prevalen* or daily or weekly or current or rate or rates or behavio#r* or knowledge or prevent* or attitude* or avoid* or perceive* or percept* or promot* or program* or change*)) OR (AB (marijuana N5 (reduc* or decreas* or increas* or lower* or fewer or improv* or enhance* or extend* or develop* or prevalen* or daily or weekly or current or rate or rates or behavio#r* or knowledge or prevent* or attitude* or avoid* or perceive* or percept* or promot* or program* or change*)) | 2,283 |
| S89 | (TI (cannabis N5 (reduc* or decreas* or increas* or lower* or fewer or improv* or enhance* or extend* or develop* or prevalen* or daily or weekly or current or rate or rates or behavio#r* or knowledge or prevent* or attitude* or avoid* or perceive* or percept* or promot* or program* or change*)) OR (AB (cannabis N5 (reduc* or decreas* or increas* or lower* or fewer or improv* or enhance* or extend* or develop* or prevalen* or daily or weekly or current or rate or rates or behavio#r* or knowledge or prevent* or attitude* or avoid* or perceive* or percept* or promot* or program* or change*)) | 2,440 |
| S90 | (TI (solvent# N5 (reduc* or decreas* or increas* or lower* or fewer or improv* or enhance* or extend* or develop* or prevalen* or daily or weekly or current or rate or rates or behavio#r* or knowledge or prevent* or attitude* or avoid* or perceive* or percept* or promot* or program* or change*)) OR (AB (solvent# N5 (reduc* or decreas* or increas* or lower* or fewer or improv* or enhance* or extend* or develop* or prevalen* or daily or weekly or current or rate or rates or behavio#r* or knowledge or prevent* or attitude* or avoid* or perceive* or percept* or promot* or program* or change*)) | 354 |
| S91 | (MH "Substance Use Disorders+/PC") | 18,756 |
| S92 | (MH "Alcohol Drinking/PC") OR (MH "Binge Drinking/PC") | 2,204 |
| S93 | (TI (alcohol N5 (reduc* or decreas* or increas* or lower* or fewer or improv* or enhance* or extend* or develop* or prevalen* or daily or weekly or current or rate or rates or behavio#r* or knowledge or prevent* or attitude* or avoid* or perceive* or percept* or promot* or program* or change*)) OR (AB (alcohol N5 (reduc* or decreas* or increas* or lower* or fewer or improv* or enhance* or extend* or develop* or prevalen* or daily or weekly or current or rate or rates or behavio#r* or knowledge or prevent* or attitude* or avoid* or perceive* or percept* or promot* or program* or change*)) | 24,244 |
| S94 | (TI ("binge drink*" N5 (reduc* or decreas* or increas* or lower* or fewer or improv* or enhance* or extend* or develop* or prevalen* or daily or weekly or current or rate or rates or behavio#r* or knowledge or prevent* or attitude* or avoid* or perceive* or percept* or promot* or program* or change*)) OR (AB ("binge drink*" N5 (reduc* or decreas* or increas* or lower* or fewer or improv* or enhance* or extend* or develop* or prevalen* or daily or weekly or current or rate or rates or behavio#r* or knowledge or prevent* or attitude* or avoid* or perceive* or percept* or promot* or program* or change*)) | 1,232 |
| S95 | (TI ("underage drink*" N5 (reduc* or decreas* or increas* or lower* or fewer or improv* or enhance* or extend* or develop* or prevalen* or daily or weekly or current or rate or rates or behavio#r* or knowledge or prevent* or attitude* or avoid* or perceive* or percept* or promot* or program* or change*)) OR (AB ("underage drink*" N5 (reduc* or decreas* or increas* or lower* or fewer or improv* or enhance* or extend* or develop* or prevalen* or daily or weekly or current or rate or rates or behavio#r* or knowledge or prevent* or attitude* or avoid* or perceive* or percept* or promot* or program* or change*)) | 172 |
| S96 | (TI (substance# N5 (reduc* or decreas* or increas* or lower* or fewer or improv* or enhance* or extend* or develop* or prevalen* or daily or weekly or current or rate or rates or behavio#r* or knowledge or prevent* or attitude* or avoid* or perceive* or percept* or promot* or program* or change*)) OR (AB (substance# N5 (reduc* or decreas* or increas* or lower* or fewer or improv* or enhance* or extend* or develop* or prevalen* or daily or weekly or current or rate or rates or behavio#r* or knowledge or prevent* or attitude* or avoid* or perceive* or percept* or promot* or program* or change*)) | 15,037 |
| S97 | (TI (drug# N5 (reduc* or decreas* or increas* or lower* or fewer or improv* or enhance* or extend* or develop* or prevalen* or daily or weekly or current or rate or rates or behavio#r* or knowledge or prevent* or attitude* or avoid* or perceive* or percept* or promot* or program* or change*)) OR (AB (drug# N5 (reduc* or decreas* or increas* or lower* or fewer or improv* or enhance* or extend* or develop* or prevalen* or daily or weekly or current or rate or rates or behavio#r* or knowledge or prevent* or attitude* or avoid* or perceive* or percept* or promot* or program* or change*)) | 59,613 |
| S98 | (MH "Academic Performance+") | 11,580 |
| S99 | (TI ("education* attain*" N5 (reduc* or decreas* or increas* or lower* or fewer or improv* or enhance* or extend* or develop* or prevalen* or daily or weekly or current or rate or rates or behavio#r* or knowledge or prevent* or attitude* or avoid* or perceive* or percept* or promot* or program* or change*)) OR (AB ("education* attain*" N5 (reduc* or decreas* or increas* or lower* or fewer or improv* or enhance* or extend* or develop* or prevalen* or daily or weekly or current or rate or rates or behavio#r* or knowledge or prevent* or attitude* or avoid* or perceive* or percept* or promot* or program* or change*)) | 1,188 |
| S100 | (TI ("academic attain*" N5 (reduc* or decreas* or increas* or lower* or fewer or improv* or enhance* or extend* or develop* or prevalen* or daily or weekly or current or rate or rates or behavio#r* or knowledge or prevent* or attitude* or avoid* or perceive* or percept* or promot* or program* or change*)) OR (AB ("academic attain*" N5 (reduc* or decreas* or increas* or lower* or fewer or improv* or enhance* or extend* or develop* or prevalen* or daily or weekly or current or rate or rates or behavio#r* or knowledge or prevent* or attitude* or avoid* or perceive* or percept* or promot* or program* or change*)) | 33 |
| S101 | (TI ("student* attain*" N5 (reduc* or decreas* or increas* or lower* or fewer or improv* or enhance* or extend* or develop* or prevalen* or daily or weekly or current or rate or rates or behavio#r* or knowledge or prevent* or attitude* or avoid* or perceive* or percept* or promot* or program* or change*)) OR (AB ("student* attain*" N5 (reduc* or decreas* or increas* or lower* or fewer or improv* or enhance* or extend* or develop* or prevalen* or daily or weekly or current or rate or rates or behavio#r* or knowledge or prevent* or attitude* or avoid* or perceive* or percept* or promot* or program* or change*)) | 13 |
| S102 | (TI ("education* achieve*" N5 (reduc* or decreas* or increas* or lower* or fewer or improv* or enhance* or extend* or develop* or prevalen* or daily or weekly or current or rate or rates or behavio#r* or knowledge or prevent* or attitude* or avoid* or perceive* or percept* or promot* or program* or change*)) OR (AB ("education* achieve*" N5 (reduc* or decreas* or increas* or lower* or fewer or improv* or enhance* or extend* or develop* or prevalen* or daily or weekly or current or rate or rates or behavio#r* or knowledge or prevent* or attitude* or avoid* or perceive* or percept* or promot* or program* or change*)) | 199 |
| S103 | (TI ("academic achieve*" N5 (reduc* or decreas* or increas* or lower* or fewer or improv* or enhance* or extend* or develop* or prevalen* or daily or weekly or current or rate or rates or behavio#r* or knowledge or prevent* or attitude* or avoid* or perceive* or percept* or promot* or program* or change*)) OR (AB ("academic achieve*" N5 (reduc* or decreas* or increas* or lower* or fewer or improv* or enhance* or extend* or develop* or prevalen* or daily or weekly or current or rate or rates or behavio#r* or knowledge or prevent* or attitude* or avoid* or perceive* or percept* or promot* or program* or change*)) | 845 |
| S104 | (TI ("student* achieve*" N5 (reduc* or decreas* or increas* or lower* or fewer or improv* or enhance* or extend* or develop* or prevalen* or daily or weekly or current or rate or rates or behavio#r* or knowledge or prevent* or attitude* or avoid* or perceive* or percept* or promot* or program* or change*)) OR (AB ("student* achieve*" N5 (reduc* or decreas* or increas* or lower* or fewer or improv* or enhance* or extend* or develop* or prevalen* or daily or weekly or current or rate or rates or behavio#r* or knowledge or prevent* or attitude* or avoid* or perceive* or percept* or promot* or program* or change*)) | 173 |
| S105 | (TI ("education* perform*" N5 (reduc* or decreas* or increas* or lower* or fewer or improv* or enhance* or extend* or develop* or prevalen* or daily or weekly or current or rate or rates or behavio#r* or knowledge or prevent* or attitude* or avoid* or perceive* or percept* or promot* or program* or change*)) OR (AB ("education* perform*" N5 (reduc* or decreas* or increas* or lower* or fewer or improv* or enhance* or extend* or develop* or prevalen* or daily or weekly or current or rate or rates or behavio#r* or knowledge or prevent* or attitude* or avoid* or perceive* or percept* or promot* or program* or change*)) | 58 |
| S106 | (TI ("academic perform*" N5 (reduc* or decreas* or increas* or lower* or fewer or improv* or enhance* or extend* or develop* or prevalen* or daily or weekly or current or rate or rates or behavio#r* or knowledge or prevent* or attitude* or avoid* or perceive* or percept* or promot* or program* or change*)) OR (AB ("academic perform*" N5 (reduc* or decreas* or increas* or lower* or fewer or improv* or enhance* or extend* or develop* or prevalen* or daily or weekly or current or rate or rates or behavio#r* or knowledge or prevent* or attitude* or avoid* or perceive* or percept* or promot* or program* or change*)) | 976 |
| S107 | (TI ("student* perform*" N5 (reduc* or decreas* or increas* or lower* or fewer or improv* or enhance* or extend* or develop* or prevalen* or daily or weekly or current or rate or rates or behavio#r* or knowledge or prevent* or attitude* or avoid* or perceive* or percept* or promot* or program* or change*)) OR (AB ("student* perform*" N5 (reduc* or decreas* or increas* or lower* or fewer or improv* or enhance* or extend* or develop* or prevalen* or daily or weekly or current or rate or rates or behavio#r* or knowledge or prevent* or attitude* or avoid* or perceive* or percept* or promot* or program* or change*)) | 480 |
| S108 | (TI ("education* outcome#" N5 (reduc* or decreas* or increas* or lower* or fewer or improv* or enhance* or extend* or develop* or prevalen* or daily or weekly or current or rate or rates or behavio#r* or knowledge or prevent* or attitude* or avoid* or perceive* or percept* or promot* or program* or change*)) OR (AB ("education* outcome#" N5 (reduc* or decreas* or increas* or lower* or fewer or improv* or enhance* or extend* or develop* or prevalen* or daily or weekly or current or rate or rates or behavio#r* or knowledge or prevent* or attitude* or avoid* or perceive* or percept* or promot* or program* or change*)) | 422 |
| S109 | (TI ("academic outcome#" N5 (reduc* or decreas* or increas* or lower* or fewer or improv* or enhance* or extend* or develop* or prevalen* or daily or weekly or current or rate or rates or behavio#r* or knowledge or prevent* or attitude* or avoid* or perceive* or percept* or promot* or program* or change*)) OR (AB ("academic outcome#" N5 (reduc* or decreas* or increas* or lower* or fewer or improv* or enhance* or extend* or develop* or prevalen* or daily or weekly or current or rate or rates or behavio#r* or knowledge or prevent* or attitude* or avoid* or perceive* or percept* or promot* or program* or change*)) | 231 |
| S110 | (TI ("student* outcome#" N5 (reduc* or decreas* or increas* or lower* or fewer or improv* or enhance* or extend* or develop* or prevalen* or daily or weekly or current or rate or rates or behavio#r* or knowledge or prevent* or attitude* or avoid* or perceive* or percept* or promot* or program* or change*)) OR (AB ("student* outcome#" N5 (reduc* or decreas* or increas* or lower* or fewer or improv* or enhance* or extend* or develop* or prevalen* or daily or weekly or current or rate or rates or behavio#r* or knowledge or prevent* or attitude* or avoid* or perceive* or percept* or promot* or program* or change*)) | 208 |
| S111 | (TI ("education* commitment" N5 (reduc* or decreas* or increas* or lower* or fewer or improv* or enhance* or extend* or develop* or prevalen* or daily or weekly or current or rate or rates or behavio#r* or knowledge or prevent* or attitude* or avoid* or perceive* or percept* or promot* or program* or change*)) OR (AB ("education* commitment" N5 (reduc* or decreas* or increas* or lower* or fewer or improv* or enhance* or extend* or develop* or prevalen* or daily or weekly or current or rate or rates or behavio#r* or knowledge or prevent* or attitude* or avoid* or perceive* or percept* or promot* or program* or change*)) | 3 |
| S112 | (TI ("academic commitment" N5 (reduc* or decreas* or increas* or lower* or fewer or improv* or enhance* or extend* or develop* or prevalen* or daily or weekly or current or rate or rates or behavio#r* or knowledge or prevent* or attitude* or avoid* or perceive* or percept* or promot* or program* or change*)) OR (AB ("academic commitment" N5 (reduc* or decreas* or increas* or lower* or fewer or improv* or enhance* or extend* or develop* or prevalen* or daily or weekly or current or rate or rates or behavio#r* or knowledge or prevent* or attitude* or avoid* or perceive* or percept* or promot* or program* or change*)) | 5 |
| S113 | (TI ("student* commitment" N5 (reduc* or decreas* or increas* or lower* or fewer or improv* or enhance* or extend* or develop* or prevalen* or daily or weekly or current or rate or rates or behavio#r* or knowledge or prevent* or attitude* or avoid* or perceive* or percept* or promot* or program* or change*)) OR (AB ("student* commitment" N5 (reduc* or decreas* or increas* or lower* or fewer or improv* or enhance* or extend* or develop* or prevalen* or daily or weekly or current or rate or rates or behavio#r* or knowledge or prevent* or attitude* or avoid* or perceive* or percept* or promot* or program* or change*)) | 17 |
| S114 | (TI ("education* engage*" N5 (reduc* or decreas* or increas* or lower* or fewer or improv* or enhance* or extend* or develop* or prevalen* or daily or weekly or current or rate or rates or behavio#r* or knowledge or prevent* or attitude* or avoid* or perceive* or percept* or promot* or program* or change*)) OR (AB ("education* engage*" N5 (reduc* or decreas* or increas* or lower* or fewer or improv* or enhance* or extend* or develop* or prevalen* or daily or weekly or current or rate or rates or behavio#r* or knowledge or prevent* or attitude* or avoid* or perceive* or percept* or promot* or program* or change*)) | 12 |
| S115 | (TI ("academic engage*" N5 (reduc* or decreas* or increas* or lower* or fewer or improv* or enhance* or extend* or develop* or prevalen* or daily or weekly or current or rate or rates or behavio#r* or knowledge or prevent* or attitude* or avoid* or perceive* or percept* or promot* or program* or change*)) OR (AB ("academic engage*" N5 (reduc* or decreas* or increas* or lower* or fewer or improv* or enhance* or extend* or develop* or prevalen* or daily or weekly or current or rate or rates or behavio#r* or knowledge or prevent* or attitude* or avoid* or perceive* or percept* or promot* or program* or change*)) | 81 |
| S116 | (TI ("student* engage*" N5 (reduc* or decreas* or increas* or lower* or fewer or improv* or enhance* or extend* or develop* or prevalen* or daily or weekly or current or rate or rates or behavio#r* or knowledge or prevent* or attitude* or avoid* or perceive* or percept* or promot* or program* or change*)) OR (AB ("student* engage*" N5 (reduc* or decreas* or increas* or lower* or fewer or improv* or enhance* or extend* or develop* or prevalen* or daily or weekly or current or rate or rates or behavio#r* or knowledge or prevent* or attitude* or avoid* or perceive* or percept* or promot* or program* or change*)) | 392 |
| S117 | (TI (dropout N5 (reduc* or decreas* or increas* or lower* or fewer or improv* or enhance* or extend* or develop* or prevalen* or daily or weekly or current or rate or rates or behavio#r* or knowledge or prevent* or attitude* or avoid* or perceive* or percept* or promot* or program* or change*)) OR (AB (dropout N5 (reduc* or decreas* or increas* or lower* or fewer or improv* or enhance* or extend* or develop* or prevalen* or daily or weekly or current or rate or rates or behavio#r* or knowledge or prevent* or attitude* or avoid* or perceive* or percept* or promot* or program* or change*)) | 1,793 |
| S118 | (TI (absentee* N5 (reduc* or decreas* or increas* or lower* or fewer or improv* or enhance* or extend* or develop* or prevalen* or daily or weekly or current or rate or rates or behavio#r* or knowledge or prevent* or attitude* or avoid* or perceive* or percept* or promot* or program* or change*)) OR (AB (absentee* N5 (reduc* or decreas* or increas* or lower* or fewer or improv* or enhance* or extend* or develop* or prevalen* or daily or weekly or current or rate or rates or behavio#r* or knowledge or prevent* or attitude* or avoid* or perceive* or percept* or promot* or program* or change*)) | 1,079 |
| S119 | (MH "Aggression/PC") OR (MH "Bullying+/PC") OR (MH "Student Abuse/PC") OR (MH "Verbal Abuse/PC") OR (MH "Violence/PC") OR (MH "Exposure to Violence/PC") OR (MH "Gender-Based Violence/PC") OR (MH "Gun Violence/PC") OR (MH "School Violence/PC") OR (MH "Assault and Battery/PC") | 6,388 |
| S120 | (MH "Bullying+/PC") | 1,689 |
| S121 | (MH "Juvenile Delinquency/PC") | 412 |
| S122 | (MH "Student Discipline") | 526 |
| S123 | (MH "Disruptive Behavior/PC") | 560 |
| S124 | (TI ((violent or violence) N5 (reduc* or decreas* or increas* or lower* or fewer or improv* or enhance* or extend* or develop* or prevalen* or daily or weekly or current or rate or rates or behavio#r* or knowledge or prevent* or attitude* or avoid* or perceive* or percept* or promot* or program* or change*))) OR (AB ((violent or violence) N5 (reduc* or decreas* or increas* or lower* or fewer or improv* or enhance* or extend* or develop* or prevalen* or daily or weekly or current or rate or rates or behavio#r* or knowledge or prevent* or attitude* or avoid* or perceive* or percept* or promot* or program* or change*))) | 13,183 |
| S125 | (TI ((aggression or aggressive) N5 (reduc* or decreas* or increas* or lower* or fewer or improv* or enhance* or extend* or develop* or prevalen* or daily or weekly or current or rate or rates or behavio#r* or knowledge or prevent* or attitude* or avoid* or perceive* or percept* or promot* or program* or change*))) OR (AB ((aggression or aggressive) N5 (reduc* or decreas* or increas* or lower* or fewer or improv* or enhance* or extend* or develop* or prevalen* or daily or weekly or current or rate or rates or behavio#r* or knowledge or prevent* or attitude* or avoid* or perceive* or percept* or promot* or program* or change*))) | 10,397 |
| S126 | (TI ((hostile or hostility) N5 (reduc* or decreas* or increas* or lower* or fewer or improv* or enhance* or extend* or develop* or prevalen* or daily or weekly or current or rate or rates or behavio#r* or knowledge or prevent* or attitude* or avoid* or perceive* or percept* or promot* or program* or change*))) OR (AB ((hostile or hostility) N5 (reduc* or decreas* or increas* or lower* or fewer or improv* or enhance* or extend* or develop* or prevalen* or daily or weekly or current or rate or rates or behavio#r* or knowledge or prevent* or attitude* or avoid* or perceive* or percept* or promot* or program* or change*))) | 1,040 |
| S127 | (TI (assault* N5 (reduc* or decreas* or increas* or lower* or fewer or improv* or enhance* or extend* or develop* or prevalen* or daily or weekly or current or rate or rates or behavio#r* or knowledge or prevent* or attitude* or avoid* or perceive* or percept* or promot* or program* or change*)) OR (AB (assault* N5 (reduc* or decreas* or increas* or lower* or fewer or improv* or enhance* or extend* or develop* or prevalen* or daily or weekly or current or rate or rates or behavio#r* or knowledge or prevent* or attitude* or avoid* or perceive* or percept* or promot* or program* or change*)) | 1,744 |
| S128 | (TI (crime N5 (reduc* or decreas* or increas* or lower* or fewer or improv* or enhance* or extend* or develop* or prevalen* or daily or weekly or current or rate or rates or behavio#r* or knowledge or prevent* or attitude* or avoid* or perceive* or percept* or promot* or program* or change*)) OR (AB (crime N5 (reduc* or decreas* or increas* or lower* or fewer or improv* or enhance* or extend* or develop* or prevalen* or daily or weekly or current or rate or rates or behavio#r* or knowledge or prevent* or attitude* or avoid* or perceive* or percept* or promot* or program* or change*)) | 1,646 |
| S129 | (TI ((abuse# or abusive) N5 (reduc* or decreas* or increas* or lower* or fewer or improv* or enhance* or extend* or develop* or prevalen* or daily or weekly or current or rate or rates or behavio#r* or knowledge or prevent* or attitude* or avoid* or perceive* or percept* or promot* or program* or change*))) OR (AB ((abuse# or abusive) N5 (reduc* or decreas* or increas* or lower* or fewer or improv* or enhance* or extend* or develop* or prevalen* or daily or weekly or current or rate or rates or behavio#r* or knowledge or prevent* or attitude* or avoid* or perceive* or percept* or promot* or program* or change*))) | 14,318 |
| S130 | (TI (misbehav* N5 (reduc* or decreas* or increas* or lower* or fewer or improv* or enhance* or extend* or develop* or prevalen* or daily or weekly or current or rate or rates or behavio#r* or knowledge or prevent* or attitude* or avoid* or perceive* or percept* or promot* or program* or change*)) OR (AB (misbehav* N5 (reduc* or decreas* or increas* or lower* or fewer or improv* or enhance* or extend* or develop* or prevalen* or daily or weekly or current or rate or rates or behavio#r* or knowledge or prevent* or attitude* or avoid* or perceive* or percept* or promot* or program* or change*)) | 64 |
| S131 | (TI (threat# N5 (reduc* or decreas* or increas* or lower* or fewer or improv* or enhance* or extend* or develop* or prevalen* or daily or weekly or current or rate or rates or behavio#r* or knowledge or prevent* or attitude* or avoid* or perceive* or percept* or promot* or program* or change*)) OR (AB (threat# N5 (reduc* or decreas* or increas* or lower* or fewer or improv* or enhance* or extend* or develop* or prevalen* or daily or weekly or current or rate or rates or behavio#r* or knowledge or prevent* or attitude* or avoid* or perceive* or percept* or promot* or program* or change*)) | 4,220 |
| S132 | (TI (conduct N5 (reduc* or decreas* or increas* or lower* or fewer or improv* or enhance* or extend* or develop* or prevalen* or daily or weekly or current or rate or rates or behavio#r* or knowledge or prevent* or attitude* or avoid* or perceive* or percept* or promot* or program* or change*)) OR (AB (conduct N5 (reduc* or decreas* or increas* or lower* or fewer or improv* or enhance* or extend* or develop* or prevalen* or daily or weekly or current or rate or rates or behavio#r* or knowledge or prevent* or attitude* or avoid* or perceive* or percept* or promot* or program* or change*)) | 4,217 |
| S133 | (TI (delinquen* N5 (reduc* or decreas* or increas* or lower* or fewer or improv* or enhance* or extend* or develop* or prevalen* or daily or weekly or current or rate or rates or behavio#r* or knowledge or prevent* or attitude* or avoid* or perceive* or percept* or promot* or program* or change*)) OR (AB (delinquen* N5 (reduc* or decreas* or increas* or lower* or fewer or improv* or enhance* or extend* or develop* or prevalen* or daily or weekly or current or rate or rates or behavio#r* or knowledge or prevent* or attitude* or avoid* or perceive* or percept* or promot* or program* or change*)) | 1,265 |
| S134 | (TI ("disrupt* behavio#r*" N5 (reduc* or decreas* or increas* or lower* or fewer or improv* or enhance* or extend* or develop* or prevalen* or daily or weekly or current or rate or rates or behavio#r* or knowledge or prevent* or attitude* or avoid* or perceive* or percept* or promot* or program* or change*)) OR (AB ("disrupt* behavio#r*" N5 (reduc* or decreas* or increas* or lower* or fewer or improv* or enhance* or extend* or develop* or prevalen* or daily or weekly or current or rate or rates or behavio#r* or knowledge or prevent* or attitude* or avoid* or perceive* or percept* or promot* or program* or change*)) | 1,866 |
| S135 | (TI ("problem* behavio#r*" N5 (reduc* or decreas* or increas* or lower* or fewer or improv* or enhance* or extend* or develop* or prevalen* or daily or weekly or current or rate or rates or behavio#r* or knowledge or prevent* or attitude* or avoid* or perceive* or percept* or promot* or program* or change*)) OR (AB ("problem* behavio#r*" N5 (reduc* or decreas* or increas* or lower* or fewer or improv* or enhance* or extend* or develop* or prevalen* or daily or weekly or current or rate or rates or behavio#r* or knowledge or prevent* or attitude* or avoid* or perceive* or percept* or promot* or program* or change*)) | 2,997 |
| S136 | (TI ("volatile behavio#r*" N5 (reduc* or decreas* or increas* or lower* or fewer or improv* or enhance* or extend* or develop* or prevalen* or daily or weekly or current or rate or rates or behavio#r* or knowledge or prevent* or attitude* or avoid* or perceive* or percept* or promot* or program* or change*)) OR (AB ("volatile behavio#r*" N5 (reduc* or decreas* or increas* or lower* or fewer or improv* or enhance* or extend* or develop* or prevalen* or daily or weekly or current or rate or rates or behavio#r* or knowledge or prevent* or attitude* or avoid* or perceive* or percept* or promot* or program* or change*)) | 5 |
| S137 | (TI ((antisocial or "anti-social") N5 (reduc* or decreas* or increas* or lower* or fewer or improv* or enhance* or extend* or develop* or prevalen* or daily or weekly or current or rate or rates or behavio#r* or knowledge or prevent* or attitude* or avoid* or perceive* or percept* or promot* or program* or change*))) OR (AB ((antisocial or "anti-social") N5 (reduc* or decreas* or increas* or lower* or fewer or improv* or enhance* or extend* or develop* or prevalen* or daily or weekly or current or rate or rates or behavio#r* or knowledge or prevent* or attitude* or avoid* or perceive* or percept* or promot* or program* or change*))) | 1,918 |
| S138 | (TI (perpetrat* N5 (reduc* or decreas* or increas* or lower* or fewer or improv* or enhance* or extend* or develop* or prevalen* or daily or weekly or current or rate or rates or behavio#r* or knowledge or prevent* or attitude* or avoid* or perceive* or percept* or promot* or program* or change*)) OR (AB (perpetrat* N5 (reduc* or decreas* or increas* or lower* or fewer or improv* or enhance* or extend* or develop* or prevalen* or daily or weekly or current or rate or rates or behavio#r* or knowledge or prevent* or attitude* or avoid* or perceive* or percept* or promot* or program* or change*)) | 1,338 |
| S139 | (TI (bully* N5 (reduc* or decreas* or increas* or lower* or fewer or improv* or enhance* or extend* or develop* or prevalen* or daily or weekly or current or rate or rates or behavio#r* or knowledge or prevent* or attitude* or avoid* or perceive* or percept* or promot* or program* or change*)) OR (AB (bully* N5 (reduc* or decreas* or increas* or lower* or fewer or improv* or enhance* or extend* or develop* or prevalen* or daily or weekly or current or rate or rates or behavio#r* or knowledge or prevent* or attitude* or avoid* or perceive* or percept* or promot* or program* or change*)) | 2,201 |
| S140 | (TI (victim* N5 (reduc* or decreas* or increas* or lower* or fewer or improv* or enhance* or extend* or develop* or prevalen* or daily or weekly or current or rate or rates or behavio#r* or knowledge or prevent* or attitude* or avoid* or perceive* or percept* or promot* or program* or change*)) OR (AB (victim* N5 (reduc* or decreas* or increas* or lower* or fewer or improv* or enhance* or extend* or develop* or prevalen* or daily or weekly or current or rate or rates or behavio#r* or knowledge or prevent* or attitude* or avoid* or perceive* or percept* or promot* or program* or change*)) | 5,165 |
| S141 | (TI (cyberbully* N5 (reduc* or decreas* or increas* or lower* or fewer or improv* or enhance* or extend* or develop* or prevalen* or daily or weekly or current or rate or rates or behavio#r* or knowledge or prevent* or attitude* or avoid* or perceive* or percept* or promot* or program* or change*)) OR (AB (cyberbully* N5 (reduc* or decreas* or increas* or lower* or fewer or improv* or enhance* or extend* or develop* or prevalen* or daily or weekly or current or rate or rates or behavio#r* or knowledge or prevent* or attitude* or avoid* or perceive* or percept* or promot* or program* or change*)) | 293 |
| S142 | (TI (conflict* N5 (reduc* or decreas* or increas* or lower* or fewer or improv* or enhance* or extend* or develop* or prevalen* or daily or weekly or current or rate or rates or behavio#r* or knowledge or prevent* or attitude* or avoid* or perceive* or percept* or promot* or program* or change*)) OR (AB (conflict* N5 (reduc* or decreas* or increas* or lower* or fewer or improv* or enhance* or extend* or develop* or prevalen* or daily or weekly or current or rate or rates or behavio#r* or knowledge or prevent* or attitude* or avoid* or perceive* or percept* or promot* or program* or change*)) | 7,243 |
| S143 | (TI ("positive behavio#r*" N5 (reduc* or decreas* or increas* or lower* or fewer or improv* or enhance* or extend* or develop* or prevalen* or daily or weekly or current or rate or rates or behavio#r* or knowledge or prevent* or attitude* or avoid* or perceive* or percept* or promot* or program* or change*)) OR (AB ("positive behavio#r*" N5 (reduc* or decreas* or increas* or lower* or fewer or improv* or enhance* or extend* or develop* or prevalen* or daily or weekly or current or rate or rates or behavio#r* or knowledge or prevent* or attitude* or avoid* or perceive* or percept* or promot* or program* or change*)) | 1,184 |
| S144 | (TI ("improv* behavio#r*" N5 (reduc* or decreas* or increas* or lower* or fewer or improv* or enhance* or extend* or develop* or prevalen* or daily or weekly or current or rate or rates or behavio#r* or knowledge or prevent* or attitude* or avoid* or perceive* or percept* or promot* or program* or change*)) OR (AB ("improv* behavio#r*" N5 (reduc* or decreas* or increas* or lower* or fewer or improv* or enhance* or extend* or develop* or prevalen* or daily or weekly or current or rate or rates or behavio#r* or knowledge or prevent* or attitude* or avoid* or perceive* or percept* or promot* or program* or change*)) | 597 |
| S145 | (TI ("social behavio#r*" N5 (reduc* or decreas* or increas* or lower* or fewer or improv* or enhance* or extend* or develop* or prevalen* or daily or weekly or current or rate or rates or behavio#r* or knowledge or prevent* or attitude* or avoid* or perceive* or percept* or promot* or program* or change*)) OR (AB ("social behavio#r*" N5 (reduc* or decreas* or increas* or lower* or fewer or improv* or enhance* or extend* or develop* or prevalen* or daily or weekly or current or rate or rates or behavio#r* or knowledge or prevent* or attitude* or avoid* or perceive* or percept* or promot* or program* or change*)) | 2,260 |
| S146 | (TI (("social-emotion*" or "socio-emotion*") N5 (reduc* or decreas* or increas* or lower* or fewer or improv* or enhance* or extend* or develop* or prevalen* or daily or weekly or current or rate or rates or behavio#r* or knowledge or prevent* or attitude* or avoid* or perceive* or percept* or promot* or program* or change*))) OR (AB (("social-emotion*" or "socio-emotion*") N5 (reduc* or decreas* or increas* or lower* or fewer or improv* or enhance* or extend* or develop* or prevalen* or daily or weekly or current or rate or rates or behavio#r* or knowledge or prevent* or attitude* or avoid* or perceive* or percept* or promot* or program* or change*))) | 1,276 |
| S147 | (TI ("social-charact*" N5 (reduc* or decreas* or increas* or lower* or fewer or improv* or enhance* or extend* or develop* or prevalen* or daily or weekly or current or rate or rates or behavio#r* or knowledge or prevent* or attitude* or avoid* or perceive* or percept* or promot* or program* or change*)) OR (AB ("social-charact*" N5 (reduc* or decreas* or increas* or lower* or fewer or improv* or enhance* or extend* or develop* or prevalen* or daily or weekly or current or rate or rates or behavio#r* or knowledge or prevent* or attitude* or avoid* or perceive* or percept* or promot* or program* or change*)) | 90 |
| S148 | (TI ("social-inclusion" N5 (reduc* or decreas* or increas* or lower* or fewer or improv* or enhance* or extend* or develop* or prevalen* or daily or weekly or current or rate or rates or behavio#r* or knowledge or prevent* or attitude* or avoid* or perceive* or percept* or promot* or program* or change*)) OR (AB ("social-inclusion" N5 (reduc* or decreas* or increas* or lower* or fewer or improv* or enhance* or extend* or develop* or prevalen* or daily or weekly or current or rate or rates or behavio#r* or knowledge or prevent* or attitude* or avoid* or perceive* or percept* or promot* or program* or change*)) | 458 |
| S149 | (TI ("social develop*" N5 (reduc* or decreas* or increas* or lower* or fewer or improv* or enhance* or extend* or develop* or prevalen* or daily or weekly or current or rate or rates or behavio#r* or knowledge or prevent* or attitude* or avoid* or perceive* or percept* or promot* or program* or change*)) OR (AB ("social develop*" N5 (reduc* or decreas* or increas* or lower* or fewer or improv* or enhance* or extend* or develop* or prevalen* or daily or weekly or current or rate or rates or behavio#r* or knowledge or prevent* or attitude* or avoid* or perceive* or percept* or promot* or program* or change*)) | 1,197 |
| S150 | (TI ("social competen*" N5 (reduc* or decreas* or increas* or lower* or fewer or improv* or enhance* or extend* or develop* or prevalen* or daily or weekly or current or rate or rates or behavio#r* or knowledge or prevent* or attitude* or avoid* or perceive* or percept* or promot* or program* or change*)) OR (AB ("social competen*" N5 (reduc* or decreas* or increas* or lower* or fewer or improv* or enhance* or extend* or develop* or prevalen* or daily or weekly or current or rate or rates or behavio#r* or knowledge or prevent* or attitude* or avoid* or perceive* or percept* or promot* or program* or change*)) | 611 |
| S151 | (TI ("emotion* develop*" N5 (reduc* or decreas* or increas* or lower* or fewer or improv* or enhance* or extend* or develop* or prevalen* or daily or weekly or current or rate or rates or behavio#r* or knowledge or prevent* or attitude* or avoid* or perceive* or percept* or promot* or program* or change*)) OR (AB ("emotion* develop*" N5 (reduc* or decreas* or increas* or lower* or fewer or improv* or enhance* or extend* or develop* or prevalen* or daily or weekly or current or rate or rates or behavio#r* or knowledge or prevent* or attitude* or avoid* or perceive* or percept* or promot* or program* or change*)) | 854 |
| S152 | (TI (conduct N5 (reduc* or decreas* or increas* or lower* or fewer or improv* or enhance* or extend* or develop* or prevalen* or daily or weekly or current or rate or rates or behavio#r* or knowledge or prevent* or attitude* or avoid* or perceive* or percept* or promot* or program* or change*)) OR (AB (conduct N5 (reduc* or decreas* or increas* or lower* or fewer or improv* or enhance* or extend* or develop* or prevalen* or daily or weekly or current or rate or rates or behavio#r* or knowledge or prevent* or attitude* or avoid* or perceive* or percept* or promot* or program* or change*)) | 4,217 |
| S153 | (TI (respect N5 (reduc* or decreas* or increas* or lower* or fewer or improv* or enhance* or extend* or develop* or prevalen* or daily or weekly or current or rate or rates or behavio#r* or knowledge or prevent* or attitude* or avoid* or perceive* or percept* or promot* or program* or change*)) OR (AB (respect N5 (reduc* or decreas* or increas* or lower* or fewer or improv* or enhance* or extend* or develop* or prevalen* or daily or weekly or current or rate or rates or behavio#r* or knowledge or prevent* or attitude* or avoid* or perceive* or percept* or promot* or program* or change*)) | 7,656 |
| S154 | (TI ((safety or safe) N5 (reduc* or decreas* or increas* or lower* or fewer or improv* or enhance* or extend* or develop* or prevalen* or daily or weekly or current or rate or rates or behavio#r* or knowledge or prevent* or attitude* or avoid* or perceive* or percept* or promot* or program* or change*))) OR (AB ((safety or safe) N5 (reduc* or decreas* or increas* or lower* or fewer or improv* or enhance* or extend* or develop* or prevalen* or daily or weekly or current or rate or rates or behavio#r* or knowledge or prevent* or attitude* or avoid* or perceive* or percept* or promot* or program* or change*))) | 47,570 |
| S155 | S82 OR S83 OR S84 OR S85 OR S86 OR S87 OR S88 OR S89 OR S90 OR S91 OR S92 OR S93 OR S94 OR S95 OR S96 OR S97 OR S98 OR S99 OR S100 OR S101 OR S102 OR S103 OR S104 OR S105 OR S106 OR S107 OR S108 OR S109 OR S110 OR S111 OR S112 OR S113 OR S114 OR S115 OR S116 OR S117 OR S118 OR S119 OR S120 OR S121 OR S122 OR S123 OR S124 OR S125 OR S126 OR S127 OR S128 OR S129 OR S130 OR S131 OR S132 OR S133 OR S134 OR S135 OR S136 OR S137 OR S138 OR S139 OR S140 OR S141 OR S142 OR S143 OR S144 OR S145 OR S146 OR S147 OR S148 OR S149 OR S150 OR S151 OR S152 OR S153 OR S154 | 281,177 |
| S156 | (MH "Program Development+") | 75,647 |
| S157 | (MH "Evaluation Research+") | 160,711 |
| S158 | (MH "Validation Studies") | 162,963 |
| S159 | (MH "Clinical Trials+") | 272,392 |
| S160 | (TI prevent*) OR (AB prevent*) | 322,507 |
| S161 | (TI program*) OR (AB program*) | 322,477 |
| S162 | (TI intervent*) OR (AB intervent*) | 387,227 |
| S163 | (TI "restorative approach*") OR (AB "restorative approach*") | 73 |
| S164 | (TI "control school#") OR (AB "control school#") | 439 |
| S165 | (TI (trial or trials)) OR (AB (trial or trials)) | 301,818 |
| S166 | S156 OR S157 OR S158 OR S159 OR S160 OR S161 OR S162 OR S163 OR S164 OR S165 | 1,316,679 |
| S167 | S45 AND S81 AND S155 AND S166 | 6,011 |

## ClinicalTrials.gov

| Database name | ClinicalTrials.gov |
| --- | --- |
| Database platform | [www.clinicaltrials.gov](http://www.clinicaltrials.gov) |
| Dates of database coverage | Complete database to date searched. |
| Date searched | 27/01/2020 |
| Searched by | JF |
| Number of results | 991 |
| EndNote import order | 20 |
| Number of results once duplicates removed | 935 |
| Search strategy notes | None |

Title: school OR schools OR schoolwide

## Wiley Cochrane Library

| Database name | Cochrane Library |
| --- | --- |
| Database platform | Wiley |
| Dates of database coverage | Issue 1 or 12, January 2020 |
| Date searched | 27/01/2020 |
| Searched by | JF |
| Number of results | Alcohol & drugs   - CDSR: 25 - CENTRAL: 1494   Academic attainment   - CDSR: 3 - CENTRAL: 358   Violence   - CDSR: 22 - CENTRAL: 1414 |
| EndNote import order | 19 |
| Number of results once duplicates removed | 268 |
| Search strategy notes | * is used for truncation. ? is used for an optional wildcard. Search strings ending with :ti,ab,kw search the title, abstract and keywords only. NEXT searches for terms next to each other, in the order entered. NEAR/*n* searches for terms within *n* words of each other.  Due to interface issues, the search was split into three parts:   1. population and whole-school interventions which have an impact on levels of violence, 2. population and whole-school interventions which have an impact on substance abuse, 3. population and whole school interventions which have an impact on educational attainment   As the Cochrane Library contains systematic reviews or trials, the limit to quantitative methodologies was not added. |

| ID | Search | Hits |
| --- | --- | --- |
| #1 | MeSH descriptor: [Child] this term only | 1120 |
| #2 | MeSH descriptor: [Adolescent] this term only | 102003 |
| #3 | MeSH descriptor: [Minors] this term only | 8 |
| #4 | MeSH descriptor: [Students] this term only | 2547 |
| #5 | MeSH descriptor: [School Teachers] this term only | 79 |
| #6 | MeSH descriptor: [Parents] explode all trees | 4661 |
| #7 | (child*):ti,ab,kw | 148520 |
| #8 | (boy?):ti,ab,kw | 6527 |
| #9 | (girl?):ti,ab,kw | 6994 |
| #10 | (schoolage or (school near/1 age)):ti,ab,kw | 1205 |
| #11 | (schoolchild*):ti,ab,kw | 1390 |
| #12 | (youngster?):ti,ab,kw | 155 |
| #13 | (minor?):ti,ab,kw | 16093 |
| #14 | (pupil* or student*):ti,ab,kw | 36541 |
| #15 | MeSH descriptor: [Pupil] this term only | 663 |
| #16 | MeSH descriptor: [Students, Health Occupations] explode all trees | 1581 |
| #17 | #15 or #16 | 2243 |
| #18 | #14 NOT #17 | 34298 |
| #19 | (prepubescen*):ti,ab,kw | 93 |
| #20 | (pubescent*):ti,ab,kw | 49 |
| #21 | (adolescent*):ti,ab,kw | 132435 |
| #22 | (juvenil*):ti,ab,kw | 3498 |
| #23 | (underage*):ti,ab,kw | 234 |
| #24 | (preteen* or pre-teen*):ti,ab,kw | 50 |
| #25 | (teen or teens):ti,ab,kw | 1354 |
| #26 | (teenage*):ti,ab,kw | 1221 |
| #27 | (youth or youths):ti,ab,kw | 6514 |
| #28 | (young NEXT person*):ti,ab,kw | 249 |
| #29 | (young NEXT people*):ti,ab,kw | 2170 |
| #30 | (kid or kids):ti,ab,kw | 981 |
| #31 | (transition NEAR/4 adult*):ti,ab,kw | 226 |
| #32 | (emerging NEXT adult*):ti,ab,kw | 141 |
| #33 | (young NEXT adult*):ti,ab,kw | 77963 |
| #34 | (parent?):ti,ab,kw | 26471 |
| #35 | (mother?):ti,ab,kw | 16586 |
| #36 | (father?):ti,ab,kw | 1323 |
| #37 | (guardian?):ti,ab,kw | 3393 |
| #38 | (teacher?):ti,ab,kw | 5003 |
| #39 | ((5 or 6 or 7 or 8 or 9 or 10 or 11 or 12 or 13 or 14 or 15 or 16 or 17 or 19) NEAR/1 (year* NEXT old or year* NEXT age)):ti,ab,kw | 11426 |
| #40 | ((five or six or seven or eight or nine or ten or eleven or twelve or thirteen or fourteen or fifteen or sixteen or seventeen or eighteen) NEAR/1 (year* NEXT old or year* NEXT age)):ti,ab,kw | 593 |
| #41 | ((primary or secondary or year) NEAR/1 (1 or 2 or 3 or 4 or 5 or 6 or 7 or 8 or 9 or 10 or 11 or 12 or 13)):ti,ab,kw | 91913 |
| #42 | (grade? NEAR/1 (first or second or third or fourth or fifth or sixth or seventh or eighth or ninth or tenth or eleventh or twelfth)):ti,ab,kw | 1668 |
| #43 | #1 or #2 or #3 or #4 or #5 or #6 or #7 or #8 or #9 or #10 or #11 or #12 or #13 or #18 or #19 or #20 or #21 or #22 or #23 or #24 or #25 or #26 or #27 or #28 or #29 or #30 or #31 or #32 or #33 or #34 or #35 or #36 or #37 or #38 or #39 or #40 or #41 or #42 | 396981 |
| #44 | MeSH descriptor: [Schools] this term only and with qualifier(s): [organization & administration - OG] | 78 |
| #45 | MeSH descriptor: [School Health Services] this term only and with qualifier(s): [organization & administration - OG] | 433 |
| #46 | (school? NEAR/5 (based or level or wide or led or ethos or environment or organi?ation* or climate or toolkit? or approach* or (action NEXT group?) or practice? or whole or health* or leader* or cultur* or manag* or governance or system? or communit*)):ti,ab,kw | 8907 |
| #47 | (schoolwide):ti,ab,kw | 84 |
| #48 | (restor* NEXT justice):ti,ab,kw | 15 |
| #49 | MeSH descriptor: [Schools] this term only | 1842 |
| #50 | (school?):ti,ab,kw | 31309 |
| #51 | #49 or #50 | 31309 |
| #52 | (peer-to-peer):ti,ab,kw | 136 |
| #53 | (peer NEAR/2 (led or leader* or participat* or mediat* or helper? or resistan* or wide or action? or influen*)):ti,ab,kw | 980 |
| #54 | (relationship* NEAR/2 (led or leader* or participat* or mediat* or helper? or resistan* or wide or action? or influen*)):ti,ab,kw | 877 |
| #55 | MeSH descriptor: [Community Networks] this term only | 160 |
| #56 | MeSH descriptor: [Community Participation] this term only | 276 |
| #57 | ((community or communities) NEAR/2 (led or leader* or participat* or mediat* or helper? or resistan* or wide or action? or influen* or relationship*)):ti,ab,kw | 2160 |
| #58 | (pupil? NEAR/2 (led or leader* or participat* or mediat* or helper? or resistan* or wide or action? or influen* or relationship*)):ti,ab,kw | 59 |
| #59 | #58 NOT #15 | 50 |
| #60 | ((child or children) NEAR/2 (led or leader* or participat* or mediat* or helper? or resistan* or wide or action? or influen*)):ti,ab,kw | 2622 |
| #61 | ((young people or young person) NEAR/2 (led or leader* or participat* or mediat* or helper? or resistan* or wide or action? or influen*)):ti,ab,kw | 1376 |
| #62 | (teen* NEAR/2 (led or leader* or participat* or mediat* or helper? or resistan* or wide or action? or influen*)):ti,ab,kw | 75 |
| #63 | (adolescent* NEAR/2 (led or leader* or participat* or mediat* or helper? or resistan* or wide or action? or influen*)):ti,ab,kw | 595 |
| #64 | (youth? NEAR/2 (led or leader* or participat* or mediat* or helper? or resistan* or wide or action? or influen*)):ti,ab,kw | 233 |
| #65 | (student? NEAR/2 (led or leader* or participat* or mediat* or helper? or resistan* or wide or action? or influen*)):ti,ab,kw | 1363 |
| #66 | #65 NOT #16 | 1178 |
| #67 | (teach* NEAR/2 (led or leader* or participat* or mediat* or helper? or resistan* or wide or action? or influen* or relationship*)):ti,ab,kw | 464 |
| #68 | (curricul* NEAR/2 (led or leader* or participat* or mediat* or helper? or resistan* or wide or action? or influen* or relationship*)):ti,ab,kw | 78 |
| #69 | (administrat* NEAR/2 (led or leader* or participat* or mediat* or helper? or resistan* or wide or action? or influen* or relationship*)):ti,ab,kw | 2018 |
| #70 | ((personnel or staff) NEAR/2 (led or leader* or participat* or mediat* or helper? or resistan* or wide or action? or influen* or relationship*)):ti,ab,kw | 451 |
| #71 | (volunteer* NEAR/2 (led or leader* or participat* or mediat* or helper? or resistan* or wide or action? or influen* or relationship*)):ti,ab,kw | 2438 |
| #72 | ((advocacy or advocate) NEAR/2 (led or leader* or participat* or mediat* or helper? or resistan* or wide or action? or influen* or relationship*)):ti,ab,kw | 19 |
| #73 | (parent* NEAR/2 (led or leader* or participat* or mediat* or helper? or resistan* or wide or action? or influen* or relationship*)):ti,ab,kw | 1748 |
| #74 | MeSH descriptor: [Social Environment] this term only | 897 |
| #75 | (environment* NEAR/2 (led or leader* or participat* or mediat* or helper? or resistan* or wide or action? or influen* or relationship*)):ti,ab,kw | 551 |
| #76 | (socio-ecolog* NEAR/2 (led or leader* or participat* or mediat* or helper? or resistan* or wide or action? or influen* or relationship*)):ti,ab,kw | 0 |
| #77 | MeSH descriptor: [Social Support] this term only | 3222 |
| #78 | MeSH descriptor: [Social Learning] this term only | 28 |
| #79 | (learn* NEAR/2 (led or leader* or participat* or mediat* or helper? or resistan* or wide or action? or influen* or relationship*)):ti,ab,kw | 505 |
| #80 | ((policy or policies) NEAR/2 (led or leader* or participat* or mediat* or helper? or resistan* or wide or action? or influen* or relationship*)):ti,ab,kw | 145 |
| #81 | #52 or #53 or #54 or #55 or #56 or #57 or #59 or #60 or #61 or #62 or #63 or #64 or #66 or #67 or #68 or #69 or #70 or #71 or #72 or #73 or #74 or #75 or #76 or #77 or #78 or #79 or #80 | 21160 |
| #82 | #51 and #81 | 3270 |
| #83 | #44 or #45 or #46 or #47 or #48 or #82 | 10673 |
| #84 | MeSH descriptor: [Smoking Prevention] this term only | 1584 |
| #85 | MeSH descriptor: [Tobacco Smoking] explode all trees and with qualifier(s): [prevention & control - PC] | 18 |
| #86 | MeSH descriptor: [Vaping] this term only and with qualifier(s): [prevention & control - PC] | 0 |
| #87 | MeSH descriptor: [Marijuana Smoking] this term only and with qualifier(s): [prevention & control - PC] | 67 |
| #88 | MeSH descriptor: [Smoking Cessation] this term only | 3862 |
| #89 | MeSH descriptor: [Tobacco Use Cessation] this term only | 96 |
| #90 | MeSH descriptor: [Smoking Reduction] this term only | 11 |
| #91 | ((smoking or smoked or smoke or smoker) NEAR/5 (reduc* or decreas* or increas* or lower* or fewer OR improv* or enhance* or extend* or develop* or prevalen* or daily or weekly or current or rate or rates or behavio?r* or knowledge or prevent* or attitude* or avoid* or perceive* or percept* or promot* or program* or change*)):ti,ab,kw | 12504 |
| #92 | (tobacco NEAR/5 (reduc* or decreas* or increas* or lower* or fewer OR improv* or enhance* or extend* or develop* or prevalen* or daily or weekly or current or rate or rates or behavio?r* or knowledge or prevent* or attitude* or avoid* or perceive* or percept* or promot* or program* or change*)):ti,ab,kw | 2737 |
| #93 | (cigarette? NEAR/5 (reduc* or decreas* or increas* or lower* or fewer OR improv* or enhance* or extend* or develop* or prevalen* or daily or weekly or current or rate or rates or behavio?r* or knowledge or prevent* or attitude* or avoid* or perceive* or percept* or promot* or program* or change*)):ti,ab,kw | 3051 |
| #94 | (marijuana NEAR/5 (reduc* or decreas* or increas* or lower* or fewer OR improv* or enhance* or extend* or develop* or prevalen* or daily or weekly or current or rate or rates or behavio?r* or knowledge or prevent* or attitude* or avoid* or perceive* or percept* or promot* or program* or change*)):ti,ab,kw | 620 |
| #95 | (cannabis NEAR/5 (reduc* or decreas* or increas* or lower* or fewer OR improv* or enhance* or extend* or develop* or prevalen* or daily or weekly or current or rate or rates or behavio?r* or knowledge or prevent* or attitude* or avoid* or perceive* or percept* or promot* or program* or change*)):ti,ab,kw | 786 |
| #96 | (solvent? NEAR/5 (reduc* or decreas* or increas* or lower* or fewer OR improv* or enhance* or extend* or develop* or prevalen* or daily or weekly or current or rate or rates or behavio?r* or knowledge or prevent* or attitude* or avoid* or perceive* or percept* or promot* or program* or change*)):ti,ab,kw | 148 |
| #97 | MeSH descriptor: [Substance-Related Disorders] explode all trees and with qualifier(s): [prevention & control - PC] | 1520 |
| #98 | MeSH descriptor: [Alcohol Drinking] this term only and with qualifier(s): [prevention & control - PC] | 773 |
| #99 | MeSH descriptor: [Binge Drinking] this term only and with qualifier(s): [prevention & control - PC] | 66 |
| #100 | MeSH descriptor: [Underage Drinking] this term only and with qualifier(s): [prevention & control - PC] | 39 |
| #101 | (alcohol NEAR/5 (reduc* or decreas* or increas* or lower* or fewer OR improv* or enhance* or extend* or develop* or prevalen* or daily or weekly or current or rate or rates or behavio?r* or knowledge or prevent* or attitude* or avoid* or perceive* or percept* or promot* or program* or change*)):ti,ab,kw | 8870 |
| #102 | ((binge NEXT drink*) NEAR/5 (reduc* or decreas* or increas* or lower* or fewer OR improv* or enhance* or extend* or develop* or prevalen* or daily or weekly or current or rate or rates or behavio?r* or knowledge or prevent* or attitude* or avoid* or perceive* or percept* or promot* or program* or change*)):ti,ab,kw | 341 |
| #103 | ((underage NEXT drink*) NEAR/5 (reduc* or decreas* or increas* or lower* or fewer OR improv* or enhance* or extend* or develop* or prevalen* or daily or weekly or current or rate or rates or behavio?r* or knowledge or prevent* or attitude* or avoid* or perceive* or percept* or promot* or program* or change*)):ti,ab,kw | 69 |
| #104 | (substance? NEAR/5 (reduc* or decreas* or increas* or lower* or fewer OR improv* or enhance* or extend* or develop* or prevalen* or daily or weekly or current or rate or rates or behavio?r* or knowledge or prevent* or attitude* or avoid* or perceive* or percept* or promot* or program* or change*)):ti,ab,kw | 4757 |
| #105 | (drug? NEAR/5 (reduc* or decreas* or increas* or lower* or fewer OR improv* or enhance* or extend* or develop* or prevalen* or daily or weekly or current or rate or rates or behavio?r* or knowledge or prevent* or attitude* or avoid* or perceive* or percept* or promot* or program* or change*)):ti,ab,kw | 97713 |
| #106 | MeSH descriptor: [Academic Performance] explode all trees | 42 |
| #107 | ((education* NEXT attain*) NEAR/5 (reduc* or decreas* or increas* or lower* or fewer OR improv* or enhance* or extend* or develop* or prevalen* or daily or weekly or current or rate or rates or behavio?r* or knowledge or prevent* or attitude* or avoid* or perceive* or percept* or promot* or program* or change*)):ti,ab,kw | 133 |
| #108 | ((academic NEXT attain*) NEAR/5 (reduc* or decreas* or increas* or lower* or fewer OR improv* or enhance* or extend* or develop* or prevalen* or daily or weekly or current or rate or rates or behavio?r* or knowledge or prevent* or attitude* or avoid* or perceive* or percept* or promot* or program* or change*)):ti,ab,kw | 4 |
| #109 | ((student* NEXT attain*) NEAR/5 (reduc* or decreas* or increas* or lower* or fewer OR improv* or enhance* or extend* or develop* or prevalen* or daily or weekly or current or rate or rates or behavio?r* or knowledge or prevent* or attitude* or avoid* or perceive* or percept* or promot* or program* or change*)):ti,ab,kw | 3 |
| #110 | ((education* NEXT achieve*) NEAR/5 (reduc* or decreas* or increas* or lower* or fewer OR improv* or enhance* or extend* or develop* or prevalen* or daily or weekly or current or rate or rates or behavio?r* or knowledge or prevent* or attitude* or avoid* or perceive* or percept* or promot* or program* or change*)):ti,ab,kw | 30 |
| #111 | ((academic NEXT achieve*) NEAR/5 (reduc* or decreas* or increas* or lower* or fewer OR improv* or enhance* or extend* or develop* or prevalen* or daily or weekly or current or rate or rates or behavio?r* or knowledge or prevent* or attitude* or avoid* or perceive* or percept* or promot* or program* or change*)):ti,ab,kw | 239 |
| #112 | ((student* NEXT achieve*) NEAR/5 (reduc* or decreas* or increas* or lower* or fewer OR improv* or enhance* or extend* or develop* or prevalen* or daily or weekly or current or rate or rates or behavio?r* or knowledge or prevent* or attitude* or avoid* or perceive* or percept* or promot* or program* or change*)):ti,ab,kw | 16 |
| #113 | ((education* NEXT perform*) NEAR/5 (reduc* or decreas* or increas* or lower* or fewer OR improv* or enhance* or extend* or develop* or prevalen* or daily or weekly or current or rate or rates or behavio?r* or knowledge or prevent* or attitude* or avoid* or perceive* or percept* or promot* or program* or change*)):ti,ab,kw | 21 |
| #114 | ((academic NEXT perform*) NEAR/5 (reduc* or decreas* or increas* or lower* or fewer OR improv* or enhance* or extend* or develop* or prevalen* or daily or weekly or current or rate or rates or behavio?r* or knowledge or prevent* or attitude* or avoid* or perceive* or percept* or promot* or program* or change*)):ti,ab,kw | 277 |
| #115 | ((student* NEXT perform*) NEAR/5 (reduc* or decreas* or increas* or lower* or fewer OR improv* or enhance* or extend* or develop* or prevalen* or daily or weekly or current or rate or rates or behavio?r* or knowledge or prevent* or attitude* or avoid* or perceive* or percept* or promot* or program* or change*)):ti,ab,kw | 123 |
| #116 | ((education* NEXT outcome?) NEAR/5 (reduc* or decreas* or increas* or lower* or fewer OR improv* or enhance* or extend* or develop* or prevalen* or daily or weekly or current or rate or rates or behavio?r* or knowledge or prevent* or attitude* or avoid* or perceive* or percept* or promot* or program* or change*)):ti,ab,kw | 135 |
| #117 | ((academic NEXT outcome?) NEAR/5 (reduc* or decreas* or increas* or lower* or fewer OR improv* or enhance* or extend* or develop* or prevalen* or daily or weekly or current or rate or rates or behavio?r* or knowledge or prevent* or attitude* or avoid* or perceive* or percept* or promot* or program* or change*)):ti,ab,kw | 53 |
| #118 | ((student* NEXT outcome?) NEAR/5 (reduc* or decreas* or increas* or lower* or fewer OR improv* or enhance* or extend* or develop* or prevalen* or daily or weekly or current or rate or rates or behavio?r* or knowledge or prevent* or attitude* or avoid* or perceive* or percept* or promot* or program* or change*)):ti,ab,kw | 40 |
| #119 | ((education* NEXT commitment) NEAR/5 (reduc* or decreas* or increas* or lower* or fewer OR improv* or enhance* or extend* or develop* or prevalen* or daily or weekly or current or rate or rates or behavio?r* or knowledge or prevent* or attitude* or avoid* or perceive* or percept* or promot* or program* or change*)):ti,ab,kw | 1 |
| #120 | ((academic NEXT commitment) NEAR/5 (reduc* or decreas* or increas* or lower* or fewer OR improv* or enhance* or extend* or develop* or prevalen* or daily or weekly or current or rate or rates or behavio?r* or knowledge or prevent* or attitude* or avoid* or perceive* or percept* or promot* or program* or change*)):ti,ab,kw | 0 |
| #121 | ((student* NEXT commitment) NEAR/5 (reduc* or decreas* or increas* or lower* or fewer OR improv* or enhance* or extend* or develop* or prevalen* or daily or weekly or current or rate or rates or behavio?r* or knowledge or prevent* or attitude* or avoid* or perceive* or percept* or promot* or program* or change*)):ti,ab,kw | 0 |
| #122 | ((education* NEXT engage*) NEAR/5 (reduc* or decreas* or increas* or lower* or fewer OR improv* or enhance* or extend* or develop* or prevalen* or daily or weekly or current or rate or rates or behavio?r* or knowledge or prevent* or attitude* or avoid* or perceive* or percept* or promot* or program* or change*)):ti,ab,kw | 3 |
| #123 | ((academic NEXT engage*) NEAR/5 (reduc* or decreas* or increas* or lower* or fewer OR improv* or enhance* or extend* or develop* or prevalen* or daily or weekly or current or rate or rates or behavio?r* or knowledge or prevent* or attitude* or avoid* or perceive* or percept* or promot* or program* or change*)):ti,ab,kw | 14 |
| #124 | ((student* NEXT engage*) NEAR/5 (reduc* or decreas* or increas* or lower* or fewer OR improv* or enhance* or extend* or develop* or prevalen* or daily or weekly or current or rate or rates or behavio?r* or knowledge or prevent* or attitude* or avoid* or perceive* or percept* or promot* or program* or change*)):ti,ab,kw | 31 |
| #125 | (dropout NEAR/5 (reduc* or decreas* or increas* or lower* or fewer OR improv* or enhance* or extend* or develop* or prevalen* or daily or weekly or current or rate or rates or behavio?r* or knowledge or prevent* or attitude* or avoid* or perceive* or percept* or promot* or program* or change*)):ti,ab,kw | 3341 |
| #126 | (absentee* NEAR/5 (reduc* or decreas* or increas* or lower* or fewer OR improv* or enhance* or extend* or develop* or prevalen* or daily or weekly or current or rate or rates or behavio?r* or knowledge or prevent* or attitude* or avoid* or perceive* or percept* or promot* or program* or change*)):ti,ab,kw | 435 |
| #127 | MeSH descriptor: [Violence] this term only and with qualifier(s): [prevention & control - PC] | 215 |
| #128 | MeSH descriptor: [Aggression] this term only | 1144 |
| #129 | MeSH descriptor: [Physical Abuse] this term only and with qualifier(s): [prevention & control - PC] | 10 |
| #130 | MeSH descriptor: [Gender-Based Violence] this term only and with qualifier(s): [prevention & control - PC] | 3 |
| #131 | MeSH descriptor: [Gun Violence] this term only and with qualifier(s): [prevention & control - PC] | 0 |
| #132 | MeSH descriptor: [Bullying] explode all trees and with qualifier(s): [prevention & control - PC] | 36 |
| #133 | ((violent or violence) NEAR/5 (reduc* or decreas* or increas* or lower* or fewer OR improv* or enhance* or extend* or develop* or prevalen* or daily or weekly or current or rate or rates or behavio?r* or knowledge or prevent* or attitude* or avoid* or perceive* or percept* or promot* or program* or change*)):ti,ab,kw | 1538 |
| #134 | ((aggression or aggressive) NEAR/5 (reduc* or decreas* or increas* or lower* or fewer OR improv* or enhance* or extend* or develop* or prevalen* or daily or weekly or current or rate or rates or behavio?r* or knowledge or prevent* or attitude* or avoid* or perceive* or percept* or promot* or program* or change*)):ti,ab,kw | 3315 |
| #135 | ((hostile or hostility) NEAR/5 (reduc* or decreas* or increas* or lower* or fewer OR improv* or enhance* or extend* or develop* or prevalen* or daily or weekly or current or rate or rates or behavio?r* or knowledge or prevent* or attitude* or avoid* or perceive* or percept* or promot* or program* or change*)):ti,ab,kw | 427 |
| #136 | (assault* NEAR/5 (reduc* or decreas* or increas* or lower* or fewer OR improv* or enhance* or extend* or develop* or prevalen* or daily or weekly or current or rate or rates or behavio?r* or knowledge or prevent* or attitude* or avoid* or perceive* or percept* or promot* or program* or change*)):ti,ab,kw | 196 |
| #137 | (crime NEAR/5 (reduc* or decreas* or increas* or lower* or fewer OR improv* or enhance* or extend* or develop* or prevalen* or daily or weekly or current or rate or rates or behavio?r* or knowledge or prevent* or attitude* or avoid* or perceive* or percept* or promot* or program* or change*)):ti,ab,kw | 231 |
| #138 | ((abuse? or abusive) NEAR/5 (reduc* or decreas* or increas* or lower* or fewer OR improv* or enhance* or extend* or develop* or prevalen* or daily or weekly or current or rate or rates or behavio?r* or knowledge or prevent* or attitude* or avoid* or perceive* or percept* or promot* or program* or change*)):ti,ab,kw | 3122 |
| #139 | (misbehav* NEAR/5 (reduc* or decreas* or increas* or lower* or fewer OR improv* or enhance* or extend* or develop* or prevalen* or daily or weekly or current or rate or rates or behavio?r* or knowledge or prevent* or attitude* or avoid* or perceive* or percept* or promot* or program* or change*)):ti,ab,kw | 19 |
| #140 | (threat? NEAR/5 (reduc* or decreas* or increas* or lower* or fewer OR improv* or enhance* or extend* or develop* or prevalen* or daily or weekly or current or rate or rates or behavio?r* or knowledge or prevent* or attitude* or avoid* or perceive* or percept* or promot* or program* or change*)):ti,ab,kw | 778 |
| #141 | (conduct NEAR/5 (reduc* or decreas* or increas* or lower* or fewer OR improv* or enhance* or extend* or develop* or prevalen* or daily or weekly or current or rate or rates or behavio?r* or knowledge or prevent* or attitude* or avoid* or perceive* or percept* or promot* or program* or change*)):ti,ab,kw | 1429 |
| #142 | (delinquen* NEAR/5 (reduc* or decreas* or increas* or lower* or fewer OR improv* or enhance* or extend* or develop* or prevalen* or daily or weekly or current or rate or rates or behavio?r* or knowledge or prevent* or attitude* or avoid* or perceive* or percept* or promot* or program* or change*)):ti,ab,kw | 354 |
| #143 | ((disrupt* NEXT behavio?r*) NEAR/5 (reduc* or decreas* or increas* or lower* or fewer OR improv* or enhance* or extend* or develop* or prevalen* or daily or weekly or current or rate or rates or behavio?r* or knowledge or prevent* or attitude* or avoid* or perceive* or percept* or promot* or program* or change*)):ti,ab,kw | 835 |
| #144 | ((problem* NEXT behavio?r*) NEAR/5 (reduc* or decreas* or increas* or lower* or fewer OR improv* or enhance* or extend* or develop* or prevalen* or daily or weekly or current or rate or rates or behavio?r* or knowledge or prevent* or attitude* or avoid* or perceive* or percept* or promot* or program* or change*)):ti,ab,kw | 1217 |
| #145 | ((volatile NEXT behavio?r*) NEAR/5 (reduc* or decreas* or increas* or lower* or fewer OR improv* or enhance* or extend* or develop* or prevalen* or daily or weekly or current or rate or rates or behavio?r* or knowledge or prevent* or attitude* or avoid* or perceive* or percept* or promot* or program* or change*)):ti,ab,kw | 2 |
| #146 | ((antisocial or anti-social) NEAR/5 (reduc* or decreas* or increas* or lower* or fewer OR improv* or enhance* or extend* or develop* or prevalen* or daily or weekly or current or rate or rates or behavio?r* or knowledge or prevent* or attitude* or avoid* or perceive* or percept* or promot* or program* or change*)):ti,ab,kw | 443 |
| #147 | (perpetrat* NEAR/5 (reduc* or decreas* or increas* or lower* or fewer OR improv* or enhance* or extend* or develop* or prevalen* or daily or weekly or current or rate or rates or behavio?r* or knowledge or prevent* or attitude* or avoid* or perceive* or percept* or promot* or program* or change*)):ti,ab,kw | 151 |
| #148 | (bully* NEAR/5 (reduc* or decreas* or increas* or lower* or fewer OR improv* or enhance* or extend* or develop* or prevalen* or daily or weekly or current or rate or rates or behavio?r* or knowledge or prevent* or attitude* or avoid* or perceive* or percept* or promot* or program* or change*)):ti,ab,kw | 181 |
| #149 | (victimi* NEAR/5 (reduc* or decreas* or increas* or lower* or fewer OR improv* or enhance* or extend* or develop* or prevalen* or daily or weekly or current or rate or rates or behavio?r* or knowledge or prevent* or attitude* or avoid* or perceive* or percept* or promot* or program* or change*)):ti,ab,kw | 223 |
| #150 | (cyberbully* NEAR/5 (reduc* or decreas* or increas* or lower* or fewer OR improv* or enhance* or extend* or develop* or prevalen* or daily or weekly or current or rate or rates or behavio?r* or knowledge or prevent* or attitude* or avoid* or perceive* or percept* or promot* or program* or change*)):ti,ab,kw | 26 |
| #151 | (conflict* NEAR/5 (reduc* or decreas* or increas* or lower* or fewer OR improv* or enhance* or extend* or develop* or prevalen* or daily or weekly or current or rate or rates or behavio?r* or knowledge or prevent* or attitude* or avoid* or perceive* or percept* or promot* or program* or change*)):ti,ab,kw | 1594 |
| #152 | ((positive NEXT behavio?r*) NEAR/5 (reduc* or decreas* or increas* or lower* or fewer OR improv* or enhance* or extend* or develop* or prevalen* or daily or weekly or current or rate or rates or behavio?r* or knowledge or prevent* or attitude* or avoid* or perceive* or percept* or promot* or program* or change*)):ti,ab,kw | 309 |
| #153 | ((improv* NEXT behavio?r*) NEAR/5 (reduc* or decreas* or increas* or lower* or fewer OR improv* or enhance* or extend* or develop* or prevalen* or daily or weekly or current or rate or rates or behavio?r* or knowledge or prevent* or attitude* or avoid* or perceive* or percept* or promot* or program* or change*)):ti,ab,kw | 412 |
| #154 | ((social NEXT behavio?r*) NEAR/5 (reduc* or decreas* or increas* or lower* or fewer OR improv* or enhance* or extend* or develop* or prevalen* or daily or weekly or current or rate or rates or behavio?r* or knowledge or prevent* or attitude* or avoid* or perceive* or percept* or promot* or program* or change*)):ti,ab,kw | 3180 |
| #155 | ((social-emotion* or socio-emotion*) NEAR/5 (reduc* or decreas* or increas* or lower* or fewer OR improv* or enhance* or extend* or develop* or prevalen* or daily or weekly or current or rate or rates or behavio?r* or knowledge or prevent* or attitude* or avoid* or perceive* or percept* or promot* or program* or change*)):ti,ab,kw | 404 |
| #156 | ((social-charact*) NEAR/5 (reduc* or decreas* or increas* or lower* or fewer OR improv* or enhance* or extend* or develop* or prevalen* or daily or weekly or current or rate or rates or behavio?r* or knowledge or prevent* or attitude* or avoid* or perceive* or percept* or promot* or program* or change*)):ti,ab,kw | 8 |
| #157 | (social-inclusion NEAR/5 (reduc* or decreas* or increas* or lower* or fewer OR improv* or enhance* or extend* or develop* or prevalen* or daily or weekly or current or rate or rates or behavio?r* or knowledge or prevent* or attitude* or avoid* or perceive* or percept* or promot* or program* or change*)):ti,ab,kw | 25 |
| #158 | ((social NEXT develop*) NEAR/5 (reduc* or decreas* or increas* or lower* or fewer OR improv* or enhance* or extend* or develop* or prevalen* or daily or weekly or current or rate or rates or behavio?r* or knowledge or prevent* or attitude* or avoid* or perceive* or percept* or promot* or program* or change*)):ti,ab,kw | 175 |
| #159 | ((social NEXT competen*) NEAR/5 (reduc* or decreas* or increas* or lower* or fewer OR improv* or enhance* or extend* or develop* or prevalen* or daily or weekly or current or rate or rates or behavio?r* or knowledge or prevent* or attitude* or avoid* or perceive* or percept* or promot* or program* or change*)):ti,ab,kw | 242 |
| #160 | ((emotion* NEXT develop*) NEAR/5 (reduc* or decreas* or increas* or lower* or fewer OR improv* or enhance* or extend* or develop* or prevalen* or daily or weekly or current or rate or rates or behavio?r* or knowledge or prevent* or attitude* or avoid* or perceive* or percept* or promot* or program* or change*)):ti,ab,kw | 180 |
| #161 | (conduct NEAR/5 (reduc* or decreas* or increas* or lower* or fewer OR improv* or enhance* or extend* or develop* or prevalen* or daily or weekly or current or rate or rates or behavio?r* or knowledge or prevent* or attitude* or avoid* or perceive* or percept* or promot* or program* or change*)):ti,ab,kw | 1429 |
| #162 | (respect NEAR/5 (reduc* or decreas* or increas* or lower* or fewer OR improv* or enhance* or extend* or develop* or prevalen* or daily or weekly or current or rate or rates or behavio?r* or knowledge or prevent* or attitude* or avoid* or perceive* or percept* or promot* or program* or change*)):ti,ab,kw | 4428 |
| #163 | ((safety or safe) NEAR/5 (reduc* or decreas* or increas* or lower* or fewer OR improv* or enhance* or extend* or develop* or prevalen* or daily or weekly or current or rate or rates or behavio?r* or knowledge or prevent* or attitude* or avoid* or perceive* or percept* or promot* or program* or change*)):ti,ab,kw | 33637 |
| #164 | #84 or #85 or #86 or #87 or #88 or #89 or #90 or #91 or #92 or #93 or #94 or #95 or #96 or #97 or #99 or #100 or #101 or #102 or #103 or #104 or #105 | 121646 |
| #165 | #106 or #107 or #108 or #109 or #110 or #111 or #112 or #113 or #114 or #115 or #116 or #117 or #118 or #119 or #120 or #121 or #122 or #123 or #124 or #125 or #126 | 4840 |
| #166 | #127 or #128 or #129 or #130 or #131 or #132 or #133 or #134 or #135 or #136 or #137 or #138 or #139 or #140 or #141 or #142 or #143 or #144 or #145 or #146 or #147 or #148 or #149 or #150 or #151 or #152 or #153 or #154 or #155 or #156 or #157 or #158 or #159 or #160 or #161 or #162 or #163 | 55061 |
| #167 | MeSH descriptor: [Program Evaluation] explode all trees | 5973 |
| #168 | MeSH descriptor: [Evaluation Studies] this term only | 1 |
| #169 | MeSH descriptor: [Validation Studies] this term only | 0 |
| #170 | MeSH descriptor: [Clinical Trial] explode all trees | 147 |
| #171 | (prevent*):ti,ab,kw | 215586 |
| #172 | (program*):ti,ab,kw | 112268 |
| #173 | (intervent*):ti,ab,kw | 363661 |
| #174 | (restorative NEXT approach*):ti,ab,kw | 24 |
| #175 | (control NEXT school?):ti,ab,kw | 718 |
| #176 | (trial or trials):ti,ab,kw | 836454 |
| #177 | #167 or #168 or #169 or #170 or #171 or #172 or #173 or #174 or #175 or #176 | 1063096 |
| #178 | #43 and #83 and #164 and #177 | 1713 |
| #179 | #43 and #83 and #165 and #177 | 361 |
| #180 | #43 and #83 and #166 and #177 | 1436 |

## EPPI-Centre database of health promotion research (Bibliomap)

| Database name | Database of health promotion research (Bibliomap) |
| --- | --- |
| Database platform | EPPI-Centre |
| Dates of database coverage | Complete database to date searched. |
| Date searched | 23/01/2020 |
| Searched by | JF |
| Number of results | 0 |
| EndNote import order | N/A |
| Number of results once duplicates removed | 0 |
| Search strategy notes | None |

Freetext: “school OR schools OR schoolwide”

## EPPI-Centre Database of promoting health effectiveness reviews (DoPHER)

| Database name | Database of promoting health effectiveness reviews (DoPHER) |
| --- | --- |
| Database platform | EPPI-Centre |
| Dates of database coverage | Complete database to date searched. |
| Date searched | 23/01/2020 |
| Searched by | JF |
| Number of results | 0 |
| EndNote import order | N/A |
| Number of results once duplicates removed | 0 |
| Search strategy notes | None |

Freetext: “school OR schools OR schoolwide”

## OvidSP Econlit

| Database name | Econlit |
| --- | --- |
| Database platform | OvidSP |
| Dates of database coverage | 1886 to January 09 2020 |
| Date searched | 16/01/2020 |
| Searched by | JF |
| Number of results | 223 |
| EndNote import order | 4 |
| Number of results once duplicates removed | 208 |
| Search strategy notes | Search lines ending in .ti,ab,kw. search in the title, abstract and author keywords only. Search lines ending in .jx. search in the journal title only. or/*x-y* combines search sets in the range *x-y* with Boolean operator OR. * is used for truncation of words. # is used for a compulsory wildcard. ? is used for an optional wildcard. adj*n* searches for terms within *n* words or each other. Words in square brackets [ ] are comments and are not included in the search terms. |

1. child*.ti,ab,kw. (27019)
2. boy?.ti,ab,kw. (1760)
3. girl?.ti,ab,kw. (2252)
4. (schoolage or (school adj1 age)).ti,ab,kw. (352)
5. schoolchild*.ti,ab,kw. (83)
6. youngster?.ti,ab,kw. (95)
7. minor?.ti,ab,kw. (3158)
8. ((pupil* or student*) not ((pupil adj5 eye?) or (student* adj medic*))).ti,ab,kw. (20959)
9. prepubescen*.ti,ab,kw. (3)
10. pubescent*.ti,ab,kw. (3)
11. adolescent*.ti,ab,kw. (1916)
12. juvenil*.ti,ab,kw. (389)
13. underage*.ti,ab,kw. (72)
14. (preteen* or pre-teen*).ti,ab,kw. (12)
15. (teen or teens).ti,ab,kw. (577)
16. teenage*.ti,ab,kw. (1033)
17. (youth or youths).ti,ab,kw. (5840)
18. young person*.ti,ab,kw. (94)
19. young people*.ti,ab,kw. (1841)
20. (kid or kids).ti,ab,kw. (331)
21. (transition adj4 adult*).ti,ab,kw. (157)
22. emerging adult*.ti,ab,kw. (18)
23. young adult.ti,ab,kw. (327)
24. parent?.ti,ab,kw. (9273)
25. mother?.ti,ab,kw. (5191)
26. father?.ti,ab,kw. (2260)
27. guardian?.ti,ab,kw. (192)
28. teacher?.ti,ab,kw. (4462)
29. child*.jx. (0)
30. adolescen*.jx. (0)
31. youth*.jx. (0)
32. school*.jx. (2343)
33. (("5" or "6" or "7" or "8" or "9" or "10" or "11" or "12" or "13" or "14" or "15" or "16" or "17" or "18") adj (year* old or year* of age)).ti,ab,kw. (563)
34. ((five or six or seven or eight or nine or ten or eleven or twelve or thirteen or fourteen or fifteen or sixteen or seventeen or eighteen) adj (year* old or year* of age)).ti,ab,kw. (212)
35. (age* adj ("5" or "6" or "7" or "8" or "9" or "10" or "11" or "12" or "13" or "14" or "15" or "16" or "17" or "18") adj year*).ti,ab,kw. (66)
36. (age* adj (five or six or seven or eight or nine or ten or eleven or twelve or thirteen or fourteen or fifteen or sixteen or seventeen or eighteen) adj year*).ti,ab,kw. (16)
37. ((primary or secondary or year) adj1 ("1" or "2" or "3" or "4" or "5" or "6" or "7" or "8" or "9" or "10" or "11" or "12" or "13")).ti,ab,kw. (3499)
38. (grade? adj1 (first or second or third or fourth or fifth or sixth or seventh or eighth or ninth or tenth or eleventh or twelfth)).ti,ab,kw. (444)
39. or/1-38 (69786)
40. (school? adj5 (based or level or wide or led or ethos or environment or organi?ation* or climate or toolkit? or approach* or action group? or practice? or whole or health* or leader* or cultur* or manag* or governance or system? or communit*)).ti,ab. (5710)
41. schoolwide.ti,ab. (6)
42. restor* justice.ti,ab. (35)
43. school?.ti,ab,jx. (33502)
44. "peer-to-peer".ti,ab. (336)
45. (peer adj2 (led or leader* or participat* or mediat* or helper? or resistan* or wide or action? or influen*)).ti,ab. (220)
46. (relationship* adj2 (led or leader* or participat* or mediat* or helper? or resistan* or wide or action? or influen*)).ti,ab. (1184)
47. ((community or communities) adj2 (led or leader* or participat* or mediat* or helper? or resistan* or wide or action? or influen* or relationship*)).ti,ab. (1517)
48. (pupil? adj2 (led or leader* or participat* or mediat* or helper? or resistan* or wide or action? or influen* relationship*)).ti,ab. (4)
49. ((child or children) adj2 (led or leader* or participat* or mediat* or helper? or resistan* or wide or action? or influen*)).ti,ab. (418)
50. ((young people or young person) adj2 (led or leader* or participat* or mediat* or helper? or resistan* or wide or action? or influen*)).ti,ab. (33)
51. (teen* adj2 (led or leader* or participat* or mediat* or helper? or resistan* or wide or action? or influen*)).ti,ab. (31)
52. (adolescent* adj2 (led or leader* or participat* or mediat* or helper? or resistan* or wide or action? or influen*)).ti,ab. (66)
53. (youth? adj2 (led or leader* or participat* or mediat* or helper? or resistan* or wide or action? or influen*)).ti,ab. (137)
54. (student? adj2 (led or leader* or participat* or mediat* or helper? or resistan* or wide or action? or influen*)).ti,ab. (519)
55. (teach* adj2 (led or leader* or participat* or mediat* or helper? or resistan* or wide or action? or influen* or relationship*)).ti,ab. (259)
56. (curricul* adj2 (led or leader* or participat* or mediat* or helper? or resistan* or wide or action? or influen* or relationship*)).ti,ab. (22)
57. (administrat* adj2 (led or leader* or participat* or mediat* or helper? or resistan* or wide or action? or influen* or relationship*)).ti,ab. (300)
58. ((personnel or staff) adj2 (led or leader* or participat* or mediat* or helper? or resistan* or wide or action? or influen* or relationship*)).ti,ab. (115)
59. (volunteer* adj2 (led or leader* or participat* or mediat* or helper? or resistan* or wide or action? or influen* or relationship*)).ti,ab. (140)
60. ((advocacy or advocate) adj2 (led or leader* or participat* or mediat* or helper? or resistan* or wide or action? or influen* or relationship*)).ti,ab. (54)
61. (parent* adj2 (led or leader* or participat* or mediat* or helper? or resistan* or wide or action? or influen*)).ti,ab. (389)
62. (environment* adj2 (led or leader* or participat* or mediat* or helper? or resistan* or wide or action? or influen* or relationship*)).ti,ab. (1665)
63. (socio-ecolog* adj2 (led or leader* or participat* or mediat* or helper? or resistan* or wide or action? or influen* or relationship*)).ti,ab. (0)
64. (learn* adj2 (led or leader* or participat* or mediat* or helper? or resistan* or wide or action? or influen* or relationship*)).ti,ab. (496)
65. ((policy or policies) adj2 (led or leader* or participat* or mediat* or helper? or resistan* or wide or action? or influen* or relationship*)).ti,ab. (4838)
66. or/44-65 (12292)
67. 43 and 66 (1108)
68. or/40-42,67 (6554)
69. ((smoking or smoked or smoke or smoker) adj5 (reduc* or decreas* or increas* or lower* or fewer or improv* or enhance* or extend* or develop* or prevalen* or daily or weekly or current or rate or rates or behavio?r* or knowledge or prevent* or attitude* or avoid* or perceive* or percept* or promot* or program* or change*)).ti,ab. (1062)
70. (tobacco adj5 (reduc* or decreas* or increas* or lower* or fewer or improv* or enhance* or extend* or develop* or prevalen* or daily or weekly or current or rate or rates or behavio?r* or knowledge or prevent* or attitude* or avoid* or perceive* or percept* or promot* or program* or change*)).ti,ab. (413)
71. (cigarette? adj5 (reduc* or decreas* or increas* or lower* or fewer or improv* or enhance* or extend* or develop* or prevalen* or daily or weekly or current or rate or rates or behavio?r* or knowledge or prevent* or attitude* or avoid* or perceive* or percept* or promot* or program* or change*)).ti,ab. (489)
72. (marijuana adj5 (reduc* or decreas* or increas* or lower* or fewer or improv* or enhance* or extend* or develop* or prevalen* or daily or weekly or current or rate or rates or behavio?r* or knowledge or prevent* or attitude* or avoid* or perceive* or percept* or promot* or program* or change*)).ti,ab. (95)
73. (cannabis adj5 (reduc* or decreas* or increas* or lower* or fewer or improv* or enhance* or extend* or develop* or prevalen* or daily or weekly or current or rate or rates or behavio?r* or knowledge or prevent* or attitude* or avoid* or perceive* or percept* or promot* or program* or change*)).ti,ab. (48)
74. (solvent? adj5 (reduc* or decreas* or increas* or lower* or fewer or improv* or enhance* or extend* or develop* or prevalen* or daily or weekly or current or rate or rates or behavio?r* or knowledge or prevent* or attitude* or avoid* or perceive* or percept* or promot* or program* or change*)).ti,ab. (31)
75. (alcohol adj5 (reduc* or decreas* or increas* or lower* or fewer or improv* or enhance* or extend* or develop* or prevalen* or daily or weekly or current or rate or rates or behavio?r* or knowledge or prevent* or attitude* or avoid* or perceive* or percept* or promot* or program* or change*)).ti,ab. (599)
76. (binge drink* adj5 (reduc* or decreas* or increas* or lower* or fewer or improv* or enhance* or extend* or develop* or prevalen* or daily or weekly or current or rate or rates or behavio?r* or knowledge or prevent* or attitude* or avoid* or perceive* or percept* or promot* or program* or change*)).ti,ab. (48)
77. (underage drink* adj5 (reduc* or decreas* or increas* or lower* or fewer or improv* or enhance* or extend* or develop* or prevalen* or daily or weekly or current or rate or rates or behavio?r* or knowledge or prevent* or attitude* or avoid* or perceive* or percept* or promot* or program* or change*)).ti,ab. (9)
78. (substance? adj5 (reduc* or decreas* or increas* or lower* or fewer or improv* or enhance* or extend* or develop* or prevalen* or daily or weekly or current or rate or rates or behavio?r* or knowledge or prevent* or attitude* or
79. avoid* or perceive* or percept* or promot* or program* or change*)).ti,ab. (267)
80. (drug? adj5 (reduc* or decreas* or increas* or lower* or fewer or improv* or enhance* or extend* or develop* or prevalen* or daily or weekly or current or rate or rates or behavio?r* or knowledge or prevent* or attitude* or avoid* or perceive* or percept* or promot* or program* or change*)).ti,ab. (1603)
81. (education* attain* adj5 (reduc* or decreas* or increas* or lower* or fewer or improv* or enhance* or extend* or develop* or prevalen* or daily or weekly or current or rate or rates or behavio?r* or knowledge or prevent* or attitude* or avoid* or perceive* or percept* or promot* or program* or change*)).ti,ab. (1092)
82. (academic attain* adj5 (reduc* or decreas* or increas* or lower* or fewer or improv* or enhance* or extend* or develop* or prevalen* or daily or weekly or current or rate or rates or behavio?r* or knowledge or prevent* or attitude* or avoid* or perceive* or percept* or promot* or program* or change*)).ti,ab. (5)
83. (student* attain* adj5 (reduc* or decreas* or increas* or lower* or fewer or improv* or enhance* or extend* or develop* or prevalen* or daily or weekly or current or rate or rates or behavio?r* or knowledge or prevent* or attitude* or avoid* or perceive* or percept* or promot* or program* or change*)).ti,ab. (9)
84. (education* achieve* adj5 (reduc* or decreas* or increas* or lower* or fewer or improv* or enhance* or extend* or develop* or prevalen* or daily or weekly or current or rate or rates or behavio?r* or knowledge or prevent* or attitude* or avoid* or perceive* or percept* or promot* or program* or change*)).ti,ab. (95)
85. (academic achieve* adj5 (reduc* or decreas* or increas* or lower* or fewer or improv* or enhance* or extend* or develop* or prevalen* or daily or weekly or current or rate or rates or behavio?r* or knowledge or prevent* or attitude* or avoid* or perceive* or percept* or promot* or program* or change*)).ti,ab. (138)
86. (student* achieve* adj5 (reduc* or decreas* or increas* or lower* or fewer or improv* or enhance* or extend* or develop* or prevalen* or daily or weekly or current or rate or rates or behavio?r* or knowledge or prevent* or attitude* or avoid* or perceive* or percept* or promot* or program* or change*)).ti,ab. (277)
87. (education* perform* adj5 (reduc* or decreas* or increas* or lower* or fewer or improv* or enhance* or extend* or develop* or prevalen* or daily or weekly or current or rate or rates or behavio?r* or knowledge or prevent* or attitude* or avoid* or perceive* or percept* or promot* or program* or change*)).ti,ab. (49)
88. (academic perform* adj5 (reduc* or decreas* or increas* or lower* or fewer or improv* or enhance* or extend* or develop* or prevalen* or daily or weekly or current or rate or rates or behavio?r* or knowledge or prevent* or attitude* or avoid* or perceive* or percept* or promot* or program* or change*)).ti,ab. (170)
89. (student* perform* adj5 (reduc* or decreas* or increas* or lower* or fewer or improv* or enhance* or extend* or develop* or prevalen* or daily or weekly or current or rate or rates or behavio?r* or knowledge or prevent* or attitude* or avoid* or perceive* or percept* or promot* or program* or change*)).ti,ab. (200)
90. (education* outcome? adj5 (reduc* or decreas* or increas* or lower* or fewer or improv* or enhance* or extend* or develop* or prevalen* or daily or weekly or current or rate or rates or behavio?r* or knowledge or prevent* or attitude* or avoid* or perceive* or percept* or promot* or program* or change*)).ti,ab. (319)
91. (academic outcome? adj5 (reduc* or decreas* or increas* or lower* or fewer or improv* or enhance* or extend* or develop* or prevalen* or daily or weekly or current or rate or rates or behavio?r* or knowledge or prevent* or attitude* or avoid* or perceive* or percept* or promot* or program* or change*)).ti,ab. (48)
92. (student* outcome? adj5 (reduc* or decreas* or increas* or lower* or fewer or improv* or enhance* or extend* or develop* or prevalen* or daily or weekly or current or rate or rates or behavio?r* or knowledge or prevent* or attitude* or avoid* or perceive* or percept* or promot* or program* or change*)).ti,ab. (99)
93. (education* commitment adj5 (reduc* or decreas* or increas* or lower* or fewer or improv* or enhance* or extend* or develop* or prevalen* or daily or weekly or current or rate or rates or behavio?r* or knowledge or prevent* or attitude* or avoid* or perceive* or percept* or promot* or program* or change*)).ti,ab. (0)
94. (academic commitment adj5 (reduc* or decreas* or increas* or lower* or fewer or improv* or enhance* or extend* or develop* or prevalen* or daily or weekly or current or rate or rates or behavio?r* or knowledge or prevent* or attitude* or avoid* or perceive* or percept* or promot* or program* or change*)).ti,ab. (2)
95. (student* commitment adj5 (reduc* or decreas* or increas* or lower* or fewer or improv* or enhance* or extend* or develop* or prevalen* or daily or weekly or current or rate or rates or behavio?r* or knowledge or prevent* or attitude* or avoid* or perceive* or percept* or promot* or program* or change*)).ti,ab. (2)
96. (education* engage* adj5 (reduc* or decreas* or increas* or lower* or fewer or improv* or enhance* or extend* or develop* or prevalen* or daily or weekly or current or rate or rates or behavio?r* or knowledge or prevent* or attitude* or avoid* or perceive* or percept* or promot* or program* or change*)).ti,ab. (1)
97. (academic engage* adj5 (reduc* or decreas* or increas* or lower* or fewer or improv* or enhance* or extend* or develop* or prevalen* or daily or weekly or current or rate or rates or behavio?r* or knowledge or prevent* or attitude* or avoid* or perceive* or percept* or promot* or program* or change*)).ti,ab. (2)
98. (student* engage* adj5 (reduc* or decreas* or increas* or lower* or fewer or improv* or enhance* or extend* or develop* or prevalen* or daily or weekly or current or rate or rates or behavio?r* or knowledge or prevent* or attitude* or avoid* or perceive* or percept* or promot* or program* or change*)).ti,ab. (20)
99. (dropout adj5 (reduc* or decreas* or increas* or lower* or fewer or improv* or enhance* or extend* or develop* or prevalen* or daily or weekly or current or rate or rates or behavio?r* or knowledge or prevent* or attitude* or avoid* or perceive* or percept* or promot* or program* or change*)).ti,ab. (413)
100. (absentee* adj5 (reduc* or decreas* or increas* or lower* or fewer or improv* or enhance* or extend* or develop* or prevalen* or daily or weekly or current or rate or rates or behavio?r* or knowledge or prevent* or attitude* or avoid* or perceive* or percept* or promot* or program* or change*)).ti,ab. (233)
101. ((violent or violence) adj5 (reduc* or decreas* or increas* or lower* or fewer or improv* or enhance* or extend* or develop* or prevalen* or daily or weekly or current or rate or rates or behavio?r* or knowledge or prevent* or attitude* or avoid* or perceive* or percept* or promot* or program* or change*)).ti,ab. (1477)
102. ((aggression or aggressive) adj5 (reduc* or decreas* or increas* or lower* or fewer or improv* or enhance* or extend* or develop* or prevalen* or daily or weekly or current or rate or rates or behavio?r* or knowledge or prevent* or attitude* or avoid* or perceive* or percept* or promot* or program* or change*)).ti,ab. (633)
103. ((hostile or hostility) adj5 (reduc* or decreas* or increas* or lower* or fewer or improv* or enhance* or extend* or develop* or prevalen* or daily or weekly or current or rate or rates or behavio?r* or knowledge or prevent* or attitude* or avoid* or perceive* or percept* or promot* or program* or change*)).ti,ab. (190)
104. (assault* adj5 (reduc* or decreas* or increas* or lower* or fewer or improv* or enhance* or extend* or develop* or prevalen* or daily or weekly or current or rate or rates or behavio?r* or knowledge or prevent* or attitude* or avoid* or perceive* or percept* or promot* or program* or change*)).ti,ab. (81)
105. (crime adj5 (reduc* or decreas* or increas* or lower* or fewer or improv* or enhance* or extend* or develop* or prevalen* or daily or weekly or current or rate or rates or behavio?r* or knowledge or prevent* or attitude* or avoid* or perceive* or percept* or promot* or program* or change*)).ti,ab. (2007)
106. ((abuse? or abusive) adj5 (reduc* or decreas* or increas* or lower* or fewer or improv* or enhance* or extend* or develop* or prevalen* or daily or weekly or current or rate or rates or behavio?r* or knowledge or prevent* or attitude* or avoid* or perceive* or percept* or promot* or program* or change*)).ti,ab. (549)
107. (misbehav* adj5 (reduc* or decreas* or increas* or lower* or fewer or improv* or enhance* or extend* or develop* or prevalen* or daily or weekly or current or rate or rates or behavio?r* or knowledge or prevent* or attitude* or avoid* or perceive* or percept* or promot* or program* or change*)).ti,ab. (43)
108. (threat? adj5 (reduc* or decreas* or increas* or lower* or fewer or improv* or enhance* or extend* or develop* or prevalen* or daily or weekly or current or rate or rates or behavio?r* or knowledge or prevent* or attitude* or avoid* or perceive* or percept* or promot* or program* or change*)).ti,ab. (1539)
109. (conduct adj5 (reduc* or decreas* or increas* or lower* or fewer or improv* or enhance* or extend* or develop* or prevalen* or daily or weekly or current or rate or rates or behavio?r* or knowledge or prevent* or attitude* or avoid* or perceive* or percept* or promot* or program* or change*)).ti,ab. (990)
110. (delinquen* adj5 (reduc* or decreas* or increas* or lower* or fewer or improv* or enhance* or extend* or develop* or prevalen* or daily or weekly or current or rate or rates or behavio?r* or knowledge or prevent* or attitude* or avoid* or perceive* or percept* or promot* or program* or change*)).ti,ab. (360)
111. (disrupt* behavio?r* adj5 (reduc* or decreas* or increas* or lower* or fewer or improv* or enhance* or extend* or develop* or prevalen* or daily or weekly or current or rate or rates or behavio?r* or knowledge or prevent* or attitude* or avoid* or perceive* or percept* or promot* or program* or change*)).ti,ab. (17)
112. (problem* behavio?r* adj5 (reduc* or decreas* or increas* or lower* or fewer or improv* or enhance* or extend* or develop* or prevalen* or daily or weekly or current or rate or rates or behavio?r* or knowledge or prevent* or attitude* or avoid* or perceive* or percept* or promot* or program* or change*)).ti,ab. (73)
113. (volatile behavio?r* adj5 (reduc* or decreas* or increas* or lower* or fewer or improv* or enhance* or extend* or develop* or prevalen* or daily or weekly or current or rate or rates or behavio?r* or knowledge or prevent* or attitude* or avoid* or perceive* or percept* or promot* or program* or change*)).ti,ab. (27)
114. ((antisocial or anti-social) adj5 (reduc* or decreas* or increas* or lower* or fewer or improv* or enhance* or extend* or develop* or prevalen* or daily or weekly or current or rate or rates or behavio?r* or knowledge or prevent* or attitude* or avoid* or perceive* or percept* or promot* or program* or change*)).ti,ab. (126)
115. (perpetrat* adj5 (reduc* or decreas* or increas* or lower* or fewer or improv* or enhance* or extend* or develop* or prevalen* or daily or weekly or current or rate or rates or behavio?r* or knowledge or prevent* or attitude* or avoid* or perceive* or percept* or promot* or program* or change*)).ti,ab. (28)
116. (bully* adj5 (reduc* or decreas* or increas* or lower* or fewer or improv* or enhance* or extend* or develop* or prevalen* or daily or weekly or current or rate or rates or behavio?r* or knowledge or prevent* or attitude* or avoid* or perceive* or percept* or promot* or program* or change*)).ti,ab. (55)
117. (victim* adj5 (reduc* or decreas* or increas* or lower* or fewer or improv* or enhance* or extend* or develop* or prevalen* or daily or weekly or current or rate or rates or behavio?r* or knowledge or prevent* or attitude* or avoid* or perceive* or percept* or promot* or program* or change*)).ti,ab. (358)
118. (cyberbully* adj5 (reduc* or decreas* or increas* or lower* or fewer or improv* or enhance* or extend* or develop* or prevalen* or daily or weekly or current or rate or rates or behavio?r* or knowledge or prevent* or attitude* or avoid* or perceive* or percept* or promot* or program* or change*)).ti,ab. (3)
119. (conflict* adj5 (reduc* or decreas* or increas* or lower* or fewer or improv* or enhance* or extend* or develop* or prevalen* or daily or weekly or current or rate or rates or behavio?r* or knowledge or prevent* or attitude* or avoid* or perceive* or percept* or promot* or program* or change*)).ti,ab. (3905)
120. (positive behavio?r* adj5 (reduc* or decreas* or increas* or lower* or fewer or improv* or enhance* or extend* or develop* or prevalen* or daily or weekly or current or rate or rates or behavio?r* or knowledge or prevent* or attitude* or avoid* or perceive* or percept* or promot* or program* or change*)).ti,ab. (40)
121. (improv* behavio?r* adj5 (reduc* or decreas* or increas* or lower* or fewer or improv* or enhance* or extend* or develop* or prevalen* or daily or weekly or current or rate or rates or behavio?r* or knowledge or prevent* or attitude* or avoid* or perceive* or percept* or promot* or program* or change*)).ti,ab. (36)
122. (social behavio?r* adj5 (reduc* or decreas* or increas* or lower* or fewer or improv* or enhance* or extend* or develop* or prevalen* or daily or weekly or current or rate or rates or behavio?r* or knowledge or prevent* or attitude* or avoid* or perceive* or percept* or promot* or program* or change*)).ti,ab. (541)
123. ((social-emotion* or socio-emotion*) adj5 (reduc* or decreas* or increas* or lower* or fewer or improv* or enhance* or extend* or develop* or prevalen* or daily or weekly or current or rate or rates or behavio?r* or knowledge or prevent* or attitude* or avoid* or perceive* or percept* or promot* or program* or change*)).ti,ab. (59)
124. (social-charact* adj5 (reduc* or decreas* or increas* or lower* or fewer or improv* or enhance* or extend* or develop* or prevalen* or daily or weekly or current or rate or rates or behavio?r* or knowledge or prevent* or attitude* or avoid* or perceive* or percept* or promot* or program* or change*)).ti,ab. (25)
125. (social-inclusion adj5 (reduc* or decreas* or increas* or lower* or fewer or improv* or enhance* or extend* or develop* or prevalen* or daily or weekly or current or rate or rates or behavio?r* or knowledge or prevent* or attitude* or avoid* or perceive* or percept* or promot* or program* or change*)).ti,ab. (142)
126. (social develop* adj5 (reduc* or decreas* or increas* or lower* or fewer or improv* or enhance* or extend* or develop* or prevalen* or daily or weekly or current or rate or rates or behavio?r* or knowledge or prevent* or attitude* or avoid* or perceive* or percept* or promot* or program* or change*)).ti,ab. (1637)
127. (social competen* adj5 (reduc* or decreas* or increas* or lower* or fewer or improv* or enhance* or extend* or develop* or prevalen* or daily or weekly or current or rate or rates or behavio?r* or knowledge or prevent* or attitude* or avoid* or perceive* or percept* or promot* or program* or change*)).ti,ab. (11)
128. (emotion* develop* adj5 (reduc* or decreas* or increas* or lower* or fewer or improv* or enhance* or extend* or develop* or prevalen* or daily or weekly or current or rate or rates or behavio?r* or knowledge or prevent* or attitude* or avoid* or perceive* or percept* or promot* or program* or change*)).ti,ab. (41)
129. (conduct adj5 (reduc* or decreas* or increas* or lower* or fewer or improv* or enhance* or extend* or develop* or prevalen* or daily or weekly or current or rate or rates or behavio?r* or knowledge or prevent* or attitude* or avoid* or perceive* or percept* or promot* or program* or change*)).ti,ab. (990)
130. (respect adj5 (reduc* or decreas* or increas* or lower* or fewer or improv* or enhance* or extend* or develop* or prevalen* or daily or weekly or current or rate or rates or behavio?r* or knowledge or prevent* or attitude* or avoid* or perceive* or percept* or promot* or program* or change*)).ti,ab. (3353)
131. ((safety or safe) adj5 (reduc* or decreas* or increas* or lower* or fewer or improv* or enhance* or extend* or develop* or prevalen* or daily or weekly or current or rate or rates or behavio?r* or knowledge or prevent* or attitude* or avoid* or perceive* or percept* or promot* or program* or change*)).ti,ab. (2679)
132. or/69-130 (26093)
133. prevent*.ti,ab. (15825)
134. program*.ti,ab. (63714)
135. intervent*.ti,ab. (21316)
136. restorative approach*.ti,ab. (2)
137. control school?.ti,ab. (23)
138. (trial or trials).ti,ab. (3873)
139. or/132-137 (98302)
140. 39 and 68 and 131 and 138 (223)

## EBSCO Education Abstracts (H.W. Wilson)

| Database name | Education Abstracts (H.W. Wilson) |
| --- | --- |
| Database platform | EBSCO |
| Dates of database coverage | Complete database to date searched. |
| Date searched | 17/01/2020 |
| Searched by | JF |
| Number of results | 4567 |
| EndNote import order | 10 |
| Number of results once duplicates removed | 2056 |
| Search strategy notes | * is used for truncation. # is used for an optional wildcard. Search strings starting with TI search the title only. Search strings starting with AB search the abstract only. Search strings starting with SO search the journal title only. N/*n* search for terms within *n* words of each other, in any order. |

| Search number | Query |
| --- | --- |
| S1 | (TI child*) or (AB child*) |
| S2 | (TI boy#) or (AB boy#) |
| S3 | (TI girl#) or (AB girl#) |
| S4 | (TI (schoolage or (school N1 age))) or (AB (schoolage or (school N1 age))) |
| S5 | (TI schoolchild*) or (AB schoolchild*) |
| S6 | (TI youngster#) OR (AB youngster#) |
| S7 | (TI minor#) OR (AB minor#) |
| S8 | (TI pupil*) OR (AB pupil*) |
| S9 | ((TI student*) OR (AB student*)) NOT ((TI "medical student*") or (AB "medical student*")) |
| S10 | (TI prepubescen*) OR (AB prepubescen*) |
| S11 | (TI pubescent*) or (AB pubescent*) |
| S12 | (TI adolescent*) OR (AB adolescent*) |
| S13 | (TI juvenil*) OR (AB juvenil*) |
| S14 | (TI underage*) or (AB underage*) |
| S15 | (TI (preteen* or pre-teen*)) OR (AB (preteen* or pre-teen*)) |
| S16 | (TI (teen or teens)) OR (AB (teen or teens)) |
| S17 | (TI teenage*) OR (AB teenage*) |
| S18 | (TI (youth or youths)) OR (AB (youth or youths)) |
| S19 | (TI "young person*") OR (AB "young person*") |
| S20 | (TI "young people*") OR (AB "young people*") |
| S21 | (TI (kid or kids)) OR (AB (kid or kids)) |
| S22 | (TI (transition n4 adult*)) OR (AB (transition N4 adult*)) |
| S23 | (TI "emerging adult*") OR (AB "emerging adult*") |
| S24 | (TI "young adult*") OR (AB "young adult*") |
| S25 | (TI parent#) OR (AB parent#) |
| S26 | (TI mother#) OR (AB mother#) |
| S27 | (TI father#) or (AB father#) |
| S28 | (TI guardian#) OR (AB guardian#) |
| S29 | (TI teacher#) OR (AB teacher#) |
| S30 | (SO child*) |
| S31 | (SO adolescen*) |
| S32 | (SO youth*) |
| S33 | (SO school*) |
| S34 | (TI ("5" or "6" or "7" or "8" or "9" or "10" or "11" or "12" or "13" or "14" or "15" or "16" or "17" or "18") N1 ("year* old" or "year* of age")) OR (AB ("5" or "6" or "7" or "8" or "9" or "10" or "11" or "12" or "13" or "14" or "15" or "16" or "17" or "18") N1 ("year* old" or "year* of age")) |
| S35 | (TI (five or six or seven or eight or nine or ten or eleven or twelve or thirteen or fourteen or fifteen or sixteen or seventeen or eighteen) N1 ("year* old" or "year* of age")) OR (AB (five or six or seven or eight or nine or ten or eleven or twelve or thirteen or fourteen or fifteen or sixteen or seventeen or eighteen) N1 ("year* old" or "year* of age")) |
| S36 | (TI (age* N1 ("5" or "6" or "7" or "8" or "9" or "10" or "11" or "12" or "13" or "14" or "15" or "16" or "17" or "18") N1 year*)) OR (AB (age* N1 ("5" or "6" or "7" or "8" or "9" or "10" or "11" or "12" or "13" or "14" or "15" or "16" or "17" or "18") N1 year*)) |
| S37 | (TI (age* N1 (five or six or seven or eight or nine or ten or eleven or twelve or thirteen or fourteen or fifteen or sixteen or seventeen or eighteen) N1 year*)) OR (AB (age* N1 (five or six or seven or eight or nine or ten or eleven or twelve or thirteen or fourteen or fifteen or sixteen or seventeen or eighteen) N1 year*)) |
| S38 | (TI (primary or secondary or year) N1 ("1" or "2" or "3" or "4" or "5" or "6" or "7" or "8" or "9" or "10" or "11" or "12" or "13")) OR (AB (primary or secondary or year) N1 ("1" or "2" or "3" or "4" or "5" or "6" or "7" or "8" or "9" or "10" or "11" or "12" or "13")) |
| S39 | (TI grade# N1 (first or second or third or fourth or fifth or sixth or seventh or eighth or ninth or tenth or eleventh or twelfth)) OR (AB grade# N1 (first or second or third or fourth or fifth or sixth or seventh or eighth or ninth or tenth or eleventh or twelfth)) |
| S40 | S1 OR S2 OR S3 OR S4 OR S5 OR S6 OR S7 OR S8 OR S9 OR S10 OR S11 OR S12 OR S13 OR S14 OR S15 OR S16 OR S17 OR S18 OR S19 OR S20 OR S21 OR S22 OR S23 OR S24 OR S25 OR S26 OR S27 OR S28 OR S29 OR S30 OR S31 OR S32 OR S33 OR S34 OR S35 OR S36 OR S37 OR S38 OR S39 |
| S41 | (TI (school# N5 (based or level or wide or led or ethos or environment or organi#ation* or climate or toolkit# or approach* or "action group#" or practice# or whole or health* or leader* or cultur* or manag* or governance or system# or communit*))) OR (AB (school# N5 (based or level or wide or led or ethos or environment or organi#ation* or climate or toolkit# or approach* or "action group#" or practice# or whole or health* or leader* or cultur* or manag* or governance or system# or communit*))) |
| S42 | (TI schoolwide) or (AB schoolwide) |
| S43 | (TI "restor* justice") OR (AB "restor* justice") |
| S44 | (TI school#) or (AB school#) |
| S45 | (TI "peer-to-peer") OR (AB "peer-to-peer") |
| S46 | (TI (peer N2 (led or leader* or participat* or mediat* or helper# or resistan* or wide or action# or influen*))) OR (AB (peer N2 (led or leader* or participat* or mediat* or helper# or resistan* or wide or action# or influen*))) |
| S47 | (TI (relationship* N2 (led or leader* or participat* or mediat* or helper# or resistan* or wide or action# or influen*))) OR (AB (relationship* N2 (led or leader* or participat* or mediat* or helper# or resistan* or wide or action# or influen*))) |
| S48 | (TI ((community or communities) N2 (led or leader* or participat* or mediat* or helper# or resistan* or wide or action# or influen* or relationship*))) OR (AB ((community or communities) N2 (led or leader* or participat* or mediat* or helper# or resistan* or wide or action# or influen* or relationship*))) |
| S49 | ((TI (pupil# N2 (led or leader* or participat* or mediat* or helper# or resistan* or wide or action# or influen* or relationship*)) OR (AB (pupil# N2 (led or leader* or participat* or mediat* or helper# or resistan* or wide or action# or influen* or relationship*))) |
| S50 | (TI ((child or children) N2 (led or leader* or participat* or mediat* or helper# or resistan* or wide or action# or influen*))) OR (AB (child or children*) N2 (led or leader* or participat* or mediat* or helper# or resistan* or wide or action# or influen*))) |
| S51 | (TI (("young people" or "young person") N2 (led or leader* or participat* or mediat* or helper# or resistan* or wide or action# or influen*))) OR (AB (("young people" or "young person") N2 (led or leader* or participat* or mediat* or helper# or resistan* or wide or action# or influen*))) |
| S52 | (TI ((teen or teens or teenage*) N2 (led or leader* or participat* or mediat* or helper# or resistan* or wide or action# or influen*))) OR (AB ((teen or teens or teenage*) N2 (led or leader* or participat* or mediat* or helper# or resistan* or wide or action# or influen*))) |
| S53 | (TI (adolescen* N2 (led or leader* or participat* or mediat* or helper# or resistan* or wide or action# or influen*))) OR (AB (adolescen* N2 (led or leader* or participat* or mediat* or helper# or resistan* or wide or action# or influen*))) |
| S54 | (TI (youth# N2 (led or leader* or participat* or mediat* or helper# or resistan* or wide or action# or influen*))) OR (AB (youth# N2 (led or leader* or participat* or mediat* or helper# or resistan* or wide or action# or influen*))) |
| S55 | (TI (student# N2 (led or leader* or participat* or mediat* or helper# or resistan* or wide or action# or influen* or relationship*))) OR (AB (student# N2 (led or leader* or participat* or mediat* or helper# or resistan* or wide or action# or influen* or relationship*))) |
| S56 | (TI (teach* N2 (led or leader* or participat* or mediat* or helper# or resistan* or wide or action# or influen* or relationship*))) OR (AB (teach* N2 (led or leader* or participat* or mediat* or helper# or resistan* or wide or action# or influen* or relationship*))) |
| S57 | (TI (curricul* N2 (led or leader* or participat* or mediat* or helper# or resistan* or wide or action# or influen* or relationship*))) OR (AB (curricul* N2 (led or leader* or participat* or mediat* or helper# or resistan* or wide or action# or influen* or relationship*))) |
| S58 | (TI (administrat* N2 (led or leader* or participat* or mediat* or helper# or resistan* or wide or action# or influen* or relationship*))) OR (AB (administrat* N2 (led or leader* or participat* or mediat* or helper# or resistan* or wide or action# or influen* or relationship*))) |
| S59 | (TI ((personnel or staff) N2 (led or leader* or participat* or mediat* or helper# or resistan* or wide or action# or influen* or relationship*))) OR (AB ((personnel or staff) N2 (led or leader* or participat* or mediat* or helper# or resistan* or wide or action# or influen* or relationship*))) |
| S60 | (TI (volunteer* N2 (led or leader* or participat* or mediat* or helper# or resistan* or wide or action# or influen* or relationship*))) OR (AB (volulnteer* N2 (led or leader* or participat* or mediat* or helper# or resistan* or wide or action# or influen* or relationship*))) |
| S61 | (TI ((advocacy or advocate) N2 (led or leader* or participat* or mediat* or helper# or resistan* or wide or action# or influen* or relationship*))) OR (AB ((advocacy or advocate) N2 (led or leader* or participat* or mediat* or helper# or resistan* or wide or action# or influen* or relationship*))) |
| S62 | (TI (parent* N2 (led or leader* or participat* or mediat* or helper# or resistan* or wide or action# or influen*))) OR (AB (parent* N2 (led or leader* or participat* or mediat* or helper# or resistan* or wide or action# or influen*))) |
| S63 | (TI (environment* N2 (led or leader* or participat* or mediat* or helper# or resistan* or wide or action# or influen* or relationship*))) OR (AB (environment* N2 (led or leader* or participat* or mediat* or helper# or resistan* or wide or action# or influen* or relationship*))) |
| S64 | (TI ("socio-ecolog*" N2 (led or leader* or participat* or mediat* or helper# or resistan* or wide or action# or influen* or relationship*))) OR (AB ("socio-ecolog*" N2 (led or leader* or participat* or mediat* or helper# or resistan* or wide or action# or influen* or relationship*))) |
| S65 | (TI (learn* N2 (led or leader* or participat* or mediat* or helper# or resistan* or wide or action# or influen* or relationship*))) OR (AB (learn* N2 (led or leader* or participat* or mediat* or helper# or resistan* or wide or action# or influen* or relationship*))) |
| S66 | (TI ((policy or policies) N2 (led or leader* or participat* or mediat* or helper# or resistan* or wide or action# or influen* or relationship*))) OR (AB ((policy or policies) N2 (led or leader* or participat* or mediat* or helper# or resistan* or wide or action# or influen* or relationship*))) |
| S67 | S45 OR S46 OR S47 OR S48 OR S49 OR S50 OR S51 OR S52 OR S53 OR S54 OR S55 OR S56 OR S57 OR S58 OR S59 OR S60 OR S61 OR S62 OR S63 OR S64 OR S65 OR S66 |
| S68 | S44 AND S67 |
| S69 | S41 OR S42 OR S43 OR S68 |
| S70 | (TI ((smoking or smoked or smoke or smoker) N5 (reduc* or decreas* or increas* or lower* or fewer or improv* or enhance* or extend* or develop* or prevalen* or daily or weekly or current or rate or rates or behavio#r* or knowledge or prevent* or attitude* or avoid* or perceive* or percept* or promot* or program* or change*))) OR (AB ((smoking or smoked or smoke or smoker) N5 (reduc* or decreas* or increas* or lower* or fewer or improv* or enhance* or extend* or develop* or prevalen* or daily or weekly or current or rate or rates or behavio#r* or knowledge or prevent* or attitude* or avoid* or perceive* or percept* or promot* or program* or change*))) |
| S71 | (TI (tobacco N5 (reduc* or decreas* or increas* or lower* or fewer or improv* or enhance* or extend* or develop* or prevalen* or daily or weekly or current or rate or rates or behavio#r* or knowledge or prevent* or attitude* or avoid* or perceive* or percept* or promot* or program* or change*)) OR (AB (tobacco N5 (reduc* or decreas* or increas* or lower* or fewer or improv* or enhance* or extend* or develop* or prevalen* or daily or weekly or current or rate or rates or behavio#r* or knowledge or prevent* or attitude* or avoid* or perceive* or percept* or promot* or program* or change*)) |
| S72 | (TI (cigarette# N5 (reduc* or decreas* or increas* or lower* or fewer or improv* or enhance* or extend* or develop* or prevalen* or daily or weekly or current or rate or rates or behavio#r* or knowledge or prevent* or attitude* or avoid* or perceive* or percept* or promot* or program* or change*)) OR (AB (cigarette# N5 (reduc* or decreas* or increas* or lower* or fewer or improv* or enhance* or extend* or develop* or prevalen* or daily or weekly or current or rate or rates or behavio#r* or knowledge or prevent* or attitude* or avoid* or perceive* or percept* or promot* or program* or change*)) |
| S73 | (TI (marijuana N5 (reduc* or decreas* or increas* or lower* or fewer or improv* or enhance* or extend* or develop* or prevalen* or daily or weekly or current or rate or rates or behavio#r* or knowledge or prevent* or attitude* or avoid* or perceive* or percept* or promot* or program* or change*)) OR (AB (marijuana N5 (reduc* or decreas* or increas* or lower* or fewer or improv* or enhance* or extend* or develop* or prevalen* or daily or weekly or current or rate or rates or behavio#r* or knowledge or prevent* or attitude* or avoid* or perceive* or percept* or promot* or program* or change*)) |
| S74 | (TI (cannabis N5 (reduc* or decreas* or increas* or lower* or fewer or improv* or enhance* or extend* or develop* or prevalen* or daily or weekly or current or rate or rates or behavio#r* or knowledge or prevent* or attitude* or avoid* or perceive* or percept* or promot* or program* or change*)) OR (AB (cannabis N5 (reduc* or decreas* or increas* or lower* or fewer or improv* or enhance* or extend* or develop* or prevalen* or daily or weekly or current or rate or rates or behavio#r* or knowledge or prevent* or attitude* or avoid* or perceive* or percept* or promot* or program* or change*)) |
| S75 | (TI (solvent# N5 (reduc* or decreas* or increas* or lower* or fewer or improv* or enhance* or extend* or develop* or prevalen* or daily or weekly or current or rate or rates or behavio#r* or knowledge or prevent* or attitude* or avoid* or perceive* or percept* or promot* or program* or change*)) OR (AB (solvent# N5 (reduc* or decreas* or increas* or lower* or fewer or improv* or enhance* or extend* or develop* or prevalen* or daily or weekly or current or rate or rates or behavio#r* or knowledge or prevent* or attitude* or avoid* or perceive* or percept* or promot* or program* or change*)) |
| S76 | (TI (alcohol N5 (reduc* or decreas* or increas* or lower* or fewer or improv* or enhance* or extend* or develop* or prevalen* or daily or weekly or current or rate or rates or behavio#r* or knowledge or prevent* or attitude* or avoid* or perceive* or percept* or promot* or program* or change*)) OR (AB (alcohol N5 (reduc* or decreas* or increas* or lower* or fewer or improv* or enhance* or extend* or develop* or prevalen* or daily or weekly or current or rate or rates or behavio#r* or knowledge or prevent* or attitude* or avoid* or perceive* or percept* or promot* or program* or change*)) |
| S77 | (TI ("binge drink*" N5 (reduc* or decreas* or increas* or lower* or fewer or improv* or enhance* or extend* or develop* or prevalen* or daily or weekly or current or rate or rates or behavio#r* or knowledge or prevent* or attitude* or avoid* or perceive* or percept* or promot* or program* or change*)) OR (AB ("binge drink*" N5 (reduc* or decreas* or increas* or lower* or fewer or improv* or enhance* or extend* or develop* or prevalen* or daily or weekly or current or rate or rates or behavio#r* or knowledge or prevent* or attitude* or avoid* or perceive* or percept* or promot* or program* or change*)) |
| S78 | (TI ("underage drink*" N5 (reduc* or decreas* or increas* or lower* or fewer or improv* or enhance* or extend* or develop* or prevalen* or daily or weekly or current or rate or rates or behavio#r* or knowledge or prevent* or attitude* or avoid* or perceive* or percept* or promot* or program* or change*)) OR (AB ("underage drink*" N5 (reduc* or decreas* or increas* or lower* or fewer or improv* or enhance* or extend* or develop* or prevalen* or daily or weekly or current or rate or rates or behavio#r* or knowledge or prevent* or attitude* or avoid* or perceive* or percept* or promot* or program* or change*)) |
| S79 | (TI ("underage drink*" N5 (reduc* or decreas* or increas* or lower* or fewer or improv* or enhance* or extend* or develop* or prevalen* or daily or weekly or current or rate or rates or behavio#r* or knowledge or prevent* or attitude* or avoid* or perceive* or percept* or promot* or program* or change*)) OR (AB ("underage drink*" N5 (reduc* or decreas* or increas* or lower* or fewer or improv* or enhance* or extend* or develop* or prevalen* or daily or weekly or current or rate or rates or behavio#r* or knowledge or prevent* or attitude* or avoid* or perceive* or percept* or promot* or program* or change*)) |
| S80 | (TI (substance# N5 (reduc* or decreas* or increas* or lower* or fewer or improv* or enhance* or extend* or develop* or prevalen* or daily or weekly or current or rate or rates or behavio#r* or knowledge or prevent* or attitude* or avoid* or perceive* or percept* or promot* or program* or change*)) OR (AB (substance# N5 (reduc* or decreas* or increas* or lower* or fewer or improv* or enhance* or extend* or develop* or prevalen* or daily or weekly or current or rate or rates or behavio#r* or knowledge or prevent* or attitude* or avoid* or perceive* or percept* or promot* or program* or change*)) |
| S81 | (TI (drug# N5 (reduc* or decreas* or increas* or lower* or fewer or improv* or enhance* or extend* or develop* or prevalen* or daily or weekly or current or rate or rates or behavio#r* or knowledge or prevent* or attitude* or avoid* or perceive* or percept* or promot* or program* or change*)) OR (AB (drug# N5 (reduc* or decreas* or increas* or lower* or fewer or improv* or enhance* or extend* or develop* or prevalen* or daily or weekly or current or rate or rates or behavio#r* or knowledge or prevent* or attitude* or avoid* or perceive* or percept* or promot* or program* or change*)) |
| S82 | (TI ("education* attain*" N5 (reduc* or decreas* or increas* or lower* or fewer or improv* or enhance* or extend* or develop* or prevalen* or daily or weekly or current or rate or rates or behavio#r* or knowledge or prevent* or attitude* or avoid* or perceive* or percept* or promot* or program* or change*)) OR (AB ("education* attain*" N5 (reduc* or decreas* or increas* or lower* or fewer or improv* or enhance* or extend* or develop* or prevalen* or daily or weekly or current or rate or rates or behavio#r* or knowledge or prevent* or attitude* or avoid* or perceive* or percept* or promot* or program* or change*)) |
| S83 | (TI ("academic attain*" N5 (reduc* or decreas* or increas* or lower* or fewer or improv* or enhance* or extend* or develop* or prevalen* or daily or weekly or current or rate or rates or behavio#r* or knowledge or prevent* or attitude* or avoid* or perceive* or percept* or promot* or program* or change*)) OR (AB ("academic attain*" N5 (reduc* or decreas* or increas* or lower* or fewer or improv* or enhance* or extend* or develop* or prevalen* or daily or weekly or current or rate or rates or behavio#r* or knowledge or prevent* or attitude* or avoid* or perceive* or percept* or promot* or program* or change*)) |
| S84 | (TI ("student* attain*" N5 (reduc* or decreas* or increas* or lower* or fewer or improv* or enhance* or extend* or develop* or prevalen* or daily or weekly or current or rate or rates or behavio#r* or knowledge or prevent* or attitude* or avoid* or perceive* or percept* or promot* or program* or change*)) OR (AB ("student* attain*" N5 (reduc* or decreas* or increas* or lower* or fewer or improv* or enhance* or extend* or develop* or prevalen* or daily or weekly or current or rate or rates or behavio#r* or knowledge or prevent* or attitude* or avoid* or perceive* or percept* or promot* or program* or change*)) |
| S85 | (TI ("education* achieve*" N5 (reduc* or decreas* or increas* or lower* or fewer or improv* or enhance* or extend* or develop* or prevalen* or daily or weekly or current or rate or rates or behavio#r* or knowledge or prevent* or attitude* or avoid* or perceive* or percept* or promot* or program* or change*)) OR (AB ("education* achieve*" N5 (reduc* or decreas* or increas* or lower* or fewer or improv* or enhance* or extend* or develop* or prevalen* or daily or weekly or current or rate or rates or behavio#r* or knowledge or prevent* or attitude* or avoid* or perceive* or percept* or promot* or program* or change*)) |
| S86 | (TI ("academic achieve*" N5 (reduc* or decreas* or increas* or lower* or fewer or improv* or enhance* or extend* or develop* or prevalen* or daily or weekly or current or rate or rates or behavio#r* or knowledge or prevent* or attitude* or avoid* or perceive* or percept* or promot* or program* or change*)) OR (AB ("academic achieve*" N5 (reduc* or decreas* or increas* or lower* or fewer or improv* or enhance* or extend* or develop* or prevalen* or daily or weekly or current or rate or rates or behavio#r* or knowledge or prevent* or attitude* or avoid* or perceive* or percept* or promot* or program* or change*)) |
| S87 | (TI ("student* achieve*" N5 (reduc* or decreas* or increas* or lower* or fewer or improv* or enhance* or extend* or develop* or prevalen* or daily or weekly or current or rate or rates or behavio#r* or knowledge or prevent* or attitude* or avoid* or perceive* or percept* or promot* or program* or change*)) OR (AB ("student* achieve*" N5 (reduc* or decreas* or increas* or lower* or fewer or improv* or enhance* or extend* or develop* or prevalen* or daily or weekly or current or rate or rates or behavio#r* or knowledge or prevent* or attitude* or avoid* or perceive* or percept* or promot* or program* or change*)) |
| S88 | (TI ("education* perform*" N5 (reduc* or decreas* or increas* or lower* or fewer or improv* or enhance* or extend* or develop* or prevalen* or daily or weekly or current or rate or rates or behavio#r* or knowledge or prevent* or attitude* or avoid* or perceive* or percept* or promot* or program* or change*)) OR (AB ("education* perform*" N5 (reduc* or decreas* or increas* or lower* or fewer or improv* or enhance* or extend* or develop* or prevalen* or daily or weekly or current or rate or rates or behavio#r* or knowledge or prevent* or attitude* or avoid* or perceive* or percept* or promot* or program* or change*)) |
| S89 | (TI ("academic perform*" N5 (reduc* or decreas* or increas* or lower* or fewer or improv* or enhance* or extend* or develop* or prevalen* or daily or weekly or current or rate or rates or behavio#r* or knowledge or prevent* or attitude* or avoid* or perceive* or percept* or promot* or program* or change*)) OR (AB ("academic perform*" N5 (reduc* or decreas* or increas* or lower* or fewer or improv* or enhance* or extend* or develop* or prevalen* or daily or weekly or current or rate or rates or behavio#r* or knowledge or prevent* or attitude* or avoid* or perceive* or percept* or promot* or program* or change*)) |
| S90 | (TI ("student* perform*" N5 (reduc* or decreas* or increas* or lower* or fewer or improv* or enhance* or extend* or develop* or prevalen* or daily or weekly or current or rate or rates or behavio#r* or knowledge or prevent* or attitude* or avoid* or perceive* or percept* or promot* or program* or change*)) OR (AB ("student* perform*" N5 (reduc* or decreas* or increas* or lower* or fewer or improv* or enhance* or extend* or develop* or prevalen* or daily or weekly or current or rate or rates or behavio#r* or knowledge or prevent* or attitude* or avoid* or perceive* or percept* or promot* or program* or change*)) |
| S91 | (TI ("education* outcome#" N5 (reduc* or decreas* or increas* or lower* or fewer or improv* or enhance* or extend* or develop* or prevalen* or daily or weekly or current or rate or rates or behavio#r* or knowledge or prevent* or attitude* or avoid* or perceive* or percept* or promot* or program* or change*)) OR (AB ("education* outcome#" N5 (reduc* or decreas* or increas* or lower* or fewer or improv* or enhance* or extend* or develop* or prevalen* or daily or weekly or current or rate or rates or behavio#r* or knowledge or prevent* or attitude* or avoid* or perceive* or percept* or promot* or program* or change*)) |
| S92 | (TI ("academic outcome#" N5 (reduc* or decreas* or increas* or lower* or fewer or improv* or enhance* or extend* or develop* or prevalen* or daily or weekly or current or rate or rates or behavio#r* or knowledge or prevent* or attitude* or avoid* or perceive* or percept* or promot* or program* or change*)) OR (AB ("academic outcome#" N5 (reduc* or decreas* or increas* or lower* or fewer or improv* or enhance* or extend* or develop* or prevalen* or daily or weekly or current or rate or rates or behavio#r* or knowledge or prevent* or attitude* or avoid* or perceive* or percept* or promot* or program* or change*)) |
| S93 | (TI ("student* outcome#" N5 (reduc* or decreas* or increas* or lower* or fewer or improv* or enhance* or extend* or develop* or prevalen* or daily or weekly or current or rate or rates or behavio#r* or knowledge or prevent* or attitude* or avoid* or perceive* or percept* or promot* or program* or change*)) OR (AB ("student* outcome#" N5 (reduc* or decreas* or increas* or lower* or fewer or improv* or enhance* or extend* or develop* or prevalen* or daily or weekly or current or rate or rates or behavio#r* or knowledge or prevent* or attitude* or avoid* or perceive* or percept* or promot* or program* or change*)) |
| S94 | (TI ("education* commitment" N5 (reduc* or decreas* or increas* or lower* or fewer or improv* or enhance* or extend* or develop* or prevalen* or daily or weekly or current or rate or rates or behavio#r* or knowledge or prevent* or attitude* or avoid* or perceive* or percept* or promot* or program* or change*)) OR (AB ("education* commitment" N5 (reduc* or decreas* or increas* or lower* or fewer or improv* or enhance* or extend* or develop* or prevalen* or daily or weekly or current or rate or rates or behavio#r* or knowledge or prevent* or attitude* or avoid* or perceive* or percept* or promot* or program* or change*)) |
| S95 | (TI ("education* commitment" N5 (reduc* or decreas* or increas* or lower* or fewer or improv* or enhance* or extend* or develop* or prevalen* or daily or weekly or current or rate or rates or behavio#r* or knowledge or prevent* or attitude* or avoid* or perceive* or percept* or promot* or program* or change*)) OR (AB ("education* commitment" N5 (reduc* or decreas* or increas* or lower* or fewer or improv* or enhance* or extend* or develop* or prevalen* or daily or weekly or current or rate or rates or behavio#r* or knowledge or prevent* or attitude* or avoid* or perceive* or percept* or promot* or program* or change*)) |
| S96 | (TI ("academic commitment" N5 (reduc* or decreas* or increas* or lower* or fewer or improv* or enhance* or extend* or develop* or prevalen* or daily or weekly or current or rate or rates or behavio#r* or knowledge or prevent* or attitude* or avoid* or perceive* or percept* or promot* or program* or change*)) OR (AB ("academic commitment" N5 (reduc* or decreas* or increas* or lower* or fewer or improv* or enhance* or extend* or develop* or prevalen* or daily or weekly or current or rate or rates or behavio#r* or knowledge or prevent* or attitude* or avoid* or perceive* or percept* or promot* or program* or change*)) |
| S97 | (TI ("academic commitment" N5 (reduc* or decreas* or increas* or lower* or fewer or improv* or enhance* or extend* or develop* or prevalen* or daily or weekly or current or rate or rates or behavio#r* or knowledge or prevent* or attitude* or avoid* or perceive* or percept* or promot* or program* or change*)) OR (AB ("academic commitment" N5 (reduc* or decreas* or increas* or lower* or fewer or improv* or enhance* or extend* or develop* or prevalen* or daily or weekly or current or rate or rates or behavio#r* or knowledge or prevent* or attitude* or avoid* or perceive* or percept* or promot* or program* or change*)) |
| S98 | (TI ("student* commitment" N5 (reduc* or decreas* or increas* or lower* or fewer or improv* or enhance* or extend* or develop* or prevalen* or daily or weekly or current or rate or rates or behavio#r* or knowledge or prevent* or attitude* or avoid* or perceive* or percept* or promot* or program* or change*)) OR (AB ("student* commitment" N5 (reduc* or decreas* or increas* or lower* or fewer or improv* or enhance* or extend* or develop* or prevalen* or daily or weekly or current or rate or rates or behavio#r* or knowledge or prevent* or attitude* or avoid* or perceive* or percept* or promot* or program* or change*)) |
| S99 | (TI ("education* engage*" N5 (reduc* or decreas* or increas* or lower* or fewer or improv* or enhance* or extend* or develop* or prevalen* or daily or weekly or current or rate or rates or behavio#r* or knowledge or prevent* or attitude* or avoid* or perceive* or percept* or promot* or program* or change*)) OR (AB ("education* engage*" N5 (reduc* or decreas* or increas* or lower* or fewer or improv* or enhance* or extend* or develop* or prevalen* or daily or weekly or current or rate or rates or behavio#r* or knowledge or prevent* or attitude* or avoid* or perceive* or percept* or promot* or program* or change*)) |
| S100 | (TI ("academic engage*" N5 (reduc* or decreas* or increas* or lower* or fewer or improv* or enhance* or extend* or develop* or prevalen* or daily or weekly or current or rate or rates or behavio#r* or knowledge or prevent* or attitude* or avoid* or perceive* or percept* or promot* or program* or change*)) OR (AB ("academic engage*" N5 (reduc* or decreas* or increas* or lower* or fewer or improv* or enhance* or extend* or develop* or prevalen* or daily or weekly or current or rate or rates or behavio#r* or knowledge or prevent* or attitude* or avoid* or perceive* or percept* or promot* or program* or change*)) |
| S101 | (TI ("student* engage*" N5 (reduc* or decreas* or increas* or lower* or fewer or improv* or enhance* or extend* or develop* or prevalen* or daily or weekly or current or rate or rates or behavio#r* or knowledge or prevent* or attitude* or avoid* or perceive* or percept* or promot* or program* or change*)) OR (AB ("student* engage*" N5 (reduc* or decreas* or increas* or lower* or fewer or improv* or enhance* or extend* or develop* or prevalen* or daily or weekly or current or rate or rates or behavio#r* or knowledge or prevent* or attitude* or avoid* or perceive* or percept* or promot* or program* or change*)) |
| S102 | (TI (dropout N5 (reduc* or decreas* or increas* or lower* or fewer or improv* or enhance* or extend* or develop* or prevalen* or daily or weekly or current or rate or rates or behavio#r* or knowledge or prevent* or attitude* or avoid* or perceive* or percept* or promot* or program* or change*)) OR (AB (dropout N5 (reduc* or decreas* or increas* or lower* or fewer or improv* or enhance* or extend* or develop* or prevalen* or daily or weekly or current or rate or rates or behavio#r* or knowledge or prevent* or attitude* or avoid* or perceive* or percept* or promot* or program* or change*)) |
| S103 | (TI (absentee* N5 (reduc* or decreas* or increas* or lower* or fewer or improv* or enhance* or extend* or develop* or prevalen* or daily or weekly or current or rate or rates or behavio#r* or knowledge or prevent* or attitude* or avoid* or perceive* or percept* or promot* or program* or change*)) OR (AB (absentee* N5 (reduc* or decreas* or increas* or lower* or fewer or improv* or enhance* or extend* or develop* or prevalen* or daily or weekly or current or rate or rates or behavio#r* or knowledge or prevent* or attitude* or avoid* or perceive* or percept* or promot* or program* or change*)) |
| S104 | (TI ((violent or violence) N5 (reduc* or decreas* or increas* or lower* or fewer or improv* or enhance* or extend* or develop* or prevalen* or daily or weekly or current or rate or rates or behavio#r* or knowledge or prevent* or attitude* or avoid* or perceive* or percept* or promot* or program* or change*))) OR (AB ((violent or violence) N5 (reduc* or decreas* or increas* or lower* or fewer or improv* or enhance* or extend* or develop* or prevalen* or daily or weekly or current or rate or rates or behavio#r* or knowledge or prevent* or attitude* or avoid* or perceive* or percept* or promot* or program* or change*))) |
| S105 | (TI ((aggression or aggressive) N5 (reduc* or decreas* or increas* or lower* or fewer or improv* or enhance* or extend* or develop* or prevalen* or daily or weekly or current or rate or rates or behavio#r* or knowledge or prevent* or attitude* or avoid* or perceive* or percept* or promot* or program* or change*))) OR (AB ((aggression or aggressive) N5 (reduc* or decreas* or increas* or lower* or fewer or improv* or enhance* or extend* or develop* or prevalen* or daily or weekly or current or rate or rates or behavio#r* or knowledge or prevent* or attitude* or avoid* or perceive* or percept* or promot* or program* or change*))) |
| S106 | (TI ((hostile or hostility) N5 (reduc* or decreas* or increas* or lower* or fewer or improv* or enhance* or extend* or develop* or prevalen* or daily or weekly or current or rate or rates or behavio#r* or knowledge or prevent* or attitude* or avoid* or perceive* or percept* or promot* or program* or change*))) OR (AB ((hostile or hostility) N5 (reduc* or decreas* or increas* or lower* or fewer or improv* or enhance* or extend* or develop* or prevalen* or daily or weekly or current or rate or rates or behavio#r* or knowledge or prevent* or attitude* or avoid* or perceive* or percept* or promot* or program* or change*))) |
| S107 | (TI (assault* N5 (reduc* or decreas* or increas* or lower* or fewer or improv* or enhance* or extend* or develop* or prevalen* or daily or weekly or current or rate or rates or behavio#r* or knowledge or prevent* or attitude* or avoid* or perceive* or percept* or promot* or program* or change*)) OR (AB (assault* N5 (reduc* or decreas* or increas* or lower* or fewer or improv* or enhance* or extend* or develop* or prevalen* or daily or weekly or current or rate or rates or behavio#r* or knowledge or prevent* or attitude* or avoid* or perceive* or percept* or promot* or program* or change*)) |
| S108 | (TI (crime N5 (reduc* or decreas* or increas* or lower* or fewer or improv* or enhance* or extend* or develop* or prevalen* or daily or weekly or current or rate or rates or behavio#r* or knowledge or prevent* or attitude* or avoid* or perceive* or percept* or promot* or program* or change*)) OR (AB (crime N5 (reduc* or decreas* or increas* or lower* or fewer or improv* or enhance* or extend* or develop* or prevalen* or daily or weekly or current or rate or rates or behavio#r* or knowledge or prevent* or attitude* or avoid* or perceive* or percept* or promot* or program* or change*)) |
| S109 | (TI ((abuse# or abusive) N5 (reduc* or decreas* or increas* or lower* or fewer or improv* or enhance* or extend* or develop* or prevalen* or daily or weekly or current or rate or rates or behavio#r* or knowledge or prevent* or attitude* or avoid* or perceive* or percept* or promot* or program* or change*))) OR (AB ((abuse# or abusive) N5 (reduc* or decreas* or increas* or lower* or fewer or improv* or enhance* or extend* or develop* or prevalen* or daily or weekly or current or rate or rates or behavio#r* or knowledge or prevent* or attitude* or avoid* or perceive* or percept* or promot* or program* or change*))) |
| S110 | (TI (misbehav* N5 (reduc* or decreas* or increas* or lower* or fewer or improv* or enhance* or extend* or develop* or prevalen* or daily or weekly or current or rate or rates or behavio#r* or knowledge or prevent* or attitude* or avoid* or perceive* or percept* or promot* or program* or change*)) OR (AB (misbehav* N5 (reduc* or decreas* or increas* or lower* or fewer or improv* or enhance* or extend* or develop* or prevalen* or daily or weekly or current or rate or rates or behavio#r* or knowledge or prevent* or attitude* or avoid* or perceive* or percept* or promot* or program* or change*)) |
| S111 | (TI (threat# N5 (reduc* or decreas* or increas* or lower* or fewer or improv* or enhance* or extend* or develop* or prevalen* or daily or weekly or current or rate or rates or behavio#r* or knowledge or prevent* or attitude* or avoid* or perceive* or percept* or promot* or program* or change*)) OR (AB (threat# N5 (reduc* or decreas* or increas* or lower* or fewer or improv* or enhance* or extend* or develop* or prevalen* or daily or weekly or current or rate or rates or behavio#r* or knowledge or prevent* or attitude* or avoid* or perceive* or percept* or promot* or program* or change*)) |
| S112 | (TI (conduct N5 (reduc* or decreas* or increas* or lower* or fewer or improv* or enhance* or extend* or develop* or prevalen* or daily or weekly or current or rate or rates or behavio#r* or knowledge or prevent* or attitude* or avoid* or perceive* or percept* or promot* or program* or change*)) OR (AB (conduct N5 (reduc* or decreas* or increas* or lower* or fewer or improv* or enhance* or extend* or develop* or prevalen* or daily or weekly or current or rate or rates or behavio#r* or knowledge or prevent* or attitude* or avoid* or perceive* or percept* or promot* or program* or change*)) |
| S113 | (TI (delinquen* N5 (reduc* or decreas* or increas* or lower* or fewer or improv* or enhance* or extend* or develop* or prevalen* or daily or weekly or current or rate or rates or behavio#r* or knowledge or prevent* or attitude* or avoid* or perceive* or percept* or promot* or program* or change*)) OR (AB (delinquen* N5 (reduc* or decreas* or increas* or lower* or fewer or improv* or enhance* or extend* or develop* or prevalen* or daily or weekly or current or rate or rates or behavio#r* or knowledge or prevent* or attitude* or avoid* or perceive* or percept* or promot* or program* or change*)) |
| S114 | (TI ("disrupt* behavio#r*" N5 (reduc* or decreas* or increas* or lower* or fewer or improv* or enhance* or extend* or develop* or prevalen* or daily or weekly or current or rate or rates or behavio#r* or knowledge or prevent* or attitude* or avoid* or perceive* or percept* or promot* or program* or change*)) OR (AB ("disrupt* behavio#r*" N5 (reduc* or decreas* or increas* or lower* or fewer or improv* or enhance* or extend* or develop* or prevalen* or daily or weekly or current or rate or rates or behavio#r* or knowledge or prevent* or attitude* or avoid* or perceive* or percept* or promot* or program* or change*)) |
| S115 | (TI ("problem* behavio#r*" N5 (reduc* or decreas* or increas* or lower* or fewer or improv* or enhance* or extend* or develop* or prevalen* or daily or weekly or current or rate or rates or behavio#r* or knowledge or prevent* or attitude* or avoid* or perceive* or percept* or promot* or program* or change*)) OR (AB ("problem* behavio#r*" N5 (reduc* or decreas* or increas* or lower* or fewer or improv* or enhance* or extend* or develop* or prevalen* or daily or weekly or current or rate or rates or behavio#r* or knowledge or prevent* or attitude* or avoid* or perceive* or percept* or promot* or program* or change*)) |
| S116 | (TI ("volatile behavio#r*" N5 (reduc* or decreas* or increas* or lower* or fewer or improv* or enhance* or extend* or develop* or prevalen* or daily or weekly or current or rate or rates or behavio#r* or knowledge or prevent* or attitude* or avoid* or perceive* or percept* or promot* or program* or change*)) OR (AB ("volatile behavio#r*" N5 (reduc* or decreas* or increas* or lower* or fewer or improv* or enhance* or extend* or develop* or prevalen* or daily or weekly or current or rate or rates or behavio#r* or knowledge or prevent* or attitude* or avoid* or perceive* or percept* or promot* or program* or change*)) |
| S117 | (TI ("volatile behavio#r*" N5 (reduc* or decreas* or increas* or lower* or fewer or improv* or enhance* or extend* or develop* or prevalen* or daily or weekly or current or rate or rates or behavio#r* or knowledge or prevent* or attitude* or avoid* or perceive* or percept* or promot* or program* or change*)) OR (AB ("volatile behavio#r*" N5 (reduc* or decreas* or increas* or lower* or fewer or improv* or enhance* or extend* or develop* or prevalen* or daily or weekly or current or rate or rates or behavio#r* or knowledge or prevent* or attitude* or avoid* or perceive* or percept* or promot* or program* or change*)) |
| S118 | (TI ((antisocial or "anti-social") N5 (reduc* or decreas* or increas* or lower* or fewer or improv* or enhance* or extend* or develop* or prevalen* or daily or weekly or current or rate or rates or behavio#r* or knowledge or prevent* or attitude* or avoid* or perceive* or percept* or promot* or program* or change*))) OR (AB ((antisocial or "anti-social") N5 (reduc* or decreas* or increas* or lower* or fewer or improv* or enhance* or extend* or develop* or prevalen* or daily or weekly or current or rate or rates or behavio#r* or knowledge or prevent* or attitude* or avoid* or perceive* or percept* or promot* or program* or change*))) |
| S119 | (TI (perpetrat* N5 (reduc* or decreas* or increas* or lower* or fewer or improv* or enhance* or extend* or develop* or prevalen* or daily or weekly or current or rate or rates or behavio#r* or knowledge or prevent* or attitude* or avoid* or perceive* or percept* or promot* or program* or change*)) OR (AB (perpetrat* N5 (reduc* or decreas* or increas* or lower* or fewer or improv* or enhance* or extend* or develop* or prevalen* or daily or weekly or current or rate or rates or behavio#r* or knowledge or prevent* or attitude* or avoid* or perceive* or percept* or promot* or program* or change*)) |
| S120 | (TI (bully* N5 (reduc* or decreas* or increas* or lower* or fewer or improv* or enhance* or extend* or develop* or prevalen* or daily or weekly or current or rate or rates or behavio#r* or knowledge or prevent* or attitude* or avoid* or perceive* or percept* or promot* or program* or change*)) OR (AB (bully* N5 (reduc* or decreas* or increas* or lower* or fewer or improv* or enhance* or extend* or develop* or prevalen* or daily or weekly or current or rate or rates or behavio#r* or knowledge or prevent* or attitude* or avoid* or perceive* or percept* or promot* or program* or change*)) |
| S121 | (TI (victim* N5 (reduc* or decreas* or increas* or lower* or fewer or improv* or enhance* or extend* or develop* or prevalen* or daily or weekly or current or rate or rates or behavio#r* or knowledge or prevent* or attitude* or avoid* or perceive* or percept* or promot* or program* or change*)) OR (AB (victim* N5 (reduc* or decreas* or increas* or lower* or fewer or improv* or enhance* or extend* or develop* or prevalen* or daily or weekly or current or rate or rates or behavio#r* or knowledge or prevent* or attitude* or avoid* or perceive* or percept* or promot* or program* or change*)) |
| S122 | (TI (cyberbully* N5 (reduc* or decreas* or increas* or lower* or fewer or improv* or enhance* or extend* or develop* or prevalen* or daily or weekly or current or rate or rates or behavio#r* or knowledge or prevent* or attitude* or avoid* or perceive* or percept* or promot* or program* or change*)) OR (AB (cyberbully* N5 (reduc* or decreas* or increas* or lower* or fewer or improv* or enhance* or extend* or develop* or prevalen* or daily or weekly or current or rate or rates or behavio#r* or knowledge or prevent* or attitude* or avoid* or perceive* or percept* or promot* or program* or change*)) |
| S123 | (TI (conflict* N5 (reduc* or decreas* or increas* or lower* or fewer or improv* or enhance* or extend* or develop* or prevalen* or daily or weekly or current or rate or rates or behavio#r* or knowledge or prevent* or attitude* or avoid* or perceive* or percept* or promot* or program* or change*)) OR (AB (conflict* N5 (reduc* or decreas* or increas* or lower* or fewer or improv* or enhance* or extend* or develop* or prevalen* or daily or weekly or current or rate or rates or behavio#r* or knowledge or prevent* or attitude* or avoid* or perceive* or percept* or promot* or program* or change*)) |
| S124 | (TI ("positive behavio#r*" N5 (reduc* or decreas* or increas* or lower* or fewer or improv* or enhance* or extend* or develop* or prevalen* or daily or weekly or current or rate or rates or behavio#r* or knowledge or prevent* or attitude* or avoid* or perceive* or percept* or promot* or program* or change*)) OR (AB ("positive behavio#r*" N5 (reduc* or decreas* or increas* or lower* or fewer or improv* or enhance* or extend* or develop* or prevalen* or daily or weekly or current or rate or rates or behavio#r* or knowledge or prevent* or attitude* or avoid* or perceive* or percept* or promot* or program* or change*)) |
| S125 | (TI ("improv* behavio#r*" N5 (reduc* or decreas* or increas* or lower* or fewer or improv* or enhance* or extend* or develop* or prevalen* or daily or weekly or current or rate or rates or behavio#r* or knowledge or prevent* or attitude* or avoid* or perceive* or percept* or promot* or program* or change*)) OR (AB ("improv* behavio#r*" N5 (reduc* or decreas* or increas* or lower* or fewer or improv* or enhance* or extend* or develop* or prevalen* or daily or weekly or current or rate or rates or behavio#r* or knowledge or prevent* or attitude* or avoid* or perceive* or percept* or promot* or program* or change*)) |
| S126 | (TI ("social behavio#r*" N5 (reduc* or decreas* or increas* or lower* or fewer or improv* or enhance* or extend* or develop* or prevalen* or daily or weekly or current or rate or rates or behavio#r* or knowledge or prevent* or attitude* or avoid* or perceive* or percept* or promot* or program* or change*)) OR (AB ("social behavio#r*" N5 (reduc* or decreas* or increas* or lower* or fewer or improv* or enhance* or extend* or develop* or prevalen* or daily or weekly or current or rate or rates or behavio#r* or knowledge or prevent* or attitude* or avoid* or perceive* or percept* or promot* or program* or change*)) |
| S127 | (TI (("social-emotion*" or "socio-emotion*") N5 (reduc* or decreas* or increas* or lower* or fewer or improv* or enhance* or extend* or develop* or prevalen* or daily or weekly or current or rate or rates or behavio#r* or knowledge or prevent* or attitude* or avoid* or perceive* or percept* or promot* or program* or change*))) OR (AB (("social-emotion*" or "socio-emotion*") N5 (reduc* or decreas* or increas* or lower* or fewer or improv* or enhance* or extend* or develop* or prevalen* or daily or weekly or current or rate or rates or behavio#r* or knowledge or prevent* or attitude* or avoid* or perceive* or percept* or promot* or program* or change*))) |
| S128 | (TI ("social-charact*" N5 (reduc* or decreas* or increas* or lower* or fewer or improv* or enhance* or extend* or develop* or prevalen* or daily or weekly or current or rate or rates or behavio#r* or knowledge or prevent* or attitude* or avoid* or perceive* or percept* or promot* or program* or change*)) OR (AB ("social-charact*" N5 (reduc* or decreas* or increas* or lower* or fewer or improv* or enhance* or extend* or develop* or prevalen* or daily or weekly or current or rate or rates or behavio#r* or knowledge or prevent* or attitude* or avoid* or perceive* or percept* or promot* or program* or change*)) |
| S129 | (TI ("social-inclusion" N5 (reduc* or decreas* or increas* or lower* or fewer or improv* or enhance* or extend* or develop* or prevalen* or daily or weekly or current or rate or rates or behavio#r* or knowledge or prevent* or attitude* or avoid* or perceive* or percept* or promot* or program* or change*)) OR (AB ("social-inclusion" N5 (reduc* or decreas* or increas* or lower* or fewer or improv* or enhance* or extend* or develop* or prevalen* or daily or weekly or current or rate or rates or behavio#r* or knowledge or prevent* or attitude* or avoid* or perceive* or percept* or promot* or program* or change*)) |
| S130 | (TI ("social develop*" N5 (reduc* or decreas* or increas* or lower* or fewer or improv* or enhance* or extend* or develop* or prevalen* or daily or weekly or current or rate or rates or behavio#r* or knowledge or prevent* or attitude* or avoid* or perceive* or percept* or promot* or program* or change*)) OR (AB ("social develop*" N5 (reduc* or decreas* or increas* or lower* or fewer or improv* or enhance* or extend* or develop* or prevalen* or daily or weekly or current or rate or rates or behavio#r* or knowledge or prevent* or attitude* or avoid* or perceive* or percept* or promot* or program* or change*)) |
| S131 | (TI ("social competen*" N5 (reduc* or decreas* or increas* or lower* or fewer or improv* or enhance* or extend* or develop* or prevalen* or daily or weekly or current or rate or rates or behavio#r* or knowledge or prevent* or attitude* or avoid* or perceive* or percept* or promot* or program* or change*)) OR (AB ("social competen*" N5 (reduc* or decreas* or increas* or lower* or fewer or improv* or enhance* or extend* or develop* or prevalen* or daily or weekly or current or rate or rates or behavio#r* or knowledge or prevent* or attitude* or avoid* or perceive* or percept* or promot* or program* or change*)) |
| S132 | (TI ("emotion* develop*" N5 (reduc* or decreas* or increas* or lower* or fewer or improv* or enhance* or extend* or develop* or prevalen* or daily or weekly or current or rate or rates or behavio#r* or knowledge or prevent* or attitude* or avoid* or perceive* or percept* or promot* or program* or change*)) OR (AB ("emotion* develop*" N5 (reduc* or decreas* or increas* or lower* or fewer or improv* or enhance* or extend* or develop* or prevalen* or daily or weekly or current or rate or rates or behavio#r* or knowledge or prevent* or attitude* or avoid* or perceive* or percept* or promot* or program* or change*)) |
| S133 | (TI (conduct N5 (reduc* or decreas* or increas* or lower* or fewer or improv* or enhance* or extend* or develop* or prevalen* or daily or weekly or current or rate or rates or behavio#r* or knowledge or prevent* or attitude* or avoid* or perceive* or percept* or promot* or program* or change*)) OR (AB (conduct N5 (reduc* or decreas* or increas* or lower* or fewer or improv* or enhance* or extend* or develop* or prevalen* or daily or weekly or current or rate or rates or behavio#r* or knowledge or prevent* or attitude* or avoid* or perceive* or percept* or promot* or program* or change*)) |
| S134 | (TI (respect N5 (reduc* or decreas* or increas* or lower* or fewer or improv* or enhance* or extend* or develop* or prevalen* or daily or weekly or current or rate or rates or behavio#r* or knowledge or prevent* or attitude* or avoid* or perceive* or percept* or promot* or program* or change*)) OR (AB (respect N5 (reduc* or decreas* or increas* or lower* or fewer or improv* or enhance* or extend* or develop* or prevalen* or daily or weekly or current or rate or rates or behavio#r* or knowledge or prevent* or attitude* or avoid* or perceive* or percept* or promot* or program* or change*)) |
| S135 | (TI ((safety or safe) N5 (reduc* or decreas* or increas* or lower* or fewer or improv* or enhance* or extend* or develop* or prevalen* or daily or weekly or current or rate or rates or behavio#r* or knowledge or prevent* or attitude* or avoid* or perceive* or percept* or promot* or program* or change*))) OR (AB ((safety or safe) N5 (reduc* or decreas* or increas* or lower* or fewer or improv* or enhance* or extend* or develop* or prevalen* or daily or weekly or current or rate or rates or behavio#r* or knowledge or prevent* or attitude* or avoid* or perceive* or percept* or promot* or program* or change*))) |
| S136 | S70 OR S71 OR S72 OR S73 OR S74 OR S75 OR S76 OR S77 OR S78 OR S79 OR S80 OR S81 OR S82 OR S83 OR S84 OR S85 OR S86 OR S87 OR S88 OR S89 OR S90 OR S91 OR S92 OR S93 OR S94 OR S95 OR S96 OR S97 OR S98 OR S99 OR S100 OR S101 OR S102 OR S103 OR S104 OR S105 OR S106 OR S107 OR S108 OR S109 OR S110 OR S111 OR S112 OR S113 OR S114 OR S115 OR S116 OR S117 OR S118 OR S119 OR S120 OR S121 OR S122 OR S123 OR S124 OR S125 OR S126 OR S127 OR S128 OR S129 OR S130 OR S131 OR S132 OR S133 OR S134 OR S135 |
| S137 | (TI prevent*) OR (AB prevent*) |
| S138 | (TI program*) OR (AB program*) |
| S139 | (TI intervent*) OR (AB intervent*) |
| S140 | (TI "restorative approach*") OR (AB "restorative approach*") |
| S141 | (TI "control school#") OR (AB "control school#") |
| S142 | (TI (trial or trials)) OR (AB (trial or trials)) |
| S143 | S137 OR S138 OR S139 OR S140 OR S141 OR S142 |
| S144 | S40 AND S69 AND S136 AND S143 |

## ProQuest Education Database

| Database name | Education Database |
| --- | --- |
| Database platform | ProQuest |
| Dates of database coverage | Complete database to date searched. |
| Date searched | 24/01/2020 |
| Searched by | JF |
| Number of results | Alcohol & drugs: 753 Academic attainment: 3957 Violence: 4405 |
| EndNote import order | 17 |
| Number of results once duplicates removed | 2209 |
| Search strategy notes | Due to limitations with the search interface, this search only includes terms for concept 2. These are split into three groups:   1. whole-school interventions which have an impact on levels of violence 2. whole-school interventions which have an impact on substance abuse 3. whole-school interventions which have an impact on educational attainment   * is used for truncation. ? is used for a mandatory wildcard. Search strings starting with TI search the title only. Search strings starting with AB search the abstract only. NEAR/*n* search for terms within *n* words of each other, in any order. PRE/*n* search for terms within *n* words of each other in the order entered. |

**Search for whole school interventions which have an impact on levels of violence**

((ti((school* NEAR/5 (based OR level OR wide OR led OR ethos OR environment OR organi?ation* OR climate OR toolkit OR approach OR action PRE/0 group OR practice OR whole OR health* OR leader OR cultur* OR manag* OR governance OR system* OR communit*)) OR schoolwide OR restor* PRE/0 justice) OR ab((school* NEAR/5 (based OR level OR wide OR led OR ethos OR environment OR organi?ation* OR climate OR toolkit OR approach OR action PRE/0 group OR practice OR whole OR health* OR leader OR cultur* OR manag* OR governance OR system* OR communit*)) OR schoolwide OR restor* PRE/0 justice) OR ((ti("peer-to-peer" OR ((peer OR pupil OR child OR children OR young PRE/0 people OR young PRE/0 person OR teen* OR adolescent* OR youth* OR student OR parent OR teach* OR curricul* OR administrat* OR personnel OR staff OR volunteer OR learn* OR advocacy OR advocate OR environment OR socio-ecolog* OR social PRE/0 support OR policy OR policies) NEAR/2 (led OR leader OR participat* OR mediat* OR helper* OR resistan* OR wide OR action* OR influen*))) OR ab("peer-to-peer" OR ((peer OR pupil OR child OR children OR young PRE/0 people OR young PRE/0 person OR teen* OR adolescent* OR youth* OR student OR parent OR teach* OR curricul* OR administrat* OR personnel OR staff OR volunteer OR learn* OR advocacy OR advocate OR environment OR socio-ecolog* OR social PRE/0 support OR policy OR policies) NEAR/2 (led OR leader OR participat* OR mediat* OR helper* OR resistan* OR wide OR action* OR influen*)))) AND noft(school*))) AND (ti(prevent* OR program* OR intervent* OR restorative PRE/0 approach* OR control PRE/0 school* OR trial OR trials) OR ab(prevent* OR program* OR intervent* OR restorative PRE/0 approach* OR control PRE/0 school* OR trial OR trials)) AND (ti(child OR boy OR girl OR schoolage OR school PRE/0 age OR schoolchild OR youngster OR minor OR pupil OR student OR prepubescent OR pubescent OR adolescent OR juvenile OR underage OR preteen OR pre-teen OR teen OR teens OR teenage* OR youth OR youths OR young PRE/0 person OR young PRE/0 people OR (transition NEAR/4 adult) OR emerging adult OR young adult OR parent OR mother OR father OR guardian OR teacher) OR ab(child OR boy OR girl OR schoolage OR school PRE/0 age OR schoolchild OR youngster OR minor OR pupil OR student OR prepubescent OR pubescent OR adolescent OR juvenile OR underage OR preteen OR pre-teen OR teen OR teens OR teenage* OR youth OR youths OR young PRE/0 person OR young PRE/0 people OR (transition NEAR/4 adult) OR emerging adult OR young adult OR parent OR mother OR father OR guardian OR teacher))) AND (ti(violent OR violence OR aggression OR aggressive OR hostile OR hostility OR assault* OR crime OR abuse* OR abusive OR misbehav* OR threat* OR conduct OR delinquen* OR disrupt* OR ((problem* OR volatile OR positive OR improv* OR social) PRE/0 behavio*) OR antisocial OR "anti-social" OR perpetrat* OR bully* OR victim* OR cyberbully* OR conflict* OR social-emotion* OR socio-emotion* OR social-charact* OR "social-inclusion" OR social PRE/0 develop* OR social PRE/0 competen* OR emotion* PRE/0 develop* OR conduct OR respect OR safety OR safe) OR ab(violent OR violence OR aggression OR aggressive OR hostile OR hostility OR assault* OR crime OR abuse* OR abusive OR misbehav* OR threat* OR conduct OR delinquen* OR disrupt* OR ((problem* OR volatile OR positive OR improv* OR social) PRE/0 behavio*) OR antisocial OR "anti-social" OR perpetrat* OR bully* OR victim* OR cyberbully* OR conflict* OR social-emotion* OR socio-emotion* OR social-charact* OR "social-inclusion" OR social PRE/0 develop* OR social PRE/0 competen* OR emotion* PRE/0 develop* OR conduct OR respect OR safety OR safe))

**Search for whole-school interventions which have an impact on levels of alcohol and drugs**

((ti((school* NEAR/5 (based OR level OR wide OR led OR ethos OR environment OR organi?ation* OR climate OR toolkit OR approach OR action PRE/0 group OR practice OR whole OR health* OR leader OR cultur* OR manag* OR governance OR system* OR communit*)) OR schoolwide OR restor* PRE/0 justice) OR ab((school* NEAR/5 (based OR level OR wide OR led OR ethos OR environment OR organi?ation* OR climate OR toolkit OR approach OR action PRE/0 group OR practice OR whole OR health* OR leader OR cultur* OR manag* OR governance OR system* OR communit*)) OR schoolwide OR restor* PRE/0 justice) OR ((ti("peer-to-peer" OR ((peer OR pupil OR child OR children OR young PRE/0 people OR young PRE/0 person OR teen* OR adolescent* OR youth* OR student OR parent OR teach* OR curricul* OR administrat* OR personnel OR staff OR volunteer OR learn* OR advocacy OR advocate OR environment OR socio-ecolog* OR social PRE/0 support OR policy OR policies) NEAR/2 (led OR leader OR participat* OR mediat* OR helper* OR resistan* OR wide OR action* OR influen*))) OR ab("peer-to-peer" OR ((peer OR pupil OR child OR children OR young PRE/0 people OR young PRE/0 person OR teen* OR adolescent* OR youth* OR student OR parent OR teach* OR curricul* OR administrat* OR personnel OR staff OR volunteer OR learn* OR advocacy OR advocate OR environment OR socio-ecolog* OR social PRE/0 support OR policy OR policies) NEAR/2 (led OR leader OR participat* OR mediat* OR helper* OR resistan* OR wide OR action* OR influen*)))) AND noft(school*))) AND (ti(prevent* OR program* OR intervent* OR restorative PRE/0 approach* OR control PRE/0 school* OR trial OR trials) OR ab(prevent* OR program* OR intervent* OR restorative PRE/0 approach* OR control PRE/0 school* OR trial OR trials)) AND (ti(child OR boy OR girl OR schoolage OR school PRE/0 age OR schoolchild OR youngster OR minor OR pupil OR student OR prepubescent OR pubescent OR adolescent OR juvenile OR underage OR preteen OR pre-teen OR teen OR teens OR teenage* OR youth OR youths OR young PRE/0 person OR young PRE/0 people OR (transition NEAR/4 adult) OR emerging adult OR young adult OR parent OR mother OR father OR guardian OR teacher) OR ab(child OR boy OR girl OR schoolage OR school PRE/0 age OR schoolchild OR youngster OR minor OR pupil OR student OR prepubescent OR pubescent OR adolescent OR juvenile OR underage OR preteen OR pre-teen OR teen OR teens OR teenage* OR youth OR youths OR young PRE/0 person OR young PRE/0 people OR (transition NEAR/4 adult) OR emerging adult OR young adult OR parent OR mother OR father OR guardian OR teacher))) AND (ti(alcohol OR binge PRE/0 drink* OR underage PRE/0 drink* OR substance OR substances OR drug OR drugs) OR ab(alcohol OR binge PRE/0 drink* OR underage PRE/0 drink* OR substance OR substances OR drug OR drugs))

**Search for whole-school interventions which have an impact on levels of educational attainment**

((ti((school* NEAR/5 (based OR level OR wide OR led OR ethos OR environment OR organi?ation* OR climate OR toolkit OR approach OR action PRE/0 group OR practice OR whole OR health* OR leader OR cultur* OR manag* OR governance OR system* OR communit*)) OR schoolwide OR restor* PRE/0 justice) OR ab((school* NEAR/5 (based OR level OR wide OR led OR ethos OR environment OR organi?ation* OR climate OR toolkit OR approach OR action PRE/0 group OR practice OR whole OR health* OR leader OR cultur* OR manag* OR governance OR system* OR communit*)) OR schoolwide OR restor* PRE/0 justice) OR ((ti("peer-to-peer" OR ((peer OR pupil OR child OR children OR young PRE/0 people OR young PRE/0 person OR teen* OR adolescent* OR youth* OR student OR parent OR teach* OR curricul* OR administrat* OR personnel OR staff OR volunteer OR learn* OR advocacy OR advocate OR environment OR socio-ecolog* OR social PRE/0 support OR policy OR policies) NEAR/2 (led OR leader OR participat* OR mediat* OR helper* OR resistan* OR wide OR action* OR influen*))) OR ab("peer-to-peer" OR ((peer OR pupil OR child OR children OR young PRE/0 people OR young PRE/0 person OR teen* OR adolescent* OR youth* OR student OR parent OR teach* OR curricul* OR administrat* OR personnel OR staff OR volunteer OR learn* OR advocacy OR advocate OR environment OR socio-ecolog* OR social PRE/0 support OR policy OR policies) NEAR/2 (led OR leader OR participat* OR mediat* OR helper* OR resistan* OR wide OR action* OR influen*)))) AND noft(school*))) AND (ti(prevent* OR program* OR intervent* OR restorative PRE/0 approach* OR control PRE/0 school* OR trial OR trials) OR ab(prevent* OR program* OR intervent* OR restorative PRE/0 approach* OR control PRE/0 school* OR trial OR trials)) AND (ti(child OR boy OR girl OR schoolage OR school PRE/0 age OR schoolchild OR youngster OR minor OR pupil OR student OR prepubescent OR pubescent OR adolescent OR juvenile OR underage OR preteen OR pre-teen OR teen OR teens OR teenage* OR youth OR youths OR young PRE/0 person OR young PRE/0 people OR (transition NEAR/4 adult) OR emerging adult OR young adult OR parent OR mother OR father OR guardian OR teacher) OR ab(child OR boy OR girl OR schoolage OR school PRE/0 age OR schoolchild OR youngster OR minor OR pupil OR student OR prepubescent OR pubescent OR adolescent OR juvenile OR underage OR preteen OR pre-teen OR teen OR teens OR teenage* OR youth OR youths OR young PRE/0 person OR young PRE/0 people OR (transition NEAR/4 adult) OR emerging adult OR young adult OR parent OR mother OR father OR guardian OR teacher))) AND (ti(((education* OR academic OR student) NEAR/1 (attain* OR achieve* OR perform* OR outcome* OR commitment OR engage*)) OR dropout OR absentee*) OR ab(((education* OR academic OR student) NEAR/1 (attain* OR achieve* OR perform* OR outcome* OR commitment OR engage*)) OR dropout OR absentee*))

## EBSCO Educational Administration Abstracts

| Database name | Educational Administration Abstracts |
| --- | --- |
| Database platform | EBSCO |
| Dates of database coverage | Complete database to date searched. |
| Date searched | 17/01/2020 |
| Searched by | JF |
| Number of results | 1429 |
| EndNote import order | 11 |
| Number of results once duplicates removed | 511 |
| Search strategy notes | * is used for truncation. # is used for an optional wildcard. Search strings starting with TI search the title only. Search strings starting with AB search the abstract only. Search strings starting with SO search the journal title only. N/*n* search for terms within *n* words of each other, in any order. |

| Search number | Query |
| --- | --- |
| S1 | (TI child*) or (AB child*) |
| S2 | (TI boy#) or (AB boy#) |
| S3 | (TI girl#) or (AB girl#) |
| S4 | (TI (schoolage or (school N1 age))) or (AB (schoolage or (school N1 age))) |
| S5 | (TI schoolchild*) or (AB schoolchild*) |
| S6 | (TI youngster#) OR (AB youngster#) |
| S7 | (TI minor#) OR (AB minor#) |
| S8 | (TI pupil*) OR (AB pupil*) |
| S9 | ((TI student*) OR (AB student*)) NOT ((TI "medical student*") or (AB "medical student*")) |
| S10 | (TI prepubescen*) OR (AB prepubescen*) |
| S11 | (TI pubescent*) or (AB pubescent*) |
| S12 | (TI adolescent*) OR (AB adolescent*) |
| S13 | (TI juvenil*) OR (AB juvenil*) |
| S14 | (TI underage*) or (AB underage*) |
| S15 | (TI (preteen* or pre-teen*)) OR (AB (preteen* or pre-teen*)) |
| S16 | (TI (teen or teens)) OR (AB (teen or teens)) |
| S17 | (TI teenage*) OR (AB teenage*) |
| S18 | (TI (youth or youths)) OR (AB (youth or youths)) |
| S19 | (TI "young person*") OR (AB "young person*") |
| S20 | (TI "young people*") OR (AB "young people*") |
| S21 | (TI (kid or kids)) OR (AB (kid or kids)) |
| S22 | (TI (transition n4 adult*)) OR (AB (transition N4 adult*)) |
| S23 | (TI "emerging adult*") OR (AB "emerging adult*") |
| S24 | (TI "young adult*") OR (AB "young adult*") |
| S25 | (TI parent#) OR (AB parent#) |
| S26 | (TI mother#) OR (AB mother#) |
| S27 | (TI father#) or (AB father#) |
| S28 | (TI guardian#) OR (AB guardian#) |
| S29 | (TI teacher#) OR (AB teacher#) |
| S30 | (SO child*) |
| S31 | (SO adolescen*) |
| S32 | (SO youth*) |
| S33 | (SO school*) |
| S34 | (TI ("5" or "6" or "7" or "8" or "9" or "10" or "11" or "12" or "13" or "14" or "15" or "16" or "17" or "18") N1 ("year* old" or "year* of age")) OR (AB ("5" or "6" or "7" or "8" or "9" or "10" or "11" or "12" or "13" or "14" or "15" or "16" or "17" or "18") N1 ("year* old" or "year* of age")) |
| S35 | (TI (five or six or seven or eight or nine or ten or eleven or twelve or thirteen or fourteen or fifteen or sixteen or seventeen or eighteen) N1 ("year* old" or "year* of age")) OR (AB (five or six or seven or eight or nine or ten or eleven or twelve or thirteen or fourteen or fifteen or sixteen or seventeen or eighteen) N1 ("year* old" or "year* of age")) |
| S36 | (TI (age* N1 ("5" or "6" or "7" or "8" or "9" or "10" or "11" or "12" or "13" or "14" or "15" or "16" or "17" or "18") N1 year*)) OR (AB (age* N1 ("5" or "6" or "7" or "8" or "9" or "10" or "11" or "12" or "13" or "14" or "15" or "16" or "17" or "18") N1 year*)) |
| S37 | (TI (age* N1 (five or six or seven or eight or nine or ten or eleven or twelve or thirteen or fourteen or fifteen or sixteen or seventeen or eighteen) N1 year*)) OR (AB (age* N1 (five or six or seven or eight or nine or ten or eleven or twelve or thirteen or fourteen or fifteen or sixteen or seventeen or eighteen) N1 year*)) |
| S38 | (TI (primary or secondary or year) N1 ("1" or "2" or "3" or "4" or "5" or "6" or "7" or "8" or "9" or "10" or "11" or "12" or "13")) OR (AB (primary or secondary or year) N1 ("1" or "2" or "3" or "4" or "5" or "6" or "7" or "8" or "9" or "10" or "11" or "12" or "13")) |
| S39 | (TI grade# N1 (first or second or third or fourth or fifth or sixth or seventh or eighth or ninth or tenth or eleventh or twelfth)) OR (AB grade# N1 (first or second or third or fourth or fifth or sixth or seventh or eighth or ninth or tenth or eleventh or twelfth)) |
| S40 | S1 OR S2 OR S3 OR S4 OR S5 OR S6 OR S7 OR S8 OR S9 OR S10 OR S11 OR S12 OR S13 OR S14 OR S15 OR S16 OR S17 OR S18 OR S19 OR S20 OR S21 OR S22 OR S23 OR S24 OR S25 OR S26 OR S27 OR S28 OR S29 OR S30 OR S31 OR S32 OR S33 OR S34 OR S35 OR S36 OR S37 OR S38 OR S39 |
| S41 | (TI (school# N5 (based or level or wide or led or ethos or environment or organi#ation* or climate or toolkit# or approach* or "action group#" or practice# or whole or health* or leader* or cultur* or manag* or governance or system# or communit*))) OR (AB (school# N5 (based or level or wide or led or ethos or environment or organi#ation* or climate or toolkit# or approach* or "action group#" or practice# or whole or health* or leader* or cultur* or manag* or governance or system# or communit*))) |
| S42 | (TI schoolwide) or (AB schoolwide) |
| S43 | (TI "restor* justice") OR (AB "restor* justice") |
| S44 | (TI school#) or (AB school#) |
| S45 | (TI "peer-to-peer") OR (AB "peer-to-peer") |
| S46 | (TI (peer N2 (led or leader* or participat* or mediat* or helper# or resistan* or wide or action# or influen*))) OR (AB (peer N2 (led or leader* or participat* or mediat* or helper# or resistan* or wide or action# or influen*))) |
| S47 | (TI (relationship* N2 (led or leader* or participat* or mediat* or helper# or resistan* or wide or action# or influen*))) OR (AB (relationship* N2 (led or leader* or participat* or mediat* or helper# or resistan* or wide or action# or influen*))) |
| S48 | (TI ((community or communities) N2 (led or leader* or participat* or mediat* or helper# or resistan* or wide or action# or influen* or relationship*))) OR (AB ((community or communities) N2 (led or leader* or participat* or mediat* or helper# or resistan* or wide or action# or influen* or relationship*))) |
| S49 | ((TI (pupil# N2 (led or leader* or participat* or mediat* or helper# or resistan* or wide or action# or influen* or relationship*)) OR (AB (pupil# N2 (led or leader* or participat* or mediat* or helper# or resistan* or wide or action# or influen* or relationship*))) |
| S50 | (TI ((child or children) N2 (led or leader* or participat* or mediat* or helper# or resistan* or wide or action# or influen*))) OR (AB (child or children*) N2 (led or leader* or participat* or mediat* or helper# or resistan* or wide or action# or influen*))) |
| S51 | (TI (("young people" or "young person") N2 (led or leader* or participat* or mediat* or helper# or resistan* or wide or action# or influen*))) OR (AB (("young people" or "young person") N2 (led or leader* or participat* or mediat* or helper# or resistan* or wide or action# or influen*))) |
| S52 | (TI ((teen or teens or teenage*) N2 (led or leader* or participat* or mediat* or helper# or resistan* or wide or action# or influen*))) OR (AB ((teen or teens or teenage*) N2 (led or leader* or participat* or mediat* or helper# or resistan* or wide or action# or influen*))) |
| S53 | (TI (adolescen* N2 (led or leader* or participat* or mediat* or helper# or resistan* or wide or action# or influen*))) OR (AB (adolescen* N2 (led or leader* or participat* or mediat* or helper# or resistan* or wide or action# or influen*))) |
| S54 | (TI (youth# N2 (led or leader* or participat* or mediat* or helper# or resistan* or wide or action# or influen*))) OR (AB (youth# N2 (led or leader* or participat* or mediat* or helper# or resistan* or wide or action# or influen*))) |
| S55 | (TI (student# N2 (led or leader* or participat* or mediat* or helper# or resistan* or wide or action# or influen* or relationship*))) OR (AB (student# N2 (led or leader* or participat* or mediat* or helper# or resistan* or wide or action# or influen* or relationship*))) |
| S56 | (TI (teach* N2 (led or leader* or participat* or mediat* or helper# or resistan* or wide or action# or influen* or relationship*))) OR (AB (teach* N2 (led or leader* or participat* or mediat* or helper# or resistan* or wide or action# or influen* or relationship*))) |
| S57 | (TI (curricul* N2 (led or leader* or participat* or mediat* or helper# or resistan* or wide or action# or influen* or relationship*))) OR (AB (curricul* N2 (led or leader* or participat* or mediat* or helper# or resistan* or wide or action# or influen* or relationship*))) |
| S58 | (TI (administrat* N2 (led or leader* or participat* or mediat* or helper# or resistan* or wide or action# or influen* or relationship*))) OR (AB (administrat* N2 (led or leader* or participat* or mediat* or helper# or resistan* or wide or action# or influen* or relationship*))) |
| S59 | (TI ((personnel or staff) N2 (led or leader* or participat* or mediat* or helper# or resistan* or wide or action# or influen* or relationship*))) OR (AB ((personnel or staff) N2 (led or leader* or participat* or mediat* or helper# or resistan* or wide or action# or influen* or relationship*))) |
| S60 | (TI (volunteer* N2 (led or leader* or participat* or mediat* or helper# or resistan* or wide or action# or influen* or relationship*))) OR (AB (volulnteer* N2 (led or leader* or participat* or mediat* or helper# or resistan* or wide or action# or influen* or relationship*))) |
| S61 | (TI ((advocacy or advocate) N2 (led or leader* or participat* or mediat* or helper# or resistan* or wide or action# or influen* or relationship*))) OR (AB ((advocacy or advocate) N2 (led or leader* or participat* or mediat* or helper# or resistan* or wide or action# or influen* or relationship*))) |
| S62 | (TI (parent* N2 (led or leader* or participat* or mediat* or helper# or resistan* or wide or action# or influen*))) OR (AB (parent* N2 (led or leader* or participat* or mediat* or helper# or resistan* or wide or action# or influen*))) |
| S63 | (TI (environment* N2 (led or leader* or participat* or mediat* or helper# or resistan* or wide or action# or influen* or relationship*))) OR (AB (environment* N2 (led or leader* or participat* or mediat* or helper# or resistan* or wide or action# or influen* or relationship*))) |
| S64 | (TI ("socio-ecolog*" N2 (led or leader* or participat* or mediat* or helper# or resistan* or wide or action# or influen* or relationship*))) OR (AB ("socio-ecolog*" N2 (led or leader* or participat* or mediat* or helper# or resistan* or wide or action# or influen* or relationship*))) |
| S65 | (TI (learn* N2 (led or leader* or participat* or mediat* or helper# or resistan* or wide or action# or influen* or relationship*))) OR (AB (learn* N2 (led or leader* or participat* or mediat* or helper# or resistan* or wide or action# or influen* or relationship*))) |
| S66 | (TI ((policy or policies) N2 (led or leader* or participat* or mediat* or helper# or resistan* or wide or action# or influen* or relationship*))) OR (AB ((policy or policies) N2 (led or leader* or participat* or mediat* or helper# or resistan* or wide or action# or influen* or relationship*))) |
| S67 | S45 OR S46 OR S47 OR S48 OR S49 OR S50 OR S51 OR S52 OR S53 OR S54 OR S55 OR S56 OR S57 OR S58 OR S59 OR S60 OR S61 OR S62 OR S63 OR S64 OR S65 OR S66 |
| S68 | S44 AND S67 |
| S69 | S41 OR S42 OR S43 OR S68 |
| S70 | (TI ((smoking or smoked or smoke or smoker) N5 (reduc* or decreas* or increas* or lower* or fewer or improv* or enhance* or extend* or develop* or prevalen* or daily or weekly or current or rate or rates or behavio#r* or knowledge or prevent* or attitude* or avoid* or perceive* or percept* or promot* or program* or change*))) OR (AB ((smoking or smoked or smoke or smoker) N5 (reduc* or decreas* or increas* or lower* or fewer or improv* or enhance* or extend* or develop* or prevalen* or daily or weekly or current or rate or rates or behavio#r* or knowledge or prevent* or attitude* or avoid* or perceive* or percept* or promot* or program* or change*))) |
| S71 | (TI (tobacco N5 (reduc* or decreas* or increas* or lower* or fewer or improv* or enhance* or extend* or develop* or prevalen* or daily or weekly or current or rate or rates or behavio#r* or knowledge or prevent* or attitude* or avoid* or perceive* or percept* or promot* or program* or change*)) OR (AB (tobacco N5 (reduc* or decreas* or increas* or lower* or fewer or improv* or enhance* or extend* or develop* or prevalen* or daily or weekly or current or rate or rates or behavio#r* or knowledge or prevent* or attitude* or avoid* or perceive* or percept* or promot* or program* or change*)) |
| S72 | (TI (cigarette# N5 (reduc* or decreas* or increas* or lower* or fewer or improv* or enhance* or extend* or develop* or prevalen* or daily or weekly or current or rate or rates or behavio#r* or knowledge or prevent* or attitude* or avoid* or perceive* or percept* or promot* or program* or change*)) OR (AB (cigarette# N5 (reduc* or decreas* or increas* or lower* or fewer or improv* or enhance* or extend* or develop* or prevalen* or daily or weekly or current or rate or rates or behavio#r* or knowledge or prevent* or attitude* or avoid* or perceive* or percept* or promot* or program* or change*)) |
| S73 | (TI (marijuana N5 (reduc* or decreas* or increas* or lower* or fewer or improv* or enhance* or extend* or develop* or prevalen* or daily or weekly or current or rate or rates or behavio#r* or knowledge or prevent* or attitude* or avoid* or perceive* or percept* or promot* or program* or change*)) OR (AB (marijuana N5 (reduc* or decreas* or increas* or lower* or fewer or improv* or enhance* or extend* or develop* or prevalen* or daily or weekly or current or rate or rates or behavio#r* or knowledge or prevent* or attitude* or avoid* or perceive* or percept* or promot* or program* or change*)) |
| S74 | (TI (cannabis N5 (reduc* or decreas* or increas* or lower* or fewer or improv* or enhance* or extend* or develop* or prevalen* or daily or weekly or current or rate or rates or behavio#r* or knowledge or prevent* or attitude* or avoid* or perceive* or percept* or promot* or program* or change*)) OR (AB (cannabis N5 (reduc* or decreas* or increas* or lower* or fewer or improv* or enhance* or extend* or develop* or prevalen* or daily or weekly or current or rate or rates or behavio#r* or knowledge or prevent* or attitude* or avoid* or perceive* or percept* or promot* or program* or change*)) |
| S75 | (TI (solvent# N5 (reduc* or decreas* or increas* or lower* or fewer or improv* or enhance* or extend* or develop* or prevalen* or daily or weekly or current or rate or rates or behavio#r* or knowledge or prevent* or attitude* or avoid* or perceive* or percept* or promot* or program* or change*)) OR (AB (solvent# N5 (reduc* or decreas* or increas* or lower* or fewer or improv* or enhance* or extend* or develop* or prevalen* or daily or weekly or current or rate or rates or behavio#r* or knowledge or prevent* or attitude* or avoid* or perceive* or percept* or promot* or program* or change*)) |
| S76 | (TI (alcohol N5 (reduc* or decreas* or increas* or lower* or fewer or improv* or enhance* or extend* or develop* or prevalen* or daily or weekly or current or rate or rates or behavio#r* or knowledge or prevent* or attitude* or avoid* or perceive* or percept* or promot* or program* or change*)) OR (AB (alcohol N5 (reduc* or decreas* or increas* or lower* or fewer or improv* or enhance* or extend* or develop* or prevalen* or daily or weekly or current or rate or rates or behavio#r* or knowledge or prevent* or attitude* or avoid* or perceive* or percept* or promot* or program* or change*)) |
| S77 | (TI ("binge drink*" N5 (reduc* or decreas* or increas* or lower* or fewer or improv* or enhance* or extend* or develop* or prevalen* or daily or weekly or current or rate or rates or behavio#r* or knowledge or prevent* or attitude* or avoid* or perceive* or percept* or promot* or program* or change*)) OR (AB ("binge drink*" N5 (reduc* or decreas* or increas* or lower* or fewer or improv* or enhance* or extend* or develop* or prevalen* or daily or weekly or current or rate or rates or behavio#r* or knowledge or prevent* or attitude* or avoid* or perceive* or percept* or promot* or program* or change*)) |
| S78 | (TI ("underage drink*" N5 (reduc* or decreas* or increas* or lower* or fewer or improv* or enhance* or extend* or develop* or prevalen* or daily or weekly or current or rate or rates or behavio#r* or knowledge or prevent* or attitude* or avoid* or perceive* or percept* or promot* or program* or change*)) OR (AB ("underage drink*" N5 (reduc* or decreas* or increas* or lower* or fewer or improv* or enhance* or extend* or develop* or prevalen* or daily or weekly or current or rate or rates or behavio#r* or knowledge or prevent* or attitude* or avoid* or perceive* or percept* or promot* or program* or change*)) |
| S79 | (TI ("underage drink*" N5 (reduc* or decreas* or increas* or lower* or fewer or improv* or enhance* or extend* or develop* or prevalen* or daily or weekly or current or rate or rates or behavio#r* or knowledge or prevent* or attitude* or avoid* or perceive* or percept* or promot* or program* or change*)) OR (AB ("underage drink*" N5 (reduc* or decreas* or increas* or lower* or fewer or improv* or enhance* or extend* or develop* or prevalen* or daily or weekly or current or rate or rates or behavio#r* or knowledge or prevent* or attitude* or avoid* or perceive* or percept* or promot* or program* or change*)) |
| S80 | (TI (substance# N5 (reduc* or decreas* or increas* or lower* or fewer or improv* or enhance* or extend* or develop* or prevalen* or daily or weekly or current or rate or rates or behavio#r* or knowledge or prevent* or attitude* or avoid* or perceive* or percept* or promot* or program* or change*)) OR (AB (substance# N5 (reduc* or decreas* or increas* or lower* or fewer or improv* or enhance* or extend* or develop* or prevalen* or daily or weekly or current or rate or rates or behavio#r* or knowledge or prevent* or attitude* or avoid* or perceive* or percept* or promot* or program* or change*)) |
| S81 | (TI (drug# N5 (reduc* or decreas* or increas* or lower* or fewer or improv* or enhance* or extend* or develop* or prevalen* or daily or weekly or current or rate or rates or behavio#r* or knowledge or prevent* or attitude* or avoid* or perceive* or percept* or promot* or program* or change*)) OR (AB (drug# N5 (reduc* or decreas* or increas* or lower* or fewer or improv* or enhance* or extend* or develop* or prevalen* or daily or weekly or current or rate or rates or behavio#r* or knowledge or prevent* or attitude* or avoid* or perceive* or percept* or promot* or program* or change*)) |
| S82 | (TI ("education* attain*" N5 (reduc* or decreas* or increas* or lower* or fewer or improv* or enhance* or extend* or develop* or prevalen* or daily or weekly or current or rate or rates or behavio#r* or knowledge or prevent* or attitude* or avoid* or perceive* or percept* or promot* or program* or change*)) OR (AB ("education* attain*" N5 (reduc* or decreas* or increas* or lower* or fewer or improv* or enhance* or extend* or develop* or prevalen* or daily or weekly or current or rate or rates or behavio#r* or knowledge or prevent* or attitude* or avoid* or perceive* or percept* or promot* or program* or change*)) |
| S83 | (TI ("academic attain*" N5 (reduc* or decreas* or increas* or lower* or fewer or improv* or enhance* or extend* or develop* or prevalen* or daily or weekly or current or rate or rates or behavio#r* or knowledge or prevent* or attitude* or avoid* or perceive* or percept* or promot* or program* or change*)) OR (AB ("academic attain*" N5 (reduc* or decreas* or increas* or lower* or fewer or improv* or enhance* or extend* or develop* or prevalen* or daily or weekly or current or rate or rates or behavio#r* or knowledge or prevent* or attitude* or avoid* or perceive* or percept* or promot* or program* or change*)) |
| S84 | (TI ("student* attain*" N5 (reduc* or decreas* or increas* or lower* or fewer or improv* or enhance* or extend* or develop* or prevalen* or daily or weekly or current or rate or rates or behavio#r* or knowledge or prevent* or attitude* or avoid* or perceive* or percept* or promot* or program* or change*)) OR (AB ("student* attain*" N5 (reduc* or decreas* or increas* or lower* or fewer or improv* or enhance* or extend* or develop* or prevalen* or daily or weekly or current or rate or rates or behavio#r* or knowledge or prevent* or attitude* or avoid* or perceive* or percept* or promot* or program* or change*)) |
| S85 | (TI ("education* achieve*" N5 (reduc* or decreas* or increas* or lower* or fewer or improv* or enhance* or extend* or develop* or prevalen* or daily or weekly or current or rate or rates or behavio#r* or knowledge or prevent* or attitude* or avoid* or perceive* or percept* or promot* or program* or change*)) OR (AB ("education* achieve*" N5 (reduc* or decreas* or increas* or lower* or fewer or improv* or enhance* or extend* or develop* or prevalen* or daily or weekly or current or rate or rates or behavio#r* or knowledge or prevent* or attitude* or avoid* or perceive* or percept* or promot* or program* or change*)) |
| S86 | (TI ("academic achieve*" N5 (reduc* or decreas* or increas* or lower* or fewer or improv* or enhance* or extend* or develop* or prevalen* or daily or weekly or current or rate or rates or behavio#r* or knowledge or prevent* or attitude* or avoid* or perceive* or percept* or promot* or program* or change*)) OR (AB ("academic achieve*" N5 (reduc* or decreas* or increas* or lower* or fewer or improv* or enhance* or extend* or develop* or prevalen* or daily or weekly or current or rate or rates or behavio#r* or knowledge or prevent* or attitude* or avoid* or perceive* or percept* or promot* or program* or change*)) |
| S87 | (TI ("student* achieve*" N5 (reduc* or decreas* or increas* or lower* or fewer or improv* or enhance* or extend* or develop* or prevalen* or daily or weekly or current or rate or rates or behavio#r* or knowledge or prevent* or attitude* or avoid* or perceive* or percept* or promot* or program* or change*)) OR (AB ("student* achieve*" N5 (reduc* or decreas* or increas* or lower* or fewer or improv* or enhance* or extend* or develop* or prevalen* or daily or weekly or current or rate or rates or behavio#r* or knowledge or prevent* or attitude* or avoid* or perceive* or percept* or promot* or program* or change*)) |
| S88 | (TI ("education* perform*" N5 (reduc* or decreas* or increas* or lower* or fewer or improv* or enhance* or extend* or develop* or prevalen* or daily or weekly or current or rate or rates or behavio#r* or knowledge or prevent* or attitude* or avoid* or perceive* or percept* or promot* or program* or change*)) OR (AB ("education* perform*" N5 (reduc* or decreas* or increas* or lower* or fewer or improv* or enhance* or extend* or develop* or prevalen* or daily or weekly or current or rate or rates or behavio#r* or knowledge or prevent* or attitude* or avoid* or perceive* or percept* or promot* or program* or change*)) |
| S89 | (TI ("academic perform*" N5 (reduc* or decreas* or increas* or lower* or fewer or improv* or enhance* or extend* or develop* or prevalen* or daily or weekly or current or rate or rates or behavio#r* or knowledge or prevent* or attitude* or avoid* or perceive* or percept* or promot* or program* or change*)) OR (AB ("academic perform*" N5 (reduc* or decreas* or increas* or lower* or fewer or improv* or enhance* or extend* or develop* or prevalen* or daily or weekly or current or rate or rates or behavio#r* or knowledge or prevent* or attitude* or avoid* or perceive* or percept* or promot* or program* or change*)) |
| S90 | (TI ("student* perform*" N5 (reduc* or decreas* or increas* or lower* or fewer or improv* or enhance* or extend* or develop* or prevalen* or daily or weekly or current or rate or rates or behavio#r* or knowledge or prevent* or attitude* or avoid* or perceive* or percept* or promot* or program* or change*)) OR (AB ("student* perform*" N5 (reduc* or decreas* or increas* or lower* or fewer or improv* or enhance* or extend* or develop* or prevalen* or daily or weekly or current or rate or rates or behavio#r* or knowledge or prevent* or attitude* or avoid* or perceive* or percept* or promot* or program* or change*)) |
| S91 | (TI ("education* outcome#" N5 (reduc* or decreas* or increas* or lower* or fewer or improv* or enhance* or extend* or develop* or prevalen* or daily or weekly or current or rate or rates or behavio#r* or knowledge or prevent* or attitude* or avoid* or perceive* or percept* or promot* or program* or change*)) OR (AB ("education* outcome#" N5 (reduc* or decreas* or increas* or lower* or fewer or improv* or enhance* or extend* or develop* or prevalen* or daily or weekly or current or rate or rates or behavio#r* or knowledge or prevent* or attitude* or avoid* or perceive* or percept* or promot* or program* or change*)) |
| S92 | (TI ("academic outcome#" N5 (reduc* or decreas* or increas* or lower* or fewer or improv* or enhance* or extend* or develop* or prevalen* or daily or weekly or current or rate or rates or behavio#r* or knowledge or prevent* or attitude* or avoid* or perceive* or percept* or promot* or program* or change*)) OR (AB ("academic outcome#" N5 (reduc* or decreas* or increas* or lower* or fewer or improv* or enhance* or extend* or develop* or prevalen* or daily or weekly or current or rate or rates or behavio#r* or knowledge or prevent* or attitude* or avoid* or perceive* or percept* or promot* or program* or change*)) |
| S93 | (TI ("student* outcome#" N5 (reduc* or decreas* or increas* or lower* or fewer or improv* or enhance* or extend* or develop* or prevalen* or daily or weekly or current or rate or rates or behavio#r* or knowledge or prevent* or attitude* or avoid* or perceive* or percept* or promot* or program* or change*)) OR (AB ("student* outcome#" N5 (reduc* or decreas* or increas* or lower* or fewer or improv* or enhance* or extend* or develop* or prevalen* or daily or weekly or current or rate or rates or behavio#r* or knowledge or prevent* or attitude* or avoid* or perceive* or percept* or promot* or program* or change*)) |
| S94 | (TI ("education* commitment" N5 (reduc* or decreas* or increas* or lower* or fewer or improv* or enhance* or extend* or develop* or prevalen* or daily or weekly or current or rate or rates or behavio#r* or knowledge or prevent* or attitude* or avoid* or perceive* or percept* or promot* or program* or change*)) OR (AB ("education* commitment" N5 (reduc* or decreas* or increas* or lower* or fewer or improv* or enhance* or extend* or develop* or prevalen* or daily or weekly or current or rate or rates or behavio#r* or knowledge or prevent* or attitude* or avoid* or perceive* or percept* or promot* or program* or change*)) |
| S95 | (TI ("education* commitment" N5 (reduc* or decreas* or increas* or lower* or fewer or improv* or enhance* or extend* or develop* or prevalen* or daily or weekly or current or rate or rates or behavio#r* or knowledge or prevent* or attitude* or avoid* or perceive* or percept* or promot* or program* or change*)) OR (AB ("education* commitment" N5 (reduc* or decreas* or increas* or lower* or fewer or improv* or enhance* or extend* or develop* or prevalen* or daily or weekly or current or rate or rates or behavio#r* or knowledge or prevent* or attitude* or avoid* or perceive* or percept* or promot* or program* or change*)) |
| S96 | (TI ("academic commitment" N5 (reduc* or decreas* or increas* or lower* or fewer or improv* or enhance* or extend* or develop* or prevalen* or daily or weekly or current or rate or rates or behavio#r* or knowledge or prevent* or attitude* or avoid* or perceive* or percept* or promot* or program* or change*)) OR (AB ("academic commitment" N5 (reduc* or decreas* or increas* or lower* or fewer or improv* or enhance* or extend* or develop* or prevalen* or daily or weekly or current or rate or rates or behavio#r* or knowledge or prevent* or attitude* or avoid* or perceive* or percept* or promot* or program* or change*)) |
| S97 | (TI ("academic commitment" N5 (reduc* or decreas* or increas* or lower* or fewer or improv* or enhance* or extend* or develop* or prevalen* or daily or weekly or current or rate or rates or behavio#r* or knowledge or prevent* or attitude* or avoid* or perceive* or percept* or promot* or program* or change*)) OR (AB ("academic commitment" N5 (reduc* or decreas* or increas* or lower* or fewer or improv* or enhance* or extend* or develop* or prevalen* or daily or weekly or current or rate or rates or behavio#r* or knowledge or prevent* or attitude* or avoid* or perceive* or percept* or promot* or program* or change*)) |
| S98 | (TI ("student* commitment" N5 (reduc* or decreas* or increas* or lower* or fewer or improv* or enhance* or extend* or develop* or prevalen* or daily or weekly or current or rate or rates or behavio#r* or knowledge or prevent* or attitude* or avoid* or perceive* or percept* or promot* or program* or change*)) OR (AB ("student* commitment" N5 (reduc* or decreas* or increas* or lower* or fewer or improv* or enhance* or extend* or develop* or prevalen* or daily or weekly or current or rate or rates or behavio#r* or knowledge or prevent* or attitude* or avoid* or perceive* or percept* or promot* or program* or change*)) |
| S99 | (TI ("education* engage*" N5 (reduc* or decreas* or increas* or lower* or fewer or improv* or enhance* or extend* or develop* or prevalen* or daily or weekly or current or rate or rates or behavio#r* or knowledge or prevent* or attitude* or avoid* or perceive* or percept* or promot* or program* or change*)) OR (AB ("education* engage*" N5 (reduc* or decreas* or increas* or lower* or fewer or improv* or enhance* or extend* or develop* or prevalen* or daily or weekly or current or rate or rates or behavio#r* or knowledge or prevent* or attitude* or avoid* or perceive* or percept* or promot* or program* or change*)) |
| S100 | (TI ("academic engage*" N5 (reduc* or decreas* or increas* or lower* or fewer or improv* or enhance* or extend* or develop* or prevalen* or daily or weekly or current or rate or rates or behavio#r* or knowledge or prevent* or attitude* or avoid* or perceive* or percept* or promot* or program* or change*)) OR (AB ("academic engage*" N5 (reduc* or decreas* or increas* or lower* or fewer or improv* or enhance* or extend* or develop* or prevalen* or daily or weekly or current or rate or rates or behavio#r* or knowledge or prevent* or attitude* or avoid* or perceive* or percept* or promot* or program* or change*)) |
| S101 | (TI ("student* engage*" N5 (reduc* or decreas* or increas* or lower* or fewer or improv* or enhance* or extend* or develop* or prevalen* or daily or weekly or current or rate or rates or behavio#r* or knowledge or prevent* or attitude* or avoid* or perceive* or percept* or promot* or program* or change*)) OR (AB ("student* engage*" N5 (reduc* or decreas* or increas* or lower* or fewer or improv* or enhance* or extend* or develop* or prevalen* or daily or weekly or current or rate or rates or behavio#r* or knowledge or prevent* or attitude* or avoid* or perceive* or percept* or promot* or program* or change*)) |
| S102 | (TI (dropout N5 (reduc* or decreas* or increas* or lower* or fewer or improv* or enhance* or extend* or develop* or prevalen* or daily or weekly or current or rate or rates or behavio#r* or knowledge or prevent* or attitude* or avoid* or perceive* or percept* or promot* or program* or change*)) OR (AB (dropout N5 (reduc* or decreas* or increas* or lower* or fewer or improv* or enhance* or extend* or develop* or prevalen* or daily or weekly or current or rate or rates or behavio#r* or knowledge or prevent* or attitude* or avoid* or perceive* or percept* or promot* or program* or change*)) |
| S103 | (TI (absentee* N5 (reduc* or decreas* or increas* or lower* or fewer or improv* or enhance* or extend* or develop* or prevalen* or daily or weekly or current or rate or rates or behavio#r* or knowledge or prevent* or attitude* or avoid* or perceive* or percept* or promot* or program* or change*)) OR (AB (absentee* N5 (reduc* or decreas* or increas* or lower* or fewer or improv* or enhance* or extend* or develop* or prevalen* or daily or weekly or current or rate or rates or behavio#r* or knowledge or prevent* or attitude* or avoid* or perceive* or percept* or promot* or program* or change*)) |
| S104 | (TI ((violent or violence) N5 (reduc* or decreas* or increas* or lower* or fewer or improv* or enhance* or extend* or develop* or prevalen* or daily or weekly or current or rate or rates or behavio#r* or knowledge or prevent* or attitude* or avoid* or perceive* or percept* or promot* or program* or change*))) OR (AB ((violent or violence) N5 (reduc* or decreas* or increas* or lower* or fewer or improv* or enhance* or extend* or develop* or prevalen* or daily or weekly or current or rate or rates or behavio#r* or knowledge or prevent* or attitude* or avoid* or perceive* or percept* or promot* or program* or change*))) |
| S105 | (TI ((aggression or aggressive) N5 (reduc* or decreas* or increas* or lower* or fewer or improv* or enhance* or extend* or develop* or prevalen* or daily or weekly or current or rate or rates or behavio#r* or knowledge or prevent* or attitude* or avoid* or perceive* or percept* or promot* or program* or change*))) OR (AB ((aggression or aggressive) N5 (reduc* or decreas* or increas* or lower* or fewer or improv* or enhance* or extend* or develop* or prevalen* or daily or weekly or current or rate or rates or behavio#r* or knowledge or prevent* or attitude* or avoid* or perceive* or percept* or promot* or program* or change*))) |
| S106 | (TI ((hostile or hostility) N5 (reduc* or decreas* or increas* or lower* or fewer or improv* or enhance* or extend* or develop* or prevalen* or daily or weekly or current or rate or rates or behavio#r* or knowledge or prevent* or attitude* or avoid* or perceive* or percept* or promot* or program* or change*))) OR (AB ((hostile or hostility) N5 (reduc* or decreas* or increas* or lower* or fewer or improv* or enhance* or extend* or develop* or prevalen* or daily or weekly or current or rate or rates or behavio#r* or knowledge or prevent* or attitude* or avoid* or perceive* or percept* or promot* or program* or change*))) |
| S107 | (TI (assault* N5 (reduc* or decreas* or increas* or lower* or fewer or improv* or enhance* or extend* or develop* or prevalen* or daily or weekly or current or rate or rates or behavio#r* or knowledge or prevent* or attitude* or avoid* or perceive* or percept* or promot* or program* or change*)) OR (AB (assault* N5 (reduc* or decreas* or increas* or lower* or fewer or improv* or enhance* or extend* or develop* or prevalen* or daily or weekly or current or rate or rates or behavio#r* or knowledge or prevent* or attitude* or avoid* or perceive* or percept* or promot* or program* or change*)) |
| S108 | (TI (crime N5 (reduc* or decreas* or increas* or lower* or fewer or improv* or enhance* or extend* or develop* or prevalen* or daily or weekly or current or rate or rates or behavio#r* or knowledge or prevent* or attitude* or avoid* or perceive* or percept* or promot* or program* or change*)) OR (AB (crime N5 (reduc* or decreas* or increas* or lower* or fewer or improv* or enhance* or extend* or develop* or prevalen* or daily or weekly or current or rate or rates or behavio#r* or knowledge or prevent* or attitude* or avoid* or perceive* or percept* or promot* or program* or change*)) |
| S109 | (TI ((abuse# or abusive) N5 (reduc* or decreas* or increas* or lower* or fewer or improv* or enhance* or extend* or develop* or prevalen* or daily or weekly or current or rate or rates or behavio#r* or knowledge or prevent* or attitude* or avoid* or perceive* or percept* or promot* or program* or change*))) OR (AB ((abuse# or abusive) N5 (reduc* or decreas* or increas* or lower* or fewer or improv* or enhance* or extend* or develop* or prevalen* or daily or weekly or current or rate or rates or behavio#r* or knowledge or prevent* or attitude* or avoid* or perceive* or percept* or promot* or program* or change*))) |
| S110 | (TI (misbehav* N5 (reduc* or decreas* or increas* or lower* or fewer or improv* or enhance* or extend* or develop* or prevalen* or daily or weekly or current or rate or rates or behavio#r* or knowledge or prevent* or attitude* or avoid* or perceive* or percept* or promot* or program* or change*)) OR (AB (misbehav* N5 (reduc* or decreas* or increas* or lower* or fewer or improv* or enhance* or extend* or develop* or prevalen* or daily or weekly or current or rate or rates or behavio#r* or knowledge or prevent* or attitude* or avoid* or perceive* or percept* or promot* or program* or change*)) |
| S111 | (TI (threat# N5 (reduc* or decreas* or increas* or lower* or fewer or improv* or enhance* or extend* or develop* or prevalen* or daily or weekly or current or rate or rates or behavio#r* or knowledge or prevent* or attitude* or avoid* or perceive* or percept* or promot* or program* or change*)) OR (AB (threat# N5 (reduc* or decreas* or increas* or lower* or fewer or improv* or enhance* or extend* or develop* or prevalen* or daily or weekly or current or rate or rates or behavio#r* or knowledge or prevent* or attitude* or avoid* or perceive* or percept* or promot* or program* or change*)) |
| S112 | (TI (conduct N5 (reduc* or decreas* or increas* or lower* or fewer or improv* or enhance* or extend* or develop* or prevalen* or daily or weekly or current or rate or rates or behavio#r* or knowledge or prevent* or attitude* or avoid* or perceive* or percept* or promot* or program* or change*)) OR (AB (conduct N5 (reduc* or decreas* or increas* or lower* or fewer or improv* or enhance* or extend* or develop* or prevalen* or daily or weekly or current or rate or rates or behavio#r* or knowledge or prevent* or attitude* or avoid* or perceive* or percept* or promot* or program* or change*)) |
| S113 | (TI (delinquen* N5 (reduc* or decreas* or increas* or lower* or fewer or improv* or enhance* or extend* or develop* or prevalen* or daily or weekly or current or rate or rates or behavio#r* or knowledge or prevent* or attitude* or avoid* or perceive* or percept* or promot* or program* or change*)) OR (AB (delinquen* N5 (reduc* or decreas* or increas* or lower* or fewer or improv* or enhance* or extend* or develop* or prevalen* or daily or weekly or current or rate or rates or behavio#r* or knowledge or prevent* or attitude* or avoid* or perceive* or percept* or promot* or program* or change*)) |
| S114 | (TI ("disrupt* behavio#r*" N5 (reduc* or decreas* or increas* or lower* or fewer or improv* or enhance* or extend* or develop* or prevalen* or daily or weekly or current or rate or rates or behavio#r* or knowledge or prevent* or attitude* or avoid* or perceive* or percept* or promot* or program* or change*)) OR (AB ("disrupt* behavio#r*" N5 (reduc* or decreas* or increas* or lower* or fewer or improv* or enhance* or extend* or develop* or prevalen* or daily or weekly or current or rate or rates or behavio#r* or knowledge or prevent* or attitude* or avoid* or perceive* or percept* or promot* or program* or change*)) |
| S115 | (TI ("problem* behavio#r*" N5 (reduc* or decreas* or increas* or lower* or fewer or improv* or enhance* or extend* or develop* or prevalen* or daily or weekly or current or rate or rates or behavio#r* or knowledge or prevent* or attitude* or avoid* or perceive* or percept* or promot* or program* or change*)) OR (AB ("problem* behavio#r*" N5 (reduc* or decreas* or increas* or lower* or fewer or improv* or enhance* or extend* or develop* or prevalen* or daily or weekly or current or rate or rates or behavio#r* or knowledge or prevent* or attitude* or avoid* or perceive* or percept* or promot* or program* or change*)) |
| S116 | (TI ("volatile behavio#r*" N5 (reduc* or decreas* or increas* or lower* or fewer or improv* or enhance* or extend* or develop* or prevalen* or daily or weekly or current or rate or rates or behavio#r* or knowledge or prevent* or attitude* or avoid* or perceive* or percept* or promot* or program* or change*)) OR (AB ("volatile behavio#r*" N5 (reduc* or decreas* or increas* or lower* or fewer or improv* or enhance* or extend* or develop* or prevalen* or daily or weekly or current or rate or rates or behavio#r* or knowledge or prevent* or attitude* or avoid* or perceive* or percept* or promot* or program* or change*)) |
| S117 | (TI ("volatile behavio#r*" N5 (reduc* or decreas* or increas* or lower* or fewer or improv* or enhance* or extend* or develop* or prevalen* or daily or weekly or current or rate or rates or behavio#r* or knowledge or prevent* or attitude* or avoid* or perceive* or percept* or promot* or program* or change*)) OR (AB ("volatile behavio#r*" N5 (reduc* or decreas* or increas* or lower* or fewer or improv* or enhance* or extend* or develop* or prevalen* or daily or weekly or current or rate or rates or behavio#r* or knowledge or prevent* or attitude* or avoid* or perceive* or percept* or promot* or program* or change*)) |
| S118 | (TI ((antisocial or "anti-social") N5 (reduc* or decreas* or increas* or lower* or fewer or improv* or enhance* or extend* or develop* or prevalen* or daily or weekly or current or rate or rates or behavio#r* or knowledge or prevent* or attitude* or avoid* or perceive* or percept* or promot* or program* or change*))) OR (AB ((antisocial or "anti-social") N5 (reduc* or decreas* or increas* or lower* or fewer or improv* or enhance* or extend* or develop* or prevalen* or daily or weekly or current or rate or rates or behavio#r* or knowledge or prevent* or attitude* or avoid* or perceive* or percept* or promot* or program* or change*))) |
| S119 | (TI (perpetrat* N5 (reduc* or decreas* or increas* or lower* or fewer or improv* or enhance* or extend* or develop* or prevalen* or daily or weekly or current or rate or rates or behavio#r* or knowledge or prevent* or attitude* or avoid* or perceive* or percept* or promot* or program* or change*)) OR (AB (perpetrat* N5 (reduc* or decreas* or increas* or lower* or fewer or improv* or enhance* or extend* or develop* or prevalen* or daily or weekly or current or rate or rates or behavio#r* or knowledge or prevent* or attitude* or avoid* or perceive* or percept* or promot* or program* or change*)) |
| S120 | (TI (bully* N5 (reduc* or decreas* or increas* or lower* or fewer or improv* or enhance* or extend* or develop* or prevalen* or daily or weekly or current or rate or rates or behavio#r* or knowledge or prevent* or attitude* or avoid* or perceive* or percept* or promot* or program* or change*)) OR (AB (bully* N5 (reduc* or decreas* or increas* or lower* or fewer or improv* or enhance* or extend* or develop* or prevalen* or daily or weekly or current or rate or rates or behavio#r* or knowledge or prevent* or attitude* or avoid* or perceive* or percept* or promot* or program* or change*)) |
| S121 | (TI (victim* N5 (reduc* or decreas* or increas* or lower* or fewer or improv* or enhance* or extend* or develop* or prevalen* or daily or weekly or current or rate or rates or behavio#r* or knowledge or prevent* or attitude* or avoid* or perceive* or percept* or promot* or program* or change*)) OR (AB (victim* N5 (reduc* or decreas* or increas* or lower* or fewer or improv* or enhance* or extend* or develop* or prevalen* or daily or weekly or current or rate or rates or behavio#r* or knowledge or prevent* or attitude* or avoid* or perceive* or percept* or promot* or program* or change*)) |
| S122 | (TI (cyberbully* N5 (reduc* or decreas* or increas* or lower* or fewer or improv* or enhance* or extend* or develop* or prevalen* or daily or weekly or current or rate or rates or behavio#r* or knowledge or prevent* or attitude* or avoid* or perceive* or percept* or promot* or program* or change*)) OR (AB (cyberbully* N5 (reduc* or decreas* or increas* or lower* or fewer or improv* or enhance* or extend* or develop* or prevalen* or daily or weekly or current or rate or rates or behavio#r* or knowledge or prevent* or attitude* or avoid* or perceive* or percept* or promot* or program* or change*)) |
| S123 | (TI (conflict* N5 (reduc* or decreas* or increas* or lower* or fewer or improv* or enhance* or extend* or develop* or prevalen* or daily or weekly or current or rate or rates or behavio#r* or knowledge or prevent* or attitude* or avoid* or perceive* or percept* or promot* or program* or change*)) OR (AB (conflict* N5 (reduc* or decreas* or increas* or lower* or fewer or improv* or enhance* or extend* or develop* or prevalen* or daily or weekly or current or rate or rates or behavio#r* or knowledge or prevent* or attitude* or avoid* or perceive* or percept* or promot* or program* or change*)) |
| S124 | (TI ("positive behavio#r*" N5 (reduc* or decreas* or increas* or lower* or fewer or improv* or enhance* or extend* or develop* or prevalen* or daily or weekly or current or rate or rates or behavio#r* or knowledge or prevent* or attitude* or avoid* or perceive* or percept* or promot* or program* or change*)) OR (AB ("positive behavio#r*" N5 (reduc* or decreas* or increas* or lower* or fewer or improv* or enhance* or extend* or develop* or prevalen* or daily or weekly or current or rate or rates or behavio#r* or knowledge or prevent* or attitude* or avoid* or perceive* or percept* or promot* or program* or change*)) |
| S125 | (TI ("improv* behavio#r*" N5 (reduc* or decreas* or increas* or lower* or fewer or improv* or enhance* or extend* or develop* or prevalen* or daily or weekly or current or rate or rates or behavio#r* or knowledge or prevent* or attitude* or avoid* or perceive* or percept* or promot* or program* or change*)) OR (AB ("improv* behavio#r*" N5 (reduc* or decreas* or increas* or lower* or fewer or improv* or enhance* or extend* or develop* or prevalen* or daily or weekly or current or rate or rates or behavio#r* or knowledge or prevent* or attitude* or avoid* or perceive* or percept* or promot* or program* or change*)) |
| S126 | (TI ("social behavio#r*" N5 (reduc* or decreas* or increas* or lower* or fewer or improv* or enhance* or extend* or develop* or prevalen* or daily or weekly or current or rate or rates or behavio#r* or knowledge or prevent* or attitude* or avoid* or perceive* or percept* or promot* or program* or change*)) OR (AB ("social behavio#r*" N5 (reduc* or decreas* or increas* or lower* or fewer or improv* or enhance* or extend* or develop* or prevalen* or daily or weekly or current or rate or rates or behavio#r* or knowledge or prevent* or attitude* or avoid* or perceive* or percept* or promot* or program* or change*)) |
| S127 | (TI (("social-emotion*" or "socio-emotion*") N5 (reduc* or decreas* or increas* or lower* or fewer or improv* or enhance* or extend* or develop* or prevalen* or daily or weekly or current or rate or rates or behavio#r* or knowledge or prevent* or attitude* or avoid* or perceive* or percept* or promot* or program* or change*))) OR (AB (("social-emotion*" or "socio-emotion*") N5 (reduc* or decreas* or increas* or lower* or fewer or improv* or enhance* or extend* or develop* or prevalen* or daily or weekly or current or rate or rates or behavio#r* or knowledge or prevent* or attitude* or avoid* or perceive* or percept* or promot* or program* or change*))) |
| S128 | (TI ("social-charact*" N5 (reduc* or decreas* or increas* or lower* or fewer or improv* or enhance* or extend* or develop* or prevalen* or daily or weekly or current or rate or rates or behavio#r* or knowledge or prevent* or attitude* or avoid* or perceive* or percept* or promot* or program* or change*)) OR (AB ("social-charact*" N5 (reduc* or decreas* or increas* or lower* or fewer or improv* or enhance* or extend* or develop* or prevalen* or daily or weekly or current or rate or rates or behavio#r* or knowledge or prevent* or attitude* or avoid* or perceive* or percept* or promot* or program* or change*)) |
| S129 | (TI ("social-inclusion" N5 (reduc* or decreas* or increas* or lower* or fewer or improv* or enhance* or extend* or develop* or prevalen* or daily or weekly or current or rate or rates or behavio#r* or knowledge or prevent* or attitude* or avoid* or perceive* or percept* or promot* or program* or change*)) OR (AB ("social-inclusion" N5 (reduc* or decreas* or increas* or lower* or fewer or improv* or enhance* or extend* or develop* or prevalen* or daily or weekly or current or rate or rates or behavio#r* or knowledge or prevent* or attitude* or avoid* or perceive* or percept* or promot* or program* or change*)) |
| S130 | (TI ("social develop*" N5 (reduc* or decreas* or increas* or lower* or fewer or improv* or enhance* or extend* or develop* or prevalen* or daily or weekly or current or rate or rates or behavio#r* or knowledge or prevent* or attitude* or avoid* or perceive* or percept* or promot* or program* or change*)) OR (AB ("social develop*" N5 (reduc* or decreas* or increas* or lower* or fewer or improv* or enhance* or extend* or develop* or prevalen* or daily or weekly or current or rate or rates or behavio#r* or knowledge or prevent* or attitude* or avoid* or perceive* or percept* or promot* or program* or change*)) |
| S131 | (TI ("social competen*" N5 (reduc* or decreas* or increas* or lower* or fewer or improv* or enhance* or extend* or develop* or prevalen* or daily or weekly or current or rate or rates or behavio#r* or knowledge or prevent* or attitude* or avoid* or perceive* or percept* or promot* or program* or change*)) OR (AB ("social competen*" N5 (reduc* or decreas* or increas* or lower* or fewer or improv* or enhance* or extend* or develop* or prevalen* or daily or weekly or current or rate or rates or behavio#r* or knowledge or prevent* or attitude* or avoid* or perceive* or percept* or promot* or program* or change*)) |
| S132 | (TI ("emotion* develop*" N5 (reduc* or decreas* or increas* or lower* or fewer or improv* or enhance* or extend* or develop* or prevalen* or daily or weekly or current or rate or rates or behavio#r* or knowledge or prevent* or attitude* or avoid* or perceive* or percept* or promot* or program* or change*)) OR (AB ("emotion* develop*" N5 (reduc* or decreas* or increas* or lower* or fewer or improv* or enhance* or extend* or develop* or prevalen* or daily or weekly or current or rate or rates or behavio#r* or knowledge or prevent* or attitude* or avoid* or perceive* or percept* or promot* or program* or change*)) |
| S133 | (TI (conduct N5 (reduc* or decreas* or increas* or lower* or fewer or improv* or enhance* or extend* or develop* or prevalen* or daily or weekly or current or rate or rates or behavio#r* or knowledge or prevent* or attitude* or avoid* or perceive* or percept* or promot* or program* or change*)) OR (AB (conduct N5 (reduc* or decreas* or increas* or lower* or fewer or improv* or enhance* or extend* or develop* or prevalen* or daily or weekly or current or rate or rates or behavio#r* or knowledge or prevent* or attitude* or avoid* or perceive* or percept* or promot* or program* or change*)) |
| S134 | (TI (respect N5 (reduc* or decreas* or increas* or lower* or fewer or improv* or enhance* or extend* or develop* or prevalen* or daily or weekly or current or rate or rates or behavio#r* or knowledge or prevent* or attitude* or avoid* or perceive* or percept* or promot* or program* or change*)) OR (AB (respect N5 (reduc* or decreas* or increas* or lower* or fewer or improv* or enhance* or extend* or develop* or prevalen* or daily or weekly or current or rate or rates or behavio#r* or knowledge or prevent* or attitude* or avoid* or perceive* or percept* or promot* or program* or change*)) |
| S135 | (TI ((safety or safe) N5 (reduc* or decreas* or increas* or lower* or fewer or improv* or enhance* or extend* or develop* or prevalen* or daily or weekly or current or rate or rates or behavio#r* or knowledge or prevent* or attitude* or avoid* or perceive* or percept* or promot* or program* or change*))) OR (AB ((safety or safe) N5 (reduc* or decreas* or increas* or lower* or fewer or improv* or enhance* or extend* or develop* or prevalen* or daily or weekly or current or rate or rates or behavio#r* or knowledge or prevent* or attitude* or avoid* or perceive* or percept* or promot* or program* or change*))) |
| S136 | S70 OR S71 OR S72 OR S73 OR S74 OR S75 OR S76 OR S77 OR S78 OR S79 OR S80 OR S81 OR S82 OR S83 OR S84 OR S85 OR S86 OR S87 OR S88 OR S89 OR S90 OR S91 OR S92 OR S93 OR S94 OR S95 OR S96 OR S97 OR S98 OR S99 OR S100 OR S101 OR S102 OR S103 OR S104 OR S105 OR S106 OR S107 OR S108 OR S109 OR S110 OR S111 OR S112 OR S113 OR S114 OR S115 OR S116 OR S117 OR S118 OR S119 OR S120 OR S121 OR S122 OR S123 OR S124 OR S125 OR S126 OR S127 OR S128 OR S129 OR S130 OR S131 OR S132 OR S133 OR S134 OR S135 |
| S137 | (TI prevent*) OR (AB prevent*) |
| S138 | (TI program*) OR (AB program*) |
| S139 | (TI intervent*) OR (AB intervent*) |
| S140 | (TI "restorative approach*") OR (AB "restorative approach*") |
| S141 | (TI "control school#") OR (AB "control school#") |
| S142 | (TI (trial or trials)) OR (AB (trial or trials)) |
| S143 | S137 OR S138 OR S139 OR S140 OR S141 OR S142 |
| S144 | S40 AND S69 AND S136 AND S143 |

## EBSCO Educational Resources Information Center (ERIC)

| Database name | Educational Resources Information Center (ERIC) |
| --- | --- |
| Database platform | EBSCO |
| Dates of database coverage | Complete database to date searched. |
| Date searched | 17/01/2020 |
| Searched by | JF |
| Number of results | 14,891 |
| EndNote import order | 12 |
| Number of results once duplicates removed | 10,140 |
| Search strategy notes | * is used for truncation. # is used for an optional wildcard. Search strings starting with TI search the title only. Search strings starting with AB search the abstract only. Search strings starting with SO search the journal title only. N/*n* search for terms within *n* words of each other, in any order. |

| Search number | Query |
| --- | --- |
| S1 | (TI child*) or (AB child*) |
| S2 | (TI boy#) or (AB boy#) |
| S3 | (TI girl#) or (AB girl#) |
| S4 | (TI (schoolage or (school N1 age))) or (AB (schoolage or (school N1 age))) |
| S5 | (TI schoolchild*) or (AB schoolchild*) |
| S6 | (TI youngster#) OR (AB youngster#) |
| S7 | (TI minor#) OR (AB minor#) |
| S8 | (TI pupil*) OR (AB pupil*) |
| S9 | ((TI student*) OR (AB student*)) NOT ((TI "medical student*") or (AB "medical student*")) |
| S10 | (TI prepubescen*) OR (AB prepubescen*) |
| S11 | (TI pubescent*) or (AB pubescent*) |
| S12 | (TI adolescent*) OR (AB adolescent*) |
| S13 | (TI juvenil*) OR (AB juvenil*) |
| S14 | (TI underage*) or (AB underage*) |
| S15 | (TI (preteen* or pre-teen*)) OR (AB (preteen* or pre-teen*)) |
| S16 | (TI (teen or teens)) OR (AB (teen or teens)) |
| S17 | (TI teenage*) OR (AB teenage*) |
| S18 | (TI (youth or youths)) OR (AB (youth or youths)) |
| S19 | (TI "young person*") OR (AB "young person*") |
| S20 | (TI "young people*") OR (AB "young people*") |
| S21 | (TI (kid or kids)) OR (AB (kid or kids)) |
| S22 | (TI (transition n4 adult*)) OR (AB (transition N4 adult*)) |
| S23 | (TI "emerging adult*") OR (AB "emerging adult*") |
| S24 | (TI "young adult*") OR (AB "young adult*") |
| S25 | (TI parent#) OR (AB parent#) |
| S26 | (TI mother#) OR (AB mother#) |
| S27 | (TI father#) or (AB father#) |
| S28 | (TI guardian#) OR (AB guardian#) |
| S29 | (TI teacher#) OR (AB teacher#) |
| S30 | (SO child*) |
| S31 | (SO adolescen*) |
| S32 | (SO youth*) |
| S33 | (SO school*) |
| S34 | (TI ("5" or "6" or "7" or "8" or "9" or "10" or "11" or "12" or "13" or "14" or "15" or "16" or "17" or "18") N1 ("year* old" or "year* of age")) OR (AB ("5" or "6" or "7" or "8" or "9" or "10" or "11" or "12" or "13" or "14" or "15" or "16" or "17" or "18") N1 ("year* old" or "year* of age")) |
| S35 | (TI (five or six or seven or eight or nine or ten or eleven or twelve or thirteen or fourteen or fifteen or sixteen or seventeen or eighteen) N1 ("year* old" or "year* of age")) OR (AB (five or six or seven or eight or nine or ten or eleven or twelve or thirteen or fourteen or fifteen or sixteen or seventeen or eighteen) N1 ("year* old" or "year* of age")) |
| S36 | (TI (age* N1 ("5" or "6" or "7" or "8" or "9" or "10" or "11" or "12" or "13" or "14" or "15" or "16" or "17" or "18") N1 year*)) OR (AB (age* N1 ("5" or "6" or "7" or "8" or "9" or "10" or "11" or "12" or "13" or "14" or "15" or "16" or "17" or "18") N1 year*)) |
| S37 | (TI (age* N1 (five or six or seven or eight or nine or ten or eleven or twelve or thirteen or fourteen or fifteen or sixteen or seventeen or eighteen) N1 year*)) OR (AB (age* N1 (five or six or seven or eight or nine or ten or eleven or twelve or thirteen or fourteen or fifteen or sixteen or seventeen or eighteen) N1 year*)) |
| S38 | (TI (primary or secondary or year) N1 ("1" or "2" or "3" or "4" or "5" or "6" or "7" or "8" or "9" or "10" or "11" or "12" or "13")) OR (AB (primary or secondary or year) N1 ("1" or "2" or "3" or "4" or "5" or "6" or "7" or "8" or "9" or "10" or "11" or "12" or "13")) |
| S39 | (TI grade# N1 (first or second or third or fourth or fifth or sixth or seventh or eighth or ninth or tenth or eleventh or twelfth)) OR (AB grade# N1 (first or second or third or fourth or fifth or sixth or seventh or eighth or ninth or tenth or eleventh or twelfth)) |
| S40 | S1 OR S2 OR S3 OR S4 OR S5 OR S6 OR S7 OR S8 OR S9 OR S10 OR S11 OR S12 OR S13 OR S14 OR S15 OR S16 OR S17 OR S18 OR S19 OR S20 OR S21 OR S22 OR S23 OR S24 OR S25 OR S26 OR S27 OR S28 OR S29 OR S30 OR S31 OR S32 OR S33 OR S34 OR S35 OR S36 OR S37 OR S38 OR S39 |
| S41 | (TI (school# N5 (based or level or wide or led or ethos or environment or organi#ation* or climate or toolkit# or approach* or "action group#" or practice# or whole or health* or leader* or cultur* or manag* or governance or system# or communit*))) OR (AB (school# N5 (based or level or wide or led or ethos or environment or organi#ation* or climate or toolkit# or approach* or "action group#" or practice# or whole or health* or leader* or cultur* or manag* or governance or system# or communit*))) |
| S42 | (TI schoolwide) or (AB schoolwide) |
| S43 | (TI "restor* justice") OR (AB "restor* justice") |
| S44 | (TI school#) or (AB school#) |
| S45 | (TI "peer-to-peer") OR (AB "peer-to-peer") |
| S46 | (TI (peer N2 (led or leader* or participat* or mediat* or helper# or resistan* or wide or action# or influen*))) OR (AB (peer N2 (led or leader* or participat* or mediat* or helper# or resistan* or wide or action# or influen*))) |
| S47 | (TI (relationship* N2 (led or leader* or participat* or mediat* or helper# or resistan* or wide or action# or influen*))) OR (AB (relationship* N2 (led or leader* or participat* or mediat* or helper# or resistan* or wide or action# or influen*))) |
| S48 | (TI ((community or communities) N2 (led or leader* or participat* or mediat* or helper# or resistan* or wide or action# or influen* or relationship*))) OR (AB ((community or communities) N2 (led or leader* or participat* or mediat* or helper# or resistan* or wide or action# or influen* or relationship*))) |
| S49 | ((TI (pupil# N2 (led or leader* or participat* or mediat* or helper# or resistan* or wide or action# or influen* or relationship*)) OR (AB (pupil# N2 (led or leader* or participat* or mediat* or helper# or resistan* or wide or action# or influen* or relationship*))) |
| S50 | (TI ((child or children) N2 (led or leader* or participat* or mediat* or helper# or resistan* or wide or action# or influen*))) OR (AB (child or children*) N2 (led or leader* or participat* or mediat* or helper# or resistan* or wide or action# or influen*))) |
| S51 | (TI (("young people" or "young person") N2 (led or leader* or participat* or mediat* or helper# or resistan* or wide or action# or influen*))) OR (AB (("young people" or "young person") N2 (led or leader* or participat* or mediat* or helper# or resistan* or wide or action# or influen*))) |
| S52 | (TI ((teen or teens or teenage*) N2 (led or leader* or participat* or mediat* or helper# or resistan* or wide or action# or influen*))) OR (AB ((teen or teens or teenage*) N2 (led or leader* or participat* or mediat* or helper# or resistan* or wide or action# or influen*))) |
| S53 | (TI (adolescen* N2 (led or leader* or participat* or mediat* or helper# or resistan* or wide or action# or influen*))) OR (AB (adolescen* N2 (led or leader* or participat* or mediat* or helper# or resistan* or wide or action# or influen*))) |
| S54 | (TI (youth# N2 (led or leader* or participat* or mediat* or helper# or resistan* or wide or action# or influen*))) OR (AB (youth# N2 (led or leader* or participat* or mediat* or helper# or resistan* or wide or action# or influen*))) |
| S55 | (TI (student# N2 (led or leader* or participat* or mediat* or helper# or resistan* or wide or action# or influen* or relationship*))) OR (AB (student# N2 (led or leader* or participat* or mediat* or helper# or resistan* or wide or action# or influen* or relationship*))) |
| S56 | (TI (teach* N2 (led or leader* or participat* or mediat* or helper# or resistan* or wide or action# or influen* or relationship*))) OR (AB (teach* N2 (led or leader* or participat* or mediat* or helper# or resistan* or wide or action# or influen* or relationship*))) |
| S57 | (TI (curricul* N2 (led or leader* or participat* or mediat* or helper# or resistan* or wide or action# or influen* or relationship*))) OR (AB (curricul* N2 (led or leader* or participat* or mediat* or helper# or resistan* or wide or action# or influen* or relationship*))) |
| S58 | (TI (administrat* N2 (led or leader* or participat* or mediat* or helper# or resistan* or wide or action# or influen* or relationship*))) OR (AB (administrat* N2 (led or leader* or participat* or mediat* or helper# or resistan* or wide or action# or influen* or relationship*))) |
| S59 | (TI ((personnel or staff) N2 (led or leader* or participat* or mediat* or helper# or resistan* or wide or action# or influen* or relationship*))) OR (AB ((personnel or staff) N2 (led or leader* or participat* or mediat* or helper# or resistan* or wide or action# or influen* or relationship*))) |
| S60 | (TI (volunteer* N2 (led or leader* or participat* or mediat* or helper# or resistan* or wide or action# or influen* or relationship*))) OR (AB (volulnteer* N2 (led or leader* or participat* or mediat* or helper# or resistan* or wide or action# or influen* or relationship*))) |
| S61 | (TI ((advocacy or advocate) N2 (led or leader* or participat* or mediat* or helper# or resistan* or wide or action# or influen* or relationship*))) OR (AB ((advocacy or advocate) N2 (led or leader* or participat* or mediat* or helper# or resistan* or wide or action# or influen* or relationship*))) |
| S62 | (TI (parent* N2 (led or leader* or participat* or mediat* or helper# or resistan* or wide or action# or influen*))) OR (AB (parent* N2 (led or leader* or participat* or mediat* or helper# or resistan* or wide or action# or influen*))) |
| S63 | (TI (environment* N2 (led or leader* or participat* or mediat* or helper# or resistan* or wide or action# or influen* or relationship*))) OR (AB (environment* N2 (led or leader* or participat* or mediat* or helper# or resistan* or wide or action# or influen* or relationship*))) |
| S64 | (TI ("socio-ecolog*" N2 (led or leader* or participat* or mediat* or helper# or resistan* or wide or action# or influen* or relationship*))) OR (AB ("socio-ecolog*" N2 (led or leader* or participat* or mediat* or helper# or resistan* or wide or action# or influen* or relationship*))) |
| S65 | (TI (learn* N2 (led or leader* or participat* or mediat* or helper# or resistan* or wide or action# or influen* or relationship*))) OR (AB (learn* N2 (led or leader* or participat* or mediat* or helper# or resistan* or wide or action# or influen* or relationship*))) |
| S66 | (TI ((policy or policies) N2 (led or leader* or participat* or mediat* or helper# or resistan* or wide or action# or influen* or relationship*))) OR (AB ((policy or policies) N2 (led or leader* or participat* or mediat* or helper# or resistan* or wide or action# or influen* or relationship*))) |
| S67 | S45 OR S46 OR S47 OR S48 OR S49 OR S50 OR S51 OR S52 OR S53 OR S54 OR S55 OR S56 OR S57 OR S58 OR S59 OR S60 OR S61 OR S62 OR S63 OR S64 OR S65 OR S66 |
| S68 | S44 AND S67 |
| S69 | S41 OR S42 OR S43 OR S68 |
| S70 | (TI ((smoking or smoked or smoke or smoker) N5 (reduc* or decreas* or increas* or lower* or fewer or improv* or enhance* or extend* or develop* or prevalen* or daily or weekly or current or rate or rates or behavio#r* or knowledge or prevent* or attitude* or avoid* or perceive* or percept* or promot* or program* or change*))) OR (AB ((smoking or smoked or smoke or smoker) N5 (reduc* or decreas* or increas* or lower* or fewer or improv* or enhance* or extend* or develop* or prevalen* or daily or weekly or current or rate or rates or behavio#r* or knowledge or prevent* or attitude* or avoid* or perceive* or percept* or promot* or program* or change*))) |
| S71 | (TI (tobacco N5 (reduc* or decreas* or increas* or lower* or fewer or improv* or enhance* or extend* or develop* or prevalen* or daily or weekly or current or rate or rates or behavio#r* or knowledge or prevent* or attitude* or avoid* or perceive* or percept* or promot* or program* or change*)) OR (AB (tobacco N5 (reduc* or decreas* or increas* or lower* or fewer or improv* or enhance* or extend* or develop* or prevalen* or daily or weekly or current or rate or rates or behavio#r* or knowledge or prevent* or attitude* or avoid* or perceive* or percept* or promot* or program* or change*)) |
| S72 | (TI (cigarette# N5 (reduc* or decreas* or increas* or lower* or fewer or improv* or enhance* or extend* or develop* or prevalen* or daily or weekly or current or rate or rates or behavio#r* or knowledge or prevent* or attitude* or avoid* or perceive* or percept* or promot* or program* or change*)) OR (AB (cigarette# N5 (reduc* or decreas* or increas* or lower* or fewer or improv* or enhance* or extend* or develop* or prevalen* or daily or weekly or current or rate or rates or behavio#r* or knowledge or prevent* or attitude* or avoid* or perceive* or percept* or promot* or program* or change*)) |
| S73 | (TI (marijuana N5 (reduc* or decreas* or increas* or lower* or fewer or improv* or enhance* or extend* or develop* or prevalen* or daily or weekly or current or rate or rates or behavio#r* or knowledge or prevent* or attitude* or avoid* or perceive* or percept* or promot* or program* or change*)) OR (AB (marijuana N5 (reduc* or decreas* or increas* or lower* or fewer or improv* or enhance* or extend* or develop* or prevalen* or daily or weekly or current or rate or rates or behavio#r* or knowledge or prevent* or attitude* or avoid* or perceive* or percept* or promot* or program* or change*)) |
| S74 | (TI (cannabis N5 (reduc* or decreas* or increas* or lower* or fewer or improv* or enhance* or extend* or develop* or prevalen* or daily or weekly or current or rate or rates or behavio#r* or knowledge or prevent* or attitude* or avoid* or perceive* or percept* or promot* or program* or change*)) OR (AB (cannabis N5 (reduc* or decreas* or increas* or lower* or fewer or improv* or enhance* or extend* or develop* or prevalen* or daily or weekly or current or rate or rates or behavio#r* or knowledge or prevent* or attitude* or avoid* or perceive* or percept* or promot* or program* or change*)) |
| S75 | (TI (solvent# N5 (reduc* or decreas* or increas* or lower* or fewer or improv* or enhance* or extend* or develop* or prevalen* or daily or weekly or current or rate or rates or behavio#r* or knowledge or prevent* or attitude* or avoid* or perceive* or percept* or promot* or program* or change*)) OR (AB (solvent# N5 (reduc* or decreas* or increas* or lower* or fewer or improv* or enhance* or extend* or develop* or prevalen* or daily or weekly or current or rate or rates or behavio#r* or knowledge or prevent* or attitude* or avoid* or perceive* or percept* or promot* or program* or change*)) |
| S76 | (TI (alcohol N5 (reduc* or decreas* or increas* or lower* or fewer or improv* or enhance* or extend* or develop* or prevalen* or daily or weekly or current or rate or rates or behavio#r* or knowledge or prevent* or attitude* or avoid* or perceive* or percept* or promot* or program* or change*)) OR (AB (alcohol N5 (reduc* or decreas* or increas* or lower* or fewer or improv* or enhance* or extend* or develop* or prevalen* or daily or weekly or current or rate or rates or behavio#r* or knowledge or prevent* or attitude* or avoid* or perceive* or percept* or promot* or program* or change*)) |
| S77 | (TI ("binge drink*" N5 (reduc* or decreas* or increas* or lower* or fewer or improv* or enhance* or extend* or develop* or prevalen* or daily or weekly or current or rate or rates or behavio#r* or knowledge or prevent* or attitude* or avoid* or perceive* or percept* or promot* or program* or change*)) OR (AB ("binge drink*" N5 (reduc* or decreas* or increas* or lower* or fewer or improv* or enhance* or extend* or develop* or prevalen* or daily or weekly or current or rate or rates or behavio#r* or knowledge or prevent* or attitude* or avoid* or perceive* or percept* or promot* or program* or change*)) |
| S78 | (TI ("underage drink*" N5 (reduc* or decreas* or increas* or lower* or fewer or improv* or enhance* or extend* or develop* or prevalen* or daily or weekly or current or rate or rates or behavio#r* or knowledge or prevent* or attitude* or avoid* or perceive* or percept* or promot* or program* or change*)) OR (AB ("underage drink*" N5 (reduc* or decreas* or increas* or lower* or fewer or improv* or enhance* or extend* or develop* or prevalen* or daily or weekly or current or rate or rates or behavio#r* or knowledge or prevent* or attitude* or avoid* or perceive* or percept* or promot* or program* or change*)) |
| S79 | (TI ("underage drink*" N5 (reduc* or decreas* or increas* or lower* or fewer or improv* or enhance* or extend* or develop* or prevalen* or daily or weekly or current or rate or rates or behavio#r* or knowledge or prevent* or attitude* or avoid* or perceive* or percept* or promot* or program* or change*)) OR (AB ("underage drink*" N5 (reduc* or decreas* or increas* or lower* or fewer or improv* or enhance* or extend* or develop* or prevalen* or daily or weekly or current or rate or rates or behavio#r* or knowledge or prevent* or attitude* or avoid* or perceive* or percept* or promot* or program* or change*)) |
| S80 | (TI (substance# N5 (reduc* or decreas* or increas* or lower* or fewer or improv* or enhance* or extend* or develop* or prevalen* or daily or weekly or current or rate or rates or behavio#r* or knowledge or prevent* or attitude* or avoid* or perceive* or percept* or promot* or program* or change*)) OR (AB (substance# N5 (reduc* or decreas* or increas* or lower* or fewer or improv* or enhance* or extend* or develop* or prevalen* or daily or weekly or current or rate or rates or behavio#r* or knowledge or prevent* or attitude* or avoid* or perceive* or percept* or promot* or program* or change*)) |
| S81 | (TI (drug# N5 (reduc* or decreas* or increas* or lower* or fewer or improv* or enhance* or extend* or develop* or prevalen* or daily or weekly or current or rate or rates or behavio#r* or knowledge or prevent* or attitude* or avoid* or perceive* or percept* or promot* or program* or change*)) OR (AB (drug# N5 (reduc* or decreas* or increas* or lower* or fewer or improv* or enhance* or extend* or develop* or prevalen* or daily or weekly or current or rate or rates or behavio#r* or knowledge or prevent* or attitude* or avoid* or perceive* or percept* or promot* or program* or change*)) |
| S82 | (TI ("education* attain*" N5 (reduc* or decreas* or increas* or lower* or fewer or improv* or enhance* or extend* or develop* or prevalen* or daily or weekly or current or rate or rates or behavio#r* or knowledge or prevent* or attitude* or avoid* or perceive* or percept* or promot* or program* or change*)) OR (AB ("education* attain*" N5 (reduc* or decreas* or increas* or lower* or fewer or improv* or enhance* or extend* or develop* or prevalen* or daily or weekly or current or rate or rates or behavio#r* or knowledge or prevent* or attitude* or avoid* or perceive* or percept* or promot* or program* or change*)) |
| S83 | (TI ("academic attain*" N5 (reduc* or decreas* or increas* or lower* or fewer or improv* or enhance* or extend* or develop* or prevalen* or daily or weekly or current or rate or rates or behavio#r* or knowledge or prevent* or attitude* or avoid* or perceive* or percept* or promot* or program* or change*)) OR (AB ("academic attain*" N5 (reduc* or decreas* or increas* or lower* or fewer or improv* or enhance* or extend* or develop* or prevalen* or daily or weekly or current or rate or rates or behavio#r* or knowledge or prevent* or attitude* or avoid* or perceive* or percept* or promot* or program* or change*)) |
| S84 | (TI ("student* attain*" N5 (reduc* or decreas* or increas* or lower* or fewer or improv* or enhance* or extend* or develop* or prevalen* or daily or weekly or current or rate or rates or behavio#r* or knowledge or prevent* or attitude* or avoid* or perceive* or percept* or promot* or program* or change*)) OR (AB ("student* attain*" N5 (reduc* or decreas* or increas* or lower* or fewer or improv* or enhance* or extend* or develop* or prevalen* or daily or weekly or current or rate or rates or behavio#r* or knowledge or prevent* or attitude* or avoid* or perceive* or percept* or promot* or program* or change*)) |
| S85 | (TI ("education* achieve*" N5 (reduc* or decreas* or increas* or lower* or fewer or improv* or enhance* or extend* or develop* or prevalen* or daily or weekly or current or rate or rates or behavio#r* or knowledge or prevent* or attitude* or avoid* or perceive* or percept* or promot* or program* or change*)) OR (AB ("education* achieve*" N5 (reduc* or decreas* or increas* or lower* or fewer or improv* or enhance* or extend* or develop* or prevalen* or daily or weekly or current or rate or rates or behavio#r* or knowledge or prevent* or attitude* or avoid* or perceive* or percept* or promot* or program* or change*)) |
| S86 | (TI ("academic achieve*" N5 (reduc* or decreas* or increas* or lower* or fewer or improv* or enhance* or extend* or develop* or prevalen* or daily or weekly or current or rate or rates or behavio#r* or knowledge or prevent* or attitude* or avoid* or perceive* or percept* or promot* or program* or change*)) OR (AB ("academic achieve*" N5 (reduc* or decreas* or increas* or lower* or fewer or improv* or enhance* or extend* or develop* or prevalen* or daily or weekly or current or rate or rates or behavio#r* or knowledge or prevent* or attitude* or avoid* or perceive* or percept* or promot* or program* or change*)) |
| S87 | (TI ("student* achieve*" N5 (reduc* or decreas* or increas* or lower* or fewer or improv* or enhance* or extend* or develop* or prevalen* or daily or weekly or current or rate or rates or behavio#r* or knowledge or prevent* or attitude* or avoid* or perceive* or percept* or promot* or program* or change*)) OR (AB ("student* achieve*" N5 (reduc* or decreas* or increas* or lower* or fewer or improv* or enhance* or extend* or develop* or prevalen* or daily or weekly or current or rate or rates or behavio#r* or knowledge or prevent* or attitude* or avoid* or perceive* or percept* or promot* or program* or change*)) |
| S88 | (TI ("education* perform*" N5 (reduc* or decreas* or increas* or lower* or fewer or improv* or enhance* or extend* or develop* or prevalen* or daily or weekly or current or rate or rates or behavio#r* or knowledge or prevent* or attitude* or avoid* or perceive* or percept* or promot* or program* or change*)) OR (AB ("education* perform*" N5 (reduc* or decreas* or increas* or lower* or fewer or improv* or enhance* or extend* or develop* or prevalen* or daily or weekly or current or rate or rates or behavio#r* or knowledge or prevent* or attitude* or avoid* or perceive* or percept* or promot* or program* or change*)) |
| S89 | (TI ("academic perform*" N5 (reduc* or decreas* or increas* or lower* or fewer or improv* or enhance* or extend* or develop* or prevalen* or daily or weekly or current or rate or rates or behavio#r* or knowledge or prevent* or attitude* or avoid* or perceive* or percept* or promot* or program* or change*)) OR (AB ("academic perform*" N5 (reduc* or decreas* or increas* or lower* or fewer or improv* or enhance* or extend* or develop* or prevalen* or daily or weekly or current or rate or rates or behavio#r* or knowledge or prevent* or attitude* or avoid* or perceive* or percept* or promot* or program* or change*)) |
| S90 | (TI ("student* perform*" N5 (reduc* or decreas* or increas* or lower* or fewer or improv* or enhance* or extend* or develop* or prevalen* or daily or weekly or current or rate or rates or behavio#r* or knowledge or prevent* or attitude* or avoid* or perceive* or percept* or promot* or program* or change*)) OR (AB ("student* perform*" N5 (reduc* or decreas* or increas* or lower* or fewer or improv* or enhance* or extend* or develop* or prevalen* or daily or weekly or current or rate or rates or behavio#r* or knowledge or prevent* or attitude* or avoid* or perceive* or percept* or promot* or program* or change*)) |
| S91 | (TI ("education* outcome#" N5 (reduc* or decreas* or increas* or lower* or fewer or improv* or enhance* or extend* or develop* or prevalen* or daily or weekly or current or rate or rates or behavio#r* or knowledge or prevent* or attitude* or avoid* or perceive* or percept* or promot* or program* or change*)) OR (AB ("education* outcome#" N5 (reduc* or decreas* or increas* or lower* or fewer or improv* or enhance* or extend* or develop* or prevalen* or daily or weekly or current or rate or rates or behavio#r* or knowledge or prevent* or attitude* or avoid* or perceive* or percept* or promot* or program* or change*)) |
| S92 | (TI ("academic outcome#" N5 (reduc* or decreas* or increas* or lower* or fewer or improv* or enhance* or extend* or develop* or prevalen* or daily or weekly or current or rate or rates or behavio#r* or knowledge or prevent* or attitude* or avoid* or perceive* or percept* or promot* or program* or change*)) OR (AB ("academic outcome#" N5 (reduc* or decreas* or increas* or lower* or fewer or improv* or enhance* or extend* or develop* or prevalen* or daily or weekly or current or rate or rates or behavio#r* or knowledge or prevent* or attitude* or avoid* or perceive* or percept* or promot* or program* or change*)) |
| S93 | (TI ("student* outcome#" N5 (reduc* or decreas* or increas* or lower* or fewer or improv* or enhance* or extend* or develop* or prevalen* or daily or weekly or current or rate or rates or behavio#r* or knowledge or prevent* or attitude* or avoid* or perceive* or percept* or promot* or program* or change*)) OR (AB ("student* outcome#" N5 (reduc* or decreas* or increas* or lower* or fewer or improv* or enhance* or extend* or develop* or prevalen* or daily or weekly or current or rate or rates or behavio#r* or knowledge or prevent* or attitude* or avoid* or perceive* or percept* or promot* or program* or change*)) |
| S94 | (TI ("education* commitment" N5 (reduc* or decreas* or increas* or lower* or fewer or improv* or enhance* or extend* or develop* or prevalen* or daily or weekly or current or rate or rates or behavio#r* or knowledge or prevent* or attitude* or avoid* or perceive* or percept* or promot* or program* or change*)) OR (AB ("education* commitment" N5 (reduc* or decreas* or increas* or lower* or fewer or improv* or enhance* or extend* or develop* or prevalen* or daily or weekly or current or rate or rates or behavio#r* or knowledge or prevent* or attitude* or avoid* or perceive* or percept* or promot* or program* or change*)) |
| S95 | (TI ("education* commitment" N5 (reduc* or decreas* or increas* or lower* or fewer or improv* or enhance* or extend* or develop* or prevalen* or daily or weekly or current or rate or rates or behavio#r* or knowledge or prevent* or attitude* or avoid* or perceive* or percept* or promot* or program* or change*)) OR (AB ("education* commitment" N5 (reduc* or decreas* or increas* or lower* or fewer or improv* or enhance* or extend* or develop* or prevalen* or daily or weekly or current or rate or rates or behavio#r* or knowledge or prevent* or attitude* or avoid* or perceive* or percept* or promot* or program* or change*)) |
| S96 | (TI ("academic commitment" N5 (reduc* or decreas* or increas* or lower* or fewer or improv* or enhance* or extend* or develop* or prevalen* or daily or weekly or current or rate or rates or behavio#r* or knowledge or prevent* or attitude* or avoid* or perceive* or percept* or promot* or program* or change*)) OR (AB ("academic commitment" N5 (reduc* or decreas* or increas* or lower* or fewer or improv* or enhance* or extend* or develop* or prevalen* or daily or weekly or current or rate or rates or behavio#r* or knowledge or prevent* or attitude* or avoid* or perceive* or percept* or promot* or program* or change*)) |
| S97 | (TI ("academic commitment" N5 (reduc* or decreas* or increas* or lower* or fewer or improv* or enhance* or extend* or develop* or prevalen* or daily or weekly or current or rate or rates or behavio#r* or knowledge or prevent* or attitude* or avoid* or perceive* or percept* or promot* or program* or change*)) OR (AB ("academic commitment" N5 (reduc* or decreas* or increas* or lower* or fewer or improv* or enhance* or extend* or develop* or prevalen* or daily or weekly or current or rate or rates or behavio#r* or knowledge or prevent* or attitude* or avoid* or perceive* or percept* or promot* or program* or change*)) |
| S98 | (TI ("student* commitment" N5 (reduc* or decreas* or increas* or lower* or fewer or improv* or enhance* or extend* or develop* or prevalen* or daily or weekly or current or rate or rates or behavio#r* or knowledge or prevent* or attitude* or avoid* or perceive* or percept* or promot* or program* or change*)) OR (AB ("student* commitment" N5 (reduc* or decreas* or increas* or lower* or fewer or improv* or enhance* or extend* or develop* or prevalen* or daily or weekly or current or rate or rates or behavio#r* or knowledge or prevent* or attitude* or avoid* or perceive* or percept* or promot* or program* or change*)) |
| S99 | (TI ("education* engage*" N5 (reduc* or decreas* or increas* or lower* or fewer or improv* or enhance* or extend* or develop* or prevalen* or daily or weekly or current or rate or rates or behavio#r* or knowledge or prevent* or attitude* or avoid* or perceive* or percept* or promot* or program* or change*)) OR (AB ("education* engage*" N5 (reduc* or decreas* or increas* or lower* or fewer or improv* or enhance* or extend* or develop* or prevalen* or daily or weekly or current or rate or rates or behavio#r* or knowledge or prevent* or attitude* or avoid* or perceive* or percept* or promot* or program* or change*)) |
| S100 | (TI ("academic engage*" N5 (reduc* or decreas* or increas* or lower* or fewer or improv* or enhance* or extend* or develop* or prevalen* or daily or weekly or current or rate or rates or behavio#r* or knowledge or prevent* or attitude* or avoid* or perceive* or percept* or promot* or program* or change*)) OR (AB ("academic engage*" N5 (reduc* or decreas* or increas* or lower* or fewer or improv* or enhance* or extend* or develop* or prevalen* or daily or weekly or current or rate or rates or behavio#r* or knowledge or prevent* or attitude* or avoid* or perceive* or percept* or promot* or program* or change*)) |
| S101 | (TI ("student* engage*" N5 (reduc* or decreas* or increas* or lower* or fewer or improv* or enhance* or extend* or develop* or prevalen* or daily or weekly or current or rate or rates or behavio#r* or knowledge or prevent* or attitude* or avoid* or perceive* or percept* or promot* or program* or change*)) OR (AB ("student* engage*" N5 (reduc* or decreas* or increas* or lower* or fewer or improv* or enhance* or extend* or develop* or prevalen* or daily or weekly or current or rate or rates or behavio#r* or knowledge or prevent* or attitude* or avoid* or perceive* or percept* or promot* or program* or change*)) |
| S102 | (TI (dropout N5 (reduc* or decreas* or increas* or lower* or fewer or improv* or enhance* or extend* or develop* or prevalen* or daily or weekly or current or rate or rates or behavio#r* or knowledge or prevent* or attitude* or avoid* or perceive* or percept* or promot* or program* or change*)) OR (AB (dropout N5 (reduc* or decreas* or increas* or lower* or fewer or improv* or enhance* or extend* or develop* or prevalen* or daily or weekly or current or rate or rates or behavio#r* or knowledge or prevent* or attitude* or avoid* or perceive* or percept* or promot* or program* or change*)) |
| S103 | (TI (absentee* N5 (reduc* or decreas* or increas* or lower* or fewer or improv* or enhance* or extend* or develop* or prevalen* or daily or weekly or current or rate or rates or behavio#r* or knowledge or prevent* or attitude* or avoid* or perceive* or percept* or promot* or program* or change*)) OR (AB (absentee* N5 (reduc* or decreas* or increas* or lower* or fewer or improv* or enhance* or extend* or develop* or prevalen* or daily or weekly or current or rate or rates or behavio#r* or knowledge or prevent* or attitude* or avoid* or perceive* or percept* or promot* or program* or change*)) |
| S104 | (TI ((violent or violence) N5 (reduc* or decreas* or increas* or lower* or fewer or improv* or enhance* or extend* or develop* or prevalen* or daily or weekly or current or rate or rates or behavio#r* or knowledge or prevent* or attitude* or avoid* or perceive* or percept* or promot* or program* or change*))) OR (AB ((violent or violence) N5 (reduc* or decreas* or increas* or lower* or fewer or improv* or enhance* or extend* or develop* or prevalen* or daily or weekly or current or rate or rates or behavio#r* or knowledge or prevent* or attitude* or avoid* or perceive* or percept* or promot* or program* or change*))) |
| S105 | (TI ((aggression or aggressive) N5 (reduc* or decreas* or increas* or lower* or fewer or improv* or enhance* or extend* or develop* or prevalen* or daily or weekly or current or rate or rates or behavio#r* or knowledge or prevent* or attitude* or avoid* or perceive* or percept* or promot* or program* or change*))) OR (AB ((aggression or aggressive) N5 (reduc* or decreas* or increas* or lower* or fewer or improv* or enhance* or extend* or develop* or prevalen* or daily or weekly or current or rate or rates or behavio#r* or knowledge or prevent* or attitude* or avoid* or perceive* or percept* or promot* or program* or change*))) |
| S106 | (TI ((hostile or hostility) N5 (reduc* or decreas* or increas* or lower* or fewer or improv* or enhance* or extend* or develop* or prevalen* or daily or weekly or current or rate or rates or behavio#r* or knowledge or prevent* or attitude* or avoid* or perceive* or percept* or promot* or program* or change*))) OR (AB ((hostile or hostility) N5 (reduc* or decreas* or increas* or lower* or fewer or improv* or enhance* or extend* or develop* or prevalen* or daily or weekly or current or rate or rates or behavio#r* or knowledge or prevent* or attitude* or avoid* or perceive* or percept* or promot* or program* or change*))) |
| S107 | (TI (assault* N5 (reduc* or decreas* or increas* or lower* or fewer or improv* or enhance* or extend* or develop* or prevalen* or daily or weekly or current or rate or rates or behavio#r* or knowledge or prevent* or attitude* or avoid* or perceive* or percept* or promot* or program* or change*)) OR (AB (assault* N5 (reduc* or decreas* or increas* or lower* or fewer or improv* or enhance* or extend* or develop* or prevalen* or daily or weekly or current or rate or rates or behavio#r* or knowledge or prevent* or attitude* or avoid* or perceive* or percept* or promot* or program* or change*)) |
| S108 | (TI (crime N5 (reduc* or decreas* or increas* or lower* or fewer or improv* or enhance* or extend* or develop* or prevalen* or daily or weekly or current or rate or rates or behavio#r* or knowledge or prevent* or attitude* or avoid* or perceive* or percept* or promot* or program* or change*)) OR (AB (crime N5 (reduc* or decreas* or increas* or lower* or fewer or improv* or enhance* or extend* or develop* or prevalen* or daily or weekly or current or rate or rates or behavio#r* or knowledge or prevent* or attitude* or avoid* or perceive* or percept* or promot* or program* or change*)) |
| S109 | (TI ((abuse# or abusive) N5 (reduc* or decreas* or increas* or lower* or fewer or improv* or enhance* or extend* or develop* or prevalen* or daily or weekly or current or rate or rates or behavio#r* or knowledge or prevent* or attitude* or avoid* or perceive* or percept* or promot* or program* or change*))) OR (AB ((abuse# or abusive) N5 (reduc* or decreas* or increas* or lower* or fewer or improv* or enhance* or extend* or develop* or prevalen* or daily or weekly or current or rate or rates or behavio#r* or knowledge or prevent* or attitude* or avoid* or perceive* or percept* or promot* or program* or change*))) |
| S110 | (TI (misbehav* N5 (reduc* or decreas* or increas* or lower* or fewer or improv* or enhance* or extend* or develop* or prevalen* or daily or weekly or current or rate or rates or behavio#r* or knowledge or prevent* or attitude* or avoid* or perceive* or percept* or promot* or program* or change*)) OR (AB (misbehav* N5 (reduc* or decreas* or increas* or lower* or fewer or improv* or enhance* or extend* or develop* or prevalen* or daily or weekly or current or rate or rates or behavio#r* or knowledge or prevent* or attitude* or avoid* or perceive* or percept* or promot* or program* or change*)) |
| S111 | (TI (threat# N5 (reduc* or decreas* or increas* or lower* or fewer or improv* or enhance* or extend* or develop* or prevalen* or daily or weekly or current or rate or rates or behavio#r* or knowledge or prevent* or attitude* or avoid* or perceive* or percept* or promot* or program* or change*)) OR (AB (threat# N5 (reduc* or decreas* or increas* or lower* or fewer or improv* or enhance* or extend* or develop* or prevalen* or daily or weekly or current or rate or rates or behavio#r* or knowledge or prevent* or attitude* or avoid* or perceive* or percept* or promot* or program* or change*)) |
| S112 | (TI (conduct N5 (reduc* or decreas* or increas* or lower* or fewer or improv* or enhance* or extend* or develop* or prevalen* or daily or weekly or current or rate or rates or behavio#r* or knowledge or prevent* or attitude* or avoid* or perceive* or percept* or promot* or program* or change*)) OR (AB (conduct N5 (reduc* or decreas* or increas* or lower* or fewer or improv* or enhance* or extend* or develop* or prevalen* or daily or weekly or current or rate or rates or behavio#r* or knowledge or prevent* or attitude* or avoid* or perceive* or percept* or promot* or program* or change*)) |
| S113 | (TI (delinquen* N5 (reduc* or decreas* or increas* or lower* or fewer or improv* or enhance* or extend* or develop* or prevalen* or daily or weekly or current or rate or rates or behavio#r* or knowledge or prevent* or attitude* or avoid* or perceive* or percept* or promot* or program* or change*)) OR (AB (delinquen* N5 (reduc* or decreas* or increas* or lower* or fewer or improv* or enhance* or extend* or develop* or prevalen* or daily or weekly or current or rate or rates or behavio#r* or knowledge or prevent* or attitude* or avoid* or perceive* or percept* or promot* or program* or change*)) |
| S114 | (TI ("disrupt* behavio#r*" N5 (reduc* or decreas* or increas* or lower* or fewer or improv* or enhance* or extend* or develop* or prevalen* or daily or weekly or current or rate or rates or behavio#r* or knowledge or prevent* or attitude* or avoid* or perceive* or percept* or promot* or program* or change*)) OR (AB ("disrupt* behavio#r*" N5 (reduc* or decreas* or increas* or lower* or fewer or improv* or enhance* or extend* or develop* or prevalen* or daily or weekly or current or rate or rates or behavio#r* or knowledge or prevent* or attitude* or avoid* or perceive* or percept* or promot* or program* or change*)) |
| S115 | (TI ("problem* behavio#r*" N5 (reduc* or decreas* or increas* or lower* or fewer or improv* or enhance* or extend* or develop* or prevalen* or daily or weekly or current or rate or rates or behavio#r* or knowledge or prevent* or attitude* or avoid* or perceive* or percept* or promot* or program* or change*)) OR (AB ("problem* behavio#r*" N5 (reduc* or decreas* or increas* or lower* or fewer or improv* or enhance* or extend* or develop* or prevalen* or daily or weekly or current or rate or rates or behavio#r* or knowledge or prevent* or attitude* or avoid* or perceive* or percept* or promot* or program* or change*)) |
| S116 | (TI ("volatile behavio#r*" N5 (reduc* or decreas* or increas* or lower* or fewer or improv* or enhance* or extend* or develop* or prevalen* or daily or weekly or current or rate or rates or behavio#r* or knowledge or prevent* or attitude* or avoid* or perceive* or percept* or promot* or program* or change*)) OR (AB ("volatile behavio#r*" N5 (reduc* or decreas* or increas* or lower* or fewer or improv* or enhance* or extend* or develop* or prevalen* or daily or weekly or current or rate or rates or behavio#r* or knowledge or prevent* or attitude* or avoid* or perceive* or percept* or promot* or program* or change*)) |
| S117 | (TI ("volatile behavio#r*" N5 (reduc* or decreas* or increas* or lower* or fewer or improv* or enhance* or extend* or develop* or prevalen* or daily or weekly or current or rate or rates or behavio#r* or knowledge or prevent* or attitude* or avoid* or perceive* or percept* or promot* or program* or change*)) OR (AB ("volatile behavio#r*" N5 (reduc* or decreas* or increas* or lower* or fewer or improv* or enhance* or extend* or develop* or prevalen* or daily or weekly or current or rate or rates or behavio#r* or knowledge or prevent* or attitude* or avoid* or perceive* or percept* or promot* or program* or change*)) |
| S118 | (TI ((antisocial or "anti-social") N5 (reduc* or decreas* or increas* or lower* or fewer or improv* or enhance* or extend* or develop* or prevalen* or daily or weekly or current or rate or rates or behavio#r* or knowledge or prevent* or attitude* or avoid* or perceive* or percept* or promot* or program* or change*))) OR (AB ((antisocial or "anti-social") N5 (reduc* or decreas* or increas* or lower* or fewer or improv* or enhance* or extend* or develop* or prevalen* or daily or weekly or current or rate or rates or behavio#r* or knowledge or prevent* or attitude* or avoid* or perceive* or percept* or promot* or program* or change*))) |
| S119 | (TI (perpetrat* N5 (reduc* or decreas* or increas* or lower* or fewer or improv* or enhance* or extend* or develop* or prevalen* or daily or weekly or current or rate or rates or behavio#r* or knowledge or prevent* or attitude* or avoid* or perceive* or percept* or promot* or program* or change*)) OR (AB (perpetrat* N5 (reduc* or decreas* or increas* or lower* or fewer or improv* or enhance* or extend* or develop* or prevalen* or daily or weekly or current or rate or rates or behavio#r* or knowledge or prevent* or attitude* or avoid* or perceive* or percept* or promot* or program* or change*)) |
| S120 | (TI (bully* N5 (reduc* or decreas* or increas* or lower* or fewer or improv* or enhance* or extend* or develop* or prevalen* or daily or weekly or current or rate or rates or behavio#r* or knowledge or prevent* or attitude* or avoid* or perceive* or percept* or promot* or program* or change*)) OR (AB (bully* N5 (reduc* or decreas* or increas* or lower* or fewer or improv* or enhance* or extend* or develop* or prevalen* or daily or weekly or current or rate or rates or behavio#r* or knowledge or prevent* or attitude* or avoid* or perceive* or percept* or promot* or program* or change*)) |
| S121 | (TI (victim* N5 (reduc* or decreas* or increas* or lower* or fewer or improv* or enhance* or extend* or develop* or prevalen* or daily or weekly or current or rate or rates or behavio#r* or knowledge or prevent* or attitude* or avoid* or perceive* or percept* or promot* or program* or change*)) OR (AB (victim* N5 (reduc* or decreas* or increas* or lower* or fewer or improv* or enhance* or extend* or develop* or prevalen* or daily or weekly or current or rate or rates or behavio#r* or knowledge or prevent* or attitude* or avoid* or perceive* or percept* or promot* or program* or change*)) |
| S122 | (TI (cyberbully* N5 (reduc* or decreas* or increas* or lower* or fewer or improv* or enhance* or extend* or develop* or prevalen* or daily or weekly or current or rate or rates or behavio#r* or knowledge or prevent* or attitude* or avoid* or perceive* or percept* or promot* or program* or change*)) OR (AB (cyberbully* N5 (reduc* or decreas* or increas* or lower* or fewer or improv* or enhance* or extend* or develop* or prevalen* or daily or weekly or current or rate or rates or behavio#r* or knowledge or prevent* or attitude* or avoid* or perceive* or percept* or promot* or program* or change*)) |
| S123 | (TI (conflict* N5 (reduc* or decreas* or increas* or lower* or fewer or improv* or enhance* or extend* or develop* or prevalen* or daily or weekly or current or rate or rates or behavio#r* or knowledge or prevent* or attitude* or avoid* or perceive* or percept* or promot* or program* or change*)) OR (AB (conflict* N5 (reduc* or decreas* or increas* or lower* or fewer or improv* or enhance* or extend* or develop* or prevalen* or daily or weekly or current or rate or rates or behavio#r* or knowledge or prevent* or attitude* or avoid* or perceive* or percept* or promot* or program* or change*)) |
| S124 | (TI ("positive behavio#r*" N5 (reduc* or decreas* or increas* or lower* or fewer or improv* or enhance* or extend* or develop* or prevalen* or daily or weekly or current or rate or rates or behavio#r* or knowledge or prevent* or attitude* or avoid* or perceive* or percept* or promot* or program* or change*)) OR (AB ("positive behavio#r*" N5 (reduc* or decreas* or increas* or lower* or fewer or improv* or enhance* or extend* or develop* or prevalen* or daily or weekly or current or rate or rates or behavio#r* or knowledge or prevent* or attitude* or avoid* or perceive* or percept* or promot* or program* or change*)) |
| S125 | (TI ("improv* behavio#r*" N5 (reduc* or decreas* or increas* or lower* or fewer or improv* or enhance* or extend* or develop* or prevalen* or daily or weekly or current or rate or rates or behavio#r* or knowledge or prevent* or attitude* or avoid* or perceive* or percept* or promot* or program* or change*)) OR (AB ("improv* behavio#r*" N5 (reduc* or decreas* or increas* or lower* or fewer or improv* or enhance* or extend* or develop* or prevalen* or daily or weekly or current or rate or rates or behavio#r* or knowledge or prevent* or attitude* or avoid* or perceive* or percept* or promot* or program* or change*)) |
| S126 | (TI ("social behavio#r*" N5 (reduc* or decreas* or increas* or lower* or fewer or improv* or enhance* or extend* or develop* or prevalen* or daily or weekly or current or rate or rates or behavio#r* or knowledge or prevent* or attitude* or avoid* or perceive* or percept* or promot* or program* or change*)) OR (AB ("social behavio#r*" N5 (reduc* or decreas* or increas* or lower* or fewer or improv* or enhance* or extend* or develop* or prevalen* or daily or weekly or current or rate or rates or behavio#r* or knowledge or prevent* or attitude* or avoid* or perceive* or percept* or promot* or program* or change*)) |
| S127 | (TI (("social-emotion*" or "socio-emotion*") N5 (reduc* or decreas* or increas* or lower* or fewer or improv* or enhance* or extend* or develop* or prevalen* or daily or weekly or current or rate or rates or behavio#r* or knowledge or prevent* or attitude* or avoid* or perceive* or percept* or promot* or program* or change*))) OR (AB (("social-emotion*" or "socio-emotion*") N5 (reduc* or decreas* or increas* or lower* or fewer or improv* or enhance* or extend* or develop* or prevalen* or daily or weekly or current or rate or rates or behavio#r* or knowledge or prevent* or attitude* or avoid* or perceive* or percept* or promot* or program* or change*))) |
| S128 | (TI ("social-charact*" N5 (reduc* or decreas* or increas* or lower* or fewer or improv* or enhance* or extend* or develop* or prevalen* or daily or weekly or current or rate or rates or behavio#r* or knowledge or prevent* or attitude* or avoid* or perceive* or percept* or promot* or program* or change*)) OR (AB ("social-charact*" N5 (reduc* or decreas* or increas* or lower* or fewer or improv* or enhance* or extend* or develop* or prevalen* or daily or weekly or current or rate or rates or behavio#r* or knowledge or prevent* or attitude* or avoid* or perceive* or percept* or promot* or program* or change*)) |
| S129 | (TI ("social-inclusion" N5 (reduc* or decreas* or increas* or lower* or fewer or improv* or enhance* or extend* or develop* or prevalen* or daily or weekly or current or rate or rates or behavio#r* or knowledge or prevent* or attitude* or avoid* or perceive* or percept* or promot* or program* or change*)) OR (AB ("social-inclusion" N5 (reduc* or decreas* or increas* or lower* or fewer or improv* or enhance* or extend* or develop* or prevalen* or daily or weekly or current or rate or rates or behavio#r* or knowledge or prevent* or attitude* or avoid* or perceive* or percept* or promot* or program* or change*)) |
| S130 | (TI ("social develop*" N5 (reduc* or decreas* or increas* or lower* or fewer or improv* or enhance* or extend* or develop* or prevalen* or daily or weekly or current or rate or rates or behavio#r* or knowledge or prevent* or attitude* or avoid* or perceive* or percept* or promot* or program* or change*)) OR (AB ("social develop*" N5 (reduc* or decreas* or increas* or lower* or fewer or improv* or enhance* or extend* or develop* or prevalen* or daily or weekly or current or rate or rates or behavio#r* or knowledge or prevent* or attitude* or avoid* or perceive* or percept* or promot* or program* or change*)) |
| S131 | (TI ("social competen*" N5 (reduc* or decreas* or increas* or lower* or fewer or improv* or enhance* or extend* or develop* or prevalen* or daily or weekly or current or rate or rates or behavio#r* or knowledge or prevent* or attitude* or avoid* or perceive* or percept* or promot* or program* or change*)) OR (AB ("social competen*" N5 (reduc* or decreas* or increas* or lower* or fewer or improv* or enhance* or extend* or develop* or prevalen* or daily or weekly or current or rate or rates or behavio#r* or knowledge or prevent* or attitude* or avoid* or perceive* or percept* or promot* or program* or change*)) |
| S132 | (TI ("emotion* develop*" N5 (reduc* or decreas* or increas* or lower* or fewer or improv* or enhance* or extend* or develop* or prevalen* or daily or weekly or current or rate or rates or behavio#r* or knowledge or prevent* or attitude* or avoid* or perceive* or percept* or promot* or program* or change*)) OR (AB ("emotion* develop*" N5 (reduc* or decreas* or increas* or lower* or fewer or improv* or enhance* or extend* or develop* or prevalen* or daily or weekly or current or rate or rates or behavio#r* or knowledge or prevent* or attitude* or avoid* or perceive* or percept* or promot* or program* or change*)) |
| S133 | (TI (conduct N5 (reduc* or decreas* or increas* or lower* or fewer or improv* or enhance* or extend* or develop* or prevalen* or daily or weekly or current or rate or rates or behavio#r* or knowledge or prevent* or attitude* or avoid* or perceive* or percept* or promot* or program* or change*)) OR (AB (conduct N5 (reduc* or decreas* or increas* or lower* or fewer or improv* or enhance* or extend* or develop* or prevalen* or daily or weekly or current or rate or rates or behavio#r* or knowledge or prevent* or attitude* or avoid* or perceive* or percept* or promot* or program* or change*)) |
| S134 | (TI (respect N5 (reduc* or decreas* or increas* or lower* or fewer or improv* or enhance* or extend* or develop* or prevalen* or daily or weekly or current or rate or rates or behavio#r* or knowledge or prevent* or attitude* or avoid* or perceive* or percept* or promot* or program* or change*)) OR (AB (respect N5 (reduc* or decreas* or increas* or lower* or fewer or improv* or enhance* or extend* or develop* or prevalen* or daily or weekly or current or rate or rates or behavio#r* or knowledge or prevent* or attitude* or avoid* or perceive* or percept* or promot* or program* or change*)) |
| S135 | (TI ((safety or safe) N5 (reduc* or decreas* or increas* or lower* or fewer or improv* or enhance* or extend* or develop* or prevalen* or daily or weekly or current or rate or rates or behavio#r* or knowledge or prevent* or attitude* or avoid* or perceive* or percept* or promot* or program* or change*))) OR (AB ((safety or safe) N5 (reduc* or decreas* or increas* or lower* or fewer or improv* or enhance* or extend* or develop* or prevalen* or daily or weekly or current or rate or rates or behavio#r* or knowledge or prevent* or attitude* or avoid* or perceive* or percept* or promot* or program* or change*))) |
| S136 | S70 OR S71 OR S72 OR S73 OR S74 OR S75 OR S76 OR S77 OR S78 OR S79 OR S80 OR S81 OR S82 OR S83 OR S84 OR S85 OR S86 OR S87 OR S88 OR S89 OR S90 OR S91 OR S92 OR S93 OR S94 OR S95 OR S96 OR S97 OR S98 OR S99 OR S100 OR S101 OR S102 OR S103 OR S104 OR S105 OR S106 OR S107 OR S108 OR S109 OR S110 OR S111 OR S112 OR S113 OR S114 OR S115 OR S116 OR S117 OR S118 OR S119 OR S120 OR S121 OR S122 OR S123 OR S124 OR S125 OR S126 OR S127 OR S128 OR S129 OR S130 OR S131 OR S132 OR S133 OR S134 OR S135 |
| S137 | (TI prevent*) OR (AB prevent*) |
| S138 | (TI program*) OR (AB program*) |
| S139 | (TI intervent*) OR (AB intervent*) |
| S140 | (TI "restorative approach*") OR (AB "restorative approach*") |
| S141 | (TI "control school#") OR (AB "control school#") |
| S142 | (TI (trial or trials)) OR (AB (trial or trials)) |
| S143 | S137 OR S138 OR S139 OR S140 OR S141 OR S142 |
| S144 | S40 AND S69 AND S136 AND S143 |

## OvidSP Embase

| Database name | Embase Classic + Embase |
| --- | --- |
| Database platform | OvidSP |
| Dates of database coverage | 1947 to 2020 January 14 |
| Date searched | 16/01/2020 |
| Searched by | JF |
| Number of results | 11,214 |
| EndNote import order | 2 |
| Number of results once duplicates removed | 4746 |
| Search strategy notes | Search lines ending in a ‘/’ are subject heading searches. Search lines ending in ‘/*ab*’ are subject headings with subheadings added. The subheading is listed in square brackets at the end of the line. Search lines beginning ‘exp’ are exploded subject heading searches. Search lines ending in .ti,ab,kw. search in the title, abstract and author keywords only. Search lines ending in .jx. search in the journal title only. or/*x-y* combines search sets in the range *x-y* with Boolean operator OR. * is used for truncation of words. # is used for a compulsory wildcard. ? is used for an optional wildcard. adj*n* searches for terms within *n* words or each other. Words in square brackets [ ] are comments and are not included in the search terms. |

- 1. child/ (1924853)
  2. adolescent/ (1621937)
  3. juvenile/ (47977)
  4. boy/ (45435)
  5. girl/ (59452)
  6. school child/ (358089)
  7. "minor (person)"/ (602)
  8. student/ (116870)
  9. elementary student/ (1379)
  10. high school student/ (7435)
  11. middle school student/ (1246)
  12. exp teacher/ (40459)
  13. exp parent/ (276771)
  14. child*.ti,ab,kw. (1883734)
  15. boy?.ti,ab,kw. (207682)
  16. girl?.ti,ab,kw. (211838)
  17. (schoolage or (school adj1 age)).ti,ab,kw. (19062)
  18. schoolchild*.ti,ab,kw. (17994)
  19. youngster?.ti,ab,kw. (3648)
  20. minor?.ti,ab,kw. (299831)
  21. (pupil* or student*).ti,ab,kw. not (pupil/ or exp health student/) [removes items about eyes] (316157)
  22. prepubescen*.ti,ab,kw. (1190)
  23. pubescent*.ti,ab,kw. (1156)
  24. adolescent*.ti,ab,kw. (333579)
  25. juvenil*.ti,ab,kw. (105349)
  26. underage*.ti,ab,kw. (1540)
  27. (preteen* or pre-teen*).ti,ab,kw. (603)
  28. (teen or teens).ti,ab,kw. (14289)
  29. teenage*.ti,ab,kw. (29274)
  30. (youth or youths).ti,ab,kw. (89943)
  31. young person*.ti,ab,kw. (5955)
  32. young people*.ti,ab,kw. (39714)
  33. (kid or kids).ti,ab,kw. (10928)
  34. (transition adj4 adult*).ti,ab,kw. (6628)
  35. emerging adult*.ti,ab,kw. (2772)
  36. young adult*.ti,ab,kw. (125460)
  37. parent?.ti,ab,kw. (347208)
  38. mother?.ti,ab,kw. (292322)
  39. father?.ti,ab,kw. (57300)
  40. guardian?.ti,ab,kw. (9552)
  41. teacher?.ti,ab,kw. (54468)
  42. child*.jx. (197606)
  43. adolescen*.jx. (57139)
  44. youth*.jx. (2651)
  45. school*.jx. (25215)
  46. (("5" or "6" or "7" or "8" or "9" or "10" or "11" or "12" or "13" or "14" or "15" or "16" or "17" or "18") adj (year* old or year* of age)).ti,ab,kw. (315749)
  47. ((five or six or seven or eight or nine or ten or eleven or twelve or thirteen or fourteen or fifteen or sixteen or seventeen or eighteen) adj (year* old or year* of age)).ti,ab,kw. (27403)
  48. (age* adj ("5" or "6" or "7" or "8" or "9" or "10" or "11" or "12" or "13" or "14" or "15" or "16" or "17" or "18") adj year*).ti,ab,kw. (68681)
  49. (age* adj (five or six or seven or eight or nine or ten or eleven or twelve or thirteen or fourteen or fifteen or sixteen or seventeen or eighteen) adj year*).ti,ab,kw. (1206)
  50. ((primary or secondary or year) adj1 ("1" or "2" or "3" or "4" or "5" or "6" or "7" or "8" or "9" or "10" or "11" or "12" or "13")).ti,ab,kw. (883401)
  51. (grade? adj1 (first or second or third or fourth or fifth or sixth or seventh or eighth or ninth or tenth or eleventh or twelfth)).ti,ab,kw. (12184)
  52. or/1-51 (5335063)
  53. school/ (75254)
  54. high school/ (20068)
  55. middle school/ (1636)
  56. primary school/ (12943)
  57. exp school health service/ (22564)
  58. or/53-57 (122436)
  59. "organization and management"/ (414459)
  60. 58 and 59 (7384)
  61. (school? adj5 (based or level or wide or led or ethos or environment or organi?ation* or climate or toolkit? or approach* or action group? or practice? or whole or health* or leader* or cultur* or manag* or governance or system? or communit*)).ti,ab,kw. (73613)
  62. schoolwide.ti,ab,kw. (104)
  63. restor* justice.ti,ab,kw. (172)
  64. school?.ti,ab,kw. (342406)
  65. or/53-56,64 (356865)
  66. "peer-to-peer".ti,ab. (1832)
  67. peer pressure/ (2331)
  68. (peer adj2 (led or leader* or participat* or mediat* or helper? or resistan* or wide or action? or influen*)).ti,ab,kw. (4730)
  69. (relationship* adj2 (led or leader* or participat* or mediat* or helper? or resistan* or wide or action? or influen*)).ti,ab,kw. (17025)
  70. community care/ (59606)
  71. community integration/ (979)
  72. community program/ (2690)
  73. community participation/ (2067)
  74. ((community or communities) adj2 (led or leader* or participat* or mediat* or helper? or resistan* or wide or action? or influen* or relationship*)).ti,ab,kw. (24311)
  75. (pupil? adj2 (led or leader* or participat* or mediat* or helper? or resistan* or wide or action? or influen* or relationship*)).ti,ab,kw. not pupil/ [removes items about eyes] (402)
  76. ((child or children) adj2 (led or leader* or participat* or mediat* or helper? or resistan* or wide or action? or influen*)).ti,ab,kw. (16950)
  77. ((young people or young person) adj2 (led or leader* or participat* or mediat* or helper? or resistan* or wide or action? or influen*)).ti,ab,kw. (406)
  78. (teen* adj2 (led or leader* or participat* or mediat* or helper? or resistan* or wide or action? or influen*)).ti,ab,kw. (498)
  79. (adolescent* adj2 (led or leader* or participat* or mediat* or helper? or resistan* or wide or action? or influen*)).ti,ab,kw. (5327)
  80. (youth? adj2 (led or leader* or participat* or mediat* or helper? or resistan* or wide or action? or influen*)).ti,ab,kw. (2264)
  81. (student? adj2 (led or leader* or participat* or mediat* or helper? or resistan* or wide or action? or influen* or relationship*)).ti,ab,kw. (16435)
  82. (teach* adj2 (led or leader* or participat* or mediat* or helper? or resistan* or wide or action? or influen* or relationship*)).ti,ab,kw. (3840)
  83. (curricul* adj2 (led or leader* or participat* or mediat* or helper? or resistan* or wide or action? or influen* or relationship*)).ti,ab,kw. (969)
  84. (administrat* adj2 (led or leader* or participat* or mediat* or helper? or resistan* or wide or action? or influen* or relationship*)).ti,ab,kw. (6790)
  85. ((personnel or staff) adj2 (led or leader* or participat* or mediat* or helper? or resistan* or wide or action? or influen* or relationship*)).ti,ab,kw. (5790)
  86. (volunteer* adj2 (led or leader* or participat* or mediat* or helper? or resistan* or wide or action? or influen* or relationship*)).ti,ab,kw. (7465)
  87. ((advocacy or advocate) adj2 (led or leader* or participat* or mediat* or helper? or resistan* or wide or action? or influen* or relationship*)).ti,ab,kw. (970)
  88. (parent* adj2 (led or leader* or participat* or mediat* or helper? or resistan* or wide or action? or influen*)).ti,ab,kw. (11730)
  89. social environment/ (33259)
  90. organizational climate/ (302)
  91. psychosocial environment/ (4240)
  92. social support/ (88163)
  93. social learning/ (2785)
  94. (environment* adj2 (led or leader* or participat* or mediat* or helper? or resistan* or wide or action? or influen* or relationship*)).ti,ab,kw. (33410)
  95. (socio-ecolog* adj2 (led or leader* or participat* or mediat* or helper? or resistan* or wide or action? or influen* or relationship*)).ti,ab,kw. (33)
  96. (learn* adj2 (led or leader* or participat* or mediat* or helper? or resistan* or wide or action? or influen* or relationship*)).ti,ab,kw. (7910)
  97. ((policy or policies) adj2 (led or leader* or participat* or mediat* or helper? or resistan* or wide or action? or influen* or relationship*)).ti,ab,kw. (6533)
  98. or/66-97 (343749)
  99. 65 and 98 (31135)
  100. or/60-63,99 (98094)
  101. prevention/ (278379)
  102. primary prevention/ (39921)
  103. pc.fs. (1158460)
  104. or/101-103 [prevention] (1450662)
  105. smoking prevention/ (584)
  106. smoking reduction/ (172)
  107. smoking/ (310344)
  108. adolescent smoking/ (1826)
  109. cigarette smoking/ (57222)
  110. smoking habit/ (23132)
  111. cannabis smoking/ (3331)
  112. vaping/ (1386)
  113. or/107-112 (380624)
  114. 104 and 113 (32748)
  115. ((smoking or smoked or smoke or smoker) adj5 (reduc* or decreas* or increas* or lower* or fewer or improv* or enhance* or extend* or develop* or prevalen* or daily or weekly or current or rate or rates or behavio?r* or knowledge or prevent* or attitude* or avoid* or perceive* or percept* or promot* or program* or change*)).ti,ab,kw. (107532)
  116. (tobacco adj5 (reduc* or decreas* or increas* or lower* or fewer or improv* or enhance* or extend* or develop* or prevalen* or daily or weekly or current or rate or rates or behavio?r* or knowledge or prevent* or attitude* or avoid* or perceive* or percept* or promot* or program* or change*)).ti,ab,kw. (30526)
  117. (cigarette? adj5 (reduc* or decreas* or increas* or lower* or fewer or improv* or enhance* or extend* or develop* or prevalen* or daily or weekly or current or rate or rates or behavio?r* or knowledge or prevent* or attitude* or avoid* or perceive* or percept* or promot* or program* or change*)).ti,ab,kw. (26360)
  118. (marijuana adj5 (reduc* or decreas* or increas* or lower* or fewer or improv* or enhance* or extend* or develop* or prevalen* or daily or weekly or current or rate or rates or behavio?r* or knowledge or prevent* or attitude* or avoid* or perceive* or percept* or promot* or program* or change*)).ti,ab,kw. (4594)
  119. (cannabis adj5 (reduc* or decreas* or increas* or lower* or fewer or improv* or enhance* or extend* or develop* or prevalen* or daily or weekly or current or rate or rates or behavio?r* or knowledge or prevent* or attitude* or avoid* or perceive* or percept* or promot* or program* or change*)).ti,ab,kw. (6407)
  120. (solvent? adj5 (reduc* or decreas* or increas* or lower* or fewer or improv* or enhance* or extend* or develop* or prevalen* or daily or weekly or current or rate or rates or behavio?r* or knowledge or prevent* or attitude* or avoid* or perceive* or percept* or promot* or program* or change*)).ti,ab,kw. (22161)
  121. or/105-106,114-120 (184990)
  122. addiction/ (54386)
  123. exp drug dependence/ (247081)
  124. drinking behavior/ (49712)
  125. alcohol abuse/ (29569)
  126. binge drinking/ (5155)
  127. underage drinking/ (1042)
  128. substance abuse/ (52890)
  129. or/122-128 (379486)
  130. 104 and 129 (29070)
  131. (alcohol adj5 (reduc* or decreas* or increas* or lower* or fewer or improv* or enhance* or extend* or develop* or prevalen* or daily or weekly or current or rate or rates or behavio?r* or knowledge or prevent* or attitude* or avoid* or perceive* or percept* or promot* or program* or change*)).ti,ab,kw. (87757)
  132. (binge drink* adj5 (reduc* or decreas* or increas* or lower* or fewer or improv* or enhance* or extend* or develop* or prevalen* or daily or weekly or current or rate or rates or behavio?r* or knowledge or prevent* or attitude* or avoid* or perceive* or percept* or promot* or program* or change*)).ti,ab,kw. (2687)
  133. (underage drink* adj5 (reduc* or decreas* or increas* or lower* or fewer or improv* or enhance* or extend* or develop* or prevalen* or daily or weekly or current or rate or rates or behavio?r* or knowledge or prevent* or attitude* or avoid* or perceive* or percept* or promot* or program* or change*)).ti,ab,kw. (269)
  134. (substance? adj5 (reduc* or decreas* or increas* or lower* or fewer or improv* or enhance* or extend* or develop* or prevalen* or daily or weekly or current or rate or rates or behavio?r* or knowledge or prevent* or attitude* or avoid* or perceive* or percept* or promot* or program* or change*)).ti,ab,kw. (63051)
  135. (drug? adj5 (reduc* or decreas* or increas* or lower* or fewer or improv* or enhance* or extend* or develop* or prevalen* or daily or weekly or current or rate or rates or behavio?r* or knowledge or prevent* or attitude* or avoid* or perceive* or percept* or promot* or program* or change*)).ti,ab,kw. (495100)
  136. or/130-135 (650221)
  137. exp academic achievement/ (36533)
  138. (education* attain* adj5 (reduc* or decreas* or increas* or lower* or fewer or improv* or enhance* or extend* or develop* or prevalen* or daily or weekly or current or rate or rates or behavio?r* or knowledge or prevent* or attitude* or avoid* or perceive* or percept* or promot* or program* or change*)).ti,ab,kw. (2708)
  139. (academic attain* adj5 (reduc* or decreas* or increas* or lower* or fewer or improv* or enhance* or extend* or develop* or prevalen* or daily or weekly or current or rate or rates or behavio?r* or knowledge or prevent* or attitude* or avoid* or perceive* or percept* or promot* or program* or change*)).ti,ab,kw. (69)
  140. (student* attain* adj5 (reduc* or decreas* or increas* or lower* or fewer or improv* or enhance* or extend* or develop* or prevalen* or daily or weekly or current or rate or rates or behavio?r* or knowledge or prevent* or attitude* or avoid* or perceive* or percept* or promot* or program* or change*)).ti,ab,kw. (28)
  141. (education* achieve* adj5 (reduc* or decreas* or increas* or lower* or fewer or improv* or enhance* or extend* or develop* or prevalen* or daily or weekly or current or rate or rates or behavio?r* or knowledge or prevent* or attitude* or avoid* or perceive* or percept* or promot* or program* or change*)).ti,ab,kw. (490)
  142. (academic achieve* adj5 (reduc* or decreas* or increas* or lower* or fewer or improv* or enhance* or extend* or develop* or prevalen* or daily or weekly or current or rate or rates or behavio?r* or knowledge or prevent* or attitude* or avoid* or perceive* or percept* or promot* or program* or change*)).ti,ab,kw. (1662)
  143. (student* achieve* adj5 (reduc* or decreas* or increas* or lower* or fewer or improv* or enhance* or extend* or develop* or prevalen* or daily or weekly or current or rate or rates or behavio?r* or knowledge or prevent* or attitude* or avoid* or perceive* or percept* or promot* or program* or change*)).ti,ab,kw. (226)
  144. (education* perform* adj5 (reduc* or decreas* or increas* or lower* or fewer or improv* or enhance* or extend* or develop* or prevalen* or daily or weekly or current or rate or rates or behavio?r* or knowledge or prevent* or attitude* or avoid* or perceive* or percept* or promot* or program* or change*)).ti,ab,kw. (142)
  145. (academic perform* adj5 (reduc* or decreas* or increas* or lower* or fewer or improv* or enhance* or extend* or develop* or prevalen* or daily or weekly or current or rate or rates or behavio?r* or knowledge or prevent* or attitude* or avoid* or perceive* or percept* or promot* or program* or change*)).ti,ab,kw. (2243)
  146. (student* perform* adj5 (reduc* or decreas* or increas* or lower* or fewer or improv* or enhance* or extend* or develop* or prevalen* or daily or weekly or current or rate or rates or behavio?r* or knowledge or prevent* or attitude* or avoid* or perceive* or percept* or promot* or program* or change*)).ti,ab,kw. (1181)
  147. (education* outcome? adj5 (reduc* or decreas* or increas* or lower* or fewer or improv* or enhance* or extend* or develop* or prevalen* or daily or weekly or current or rate or rates or behavio?r* or knowledge or prevent* or attitude* or avoid* or perceive* or percept* or promot* or program* or change*)).ti,ab,kw. (702)
  148. (academic outcome? adj5 (reduc* or decreas* or increas* or lower* or fewer or improv* or enhance* or extend* or develop* or prevalen* or daily or weekly or current or rate or rates or behavio?r* or knowledge or prevent* or attitude* or avoid* or perceive* or percept* or promot* or program* or change*)).ti,ab,kw. (318)
  149. (student* outcome? adj5 (reduc* or decreas* or increas* or lower* or fewer or improv* or enhance* or extend* or develop* or prevalen* or daily or weekly or current or rate or rates or behavio?r* or knowledge or prevent* or attitude* or avoid* or perceive* or percept* or promot* or program* or change*)).ti,ab,kw. (230)
  150. (education* commitment adj5 (reduc* or decreas* or increas* or lower* or fewer or improv* or enhance* or extend* or develop* or prevalen* or daily or weekly or current or rate or rates or behavio?r* or knowledge or prevent* or attitude* or avoid* or perceive* or percept* or promot* or program* or change*)).ti,ab,kw. (6)
  151. (academic commitment adj5 (reduc* or decreas* or increas* or lower* or fewer or improv* or enhance* or extend* or develop* or prevalen* or daily or weekly or current or rate or rates or behavio?r* or knowledge or prevent* or attitude* or avoid* or perceive* or percept* or promot* or program* or change*)).ti,ab,kw. (9)
  152. (student* commitment adj5 (reduc* or decreas* or increas* or lower* or fewer or improv* or enhance* or extend* or develop* or prevalen* or daily or weekly or current or rate or rates or behavio?r* or knowledge or prevent* or attitude* or avoid* or perceive* or percept* or promot* or program* or change*)).ti,ab,kw. (18)
  153. (education* engage* adj5 (reduc* or decreas* or increas* or lower* or fewer or improv* or enhance* or extend* or develop* or prevalen* or daily or weekly or current or rate or rates or behavio?r* or knowledge or prevent* or attitude* or avoid* or perceive* or percept* or promot* or program* or change*)).ti,ab,kw. (18)
  154. (academic engage* adj5 (reduc* or decreas* or increas* or lower* or fewer or improv* or enhance* or extend* or develop* or prevalen* or daily or weekly or current or rate or rates or behavio?r* or knowledge or prevent* or attitude* or avoid* or perceive* or percept* or promot* or program* or change*)).ti,ab,kw. (86)
  155. (student* engage* adj5 (reduc* or decreas* or increas* or lower* or fewer or improv* or enhance* or extend* or develop* or prevalen* or daily or weekly or current or rate or rates or behavio?r* or knowledge or prevent* or attitude* or avoid* or perceive* or percept* or promot* or program* or change*)).ti,ab,kw. (648)
  156. (dropout adj5 (reduc* or decreas* or increas* or lower* or fewer or improv* or enhance* or extend* or develop* or prevalen* or daily or weekly or current or rate or rates or behavio?r* or knowledge or prevent* or attitude* or avoid* or perceive* or percept* or promot* or program* or change*)).ti,ab,kw. (5751)
  157. (absentee* adj5 (reduc* or decreas* or increas* or lower* or fewer or improv* or enhance* or extend* or develop* or prevalen* or daily or weekly or current or rate or rates or behavio?r* or knowledge or prevent* or attitude* or avoid* or perceive* or percept* or promot* or program* or change*)).ti,ab,kw. (3171)
  158. or/137-157 (52813)
  159. violence/ (46271)
  160. assault/ (6749)
  161. exposure to violence/ (1201)
  162. gun violence/ (242)
  163. physical violence/ (1646)
  164. verbal hostility/ (1905)
  165. aggression/ (58668)
  166. aggressiveness/ (11413)
  167. hostility/ (11156)
  168. physical abuse/ (4897)
  169. gender based violence/ (666)
  170. exp bullying/ (6869)
  171. exp delinquency/ (12711)
  172. disruptive behavior/ (2759)
  173. antisocial behavior/ (7660)
  174. conflict/ (25691)
  175. or/159-174 (176379)
  176. 104 and 175 (10613)
  177. ((violent or violence) adj5 (reduc* or decreas* or increas* or lower* or fewer or improv* or enhance* or extend* or develop* or prevalen* or daily or weekly or current or rate or rates or behavio?r* or knowledge or prevent* or attitude* or avoid* or perceive* or percept* or promot* or program* or change*)).ti,ab,kw. (23049)
  178. ((aggression or aggressive) adj5 (reduc* or decreas* or increas* or lower* or fewer or improv* or enhance* or extend* or develop* or prevalen* or daily or weekly or current or rate or rates or behavio?r* or knowledge or prevent* or attitude* or avoid* or perceive* or percept* or promot* or program* or change*)).ti,ab,kw. (62984)
  179. ((hostile or hostility) adj5 (reduc* or decreas* or increas* or lower* or fewer or improv* or enhance* or extend* or develop* or prevalen* or daily or weekly or current or rate or rates or behavio?r* or knowledge or prevent* or attitude* or avoid* or perceive* or percept* or promot* or program* or change*)).ti,ab,kw. (3538)
  180. (assault* adj5 (reduc* or decreas* or increas* or lower* or fewer or improv* or enhance* or extend* or develop* or prevalen* or daily or weekly or current or rate or rates or behavio?r* or knowledge or prevent* or attitude* or avoid* or perceive* or percept* or promot* or program* or change*)).ti,ab,kw. (3205)
  181. (crime adj5 (reduc* or decreas* or increas* or lower* or fewer or improv* or enhance* or extend* or develop* or prevalen* or daily or weekly or current or rate or rates or behavio?r* or knowledge or prevent* or attitude* or avoid* or perceive* or percept* or promot* or program* or change*)).ti,ab,kw. (3648)
  182. ((abuse? or abusive) adj5 (reduc* or decreas* or increas* or lower* or fewer or improv* or enhance* or extend* or develop* or prevalen* or daily or weekly or current or rate or rates or behavio?r* or knowledge or prevent* or attitude* or avoid* or perceive* or percept* or promot* or program* or change*)).ti,ab,kw. (36129)
  183. (misbehav* adj5 (reduc* or decreas* or increas* or lower* or fewer or improv* or enhance* or extend* or develop* or prevalen* or daily or weekly or current or rate or rates or behavio?r* or knowledge or prevent* or attitude* or avoid* or perceive* or percept* or promot* or program* or change*)).ti,ab,kw. (162)
  184. (threat? adj5 (reduc* or decreas* or increas* or lower* or fewer or improv* or enhance* or extend* or develop* or prevalen* or daily or weekly or current or rate or rates or behavio?r* or knowledge or prevent* or attitude* or avoid* or perceive* or percept* or promot* or program* or change*)).ti,ab,kw. (14991)
  185. (conduct adj5 (reduc* or decreas* or increas* or lower* or fewer or improv* or enhance* or extend* or develop* or prevalen* or daily or weekly or current or rate or rates or behavio?r* or knowledge or prevent* or attitude* or avoid* or perceive* or percept* or promot* or program* or change*)).ti,ab,kw. (11129)
  186. (delinquen* adj5 (reduc* or decreas* or increas* or lower* or fewer or improv* or enhance* or extend* or develop* or prevalen* or daily or weekly or current or rate or rates or behavio?r* or knowledge or prevent* or attitude* or avoid* or perceive* or percept* or promot* or program* or change*)).ti,ab,kw. (3660)
  187. (disrupt* behavio?r* adj5 (reduc* or decreas* or increas* or lower* or fewer or improv* or enhance* or extend* or develop* or prevalen* or daily or weekly or current or rate or rates or behavio?r* or knowledge or prevent* or attitude* or avoid* or perceive* or percept* or promot* or program* or change*)).ti,ab,kw. (4727)
  188. (problem* behavio?r* adj5 (reduc* or decreas* or increas* or lower* or fewer or improv* or enhance* or extend* or develop* or prevalen* or daily or weekly or current or rate or rates or behavio?r* or knowledge or prevent* or attitude* or avoid* or perceive* or percept* or promot* or program* or change*)).ti,ab,kw. (6815)
  189. (volatile behavio?r* adj5 (reduc* or decreas* or increas* or lower* or fewer or improv* or enhance* or extend* or develop* or prevalen* or daily or weekly or current or rate or rates or behavio?r* or knowledge or prevent* or attitude* or avoid* or perceive* or percept* or promot* or program* or change*)).ti,ab,kw. (25)
  190. ((antisocial or anti-social) adj5 (reduc* or decreas* or increas* or lower* or fewer or improv* or enhance* or extend* or develop* or prevalen* or daily or weekly or current or rate or rates or behavio?r* or knowledge or prevent* or attitude* or avoid* or perceive* or percept* or promot* or program* or change*)).ti,ab,kw. (6640)
  191. (perpetrat* adj5 (reduc* or decreas* or increas* or lower* or fewer or improv* or enhance* or extend* or develop* or prevalen* or daily or weekly or current or rate or rates or behavio?r* or knowledge or prevent* or attitude* or avoid* or perceive* or percept* or promot* or program* or change*)).ti,ab,kw. (2007)
192 (bully* adj5 (reduc* or decreas* or increas* or lower* or fewer or improv* or enhance* or extend* or develop* or prevalen* or daily or weekly or current or rate or rates or behavio?r* or knowledge or prevent* or attitude* or avoid* or perceive* or percept* or promot* or program* or change*)).ti,ab,kw. (2899)
193 (victim* adj5 (reduc* or decreas* or increas* or lower* or fewer or improv* or enhance* or extend* or develop* or prevalen* or daily or weekly or current or rate or rates or behavio?r* or knowledge or prevent* or attitude* or avoid* or perceive* or percept* or promot* or program* or change*)).ti,ab,kw. (10035)
194 (cyberbully* adj5 (reduc* or decreas* or increas* or lower* or fewer or improv* or enhance* or extend* or develop* or prevalen* or daily or weekly or current or rate or rates or behavio?r* or knowledge or prevent* or attitude* or avoid* or perceive* or percept* or promot* or program* or change*)).ti,ab,kw. (356)
195 (conflict* adj5 (reduc* or decreas* or increas* or lower* or fewer or improv* or enhance* or extend* or develop* or prevalen* or daily or weekly or current or rate or rates or behavio?r* or knowledge or prevent* or attitude* or avoid* or perceive* or percept* or promot* or program* or change*)).ti,ab,kw. (20596)
196 (positive behavio?r* adj5 (reduc* or decreas* or increas* or lower* or fewer or improv* or enhance* or extend* or develop* or prevalen* or daily or weekly or current or rate or rates or behavio?r* or knowledge or prevent* or attitude* or avoid* or perceive* or percept* or promot* or program* or change*)).ti,ab,kw. (1969)
197 (improv* behavio?r* adj5 (reduc* or decreas* or increas* or lower* or fewer or improv* or enhance* or extend* or develop* or prevalen* or daily or weekly or current or rate or rates or behavio?r* or knowledge or prevent* or attitude* or avoid* or perceive* or percept* or promot* or program* or change*)).ti,ab,kw. (2852)
198 (social behavio?r* adj5 (reduc* or decreas* or increas* or lower* or fewer or improv* or enhance* or extend* or develop* or prevalen* or daily or weekly or current or rate or rates or behavio?r* or knowledge or prevent* or attitude* or avoid* or perceive* or percept* or promot* or program* or change*)).ti,ab,kw. (17595)
199 ((social-emotion* or socio-emotion*) adj5 (reduc* or decreas* or increas* or lower* or fewer or improv* or enhance* or extend* or develop* or prevalen* or daily or weekly or current or rate or rates or behavio?r* or knowledge or prevent* or attitude* or avoid* or perceive* or percept* or promot* or program* or change*)).ti,ab,kw. (2486)
200 (social-charact* adj5 (reduc* or decreas* or increas* or lower* or fewer or improv* or enhance* or extend* or develop* or prevalen* or daily or weekly or current or rate or rates or behavio?r* or knowledge or prevent* or attitude* or avoid* or perceive* or percept* or promot* or program* or change*)).ti,ab,kw. (282)
201 (social-inclusion adj5 (reduc* or decreas* or increas* or lower* or fewer or improv* or enhance* or extend* or develop* or prevalen* or daily or weekly or current or rate or rates or behavio?r* or knowledge or prevent* or attitude* or avoid* or perceive* or percept* or promot* or program* or change*)).ti,ab,kw. (540)
202 (social develop* adj5 (reduc* or decreas* or increas* or lower* or fewer or improv* or enhance* or extend* or develop* or prevalen* or daily or weekly or current or rate or rates or behavio?r* or knowledge or prevent* or attitude* or avoid* or perceive* or percept* or promot* or program* or change*)).ti,ab,kw. (4167)
203 (social competen* adj5 (reduc* or decreas* or increas* or lower* or fewer or improv* or enhance* or extend* or develop* or prevalen* or daily or weekly or current or rate or rates or behavio?r* or knowledge or prevent* or attitude* or avoid* or perceive* or percept* or promot* or program* or change*)).ti,ab,kw. (1482)
204 (emotion* develop* adj5 (reduc* or decreas* or increas* or lower* or fewer or improv* or enhance* or extend* or develop* or prevalen* or daily or weekly or current or rate or rates or behavio?r* or knowledge or prevent* or attitude* or avoid* or perceive* or percept* or promot* or program* or change*)).ti,ab,kw. (2363)
205 (conduct adj5 (reduc* or decreas* or increas* or lower* or fewer or improv* or enhance* or extend* or develop* or prevalen* or daily or weekly or current or rate or rates or behavio?r* or knowledge or prevent* or attitude* or avoid* or perceive* or percept* or promot* or program* or change*)).ti,ab,kw. (11129)
206 (respect adj5 (reduc* or decreas* or increas* or lower* or fewer or improv* or enhance* or extend* or develop* or prevalen* or daily or weekly or current or rate or rates or behavio?r* or knowledge or prevent* or attitude* or avoid* or perceive* or percept* or promot* or program* or change*)).ti,ab,kw. (50004)
207 ((safety or safe) adj5 (reduc* or decreas* or increas* or lower* or fewer or improv* or enhance* or extend* or develop* or prevalen* or daily or weekly or current or rate or rates or behavio?r* or knowledge or prevent* or attitude* or avoid* or perceive* or percept* or promot* or program* or change*)).ti,ab,kw. (180323)
208 or/176-207 (454521)
209 exp program evaluation/ (23010)
210 evaluation study/ (43577)
211 validation study/ (81419)
212 exp clinical trial/ (1479089)
213 prevent*.ti,ab,kw. (1886299)
214 program*.ti,ab,kw. (1147492)
215 intervent*.ti,ab,kw. (1367046)
216 restorative approach*.ti,ab,kw. (225)
217 control school?.ti,ab,kw. (987)
218 (trial or trials).ti,ab,kw. (1436452)
219 or/209-218 (5760394)
220 121 or 136 or 158 or 208 (1266009)
221 52 and 100 and 219 and 220 (11214)

## OvidSP Global Health

| Database name | Global Health |
| --- | --- |
| Database platform | OvidSP |
| Dates of database coverage | 1910 to 2020 Week 01 |
| Date searched | 16/01/2020 |
| Searched by | JF |
| Number of results | 3512 |
| EndNote import order | 5 |
| Number of results once duplicates removed | 1030 |
| Search strategy notes | Search lines ending in a ‘/’ are subject heading searches. Search lines ending in ‘/*ab*’ are subject headings with subheadings added. The subheading is listed in square brackets at the end of the line. Search lines beginning ‘exp’ are exploded subject heading searches. Search lines ending in .ti,ab. search in the title and abstract only. Search lines ending in .jx. search in the journal title only. or/*x-y* combines search sets in the range *x-y* with Boolean operator OR. * is used for truncation of words. # is used for a compulsory wildcard. ? is used for an optional wildcard. adj*n* searches for terms within *n* words or each other. Words in square brackets [ ] are comments and are not included in the search terms. |

1. children/ (352537)
2. boys/ (23850)
3. girls/ (29322)
4. school children/ (29793)
5. adolescents/ (61506)
6. youth/ (15987)
7. students/ (20915)
8. high school students/ (4390)
9. junior high school students/ (249)
10. pupils/ (109)
11. teachers/ (2731)
12. parents/ (8659)
13. fathers/ (1491)
14. mothers/ (34672)
15. child*.ti,ab. (358790)
16. boy?.ti,ab. (42241)
17. girl?.ti,ab. (44958)
18. (schoolage or (school adj1 age)).ti,ab. (4792)
19. schoolchild*.ti,ab. (10657)
20. youngster?.ti,ab. (525)
21. minor?.ti,ab. (32783)
22. (pupil* or student*).ti,ab. not (exp eyes/ or students/) (46068)
23. prepubescen*.ti,ab. (144)
24. pubescent*.ti,ab. (226)
25. adolescent*.ti,ab. (57874)
26. juvenil*.ti,ab. (8934)
27. underage*.ti,ab. (502)
28. (preteen* or pre-teen*).ti,ab. (127)
29. (teen or teens).ti,ab. (2693)
30. teenage*.ti,ab. (5196)
31. (youth or youths).ti,ab. (20724)
32. young person*.ti,ab. (1002)
33. young people*.ti,ab. (8854)
34. (kid or kids).ti,ab. (2319)
35. (transition adj4 adult*).ti,ab. (608)
36. emerging adult*.ti,ab. (727)
37. young adult.ti,ab. (5471)
38. parent?.ti,ab. (50149)
39. mother?.ti,ab. (77769)
40. father?.ti,ab. (8191)
41. guardian?.ti,ab. (2002)
42. teacher?.ti,ab. (11679)
43. child*.jx. (21928)
44. adolescen*.jx. (6247)
45. youth.jx. (370)
46. school*.jx. (2888)
47. (("5" or "6" or "7" or "8" or "9" or "10" or "11" or "12" or "13" or "14" or "15" or "16" or "17" or "18") adj (year* old or year* of age)).ti,ab. (63962)
48. ((five or six or seven or eight or nine or ten or eleven or twelve or thirteen or fourteen or fifteen or sixteen or seventeen or eighteen) adj (year* old or year* of age)).ti,ab. (4775)
49. (age* adj ("5" or "6" or "7" or "8" or "9" or "10" or "11" or "12" or "13" or "14" or "15" or "16" or "17" or "18") adj year*).ti,ab. (18144)
50. (age* adj (five or six or seven or eight or nine or ten or eleven or twelve or thirteen or fourteen or fifteen or sixteen or seventeen or eighteen) adj year*).ti,ab. (282)
51. ((primary or secondary or year) adj1 ("1" or "2" or "3" or "4" or "5" or "6" or "7" or "8" or "9" or "10" or "11" or "12" or "13")).ti,ab. (94844)
52. (grade? adj1 (first or second or third or fourth or fifth or sixth or seventh or eighth or ninth or tenth or eleventh or twelfth)).ti,ab. (3184)
53. or/1-52 [POPULATION] (666546)
54. schools/ (16808)
55. elementary schools/ (780)
56. high schools/ (581)
57. private schools/ (299)
58. public schools/ (918)
59. school health services/ (207)
60. student teacher relationships/ (96)
61. exp management/ or exp administration/ (64708)
62. or/54-59 (18901)
63. 61 and 62 (394)
64. (school? adj5 (based or level or wide or led or ethos or environment or organi?ation* or climate or toolkit? or approach* or action group? or practice? or whole or health* or leader* or cultur* or manag* or governance or system? or communit*)).ti,ab. (23985)
65. schoolwide.ti,ab. (21)
66. restor* justice.ti,ab. (12)
67. school?.ti,ab. (93088)
68. or/54-58,67 (93455)
69. "peer-to-peer".ti,ab. (192)
70. peer relationships/ or peer influence/ (2668)
71. (peer adj2 (led or leader* or participat* or mediat* or helper? or resistan* or wide or action? or influen*)).ti,ab. (1162)
72. (relationship* adj2 (led or leader* or participat* or mediat* or helper? or resistan* or wide or action? or influen*)).ti,ab. (2622)
73. community involvement/ (3925)
74. ((community or communities) adj2 (led or leader* or participat* or mediat* or helper? or resistan* or wide or action? or influen* or relationship*)).ti,ab. (8584)
75. (pupil? adj2 (led or leader* or participat* or mediat* or helper? or resistan* or wide or action? or influen* or relationship*)).ti,ab. not exp eyes/ (122)
76. student participation/ (49)
77. ((child or children) adj2 (led or leader* or participat* or mediat* or helper? or resistan* or wide or action? or influen*)).ti,ab. (4582)
78. ((young people or young person) adj2 (led or leader* or participat* or mediat* or helper? or resistan* or wide or action? or influen*)).ti,ab. (119)
79. (teen* adj2 (led or leader* or participat* or mediat* or helper? or resistan* or wide or action? or influen*)).ti,ab. (126)
80. (adolescent* adj2 (led or leader* or participat* or mediat* or helper? or resistan* or wide or action? or influen*)).ti,ab. (1465)
81. (youth? adj2 (led or leader* or participat* or mediat* or helper? or resistan* or wide or action? or influen*)).ti,ab. (676)
82. (student? adj2 (led or leader* or participat* or mediat* or helper? or resistan* or wide or action? or influen*)).ti,ab. (2340)
83. (teach* adj2 (led or leader* or participat* or mediat* or helper? or resistan* or wide or action? or influen* or relationship*)).ti,ab. (670)
84. (curricul* adj2 (led or leader* or participat* or mediat* or helper? or resistan* or wide or action? or influen* or relationship*)).ti,ab. (92)
85. (administrat* adj2 (led or leader* or participat* or mediat* or helper? or resistan* or wide or action? or influen* or relationship*)).ti,ab. (925)
86. ((personnel or staff) adj2 (led or leader* or participat* or mediat* or helper? or resistan* or wide or action? or influen* or relationship*)).ti,ab. (733)
87. (volunteer* adj2 (led or leader* or participat* or mediat* or helper? or resistan* or wide or action? or influen* or relationship*)).ti,ab. (1074)
88. ((advocacy or advocate) adj2 (led or leader* or participat* or mediat* or helper? or resistan* or wide or action? or influen* or relationship*)).ti,ab. (187)
89. (parent* adj2 (led or leader* or participat* or mediat* or helper? or resistan* or wide or action?)).ti,ab. (1128)
90. parent participation/ (68)
91. social environment/ (1514)
92. (environment* adj2 (led or leader* or participat* or mediat* or helper? or resistan* or wide or action? or influen* or relationship*)).ti,ab. (6588)
93. (socio-ecolog* adj2 (led or leader* or participat* or mediat* or helper? or resistan* or wide or action? or influen* or relationship*)).ti,ab. (18)
94. (learn* adj2 (led or leader* or participat* or mediat* or helper? or resistan* or wide or action? or influen* or relationship*)).ti,ab. (594)
95. educational policy/ (210)
96. ((policy or policies) adj2 (led or leader* or participat* or mediat* or helper? or resistan* or wide or action? or influen* or relationship*)).ti,ab. (2480)
97. or/69-96 (40305)
98. 68 and 97 (7090)
99. or/63-66,98 [SCHOOL WIDE] (28218)
100. exp prevention/ (140155)
101. smoking cessation/ or tobacco control/ (5770)
102. tobacco smoking/ (48249)
103. vaping/ (111)
104. (102 or 103) and 100 (1947)
105. ((smoking or smoked or smoke or smoker) adj5 (reduc* or decreas* or increas* or lower* or fewer or improv* or enhance* or extend* or develop* or prevalen* or daily or weekly or current or rate or rates or behavio?r* or knowledge or prevent* or attitude* or avoid* or perceive* or percept* or promot* or program* or change*)).ti,ab. (27717)
106. (tobacco adj5 (reduc* or decreas* or increas* or lower* or fewer or improv* or enhance* or extend* or develop* or prevalen* or daily or weekly or current or rate or rates or behavio?r* or knowledge or prevent* or attitude* or avoid* or perceive* or percept* or promot* or program* or change*)).ti,ab. (9772)
107. (cigarette? adj5 (reduc* or decreas* or increas* or lower* or fewer or improv* or enhance* or extend* or develop* or prevalen* or daily or weekly or current or rate or rates or behavio?r* or knowledge or prevent* or attitude* or avoid* or perceive* or percept* or promot* or program* or change*)).ti,ab. (7334)
108. (marijuana adj5 (reduc* or decreas* or increas* or lower* or fewer or improv* or enhance* or extend* or develop* or prevalen* or daily or weekly or current or rate or rates or behavio?r* or knowledge or prevent* or attitude* or avoid* or perceive* or percept* or promot* or program* or change*)).ti,ab. (1122)
109. (cannabis adj5 (reduc* or decreas* or increas* or lower* or fewer or improv* or enhance* or extend* or develop* or prevalen* or daily or weekly or current or rate or rates or behavio?r* or knowledge or prevent* or attitude* or avoid* or perceive* or percept* or promot* or program* or change*)).ti,ab. (1154)
110. (solvent? adj5 (reduc* or decreas* or increas* or lower* or fewer or improv* or enhance* or extend* or develop* or prevalen* or daily or weekly or current or rate or rates or behavio?r* or knowledge or prevent* or attitude* or avoid* or perceive* or percept* or promot* or program* or change*)).ti,ab. (2744)
111. or/101,104-110 [SMOKING] (40440)
112. exp substance abuse/ (24573)
113. alcohol intake/ (27458)
114. alcoholism/ (10533)
115. exp addiction/ (6898)
116. (or/112-115) and 100 (3236)
117. (alcohol adj5 (reduc* or decreas* or increas* or lower* or fewer or improv* or enhance* or extend* or develop* or prevalen* or daily or weekly or current or rate or rates or behavio?r* or knowledge or prevent* or attitude* or avoid* or perceive* or percept* or promot* or program* or change*)).ti,ab. (19260)
118. (binge drink* adj5 (reduc* or decreas* or increas* or lower* or fewer or improv* or enhance* or extend* or develop* or prevalen* or daily or weekly or current or rate or rates or behavio?r* or knowledge or prevent* or attitude* or avoid* or perceive* or percept* or promot* or program* or change*)).ti,ab. (882)
119. (underage drink* adj5 (reduc* or decreas* or increas* or lower* or fewer or improv* or enhance* or extend* or develop* or prevalen* or daily or weekly or current or rate or rates or behavio?r* or knowledge or prevent* or attitude* or avoid* or perceive* or percept* or promot* or program* or change*)).ti,ab. (114)
120. (substance? adj5 (reduc* or decreas* or increas* or lower* or fewer or improv* or enhance* or extend* or develop* or prevalen* or daily or weekly or current or rate or rates or behavio?r* or knowledge or prevent* or attitude* or avoid* or perceive* or percept* or promot* or program* or change*)).ti,ab. (13267)
121. (drug? adj5 (reduc* or decreas* or increas* or lower* or fewer or improv* or enhance* or extend* or develop* or prevalen* or daily or weekly or current or rate or rates or behavio?r* or knowledge or prevent* or attitude* or avoid* or perceive* or percept* or promot* or program* or change*)).ti,ab. (59488)
122. or/116-121 [DRUG ABUSE] (90819)
123. exp educational performance/ (3372)
124. educational attendance/ (1311)
125. (education* attain* adj5 (reduc* or decreas* or increas* or lower* or fewer or improv* or enhance* or extend* or develop* or prevalen* or daily or weekly or current or rate or rates or behavio?r* or knowledge or prevent* or attitude* or avoid* or perceive* or percept* or promot* or program* or change*)).ti,ab. (924)
126. (academic attain* adj5 (reduc* or decreas* or increas* or lower* or fewer or improv* or enhance* or extend* or develop* or prevalen* or daily or weekly or current or rate or rates or behavio?r* or knowledge or prevent* or attitude* or avoid* or perceive* or percept* or promot* or program* or change*)).ti,ab. (13)
127. (student* attain* adj5 (reduc* or decreas* or increas* or lower* or fewer or improv* or enhance* or extend* or develop* or prevalen* or daily or weekly or current or rate or rates or behavio?r* or knowledge or prevent* or attitude* or avoid* or perceive* or percept* or promot* or program* or change*)).ti,ab. (2)
128. (education* achieve* adj5 (reduc* or decreas* or increas* or lower* or fewer or improv* or enhance* or extend* or develop* or prevalen* or daily or weekly or current or rate or rates or behavio?r* or knowledge or prevent* or attitude* or avoid* or perceive* or percept* or promot* or program* or change*)).ti,ab. (114)
129. (academic achieve* adj5 (reduc* or decreas* or increas* or lower* or fewer or improv* or enhance* or extend* or develop* or prevalen* or daily or weekly or current or rate or rates or behavio?r* or knowledge or prevent* or attitude* or avoid* or perceive* or percept* or promot* or program* or change*)).ti,ab. (260)
130. (student* achieve* adj5 (reduc* or decreas* or increas* or lower* or fewer or improv* or enhance* or extend* or develop* or prevalen* or daily or weekly or current or rate or rates or behavio?r* or knowledge or prevent* or attitude* or avoid* or perceive* or percept* or promot* or program* or change*)).ti,ab. (26)
131. (education* perform* adj5 (reduc* or decreas* or increas* or lower* or fewer or improv* or enhance* or extend* or develop* or prevalen* or daily or weekly or current or rate or rates or behavio?r* or knowledge or prevent* or attitude* or avoid* or perceive* or percept* or promot* or program* or change*)).ti,ab. (30)
132. (academic perform* adj5 (reduc* or decreas* or increas* or lower* or fewer or improv* or enhance* or extend* or develop* or prevalen* or daily or weekly or current or rate or rates or behavio?r* or knowledge or prevent* or attitude* or avoid* or perceive* or percept* or promot* or program* or change*)).ti,ab. (353)
133. (student* perform* adj5 (reduc* or decreas* or increas* or lower* or fewer or improv* or enhance* or extend* or develop* or prevalen* or daily or weekly or current or rate or rates or behavio?r* or knowledge or prevent* or attitude* or avoid* or perceive* or percept* or promot* or program* or change*)).ti,ab. (57)
134. (education* outcome? adj5 (reduc* or decreas* or increas* or lower* or fewer or improv* or enhance* or extend* or develop* or prevalen* or daily or weekly or current or rate or rates or behavio?r* or knowledge or prevent* or attitude* or avoid* or perceive* or percept* or promot* or program* or change*)).ti,ab. (126)
135. (academic outcome? adj5 (reduc* or decreas* or increas* or lower* or fewer or improv* or enhance* or extend* or develop* or prevalen* or daily or weekly or current or rate or rates or behavio?r* or knowledge or prevent* or attitude* or avoid* or perceive* or percept* or promot* or program* or change*)).ti,ab. (44)
136. (student* outcome? adj5 (reduc* or decreas* or increas* or lower* or fewer or improv* or enhance* or extend* or develop* or prevalen* or daily or weekly or current or rate or rates or behavio?r* or knowledge or prevent* or attitude* or avoid* or perceive* or percept* or promot* or program* or change*)).ti,ab. (27)
137. (education* commitment adj5 (reduc* or decreas* or increas* or lower* or fewer or improv* or enhance* or extend* or develop* or prevalen* or daily or weekly or current or rate or rates or behavio?r* or knowledge or prevent* or attitude* or avoid* or perceive* or percept* or promot* or program* or change*)).ti,ab. (2)
138. (academic commitment adj5 (reduc* or decreas* or increas* or lower* or fewer or improv* or enhance* or extend* or develop* or prevalen* or daily or weekly or current or rate or rates or behavio?r* or knowledge or prevent* or attitude* or avoid* or perceive* or percept* or promot* or program* or change*)).ti,ab. (1)
139. (student* commitment adj5 (reduc* or decreas* or increas* or lower* or fewer or improv* or enhance* or extend* or develop* or prevalen* or daily or weekly or current or rate or rates or behavio?r* or knowledge or prevent* or attitude* or avoid* or perceive* or percept* or promot* or program* or change*)).ti,ab. (2)
140 (education* engage* adj5 (reduc* or decreas* or increas* or lower* or fewer or improv* or enhance* or extend* or develop* or prevalen* or daily or weekly or current or rate or rates or behavio?r* or knowledge or prevent* or attitude* or avoid* or perceive* or percept* or promot* or program* or change*)).ti,ab. (7)
141 (academic engage* adj5 (reduc* or decreas* or increas* or lower* or fewer or improv* or enhance* or extend* or develop* or prevalen* or daily or weekly or current or rate or rates or behavio?r* or knowledge or prevent* or attitude* or avoid* or perceive* or percept* or promot* or program* or change*)).ti,ab. (9)
142 (student* engage* adj5 (reduc* or decreas* or increas* or lower* or fewer or improv* or enhance* or extend* or develop* or prevalen* or daily or weekly or current or rate or rates or behavio?r* or knowledge or prevent* or attitude* or avoid* or perceive* or percept* or promot* or program* or change*)).ti,ab. (97)
143 (dropout adj5 (reduc* or decreas* or increas* or lower* or fewer or improv* or enhance* or extend* or develop* or prevalen* or daily or weekly or current or rate or rates or behavio?r* or knowledge or prevent* or attitude* or avoid* or perceive* or percept* or promot* or program* or change*)).ti,ab. (627)
144 (absentee* adj5 (reduc* or decreas* or increas* or lower* or fewer or improv* or enhance* or extend* or develop* or prevalen* or daily or weekly or current or rate or rates or behavio?r* or knowledge or prevent* or attitude* or avoid* or perceive* or percept* or promot* or program* or change*)).ti,ab. (686)
145 or/123-144 [ACADEMIC PERFORMANCE] (7176)
146 aggressive behaviour/ (10842)
147 aggression/ (915)
148 fighting/ (90)
149 (or/146-148) and 100 (1018)
150 ((violent or violence) adj5 (reduc* or decreas* or increas* or lower* or fewer or improv* or enhance* or extend* or develop* or prevalen* or daily or weekly or current or rate or rates or behavio?r* or knowledge or prevent* or attitude* or avoid* or perceive* or percept* or promot* or program* or change*)).ti,ab. (5079)
151 ((aggression or aggressive) adj5 (reduc* or decreas* or increas* or lower* or fewer or improv* or enhance* or extend* or develop* or prevalen* or daily or weekly or current or rate or rates or behavio?r* or knowledge or prevent* or attitude* or avoid* or perceive* or percept* or promot* or program* or change*)).ti,ab. (3207)
152 ((hostile or hostility) adj5 (reduc* or decreas* or increas* or lower* or fewer or improv* or enhance* or extend* or develop* or prevalen* or daily or weekly or current or rate or rates or behavio?r* or knowledge or prevent* or attitude* or avoid* or perceive* or percept* or promot* or program* or change*)).ti,ab. (279)
153 (assault* adj5 (reduc* or decreas* or increas* or lower* or fewer or improv* or enhance* or extend* or develop* or prevalen* or daily or weekly or current or rate or rates or behavio?r* or knowledge or prevent* or attitude* or avoid* or perceive* or percept* or promot* or program* or change*)).ti,ab. (420)
154 (crime adj5 (reduc* or decreas* or increas* or lower* or fewer or improv* or enhance* or extend* or develop* or prevalen* or daily or weekly or current or rate or rates or behavio?r* or knowledge or prevent* or attitude* or avoid* or perceive* or percept* or promot* or program* or change*)).ti,ab. (667)
155 ((abuse? or abusive) adj5 (reduc* or decreas* or increas* or lower* or fewer or improv* or enhance* or extend* or develop* or prevalen* or daily or weekly or current or rate or rates or behavio?r* or knowledge or prevent* or attitude* or avoid* or perceive* or percept* or promot* or program* or change*)).ti,ab. (4558)
156 (misbehav* adj5 (reduc* or decreas* or increas* or lower* or fewer or improv* or enhance* or extend* or develop* or prevalen* or daily or weekly or current or rate or rates or behavio?r* or knowledge or prevent* or attitude* or avoid* or perceive* or percept* or promot* or program* or change*)).ti,ab. (19)
157 (threat? adj5 (reduc* or decreas* or increas* or lower* or fewer or improv* or enhance* or extend* or develop* or prevalen* or daily or weekly or current or rate or rates or behavio?r* or knowledge or prevent* or attitude* or avoid* or perceive* or percept* or promot* or program* or change*)).ti,ab. (3571)
158 (conduct adj5 (reduc* or decreas* or increas* or lower* or fewer or improv* or enhance* or extend* or develop* or prevalen* or daily or weekly or current or rate or rates or behavio?r* or knowledge or prevent* or attitude* or avoid* or perceive* or percept* or promot* or program* or change*)).ti,ab. (1680)
159 (delinquen* adj5 (reduc* or decreas* or increas* or lower* or fewer or improv* or enhance* or extend* or develop* or prevalen* or daily or weekly or current or rate or rates or behavio?r* or knowledge or prevent* or attitude* or avoid* or perceive* or percept* or promot* or program* or change*)).ti,ab. (441)
160 (disrupt* behavio?r* adj5 (reduc* or decreas* or increas* or lower* or fewer or improv* or enhance* or extend* or develop* or prevalen* or daily or weekly or current or rate or rates or behavio?r* or knowledge or prevent* or attitude* or avoid* or perceive* or percept* or promot* or program* or change*)).ti,ab. (200)
161 (problem* behavio?r* adj5 (reduc* or decreas* or increas* or lower* or fewer or improv* or enhance* or extend* or develop* or prevalen* or daily or weekly or current or rate or rates or behavio?r* or knowledge or prevent* or attitude* or avoid* or perceive* or percept* or promot* or program* or change*)).ti,ab. (655)
162 (volatile behavio?r* adj5 (reduc* or decreas* or increas* or lower* or fewer or improv* or enhance* or extend* or develop* or prevalen* or daily or weekly or current or rate or rates or behavio?r* or knowledge or prevent* or attitude* or avoid* or perceive* or percept* or promot* or program* or change*)).ti,ab. (4)
163 ((antisocial or anti-social) adj5 (reduc* or decreas* or increas* or lower* or fewer or improv* or enhance* or extend* or develop* or prevalen* or daily or weekly or current or rate or rates or behavio?r* or knowledge or prevent* or attitude* or avoid* or perceive* or percept* or promot* or program* or change*)).ti,ab. (534)
164 (perpetrat* adj5 (reduc* or decreas* or increas* or lower* or fewer or improv* or enhance* or extend* or develop* or prevalen* or daily or weekly or current or rate or rates or behavio?r* or knowledge or prevent* or attitude* or avoid* or perceive* or percept* or promot* or program* or change*)).ti,ab. (422)
165 (bully* adj5 (reduc* or decreas* or increas* or lower* or fewer or improv* or enhance* or extend* or develop* or prevalen* or daily or weekly or current or rate or rates or behavio?r* or knowledge or prevent* or attitude* or avoid* or perceive* or percept* or promot* or program* or change*)).ti,ab. (603)
166 (victim* adj5 (reduc* or decreas* or increas* or lower* or fewer or improv* or enhance* or extend* or develop* or prevalen* or daily or weekly or current or rate or rates or behavio?r* or knowledge or prevent* or attitude* or avoid* or perceive* or percept* or promot* or program* or change*)).ti,ab. (1554)
167 (cyberbully* adj5 (reduc* or decreas* or increas* or lower* or fewer or improv* or enhance* or extend* or develop* or prevalen* or daily or weekly or current or rate or rates or behavio?r* or knowledge or prevent* or attitude* or avoid* or perceive* or percept* or promot* or program* or change*)).ti,ab. (59)
168 (conflict* adj5 (reduc* or decreas* or increas* or lower* or fewer or improv* or enhance* or extend* or develop* or prevalen* or daily or weekly or current or rate or rates or behavio?r* or knowledge or prevent* or attitude* or avoid* or perceive* or percept* or promot* or program* or change*)).ti,ab. (2324)
169 (positive behavio?r* adj5 (reduc* or decreas* or increas* or lower* or fewer or improv* or enhance* or extend* or develop* or prevalen* or daily or weekly or current or rate or rates or behavio?r* or knowledge or prevent* or attitude* or avoid* or perceive* or percept* or promot* or program* or change*)).ti,ab. (361)
170 (improv* behavio?r* adj5 (reduc* or decreas* or increas* or lower* or fewer or improv* or enhance* or extend* or develop* or prevalen* or daily or weekly or current or rate or rates or behavio?r* or knowledge or prevent* or attitude* or avoid* or perceive* or percept* or promot* or program* or change*)).ti,ab. (327)
171 (social behavio?r* adj5 (reduc* or decreas* or increas* or lower* or fewer or improv* or enhance* or extend* or develop* or prevalen* or daily or weekly or current or rate or rates or behavio?r* or knowledge or prevent* or attitude* or avoid* or perceive* or percept* or promot* or program* or change*)).ti,ab. (1139)
172 ((social-emotion* or socio-emotion*) adj5 (reduc* or decreas* or increas* or lower* or fewer or improv* or enhance* or extend* or develop* or prevalen* or daily or weekly or current or rate or rates or behavio?r* or knowledge or prevent* or attitude* or avoid* or perceive* or percept* or promot* or program* or change*)).ti,ab. (268)
173 (social-charact* adj5 (reduc* or decreas* or increas* or lower* or fewer or improv* or enhance* or extend* or develop* or prevalen* or daily or weekly or current or rate or rates or behavio?r* or knowledge or prevent* or attitude* or avoid* or perceive* or percept* or promot* or program* or change*)).ti,ab. (80)
174 (social-inclusion adj5 (reduc* or decreas* or increas* or lower* or fewer or improv* or enhance* or extend* or develop* or prevalen* or daily or weekly or current or rate or rates or behavio?r* or knowledge or prevent* or attitude* or avoid* or perceive* or percept* or promot* or program* or change*)).ti,ab. (124)
175 (social develop* adj5 (reduc* or decreas* or increas* or lower* or fewer or improv* or enhance* or extend* or develop* or prevalen* or daily or weekly or current or rate or rates or behavio?r* or knowledge or prevent* or attitude* or avoid* or perceive* or percept* or promot* or program* or change*)).ti,ab. (1102)
176 (social competen* adj5 (reduc* or decreas* or increas* or lower* or fewer or improv* or enhance* or extend* or develop* or prevalen* or daily or weekly or current or rate or rates or behavio?r* or knowledge or prevent* or attitude* or avoid* or perceive* or percept* or promot* or program* or change*)).ti,ab. (90)
177 (emotion* develop* adj5 (reduc* or decreas* or increas* or lower* or fewer or improv* or enhance* or extend* or develop* or prevalen* or daily or weekly or current or rate or rates or behavio?r* or knowledge or prevent* or attitude* or avoid* or perceive* or percept* or promot* or program* or change*)).ti,ab. (219)
178 (conduct adj5 (reduc* or decreas* or increas* or lower* or fewer or improv* or enhance* or extend* or develop* or prevalen* or daily or weekly or current or rate or rates or behavio?r* or knowledge or prevent* or attitude* or avoid* or perceive* or percept* or promot* or program* or change*)).ti,ab. (1680)
179 (respect adj5 (reduc* or decreas* or increas* or lower* or fewer or improv* or enhance* or extend* or develop* or prevalen* or daily or weekly or current or rate or rates or behavio?r* or knowledge or prevent* or attitude* or avoid* or perceive* or percept* or promot* or program* or change*)).ti,ab. (5794)
180 ((safety or safe) adj5 (reduc* or decreas* or increas* or lower* or fewer or improv* or enhance* or extend* or develop* or prevalen* or daily or weekly or current or rate or rates or behavio?r* or knowledge or prevent* or attitude* or avoid* or perceive* or percept* or promot* or program* or change*)).ti,ab. (30719)
181 or/149-180 [VIOLENCE] (62225)
182 111 or 122 or 145 or 181 (187372)
183 program evaluation/ (616)
184 program effectiveness/ (56)
185 exp clinical trials/ (57244)
186 prevent*.ti,ab. (360337)
187 program*.ti,ab. (198863)
188 intervent*.ti,ab. (183203)
189 restorative approach*.ti,ab. (10)
190 control school?.ti,ab. (590)
191 (trial or trials).ti,ab. (155201)
192 or/183-191 (733756)
193 53 and 99 and 182 and 192 (3512)

## WHO International Clinical Trials Registry Platform

| Database name | International Clinical Trials Registry Platform |
| --- | --- |
| Database platform | <http://apps.who.int/trialsearch/> |
| Dates of database coverage | Complete database to date searched. |
| Date searched | 27/01/2020 |
| Searched by | JF |
| Number of results | 601 |
| EndNote import order | 21 |
| Number of results once duplicates removed | 384 |
| Search strategy notes | None |

Title: school OR schools OR schoolwide

## OvidSP PsycINFO

| Database name | PsycINFO |
| --- | --- |
| Database platform | OvidSP |
| Dates of database coverage | 1806 to January Week 1 2020 |
| Date searched | 16/01/2020 |
| Searched by | JF |
| Number of results | 17,477 |
| EndNote import order | 6 |
| Number of results once duplicates removed | 13,548 |
| Search strategy notes | Search lines ending in a ‘/’ are subject heading searches. Search lines ending in ‘/*ab*’ are subject headings with subheadings added. The subheading is listed in square brackets at the end of the line. Search lines beginning ‘exp’ are exploded subject heading searches. Search lines ending in .ti,ab. search in the title and abstract only. Search lines ending in .jx. search in the journal title only. or/*x-y* combines search sets in the range *x-y* with Boolean operator OR. * is used for truncation of words. # is used for a compulsory wildcard. ? is used for an optional wildcard. adj*n* searches for terms within *n* words or each other. Words in square brackets [ ] are comments and are not included in the search terms. |

1 early adolescence/ (1973)
2 emerging adulthood/ (2428)
3 classmates/ (308)
4 exp elementary school students/ (42956)
5 high school students/ (31268)
6 junior high school students/ (11532)
7 middle school students/ (8354)
8 elementary school teachers/ (8367)
9 high school teachers/ (3944)
10 junior high school teachers/ (1621)
11 middle school teachers/ (930)
12 parents/ (38729)
13 adoptive parents/ (1560)
14 exp fathers/ (10661)
15 homosexual parents/ (872)
16 exp mothers/ (40040)
17 single parents/ (1566)
18 child*.ti,ab. (675478)
19 boy?.ti,ab. (71505)
20 girl?.ti,ab. (70315)
21 (schoolage or (school adj1 age)).ti,ab. (13329)
22 schoolchild*.ti,ab. (3644)
23 youngster?.ti,ab. (3607)
24 minor?.ti,ab. (24596)
25 (pupil* or student*).ti,ab. not ("pupil (eye)"/ or exp students/) (329882)
26 prepubescen*.ti,ab. (376)
27 pubescent*.ti,ab. (335)
28 adolescent*.ti,ab. (206771)
29 juvenil*.ti,ab. (24927)
30 underage*.ti,ab. (1083)
31 (preteen* or pre-teen*).ti,ab. (452)
32 (teen or teens).ti,ab. (10052)
33 teenage*.ti,ab. (12970)
34 (youth or youths).ti,ab. (93496)
35 young person*.ti,ab. (2535)
36 young people*.ti,ab. (28962)
37 (kid or kids).ti,ab. (4051)
38 (transition adj4 adult*).ti,ab. (3940)
39 emerging adult*.ti,ab. (4417)
40 young adult.ti,ab. (12375)
41 parent?.ti,ab. (211833)
42 mother?.ti,ab. (117178)
43 father?.ti,ab. (45006)
44 guardian?.ti,ab. (3410)
45 teacher?.ti,ab. (178921)
46 child*.jx. (121019)
47 adolescen*.jx. (43234)
48 youth*.jx. (13832)
49 school*.jx. (26466)
50 (("5" or "6" or "7" or "8" or "9" or "10" or "11" or "12" or "13" or "14" or "15" or "16" or "17" or "18") adj (year* old or year* of age)).ti,ab. (42739)
51 ((five or six or seven or eight or nine or ten or eleven or twelve or thirteen or fourteen or fifteen or sixteen or seventeen or eighteen) adj (year* old or year* of age)).ti,ab. (6233)
52 (age* adj ("5" or "6" or "7" or "8" or "9" or "10" or "11" or "12" or "13" or "14" or "15" or "16" or "17" or "18") adj year*).ti,ab. (7454)
53 (age* adj (five or six or seven or eight or nine or ten or eleven or twelve or thirteen or fourteen or fifteen or sixteen or seventeen or eighteen) adj year*).ti,ab. (187)
54 ((primary or secondary or year) adj1 ("1" or "2" or "3" or "4" or "5" or "6" or "7" or "8" or "9" or "10" or "11" or "12" or "13")).ti,ab. (73821)
55 (grade? adj1 (first or second or third or fourth or fifth or sixth or seventh or eighth or ninth or tenth or eleventh or twelfth)).ti,ab. (30367)
56 or/1-55 [POPULATION] (1408500)
57 elementary schools/ (7690)
58 high schools/ (6717)
59 junior high schools/ (607)
60 middle schools/ (4014)
61 or/57-60 (18204)
62 management/ (13654)
63 61 and 62 (24)
64 classroom environment/ (8479)
65 school environment/ (12360)
66 classroom management/ (1430)
67 educational administration/ (5032)
68 parent school relationship/ (2764)
69 (school? adj5 (based or level or wide or led or ethos or environment or organi?ation* or climate or toolkit? or approach* or action group? or practice? or whole or health* or leader* or cultur* or manag* or governance or system? or communit*)).ti,ab. (91914)
70 schoolwide.ti,ab. (654)
71 restorative justice/ (617)
72 school?.ti,ab. (366669)
73 61 or 72 (367207)
74 peer relations/ (15967)
75 "peer-to-peer".ti,ab. (1403)
76 (peer adj2 (led or leader* or participat* or mediat* or helper? or resistan* or wide or action? or influen*)).ti,ab. (5959)
77 (relationship* adj2 (led or leader* or participat* or mediat* or helper? or resistan* or wide or action? or influen*)).ti,ab. (26721)
78 school integration/ (1236)
79 community involvement/ (4559)
80 ((community or communities) adj2 (led or leader* or participat* or mediat* or helper? or resistan* or wide or action? or influen* or relationship*)).ti,ab. (13229)
81 (pupil? adj2 (led or leader* or participat* or mediat* or helper? or resistan* or wide or action? or influen* or relationship*)).ti,ab. not "pupil (eye)"/ (850)
82 ((child or children) adj2 (led or leader* or participat* or mediat* or helper? or resistan* or wide or action? or influen*)).ti,ab. (11390)
83 ((young people or young person) adj2 (led or leader* or participat* or mediat* or helper? or resistan* or wide or action? or influen*)).ti,ab. (459)
84 (teen* adj2 (led or leader* or participat* or mediat* or helper? or resistan* or wide or action? or influen*)).ti,ab. (442)
85 (adolescent* adj2 (led or leader* or participat* or mediat* or helper? or resistan* or wide or action? or influen*)).ti,ab. (5460)
86 (youth? adj2 (led or leader* or participat* or mediat* or helper? or resistan* or wide or action? influen*)).ti,ab. (2435)
87 (student? adj2 (led or leader* or participat* or mediat* or helper? or resistan* or wide or action? or influen* or relationship*)).ti,ab. (29114)
88 (teach* adj2 (led or leader* or participat* or mediat* or helper? or resistan* or wide or action? or influen* or relationship*)).ti,ab. (17694)
89 (curricul* adj2 (led or leader* or participat* or mediat* or helper? or resistan* or wide or action? or influen* or relationship*)).ti,ab. (1204)
90 (administrat* adj2 (led or leader* or participat* or mediat* or helper? or resistan* or wide or action? or influen* or relationship*)).ti,ab. (2438)
91 ((personnel or staff) adj2 (led or leader* or participat* or mediat* or helper? or resistan* or wide or action? or influen* or relationship*)).ti,ab. (3266)
92 (volunteer* adj2 (led or leader* or participat* or mediat* or helper? or resistan* or wide or action? or influen* or relationship*)).ti,ab. (2812)
93 ((advocacy or advocate) adj2 (led or leader* or participat* or mediat* or helper? or resistan* or wide or action? or relationship*)).ti,ab. (678)
94 (parent* adj2 (led or leader* or participat* or mediat* or helper? or resistan* or wide or action? or influen*)).ti,ab. (13129)
95 social environments/ (7084)
96 organizational climate/ (11668)
97 social support/ (35317)
98 social learning/ (4756)
99 (environment* adj2 (led or leader* or participat* or mediat* or helper? or resistan* or wide or action? or influen* or relationship*)).ti,ab. (14880)
100 (socio-ecolog* adj2 (led or leader* or participat* or mediat* or helper? or resistan* or wide or action? or influen* or relationship*)).ti,ab. (25)
101 (learn* adj2 (led or leader* or participat* or mediat* or helper? or resistan* or wide or action? or influen* or relationship*)).ti,ab. (13045)
102 education policy/ (1288)
103 ((policy or policies) adj2 (led or leader* or participat* or mediat* or helper? or resistan* or wide or action? or influen* or relationship*)).ti,ab. (4670)
104 or/74-103 (226242)
105 73 and 104 (50173)
106 or/63-71,105 [SCHOOL-WIDE INTERVENTIONS] (142219)
107 prevention/ (29571)
108 smoking cessation/ (12792)
109 "tobacco use disorder"/ (213)
110 tobacco smoking/ (30478)
111 marijuana usage/ (2795)
112 electronic cigarettes/ (1135)
113 or/109-112 (33506)
114 107 and 113 (961)
115 ((smoking or smoked or smoke or smoker) adj5 (reduc* or decreas* or increas* or lower* or fewer or improv* or enhance* or extend* or develop* or prevalen* or daily or weekly or current or rate or rates or behavio?r* or knowledge or prevent* or attitude* or avoid* or perceive* or percept* or promot* or program* or change*)).ti,ab. (26062)
116 (tobacco adj5 (reduc* or decreas* or increas* or lower* or fewer or improv* or enhance* or extend* or develop* or prevalen* or daily or weekly or current or rate or rates or behavio?r* or knowledge or prevent* or attitude* or avoid* or perceive* or percept* or promot* or program* or change*)).ti,ab. (8690)
117 (cigarette? adj5 (reduc* or decreas* or increas* or lower* or fewer or improv* or enhance* or extend* or develop* or prevalen* or daily or weekly or current or rate or rates or behavio?r* or knowledge or prevent* or attitude* or avoid* or perceive* or percept* or promot* or program* or change*)).ti,ab. (7886)
118 (marijuana adj5 (reduc* or decreas* or increas* or lower* or fewer or improv* or enhance* or extend* or develop* or prevalen* or daily or weekly or current or rate or rates or behavio?r* or knowledge or prevent* or attitude* or avoid* or perceive* or percept* or promot* or program* or change*)).ti,ab. (2910)
119 (cannabis adj5 (reduc* or decreas* or increas* or lower* or fewer or improv* or enhance* or extend* or develop* or prevalen* or daily or weekly or current or rate or rates or behavio?r* or knowledge or prevent* or attitude* or avoid* or perceive* or percept* or promot* or program* or change*)).ti,ab. (3539)
120 (solvent? adj5 (reduc* or decreas* or increas* or lower* or fewer or improv* or enhance* or extend* or develop* or prevalen* or daily or weekly or current or rate or rates or behavio?r* or knowledge or prevent* or attitude* or avoid* or perceive* or percept* or promot* or program* or change*)).ti,ab. (185)
121 or/108,114-120 [SMOKING] (41459)
122 exp "substance use disorder"/ (126746)
123 underage drinking/ (701)
124 illegal drug distribution/ (757)
125 or/122-124 (127725)
126 107 and 125 (2110)
127 (alcohol adj5 (reduc* or decreas* or increas* or lower* or fewer or improv* or enhance* or extend* or develop* or prevalen* or daily or weekly or current or rate or rates or behavio?r* or knowledge or prevent* or attitude* or avoid* or perceive* or percept* or promot* or program* or change*)).ti,ab. (39226)
128 (binge drink* adj5 (reduc* or decreas* or increas* or lower* or fewer or improv* or enhance* or extend* or develop* or prevalen* or daily or weekly or current or rate or rates or behavio?r* or knowledge or prevent* or attitude* or avoid* or perceive* or percept* or promot* or program* or change*)).ti,ab. (1653)
129 (underage drink* adj5 (reduc* or decreas* or increas* or lower* or fewer or improv* or enhance* or extend* or develop* or prevalen* or daily or weekly or current or rate or rates or behavio?r* or knowledge or prevent* or attitude* or avoid* or perceive* or percept* or promot* or program* or change*)).ti,ab. (216)
130 (substance? adj5 (reduc* or decreas* or increas* or lower* or fewer or improv* or enhance* or extend* or develop* or prevalen* or daily or weekly or current or rate or rates or behavio?r* or knowledge or prevent* or attitude* or avoid* or perceive* or percept* or promot* or program* or change*)).ti,ab. (26972)
131 (drug? adj5 (reduc* or decreas* or increas* or lower* or fewer or improv* or enhance* or extend* or develop* or prevalen* or daily or weekly or current or rate or rates or behavio?r* or knowledge or prevent* or attitude* or avoid* or perceive* or percept* or promot* or program* or change*)).ti,ab. (56816)
132 or/126-131 [SUBSTANCE ABUSE] (113052)
133 exp academic achievement/ (75574)
134 academic achievement motivation/ (5149)
135 (education* attain* adj5 (reduc* or decreas* or increas* or lower* or fewer or improv* or enhance* or extend* or develop* or prevalen* or daily or weekly or current or rate or rates or behavio?r* or knowledge or prevent* or attitude* or avoid* or perceive* or percept* or promot* or program* or change*)).ti,ab. (1758)
136 (academic attain* adj5 (reduc* or decreas* or increas* or lower* or fewer or improv* or enhance* or extend* or develop* or prevalen* or daily or weekly or current or rate or rates or behavio?r* or knowledge or prevent* or attitude* or avoid* or perceive* or percept* or promot* or program* or change*)).ti,ab. (111)
137 (student* attain* adj5 (reduc* or decreas* or increas* or lower* or fewer or improv* or enhance* or extend* or develop* or prevalen* or daily or weekly or current or rate or rates or behavio?r* or knowledge or prevent* or attitude* or avoid* or perceive* or percept* or promot* or program* or change*)).ti,ab. (60)
138 (education* achieve* adj5 (reduc* or decreas* or increas* or lower* or fewer or improv* or enhance* or extend* or develop* or prevalen* or daily or weekly or current or rate or rates or behavio?r* or knowledge or prevent* or attitude* or avoid* or perceive* or percept* or promot* or program* or change*)).ti,ab. (548)
139 (academic achieve* adj5 (reduc* or decreas* or increas* or lower* or fewer or improv* or enhance* or extend* or develop* or prevalen* or daily or weekly or current or rate or rates or behavio?r* or knowledge or prevent* or attitude* or avoid* or perceive* or percept* or promot* or program* or change*)).ti,ab. (5794)
140 (student* achieve* adj5 (reduc* or decreas* or increas* or lower* or fewer or improv* or enhance* or extend* or develop* or prevalen* or daily or weekly or current or rate or rates or behavio?r* or knowledge or prevent* or attitude* or avoid* or perceive* or percept* or promot* or program* or change*)).ti,ab. (4619)
141 (education* perform* adj5 (reduc* or decreas* or increas* or lower* or fewer or improv* or enhance* or extend* or develop* or prevalen* or daily or weekly or current or rate or rates or behavio?r* or knowledge or prevent* or attitude* or avoid* or perceive* or percept* or promot* or program* or change*)).ti,ab. (153)
142 (academic perform* adj5 (reduc* or decreas* or increas* or lower* or fewer or improv* or enhance* or extend* or develop* or prevalen* or daily or weekly or current or rate or rates or behavio?r* or knowledge or prevent* or attitude* or avoid* or perceive* or percept* or promot* or program* or change*)).ti,ab. (4083)
143 (student* perform* adj5 (reduc* or decreas* or increas* or lower* or fewer or improv* or enhance* or extend* or develop* or prevalen* or daily or weekly or current or rate or rates or behavio?r* or knowledge or prevent* or attitude* or avoid* or perceive* or percept* or promot* or program* or change*)).ti,ab. (1988)
144 (education* outcome? adj5 (reduc* or decreas* or increas* or lower* or fewer or improv* or enhance* or extend* or develop* or prevalen* or daily or weekly or current or rate or rates or behavio?r* or knowledge or prevent* or attitude* or avoid* or perceive* or percept* or promot* or program* or change*)).ti,ab. (859)
145 (academic outcome? adj5 (reduc* or decreas* or increas* or lower* or fewer or improv* or enhance* or extend* or develop* or prevalen* or daily or weekly or current or rate or rates or behavio?r* or knowledge or prevent* or attitude* or avoid* or perceive* or percept* or promot* or program* or change*)).ti,ab. (762)
146 (student* outcome? adj5 (reduc* or decreas* or increas* or lower* or fewer or improv* or enhance* or extend* or develop* or prevalen* or daily or weekly or current or rate or rates or behavio?r* or knowledge or prevent* or attitude* or avoid* or perceive* or percept* or promot* or program* or change*)).ti,ab. (1106)
147 (education* commitment adj5 (reduc* or decreas* or increas* or lower* or fewer or improv* or enhance* or extend* or develop* or prevalen* or daily or weekly or current or rate or rates or behavio?r* or knowledge or prevent* or attitude* or avoid* or perceive* or percept* or promot* or program* or change*)).ti,ab. (12)
148 (academic commitment adj5 (reduc* or decreas* or increas* or lower* or fewer or improv* or enhance* or extend* or develop* or prevalen* or daily or weekly or current or rate or rates or behavio?r* or knowledge or prevent* or attitude* or avoid* or perceive* or percept* or promot* or program* or change*)).ti,ab. (12)
149 (student* commitment adj5 (reduc* or decreas* or increas* or lower* or fewer or improv* or enhance* or extend* or develop* or prevalen* or daily or weekly or current or rate or rates or behavio?r* or knowledge or prevent* or attitude* or avoid* or perceive* or percept* or promot* or program* or change*)).ti,ab. (33)
150 (education* engage* adj5 (reduc* or decreas* or increas* or lower* or fewer or improv* or enhance* or extend* or develop* or prevalen* or daily or weekly or current or rate or rates or behavio?r* or knowledge or prevent* or attitude* or avoid* or perceive* or percept* or promot* or program* or change*)).ti,ab. (26)
151 (academic engage* adj5 (reduc* or decreas* or increas* or lower* or fewer or improv* or enhance* or extend* or develop* or prevalen* or daily or weekly or current or rate or rates or behavio?r* or knowledge or prevent* or attitude* or avoid* or perceive* or percept* or promot* or program* or change*)).ti,ab. (431)
152 (student* engage* adj5 (reduc* or decreas* or increas* or lower* or fewer or improv* or enhance* or extend* or develop* or prevalen* or daily or weekly or current or rate or rates or behavio?r* or knowledge or prevent* or attitude* or avoid* or perceive* or percept* or promot* or program* or change*)).ti,ab. (1724)
153 (dropout adj5 (reduc* or decreas* or increas* or lower* or fewer or improv* or enhance* or extend* or develop* or prevalen* or daily or weekly or current or rate or rates or behavio?r* or knowledge or prevent* or attitude* or avoid* or perceive* or percept* or promot* or program* or change*)).ti,ab. (4042)
154 (absentee* adj5 (reduc* or decreas* or increas* or lower* or fewer or improv* or enhance* or extend* or develop* or prevalen* or daily or weekly or current or rate or rates or behavio?r* or knowledge or prevent* or attitude* or avoid* or perceive* or percept* or promot* or program* or change*)).ti,ab. (1629)
155 or/133-154 [ACADEMIC ACHIEVEMENT] (94996)
156 violence/ or gun violence/ or school violence/ (32338)
157 violent crime/ or physical abuse/ (7631)
158 aggressive behavior/ or threat/ (34439)
159 dating violence/ (1135)
160 exp bullying/ (9137)
161 exp juvenile delinquency/ (19069)
162 behavior problems/ (28638)
163 antisocial behavior/ (9680)
164 emotional abuse/ (2532)
165 verbal abuse/ (496)
166 conflict/ or arguments/ (23232)
167 or/156-166 (149943)
168 107 and 167 (3960)
169 ((violent or violence) adj5 (reduc* or decreas* or increas* or lower* or fewer or improv* or enhance* or extend* or develop* or prevalen* or daily or weekly or current or rate or rates or behavio?r* or knowledge or prevent* or attitude* or avoid* or perceive* or percept* or promot* or program* or change*)).ti,ab. (28711)
170 ((aggression or aggressive) adj5 (reduc* or decreas* or increas* or lower* or fewer or improv* or enhance* or extend* or develop* or prevalen* or daily or weekly or current or rate or rates or behavio?r* or knowledge or prevent* or attitude* or avoid* or perceive* or percept* or promot* or program* or change*)).ti,ab. (33985)
171 ((hostile or hostility) adj5 (reduc* or decreas* or increas* or lower* or fewer or improv* or enhance* or extend* or develop* or prevalen* or daily or weekly or current or rate or rates or behavio?r* or knowledge or prevent* or attitude* or avoid* or perceive* or percept* or promot* or program* or change*)).ti,ab. (4963)
172 (assault* adj5 (reduc* or decreas* or increas* or lower* or fewer or improv* or enhance* or extend* or develop* or prevalen* or daily or weekly or current or rate or rates or behavio?r* or knowledge or prevent* or attitude* or avoid* or perceive* or percept* or promot* or program* or change*)).ti,ab. (3359)
173 (crime adj5 (reduc* or decreas* or increas* or lower* or fewer or improv* or enhance* or extend* or develop* or prevalen* or daily or weekly or current or rate or rates or behavio?r* or knowledge or prevent* or attitude* or avoid* or perceive* or percept* or promot* or program* or change*)).ti,ab. (8500)
174 ((abuse? or abusive) adj5 (reduc* or decreas* or increas* or lower* or fewer or improv* or enhance* or extend* or develop* or prevalen* or daily or weekly or current or rate or rates or behavio?r* or knowledge or prevent* or attitude* or avoid* or perceive* or percept* or promot* or program* or change*)).ti,ab. (32014)
175 (misbehav* adj5 (reduc* or decreas* or increas* or lower* or fewer or improv* or enhance* or extend* or develop* or prevalen* or daily or weekly or current or rate or rates or behavio?r* or knowledge or prevent* or attitude* or avoid* or perceive* or percept* or promot* or program* or change*)).ti,ab. (506)
176 (threat? adj5 (reduc* or decreas* or increas* or lower* or fewer or improv* or enhance* or extend* or develop* or prevalen* or daily or weekly or current or rate or rates or behavio?r* or knowledge or prevent* or attitude* or avoid* or perceive* or percept* or promot* or program* or change*)).ti,ab. (11226)
177 (conduct adj5 (reduc* or decreas* or increas* or lower* or fewer or improv* or enhance* or extend* or develop* or prevalen* or daily or weekly or current or rate or rates or behavio?r* or knowledge or prevent* or attitude* or avoid* or perceive* or percept* or promot* or program* or change*)).ti,ab. (7780)
178 (delinquen* adj5 (reduc* or decreas* or increas* or lower* or fewer or improv* or enhance* or extend* or develop* or prevalen* or daily or weekly or current or rate or rates or behavio?r* or knowledge or prevent* or attitude* or avoid* or perceive* or percept* or promot* or program* or change*)).ti,ab. (8673)
179 (disrupt* behavio?r* adj5 (reduc* or decreas* or increas* or lower* or fewer or improv* or enhance* or extend* or develop* or prevalen* or daily or weekly or current or rate or rates or behavio?r* or knowledge or prevent* or attitude* or avoid* or perceive* or percept* or promot* or program* or change*)).ti,ab. (6109)
180 (problem* behavio?r* adj5 (reduc* or decreas* or increas* or lower* or fewer or improv* or enhance* or extend* or develop* or prevalen* or daily or weekly or current or rate or rates or behavio?r* or knowledge or prevent* or attitude* or avoid* or perceive* or percept* or promot* or program* or change*)).ti,ab. (12385)
181 (volatile behavio?r* adj5 (reduc* or decreas* or increas* or lower* or fewer or improv* or enhance* or extend* or develop* or prevalen* or daily or weekly or current or rate or rates or behavio?r* or knowledge or prevent* or attitude* or avoid* or perceive* or percept* or promot* or program* or change*)).ti,ab. (12)
182 ((antisocial or anti-social) adj5 (reduc* or decreas* or increas* or lower* or fewer or improv* or enhance* or extend* or develop* or prevalen* or daily or weekly or current or rate or rates or behavio?r* or knowledge or prevent* or attitude* or avoid* or perceive* or percept* or promot* or program* or change*)).ti,ab. (10717)
183 (perpetrat* adj5 (reduc* or decreas* or increas* or lower* or fewer or improv* or enhance* or extend* or develop* or prevalen* or daily or weekly or current or rate or rates or behavio?r* or knowledge or prevent* or attitude* or avoid* or perceive* or percept* or promot* or program* or change*)).ti,ab. (3098)
184 (bully* adj5 (reduc* or decreas* or increas* or lower* or fewer or improv* or enhance* or extend* or develop* or prevalen* or daily or weekly or current or rate or rates or behavio?r* or knowledge or prevent* or attitude* or avoid* or perceive* or percept* or promot* or program* or change*)).ti,ab. (5464)
185 (victim* adj5 (reduc* or decreas* or increas* or lower* or fewer or improv* or enhance* or extend* or develop* or prevalen* or daily or weekly or current or rate or rates or behavio?r* or knowledge or prevent* or attitude* or avoid* or perceive* or percept* or promot* or program* or change*)).ti,ab. (13225)
186 (cyberbully* adj5 (reduc* or decreas* or increas* or lower* or fewer or improv* or enhance* or extend* or develop* or prevalen* or daily or weekly or current or rate or rates or behavio?r* or knowledge or prevent* or attitude* or avoid* or perceive* or percept* or promot* or program* or change*)).ti,ab. (837)
187 (conflict* adj5 (reduc* or decreas* or increas* or lower* or fewer or improv* or enhance* or extend* or develop* or prevalen* or daily or weekly or current or rate or rates or behavio?r* or knowledge or prevent* or attitude* or avoid* or perceive* or percept* or promot* or program* or change*)).ti,ab. (24784)
188 (positive behavio?r* adj5 (reduc* or decreas* or increas* or lower* or fewer or improv* or enhance* or extend* or develop* or prevalen* or daily or weekly or current or rate or rates or behavio?r* or knowledge or prevent* or attitude* or avoid* or perceive* or percept* or promot* or program* or change*)).ti,ab. (3800)
189 (improv* behavio?r* adj5 (reduc* or decreas* or increas* or lower* or fewer or improv* or enhance* or extend* or develop* or prevalen* or daily or weekly or current or rate or rates or behavio?r* or knowledge or prevent* or attitude* or avoid* or perceive* or percept* or promot* or program* or change*)).ti,ab. (1369)
190 (social behavio?r* adj5 (reduc* or decreas* or increas* or lower* or fewer or improv* or enhance* or extend* or develop* or prevalen* or daily or weekly or current or rate or rates or behavio?r* or knowledge or prevent* or attitude* or avoid* or perceive* or percept* or promot* or program* or change*)).ti,ab. (20509)
191 ((social-emotion* or socio-emotion*) adj5 (reduc* or decreas* or increas* or lower* or fewer or improv* or enhance* or extend* or develop* or prevalen* or daily or weekly or current or rate or rates or behavio?r* or knowledge or prevent* or attitude* or avoid* or perceive* or percept* or promot* or program* or change*)).ti,ab. (4824)
192 (social-charact* adj5 (reduc* or decreas* or increas* or lower* or fewer or improv* or enhance* or extend* or develop* or prevalen* or daily or weekly or current or rate or rates or behavio?r* or knowledge or prevent* or attitude* or avoid* or perceive* or percept* or promot* or program* or change*)).ti,ab. (304)
193 (social-inclusion adj5 (reduc* or decreas* or increas* or lower* or fewer or improv* or enhance* or extend* or develop* or prevalen* or daily or weekly or current or rate or rates or behavio?r* or knowledge or prevent* or attitude* or avoid* or perceive* or percept* or promot* or program* or change*)).ti,ab. (631)
194 (social develop* adj5 (reduc* or decreas* or increas* or lower* or fewer or improv* or enhance* or extend* or develop* or prevalen* or daily or weekly or current or rate or rates or behavio?r* or knowledge or prevent* or attitude* or avoid* or perceive* or percept* or promot* or program* or change*)).ti,ab. (6608)
195 (social competen* adj5 (reduc* or decreas* or increas* or lower* or fewer or improv* or enhance* or extend* or develop* or prevalen* or daily or weekly or current or rate or rates or behavio?r* or knowledge or prevent* or attitude* or avoid* or perceive* or percept* or promot* or program* or change*)).ti,ab. (3380)
196 (emotion* develop* adj5 (reduc* or decreas* or increas* or lower* or fewer or improv* or enhance* or extend* or develop* or prevalen* or daily or weekly or current or rate or rates or behavio?r* or knowledge or prevent* or attitude* or avoid* or perceive* or percept* or promot* or program* or change*)).ti,ab. (4904)
197 (conduct adj5 (reduc* or decreas* or increas* or lower* or fewer or improv* or enhance* or extend* or develop* or prevalen* or daily or weekly or current or rate or rates or behavio?r* or knowledge or prevent* or attitude* or avoid* or perceive* or percept* or promot* or program* or change*)).ti,ab. (7780)
198 (respect adj5 (reduc* or decreas* or increas* or lower* or fewer or improv* or enhance* or extend* or develop* or prevalen* or daily or weekly or current or rate or rates or behavio?r* or knowledge or prevent* or attitude* or avoid* or perceive* or percept* or promot* or program* or change*)).ti,ab. (12787)
199 ((safety or safe) adj5 (reduc* or decreas* or increas* or lower* or fewer or improv* or enhance* or extend* or develop* or prevalen* or daily or weekly or current or rate or rates or behavio?r* or knowledge or prevent* or attitude* or avoid* or perceive* or percept* or promot* or program* or change*)).ti,ab. (21199)
200 or/168-199 [VIOLENCE] (249466)
201 121 or 132 or 155 or 200 (459289)
202 exp program evaluation/ (20023)
203 exp clinical trials/ (11810)
204 prevent*.ti,ab. (213901)
205 program*.ti,ab. (382900)
206 intervent*.ti,ab. (365964)
207 restorative approach*.ti,ab. (117)
208 control school?.ti,ab. (615)
209 (trial or trials).ti,ab. (175203)
210 or/202-209 (919537)
211 56 and 106 and 201 and 210 (17477)

## SCOPUS

| Database name | SCOPUS |
| --- | --- |
| Database platform | [www.scopus.com](http://www.scopus.com) |
| Dates of database coverage | Complete database to search date |
| Date searched | 16/01/2020 |
| Searched by | JF |
| Number of results | 17,484 |
| EndNote import order | 14 |
| Number of results once duplicates removed | 4832 |
| Search strategy notes | All terms are searched in the title, abstract and author keywords. * is used for truncation of words. W/*n* searches for terms within *n* words or each other. |

(TITLE-ABS-KEY (child or boy or girl or schoolage or (school w/1 age) or schoolchild or youngster or minor or pupil or (student not "medical student") or prepubescent or pubescent or adolescent or juvenile or underage or preteen or "pre-teen" or teen or teenage or youth or "young person" or "young people" or kid or (transition w/4 adult) or "emerging adult" or "young adult" or parent or mother or father or guardian or teacher)) AND (TITLE-ABS-KEY ((school* W/5 (based OR level OR wide OR led OR ethos OR environment OR organization* OR organisation* OR climate OR toolkit* OR approach* OR "action group*" OR practice* OR whole OR health* OR leader* OR cultur* OR manag* OR governance OR system* OR communit*)) OR schoolwide OR "restor* justice" OR ((((peer or relationship* or child or children or "young people" or "young person" or teen or teens or teenage* or adolescen* or youth* or parent*) W/2 (led OR leader* OR participat* OR mediat* OR helper* OR resistan* OR wide OR action* or influen*)) OR ((community or communities or pupil or student or teach* or curricul* or administrat* or personnel or staff or volunteer* or advocacy or advocate or environment* or "socio-ecolog*" or learn* or policy or policies) W/2 (led OR leader* OR participat* OR mediat* OR helper* OR resistan* OR wide OR action* or influen* or relationship*)) OR "peer-to-peer") AND school*))) AND (TITLE-ABS-KEY (prevent* OR program* OR intervent* OR "restorative approach*" OR "control school*" OR trial OR trials)) AND (TITLE-ABS-KEY ((violent OR violence OR aggression OR aggressive OR hostile OR hostility OR assault* OR crime OR abuse* OR abusive OR misbehav* OR threat* OR conduct OR delinquen* OR disrupt* OR "problem* behavior*" OR "problem behaviour*" OR "volatile behavior*" OR "volatile behaviour*" OR antisocial OR "anti-social" OR perpetrat* OR bully* OR victim* OR cyberbully* OR conflict* OR "positive behavior*" OR "positive behaviour*" OR "improv* behavior*" OR "improv* behaviour*" OR "social behavior*" OR "social behaviour*" OR "social-emotion*" OR "socio-emotion*" OR "social-charact*" OR "social-inclusion" OR "social develop*" OR "social competen*" OR "emotion* develop*" OR conduct OR respect OR safety OR safe OR "education* attain*" OR "academic attain*" OR "student* attain*" OR "education* achieve*" OR "academic achieve*" OR "student achieve*" OR "education* perform*" OR "academic perform*" OR "education* perform*" OR "education* outcome*" OR "academic outcome*" OR "student* outcome*" OR "education* commitment" OR "academic commitment" OR "student* commitment" OR "education* engage*" OR "academic engage*" OR "student* engage*" OR dropout OR absentee* OR alcohol OR "binge drink*" OR "underage drink*" OR substance* OR drug* OR smoking OR smoked OR smoke OR smoker OR tobacco OR cigarette* OR marijuana OR cannabis OR solvent*) W/5 (reduc* OR decreas* OR increas* OR lower* OR fewer OR improv* OR enhance* OR extend* OR develop* OR prevalen* OR daily OR weekly OR current OR rate OR rates OR behavior* OR behaviour* OR knowledge OR prevent* OR attitude* OR avoid* OR perceive* OR percept* OR promot* OR program* OR change*)))

## OvidSP Social Policy and Practice

| Database name | Social Policy & Practice |
| --- | --- |
| Database platform | OvidSP |
| Dates of database coverage | 201910 |
| Date searched | 16/01/2020 |
| Searched by | JF |
| Number of results | 722 |
| EndNote import order | 7 |
| Number of results once duplicates removed | 441 |
| Search strategy notes | Search lines ending in .ti,ab. search in the title and abstract only. Search lines ending in .jx. search in the journal title only. or/*x-y* combines search sets in the range *x-y* with Boolean operator OR. * is used for truncation of words. # is used for a compulsory wildcard. ? is used for an optional wildcard. adj*n* searches for terms within *n* words or each other. Words in square brackets [ ] are comments and are not included in the search terms. |

1 child*.ti,ab. (78384)
2 boy?.ti,ab. (2309)
3 girl?.ti,ab. (2680)
4 (schoolage or (school adj1 age)).ti,ab. (437)
5 schoolchild*.ti,ab. (146)
6 youngster?.ti,ab. (203)
7 minor?.ti,ab. (973)
8 ((pupil* or student*) not ((pupil adj5 eye?) or (student* adj medic*))).ti,ab. (13663)
9 prepubescen*.ti,ab. (15)
10 pubescent*.ti,ab. (19)
11 adolescent*.ti,ab. (7461)
12 juvenil*.ti,ab. (1474)
13 underage*.ti,ab. (87)
14 (preteen* or pre-teen*).ti,ab. (28)
15 (teen or teens).ti,ab. (476)
16 teenage*.ti,ab. (2220)
17 (youth or youths).ti,ab. (10812)
18 young person*.ti,ab. (1492)
19 young people*.ti,ab. (24553)
20 (kid or kids).ti,ab. (474)
21 (transition adj4 adult*).ti,ab. (777)
22 emerging adult*.ti,ab. (103)
23 young adult.ti,ab. (337)
24 parent?.ti,ab. (22115)
25 mother?.ti,ab. (6618)
26 father?.ti,ab. (2564)
27 guardian?.ti,ab. (745)
28 teacher?.ti,ab. (4824)
29 child*.jx. (21796)
30 adolescen*.jx. (1277)
31 youth*.jx. (4509)
32 school*.jx. (330)
33 (("5" or "6" or "7" or "8" or "9" or "10" or "11" or "12" or "13" or "14" or "15" or "16" or "17" or "18") adj (year* old or year* of age)).ti,ab. (1712)
34 ((five or six or seven or eight or nine or ten or eleven or twelve or thirteen or fourteen or fifteen or sixteen or seventeen or eighteen) adj (year* old or year* of age)).ti,ab. (301)
35 (age* adj ("5" or "6" or "7" or "8" or "9" or "10" or "11" or "12" or "13" or "14" or "15" or "16" or "17" or "18") adj year*).ti,ab. (255)
36 (age* adj (five or six or seven or eight or nine or ten or eleven or twelve or thirteen or fourteen or fifteen or sixteen or seventeen or eighteen) adj year*).ti,ab. (20)
37 ((primary or secondary or year) adj1 ("1" or "2" or "3" or "4" or "5" or "6" or "7" or "8" or "9" or "10" or "11" or "12" or "13")).ti,ab. (3510)
38 (grade? adj1 (first or second or third or fourth or fifth or sixth or seventh or eighth or ninth or tenth or eleventh or twelfth)).ti,ab. (103)
39 or/1-38 (116037)
40 (school? adj5 (based or level or wide or led or ethos or environment or organi?ation* or climate or toolkit? or approach* or action group? or practice? or whole or health* or leader* or cultur* or manag* or governance or system? or communit*)).ti,ab. (6148)
41 schoolwide.ti,ab. (4)
42 restor* justice.ti,ab. (329)
43 school?.ti,ab,jx. (21869)
44 "peer-to-peer".ti,ab. (117)
45 (peer adj2 (led or leader* or participat* or mediat* or helper? or resistan* or wide or action? or influen*)).ti,ab. (249)
46 (relationship* adj2 (led or leader* or participat* or mediat* or helper? or resistan* or wide or action? or influen*)).ti,ab. (701)
47 ((community or communities) adj2 (led or leader* or participat* or mediat* or helper? or resistan* or wide or action? or influen* or relationship*)).ti,ab. (2283)
48 (pupil? adj2 (led or leader* or participat* or mediat* or helper? or resistan* or wide or action? or influen* or relationship*)).ti,ab. (135)
49 ((child or children) adj2 (led or leader* or participat* or mediat* or helper? or resistan* or wide or action? or influen*)).ti,ab. (1282)
50 ((young people or young person) adj2 (led or leader* or participat* or mediat* or helper? or resistan* or wide or action? or influen*)).ti,ab. (423)
51 (teen* adj2 (led or leader* or participat* or mediat* or helper? or resistan* or wide or action? or influen*)).ti,ab. (28)
52 (adolescent* adj2 (led or leader* or participat* or mediat* or helper? or resistan* or wide or action? or influen*)).ti,ab. (142)
53 (youth? adj2 (led or leader* or participat* or mediat* or helper? or resistan* or wide or action? or influen*)).ti,ab. (379)
54 (student? adj2 (led or leader* or participat* or mediat* or helper? or resistan* or wide or action? or influen*)).ti,ab. (300)
55 (teach* adj2 (led or leader* or participat* or mediat* or helper? or resistan* or wide or action? or influen* or relationship*)).ti,ab. (280)
56 (curricul* adj2 (led or leader* or participat* or mediat* or helper? or resistan* or wide or action? or influen* or relationship*)).ti,ab. (51)
57 (administrat* adj2 (led or leader* or participat* or mediat* or helper? or resistan* or wide or action? or influen* or relationship*)).ti,ab. (48)
58 ((personnel or staff) adj2 (led or leader* or participat* or mediat* or helper? or resistan* or wide or action? or influen* or relationship*)).ti,ab. (515)
59 (volunteer* adj2 (led or leader* or participat* or mediat* or helper? or resistan* or wide or action? or influen* or relationship*)).ti,ab. (243)
60 ((advocacy or advocate) adj2 (led or leader* or participat* or mediat* or helper? or resistan* or wide or action? or influen* or relationship*)).ti,ab. (147)
61 (parent* adj2 (led or leader* or participat* or mediat* or helper? or resistan* or wide or action? or influen*)).ti,ab. (728)
62 (environment* adj2 (led or leader* or participat* or mediat* or helper? or resistan* or wide or action? or influen* or relationship*)).ti,ab. (470)
63 (socio-ecolog* adj2 (led or leader* or participat* or mediat* or helper? or resistan* or wide or action? or influen* or relationship*)).ti,ab. (0)
64 (learn* adj2 (led or leader* or participat* or mediat* or helper? or resistan* or wide or action? or influen* or relationship*)).ti,ab. (527)
65 ((policy or policies) adj2 (led or leader* or participat* or mediat* or helper? or resistan* or wide or action? or influen* or relationship*)).ti,ab. (1201)
66 or/44-65 (9535)
67 43 and 66 (1300)
68 or/40-42,67 (7245)
69 ((smoking or smoked or smoke or smoker) adj5 (reduc* or decreas* or increas* or lower* or fewer or improv* or enhance* or extend* or develop* or prevalen* or daily or weekly or current or rate or rates or behavio?r* or knowledge or prevent* or attitude* or avoid* or perceive* or percept* or promot* or program* or change*)).ti,ab. (524)
70 (tobacco adj5 (reduc* or decreas* or increas* or lower* or fewer or improv* or enhance* or extend* or develop* or prevalen* or daily or weekly or current or rate or rates or behavio?r* or knowledge or prevent* or attitude* or avoid* or perceive* or percept* or promot* or program* or change*)).ti,ab. (110)
71 (cigarette? adj5 (reduc* or decreas* or increas* or lower* or fewer or improv* or enhance* or extend* or develop* or prevalen* or daily or weekly or current or rate or rates or behavio?r* or knowledge or prevent* or attitude* or avoid* or perceive* or percept* or promot* or program* or change*)).ti,ab. (61)
72 (marijuana adj5 (reduc* or decreas* or increas* or lower* or fewer or improv* or enhance* or extend* or develop* or prevalen* or daily or weekly or current or rate or rates or behavio?r* or knowledge or prevent* or attitude* or avoid* or perceive* or percept* or promot* or program* or change*)).ti,ab. (29)
73 (cannabis adj5 (reduc* or decreas* or increas* or lower* or fewer or improv* or enhance* or extend* or develop* or prevalen* or daily or weekly or current or rate or rates or behavio?r* or knowledge or prevent* or attitude* or avoid* or perceive* or percept* or promot* or program* or change*)).ti,ab. (63)
74 (solvent? adj5 (reduc* or decreas* or increas* or lower* or fewer or improv* or enhance* or extend* or develop* or prevalen* or daily or weekly or current or rate or rates or behavio?r* or knowledge or prevent* or attitude* or avoid* or perceive* or percept* or promot* or program* or change*)).ti,ab. (12)
75 (alcohol adj5 (reduc* or decreas* or increas* or lower* or fewer or improv* or enhance* or extend* or develop* or prevalen* or daily or weekly or current or rate or rates or behavio?r* or knowledge or prevent* or attitude* or avoid* or perceive* or percept* or promot* or program* or change*)).ti,ab. (1339)
76 (binge drink* adj5 (reduc* or decreas* or increas* or lower* or fewer or improv* or enhance* or extend* or develop* or prevalen* or daily or weekly or current or rate or rates or behavio?r* or knowledge or prevent* or attitude* or avoid* or perceive* or percept* or promot* or program* or change*)).ti,ab. (48)
77 (underage drink* adj5 (reduc* or decreas* or increas* or lower* or fewer or improv* or enhance* or extend* or develop* or prevalen* or daily or weekly or current or rate or rates or behavio?r* or knowledge or prevent* or attitude* or avoid* or perceive* or percept* or promot* or program* or change*)).ti,ab. (13)
78 (substance? adj5 (reduc* or decreas* or increas* or lower* or fewer or improv* or enhance* or extend* or develop* or prevalen* or daily or weekly or current or rate or rates or behavio?r* or knowledge or prevent* or attitude* or avoid* or perceive* or percept* or promot* or program* or change*)).ti,ab. (1338)
79 (drug? adj5 (reduc* or decreas* or increas* or lower* or fewer or improv* or enhance* or extend* or develop* or prevalen* or daily or weekly or current or rate or rates or behavio?r* or knowledge or prevent* or attitude* or avoid* or perceive* or percept* or promot* or program* or change*)).ti,ab. (2084)
80 (education* attain* adj5 (reduc* or decreas* or increas* or lower* or fewer or improv* or enhance* or extend* or develop* or prevalen* or daily or weekly or current or rate or rates or behavio?r* or knowledge or prevent* or attitude* or avoid* or perceive* or percept* or promot* or program* or change*)).ti,ab. (282)
81 (academic attain* adj5 (reduc* or decreas* or increas* or lower* or fewer or improv* or enhance* or extend* or develop* or prevalen* or daily or weekly or current or rate or rates or behavio?r* or knowledge or prevent* or attitude* or avoid* or perceive* or percept* or promot* or program* or change*)).ti,ab. (18)
82 (student* attain* adj5 (reduc* or decreas* or increas* or lower* or fewer or improv* or enhance* or extend* or develop* or prevalen* or daily or weekly or current or rate or rates or behavio?r* or knowledge or prevent* or attitude* or avoid* or perceive* or percept* or promot* or program* or change*)).ti,ab. (5)
83 (education* achieve* adj5 (reduc* or decreas* or increas* or lower* or fewer or improv* or enhance* or extend* or develop* or prevalen* or daily or weekly or current or rate or rates or behavio?r* or knowledge or prevent* or attitude* or avoid* or perceive* or percept* or promot* or program* or change*)).ti,ab. (116)
84 (academic achieve* adj5 (reduc* or decreas* or increas* or lower* or fewer or improv* or enhance* or extend* or develop* or prevalen* or daily or weekly or current or rate or rates or behavio?r* or knowledge or prevent* or attitude* or avoid* or perceive* or percept* or promot* or program* or change*)).ti,ab. (68)
85 (student* achieve* adj5 (reduc* or decreas* or increas* or lower* or fewer or improv* or enhance* or extend* or develop* or prevalen* or daily or weekly or current or rate or rates or behavio?r* or knowledge or prevent* or attitude* or avoid* or perceive* or percept* or promot* or program* or change*)).ti,ab. (16)
86 (education* perform* adj5 (reduc* or decreas* or increas* or lower* or fewer or improv* or enhance* or extend* or develop* or prevalen* or daily or weekly or current or rate or rates or behavio?r* or knowledge or prevent* or attitude* or avoid* or perceive* or percept* or promot* or program* or change*)).ti,ab. (36)
87 (academic perform* adj5 (reduc* or decreas* or increas* or lower* or fewer or improv* or enhance* or extend* or develop* or prevalen* or daily or weekly or current or rate or rates or behavio?r* or knowledge or prevent* or attitude* or avoid* or perceive* or percept* or promot* or program* or change*)).ti,ab. (57)
88 (student* perform* adj5 (reduc* or decreas* or increas* or lower* or fewer or improv* or enhance* or extend* or develop* or prevalen* or daily or weekly or current or rate or rates or behavio?r* or knowledge or prevent* or attitude* or avoid* or perceive* or percept* or promot* or program* or change*)).ti,ab. (8)
89 (education* outcome? adj5 (reduc* or decreas* or increas* or lower* or fewer or improv* or enhance* or extend* or develop* or prevalen* or daily or weekly or current or rate or rates or behavio?r* or knowledge or prevent* or attitude* or avoid* or perceive* or percept* or promot* or program* or change*)).ti,ab. (135)
90 (academic outcome? adj5 (reduc* or decreas* or increas* or lower* or fewer or improv* or enhance* or extend* or develop* or prevalen* or daily or weekly or current or rate or rates or behavio?r* or knowledge or prevent* or attitude* or avoid* or perceive* or percept* or promot* or program* or change*)).ti,ab. (17)
91 (student* outcome? adj5 (reduc* or decreas* or increas* or lower* or fewer or improv* or enhance* or extend* or develop* or prevalen* or daily or weekly or current or rate or rates or behavio?r* or knowledge or prevent* or attitude* or avoid* or perceive* or percept* or promot* or program* or change*)).ti,ab. (13)
92 (education* commitment adj5 (reduc* or decreas* or increas* or lower* or fewer or improv* or enhance* or extend* or develop* or prevalen* or daily or weekly or current or rate or rates or behavio?r* or knowledge or prevent* or attitude* or avoid* or perceive* or percept* or promot* or program* or change*)).ti,ab. (0)
93 (academic commitment adj5 (reduc* or decreas* or increas* or lower* or fewer or improv* or enhance* or extend* or develop* or prevalen* or daily or weekly or current or rate or rates or behavio?r* or knowledge or prevent* or attitude* or avoid* or perceive* or percept* or promot* or program* or change*)).ti,ab. (0)
94 (student* commitment adj5 (reduc* or decreas* or increas* or lower* or fewer or improv* or enhance* or extend* or develop* or prevalen* or daily or weekly or current or rate or rates or behavio?r* or knowledge or prevent* or attitude* or avoid* or perceive* or percept* or promot* or program* or change*)).ti,ab. (1)
95 (education* engage* adj5 (reduc* or decreas* or increas* or lower* or fewer or improv* or enhance* or extend* or develop* or prevalen* or daily or weekly or current or rate or rates or behavio?r* or knowledge or prevent* or attitude* or avoid* or perceive* or percept* or promot* or program* or change*)).ti,ab. (4)
96 (academic engage* adj5 (reduc* or decreas* or increas* or lower* or fewer or improv* or enhance* or extend* or develop* or prevalen* or daily or weekly or current or rate or rates or behavio?r* or knowledge or prevent* or attitude* or avoid* or perceive* or percept* or promot* or program* or change*)).ti,ab. (1)
97 (student* engage* adj5 (reduc* or decreas* or increas* or lower* or fewer or improv* or enhance* or extend* or develop* or prevalen* or daily or weekly or current or rate or rates or behavio?r* or knowledge or prevent* or attitude* or avoid* or perceive* or percept* or promot* or program* or change*)).ti,ab. (11)
98 (dropout adj5 (reduc* or decreas* or increas* or lower* or fewer or improv* or enhance* or extend* or develop* or prevalen* or daily or weekly or current or rate or rates or behavio?r* or knowledge or prevent* or attitude* or avoid* or perceive* or percept* or promot* or program* or change*)).ti,ab. (86)
99 (absentee* adj5 (reduc* or decreas* or increas* or lower* or fewer or improv* or enhance* or extend* or develop* or prevalen* or daily or weekly or current or rate or rates or behavio?r* or knowledge or prevent* or attitude* or avoid* or perceive* or percept* or promot* or program* or change*)).ti,ab. (60)
100 ((violent or violence) adj5 (reduc* or decreas* or increas* or lower* or fewer or improv* or enhance* or extend* or develop* or prevalen* or daily or weekly or current or rate or rates or behavio?r* or knowledge or prevent* or attitude* or avoid* or perceive* or percept* or promot* or program* or change*)).ti,ab. (3051)
101 ((aggression or aggressive) adj5 (reduc* or decreas* or increas* or lower* or fewer or improv* or enhance* or extend* or develop* or prevalen* or daily or weekly or current or rate or rates or behavio?r* or knowledge or prevent* or attitude* or avoid* or perceive* or percept* or promot* or program* or change*)).ti,ab. (1081)
102 ((hostile or hostility) adj5 (reduc* or decreas* or increas* or lower* or fewer or improv* or enhance* or extend* or develop* or prevalen* or daily or weekly or current or rate or rates or behavio?r* or knowledge or prevent* or attitude* or avoid* or perceive* or percept* or promot* or program* or change*)).ti,ab. (146)
103 (assault* adj5 (reduc* or decreas* or increas* or lower* or fewer or improv* or enhance* or extend* or develop* or prevalen* or daily or weekly or current or rate or rates or behavio?r* or knowledge or prevent* or attitude* or avoid* or perceive* or percept* or promot* or program* or change*)).ti,ab. (223)
104 (crime adj5 (reduc* or decreas* or increas* or lower* or fewer or improv* or enhance* or extend* or develop* or prevalen* or daily or weekly or current or rate or rates or behavio?r* or knowledge or prevent* or attitude* or avoid* or perceive* or percept* or promot* or program* or change*)).ti,ab. (2305)
105 ((abuse? or abusive) adj5 (reduc* or decreas* or increas* or lower* or fewer or improv* or enhance* or extend* or develop* or prevalen* or daily or weekly or current or rate or rates or behavio?r* or knowledge or prevent* or attitude* or avoid* or perceive* or percept* or promot* or program* or change*)).ti,ab. (5369)
106 (misbehav* adj5 (reduc* or decreas* or increas* or lower* or fewer or improv* or enhance* or extend* or develop* or prevalen* or daily or weekly or current or rate or rates or behavio?r* or knowledge or prevent* or attitude* or avoid* or perceive* or percept* or promot* or program* or change*)).ti,ab. (23)
107 (threat? adj5 (reduc* or decreas* or increas* or lower* or fewer or improv* or enhance* or extend* or develop* or prevalen* or daily or weekly or current or rate or rates or behavio?r* or knowledge or prevent* or attitude* or avoid* or perceive* or percept* or promot* or program* or change*)).ti,ab. (319)
108 (conduct adj5 (reduc* or decreas* or increas* or lower* or fewer or improv* or enhance* or extend* or develop* or prevalen* or daily or weekly or current or rate or rates or behavio?r* or knowledge or prevent* or attitude* or avoid* or perceive* or percept* or promot* or program* or change*)).ti,ab. (389)
109 (delinquen* adj5 (reduc* or decreas* or increas* or lower* or fewer or improv* or enhance* or extend* or develop* or prevalen* or daily or weekly or current or rate or rates or behavio?r* or knowledge or prevent* or attitude* or avoid* or perceive* or percept* or promot* or program* or change*)).ti,ab. (360)
110 (disrupt* behavio?r* adj5 (reduc* or decreas* or increas* or lower* or fewer or improv* or enhance* or extend* or develop* or prevalen* or daily or weekly or current or rate or rates or behavio?r* or knowledge or prevent* or attitude* or avoid* or perceive* or percept* or promot* or program* or change*)).ti,ab. (297)
111 (problem* behavio?r* adj5 (reduc* or decreas* or increas* or lower* or fewer or improv* or enhance* or extend* or develop* or prevalen* or daily or weekly or current or rate or rates or behavio?r* or knowledge or prevent* or attitude* or avoid* or perceive* or percept* or promot* or program* or change*)).ti,ab. (642)
112 (volatile behavio?r* adj5 (reduc* or decreas* or increas* or lower* or fewer or improv* or enhance* or extend* or develop* or prevalen* or daily or weekly or current or rate or rates or behavio?r* or knowledge or prevent* or attitude* or avoid* or perceive* or percept* or promot* or program* or change*)).ti,ab. (1)
113 ((antisocial or anti-social) adj5 (reduc* or decreas* or increas* or lower* or fewer or improv* or enhance* or extend* or develop* or prevalen* or daily or weekly or current or rate or rates or behavio?r* or knowledge or prevent* or attitude* or avoid* or perceive* or percept* or promot* or program* or change*)).ti,ab. (2270)
114 (perpetrat* adj5 (reduc* or decreas* or increas* or lower* or fewer or improv* or enhance* or extend* or develop* or prevalen* or daily or weekly or current or rate or rates or behavio?r* or knowledge or prevent* or attitude* or avoid* or perceive* or percept* or promot* or program* or change*)).ti,ab. (472)
115 (bully* adj5 (reduc* or decreas* or increas* or lower* or fewer or improv* or enhance* or extend* or develop* or prevalen* or daily or weekly or current or rate or rates or behavio?r* or knowledge or prevent* or attitude* or avoid* or perceive* or percept* or promot* or program* or change*)).ti,ab. (584)
116 (victim* adj5 (reduc* or decreas* or increas* or lower* or fewer or improv* or enhance* or extend* or develop* or prevalen* or daily or weekly or current or rate or rates or behavio?r* or knowledge or prevent* or attitude* or avoid* or perceive* or percept* or promot* or program* or change*)).ti,ab. (1406)
117 (cyberbully* adj5 (reduc* or decreas* or increas* or lower* or fewer or improv* or enhance* or extend* or develop* or prevalen* or daily or weekly or current or rate or rates or behavio?r* or knowledge or prevent* or attitude* or avoid* or perceive* or percept* or promot* or program* or change*)).ti,ab. (75)
118 (conflict* adj5 (reduc* or decreas* or increas* or lower* or fewer or improv* or enhance* or extend* or develop* or prevalen* or daily or weekly or current or rate or rates or behavio?r* or knowledge or prevent* or attitude* or avoid* or perceive* or percept* or promot* or program* or change*)).ti,ab. (816)
119 (positive behavio?r* adj5 (reduc* or decreas* or increas* or lower* or fewer or improv* or enhance* or extend* or develop* or prevalen* or daily or weekly or current or rate or rates or behavio?r* or knowledge or prevent* or attitude* or avoid* or perceive* or percept* or promot* or program* or change*)).ti,ab. (239)
120 (improv* behavio?r* adj5 (reduc* or decreas* or increas* or lower* or fewer or improv* or enhance* or extend* or develop* or prevalen* or daily or weekly or current or rate or rates or behavio?r* or knowledge or prevent* or attitude* or avoid* or perceive* or percept* or promot* or program* or change*)).ti,ab. (88)
121 (social behavio?r* adj5 (reduc* or decreas* or increas* or lower* or fewer or improv* or enhance* or extend* or develop* or prevalen* or daily or weekly or current or rate or rates or behavio?r* or knowledge or prevent* or attitude* or avoid* or perceive* or percept* or promot* or program* or change*)).ti,ab. (1703)
122 ((social-emotion* or socio-emotion*) adj5 (reduc* or decreas* or increas* or lower* or fewer or improv* or enhance* or extend* or develop* or prevalen* or daily or weekly or current or rate or rates or behavio?r* or knowledge or prevent* or attitude* or avoid* or perceive* or percept* or promot* or program* or change*)).ti,ab. (219)
123 (social-charact* adj5 (reduc* or decreas* or increas* or lower* or fewer or improv* or enhance* or extend* or develop* or prevalen* or daily or weekly or current or rate or rates or behavio?r* or knowledge or prevent* or attitude* or avoid* or perceive* or percept* or promot* or program* or change*)).ti,ab. (23)
124 (social-inclusion adj5 (reduc* or decreas* or increas* or lower* or fewer or improv* or enhance* or extend* or develop* or prevalen* or daily or weekly or current or rate or rates or behavio?r* or knowledge or prevent* or attitude* or avoid* or perceive* or percept* or promot* or program* or change*)).ti,ab. (935)
125 (social develop* adj5 (reduc* or decreas* or increas* or lower* or fewer or improv* or enhance* or extend* or develop* or prevalen* or daily or weekly or current or rate or rates or behavio?r* or knowledge or prevent* or attitude* or avoid* or perceive* or percept* or promot* or program* or change*)).ti,ab. (578)
126 (social competen* adj5 (reduc* or decreas* or increas* or lower* or fewer or improv* or enhance* or extend* or develop* or prevalen* or daily or weekly or current or rate or rates or behavio?r* or knowledge or prevent* or attitude* or avoid* or perceive* or percept* or promot* or program* or change*)).ti,ab. (80)
127 (emotion* develop* adj5 (reduc* or decreas* or increas* or lower* or fewer or improv* or enhance* or extend* or develop* or prevalen* or daily or weekly or current or rate or rates or behavio?r* or knowledge or prevent* or attitude* or avoid* or perceive* or percept* or promot* or program* or change*)).ti,ab. (208)
128 (conduct adj5 (reduc* or decreas* or increas* or lower* or fewer or improv* or enhance* or extend* or develop* or prevalen* or daily or weekly or current or rate or rates or behavio?r* or knowledge or prevent* or attitude* or avoid* or perceive* or percept* or promot* or program* or change*)).ti,ab. (389)
129 (respect adj5 (reduc* or decreas* or increas* or lower* or fewer or improv* or enhance* or extend* or develop* or prevalen* or daily or weekly or current or rate or rates or behavio?r* or knowledge or prevent* or attitude* or avoid* or perceive* or percept* or promot* or program* or change*)).ti,ab. (617)
130 ((safety or safe) adj5 (reduc* or decreas* or increas* or lower* or fewer or improv* or enhance* or extend* or develop* or prevalen* or daily or weekly or current or rate or rates or behavio?r* or knowledge or prevent* or attitude* or avoid* or perceive* or percept* or promot* or program* or change*)).ti,ab. (2338)
131 or/69-130 (25328)
132 prevent*.ti,ab. (17584)
133 program*.ti,ab. (33590)
134 intervent*.ti,ab. (25507)
135 restorative approach*.ti,ab. (48)
136 control school?.ti,ab. (13)
137 (trial or trials).ti,ab. (3955)
138 or/132-137 (66086)
139 39 and 68 and 131 and 138 (722)

## Clarivate Analytics Web of Science Social Sciences Citation Index

| Database name | Social Sciences Citation Index |
| --- | --- |
| Database platform | Clarivate Analytics Web of Science |
| Dates of database coverage | 1970 – present, last updated 09 January 2020 |
| Date searched | 10 January 2020 |
| Searched by | JF |
| Number of results | 11,189 |
| EndNote import order | 13 |
| Number of results once duplicates removed | 3232 |
| Search strategy notes | All terms are searched in the title, abstract and author keywords. * is used for truncation of words. W/*n* searches for terms within *n* words or each other. |

| # 1 | TS=(child* or boy$ or girl$ or schoolage or (school near/1 age) or schoolchild* or youngster$ or minor$ or pupil* or (student* not "medical student*") or prepubescen* or pubescent* or adolescent* or juvenil* or underage* or preteen* or "pre-teen*" or teen or teens or teenage* or youth or youths or "young person*" or "young people*" or kid or kids or (transition near/4 adult*) or "emerging adult*" or "young adult*" or parent$ or mother$ or father$ or guardian$ or teacher$) | 1,166,322 |  |
| --- | --- | --- | --- |
| # 2 | SO=(child* or adolescen* or youth* or school*) | 50,677 |  |
| # 3 | #2 OR #1 | 1,176,163 |  |
| # 4 | TS=(school$ near/5 (based or level or wide or led or ethos or environment or organi?ation* or climate or toolkit$ or approach* or "action group$" or practice$ or whole or health* or leader* or cultur* or manag* or governance or system$ or communit*)) | 74,819 |  |
| # 5 | TS=(schoolwide or "restor* justice") | 1,263 |  |
| # 6 | TS=(school$) | 299,298 |  |
| # 7 | TS=("peer-to-peer") | 1,659 |  |
| # 8 | TS=((peer or relationship* or child or children or "young people" or "young person" or teen or teens or teenage* or adolescen* or youth$ or parent*) near/2 (led or leader* or participat* or mediat* or helper$ or resistan* or wide or action$ or influen*)) | 72,087 |  |
| # 9 | TS=((community or communities or pupil$ or student$ or teach* or curricul* or administrat* or personnel or staff or volunteer* or advocacy or advocate or environment* or "socio-ecolog*" or learn* or policy or policies) near/2 (led or leader* or participat* or mediat* or helper$ or resistan* or wide or action$ or influen* or relationship*)) | 124,783 |  |
| # 10 | #9 OR #8 OR #7 | 190,877 |  |
| # 11 | #10 AND #6 | 35,866 |  |
| # 12 | #11 OR #5 OR #4 | 99,148 |  |
| # 13 | TS=(((smoking or smoked or smoke or smoker or tobacco or cigarette$ or marijuana or cannabis or solvent$) near/5 (reduc* or decreas* or increas* or lower* or fewer or improv* or enhance* or extend* or develop* or prevalen* or daily or weekly or current or rate or rates or behavio$r* or knowledge or prevent* or attitude* or avoid* or perceive* or percept* or promot* or program* or change*))) | 48,614 |  |
| # 14 | TS=(((alcohol or "binge drink*" or "underage drink*" or substance$ or drug$) near/5 (reduc* or decreas* or increas* or lower* or fewer or improv* or enhance* or extend* or develop* or prevalen* or daily or weekly or current or rate or rates or behavio$r* or knowledge or prevent* or attitude* or avoid* or perceive* or percept* or promot* or program* or change*))) | 91,390 |  |
| # 15 | TS=((("education* attain*" or "academic attain*" or "student* attain*" or "education* achieve*" or "academic achieve*" or "student achieve*" or "education* perform*" or "academic perform*" or "education* perform*" or "education* outcome$" or "academic outcome$" or "student* outcome$" or "education* commitment" or "academic commitment" or "student* commitment" or "education* engage*" or "academic engage*" or "student* engage*" or dropout or absentee*) near/5 (reduc* or decreas* or increas* or lower* or fewer or improv* or enhance* or extend* or develop* or prevalen* or daily or weekly or current or rate or rates or behavio$r* or knowledge or prevent* or attitude* or avoid* or perceive* or percept* or promot* or program* or change*))) | 17,322 |  |
| # 16 | TS=(((violent or violence or aggression or aggressive or hostile or hostility or assault* or crime or abuse$ or abusive or misbehav* or threat$ or conduct or delinquen* or disrupt* or "problem* behavio$r*" or "volatile behavio$r*" or antisocial or "anti-social" or perpetrat* or bully* or victim* or cyberbully* or conflict* or "positive behavio$r*" or "improv* behavio$r*" or "social behavio$r*" or "social-emotion*" or "socio-emotion*" or "social-charact*" or "social-inclusion" or "social develop*" or "social competen*" or "emotion* develop*" or conduct or respect or safety or safe) near/5 (reduc* or decreas* or increas* or lower* or fewer or improv* or enhance* or extend* or develop* or prevalen* or daily or weekly or current or rate or rates or behavio$r* or knowledge or prevent* or attitude* or avoid* or perceive* or percept* or promot* or program* or change*))) | 259,976 |  |
| # 17 | #16 OR #15 OR #14 OR #13 | 380,448 |  |
| # 18 | TS=(prevent* or program* or intervent* or "restorative approach*" or "control school$" or trial or trials) | 975,370 |  |
| # 19 | #18 AND #17 AND #12 AND #3 | 11,189 |  |

For all searches: Indexes=SSCI Timespan=All years

## EBSCO Teacher Reference Center

| Database name | Teacher Reference Center |
| --- | --- |
| Database platform | EBSCO |
| Dates of database coverage | Complete database to date searched. |
| Date searched | 24/01/2020 |
| Searched by | JF |
| Number of results | 1987 |
| EndNote import order | 18 |
| Number of results once duplicates removed | 410 |
| Search strategy notes | * is used for truncation. # is used for an optional wildcard. Search strings starting with TI search the title only. Search strings starting with AB search the abstract only. Search strings starting with SO search the journal title only. N/*n* search for terms within *n* words of each other, in any order. |

| Search number | Query | Results |
| --- | --- | --- |
| S1 | (TI child*) or (AB child*) | 86,719 |
| S2 | (TI boy#) or (AB boy#) | 8,481 |
| S3 | (TI girl#) or (AB girl#) | 10,254 |
| S4 | (TI (schoolage or (school N1 age))) or (AB (schoolage or (school N1 age))) | 898 |
| S5 | (TI schoolchild*) or (AB schoolchild*) | 918 |
| S6 | (TI youngster#) OR (AB youngster#) | 626 |
| S7 | (TI minor#) OR (AB minor#) | 1,070 |
| S8 | (TI pupil*) OR (AB pupil*) | 13,961 |
| S9 | ((TI student*) OR (AB student*)) NOT ((TI "medical student*") or (AB "medical student*")) | 227,724 |
| S10 | (TI prepubescen*) OR (AB prepubescen*) | 9 |
| S11 | (TI pubescent*) or (AB pubescent*) | 15 |
| S12 | (TI adolescent*) OR (AB adolescent*) | 6,574 |
| S13 | (TI juvenil*) OR (AB juvenil*) | 838 |
| S14 | (TI underage*) or (AB underage*) | 104 |
| S15 | (TI (preteen* or pre-teen*)) OR (AB (preteen* or pre-teen*)) | 75 |
| S16 | (TI (teen or teens)) OR (AB (teen or teens)) | 3,087 |
| S17 | (TI teenage*) OR (AB teenage*) | 4,767 |
| S18 | (TI (youth or youths)) OR (AB (youth or youths)) | 9,980 |
| S19 | (TI "young person*") OR (AB "young person*") | 183 |
| S20 | (TI "young people*") OR (AB "young people*") | 5,276 |
| S21 | (TI (kid or kids)) OR (AB (kid or kids)) | 7,400 |
| S22 | (TI (transition n4 adult*)) OR (AB (transition N4 adult*)) | 185 |
| S23 | (TI "emerging adult*") OR (AB "emerging adult*") | 59 |
| S24 | (TI "young adult*") OR (AB "young adult*") | 4,696 |
| S25 | (TI parent#) OR (AB parent#) | 23,116 |
| S26 | (TI mother#) OR (AB mother#) | 4,193 |
| S27 | (TI father#) or (AB father#) | 2,344 |
| S28 | (TI guardian#) OR (AB guardian#) | 451 |
| S29 | (TI teacher#) OR (AB teacher#) | 136,019 |
| S30 | (SO child*) | 20,386 |
| S31 | (SO adolescen*) | 3,481 |
| S32 | (SO youth*) | 0 |
| S33 | (SO school*) | 204,289 |
| S34 | (TI ("5" or "6" or "7" or "8" or "9" or "10" or "11" or "12" or "13" or "14" or "15" or "16" or "17" or "18") N1 ("year* old" or "year* of age")) OR (AB ("5" or "6" or "7" or "8" or "9" or "10" or "11" or "12" or "13" or "14" or "15" or "16" or "17" or "18") N1 ("year* old" or "year* of age")) | 661 |
| S35 | (TI (five or six or seven or eight or nine or ten or eleven or twelve or thirteen or fourteen or fifteen or sixteen or seventeen or eighteen) N1 ("year* old" or "year* of age")) OR (AB (five or six or seven or eight or nine or ten or eleven or twelve or thirteen or fourteen or fifteen or sixteen or seventeen or eighteen) N1 ("year* old" or "year* of age")) | 1,496 |
| S36 | (TI (age* N1 ("5" or "6" or "7" or "8" or "9" or "10" or "11" or "12" or "13" or "14" or "15" or "16" or "17" or "18") N1 year*)) OR (AB (age* N1 ("5" or "6" or "7" or "8" or "9" or "10" or "11" or "12" or "13" or "14" or "15" or "16" or "17" or "18") N1 year*)) | 306 |
| S37 | (TI (age* N1 (five or six or seven or eight or nine or ten or eleven or twelve or thirteen or fourteen or fifteen or sixteen or seventeen or eighteen) N1 year*)) OR (AB (age* N1 (five or six or seven or eight or nine or ten or eleven or twelve or thirteen or fourteen or fifteen or sixteen or seventeen or eighteen) N1 year*)) | 357 |
| S38 | (TI (primary or secondary or year) N1 ("1" or "2" or "3" or "4" or "5" or "6" or "7" or "8" or "9" or "10" or "11" or "12" or "13")) OR (AB (primary or secondary or year) N1 ("1" or "2" or "3" or "4" or "5" or "6" or "7" or "8" or "9" or "10" or "11" or "12" or "13")) | 5,319 |
| S39 | (TI grade# N1 (first or second or third or fourth or fifth or sixth or seventh or eighth or ninth or tenth or eleventh or twelfth)) OR (AB grade# N1 (first or second or third or fourth or fifth or sixth or seventh or eighth or ninth or tenth or eleventh or twelfth)) | 8,870 |
| S40 | S1 OR S2 OR S3 OR S4 OR S5 OR S6 OR S7 OR S8 OR S9 OR S10 OR S11 OR S12 OR S13 OR S14 OR S15 OR S16 OR S17 OR S18 OR S19 OR S20 OR S21 OR S22 OR S23 OR S24 OR S25 OR S26 OR S27 OR S28 OR S29 OR S30 OR S31 OR S32 OR S33 OR S34 OR S35 OR S36 OR S37 OR S38 OR S39 | 579,882 |
| S41 | (TI (school# N5 (based or level or wide or led or ethos or environment or organi#ation* or climate or toolkit# or approach* or "action group#" or practice# or whole or health* or leader* or cultur* or manag* or governance or system# or communit*))) OR (AB (school# N5 (based or level or wide or led or ethos or environment or organi#ation* or climate or toolkit# or approach* or "action group#" or practice# or whole or health* or leader* or cultur* or manag* or governance or system# or communit*))) | 47,238 |
| S42 | (TI schoolwide) or (AB schoolwide) | 421 |
| S43 | (TI "restor* justice") OR (AB "restor* justice") | 78 |
| S44 | (TI school#) or (AB school#) | 221,452 |
| S45 | (TI "peer-to-peer") OR (AB "peer-to-peer") | 210 |
| S46 | (TI (peer N2 (led or leader* or participat* or mediat* or helper# or resistan* or wide or action# or influen*))) OR (AB (peer N2 (led or leader* or participat* or mediat* or helper# or resistan* or wide or action# or influen*))) | 681 |
| S47 | (TI (relationship* N2 (led or leader* or participat* or mediat* or helper# or resistan* or wide or action# or influen*))) OR (AB (relationship* N2 (led or leader* or participat* or mediat* or helper# or resistan* or wide or action# or influen*))) | 924 |
| S48 | (TI ((community or communities) N2 (led or leader* or participat* or mediat* or helper# or resistan* or wide or action# or influen* or relationship*))) OR (AB ((community or communities) N2 (led or leader* or participat* or mediat* or helper# or resistan* or wide or action# or influen* or relationship*))) | 1,816 |
| S49 | ((TI (pupil# N2 (led or leader* or participat* or mediat* or helper# or resistan* or wide or action# or influen* or relationship*)) OR (AB (pupil# N2 (led or leader* or participat* or mediat* or helper# or resistan* or wide or action# or influen* or relationship*))) | 348 |
| S50 | (TI ((child or children) N2 (led or leader* or participat* or mediat* or helper# or resistan* or wide or action# or influen*))) OR (AB (child or children*) N2 (led or leader* or participat* or mediat* or helper# or resistan* or wide or action# or influen*))) | 1,582 |
| S51 | (TI (("young people" or "young person") N2 (led or leader* or participat* or mediat* or helper# or resistan* or wide or action# or influen*))) OR (AB (("young people" or "young person") N2 (led or leader* or participat* or mediat* or helper# or resistan* or wide or action# or influen*))) | 99 |
| S52 | (TI ((teen or teens or teenage*) N2 (led or leader* or participat* or mediat* or helper# or resistan* or wide or action# or influen*))) OR (AB ((teen or teens or teenage*) N2 (led or leader* or participat* or mediat* or helper# or resistan* or wide or action# or influen*))) | 97 |
| S53 | (TI (adolescen* N2 (led or leader* or participat* or mediat* or helper# or resistan* or wide or action# or influen*))) OR (AB (adolescen* N2 (led or leader* or participat* or mediat* or helper# or resistan* or wide or action# or influen*))) | 219 |
| S54 | (TI (youth# N2 (led or leader* or participat* or mediat* or helper# or resistan* or wide or action# or influen*))) OR (AB (youth# N2 (led or leader* or participat* or mediat* or helper# or resistan* or wide or action# or influen*))) | 489 |
| S55 | (TI (student# N2 (led or leader* or participat* or mediat* or helper# or resistan* or wide or action# or influen* or relationship*))) OR (AB (student# N2 (led or leader* or participat* or mediat* or helper# or resistan* or wide or action# or influen* or relationship*))) | 10,961 |
| S56 | (TI (teach* N2 (led or leader* or participat* or mediat* or helper# or resistan* or wide or action# or influen* or relationship*))) OR (AB (teach* N2 (led or leader* or participat* or mediat* or helper# or resistan* or wide or action# or influen* or relationship*))) | 8,562 |
| S57 | (TI (curricul* N2 (led or leader* or participat* or mediat* or helper# or resistan* or wide or action# or influen* or relationship*))) OR (AB (curricul* N2 (led or leader* or participat* or mediat* or helper# or resistan* or wide or action# or influen* or relationship*))) | 835 |
| S58 | (TI (administrat* N2 (led or leader* or participat* or mediat* or helper# or resistan* or wide or action# or influen* or relationship*))) OR (AB (administrat* N2 (led or leader* or participat* or mediat* or helper# or resistan* or wide or action# or influen* or relationship*))) | 690 |
| S59 | (TI ((personnel or staff) N2 (led or leader* or participat* or mediat* or helper# or resistan* or wide or action# or influen* or relationship*))) OR (AB ((personnel or staff) N2 (led or leader* or participat* or mediat* or helper# or resistan* or wide or action# or influen* or relationship*))) | 582 |
| S60 | (TI (volunteer* N2 (led or leader* or participat* or mediat* or helper# or resistan* or wide or action# or influen* or relationship*))) OR (AB (volulnteer* N2 (led or leader* or participat* or mediat* or helper# or resistan* or wide or action# or influen* or relationship*))) | 13 |
| S61 | (TI ((advocacy or advocate) N2 (led or leader* or participat* or mediat* or helper# or resistan* or wide or action# or influen* or relationship*))) OR (AB ((advocacy or advocate) N2 (led or leader* or participat* or mediat* or helper# or resistan* or wide or action# or influen* or relationship*))) | 242 |
| S62 | (TI (parent* N2 (led or leader* or participat* or mediat* or helper# or resistan* or wide or action# or influen*))) OR (AB (parent* N2 (led or leader* or participat* or mediat* or helper# or resistan* or wide or action# or influen*))) | 1,405 |
| S63 | (TI (environment* N2 (led or leader* or participat* or mediat* or helper# or resistan* or wide or action# or influen* or relationship*))) OR (AB (environment* N2 (led or leader* or participat* or mediat* or helper# or resistan* or wide or action# or influen* or relationship*))) | 999 |
| S64 | (TI ("socio-ecolog*" N2 (led or leader* or participat* or mediat* or helper# or resistan* or wide or action# or influen* or relationship*))) OR (AB ("socio-ecolog*" N2 (led or leader* or participat* or mediat* or helper# or resistan* or wide or action# or influen* or relationship*))) | 3 |
| S65 | (TI (learn* N2 (led or leader* or participat* or mediat* or helper# or resistan* or wide or action# or influen* or relationship*))) OR (AB (learn* N2 (led or leader* or participat* or mediat* or helper# or resistan* or wide or action# or influen* or relationship*))) | 3,863 |
| S66 | (TI ((policy or policies) N2 (led or leader* or participat* or mediat* or helper# or resistan* or wide or action# or influen* or relationship*))) OR (AB ((policy or policies) N2 (led or leader* or participat* or mediat* or helper# or resistan* or wide or action# or influen* or relationship*))) | 1,395 |
| S67 | S45 OR S46 OR S47 OR S48 OR S49 OR S50 OR S51 OR S52 OR S53 OR S54 OR S55 OR S56 OR S57 OR S58 OR S59 OR S60 OR S61 OR S62 OR S63 OR S64 OR S65 OR S66 | 30,451 |
| S68 | S44 AND S67 | 12,810 |
| S69 | S41 OR S42 OR S43 OR S68 | 55,088 |
| S70 | (TI ((smoking or smoked or smoke or smoker) N5 (reduc* or decreas* or increas* or lower* or fewer or improv* or enhance* or extend* or develop* or prevalen* or daily or weekly or current or rate or rates or behavio#r* or knowledge or prevent* or attitude* or avoid* or perceive* or percept* or promot* or program* or change*))) OR (AB ((smoking or smoked or smoke or smoker) N5 (reduc* or decreas* or increas* or lower* or fewer or improv* or enhance* or extend* or develop* or prevalen* or daily or weekly or current or rate or rates or behavio#r* or knowledge or prevent* or attitude* or avoid* or perceive* or percept* or promot* or program* or change*))) | 330 |
| S71 | (TI (tobacco N5 (reduc* or decreas* or increas* or lower* or fewer or improv* or enhance* or extend* or develop* or prevalen* or daily or weekly or current or rate or rates or behavio#r* or knowledge or prevent* or attitude* or avoid* or perceive* or percept* or promot* or program* or change*)) OR (AB (tobacco N5 (reduc* or decreas* or increas* or lower* or fewer or improv* or enhance* or extend* or develop* or prevalen* or daily or weekly or current or rate or rates or behavio#r* or knowledge or prevent* or attitude* or avoid* or perceive* or percept* or promot* or program* or change*)) | 202 |
| S72 | (TI (cigarette# N5 (reduc* or decreas* or increas* or lower* or fewer or improv* or enhance* or extend* or develop* or prevalen* or daily or weekly or current or rate or rates or behavio#r* or knowledge or prevent* or attitude* or avoid* or perceive* or percept* or promot* or program* or change*)) OR (AB (cigarette# N5 (reduc* or decreas* or increas* or lower* or fewer or improv* or enhance* or extend* or develop* or prevalen* or daily or weekly or current or rate or rates or behavio#r* or knowledge or prevent* or attitude* or avoid* or perceive* or percept* or promot* or program* or change*)) | 96 |
| S73 | (TI (marijuana N5 (reduc* or decreas* or increas* or lower* or fewer or improv* or enhance* or extend* or develop* or prevalen* or daily or weekly or current or rate or rates or behavio#r* or knowledge or prevent* or attitude* or avoid* or perceive* or percept* or promot* or program* or change*)) OR (AB (marijuana N5 (reduc* or decreas* or increas* or lower* or fewer or improv* or enhance* or extend* or develop* or prevalen* or daily or weekly or current or rate or rates or behavio#r* or knowledge or prevent* or attitude* or avoid* or perceive* or percept* or promot* or program* or change*)) | 66 |
| S74 | (TI (cannabis N5 (reduc* or decreas* or increas* or lower* or fewer or improv* or enhance* or extend* or develop* or prevalen* or daily or weekly or current or rate or rates or behavio#r* or knowledge or prevent* or attitude* or avoid* or perceive* or percept* or promot* or program* or change*)) OR (AB (cannabis N5 (reduc* or decreas* or increas* or lower* or fewer or improv* or enhance* or extend* or develop* or prevalen* or daily or weekly or current or rate or rates or behavio#r* or knowledge or prevent* or attitude* or avoid* or perceive* or percept* or promot* or program* or change*)) | 24 |
| S75 | (TI (solvent# N5 (reduc* or decreas* or increas* or lower* or fewer or improv* or enhance* or extend* or develop* or prevalen* or daily or weekly or current or rate or rates or behavio#r* or knowledge or prevent* or attitude* or avoid* or perceive* or percept* or promot* or program* or change*)) OR (AB (solvent# N5 (reduc* or decreas* or increas* or lower* or fewer or improv* or enhance* or extend* or develop* or prevalen* or daily or weekly or current or rate or rates or behavio#r* or knowledge or prevent* or attitude* or avoid* or perceive* or percept* or promot* or program* or change*)) | 47 |
| S76 | (TI (alcohol N5 (reduc* or decreas* or increas* or lower* or fewer or improv* or enhance* or extend* or develop* or prevalen* or daily or weekly or current or rate or rates or behavio#r* or knowledge or prevent* or attitude* or avoid* or perceive* or percept* or promot* or program* or change*)) OR (AB (alcohol N5 (reduc* or decreas* or increas* or lower* or fewer or improv* or enhance* or extend* or develop* or prevalen* or daily or weekly or current or rate or rates or behavio#r* or knowledge or prevent* or attitude* or avoid* or perceive* or percept* or promot* or program* or change*)) | 553 |
| S77 | (TI ("binge drink*" N5 (reduc* or decreas* or increas* or lower* or fewer or improv* or enhance* or extend* or develop* or prevalen* or daily or weekly or current or rate or rates or behavio#r* or knowledge or prevent* or attitude* or avoid* or perceive* or percept* or promot* or program* or change*)) OR (AB ("binge drink*" N5 (reduc* or decreas* or increas* or lower* or fewer or improv* or enhance* or extend* or develop* or prevalen* or daily or weekly or current or rate or rates or behavio#r* or knowledge or prevent* or attitude* or avoid* or perceive* or percept* or promot* or program* or change*)) | 37 |
| S78 | (TI ("underage drink*" N5 (reduc* or decreas* or increas* or lower* or fewer or improv* or enhance* or extend* or develop* or prevalen* or daily or weekly or current or rate or rates or behavio#r* or knowledge or prevent* or attitude* or avoid* or perceive* or percept* or promot* or program* or change*)) OR (AB ("underage drink*" N5 (reduc* or decreas* or increas* or lower* or fewer or improv* or enhance* or extend* or develop* or prevalen* or daily or weekly or current or rate or rates or behavio#r* or knowledge or prevent* or attitude* or avoid* or perceive* or percept* or promot* or program* or change*)) | 14 |
| S79 | (TI ("underage drink*" N5 (reduc* or decreas* or increas* or lower* or fewer or improv* or enhance* or extend* or develop* or prevalen* or daily or weekly or current or rate or rates or behavio#r* or knowledge or prevent* or attitude* or avoid* or perceive* or percept* or promot* or program* or change*)) OR (AB ("underage drink*" N5 (reduc* or decreas* or increas* or lower* or fewer or improv* or enhance* or extend* or develop* or prevalen* or daily or weekly or current or rate or rates or behavio#r* or knowledge or prevent* or attitude* or avoid* or perceive* or percept* or promot* or program* or change*)) | 2,612 |
| S80 | (TI (substance# N5 (reduc* or decreas* or increas* or lower* or fewer or improv* or enhance* or extend* or develop* or prevalen* or daily or weekly or current or rate or rates or behavio#r* or knowledge or prevent* or attitude* or avoid* or perceive* or percept* or promot* or program* or change*)) OR (AB (substance# N5 (reduc* or decreas* or increas* or lower* or fewer or improv* or enhance* or extend* or develop* or prevalen* or daily or weekly or current or rate or rates or behavio#r* or knowledge or prevent* or attitude* or avoid* or perceive* or percept* or promot* or program* or change*)) | 413 |
| S81 | (TI (drug# N5 (reduc* or decreas* or increas* or lower* or fewer or improv* or enhance* or extend* or develop* or prevalen* or daily or weekly or current or rate or rates or behavio#r* or knowledge or prevent* or attitude* or avoid* or perceive* or percept* or promot* or program* or change*)) OR (AB (drug# N5 (reduc* or decreas* or increas* or lower* or fewer or improv* or enhance* or extend* or develop* or prevalen* or daily or weekly or current or rate or rates or behavio#r* or knowledge or prevent* or attitude* or avoid* or perceive* or percept* or promot* or program* or change*)) | 1,053 |
| S82 | (TI ("education* attain*" N5 (reduc* or decreas* or increas* or lower* or fewer or improv* or enhance* or extend* or develop* or prevalen* or daily or weekly or current or rate or rates or behavio#r* or knowledge or prevent* or attitude* or avoid* or perceive* or percept* or promot* or program* or change*)) OR (AB ("education* attain*" N5 (reduc* or decreas* or increas* or lower* or fewer or improv* or enhance* or extend* or develop* or prevalen* or daily or weekly or current or rate or rates or behavio#r* or knowledge or prevent* or attitude* or avoid* or perceive* or percept* or promot* or program* or change*)) | 256 |
| S83 | (TI ("academic attain*" N5 (reduc* or decreas* or increas* or lower* or fewer or improv* or enhance* or extend* or develop* or prevalen* or daily or weekly or current or rate or rates or behavio#r* or knowledge or prevent* or attitude* or avoid* or perceive* or percept* or promot* or program* or change*)) OR (AB ("academic attain*" N5 (reduc* or decreas* or increas* or lower* or fewer or improv* or enhance* or extend* or develop* or prevalen* or daily or weekly or current or rate or rates or behavio#r* or knowledge or prevent* or attitude* or avoid* or perceive* or percept* or promot* or program* or change*)) | 24 |
| S84 | (TI ("student* attain*" N5 (reduc* or decreas* or increas* or lower* or fewer or improv* or enhance* or extend* or develop* or prevalen* or daily or weekly or current or rate or rates or behavio#r* or knowledge or prevent* or attitude* or avoid* or perceive* or percept* or promot* or program* or change*)) OR (AB ("student* attain*" N5 (reduc* or decreas* or increas* or lower* or fewer or improv* or enhance* or extend* or develop* or prevalen* or daily or weekly or current or rate or rates or behavio#r* or knowledge or prevent* or attitude* or avoid* or perceive* or percept* or promot* or program* or change*)) | 40 |
| S85 | (TI ("education* achieve*" N5 (reduc* or decreas* or increas* or lower* or fewer or improv* or enhance* or extend* or develop* or prevalen* or daily or weekly or current or rate or rates or behavio#r* or knowledge or prevent* or attitude* or avoid* or perceive* or percept* or promot* or program* or change*)) OR (AB ("education* achieve*" N5 (reduc* or decreas* or increas* or lower* or fewer or improv* or enhance* or extend* or develop* or prevalen* or daily or weekly or current or rate or rates or behavio#r* or knowledge or prevent* or attitude* or avoid* or perceive* or percept* or promot* or program* or change*)) | 131 |
| S86 | (TI ("academic achieve*" N5 (reduc* or decreas* or increas* or lower* or fewer or improv* or enhance* or extend* or develop* or prevalen* or daily or weekly or current or rate or rates or behavio#r* or knowledge or prevent* or attitude* or avoid* or perceive* or percept* or promot* or program* or change*)) OR (AB ("academic achieve*" N5 (reduc* or decreas* or increas* or lower* or fewer or improv* or enhance* or extend* or develop* or prevalen* or daily or weekly or current or rate or rates or behavio#r* or knowledge or prevent* or attitude* or avoid* or perceive* or percept* or promot* or program* or change*)) | 1,437 |
| S87 | (TI ("student* achieve*" N5 (reduc* or decreas* or increas* or lower* or fewer or improv* or enhance* or extend* or develop* or prevalen* or daily or weekly or current or rate or rates or behavio#r* or knowledge or prevent* or attitude* or avoid* or perceive* or percept* or promot* or program* or change*)) OR (AB ("student* achieve*" N5 (reduc* or decreas* or increas* or lower* or fewer or improv* or enhance* or extend* or develop* or prevalen* or daily or weekly or current or rate or rates or behavio#r* or knowledge or prevent* or attitude* or avoid* or perceive* or percept* or promot* or program* or change*)) | 1,465 |
| S88 | (TI ("education* perform*" N5 (reduc* or decreas* or increas* or lower* or fewer or improv* or enhance* or extend* or develop* or prevalen* or daily or weekly or current or rate or rates or behavio#r* or knowledge or prevent* or attitude* or avoid* or perceive* or percept* or promot* or program* or change*)) OR (AB ("education* perform*" N5 (reduc* or decreas* or increas* or lower* or fewer or improv* or enhance* or extend* or develop* or prevalen* or daily or weekly or current or rate or rates or behavio#r* or knowledge or prevent* or attitude* or avoid* or perceive* or percept* or promot* or program* or change*)) | 78 |
| S89 | (TI ("academic perform*" N5 (reduc* or decreas* or increas* or lower* or fewer or improv* or enhance* or extend* or develop* or prevalen* or daily or weekly or current or rate or rates or behavio#r* or knowledge or prevent* or attitude* or avoid* or perceive* or percept* or promot* or program* or change*)) OR (AB ("academic perform*" N5 (reduc* or decreas* or increas* or lower* or fewer or improv* or enhance* or extend* or develop* or prevalen* or daily or weekly or current or rate or rates or behavio#r* or knowledge or prevent* or attitude* or avoid* or perceive* or percept* or promot* or program* or change*)) | 792 |
| S90 | (TI ("student* perform*" N5 (reduc* or decreas* or increas* or lower* or fewer or improv* or enhance* or extend* or develop* or prevalen* or daily or weekly or current or rate or rates or behavio#r* or knowledge or prevent* or attitude* or avoid* or perceive* or percept* or promot* or program* or change*)) OR (AB ("student* perform*" N5 (reduc* or decreas* or increas* or lower* or fewer or improv* or enhance* or extend* or develop* or prevalen* or daily or weekly or current or rate or rates or behavio#r* or knowledge or prevent* or attitude* or avoid* or perceive* or percept* or promot* or program* or change*)) | 886 |
| S91 | (TI ("education* outcome#" N5 (reduc* or decreas* or increas* or lower* or fewer or improv* or enhance* or extend* or develop* or prevalen* or daily or weekly or current or rate or rates or behavio#r* or knowledge or prevent* or attitude* or avoid* or perceive* or percept* or promot* or program* or change*)) OR (AB ("education* outcome#" N5 (reduc* or decreas* or increas* or lower* or fewer or improv* or enhance* or extend* or develop* or prevalen* or daily or weekly or current or rate or rates or behavio#r* or knowledge or prevent* or attitude* or avoid* or perceive* or percept* or promot* or program* or change*)) | 274 |
| S92 | (TI ("academic outcome#" N5 (reduc* or decreas* or increas* or lower* or fewer or improv* or enhance* or extend* or develop* or prevalen* or daily or weekly or current or rate or rates or behavio#r* or knowledge or prevent* or attitude* or avoid* or perceive* or percept* or promot* or program* or change*)) OR (AB ("academic outcome#" N5 (reduc* or decreas* or increas* or lower* or fewer or improv* or enhance* or extend* or develop* or prevalen* or daily or weekly or current or rate or rates or behavio#r* or knowledge or prevent* or attitude* or avoid* or perceive* or percept* or promot* or program* or change*)) | 138 |
| S93 | (TI ("student* outcome#" N5 (reduc* or decreas* or increas* or lower* or fewer or improv* or enhance* or extend* or develop* or prevalen* or daily or weekly or current or rate or rates or behavio#r* or knowledge or prevent* or attitude* or avoid* or perceive* or percept* or promot* or program* or change*)) OR (AB ("student* outcome#" N5 (reduc* or decreas* or increas* or lower* or fewer or improv* or enhance* or extend* or develop* or prevalen* or daily or weekly or current or rate or rates or behavio#r* or knowledge or prevent* or attitude* or avoid* or perceive* or percept* or promot* or program* or change*)) | 336 |
| S94 | (TI ("education* commitment" N5 (reduc* or decreas* or increas* or lower* or fewer or improv* or enhance* or extend* or develop* or prevalen* or daily or weekly or current or rate or rates or behavio#r* or knowledge or prevent* or attitude* or avoid* or perceive* or percept* or promot* or program* or change*)) OR (AB ("education* commitment" N5 (reduc* or decreas* or increas* or lower* or fewer or improv* or enhance* or extend* or develop* or prevalen* or daily or weekly or current or rate or rates or behavio#r* or knowledge or prevent* or attitude* or avoid* or perceive* or percept* or promot* or program* or change*)) | 10 |
| S95 | (TI ("education* commitment" N5 (reduc* or decreas* or increas* or lower* or fewer or improv* or enhance* or extend* or develop* or prevalen* or daily or weekly or current or rate or rates or behavio#r* or knowledge or prevent* or attitude* or avoid* or perceive* or percept* or promot* or program* or change*)) OR (AB ("education* commitment" N5 (reduc* or decreas* or increas* or lower* or fewer or improv* or enhance* or extend* or develop* or prevalen* or daily or weekly or current or rate or rates or behavio#r* or knowledge or prevent* or attitude* or avoid* or perceive* or percept* or promot* or program* or change*)) | 2,947 |
| S96 | (TI ("academic commitment" N5 (reduc* or decreas* or increas* or lower* or fewer or improv* or enhance* or extend* or develop* or prevalen* or daily or weekly or current or rate or rates or behavio#r* or knowledge or prevent* or attitude* or avoid* or perceive* or percept* or promot* or program* or change*)) OR (AB ("academic commitment" N5 (reduc* or decreas* or increas* or lower* or fewer or improv* or enhance* or extend* or develop* or prevalen* or daily or weekly or current or rate or rates or behavio#r* or knowledge or prevent* or attitude* or avoid* or perceive* or percept* or promot* or program* or change*)) | 2,947 |
| S97 | (TI ("academic commitment" N5 (reduc* or decreas* or increas* or lower* or fewer or improv* or enhance* or extend* or develop* or prevalen* or daily or weekly or current or rate or rates or behavio#r* or knowledge or prevent* or attitude* or avoid* or perceive* or percept* or promot* or program* or change*)) OR (AB ("academic commitment" N5 (reduc* or decreas* or increas* or lower* or fewer or improv* or enhance* or extend* or develop* or prevalen* or daily or weekly or current or rate or rates or behavio#r* or knowledge or prevent* or attitude* or avoid* or perceive* or percept* or promot* or program* or change*)) | 2,947 |
| S98 | (TI ("student* commitment" N5 (reduc* or decreas* or increas* or lower* or fewer or improv* or enhance* or extend* or develop* or prevalen* or daily or weekly or current or rate or rates or behavio#r* or knowledge or prevent* or attitude* or avoid* or perceive* or percept* or promot* or program* or change*)) OR (AB ("student* commitment" N5 (reduc* or decreas* or increas* or lower* or fewer or improv* or enhance* or extend* or develop* or prevalen* or daily or weekly or current or rate or rates or behavio#r* or knowledge or prevent* or attitude* or avoid* or perceive* or percept* or promot* or program* or change*)) | 14 |
| S99 | (TI ("education* engage*" N5 (reduc* or decreas* or increas* or lower* or fewer or improv* or enhance* or extend* or develop* or prevalen* or daily or weekly or current or rate or rates or behavio#r* or knowledge or prevent* or attitude* or avoid* or perceive* or percept* or promot* or program* or change*)) OR (AB ("education* engage*" N5 (reduc* or decreas* or increas* or lower* or fewer or improv* or enhance* or extend* or develop* or prevalen* or daily or weekly or current or rate or rates or behavio#r* or knowledge or prevent* or attitude* or avoid* or perceive* or percept* or promot* or program* or change*)) | 11 |
| S100 | (TI ("academic engage*" N5 (reduc* or decreas* or increas* or lower* or fewer or improv* or enhance* or extend* or develop* or prevalen* or daily or weekly or current or rate or rates or behavio#r* or knowledge or prevent* or attitude* or avoid* or perceive* or percept* or promot* or program* or change*)) OR (AB ("academic engage*" N5 (reduc* or decreas* or increas* or lower* or fewer or improv* or enhance* or extend* or develop* or prevalen* or daily or weekly or current or rate or rates or behavio#r* or knowledge or prevent* or attitude* or avoid* or perceive* or percept* or promot* or program* or change*)) | 97 |
| S101 | (TI ("student* engage*" N5 (reduc* or decreas* or increas* or lower* or fewer or improv* or enhance* or extend* or develop* or prevalen* or daily or weekly or current or rate or rates or behavio#r* or knowledge or prevent* or attitude* or avoid* or perceive* or percept* or promot* or program* or change*)) OR (AB ("student* engage*" N5 (reduc* or decreas* or increas* or lower* or fewer or improv* or enhance* or extend* or develop* or prevalen* or daily or weekly or current or rate or rates or behavio#r* or knowledge or prevent* or attitude* or avoid* or perceive* or percept* or promot* or program* or change*)) | 735 |
| S102 | (TI (dropout N5 (reduc* or decreas* or increas* or lower* or fewer or improv* or enhance* or extend* or develop* or prevalen* or daily or weekly or current or rate or rates or behavio#r* or knowledge or prevent* or attitude* or avoid* or perceive* or percept* or promot* or program* or change*)) OR (AB (dropout N5 (reduc* or decreas* or increas* or lower* or fewer or improv* or enhance* or extend* or develop* or prevalen* or daily or weekly or current or rate or rates or behavio#r* or knowledge or prevent* or attitude* or avoid* or perceive* or percept* or promot* or program* or change*)) | 1,214 |
| S103 | (TI (absentee* N5 (reduc* or decreas* or increas* or lower* or fewer or improv* or enhance* or extend* or develop* or prevalen* or daily or weekly or current or rate or rates or behavio#r* or knowledge or prevent* or attitude* or avoid* or perceive* or percept* or promot* or program* or change*)) OR (AB (absentee* N5 (reduc* or decreas* or increas* or lower* or fewer or improv* or enhance* or extend* or develop* or prevalen* or daily or weekly or current or rate or rates or behavio#r* or knowledge or prevent* or attitude* or avoid* or perceive* or percept* or promot* or program* or change*)) | 209 |
| S104 | (TI ((violent or violence) N5 (reduc* or decreas* or increas* or lower* or fewer or improv* or enhance* or extend* or develop* or prevalen* or daily or weekly or current or rate or rates or behavio#r* or knowledge or prevent* or attitude* or avoid* or perceive* or percept* or promot* or program* or change*))) OR (AB ((violent or violence) N5 (reduc* or decreas* or increas* or lower* or fewer or improv* or enhance* or extend* or develop* or prevalen* or daily or weekly or current or rate or rates or behavio#r* or knowledge or prevent* or attitude* or avoid* or perceive* or percept* or promot* or program* or change*))) | 1,504 |
| S105 | (TI ((aggression or aggressive) N5 (reduc* or decreas* or increas* or lower* or fewer or improv* or enhance* or extend* or develop* or prevalen* or daily or weekly or current or rate or rates or behavio#r* or knowledge or prevent* or attitude* or avoid* or perceive* or percept* or promot* or program* or change*))) OR (AB ((aggression or aggressive) N5 (reduc* or decreas* or increas* or lower* or fewer or improv* or enhance* or extend* or develop* or prevalen* or daily or weekly or current or rate or rates or behavio#r* or knowledge or prevent* or attitude* or avoid* or perceive* or percept* or promot* or program* or change*))) | 450 |
| S106 | (TI ((hostile or hostility) N5 (reduc* or decreas* or increas* or lower* or fewer or improv* or enhance* or extend* or develop* or prevalen* or daily or weekly or current or rate or rates or behavio#r* or knowledge or prevent* or attitude* or avoid* or perceive* or percept* or promot* or program* or change*))) OR (AB ((hostile or hostility) N5 (reduc* or decreas* or increas* or lower* or fewer or improv* or enhance* or extend* or develop* or prevalen* or daily or weekly or current or rate or rates or behavio#r* or knowledge or prevent* or attitude* or avoid* or perceive* or percept* or promot* or program* or change*))) | 92 |
| S107 | (TI (assault* N5 (reduc* or decreas* or increas* or lower* or fewer or improv* or enhance* or extend* or develop* or prevalen* or daily or weekly or current or rate or rates or behavio#r* or knowledge or prevent* or attitude* or avoid* or perceive* or percept* or promot* or program* or change*)) OR (AB (assault* N5 (reduc* or decreas* or increas* or lower* or fewer or improv* or enhance* or extend* or develop* or prevalen* or daily or weekly or current or rate or rates or behavio#r* or knowledge or prevent* or attitude* or avoid* or perceive* or percept* or promot* or program* or change*)) | 146 |
| S108 | (TI (crime N5 (reduc* or decreas* or increas* or lower* or fewer or improv* or enhance* or extend* or develop* or prevalen* or daily or weekly or current or rate or rates or behavio#r* or knowledge or prevent* or attitude* or avoid* or perceive* or percept* or promot* or program* or change*)) OR (AB (crime N5 (reduc* or decreas* or increas* or lower* or fewer or improv* or enhance* or extend* or develop* or prevalen* or daily or weekly or current or rate or rates or behavio#r* or knowledge or prevent* or attitude* or avoid* or perceive* or percept* or promot* or program* or change*)) | 628 |
| S109 | (TI ((abuse# or abusive) N5 (reduc* or decreas* or increas* or lower* or fewer or improv* or enhance* or extend* or develop* or prevalen* or daily or weekly or current or rate or rates or behavio#r* or knowledge or prevent* or attitude* or avoid* or perceive* or percept* or promot* or program* or change*))) OR (AB ((abuse# or abusive) N5 (reduc* or decreas* or increas* or lower* or fewer or improv* or enhance* or extend* or develop* or prevalen* or daily or weekly or current or rate or rates or behavio#r* or knowledge or prevent* or attitude* or avoid* or perceive* or percept* or promot* or program* or change*))) | 819 |
| S110 | (TI (misbehav* N5 (reduc* or decreas* or increas* or lower* or fewer or improv* or enhance* or extend* or develop* or prevalen* or daily or weekly or current or rate or rates or behavio#r* or knowledge or prevent* or attitude* or avoid* or perceive* or percept* or promot* or program* or change*)) OR (AB (misbehav* N5 (reduc* or decreas* or increas* or lower* or fewer or improv* or enhance* or extend* or develop* or prevalen* or daily or weekly or current or rate or rates or behavio#r* or knowledge or prevent* or attitude* or avoid* or perceive* or percept* or promot* or program* or change*)) | 106 |
| S111 | (TI (threat# N5 (reduc* or decreas* or increas* or lower* or fewer or improv* or enhance* or extend* or develop* or prevalen* or daily or weekly or current or rate or rates or behavio#r* or knowledge or prevent* or attitude* or avoid* or perceive* or percept* or promot* or program* or change*)) OR (AB (threat# N5 (reduc* or decreas* or increas* or lower* or fewer or improv* or enhance* or extend* or develop* or prevalen* or daily or weekly or current or rate or rates or behavio#r* or knowledge or prevent* or attitude* or avoid* or perceive* or percept* or promot* or program* or change*)) | 470 |
| S112 | (TI (conduct N5 (reduc* or decreas* or increas* or lower* or fewer or improv* or enhance* or extend* or develop* or prevalen* or daily or weekly or current or rate or rates or behavio#r* or knowledge or prevent* or attitude* or avoid* or perceive* or percept* or promot* or program* or change*)) OR (AB (conduct N5 (reduc* or decreas* or increas* or lower* or fewer or improv* or enhance* or extend* or develop* or prevalen* or daily or weekly or current or rate or rates or behavio#r* or knowledge or prevent* or attitude* or avoid* or perceive* or percept* or promot* or program* or change*)) | 542 |
| S113 | (TI (delinquen* N5 (reduc* or decreas* or increas* or lower* or fewer or improv* or enhance* or extend* or develop* or prevalen* or daily or weekly or current or rate or rates or behavio#r* or knowledge or prevent* or attitude* or avoid* or perceive* or percept* or promot* or program* or change*)) OR (AB (delinquen* N5 (reduc* or decreas* or increas* or lower* or fewer or improv* or enhance* or extend* or develop* or prevalen* or daily or weekly or current or rate or rates or behavio#r* or knowledge or prevent* or attitude* or avoid* or perceive* or percept* or promot* or program* or change*)) | 155 |
| S114 | (TI ("disrupt* behavio#r*" N5 (reduc* or decreas* or increas* or lower* or fewer or improv* or enhance* or extend* or develop* or prevalen* or daily or weekly or current or rate or rates or behavio#r* or knowledge or prevent* or attitude* or avoid* or perceive* or percept* or promot* or program* or change*)) OR (AB ("disrupt* behavio#r*" N5 (reduc* or decreas* or increas* or lower* or fewer or improv* or enhance* or extend* or develop* or prevalen* or daily or weekly or current or rate or rates or behavio#r* or knowledge or prevent* or attitude* or avoid* or perceive* or percept* or promot* or program* or change*)) | 331 |
| S115 | (TI ("problem* behavio#r*" N5 (reduc* or decreas* or increas* or lower* or fewer or improv* or enhance* or extend* or develop* or prevalen* or daily or weekly or current or rate or rates or behavio#r* or knowledge or prevent* or attitude* or avoid* or perceive* or percept* or promot* or program* or change*)) OR (AB ("problem* behavio#r*" N5 (reduc* or decreas* or increas* or lower* or fewer or improv* or enhance* or extend* or develop* or prevalen* or daily or weekly or current or rate or rates or behavio#r* or knowledge or prevent* or attitude* or avoid* or perceive* or percept* or promot* or program* or change*)) | 427 |
| S116 | (TI ("volatile behavio#r*" N5 (reduc* or decreas* or increas* or lower* or fewer or improv* or enhance* or extend* or develop* or prevalen* or daily or weekly or current or rate or rates or behavio#r* or knowledge or prevent* or attitude* or avoid* or perceive* or percept* or promot* or program* or change*)) OR (AB ("volatile behavio#r*" N5 (reduc* or decreas* or increas* or lower* or fewer or improv* or enhance* or extend* or develop* or prevalen* or daily or weekly or current or rate or rates or behavio#r* or knowledge or prevent* or attitude* or avoid* or perceive* or percept* or promot* or program* or change*)) | 1 |
| S117 | (TI ("volatile behavio#r*" N5 (reduc* or decreas* or increas* or lower* or fewer or improv* or enhance* or extend* or develop* or prevalen* or daily or weekly or current or rate or rates or behavio#r* or knowledge or prevent* or attitude* or avoid* or perceive* or percept* or promot* or program* or change*)) OR (AB ("volatile behavio#r*" N5 (reduc* or decreas* or increas* or lower* or fewer or improv* or enhance* or extend* or develop* or prevalen* or daily or weekly or current or rate or rates or behavio#r* or knowledge or prevent* or attitude* or avoid* or perceive* or percept* or promot* or program* or change*)) | 2,845 |
| S118 | (TI ((antisocial or "anti-social") N5 (reduc* or decreas* or increas* or lower* or fewer or improv* or enhance* or extend* or develop* or prevalen* or daily or weekly or current or rate or rates or behavio#r* or knowledge or prevent* or attitude* or avoid* or perceive* or percept* or promot* or program* or change*))) OR (AB ((antisocial or "anti-social") N5 (reduc* or decreas* or increas* or lower* or fewer or improv* or enhance* or extend* or develop* or prevalen* or daily or weekly or current or rate or rates or behavio#r* or knowledge or prevent* or attitude* or avoid* or perceive* or percept* or promot* or program* or change*))) | 241 |
| S119 | (TI (perpetrat* N5 (reduc* or decreas* or increas* or lower* or fewer or improv* or enhance* or extend* or develop* or prevalen* or daily or weekly or current or rate or rates or behavio#r* or knowledge or prevent* or attitude* or avoid* or perceive* or percept* or promot* or program* or change*)) OR (AB (perpetrat* N5 (reduc* or decreas* or increas* or lower* or fewer or improv* or enhance* or extend* or develop* or prevalen* or daily or weekly or current or rate or rates or behavio#r* or knowledge or prevent* or attitude* or avoid* or perceive* or percept* or promot* or program* or change*)) | 40 |
| S120 | (TI (bully* N5 (reduc* or decreas* or increas* or lower* or fewer or improv* or enhance* or extend* or develop* or prevalen* or daily or weekly or current or rate or rates or behavio#r* or knowledge or prevent* or attitude* or avoid* or perceive* or percept* or promot* or program* or change*)) OR (AB (bully* N5 (reduc* or decreas* or increas* or lower* or fewer or improv* or enhance* or extend* or develop* or prevalen* or daily or weekly or current or rate or rates or behavio#r* or knowledge or prevent* or attitude* or avoid* or perceive* or percept* or promot* or program* or change*)) | 888 |
| S121 | (TI (victim* N5 (reduc* or decreas* or increas* or lower* or fewer or improv* or enhance* or extend* or develop* or prevalen* or daily or weekly or current or rate or rates or behavio#r* or knowledge or prevent* or attitude* or avoid* or perceive* or percept* or promot* or program* or change*)) OR (AB (victim* N5 (reduc* or decreas* or increas* or lower* or fewer or improv* or enhance* or extend* or develop* or prevalen* or daily or weekly or current or rate or rates or behavio#r* or knowledge or prevent* or attitude* or avoid* or perceive* or percept* or promot* or program* or change*)) | 374 |
| S122 | (TI (cyberbully* N5 (reduc* or decreas* or increas* or lower* or fewer or improv* or enhance* or extend* or develop* or prevalen* or daily or weekly or current or rate or rates or behavio#r* or knowledge or prevent* or attitude* or avoid* or perceive* or percept* or promot* or program* or change*)) OR (AB (cyberbully* N5 (reduc* or decreas* or increas* or lower* or fewer or improv* or enhance* or extend* or develop* or prevalen* or daily or weekly or current or rate or rates or behavio#r* or knowledge or prevent* or attitude* or avoid* or perceive* or percept* or promot* or program* or change*)) | 83 |
| S123 | (TI (conflict* N5 (reduc* or decreas* or increas* or lower* or fewer or improv* or enhance* or extend* or develop* or prevalen* or daily or weekly or current or rate or rates or behavio#r* or knowledge or prevent* or attitude* or avoid* or perceive* or percept* or promot* or program* or change*)) OR (AB (conflict* N5 (reduc* or decreas* or increas* or lower* or fewer or improv* or enhance* or extend* or develop* or prevalen* or daily or weekly or current or rate or rates or behavio#r* or knowledge or prevent* or attitude* or avoid* or perceive* or percept* or promot* or program* or change*)) | 1,045 |
| S124 | (TI ("positive behavio#r*" N5 (reduc* or decreas* or increas* or lower* or fewer or improv* or enhance* or extend* or develop* or prevalen* or daily or weekly or current or rate or rates or behavio#r* or knowledge or prevent* or attitude* or avoid* or perceive* or percept* or promot* or program* or change*)) OR (AB ("positive behavio#r*" N5 (reduc* or decreas* or increas* or lower* or fewer or improv* or enhance* or extend* or develop* or prevalen* or daily or weekly or current or rate or rates or behavio#r* or knowledge or prevent* or attitude* or avoid* or perceive* or percept* or promot* or program* or change*)) | 420 |
| S125 | (TI ("improv* behavio#r*" N5 (reduc* or decreas* or increas* or lower* or fewer or improv* or enhance* or extend* or develop* or prevalen* or daily or weekly or current or rate or rates or behavio#r* or knowledge or prevent* or attitude* or avoid* or perceive* or percept* or promot* or program* or change*)) OR (AB ("improv* behavio#r*" N5 (reduc* or decreas* or increas* or lower* or fewer or improv* or enhance* or extend* or develop* or prevalen* or daily or weekly or current or rate or rates or behavio#r* or knowledge or prevent* or attitude* or avoid* or perceive* or percept* or promot* or program* or change*)) | 68 |
| S126 | (TI ("social behavio#r*" N5 (reduc* or decreas* or increas* or lower* or fewer or improv* or enhance* or extend* or develop* or prevalen* or daily or weekly or current or rate or rates or behavio#r* or knowledge or prevent* or attitude* or avoid* or perceive* or percept* or promot* or program* or change*)) OR (AB ("social behavio#r*" N5 (reduc* or decreas* or increas* or lower* or fewer or improv* or enhance* or extend* or develop* or prevalen* or daily or weekly or current or rate or rates or behavio#r* or knowledge or prevent* or attitude* or avoid* or perceive* or percept* or promot* or program* or change*)) | 428 |
| S127 | (TI (("social-emotion*" or "socio-emotion*") N5 (reduc* or decreas* or increas* or lower* or fewer or improv* or enhance* or extend* or develop* or prevalen* or daily or weekly or current or rate or rates or behavio#r* or knowledge or prevent* or attitude* or avoid* or perceive* or percept* or promot* or program* or change*))) OR (AB (("social-emotion*" or "socio-emotion*") N5 (reduc* or decreas* or increas* or lower* or fewer or improv* or enhance* or extend* or develop* or prevalen* or daily or weekly or current or rate or rates or behavio#r* or knowledge or prevent* or attitude* or avoid* or perceive* or percept* or promot* or program* or change*))) | 322 |
| S128 | (TI ("social-charact*" N5 (reduc* or decreas* or increas* or lower* or fewer or improv* or enhance* or extend* or develop* or prevalen* or daily or weekly or current or rate or rates or behavio#r* or knowledge or prevent* or attitude* or avoid* or perceive* or percept* or promot* or program* or change*)) OR (AB ("social-charact*" N5 (reduc* or decreas* or increas* or lower* or fewer or improv* or enhance* or extend* or develop* or prevalen* or daily or weekly or current or rate or rates or behavio#r* or knowledge or prevent* or attitude* or avoid* or perceive* or percept* or promot* or program* or change*)) | 4 |
| S129 | (TI ("social-inclusion" N5 (reduc* or decreas* or increas* or lower* or fewer or improv* or enhance* or extend* or develop* or prevalen* or daily or weekly or current or rate or rates or behavio#r* or knowledge or prevent* or attitude* or avoid* or perceive* or percept* or promot* or program* or change*)) OR (AB ("social-inclusion" N5 (reduc* or decreas* or increas* or lower* or fewer or improv* or enhance* or extend* or develop* or prevalen* or daily or weekly or current or rate or rates or behavio#r* or knowledge or prevent* or attitude* or avoid* or perceive* or percept* or promot* or program* or change*)) | 58 |
| S130 | (TI ("social develop*" N5 (reduc* or decreas* or increas* or lower* or fewer or improv* or enhance* or extend* or develop* or prevalen* or daily or weekly or current or rate or rates or behavio#r* or knowledge or prevent* or attitude* or avoid* or perceive* or percept* or promot* or program* or change*)) OR (AB ("social develop*" N5 (reduc* or decreas* or increas* or lower* or fewer or improv* or enhance* or extend* or develop* or prevalen* or daily or weekly or current or rate or rates or behavio#r* or knowledge or prevent* or attitude* or avoid* or perceive* or percept* or promot* or program* or change*)) | 504 |
| S131 | (TI ("social competen*" N5 (reduc* or decreas* or increas* or lower* or fewer or improv* or enhance* or extend* or develop* or prevalen* or daily or weekly or current or rate or rates or behavio#r* or knowledge or prevent* or attitude* or avoid* or perceive* or percept* or promot* or program* or change*)) OR (AB ("social competen*" N5 (reduc* or decreas* or increas* or lower* or fewer or improv* or enhance* or extend* or develop* or prevalen* or daily or weekly or current or rate or rates or behavio#r* or knowledge or prevent* or attitude* or avoid* or perceive* or percept* or promot* or program* or change*)) | 121 |
| S132 | (TI ("emotion* develop*" N5 (reduc* or decreas* or increas* or lower* or fewer or improv* or enhance* or extend* or develop* or prevalen* or daily or weekly or current or rate or rates or behavio#r* or knowledge or prevent* or attitude* or avoid* or perceive* or percept* or promot* or program* or change*)) OR (AB ("emotion* develop*" N5 (reduc* or decreas* or increas* or lower* or fewer or improv* or enhance* or extend* or develop* or prevalen* or daily or weekly or current or rate or rates or behavio#r* or knowledge or prevent* or attitude* or avoid* or perceive* or percept* or promot* or program* or change*)) | 250 |
| S133 | (TI (conduct N5 (reduc* or decreas* or increas* or lower* or fewer or improv* or enhance* or extend* or develop* or prevalen* or daily or weekly or current or rate or rates or behavio#r* or knowledge or prevent* or attitude* or avoid* or perceive* or percept* or promot* or program* or change*)) OR (AB (conduct N5 (reduc* or decreas* or increas* or lower* or fewer or improv* or enhance* or extend* or develop* or prevalen* or daily or weekly or current or rate or rates or behavio#r* or knowledge or prevent* or attitude* or avoid* or perceive* or percept* or promot* or program* or change*)) | 542 |
| S134 | (TI (respect N5 (reduc* or decreas* or increas* or lower* or fewer or improv* or enhance* or extend* or develop* or prevalen* or daily or weekly or current or rate or rates or behavio#r* or knowledge or prevent* or attitude* or avoid* or perceive* or percept* or promot* or program* or change*)) OR (AB (respect N5 (reduc* or decreas* or increas* or lower* or fewer or improv* or enhance* or extend* or develop* or prevalen* or daily or weekly or current or rate or rates or behavio#r* or knowledge or prevent* or attitude* or avoid* or perceive* or percept* or promot* or program* or change*)) | 735 |
| S135 | (TI ((safety or safe) N5 (reduc* or decreas* or increas* or lower* or fewer or improv* or enhance* or extend* or develop* or prevalen* or daily or weekly or current or rate or rates or behavio#r* or knowledge or prevent* or attitude* or avoid* or perceive* or percept* or promot* or program* or change*))) OR (AB ((safety or safe) N5 (reduc* or decreas* or increas* or lower* or fewer or improv* or enhance* or extend* or develop* or prevalen* or daily or weekly or current or rate or rates or behavio#r* or knowledge or prevent* or attitude* or avoid* or perceive* or percept* or promot* or program* or change*))) | 1,533 |
| S136 | S70 OR S71 OR S72 OR S73 OR S74 OR S75 OR S76 OR S77 OR S78 OR S79 OR S80 OR S81 OR S82 OR S83 OR S84 OR S85 OR S86 OR S87 OR S88 OR S89 OR S90 OR S91 OR S92 OR S93 OR S94 OR S95 OR S96 OR S97 OR S98 OR S99 OR S100 OR S101 OR S102 OR S103 OR S104 OR S105 OR S106 OR S107 OR S108 OR S109 OR S110 OR S111 OR S112 OR S113 OR S114 OR S115 OR S116 OR S117 OR S118 OR S119 OR S120 OR S121 OR S122 OR S123 OR S124 OR S125 OR S126 OR S127 OR S128 OR S129 OR S130 OR S131 OR S132 OR S133 OR S134 OR S135 | 20,184 |
| S137 | (TI prevent*) OR (AB prevent*) | 10,689 |
| S138 | (TI program*) OR (AB program*) | 94,867 |
| S139 | (TI intervent*) OR (AB intervent*) | 12,878 |
| S140 | (TI "restorative approach*") OR (AB "restorative approach*") | 8 |
| S141 | (TI "control school#") OR (AB "control school#") | 119 |
| S142 | (TI (trial or trials)) OR (AB (trial or trials)) | 3,568 |
| S143 | S137 OR S138 OR S139 OR S140 OR S141 OR S142 | 114,531 |
| S144 | S40 AND S69 AND S136 AND S143 | 1,987 |

## Trials Register of Promoting Health Intervention (TRoPHI)

| Database name | Trials Register of Promoting Health Information (TRoPHI) |
| --- | --- |
| Database platform | <http://eppi.ioe.ac.uk/webdatabases4/Search.aspx> |
| Dates of database coverage | Complete database to date searched. |
| Date searched | 27/01/2020 |
| Searched by | JF |
| Number of results | 0 |
| EndNote import order | N/A |
| Number of results once duplicates removed | N/A |
| Search strategy notes | None |

Freetext: school OR schools OR schoolwide

## US Institution of Education Sciences, What Works Clearinghouse

| Database name | US Institution of Education Sciences, What Works Clearinghouse |
| --- | --- |
| Database platform | <http://ies.ed.gov/funding/grantsearch/index.asp> |
| Dates of database coverage | Complete database to date searched |
| Date searched | 27/01/2020 |
| Searched by | JF |
| Number of results | Not known |
| EndNote import order | N/A |
| Number of results once duplicates removed | N/A |
| Search strategy notes | None |

Website was not loading so this source was unable to be searched.

## Full search terms and strategies: 2021 search update

This provides full details of all search strings used for the 2021 update to searches on bibliographic databases and trials registers. It includes dates and number of references returned and notes explaining any unusual search techniques or syntax. The EndNote X9 import order is provided, as the deduplication technique keeps the first uploaded copy of the reference by default. After deduplication, papers retrieved by the 2020 searches were removed so only items retrieved in the update were screened.

All searches were run across the entire database unless otherwise noted. In all searches, numbers in parentheses at the end of each row show the number of hits retrieved.

### OvidSP Medline

| Database name | Medline |
| --- | --- |
| Database platform | OvidSP |
| Dates of database coverage | Ovid MEDLINE ALL 1946 to May 10, 2021 |
| Date searched | 11 May 2021 |
| Searched by | JF |
| Number of results | 9589 |
| EndNote import order | 1 |
| Number of results once 2020 results and duplicates removed | 970 |
| Search strategy notes | This is the search that all other database searches are based on.  Search lines ending in a ‘/’ are subject heading searches. Search lines ending in ‘/*ab*’ are subject headings with subheadings added. The subheading is listed in square brackets at the end of the line. Search lines beginning ‘exp’ are exploded subject heading searches. Search lines ending in .ti,ab,kf. search in the title, abstract and author keywords only. Search lines ending in .jw. search in the journal title only. or/*x-y* combines search sets in the range *x-y* with Boolean operator OR. * is used for truncation of words. # is used for a compulsory wildcard. ? is used for an optional wildcard. adj*n* searches for terms within *n* words or each other. Words in square brackets [ ] are comments and are not included in the search terms. |

1	child/ (1736286)

2	adolescent/ (2086993)

3	Minors/ (2638)

4	Students/ (63090)

5	School Teachers/ (1771)

6	exp Parents/ (118824)

7	child*.ti,ab,kf. (1495774)

8	boy?.ti,ab,kf. (151099)

9	girl?.ti,ab,kf. (154625)

10	(schoolage or (school adj1 age)).ti,ab,kf. (14583)

11	schoolchild*.ti,ab,kf. (14521)

12	youngster?.ti,ab,kf. (2583)

13	minor?.ti,ab,kf. (236495)

14	(pupil* or student*).ti,ab,kf. not (pupil/ or exp Students, Health Occupations/) (267337)

15	prepubescen*.ti,ab,kf. (1057)

16	pubescent*.ti,ab,kf. (926)

17	adolescent*.ti,ab,kf. (276284)

18	juvenil*.ti,ab,kf. (86763)

19	underage*.ti,ab,kf. (1348)

20	(preteen* or pre-teen*).ti,ab,kf. (512)

21	(teen or teens).ti,ab,kf. (11267)

22	teenage*.ti,ab,kf. (22323)

23	(youth or youths).ti,ab,kf. (84963)

24	young person*.ti,ab,kf. (3648)

25	young people*.ti,ab,kf. (30752)

26	(kid or kids).ti,ab,kf. (8504)

27	(transition adj4 adult*).ti,ab,kf. (4919)

28	emerging adult*.ti,ab,kf. (3006)

29	young adult*.ti,ab,kf. (102565)

30	parent?.ti,ab,kf. (286051)

31	mother?.ti,ab,kf. (227656)

32	father?.ti,ab,kf. (42714)

33	guardian?.ti,ab,kf. (7600)

34	teacher?.ti,ab,kf. (47154)

35	child*.jw. (163869)

36	adolescen*.jw. (48191)

37	youth*.jw. (3869)

38	school*.jw. (18264)

39	(("5" or "6" or "7" or "8" or "9" or "10" or "11" or "12" or "13" or "14" or "15" or "16" or "17" or "18") adj (year* old or year* of age)).ti,ab,kf. (236277)

40	((five or six or seven or eight or nine or ten or eleven or twelve or thirteen or fourteen or fifteen or sixteen or seventeen or eighteen) adj (year* old or year* of age)).ti,ab,kf. (19787)

41	(age* adj ("5" or "6" or "7" or "8" or "9" or "10" or "11" or "12" or "13" or "14" or "15" or "16" or "17" or "18") adj year*).ti,ab,kf. (49917)

42	(age* adj (five or six or seven or eight or nine or ten or eleven or twelve or thirteen or fourteen or fifteen or sixteen or seventeen or eighteen) adj year*).ti,ab,kf. (988)

43	((primary or secondary or year) adj1 ("1" or "2" or "3" or "4" or "5" or "6" or "7" or "8" or "9" or "10" or "11" or "12" or "13")).ti,ab,kf. (654147)

44	(grade? adj1 (first or second or third or fourth or fifth or sixth or seventh or eighth or ninth or tenth or eleventh or twelfth)).ti,ab,kf. (11420)

45	or/1-44 [POPULATION] (4791374)

46	schools/og [Organization & Administration] (1757)

47	School Health Services/og [Organization & Administration] (3901)

48	(school? adj5 (based or level or wide or led or ethos or environment or organi?ation* or climate or toolkit? or approach* or action group? or practice? or whole or health* or leader* or cultur* or manag* or governance or system? or communit*)).ti,ab,kf. (64384)

49	schoolwide.ti,ab,kf. (99)

50	restor* justice.ti,ab,kf. (164)

51	Schools/ (40860)

52	school?.ti,ab,kf,jw. (295921)

53	or/51-52 (301031)

54	"peer-to-peer".ti,ab. (1549)

55	(peer adj2 (led or leader* or participat* or mediat* or helper? or resistan* or wide or action? or influen*)).ti,ab,kf. (4395)

56	(relationship* adj2 (led or leader* or participat* or mediat* or helper? or resistan* or wide or action? or influen*)).ti,ab,kf. (17038)

57	Community Networks/ (7056)

58	Community Participation/ (17617)

59	((community or communities) adj2 (led or leader* or participat* or mediat* or helper? or resistan* or wide or action? or influen* or relationship*)).ti,ab,kf. (23642)

60	(pupil? adj2 (led or leader* or participat* or mediat* or helper? or resistan* or wide or action? or influen* or relationship*)).ti,ab,kf. not pupil/ [removes items about eyes] (296)

61	((child or children) adj2 (led or leader* or participat* or mediat* or helper? or resistan* or wide or action? or influen*)).ti,ab,kf. (14169)

62	((young people or young person) adj2 (led or leader* or participat* or mediat* or helper? or resistan* or wide or action? or influen*)).ti,ab,kf. (322)

63	(teen* adj2 (led or leader* or participat* or mediat* or helper? or resistan* or wide or action? or influen*)).ti,ab,kf. (397)

64	(adolescent* adj2 (led or leader* or participat* or mediat* or helper? or resistan* or wide or action? or influen*)).ti,ab,kf. (4995)

65	(youth? adj2 (led or leader* or participat* or mediat* or helper? or resistan* or wide or action? or influen*)).ti,ab,kf. (2125)

66	(student? adj2 (led or leader* or participat* or mediat* or helper? or resistan* or wide or action? or influen* or relationship*)).ti,ab,kf. (15287)

67	(teach* adj2 (led or leader* or participat* or mediat* or helper? or resistan* or wide or action? or influen* or relationship*)).ti,ab,kf. (3646)

68	(curricul* adj2 (led or leader* or participat* or mediat* or helper? or resistan* or wide or action? or influen* or relationship*)).ti,ab,kf. (828)

69	(administrat* adj2 (led or leader* or participat* or mediat* or helper? or resistan* or wide or action? or influen* or relationship*)).ti,ab,kf. (5270)

70	((personnel or staff) adj2 (led or leader* or participat* or mediat* or helper? or resistan* or wide or action? or influen* or relationship*)).ti,ab,kf. (4668)

71	(volunteer* adj2 (led or leader* or participat* or mediat* or helper? or resistan* or wide or action? or influen* or relationship*)).ti,ab,kf. (6115)

72	((advocacy or advocate) adj2 (led or leader* or participat* or mediat* or helper? or resistan* or wide or action? or influen* or relationship*)).ti,ab,kf. (811)

73	(parent* adj2 (led or leader* or participat* or mediat* or helper? or resistan* or wide or action? or influen*)).ti,ab,kf. (10580)

74	Social Environment/ (43396)

75	(environment* adj2 (led or leader* or participat* or mediat* or helper? or resistan* or wide or action? or influen* or relationship*)).ti,ab,kf. (30354)

76	(socio-ecolog* adj2 (led or leader* or participat* or mediat* or helper? or resistan* or wide or action? or influen* or relationship*)).ti,ab,kf. (48)

77	social support/ (73169)

78	social learning/ (507)

79	(learn* adj2 (led or leader* or participat* or mediat* or helper? or resistan* or wide or action? or influen* or relationship*)).ti,ab,kf. (7374)

80	((policy or policies) adj2 (led or leader* or participat* or mediat* or helper? or resistan* or wide or action? or influen* or relationship*)).ti,ab,kf. (6392)

81	or/54-80 (276743)

82	53 and 81 (26271)

83	or/46-50,82 [SCHOOL-WIDE INTERVENTION] (83769)

84	Smoking Prevention/ (18194)

85	exp Tobacco Smoking/pc [Prevention & Control] (208)

86	Vaping/pc [Prevention & Control] (69)

87	Marijuana Smoking/pc [Prevention & Control] (273)

88	smoking cessation/ (29755)

89	"tobacco use cessation"/ (1245)

90	smoking reduction/ (84)

91	((smoking or smoked or smoke or smoker) adj5 (reduc* or decreas* or increas* or lower* or fewer or improv* or enhance* or extend* or develop* or prevalen* or daily or weekly or current or rate or rates or behavio?r* or knowledge or prevent* or attitude* or avoid* or perceive* or percept* or promot* or program* or change*)).ti,ab,kf. (84736)

92	(tobacco adj5 (reduc* or decreas* or increas* or lower* or fewer or improv* or enhance* or extend* or develop* or prevalen* or daily or weekly or current or rate or rates or behavio?r* or knowledge or prevent* or attitude* or avoid* or perceive* or percept* or promot* or program* or change*)).ti,ab,kf. (27740)

93	(cigarette? adj5 (reduc* or decreas* or increas* or lower* or fewer or improv* or enhance* or extend* or develop* or prevalen* or daily or weekly or current or rate or rates or behavio?r* or knowledge or prevent* or attitude* or avoid* or perceive* or percept* or promot* or program* or change*)).ti,ab,kf. (22895)

94	(marijuana adj5 (reduc* or decreas* or increas* or lower* or fewer or improv* or enhance* or extend* or develop* or prevalen* or daily or weekly or current or rate or rates or behavio?r* or knowledge or prevent* or attitude* or avoid* or perceive* or percept* or promot* or program* or change*)).ti,ab,kf. (3903)

95	(cannabis adj5 (reduc* or decreas* or increas* or lower* or fewer or improv* or enhance* or extend* or develop* or prevalen* or daily or weekly or current or rate or rates or behavio?r* or knowledge or prevent* or attitude* or avoid* or perceive* or percept* or promot* or program* or change*)).ti,ab,kf. (5724)

96	(solvent? adj5 (reduc* or decreas* or increas* or lower* or fewer or improv* or enhance* or extend* or develop* or prevalen* or daily or weekly or current or rate or rates or behavio?r* or knowledge or prevent* or attitude* or avoid* or perceive* or percept* or promot* or program* or change*)).ti,ab,kf. (20655)

97	exp Substance-Related Disorders/pc [Prevention & Control] (23285)

98	Alcohol Drinking/pc [Prevention & Control] (4540)

99	Binge Drinking/pc [Prevention & Control] (210)

100	Underage Drinking/pc [Prevention & Control] (176)

101	(alcohol adj5 (reduc* or decreas* or increas* or lower* or fewer or improv* or enhance* or extend* or develop* or prevalen* or daily or weekly or current or rate or rates or behavio?r* or knowledge or prevent* or attitude* or avoid* or perceive* or percept* or promot* or program* or change*)).ti,ab,kf. (67904)

102	(binge drink* adj5 (reduc* or decreas* or increas* or lower* or fewer or improv* or enhance* or extend* or develop* or prevalen* or daily or weekly or current or rate or rates or behavio?r* or knowledge or prevent* or attitude* or avoid* or perceive* or percept* or promot* or program* or change*)).ti,ab,kf. (2158)

103	(underage drink* adj5 (reduc* or decreas* or increas* or lower* or fewer or improv* or enhance* or extend* or develop* or prevalen* or daily or weekly or current or rate or rates or behavio?r* or knowledge or prevent* or attitude* or avoid* or perceive* or percept* or promot* or program* or change*)).ti,ab,kf. (235)

104	(substance? adj5 (reduc* or decreas* or increas* or lower* or fewer or improv* or enhance* or extend* or develop* or prevalen* or daily or weekly or current or rate or rates or behavio?r* or knowledge or prevent* or attitude* or avoid* or perceive* or percept* or promot* or program* or change*)).ti,ab,kf. (49440)

105	(drug? adj5 (reduc* or decreas* or increas* or lower* or fewer or improv* or enhance* or extend* or develop* or prevalen* or daily or weekly or current or rate or rates or behavio?r* or knowledge or prevent* or attitude* or avoid* or perceive* or percept* or promot* or program* or change*)).ti,ab,kf. (377921)

106	exp Academic Performance/ (2650)

107	(education* attain* adj5 (reduc* or decreas* or increas* or lower* or fewer or improv* or enhance* or extend* or develop* or prevalen* or daily or weekly or current or rate or rates or behavio?r* or knowledge or prevent* or attitude* or avoid* or perceive* or percept* or promot* or program* or change*)).ti,ab,kf. (2740)

108	(academic attain* adj5 (reduc* or decreas* or increas* or lower* or fewer or improv* or enhance* or extend* or develop* or prevalen* or daily or weekly or current or rate or rates or behavio?r* or knowledge or prevent* or attitude* or avoid* or perceive* or percept* or promot* or program* or change*)).ti,ab,kf. (73)

109	(student* attain* adj5 (reduc* or decreas* or increas* or lower* or fewer or improv* or enhance* or extend* or develop* or prevalen* or daily or weekly or current or rate or rates or behavio?r* or knowledge or prevent* or attitude* or avoid* or perceive* or percept* or promot* or program* or change*)).ti,ab,kf. (23)

110	(education* achieve* adj5 (reduc* or decreas* or increas* or lower* or fewer or improv* or enhance* or extend* or develop* or prevalen* or daily or weekly or current or rate or rates or behavio?r* or knowledge or prevent* or attitude* or avoid* or perceive* or percept* or promot* or program* or change*)).ti,ab,kf. (417)

111	(academic achieve* adj5 (reduc* or decreas* or increas* or lower* or fewer or improv* or enhance* or extend* or develop* or prevalen* or daily or weekly or current or rate or rates or behavio?r* or knowledge or prevent* or attitude* or avoid* or perceive* or percept* or promot* or program* or change*)).ti,ab,kf. (1640)

112	(student* achieve* adj5 (reduc* or decreas* or increas* or lower* or fewer or improv* or enhance* or extend* or develop* or prevalen* or daily or weekly or current or rate or rates or behavio?r* or knowledge or prevent* or attitude* or avoid* or perceive* or percept* or promot* or program* or change*)).ti,ab,kf. (248)

113	(education* perform* adj5 (reduc* or decreas* or increas* or lower* or fewer or improv* or enhance* or extend* or develop* or prevalen* or daily or weekly or current or rate or rates or behavio?r* or knowledge or prevent* or attitude* or avoid* or perceive* or percept* or promot* or program* or change*)).ti,ab,kf. (113)

114	(academic perform* adj5 (reduc* or decreas* or increas* or lower* or fewer or improv* or enhance* or extend* or develop* or prevalen* or daily or weekly or current or rate or rates or behavio?r* or knowledge or prevent* or attitude* or avoid* or perceive* or percept* or promot* or program* or change*)).ti,ab,kf. (2082)

115	(student* perform* adj5 (reduc* or decreas* or increas* or lower* or fewer or improv* or enhance* or extend* or develop* or prevalen* or daily or weekly or current or rate or rates or behavio?r* or knowledge or prevent* or attitude* or avoid* or perceive* or percept* or promot* or program* or change*)).ti,ab,kf. (1070)

116	(education* outcome? adj5 (reduc* or decreas* or increas* or lower* or fewer or improv* or enhance* or extend* or develop* or prevalen* or daily or weekly or current or rate or rates or behavio?r* or knowledge or prevent* or attitude* or avoid* or perceive* or percept* or promot* or program* or change*)).ti,ab,kf. (692)

117	(academic outcome? adj5 (reduc* or decreas* or increas* or lower* or fewer or improv* or enhance* or extend* or develop* or prevalen* or daily or weekly or current or rate or rates or behavio?r* or knowledge or prevent* or attitude* or avoid* or perceive* or percept* or promot* or program* or change*)).ti,ab,kf. (352)

118	(student* outcome? adj5 (reduc* or decreas* or increas* or lower* or fewer or improv* or enhance* or extend* or develop* or prevalen* or daily or weekly or current or rate or rates or behavio?r* or knowledge or prevent* or attitude* or avoid* or perceive* or percept* or promot* or program* or change*)).ti,ab,kf. (244)

119	(education* commitment adj5 (reduc* or decreas* or increas* or lower* or fewer or improv* or enhance* or extend* or develop* or prevalen* or daily or weekly or current or rate or rates or behavio?r* or knowledge or prevent* or attitude* or avoid* or perceive* or percept* or promot* or program* or change*)).ti,ab,kf. (8)

120	(academic commitment adj5 (reduc* or decreas* or increas* or lower* or fewer or improv* or enhance* or extend* or develop* or prevalen* or daily or weekly or current or rate or rates or behavio?r* or knowledge or prevent* or attitude* or avoid* or perceive* or percept* or promot* or program* or change*)).ti,ab,kf. (6)

121	(student* commitment adj5 (reduc* or decreas* or increas* or lower* or fewer or improv* or enhance* or extend* or develop* or prevalen* or daily or weekly or current or rate or rates or behavio?r* or knowledge or prevent* or attitude* or avoid* or perceive* or percept* or promot* or program* or change*)).ti,ab,kf. (18)

122	(education* engage* adj5 (reduc* or decreas* or increas* or lower* or fewer or improv* or enhance* or extend* or develop* or prevalen* or daily or weekly or current or rate or rates or behavio?r* or knowledge or prevent* or attitude* or avoid* or perceive* or percept* or promot* or program* or change*)).ti,ab,kf. (18)

123	(academic engage* adj5 (reduc* or decreas* or increas* or lower* or fewer or improv* or enhance* or extend* or develop* or prevalen* or daily or weekly or current or rate or rates or behavio?r* or knowledge or prevent* or attitude* or avoid* or perceive* or percept* or promot* or program* or change*)).ti,ab,kf. (102)

124	(student* engage* adj5 (reduc* or decreas* or increas* or lower* or fewer or improv* or enhance* or extend* or develop* or prevalen* or daily or weekly or current or rate or rates or behavio?r* or knowledge or prevent* or attitude* or avoid* or perceive* or percept* or promot* or program* or change*)).ti,ab,kf. (666)

125	(dropout adj5 (reduc* or decreas* or increas* or lower* or fewer or improv* or enhance* or extend* or develop* or prevalen* or daily or weekly or current or rate or rates or behavio?r* or knowledge or prevent* or attitude* or avoid* or perceive* or percept* or promot* or program* or change*)).ti,ab,kf. (4853)

126	(absentee* adj5 (reduc* or decreas* or increas* or lower* or fewer or improv* or enhance* or extend* or develop* or prevalen* or daily or weekly or current or rate or rates or behavio?r* or knowledge or prevent* or attitude* or avoid* or perceive* or percept* or promot* or program* or change*)).ti,ab,kf. (2258)

127	Violence/pc [Prevention & Control] (6114)

128	Aggression/pc [Prevention & Control] (16)

129	Physical Abuse/pc [Prevention & Control] (75)

130	Gender-Based Violence/pc [Prevention & Control] (78)

131	Gun Violence/pc [Prevention & Control] (107)

132	exp Bullying/pc [Prevention & Control] (452)

133	((violent or violence) adj5 (reduc* or decreas* or increas* or lower* or fewer or improv* or enhance* or extend* or develop* or prevalen* or daily or weekly or current or rate or rates or behavio?r* or knowledge or prevent* or attitude* or avoid* or perceive* or percept* or promot* or program* or change*)).ti,ab,kf. (21996)

134	((aggression or aggressive) adj5 (reduc* or decreas* or increas* or lower* or fewer or improv* or enhance* or extend* or develop* or prevalen* or daily or weekly or current or rate or rates or behavio?r* or knowledge or prevent* or attitude* or avoid* or perceive* or percept* or promot* or program* or change*)).ti,ab,kf. (48245)

135	((hostile or hostility) adj5 (reduc* or decreas* or increas* or lower* or fewer or improv* or enhance* or extend* or develop* or prevalen* or daily or weekly or current or rate or rates or behavio?r* or knowledge or prevent* or attitude* or avoid* or perceive* or percept* or promot* or program* or change*)).ti,ab,kf. (2939)

136	(assault* adj5 (reduc* or decreas* or increas* or lower* or fewer or improv* or enhance* or extend* or develop* or prevalen* or daily or weekly or current or rate or rates or behavio?r* or knowledge or prevent* or attitude* or avoid* or perceive* or percept* or promot* or program* or change*)).ti,ab,kf. (2939)

137	(crime adj5 (reduc* or decreas* or increas* or lower* or fewer or improv* or enhance* or extend* or develop* or prevalen* or daily or weekly or current or rate or rates or behavio?r* or knowledge or prevent* or attitude* or avoid* or perceive* or percept* or promot* or program* or change*)).ti,ab,kf. (3489)

138	((abuse? or abusive) adj5 (reduc* or decreas* or increas* or lower* or fewer or improv* or enhance* or extend* or develop* or prevalen* or daily or weekly or current or rate or rates or behavio?r* or knowledge or prevent* or attitude* or avoid* or perceive* or percept* or promot* or program* or change*)).ti,ab,kf. (29388)

139	(misbehav* adj5 (reduc* or decreas* or increas* or lower* or fewer or improv* or enhance* or extend* or develop* or prevalen* or daily or weekly or current or rate or rates or behavio?r* or knowledge or prevent* or attitude* or avoid* or perceive* or percept* or promot* or program* or change*)).ti,ab,kf. (147)

140	(threat? adj5 (reduc* or decreas* or increas* or lower* or fewer or improv* or enhance* or extend* or develop* or prevalen* or daily or weekly or current or rate or rates or behavio?r* or knowledge or prevent* or attitude* or avoid* or perceive* or percept* or promot* or program* or change*)).ti,ab,kf. (15337)

141	(conduct adj5 (reduc* or decreas* or increas* or lower* or fewer or improv* or enhance* or extend* or develop* or prevalen* or daily or weekly or current or rate or rates or behavio?r* or knowledge or prevent* or attitude* or avoid* or perceive* or percept* or promot* or program* or change*)).ti,ab,kf. (9187)

142	(delinquen* adj5 (reduc* or decreas* or increas* or lower* or fewer or improv* or enhance* or extend* or develop* or prevalen* or daily or weekly or current or rate or rates or behavio?r* or knowledge or prevent* or attitude* or avoid* or perceive* or percept* or promot* or program* or change*)).ti,ab,kf. (3020)

143	(disrupt* behavio?r* adj5 (reduc* or decreas* or increas* or lower* or fewer or improv* or enhance* or extend* or develop* or prevalen* or daily or weekly or current or rate or rates or behavio?r* or knowledge or prevent* or attitude* or avoid* or perceive* or percept* or promot* or program* or change*)).ti,ab,kf. (3990)

144	(problem* behavio?r* adj5 (reduc* or decreas* or increas* or lower* or fewer or improv* or enhance* or extend* or develop* or prevalen* or daily or weekly or current or rate or rates or behavio?r* or knowledge or prevent* or attitude* or avoid* or perceive* or percept* or promot* or program* or change*)).ti,ab,kf. (6201)

145	(volatile behavio?r* adj5 (reduc* or decreas* or increas* or lower* or fewer or improv* or enhance* or extend* or develop* or prevalen* or daily or weekly or current or rate or rates or behavio?r* or knowledge or prevent* or attitude* or avoid* or perceive* or percept* or promot* or program* or change*)).ti,ab,kf. (28)

146	((antisocial or anti-social) adj5 (reduc* or decreas* or increas* or lower* or fewer or improv* or enhance* or extend* or develop* or prevalen* or daily or weekly or current or rate or rates or behavio?r* or knowledge or prevent* or attitude* or avoid* or perceive* or percept* or promot* or program* or change*)).ti,ab,kf. (5529)

147	(perpetrat* adj5 (reduc* or decreas* or increas* or lower* or fewer or improv* or enhance* or extend* or develop* or prevalen* or daily or weekly or current or rate or rates or behavio?r* or knowledge or prevent* or attitude* or avoid* or perceive* or percept* or promot* or program* or change*)).ti,ab,kf. (2125)

148	(bully* adj5 (reduc* or decreas* or increas* or lower* or fewer or improv* or enhance* or extend* or develop* or prevalen* or daily or weekly or current or rate or rates or behavio?r* or knowledge or prevent* or attitude* or avoid* or perceive* or percept* or promot* or program* or change*)).ti,ab,kf. (2892)

149	(victimi* adj5 (reduc* or decreas* or increas* or lower* or fewer or improv* or enhance* or extend* or develop* or prevalen* or daily or weekly or current or rate or rates or behavio?r* or knowledge or prevent* or attitude* or avoid* or perceive* or percept* or promot* or program* or change*)).ti,ab,kf. (3794)

150	(cyberbully* adj5 (reduc* or decreas* or increas* or lower* or fewer or improv* or enhance* or extend* or develop* or prevalen* or daily or weekly or current or rate or rates or behavio?r* or knowledge or prevent* or attitude* or avoid* or perceive* or percept* or promot* or program* or change*)).ti,ab,kf. (415)

151	(conflict* adj5 (reduc* or decreas* or increas* or lower* or fewer or improv* or enhance* or extend* or develop* or prevalen* or daily or weekly or current or rate or rates or behavio?r* or knowledge or prevent* or attitude* or avoid* or perceive* or percept* or promot* or program* or change*)).ti,ab,kf. (18529)

152	(positive behavio?r* adj5 (reduc* or decreas* or increas* or lower* or fewer or improv* or enhance* or extend* or develop* or prevalen* or daily or weekly or current or rate or rates or behavio?r* or knowledge or prevent* or attitude* or avoid* or perceive* or percept* or promot* or program* or change*)).ti,ab,kf. (1760)

153	(improv* behavio?r* adj5 (reduc* or decreas* or increas* or lower* or fewer or improv* or enhance* or extend* or develop* or prevalen* or daily or weekly or current or rate or rates or behavio?r* or knowledge or prevent* or attitude* or avoid* or perceive* or percept* or promot* or program* or change*)).ti,ab,kf. (2482)

154	(social behavio?r* adj5 (reduc* or decreas* or increas* or lower* or fewer or improv* or enhance* or extend* or develop* or prevalen* or daily or weekly or current or rate or rates or behavio?r* or knowledge or prevent* or attitude* or avoid* or perceive* or percept* or promot* or program* or change*)).ti,ab,kf. (16759)

155	((social-emotion* or socio-emotion*) adj5 (reduc* or decreas* or increas* or lower* or fewer or improv* or enhance* or extend* or develop* or prevalen* or daily or weekly or current or rate or rates or behavio?r* or knowledge or prevent* or attitude* or avoid* or perceive* or percept* or promot* or program* or change*)).ti,ab,kf. (2306)

156	(social-charact* adj5 (reduc* or decreas* or increas* or lower* or fewer or improv* or enhance* or extend* or develop* or prevalen* or daily or weekly or current or rate or rates or behavio?r* or knowledge or prevent* or attitude* or avoid* or perceive* or percept* or promot* or program* or change*)).ti,ab,kf. (256)

157	(social-inclusion adj5 (reduc* or decreas* or increas* or lower* or fewer or improv* or enhance* or extend* or develop* or prevalen* or daily or weekly or current or rate or rates or behavio?r* or knowledge or prevent* or attitude* or avoid* or perceive* or percept* or promot* or program* or change*)).ti,ab,kf. (461)

158	(social develop* adj5 (reduc* or decreas* or increas* or lower* or fewer or improv* or enhance* or extend* or develop* or prevalen* or daily or weekly or current or rate or rates or behavio?r* or knowledge or prevent* or attitude* or avoid* or perceive* or percept* or promot* or program* or change*)).ti,ab,kf. (4706)

159	(social competen* adj5 (reduc* or decreas* or increas* or lower* or fewer or improv* or enhance* or extend* or develop* or prevalen* or daily or weekly or current or rate or rates or behavio?r* or knowledge or prevent* or attitude* or avoid* or perceive* or percept* or promot* or program* or change*)).ti,ab,kf. (1287)

160	(emotion* develop* adj5 (reduc* or decreas* or increas* or lower* or fewer or improv* or enhance* or extend* or develop* or prevalen* or daily or weekly or current or rate or rates or behavio?r* or knowledge or prevent* or attitude* or avoid* or perceive* or percept* or promot* or program* or change*)).ti,ab,kf. (1924)

161	(conduct adj5 (reduc* or decreas* or increas* or lower* or fewer or improv* or enhance* or extend* or develop* or prevalen* or daily or weekly or current or rate or rates or behavio?r* or knowledge or prevent* or attitude* or avoid* or perceive* or percept* or promot* or program* or change*)).ti,ab,kf. (9187)

162	(respect adj5 (reduc* or decreas* or increas* or lower* or fewer or improv* or enhance* or extend* or develop* or prevalen* or daily or weekly or current or rate or rates or behavio?r* or knowledge or prevent* or attitude* or avoid* or perceive* or percept* or promot* or program* or change*)).ti,ab,kf. (40924)

163	((safety or safe) adj5 (reduc* or decreas* or increas* or lower* or fewer or improv* or enhance* or extend* or develop* or prevalen* or daily or weekly or current or rate or rates or behavio?r* or knowledge or prevent* or attitude* or avoid* or perceive* or percept* or promot* or program* or change*)).ti,ab,kf. (135430)

164	or/84-163 [BEHAVIOUR CHANGE] (979922)

165	exp Program Evaluation/ (78407)

166	Evaluation Studies/ (257784)

167	Validation Studies/ (105247)

168	exp Clinical Trial/ (891265)

169	prevent*.ti,ab,kf. (1529271)

170	program*.ti,ab,kf. (942048)

171	intervent*.ti,ab,kf. (1099352)

172	restorative approach*.ti,ab,kf. (238)

173	control school?.ti,ab,kf. (918)

174	(trial or trials).ti,ab,kf. (1112934)

175	or/165-174 [QUANTITATIVE STUDY] (4719966)

176	45 and 83 and 164 and 175 (9589)

## EBSCO CINAHL Plus

| Database name | CINAHL Plus |
| --- | --- |
| Database platform | EBSCO |
| Dates of database coverage | Complete database to date searched. |
| Date searched | 11/05/2021 |
| Searched by | JF |
| Number of results | 7075 |
| EndNote import order | 8 |
| Number of results once duplicates removed | 745 |
| Search strategy notes | * is used for truncation. # is used for an optional wildcard. Search strings starting with TI search the title only. Search strings starting with AB search the abstract only. Search strings starting with SO search the journal title only. N/*n* search for terms within *n* words of each other, in any order. |

| Search number | Query | Results |
| --- | --- | --- |
| S1 | (MH "Child") | 477,984 |
| S2 | (MH "Adolescence") | 549,061 |
| S3 | (MH "Minors (Legal)") | 735 |
| S4 | (MH "Students, High School") OR (MH "Students, Middle School") OR (MH "Students") | 30,760 |
| S5 | (MH "Teachers") | 12,437 |
| S6 | (MH "Parents+") | 101,104 |
| S7 | (TI child*) or (AB child*) | 514,064 |
| S8 | (TI boy#) or (AB boy#) | 40,230 |
| S9 | (TI girl#) or (AB girl#) | 44,440 |
| S10 | (TI (schoolage or (school N1 age))) or (AB (schoolage or (school N1 age))) | 6,759 |
| S11 | (TI schoolchild*) or (AB schoolchild*) | 4,022 |
| S12 | (TI youngster#) OR (AB youngster#) | 901 |
| S13 | (TI minor#) OR (AB minor#) | 32,183 |
| S14 | ((TI pupil*) OR (AB pupil*)) NOT (MH "Pupil") | 4,949 |
| S15 | ((TI student*) OR (AB student*)) NOT (MH "Students, Health Occupations+") | 130,439 |
| S16 | (TI prepubescen*) OR (AB prepubescen*) | 286 |
| S17 | (TI pubescent*) or (AB pubescent*) | 180 |
| S18 | (TI adolescent*) OR (AB adolescent*) | 132,398 |
| S19 | (TI juvenil*) OR (AB juvenil*) | 10,664 |
| S20 | (TI underage*) or (AB underage*) | 891 |
| S21 | (TI (preteen* or pre-teen*)) OR (AB (preteen* or pre-teen*)) | 325 |
| S22 | (TI (teen or teens)) OR (AB (teen or teens)) | 9,423 |
| S23 | (TI teenage*) OR (AB teenage*) | 11,059 |
| S24 | (TI (youth or youths)) OR (AB (youth or youths)) | 52,237 |
| S25 | (TI "young person*") OR (AB "young person*") | 1,661 |
| S26 | (TI "young people*") OR (AB "young people*") | 21,205 |
| S27 | (TI (kid or kids)) OR (AB (kid or kids)) | 10,453 |
| S28 | (TI (transition n4 adult*)) OR (AB (transition N4 adult*)) | 3,367 |
| S29 | (TI "emerging adult*") OR (AB "emerging adult*") | 2,176 |
| S30 | (TI "young adult*") OR (AB "young adult*") | 37,180 |
| S31 | (TI parent#) OR (AB parent#) | 109,434 |
| S32 | (TI mother#) OR (AB mother#) | 81,402 |
| S33 | (TI father#) or (AB father#) | 15,901 |
| S34 | (TI guardian#) OR (AB guardian#) | 3,029 |
| S35 | (TI teacher#) OR (AB teacher#) | 28,982 |
| S36 | (SO child*) | 122,943 |
| S37 | (SO adolescen*) | 43,451 |
| S38 | (SO youth*) | 9,175 |
| S39 | (SO school*) | 19,061 |
| S40 | (TI ("5" or "6" or "7" or "8" or "9" or "10" or "11" or "12" or "13" or "14" or "15" or "16" or "17" or "18") N1 ("year* old" or "year* of age")) OR (AB ("5" or "6" or "7" or "8" or "9" or "10" or "11" or "12" or "13" or "14" or "15" or "16" or "17" or "18") N1 ("year* old" or "year* of age")) | 29,738 |
| S41 | (TI (five or six or seven or eight or nine or ten or eleven or twelve or thirteen or fourteen or fifteen or sixteen or seventeen or eighteen) N1 ("year* old" or "year* of age")) OR (AB (five or six or seven or eight or nine or ten or eleven or twelve or thirteen or fourteen or fifteen or sixteen or seventeen or eighteen) N1 ("year* old" or "year* of age")) | 33,215 |
| S42 | (TI (age* N1 ("5" or "6" or "7" or "8" or "9" or "10" or "11" or "12" or "13" or "14" or "15" or "16" or "17" or "18") N1 year*)) OR (AB (age* N1 ("5" or "6" or "7" or "8" or "9" or "10" or "11" or "12" or "13" or "14" or "15" or "16" or "17" or "18") N1 year*)) | 36,617 |
| S43 | (TI (primary or secondary or year) N1 ("1" or "2" or "3" or "4" or "5" or "6" or "7" or "8" or "9" or "10" or "11" or "12" or "13")) OR (AB (primary or secondary or year) N1 ("1" or "2" or "3" or "4" or "5" or "6" or "7" or "8" or "9" or "10" or "11" or "12" or "13")) | 223,515 |
| S44 | (TI grade# N1 (first or second or third or fourth or fifth or sixth or seventh or eighth or ninth or tenth or eleventh or twelfth)) OR (AB grade# N1 (first or second or third or fourth or fifth or sixth or seventh or eighth or ninth or tenth or eleventh or twelfth)) | 8,336 |
| S45 | S1 OR S2 OR S3 OR S4 OR S5 OR S6 OR S7 OR S8 OR S9 OR S10 OR S11 OR S12 OR S13 OR S14 OR S15 OR S16 OR S17 OR S18 OR S19 OR S20 OR S21 OR S22 OR S23 OR S24 OR S25 OR S26 OR S27 OR S28 OR S29 OR S30 OR S31 OR S32 OR S33 OR S34 OR S35 OR S36 OR S37 OR S38 OR S39 OR S40 OR S41 OR S42 OR S43 OR S44 | 1,476,468 |
| S46 | (MH "Schools, Secondary/AM/OG") OR (MH "Schools, Middle/AM/OG") OR (MH "Schools, Elementary/AM/OG") OR (MH "Schools/AM/OG") | 536 |
| S47 | (MH "School Health Services+/AM/OG") | 1,015 |
| S48 | (TI (school# N5 (based or level or wide or led or ethos or environment or organi#ation* or climate or toolkit# or approach* or "action group#" or practice# or whole or health* or leader* or cultur* or manag* or governance or system# or communit*))) OR (AB (school# N5 (based or level or wide or led or ethos or environment or organi#ation* or climate or toolkit# or approach* or "action group#" or practice# or whole or health* or leader* or cultur* or manag* or governance or system# or communit*))) | 44,118 |
| S49 | (TI schoolwide) or (AB schoolwide) | 143 |
| S50 | (TI "restor* justice") OR (AB "restor* justice") | 190 |
| S51 | (MH "Schools") OR (MH "Schools, Elementary") OR (MH "Schools, Middle") OR (MH "Schools, Secondary") | 24,861 |
| S52 | (TI school#) or (AB school#) | 144,545 |
| S53 | S51 OR S52 | 150,092 |
| S54 | (TI "peer-to-peer") OR (AB "peer-to-peer") | 983 |
| S55 | (TI (peer N2 (led or leader* or participat* or mediat* or helper# or resistan* or wide or action# or influen*))) OR (AB (peer N2 (led or leader* or participat* or mediat* or helper# or resistan* or wide or action# or influen*))) | 3,847 |
| S56 | (TI (relationship* N2 (led or leader* or participat* or mediat* or helper# or resistan* or wide or action# or influen*))) OR (AB (relationship* N2 (led or leader* or participat* or mediat* or helper# or resistan* or wide or action# or influen*))) | 12,810 |
| S57 | (MH "Community Networks") | 2,565 |
| S58 | (TI ((community or communities) N2 (led or leader* or participat* or mediat* or helper# or resistan* or wide or action# or influen* or relationship*))) OR (AB ((community or communities) N2 (led or leader* or participat* or mediat* or helper# or resistan* or wide or action# or influen* or relationship*))) | 15,151 |
| S59 | ((TI (pupil# N2 (led or leader* or participat* or mediat* or helper# or resistan* or wide or action# or influen* or relationship*)) OR (AB (pupil# N2 (led or leader* or participat* or mediat* or helper# or resistan* or wide or action# or influen* or relationship*))) NOT (MH "Pupil") | 167 |
| S60 | (TI ((child or children) N2 (led or leader* or participat* or mediat* or helper# or resistan* or wide or action# or influen*))) OR (AB (child or children*) N2 (led or leader* or participat* or mediat* or helper# or resistan* or wide or action# or influen*))) | 13,236 |
| S61 | (TI (("young people" or "young person") N2 (led or leader* or participat* or mediat* or helper# or resistan* or wide or action# or influen*))) OR (AB (("young people" or "young person") N2 (led or leader* or participat* or mediat* or helper# or resistan* or wide or action# or influen*))) | 399 |
| S62 | (TI ((teen or teens or teenage*) N2 (led or leader* or participat* or mediat* or helper# or resistan* or wide or action# or influen*))) OR (AB ((teen or teens or teenage*) N2 (led or leader* or participat* or mediat* or helper# or resistan* or wide or action# or influen*))) | 427 |
| S63 | (TI (adolescen* N2 (led or leader* or participat* or mediat* or helper# or resistan* or wide or action# or influen*))) OR (AB (adolescen* N2 (led or leader* or participat* or mediat* or helper# or resistan* or wide or action# or influen*))) | 4,697 |
| S64 | (TI (youth# N2 (led or leader* or participat* or mediat* or helper# or resistan* or wide or action# or influen*))) OR (AB (youth# N2 (led or leader* or participat* or mediat* or helper# or resistan* or wide or action# or influen*))) | 2,095 |
| S65 | (TI (student# N2 (led or leader* or participat* or mediat* or helper# or resistan* or wide or action# or influen* or relationship*))) OR (AB (student# N2 (led or leader* or participat* or mediat* or helper# or resistan* or wide or action# or influen* or relationship*))) NOT (MH "Students, Health Occupations+") | 8,370 |
| S66 | (TI (teach* N2 (led or leader* or participat* or mediat* or helper# or resistan* or wide or action# or influen* or relationship*))) OR (AB (teach* N2 (led or leader* or participat* or mediat* or helper# or resistan* or wide or action# or influen* or relationship*))) | 3,339 |
| S67 | (TI (curricul* N2 (led or leader* or participat* or mediat* or helper# or resistan* or wide or action# or influen* or relationship*))) OR (AB (curricul* N2 (led or leader* or participat* or mediat* or helper# or resistan* or wide or action# or influen* or relationship*))) | 892 |
| S68 | (TI (administrat* N2 (led or leader* or participat* or mediat* or helper# or resistan* or wide or action# or influen* or relationship*))) OR (AB (administrat* N2 (led or leader* or participat* or mediat* or helper# or resistan* or wide or action# or influen* or relationship*))) | 2,559 |
| S69 | (TI ((personnel or staff) N2 (led or leader* or participat* or mediat* or helper# or resistan* or wide or action# or influen* or relationship*))) OR (AB ((personnel or staff) N2 (led or leader* or participat* or mediat* or helper# or resistan* or wide or action# or influen* or relationship*))) | 5,518 |
| S70 | (TI (volunteer* N2 (led or leader* or participat* or mediat* or helper# or resistan* or wide or action# or influen* or relationship*))) OR (AB (volulnteer* N2 (led or leader* or participat* or mediat* or helper# or resistan* or wide or action# or influen* or relationship*))) | 199 |
| S71 | (TI ((advocacy or advocate) N2 (led or leader* or participat* or mediat* or helper# or resistan* or wide or action# or influen* or relationship*))) OR (AB ((advocacy or advocate) N2 (led or leader* or participat* or mediat* or helper# or resistan* or wide or action# or influen* or relationship*))) | 1,454 |
| S72 | (TI (parent* N2 (led or leader* or participat* or mediat* or helper# or resistan* or wide or action# or influen*))) OR (AB (parent* N2 (led or leader* or participat* or mediat* or helper# or resistan* or wide or action# or influen*))) | 7,941 |
| S73 | (MH "Social Environment") | 12,447 |
| S74 | (TI (environment* N2 (led or leader* or participat* or mediat* or helper# or resistan* or wide or action# or influen* or relationship*))) OR (AB (environment* N2 (led or leader* or participat* or mediat* or helper# or resistan* or wide or action# or influen* or relationship*))) | 9,463 |
| S75 | (TI ("socio-ecolog*" N2 (led or leader* or participat* or mediat* or helper# or resistan* or wide or action# or influen* or relationship*))) OR (AB ("socio-ecolog*" N2 (led or leader* or participat* or mediat* or helper# or resistan* or wide or action# or influen* or relationship*))) | 34 |
| S76 | (TI (learn* N2 (led or leader* or participat* or mediat* or helper# or resistan* or wide or action# or influen* or relationship*))) OR (AB (learn* N2 (led or leader* or participat* or mediat* or helper# or resistan* or wide or action# or influen* or relationship*))) | 5,557 |
| S77 | (MH "School Policies") | 2,055 |
| S78 | (TI ((policy or policies) N2 (led or leader* or participat* or mediat* or helper# or resistan* or wide or action# or influen* or relationship*))) OR (AB ((policy or policies) N2 (led or leader* or participat* or mediat* or helper# or resistan* or wide or action# or influen* or relationship*))) | 5,847 |
| S79 | S54 OR S55 OR S56 OR S57 OR S58 OR S59 OR S60 OR S61 OR S62 OR S63 OR S64 OR S65 OR S66 OR S67 OR S68 OR S69 OR S70 OR S71 OR S72 OR S73 OR S74 OR S75 OR S76 OR S77 OR S78 | 108,750 |
| S80 | S53 AND S79 | 15,903 |
| S81 | S46 OR S47 OR S48 OR S49 OR S50 OR S80 | 54,065 |
| S82 | (MH "Smoking Cessation Programs") | 2,492 |
| S83 | (MH "Smoking Cessation") | 21,536 |
| S84 | (MH "Smoking/PC") | 8,148 |
| S85 | (TI ((smoking or smoked or smoke or smoker) N5 (reduc* or decreas* or increas* or lower* or fewer or improv* or enhance* or extend* or develop* or prevalen* or daily or weekly or current or rate or rates or behavio#r* or knowledge or prevent* or attitude* or avoid* or perceive* or percept* or promot* or program* or change*))) OR (AB ((smoking or smoked or smoke or smoker) N5 (reduc* or decreas* or increas* or lower* or fewer or improv* or enhance* or extend* or develop* or prevalen* or daily or weekly or current or rate or rates or behavio#r* or knowledge or prevent* or attitude* or avoid* or perceive* or percept* or promot* or program* or change*))) | 38,829 |
| S86 | (TI (tobacco N5 (reduc* or decreas* or increas* or lower* or fewer or improv* or enhance* or extend* or develop* or prevalen* or daily or weekly or current or rate or rates or behavio#r* or knowledge or prevent* or attitude* or avoid* or perceive* or percept* or promot* or program* or change*)) OR (AB (tobacco N5 (reduc* or decreas* or increas* or lower* or fewer or improv* or enhance* or extend* or develop* or prevalen* or daily or weekly or current or rate or rates or behavio#r* or knowledge or prevent* or attitude* or avoid* or perceive* or percept* or promot* or program* or change*)) | 12,227 |
| S87 | (TI (cigarette# N5 (reduc* or decreas* or increas* or lower* or fewer or improv* or enhance* or extend* or develop* or prevalen* or daily or weekly or current or rate or rates or behavio#r* or knowledge or prevent* or attitude* or avoid* or perceive* or percept* or promot* or program* or change*)) OR (AB (cigarette# N5 (reduc* or decreas* or increas* or lower* or fewer or improv* or enhance* or extend* or develop* or prevalen* or daily or weekly or current or rate or rates or behavio#r* or knowledge or prevent* or attitude* or avoid* or perceive* or percept* or promot* or program* or change*)) | 9,518 |
| S88 | (TI (marijuana N5 (reduc* or decreas* or increas* or lower* or fewer or improv* or enhance* or extend* or develop* or prevalen* or daily or weekly or current or rate or rates or behavio#r* or knowledge or prevent* or attitude* or avoid* or perceive* or percept* or promot* or program* or change*)) OR (AB (marijuana N5 (reduc* or decreas* or increas* or lower* or fewer or improv* or enhance* or extend* or develop* or prevalen* or daily or weekly or current or rate or rates or behavio#r* or knowledge or prevent* or attitude* or avoid* or perceive* or percept* or promot* or program* or change*)) | 2,658 |
| S89 | (TI (cannabis N5 (reduc* or decreas* or increas* or lower* or fewer or improv* or enhance* or extend* or develop* or prevalen* or daily or weekly or current or rate or rates or behavio#r* or knowledge or prevent* or attitude* or avoid* or perceive* or percept* or promot* or program* or change*)) OR (AB (cannabis N5 (reduc* or decreas* or increas* or lower* or fewer or improv* or enhance* or extend* or develop* or prevalen* or daily or weekly or current or rate or rates or behavio#r* or knowledge or prevent* or attitude* or avoid* or perceive* or percept* or promot* or program* or change*)) | 3,210 |
| S90 | (TI (solvent# N5 (reduc* or decreas* or increas* or lower* or fewer or improv* or enhance* or extend* or develop* or prevalen* or daily or weekly or current or rate or rates or behavio#r* or knowledge or prevent* or attitude* or avoid* or perceive* or percept* or promot* or program* or change*)) OR (AB (solvent# N5 (reduc* or decreas* or increas* or lower* or fewer or improv* or enhance* or extend* or develop* or prevalen* or daily or weekly or current or rate or rates or behavio#r* or knowledge or prevent* or attitude* or avoid* or perceive* or percept* or promot* or program* or change*)) | 425 |
| S91 | (MH "Substance Use Disorders+/PC") | 20,871 |
| S92 | (MH "Alcohol Drinking/PC") OR (MH "Binge Drinking/PC") | 2,489 |
| S93 | (TI (alcohol N5 (reduc* or decreas* or increas* or lower* or fewer or improv* or enhance* or extend* or develop* or prevalen* or daily or weekly or current or rate or rates or behavio#r* or knowledge or prevent* or attitude* or avoid* or perceive* or percept* or promot* or program* or change*)) OR (AB (alcohol N5 (reduc* or decreas* or increas* or lower* or fewer or improv* or enhance* or extend* or develop* or prevalen* or daily or weekly or current or rate or rates or behavio#r* or knowledge or prevent* or attitude* or avoid* or perceive* or percept* or promot* or program* or change*)) | 28,445 |
| S94 | (TI ("binge drink*" N5 (reduc* or decreas* or increas* or lower* or fewer or improv* or enhance* or extend* or develop* or prevalen* or daily or weekly or current or rate or rates or behavio#r* or knowledge or prevent* or attitude* or avoid* or perceive* or percept* or promot* or program* or change*)) OR (AB ("binge drink*" N5 (reduc* or decreas* or increas* or lower* or fewer or improv* or enhance* or extend* or develop* or prevalen* or daily or weekly or current or rate or rates or behavio#r* or knowledge or prevent* or attitude* or avoid* or perceive* or percept* or promot* or program* or change*)) | 1,466 |
| S95 | (TI ("underage drink*" N5 (reduc* or decreas* or increas* or lower* or fewer or improv* or enhance* or extend* or develop* or prevalen* or daily or weekly or current or rate or rates or behavio#r* or knowledge or prevent* or attitude* or avoid* or perceive* or percept* or promot* or program* or change*)) OR (AB ("underage drink*" N5 (reduc* or decreas* or increas* or lower* or fewer or improv* or enhance* or extend* or develop* or prevalen* or daily or weekly or current or rate or rates or behavio#r* or knowledge or prevent* or attitude* or avoid* or perceive* or percept* or promot* or program* or change*)) | 191 |
| S96 | (TI (substance# N5 (reduc* or decreas* or increas* or lower* or fewer or improv* or enhance* or extend* or develop* or prevalen* or daily or weekly or current or rate or rates or behavio#r* or knowledge or prevent* or attitude* or avoid* or perceive* or percept* or promot* or program* or change*)) OR (AB (substance# N5 (reduc* or decreas* or increas* or lower* or fewer or improv* or enhance* or extend* or develop* or prevalen* or daily or weekly or current or rate or rates or behavio#r* or knowledge or prevent* or attitude* or avoid* or perceive* or percept* or promot* or program* or change*)) | 17,742 |
| S97 | (TI (drug# N5 (reduc* or decreas* or increas* or lower* or fewer or improv* or enhance* or extend* or develop* or prevalen* or daily or weekly or current or rate or rates or behavio#r* or knowledge or prevent* or attitude* or avoid* or perceive* or percept* or promot* or program* or change*)) OR (AB (drug# N5 (reduc* or decreas* or increas* or lower* or fewer or improv* or enhance* or extend* or develop* or prevalen* or daily or weekly or current or rate or rates or behavio#r* or knowledge or prevent* or attitude* or avoid* or perceive* or percept* or promot* or program* or change*)) | 71,245 |
| S98 | (MH "Academic Performance+") | 13,721 |
| S99 | (TI ("education* attain*" N5 (reduc* or decreas* or increas* or lower* or fewer or improv* or enhance* or extend* or develop* or prevalen* or daily or weekly or current or rate or rates or behavio#r* or knowledge or prevent* or attitude* or avoid* or perceive* or percept* or promot* or program* or change*)) OR (AB ("education* attain*" N5 (reduc* or decreas* or increas* or lower* or fewer or improv* or enhance* or extend* or develop* or prevalen* or daily or weekly or current or rate or rates or behavio#r* or knowledge or prevent* or attitude* or avoid* or perceive* or percept* or promot* or program* or change*)) | 1,435 |
| S100 | (TI ("academic attain*" N5 (reduc* or decreas* or increas* or lower* or fewer or improv* or enhance* or extend* or develop* or prevalen* or daily or weekly or current or rate or rates or behavio#r* or knowledge or prevent* or attitude* or avoid* or perceive* or percept* or promot* or program* or change*)) OR (AB ("academic attain*" N5 (reduc* or decreas* or increas* or lower* or fewer or improv* or enhance* or extend* or develop* or prevalen* or daily or weekly or current or rate or rates or behavio#r* or knowledge or prevent* or attitude* or avoid* or perceive* or percept* or promot* or program* or change*)) | 42 |
| S101 | (TI ("student* attain*" N5 (reduc* or decreas* or increas* or lower* or fewer or improv* or enhance* or extend* or develop* or prevalen* or daily or weekly or current or rate or rates or behavio#r* or knowledge or prevent* or attitude* or avoid* or perceive* or percept* or promot* or program* or change*)) OR (AB ("student* attain*" N5 (reduc* or decreas* or increas* or lower* or fewer or improv* or enhance* or extend* or develop* or prevalen* or daily or weekly or current or rate or rates or behavio#r* or knowledge or prevent* or attitude* or avoid* or perceive* or percept* or promot* or program* or change*)) | 16 |
| S102 | (TI ("education* achieve*" N5 (reduc* or decreas* or increas* or lower* or fewer or improv* or enhance* or extend* or develop* or prevalen* or daily or weekly or current or rate or rates or behavio#r* or knowledge or prevent* or attitude* or avoid* or perceive* or percept* or promot* or program* or change*)) OR (AB ("education* achieve*" N5 (reduc* or decreas* or increas* or lower* or fewer or improv* or enhance* or extend* or develop* or prevalen* or daily or weekly or current or rate or rates or behavio#r* or knowledge or prevent* or attitude* or avoid* or perceive* or percept* or promot* or program* or change*)) | 231 |
| S103 | (TI ("academic achieve*" N5 (reduc* or decreas* or increas* or lower* or fewer or improv* or enhance* or extend* or develop* or prevalen* or daily or weekly or current or rate or rates or behavio#r* or knowledge or prevent* or attitude* or avoid* or perceive* or percept* or promot* or program* or change*)) OR (AB ("academic achieve*" N5 (reduc* or decreas* or increas* or lower* or fewer or improv* or enhance* or extend* or develop* or prevalen* or daily or weekly or current or rate or rates or behavio#r* or knowledge or prevent* or attitude* or avoid* or perceive* or percept* or promot* or program* or change*)) | 996 |
| S104 | (TI ("student* achieve*" N5 (reduc* or decreas* or increas* or lower* or fewer or improv* or enhance* or extend* or develop* or prevalen* or daily or weekly or current or rate or rates or behavio#r* or knowledge or prevent* or attitude* or avoid* or perceive* or percept* or promot* or program* or change*)) OR (AB ("student* achieve*" N5 (reduc* or decreas* or increas* or lower* or fewer or improv* or enhance* or extend* or develop* or prevalen* or daily or weekly or current or rate or rates or behavio#r* or knowledge or prevent* or attitude* or avoid* or perceive* or percept* or promot* or program* or change*)) | 187 |
| S105 | (TI ("education* perform*" N5 (reduc* or decreas* or increas* or lower* or fewer or improv* or enhance* or extend* or develop* or prevalen* or daily or weekly or current or rate or rates or behavio#r* or knowledge or prevent* or attitude* or avoid* or perceive* or percept* or promot* or program* or change*)) OR (AB ("education* perform*" N5 (reduc* or decreas* or increas* or lower* or fewer or improv* or enhance* or extend* or develop* or prevalen* or daily or weekly or current or rate or rates or behavio#r* or knowledge or prevent* or attitude* or avoid* or perceive* or percept* or promot* or program* or change*)) | 71 |
| S106 | (TI ("academic perform*" N5 (reduc* or decreas* or increas* or lower* or fewer or improv* or enhance* or extend* or develop* or prevalen* or daily or weekly or current or rate or rates or behavio#r* or knowledge or prevent* or attitude* or avoid* or perceive* or percept* or promot* or program* or change*)) OR (AB ("academic perform*" N5 (reduc* or decreas* or increas* or lower* or fewer or improv* or enhance* or extend* or develop* or prevalen* or daily or weekly or current or rate or rates or behavio#r* or knowledge or prevent* or attitude* or avoid* or perceive* or percept* or promot* or program* or change*)) | 1,198 |
| S107 | (TI ("student* perform*" N5 (reduc* or decreas* or increas* or lower* or fewer or improv* or enhance* or extend* or develop* or prevalen* or daily or weekly or current or rate or rates or behavio#r* or knowledge or prevent* or attitude* or avoid* or perceive* or percept* or promot* or program* or change*)) OR (AB ("student* perform*" N5 (reduc* or decreas* or increas* or lower* or fewer or improv* or enhance* or extend* or develop* or prevalen* or daily or weekly or current or rate or rates or behavio#r* or knowledge or prevent* or attitude* or avoid* or perceive* or percept* or promot* or program* or change*)) | 563 |
| S108 | (TI ("education* outcome#" N5 (reduc* or decreas* or increas* or lower* or fewer or improv* or enhance* or extend* or develop* or prevalen* or daily or weekly or current or rate or rates or behavio#r* or knowledge or prevent* or attitude* or avoid* or perceive* or percept* or promot* or program* or change*)) OR (AB ("education* outcome#" N5 (reduc* or decreas* or increas* or lower* or fewer or improv* or enhance* or extend* or develop* or prevalen* or daily or weekly or current or rate or rates or behavio#r* or knowledge or prevent* or attitude* or avoid* or perceive* or percept* or promot* or program* or change*)) | 513 |
| S109 | (TI ("academic outcome#" N5 (reduc* or decreas* or increas* or lower* or fewer or improv* or enhance* or extend* or develop* or prevalen* or daily or weekly or current or rate or rates or behavio#r* or knowledge or prevent* or attitude* or avoid* or perceive* or percept* or promot* or program* or change*)) OR (AB ("academic outcome#" N5 (reduc* or decreas* or increas* or lower* or fewer or improv* or enhance* or extend* or develop* or prevalen* or daily or weekly or current or rate or rates or behavio#r* or knowledge or prevent* or attitude* or avoid* or perceive* or percept* or promot* or program* or change*)) | 285 |
| S110 | (TI ("student* outcome#" N5 (reduc* or decreas* or increas* or lower* or fewer or improv* or enhance* or extend* or develop* or prevalen* or daily or weekly or current or rate or rates or behavio#r* or knowledge or prevent* or attitude* or avoid* or perceive* or percept* or promot* or program* or change*)) OR (AB ("student* outcome#" N5 (reduc* or decreas* or increas* or lower* or fewer or improv* or enhance* or extend* or develop* or prevalen* or daily or weekly or current or rate or rates or behavio#r* or knowledge or prevent* or attitude* or avoid* or perceive* or percept* or promot* or program* or change*)) | 247 |
| S111 | (TI ("education* commitment" N5 (reduc* or decreas* or increas* or lower* or fewer or improv* or enhance* or extend* or develop* or prevalen* or daily or weekly or current or rate or rates or behavio#r* or knowledge or prevent* or attitude* or avoid* or perceive* or percept* or promot* or program* or change*)) OR (AB ("education* commitment" N5 (reduc* or decreas* or increas* or lower* or fewer or improv* or enhance* or extend* or develop* or prevalen* or daily or weekly or current or rate or rates or behavio#r* or knowledge or prevent* or attitude* or avoid* or perceive* or percept* or promot* or program* or change*)) | 3 |
| S112 | (TI ("academic commitment" N5 (reduc* or decreas* or increas* or lower* or fewer or improv* or enhance* or extend* or develop* or prevalen* or daily or weekly or current or rate or rates or behavio#r* or knowledge or prevent* or attitude* or avoid* or perceive* or percept* or promot* or program* or change*)) OR (AB ("academic commitment" N5 (reduc* or decreas* or increas* or lower* or fewer or improv* or enhance* or extend* or develop* or prevalen* or daily or weekly or current or rate or rates or behavio#r* or knowledge or prevent* or attitude* or avoid* or perceive* or percept* or promot* or program* or change*)) | 5 |
| S113 | (TI ("student* commitment" N5 (reduc* or decreas* or increas* or lower* or fewer or improv* or enhance* or extend* or develop* or prevalen* or daily or weekly or current or rate or rates or behavio#r* or knowledge or prevent* or attitude* or avoid* or perceive* or percept* or promot* or program* or change*)) OR (AB ("student* commitment" N5 (reduc* or decreas* or increas* or lower* or fewer or improv* or enhance* or extend* or develop* or prevalen* or daily or weekly or current or rate or rates or behavio#r* or knowledge or prevent* or attitude* or avoid* or perceive* or percept* or promot* or program* or change*)) | 18 |
| S114 | (TI ("education* engage*" N5 (reduc* or decreas* or increas* or lower* or fewer or improv* or enhance* or extend* or develop* or prevalen* or daily or weekly or current or rate or rates or behavio#r* or knowledge or prevent* or attitude* or avoid* or perceive* or percept* or promot* or program* or change*)) OR (AB ("education* engage*" N5 (reduc* or decreas* or increas* or lower* or fewer or improv* or enhance* or extend* or develop* or prevalen* or daily or weekly or current or rate or rates or behavio#r* or knowledge or prevent* or attitude* or avoid* or perceive* or percept* or promot* or program* or change*)) | 18 |
| S115 | (TI ("academic engage*" N5 (reduc* or decreas* or increas* or lower* or fewer or improv* or enhance* or extend* or develop* or prevalen* or daily or weekly or current or rate or rates or behavio#r* or knowledge or prevent* or attitude* or avoid* or perceive* or percept* or promot* or program* or change*)) OR (AB ("academic engage*" N5 (reduc* or decreas* or increas* or lower* or fewer or improv* or enhance* or extend* or develop* or prevalen* or daily or weekly or current or rate or rates or behavio#r* or knowledge or prevent* or attitude* or avoid* or perceive* or percept* or promot* or program* or change*)) | 110 |
| S116 | (TI ("student* engage*" N5 (reduc* or decreas* or increas* or lower* or fewer or improv* or enhance* or extend* or develop* or prevalen* or daily or weekly or current or rate or rates or behavio#r* or knowledge or prevent* or attitude* or avoid* or perceive* or percept* or promot* or program* or change*)) OR (AB ("student* engage*" N5 (reduc* or decreas* or increas* or lower* or fewer or improv* or enhance* or extend* or develop* or prevalen* or daily or weekly or current or rate or rates or behavio#r* or knowledge or prevent* or attitude* or avoid* or perceive* or percept* or promot* or program* or change*)) | 495 |
| S117 | (TI (dropout N5 (reduc* or decreas* or increas* or lower* or fewer or improv* or enhance* or extend* or develop* or prevalen* or daily or weekly or current or rate or rates or behavio#r* or knowledge or prevent* or attitude* or avoid* or perceive* or percept* or promot* or program* or change*)) OR (AB (dropout N5 (reduc* or decreas* or increas* or lower* or fewer or improv* or enhance* or extend* or develop* or prevalen* or daily or weekly or current or rate or rates or behavio#r* or knowledge or prevent* or attitude* or avoid* or perceive* or percept* or promot* or program* or change*)) | 2,139 |
| S118 | (TI (absentee* N5 (reduc* or decreas* or increas* or lower* or fewer or improv* or enhance* or extend* or develop* or prevalen* or daily or weekly or current or rate or rates or behavio#r* or knowledge or prevent* or attitude* or avoid* or perceive* or percept* or promot* or program* or change*)) OR (AB (absentee* N5 (reduc* or decreas* or increas* or lower* or fewer or improv* or enhance* or extend* or develop* or prevalen* or daily or weekly or current or rate or rates or behavio#r* or knowledge or prevent* or attitude* or avoid* or perceive* or percept* or promot* or program* or change*)) | 1,246 |
| S119 | (MH "Aggression/PC") OR (MH "Bullying+/PC") OR (MH "Student Abuse/PC") OR (MH "Verbal Abuse/PC") OR (MH "Violence/PC") OR (MH "Exposure to Violence/PC") OR (MH "Gender-Based Violence/PC") OR (MH "Gun Violence/PC") OR (MH "School Violence/PC") OR (MH "Assault and Battery/PC") | 7,312 |
| S120 | (MH "Bullying+/PC") | 2,025 |
| S121 | (MH "Juvenile Delinquency/PC") | 450 |
| S122 | (MH "Student Discipline") | 608 |
| S123 | (MH "Disruptive Behavior/PC") | 667 |
| S124 | (TI ((violent or violence) N5 (reduc* or decreas* or increas* or lower* or fewer or improv* or enhance* or extend* or develop* or prevalen* or daily or weekly or current or rate or rates or behavio#r* or knowledge or prevent* or attitude* or avoid* or perceive* or percept* or promot* or program* or change*))) OR (AB ((violent or violence) N5 (reduc* or decreas* or increas* or lower* or fewer or improv* or enhance* or extend* or develop* or prevalen* or daily or weekly or current or rate or rates or behavio#r* or knowledge or prevent* or attitude* or avoid* or perceive* or percept* or promot* or program* or change*))) | 15,752 |
| S125 | (TI ((aggression or aggressive) N5 (reduc* or decreas* or increas* or lower* or fewer or improv* or enhance* or extend* or develop* or prevalen* or daily or weekly or current or rate or rates or behavio#r* or knowledge or prevent* or attitude* or avoid* or perceive* or percept* or promot* or program* or change*))) OR (AB ((aggression or aggressive) N5 (reduc* or decreas* or increas* or lower* or fewer or improv* or enhance* or extend* or develop* or prevalen* or daily or weekly or current or rate or rates or behavio#r* or knowledge or prevent* or attitude* or avoid* or perceive* or percept* or promot* or program* or change*))) | 12,163 |
| S126 | (TI ((hostile or hostility) N5 (reduc* or decreas* or increas* or lower* or fewer or improv* or enhance* or extend* or develop* or prevalen* or daily or weekly or current or rate or rates or behavio#r* or knowledge or prevent* or attitude* or avoid* or perceive* or percept* or promot* or program* or change*))) OR (AB ((hostile or hostility) N5 (reduc* or decreas* or increas* or lower* or fewer or improv* or enhance* or extend* or develop* or prevalen* or daily or weekly or current or rate or rates or behavio#r* or knowledge or prevent* or attitude* or avoid* or perceive* or percept* or promot* or program* or change*))) | 1,264 |
| S127 | (TI (assault* N5 (reduc* or decreas* or increas* or lower* or fewer or improv* or enhance* or extend* or develop* or prevalen* or daily or weekly or current or rate or rates or behavio#r* or knowledge or prevent* or attitude* or avoid* or perceive* or percept* or promot* or program* or change*)) OR (AB (assault* N5 (reduc* or decreas* or increas* or lower* or fewer or improv* or enhance* or extend* or develop* or prevalen* or daily or weekly or current or rate or rates or behavio#r* or knowledge or prevent* or attitude* or avoid* or perceive* or percept* or promot* or program* or change*)) | 2,035 |
| S128 | (TI (crime N5 (reduc* or decreas* or increas* or lower* or fewer or improv* or enhance* or extend* or develop* or prevalen* or daily or weekly or current or rate or rates or behavio#r* or knowledge or prevent* or attitude* or avoid* or perceive* or percept* or promot* or program* or change*)) OR (AB (crime N5 (reduc* or decreas* or increas* or lower* or fewer or improv* or enhance* or extend* or develop* or prevalen* or daily or weekly or current or rate or rates or behavio#r* or knowledge or prevent* or attitude* or avoid* or perceive* or percept* or promot* or program* or change*)) | 1,948 |
| S129 | (TI ((abuse# or abusive) N5 (reduc* or decreas* or increas* or lower* or fewer or improv* or enhance* or extend* or develop* or prevalen* or daily or weekly or current or rate or rates or behavio#r* or knowledge or prevent* or attitude* or avoid* or perceive* or percept* or promot* or program* or change*))) OR (AB ((abuse# or abusive) N5 (reduc* or decreas* or increas* or lower* or fewer or improv* or enhance* or extend* or develop* or prevalen* or daily or weekly or current or rate or rates or behavio#r* or knowledge or prevent* or attitude* or avoid* or perceive* or percept* or promot* or program* or change*))) | 16,460 |
| S130 | (TI (misbehav* N5 (reduc* or decreas* or increas* or lower* or fewer or improv* or enhance* or extend* or develop* or prevalen* or daily or weekly or current or rate or rates or behavio#r* or knowledge or prevent* or attitude* or avoid* or perceive* or percept* or promot* or program* or change*)) OR (AB (misbehav* N5 (reduc* or decreas* or increas* or lower* or fewer or improv* or enhance* or extend* or develop* or prevalen* or daily or weekly or current or rate or rates or behavio#r* or knowledge or prevent* or attitude* or avoid* or perceive* or percept* or promot* or program* or change*)) | 70 |
| S131 | (TI (threat# N5 (reduc* or decreas* or increas* or lower* or fewer or improv* or enhance* or extend* or develop* or prevalen* or daily or weekly or current or rate or rates or behavio#r* or knowledge or prevent* or attitude* or avoid* or perceive* or percept* or promot* or program* or change*)) OR (AB (threat# N5 (reduc* or decreas* or increas* or lower* or fewer or improv* or enhance* or extend* or develop* or prevalen* or daily or weekly or current or rate or rates or behavio#r* or knowledge or prevent* or attitude* or avoid* or perceive* or percept* or promot* or program* or change*)) | 5,084 |
| S132 | (TI (conduct N5 (reduc* or decreas* or increas* or lower* or fewer or improv* or enhance* or extend* or develop* or prevalen* or daily or weekly or current or rate or rates or behavio#r* or knowledge or prevent* or attitude* or avoid* or perceive* or percept* or promot* or program* or change*)) OR (AB (conduct N5 (reduc* or decreas* or increas* or lower* or fewer or improv* or enhance* or extend* or develop* or prevalen* or daily or weekly or current or rate or rates or behavio#r* or knowledge or prevent* or attitude* or avoid* or perceive* or percept* or promot* or program* or change*)) | 5,009 |
| S133 | (TI (delinquen* N5 (reduc* or decreas* or increas* or lower* or fewer or improv* or enhance* or extend* or develop* or prevalen* or daily or weekly or current or rate or rates or behavio#r* or knowledge or prevent* or attitude* or avoid* or perceive* or percept* or promot* or program* or change*)) OR (AB (delinquen* N5 (reduc* or decreas* or increas* or lower* or fewer or improv* or enhance* or extend* or develop* or prevalen* or daily or weekly or current or rate or rates or behavio#r* or knowledge or prevent* or attitude* or avoid* or perceive* or percept* or promot* or program* or change*)) | 1,440 |
| S134 | (TI ("disrupt* behavio#r*" N5 (reduc* or decreas* or increas* or lower* or fewer or improv* or enhance* or extend* or develop* or prevalen* or daily or weekly or current or rate or rates or behavio#r* or knowledge or prevent* or attitude* or avoid* or perceive* or percept* or promot* or program* or change*)) OR (AB ("disrupt* behavio#r*" N5 (reduc* or decreas* or increas* or lower* or fewer or improv* or enhance* or extend* or develop* or prevalen* or daily or weekly or current or rate or rates or behavio#r* or knowledge or prevent* or attitude* or avoid* or perceive* or percept* or promot* or program* or change*)) | 2,123 |
| S135 | (TI ("problem* behavio#r*" N5 (reduc* or decreas* or increas* or lower* or fewer or improv* or enhance* or extend* or develop* or prevalen* or daily or weekly or current or rate or rates or behavio#r* or knowledge or prevent* or attitude* or avoid* or perceive* or percept* or promot* or program* or change*)) OR (AB ("problem* behavio#r*" N5 (reduc* or decreas* or increas* or lower* or fewer or improv* or enhance* or extend* or develop* or prevalen* or daily or weekly or current or rate or rates or behavio#r* or knowledge or prevent* or attitude* or avoid* or perceive* or percept* or promot* or program* or change*)) | 3,403 |
| S136 | (TI ("volatile behavio#r*" N5 (reduc* or decreas* or increas* or lower* or fewer or improv* or enhance* or extend* or develop* or prevalen* or daily or weekly or current or rate or rates or behavio#r* or knowledge or prevent* or attitude* or avoid* or perceive* or percept* or promot* or program* or change*)) OR (AB ("volatile behavio#r*" N5 (reduc* or decreas* or increas* or lower* or fewer or improv* or enhance* or extend* or develop* or prevalen* or daily or weekly or current or rate or rates or behavio#r* or knowledge or prevent* or attitude* or avoid* or perceive* or percept* or promot* or program* or change*)) | 5 |
| S137 | (TI ((antisocial or "anti-social") N5 (reduc* or decreas* or increas* or lower* or fewer or improv* or enhance* or extend* or develop* or prevalen* or daily or weekly or current or rate or rates or behavio#r* or knowledge or prevent* or attitude* or avoid* or perceive* or percept* or promot* or program* or change*))) OR (AB ((antisocial or "anti-social") N5 (reduc* or decreas* or increas* or lower* or fewer or improv* or enhance* or extend* or develop* or prevalen* or daily or weekly or current or rate or rates or behavio#r* or knowledge or prevent* or attitude* or avoid* or perceive* or percept* or promot* or program* or change*))) | 2,204 |
| S138 | (TI (perpetrat* N5 (reduc* or decreas* or increas* or lower* or fewer or improv* or enhance* or extend* or develop* or prevalen* or daily or weekly or current or rate or rates or behavio#r* or knowledge or prevent* or attitude* or avoid* or perceive* or percept* or promot* or program* or change*)) OR (AB (perpetrat* N5 (reduc* or decreas* or increas* or lower* or fewer or improv* or enhance* or extend* or develop* or prevalen* or daily or weekly or current or rate or rates or behavio#r* or knowledge or prevent* or attitude* or avoid* or perceive* or percept* or promot* or program* or change*)) | 1,696 |
| S139 | (TI (bully* N5 (reduc* or decreas* or increas* or lower* or fewer or improv* or enhance* or extend* or develop* or prevalen* or daily or weekly or current or rate or rates or behavio#r* or knowledge or prevent* or attitude* or avoid* or perceive* or percept* or promot* or program* or change*)) OR (AB (bully* N5 (reduc* or decreas* or increas* or lower* or fewer or improv* or enhance* or extend* or develop* or prevalen* or daily or weekly or current or rate or rates or behavio#r* or knowledge or prevent* or attitude* or avoid* or perceive* or percept* or promot* or program* or change*)) | 2,704 |
| S140 | (TI (victim* N5 (reduc* or decreas* or increas* or lower* or fewer or improv* or enhance* or extend* or develop* or prevalen* or daily or weekly or current or rate or rates or behavio#r* or knowledge or prevent* or attitude* or avoid* or perceive* or percept* or promot* or program* or change*)) OR (AB (victim* N5 (reduc* or decreas* or increas* or lower* or fewer or improv* or enhance* or extend* or develop* or prevalen* or daily or weekly or current or rate or rates or behavio#r* or knowledge or prevent* or attitude* or avoid* or perceive* or percept* or promot* or program* or change*)) | 6,186 |
| S141 | (TI (cyberbully* N5 (reduc* or decreas* or increas* or lower* or fewer or improv* or enhance* or extend* or develop* or prevalen* or daily or weekly or current or rate or rates or behavio#r* or knowledge or prevent* or attitude* or avoid* or perceive* or percept* or promot* or program* or change*)) OR (AB (cyberbully* N5 (reduc* or decreas* or increas* or lower* or fewer or improv* or enhance* or extend* or develop* or prevalen* or daily or weekly or current or rate or rates or behavio#r* or knowledge or prevent* or attitude* or avoid* or perceive* or percept* or promot* or program* or change*)) | 428 |
| S142 | (TI (conflict* N5 (reduc* or decreas* or increas* or lower* or fewer or improv* or enhance* or extend* or develop* or prevalen* or daily or weekly or current or rate or rates or behavio#r* or knowledge or prevent* or attitude* or avoid* or perceive* or percept* or promot* or program* or change*)) OR (AB (conflict* N5 (reduc* or decreas* or increas* or lower* or fewer or improv* or enhance* or extend* or develop* or prevalen* or daily or weekly or current or rate or rates or behavio#r* or knowledge or prevent* or attitude* or avoid* or perceive* or percept* or promot* or program* or change*)) | 8,489 |
| S143 | (TI ("positive behavio#r*" N5 (reduc* or decreas* or increas* or lower* or fewer or improv* or enhance* or extend* or develop* or prevalen* or daily or weekly or current or rate or rates or behavio#r* or knowledge or prevent* or attitude* or avoid* or perceive* or percept* or promot* or program* or change*)) OR (AB ("positive behavio#r*" N5 (reduc* or decreas* or increas* or lower* or fewer or improv* or enhance* or extend* or develop* or prevalen* or daily or weekly or current or rate or rates or behavio#r* or knowledge or prevent* or attitude* or avoid* or perceive* or percept* or promot* or program* or change*)) | 1,422 |
| S144 | (TI ("improv* behavio#r*" N5 (reduc* or decreas* or increas* or lower* or fewer or improv* or enhance* or extend* or develop* or prevalen* or daily or weekly or current or rate or rates or behavio#r* or knowledge or prevent* or attitude* or avoid* or perceive* or percept* or promot* or program* or change*)) OR (AB ("improv* behavio#r*" N5 (reduc* or decreas* or increas* or lower* or fewer or improv* or enhance* or extend* or develop* or prevalen* or daily or weekly or current or rate or rates or behavio#r* or knowledge or prevent* or attitude* or avoid* or perceive* or percept* or promot* or program* or change*)) | 720 |
| S145 | (TI ("social behavio#r*" N5 (reduc* or decreas* or increas* or lower* or fewer or improv* or enhance* or extend* or develop* or prevalen* or daily or weekly or current or rate or rates or behavio#r* or knowledge or prevent* or attitude* or avoid* or perceive* or percept* or promot* or program* or change*)) OR (AB ("social behavio#r*" N5 (reduc* or decreas* or increas* or lower* or fewer or improv* or enhance* or extend* or develop* or prevalen* or daily or weekly or current or rate or rates or behavio#r* or knowledge or prevent* or attitude* or avoid* or perceive* or percept* or promot* or program* or change*)) | 2,665 |
| S146 | (TI (("social-emotion*" or "socio-emotion*") N5 (reduc* or decreas* or increas* or lower* or fewer or improv* or enhance* or extend* or develop* or prevalen* or daily or weekly or current or rate or rates or behavio#r* or knowledge or prevent* or attitude* or avoid* or perceive* or percept* or promot* or program* or change*))) OR (AB (("social-emotion*" or "socio-emotion*") N5 (reduc* or decreas* or increas* or lower* or fewer or improv* or enhance* or extend* or develop* or prevalen* or daily or weekly or current or rate or rates or behavio#r* or knowledge or prevent* or attitude* or avoid* or perceive* or percept* or promot* or program* or change*))) | 1,557 |
| S147 | (TI ("social-charact*" N5 (reduc* or decreas* or increas* or lower* or fewer or improv* or enhance* or extend* or develop* or prevalen* or daily or weekly or current or rate or rates or behavio#r* or knowledge or prevent* or attitude* or avoid* or perceive* or percept* or promot* or program* or change*)) OR (AB ("social-charact*" N5 (reduc* or decreas* or increas* or lower* or fewer or improv* or enhance* or extend* or develop* or prevalen* or daily or weekly or current or rate or rates or behavio#r* or knowledge or prevent* or attitude* or avoid* or perceive* or percept* or promot* or program* or change*)) | 108 |
| S148 | (TI ("social-inclusion" N5 (reduc* or decreas* or increas* or lower* or fewer or improv* or enhance* or extend* or develop* or prevalen* or daily or weekly or current or rate or rates or behavio#r* or knowledge or prevent* or attitude* or avoid* or perceive* or percept* or promot* or program* or change*)) OR (AB ("social-inclusion" N5 (reduc* or decreas* or increas* or lower* or fewer or improv* or enhance* or extend* or develop* or prevalen* or daily or weekly or current or rate or rates or behavio#r* or knowledge or prevent* or attitude* or avoid* or perceive* or percept* or promot* or program* or change*)) | 558 |
| S149 | (TI ("social develop*" N5 (reduc* or decreas* or increas* or lower* or fewer or improv* or enhance* or extend* or develop* or prevalen* or daily or weekly or current or rate or rates or behavio#r* or knowledge or prevent* or attitude* or avoid* or perceive* or percept* or promot* or program* or change*)) OR (AB ("social develop*" N5 (reduc* or decreas* or increas* or lower* or fewer or improv* or enhance* or extend* or develop* or prevalen* or daily or weekly or current or rate or rates or behavio#r* or knowledge or prevent* or attitude* or avoid* or perceive* or percept* or promot* or program* or change*)) | 1,447 |
| S150 | (TI ("social competen*" N5 (reduc* or decreas* or increas* or lower* or fewer or improv* or enhance* or extend* or develop* or prevalen* or daily or weekly or current or rate or rates or behavio#r* or knowledge or prevent* or attitude* or avoid* or perceive* or percept* or promot* or program* or change*)) OR (AB ("social competen*" N5 (reduc* or decreas* or increas* or lower* or fewer or improv* or enhance* or extend* or develop* or prevalen* or daily or weekly or current or rate or rates or behavio#r* or knowledge or prevent* or attitude* or avoid* or perceive* or percept* or promot* or program* or change*)) | 698 |
| S151 | (TI ("emotion* develop*" N5 (reduc* or decreas* or increas* or lower* or fewer or improv* or enhance* or extend* or develop* or prevalen* or daily or weekly or current or rate or rates or behavio#r* or knowledge or prevent* or attitude* or avoid* or perceive* or percept* or promot* or program* or change*)) OR (AB ("emotion* develop*" N5 (reduc* or decreas* or increas* or lower* or fewer or improv* or enhance* or extend* or develop* or prevalen* or daily or weekly or current or rate or rates or behavio#r* or knowledge or prevent* or attitude* or avoid* or perceive* or percept* or promot* or program* or change*)) | 1,048 |
| S152 | (TI (conduct N5 (reduc* or decreas* or increas* or lower* or fewer or improv* or enhance* or extend* or develop* or prevalen* or daily or weekly or current or rate or rates or behavio#r* or knowledge or prevent* or attitude* or avoid* or perceive* or percept* or promot* or program* or change*)) OR (AB (conduct N5 (reduc* or decreas* or increas* or lower* or fewer or improv* or enhance* or extend* or develop* or prevalen* or daily or weekly or current or rate or rates or behavio#r* or knowledge or prevent* or attitude* or avoid* or perceive* or percept* or promot* or program* or change*)) | 5,009 |
| S153 | (TI (respect N5 (reduc* or decreas* or increas* or lower* or fewer or improv* or enhance* or extend* or develop* or prevalen* or daily or weekly or current or rate or rates or behavio#r* or knowledge or prevent* or attitude* or avoid* or perceive* or percept* or promot* or program* or change*)) OR (AB (respect N5 (reduc* or decreas* or increas* or lower* or fewer or improv* or enhance* or extend* or develop* or prevalen* or daily or weekly or current or rate or rates or behavio#r* or knowledge or prevent* or attitude* or avoid* or perceive* or percept* or promot* or program* or change*)) | 8,978 |
| S154 | (TI ((safety or safe) N5 (reduc* or decreas* or increas* or lower* or fewer or improv* or enhance* or extend* or develop* or prevalen* or daily or weekly or current or rate or rates or behavio#r* or knowledge or prevent* or attitude* or avoid* or perceive* or percept* or promot* or program* or change*))) OR (AB ((safety or safe) N5 (reduc* or decreas* or increas* or lower* or fewer or improv* or enhance* or extend* or develop* or prevalen* or daily or weekly or current or rate or rates or behavio#r* or knowledge or prevent* or attitude* or avoid* or perceive* or percept* or promot* or program* or change*))) | 57,805 |
| S155 | S82 OR S83 OR S84 OR S85 OR S86 OR S87 OR S88 OR S89 OR S90 OR S91 OR S92 OR S93 OR S94 OR S95 OR S96 OR S97 OR S98 OR S99 OR S100 OR S101 OR S102 OR S103 OR S104 OR S105 OR S106 OR S107 OR S108 OR S109 OR S110 OR S111 OR S112 OR S113 OR S114 OR S115 OR S116 OR S117 OR S118 OR S119 OR S120 OR S121 OR S122 OR S123 OR S124 OR S125 OR S126 OR S127 OR S128 OR S129 OR S130 OR S131 OR S132 OR S133 OR S134 OR S135 OR S136 OR S137 OR S138 OR S139 OR S140 OR S141 OR S142 OR S143 OR S144 OR S145 OR S146 OR S147 OR S148 OR S149 OR S150 OR S151 OR S152 OR S153 OR S154 | 332,714 |
| S156 | (MH "Program Development+") | 87,675 |
| S157 | (MH "Evaluation Research+") | 281,842 |
| S158 | (MH "Validation Studies") | 286,498 |
| S159 | (MH "Clinical Trials+") | 317,066 |
| S160 | (TI prevent*) OR (AB prevent*) | 388,684 |
| S161 | (TI program*) OR (AB program*) | 382,869 |
| S162 | (TI intervent*) OR (AB intervent*) | 462,665 |
| S163 | (TI "restorative approach*") OR (AB "restorative approach*") | 89 |
| S164 | (TI "control school#") OR (AB "control school#") | 500 |
| S165 | (TI (trial or trials)) OR (AB (trial or trials)) | 361,067 |
| S166 | S156 OR S157 OR S158 OR S159 OR S160 OR S161 OR S162 OR S163 OR S164 OR S165 | 1,625,285 |
| S167 | S45 AND S81 AND S155 AND S166 | 7,075 |

## ClinicalTrials.gov

| Database name | ClinicalTrials.gov |
| --- | --- |
| Database platform | [www.clinicaltrials.gov](http://www.clinicaltrials.gov) |
| Dates of database coverage | Complete database to date searched. |
| Date searched | 11/05/2021 |
| Searched by | JF |
| Number of results | 1162 |
| EndNote import order | 20 |
| Number of results once duplicates removed | 165 |
| Search strategy notes | None |

Title: school OR schools OR schoolwide

## Wiley Cochrane Library

| Database name | Cochrane Library |
| --- | --- |
| Database platform | Wiley |
| Dates of database coverage | Issue 4 of 12, April 2021 |
| Date searched | 13/05/2021 |
| Searched by | JF |
| Number of results | Alcohol & drugs   - CDSR: 26 - CENTRAL: 1615   Academic attainment   - CDSR: 3 - CENTRAL: 429   Violence   - CDSR: 22 - CENTRAL: 1638 |
| EndNote import order | 19 |
| Number of results once duplicates removed | 989 |
| Search strategy notes | * is used for truncation. ? is used for an optional wildcard. Search strings ending with :ti,ab,kw search the title, abstract and keywords only. NEXT searches for terms next to each other, in the order entered. NEAR/*n* searches for terms within *n* words of each other.  Due to interface issues, the search was split into three parts:   1. population and whole-school interventions which have an impact on levels of violence, 2. population and whole-school interventions which have an impact on substance abuse, 3. population and whole school interventions which have an impact on educational attainment   As the Cochrane Library contains systematic reviews or trials, the limit to quantitative methodologies was not added. |

| ID | Search | | Hits | |
| --- | --- | --- | --- | --- |
| #1 | MeSH descriptor: [Child] this term only | 50035 | |  |
| #2 | MeSH descriptor: [Adolescent] this term only | 105818 | |  |
| #3 | MeSH descriptor: [Minors] this term only | 10 | |  |
| #4 | MeSH descriptor: [Students] this term only | 2815 | |  |
| #5 | MeSH descriptor: [School Teachers] this term only | 135 | |  |
| #6 | MeSH descriptor: [Parents] explode all trees | 5230 | |  |
| #7 | (child*):ti,ab,kw | 164462 | |  |
| #8 | (boy?):ti,ab,kw | 6970 | |  |
| #9 | (girl?):ti,ab,kw | 7655 | |  |
| #10 | (schoolage or (school near/1 age)):ti,ab,kw | 1364 | |  |
| #11 | (schoolchild*):ti,ab,kw | 1498 | |  |
| #12 | (youngster?):ti,ab,kw | 165 | |  |
| #13 | (minor?):ti,ab,kw | 17152 | |  |
| #14 | (pupil* or student*):ti,ab,kw | 41877 | |  |
| #15 | MeSH descriptor: [Pupil] this term only | 673 | |  |
| #16 | MeSH descriptor: [Students, Health Occupations] explode all trees | 1750 | |  |
| #17 | #15 or #16 | 2422 | |  |
| #18 | #14 NOT #17 | 39455 | |  |
| #19 | (prepubescen*):ti,ab,kw | 103 | |  |
| #20 | (pubescent*):ti,ab,kw | 58 | |  |
| #21 | (adolescent*):ti,ab,kw | 138504 | |  |
| #22 | (juvenil*):ti,ab,kw | 4007 | |  |
| #23 | (underage*):ti,ab,kw | 284 | |  |
| #24 | (preteen* or pre-teen*):ti,ab,kw | 61 | |  |
| #25 | (teen or teens):ti,ab,kw | 1666 | |  |
| #26 | (teenage*):ti,ab,kw | 1383 | |  |
| #27 | (youth or youths):ti,ab,kw | 7845 | |  |
| #28 | (young NEXT person*):ti,ab,kw | 288 | |  |
| #29 | (young NEXT people*):ti,ab,kw | 2509 | |  |
| #30 | (kid or kids):ti,ab,kw | 1177 | |  |
| #31 | (transition NEAR/4 adult*):ti,ab,kw | 260 | |  |
| #32 | (emerging NEXT adult*):ti,ab,kw | 181 | |  |
| #33 | (young NEXT adult*):ti,ab,kw | 86059 | |  |
| #34 | (parent?):ti,ab,kw | 31174 | |  |
| #35 | (mother?):ti,ab,kw | 19450 | |  |
| #36 | (father?):ti,ab,kw | 1517 | |  |
| #37 | (guardian?):ti,ab,kw | 4308 | |  |
| #38 | (teacher?):ti,ab,kw | 5855 | |  |
| #39 | ((5 or 6 or 7 or 8 or 9 or 10 or 11 or 12 or 13 or 14 or 15 or 16 or 17 or 19) NEAR/1 (year* NEXT old or year* NEXT age)):ti,ab,kw | 13105 | |  |
| #40 | ((five or six or seven or eight or nine or ten or eleven or twelve or thirteen or fourteen or fifteen or sixteen or seventeen or eighteen) NEAR/1 (year* NEXT old or year* NEXT age)):ti,ab,kw | 665 | |  |
| #41 | ((primary or secondary or year) NEAR/1 (1 or 2 or 3 or 4 or 5 or 6 or 7 or 8 or 9 or 10 or 11 or 12 or 13)):ti,ab,kw | 103529 | |  |
| #42 | (grade? NEAR/1 (first or second or third or fourth or fifth or sixth or seventh or eighth or ninth or tenth or eleventh or twelfth)):ti,ab,kw | 1845 | |  |
| #43 | #1 or #2 or #3 or #4 or #5 or #6 or #7 or #8 or #9 or #10 or #11 or #12 or #13 or #18 or #19 or #20 or #21 or #22 or #23 or #24 or #25 or #26 or #27 or #28 or #29 or #30 or #31 or #32 or #33 or #34 or #35 or #36 or #37 or #38 or #39 or #40 or #41 or #42 | 438212 | |  |
| #44 | MeSH descriptor: [Schools] this term only and with qualifier(s): [organization & administration - OG] | 98 | |  |
| #45 | MeSH descriptor: [School Health Services] this term only and with qualifier(s): [organization & administration - OG] | 471 | |  |
| #46 | (school? NEAR/5 (based or level or wide or led or ethos or environment or organi?ation* or climate or toolkit? or approach* or (action NEXT group?) or practice? or whole or health* or leader* or cultur* or manag* or governance or system? or communit*)):ti,ab,kw | 10293 | |  |
| #47 | (schoolwide):ti,ab,kw | 106 | |  |
| #48 | (restor* NEXT justice):ti,ab,kw | 14 | |  |
| #49 | MeSH descriptor: [Schools] this term only | 2098 | |  |
| #50 | (school?):ti,ab,kw | 35480 | |  |
| #51 | #49 or #50 | 35480 | |  |
| #52 | (peer-to-peer):ti,ab,kw | 178 | |  |
| #53 | (peer NEAR/2 (led or leader* or participat* or mediat* or helper? or resistan* or wide or action? or influen*)):ti,ab,kw | 1170 | |  |
| #54 | (relationship* NEAR/2 (led or leader* or participat* or mediat* or helper? or resistan* or wide or action? or influen*)):ti,ab,kw | 1005 | |  |
| #55 | MeSH descriptor: [Community Networks] this term only | 170 | |  |
| #56 | MeSH descriptor: [Community Participation] this term only | 292 | |  |
| #57 | ((community or communities) NEAR/2 (led or leader* or participat* or mediat* or helper? or resistan* or wide or action? or influen* or relationship*)):ti,ab,kw | 2513 | |  |
| #58 | (pupil? NEAR/2 (led or leader* or participat* or mediat* or helper? or resistan* or wide or action? or influen* or relationship*)):ti,ab,kw | 66 | |  |
| #59 | #58 NOT #15 | 57 | |  |
| #60 | ((child or children) NEAR/2 (led or leader* or participat* or mediat* or helper? or resistan* or wide or action? or influen*)):ti,ab,kw | 3030 | |  |
| #61 | ((young people or young person) NEAR/2 (led or leader* or participat* or mediat* or helper? or resistan* or wide or action? or influen*)):ti,ab,kw | 1618 | |  |
| #62 | (teen* NEAR/2 (led or leader* or participat* or mediat* or helper? or resistan* or wide or action? or influen*)):ti,ab,kw | 79 | |  |
| #63 | (adolescent* NEAR/2 (led or leader* or participat* or mediat* or helper? or resistan* or wide or action? or influen*)):ti,ab,kw | 685 | |  |
| #64 | (youth? NEAR/2 (led or leader* or participat* or mediat* or helper? or resistan* or wide or action? or influen*)):ti,ab,kw | 281 | |  |
| #65 | (student? NEAR/2 (led or leader* or participat* or mediat* or helper? or resistan* or wide or action? or influen*)):ti,ab,kw | 1603 | |  |
| #66 | #65 NOT #16 | 1397 | |  |
| #67 | (teach* NEAR/2 (led or leader* or participat* or mediat* or helper? or resistan* or wide or action? or influen* or relationship*)):ti,ab,kw | 551 | |  |
| #68 | (curricul* NEAR/2 (led or leader* or participat* or mediat* or helper? or resistan* or wide or action? or influen* or relationship*)):ti,ab,kw | 88 | |  |
| #69 | (administrat* NEAR/2 (led or leader* or participat* or mediat* or helper? or resistan* or wide or action? or influen* or relationship*)):ti,ab,kw | 2116 | |  |
| #70 | ((personnel or staff) NEAR/2 (led or leader* or participat* or mediat* or helper? or resistan* or wide or action? or influen* or relationship*)):ti,ab,kw | 519 | |  |
| #71 | (volunteer* NEAR/2 (led or leader* or participat* or mediat* or helper? or resistan* or wide or action? or influen* or relationship*)):ti,ab,kw | 2751 | |  |
| #72 | ((advocacy or advocate) NEAR/2 (led or leader* or participat* or mediat* or helper? or resistan* or wide or action? or influen* or relationship*)):ti,ab,kw | 27 | |  |
| #73 | (parent* NEAR/2 (led or leader* or participat* or mediat* or helper? or resistan* or wide or action? or influen* or relationship*)):ti,ab,kw | 2118 | |  |
| #74 | MeSH descriptor: [Social Environment] this term only | 892 | |  |
| #75 | (environment* NEAR/2 (led or leader* or participat* or mediat* or helper? or resistan* or wide or action? or influen* or relationship*)):ti,ab,kw | 601 | |  |
| #76 | (socio-ecolog* NEAR/2 (led or leader* or participat* or mediat* or helper? or resistan* or wide or action? or influen* or relationship*)):ti,ab,kw | 2 | |  |
| #77 | MeSH descriptor: [Social Support] this term only | 3351 | |  |
| #78 | MeSH descriptor: [Social Learning] this term only | 31 | |  |
| #79 | (learn* NEAR/2 (led or leader* or participat* or mediat* or helper? or resistan* or wide or action? or influen* or relationship*)):ti,ab,kw | 597 | |  |
| #80 | ((policy or policies) NEAR/2 (led or leader* or participat* or mediat* or helper? or resistan* or wide or action? or influen* or relationship*)):ti,ab,kw | 173 | |  |
| #81 | #52 or #53 or #54 or #55 or #56 or #57 or #59 or #60 or #61 or #62 or #63 or #64 or #66 or #67 or #68 or #69 or #70 or #71 or #72 or #73 or #74 or #75 or #76 or #77 or #78 or #79 or #80 | 23853 | |  |
| #82 | #51 and #81 | 3793 | |  |
| #83 | #44 or #45 or #46 or #47 or #48 or #82 | 12303 | |  |
| #84 | MeSH descriptor: [Smoking Prevention] this term only | 1611 | |  |
| #85 | MeSH descriptor: [Tobacco Smoking] explode all trees and with qualifier(s): [prevention & control - PC] | 33 | |  |
| #86 | MeSH descriptor: [Vaping] this term only and with qualifier(s): [prevention & control - PC] | 2 | |  |
| #87 | MeSH descriptor: [Marijuana Smoking] this term only and with qualifier(s): [prevention & control - PC] | 67 | |  |
| #88 | MeSH descriptor: [Smoking Cessation] this term only | 4180 | |  |
| #89 | MeSH descriptor: [Tobacco Use Cessation] this term only | 110 | |  |
| #90 | MeSH descriptor: [Smoking Reduction] this term only | 27 | |  |
| #91 | ((smoking or smoked or smoke or smoker) NEAR/5 (reduc* or decreas* or increas* or lower* or fewer OR improv* or enhance* or extend* or develop* or prevalen* or daily or weekly or current or rate or rates or behavio?r* or knowledge or prevent* or attitude* or avoid* or perceive* or percept* or promot* or program* or change*)):ti,ab,kw | 13706 | |  |
| #92 | (tobacco NEAR/5 (reduc* or decreas* or increas* or lower* or fewer OR improv* or enhance* or extend* or develop* or prevalen* or daily or weekly or current or rate or rates or behavio?r* or knowledge or prevent* or attitude* or avoid* or perceive* or percept* or promot* or program* or change*)):ti,ab,kw | 3139 | |  |
| #93 | (cigarette? NEAR/5 (reduc* or decreas* or increas* or lower* or fewer OR improv* or enhance* or extend* or develop* or prevalen* or daily or weekly or current or rate or rates or behavio?r* or knowledge or prevent* or attitude* or avoid* or perceive* or percept* or promot* or program* or change*)):ti,ab,kw | 3392 | |  |
| #94 | (marijuana NEAR/5 (reduc* or decreas* or increas* or lower* or fewer OR improv* or enhance* or extend* or develop* or prevalen* or daily or weekly or current or rate or rates or behavio?r* or knowledge or prevent* or attitude* or avoid* or perceive* or percept* or promot* or program* or change*)):ti,ab,kw | 650 | |  |
| #95 | (cannabis NEAR/5 (reduc* or decreas* or increas* or lower* or fewer OR improv* or enhance* or extend* or develop* or prevalen* or daily or weekly or current or rate or rates or behavio?r* or knowledge or prevent* or attitude* or avoid* or perceive* or percept* or promot* or program* or change*)):ti,ab,kw | 914 | |  |
| #96 | (solvent? NEAR/5 (reduc* or decreas* or increas* or lower* or fewer OR improv* or enhance* or extend* or develop* or prevalen* or daily or weekly or current or rate or rates or behavio?r* or knowledge or prevent* or attitude* or avoid* or perceive* or percept* or promot* or program* or change*)):ti,ab,kw | 151 | |  |
| #97 | MeSH descriptor: [Substance-Related Disorders] explode all trees and with qualifier(s): [prevention & control - PC] | 1601 | |  |
| #98 | MeSH descriptor: [Alcohol Drinking] this term only and with qualifier(s): [prevention & control - PC] | 818 | |  |
| #99 | MeSH descriptor: [Binge Drinking] this term only and with qualifier(s): [prevention & control - PC] | 78 | |  |
| #100 | MeSH descriptor: [Underage Drinking] this term only and with qualifier(s): [prevention & control - PC] | 45 | |  |
| #101 | (alcohol NEAR/5 (reduc* or decreas* or increas* or lower* or fewer OR improv* or enhance* or extend* or develop* or prevalen* or daily or weekly or current or rate or rates or behavio?r* or knowledge or prevent* or attitude* or avoid* or perceive* or percept* or promot* or program* or change*)):ti,ab,kw | 9858 | |  |
| #102 | ((binge NEXT drink*) NEAR/5 (reduc* or decreas* or increas* or lower* or fewer OR improv* or enhance* or extend* or develop* or prevalen* or daily or weekly or current or rate or rates or behavio?r* or knowledge or prevent* or attitude* or avoid* or perceive* or percept* or promot* or program* or change*)):ti,ab,kw | 379 | |  |
| #103 | ((underage NEXT drink*) NEAR/5 (reduc* or decreas* or increas* or lower* or fewer OR improv* or enhance* or extend* or develop* or prevalen* or daily or weekly or current or rate or rates or behavio?r* or knowledge or prevent* or attitude* or avoid* or perceive* or percept* or promot* or program* or change*)):ti,ab,kw | 78 | |  |
| #104 | (substance? NEAR/5 (reduc* or decreas* or increas* or lower* or fewer OR improv* or enhance* or extend* or develop* or prevalen* or daily or weekly or current or rate or rates or behavio?r* or knowledge or prevent* or attitude* or avoid* or perceive* or percept* or promot* or program* or change*)):ti,ab,kw | 5304 | |  |
| #105 | (drug? NEAR/5 (reduc* or decreas* or increas* or lower* or fewer OR improv* or enhance* or extend* or develop* or prevalen* or daily or weekly or current or rate or rates or behavio?r* or knowledge or prevent* or attitude* or avoid* or perceive* or percept* or promot* or program* or change*)):ti,ab,kw | 101037 | |  |
| #106 | MeSH descriptor: [Academic Performance] explode all trees | 87 | |  |
| #107 | ((education* NEXT attain*) NEAR/5 (reduc* or decreas* or increas* or lower* or fewer OR improv* or enhance* or extend* or develop* or prevalen* or daily or weekly or current or rate or rates or behavio?r* or knowledge or prevent* or attitude* or avoid* or perceive* or percept* or promot* or program* or change*)):ti,ab,kw | 161 | |  |
| #108 | ((academic NEXT attain*) NEAR/5 (reduc* or decreas* or increas* or lower* or fewer OR improv* or enhance* or extend* or develop* or prevalen* or daily or weekly or current or rate or rates or behavio?r* or knowledge or prevent* or attitude* or avoid* or perceive* or percept* or promot* or program* or change*)):ti,ab,kw | 6 | |  |
| #109 | ((student* NEXT attain*) NEAR/5 (reduc* or decreas* or increas* or lower* or fewer OR improv* or enhance* or extend* or develop* or prevalen* or daily or weekly or current or rate or rates or behavio?r* or knowledge or prevent* or attitude* or avoid* or perceive* or percept* or promot* or program* or change*)):ti,ab,kw | 4 | |  |
| #110 | ((education* NEXT achieve*) NEAR/5 (reduc* or decreas* or increas* or lower* or fewer OR improv* or enhance* or extend* or develop* or prevalen* or daily or weekly or current or rate or rates or behavio?r* or knowledge or prevent* or attitude* or avoid* or perceive* or percept* or promot* or program* or change*)):ti,ab,kw | 46 | |  |
| #111 | ((academic NEXT achieve*) NEAR/5 (reduc* or decreas* or increas* or lower* or fewer OR improv* or enhance* or extend* or develop* or prevalen* or daily or weekly or current or rate or rates or behavio?r* or knowledge or prevent* or attitude* or avoid* or perceive* or percept* or promot* or program* or change*)):ti,ab,kw | 267 | |  |
| #112 | ((student* NEXT achieve*) NEAR/5 (reduc* or decreas* or increas* or lower* or fewer OR improv* or enhance* or extend* or develop* or prevalen* or daily or weekly or current or rate or rates or behavio?r* or knowledge or prevent* or attitude* or avoid* or perceive* or percept* or promot* or program* or change*)):ti,ab,kw | 22 | |  |
| #113 | ((education* NEXT perform*) NEAR/5 (reduc* or decreas* or increas* or lower* or fewer OR improv* or enhance* or extend* or develop* or prevalen* or daily or weekly or current or rate or rates or behavio?r* or knowledge or prevent* or attitude* or avoid* or perceive* or percept* or promot* or program* or change*)):ti,ab,kw | 28 | |  |
| #114 | ((academic NEXT perform*) NEAR/5 (reduc* or decreas* or increas* or lower* or fewer OR improv* or enhance* or extend* or develop* or prevalen* or daily or weekly or current or rate or rates or behavio?r* or knowledge or prevent* or attitude* or avoid* or perceive* or percept* or promot* or program* or change*)):ti,ab,kw | 339 | |  |
| #115 | ((student* NEXT perform*) NEAR/5 (reduc* or decreas* or increas* or lower* or fewer OR improv* or enhance* or extend* or develop* or prevalen* or daily or weekly or current or rate or rates or behavio?r* or knowledge or prevent* or attitude* or avoid* or perceive* or percept* or promot* or program* or change*)):ti,ab,kw | 146 | |  |
| #116 | ((education* NEXT outcome?) NEAR/5 (reduc* or decreas* or increas* or lower* or fewer OR improv* or enhance* or extend* or develop* or prevalen* or daily or weekly or current or rate or rates or behavio?r* or knowledge or prevent* or attitude* or avoid* or perceive* or percept* or promot* or program* or change*)):ti,ab,kw | 154 | |  |
| #117 | ((academic NEXT outcome?) NEAR/5 (reduc* or decreas* or increas* or lower* or fewer OR improv* or enhance* or extend* or develop* or prevalen* or daily or weekly or current or rate or rates or behavio?r* or knowledge or prevent* or attitude* or avoid* or perceive* or percept* or promot* or program* or change*)):ti,ab,kw | 67 | |  |
| #118 | ((student* NEXT outcome?) NEAR/5 (reduc* or decreas* or increas* or lower* or fewer OR improv* or enhance* or extend* or develop* or prevalen* or daily or weekly or current or rate or rates or behavio?r* or knowledge or prevent* or attitude* or avoid* or perceive* or percept* or promot* or program* or change*)):ti,ab,kw | 54 | |  |
| #119 | ((education* NEXT commitment) NEAR/5 (reduc* or decreas* or increas* or lower* or fewer OR improv* or enhance* or extend* or develop* or prevalen* or daily or weekly or current or rate or rates or behavio?r* or knowledge or prevent* or attitude* or avoid* or perceive* or percept* or promot* or program* or change*)):ti,ab,kw | 1 | |  |
| #120 | ((academic NEXT commitment) NEAR/5 (reduc* or decreas* or increas* or lower* or fewer OR improv* or enhance* or extend* or develop* or prevalen* or daily or weekly or current or rate or rates or behavio?r* or knowledge or prevent* or attitude* or avoid* or perceive* or percept* or promot* or program* or change*)):ti,ab,kw | 0 | |  |
| #121 | ((student* NEXT commitment) NEAR/5 (reduc* or decreas* or increas* or lower* or fewer OR improv* or enhance* or extend* or develop* or prevalen* or daily or weekly or current or rate or rates or behavio?r* or knowledge or prevent* or attitude* or avoid* or perceive* or percept* or promot* or program* or change*)):ti,ab,kw | 1 | |  |
| #122 | ((education* NEXT engage*) NEAR/5 (reduc* or decreas* or increas* or lower* or fewer OR improv* or enhance* or extend* or develop* or prevalen* or daily or weekly or current or rate or rates or behavio?r* or knowledge or prevent* or attitude* or avoid* or perceive* or percept* or promot* or program* or change*)):ti,ab,kw | 6 | |  |
| #123 | ((academic NEXT engage*) NEAR/5 (reduc* or decreas* or increas* or lower* or fewer OR improv* or enhance* or extend* or develop* or prevalen* or daily or weekly or current or rate or rates or behavio?r* or knowledge or prevent* or attitude* or avoid* or perceive* or percept* or promot* or program* or change*)):ti,ab,kw | 20 | |  |
| #124 | ((student* NEXT engage*) NEAR/5 (reduc* or decreas* or increas* or lower* or fewer OR improv* or enhance* or extend* or develop* or prevalen* or daily or weekly or current or rate or rates or behavio?r* or knowledge or prevent* or attitude* or avoid* or perceive* or percept* or promot* or program* or change*)):ti,ab,kw | 40 | |  |
| #125 | (dropout NEAR/5 (reduc* or decreas* or increas* or lower* or fewer OR improv* or enhance* or extend* or develop* or prevalen* or daily or weekly or current or rate or rates or behavio?r* or knowledge or prevent* or attitude* or avoid* or perceive* or percept* or promot* or program* or change*)):ti,ab,kw | 3963 | |  |
| #126 | (absentee* NEAR/5 (reduc* or decreas* or increas* or lower* or fewer OR improv* or enhance* or extend* or develop* or prevalen* or daily or weekly or current or rate or rates or behavio?r* or knowledge or prevent* or attitude* or avoid* or perceive* or percept* or promot* or program* or change*)):ti,ab,kw | 530 | |  |
| #127 | MeSH descriptor: [Violence] this term only and with qualifier(s): [prevention & control - PC] | 227 | |  |
| #128 | MeSH descriptor: [Aggression] this term only | 1229 | |  |
| #129 | MeSH descriptor: [Physical Abuse] this term only and with qualifier(s): [prevention & control - PC] | 10 | |  |
| #130 | MeSH descriptor: [Gender-Based Violence] this term only and with qualifier(s): [prevention & control - PC] | 6 | |  |
| #131 | MeSH descriptor: [Gun Violence] this term only and with qualifier(s): [prevention & control - PC] | 1 | |  |
| #132 | MeSH descriptor: [Bullying] explode all trees and with qualifier(s): [prevention & control - PC] | 46 | |  |
| #133 | ((violent or violence) NEAR/5 (reduc* or decreas* or increas* or lower* or fewer OR improv* or enhance* or extend* or develop* or prevalen* or daily or weekly or current or rate or rates or behavio?r* or knowledge or prevent* or attitude* or avoid* or perceive* or percept* or promot* or program* or change*)):ti,ab,kw | 1847 | |  |
| #134 | ((aggression or aggressive) NEAR/5 (reduc* or decreas* or increas* or lower* or fewer OR improv* or enhance* or extend* or develop* or prevalen* or daily or weekly or current or rate or rates or behavio?r* or knowledge or prevent* or attitude* or avoid* or perceive* or percept* or promot* or program* or change*)):ti,ab,kw | 3531 | |  |
| #135 | ((hostile or hostility) NEAR/5 (reduc* or decreas* or increas* or lower* or fewer OR improv* or enhance* or extend* or develop* or prevalen* or daily or weekly or current or rate or rates or behavio?r* or knowledge or prevent* or attitude* or avoid* or perceive* or percept* or promot* or program* or change*)):ti,ab,kw | 457 | |  |
| #136 | (assault* NEAR/5 (reduc* or decreas* or increas* or lower* or fewer OR improv* or enhance* or extend* or develop* or prevalen* or daily or weekly or current or rate or rates or behavio?r* or knowledge or prevent* or attitude* or avoid* or perceive* or percept* or promot* or program* or change*)):ti,ab,kw | 231 | |  |
| #137 | (crime NEAR/5 (reduc* or decreas* or increas* or lower* or fewer OR improv* or enhance* or extend* or develop* or prevalen* or daily or weekly or current or rate or rates or behavio?r* or knowledge or prevent* or attitude* or avoid* or perceive* or percept* or promot* or program* or change*)):ti,ab,kw | 234 | |  |
| #138 | ((abuse? or abusive) NEAR/5 (reduc* or decreas* or increas* or lower* or fewer OR improv* or enhance* or extend* or develop* or prevalen* or daily or weekly or current or rate or rates or behavio?r* or knowledge or prevent* or attitude* or avoid* or perceive* or percept* or promot* or program* or change*)):ti,ab,kw | 3425 | |  |
| #139 | (misbehav* NEAR/5 (reduc* or decreas* or increas* or lower* or fewer OR improv* or enhance* or extend* or develop* or prevalen* or daily or weekly or current or rate or rates or behavio?r* or knowledge or prevent* or attitude* or avoid* or perceive* or percept* or promot* or program* or change*)):ti,ab,kw | 23 | |  |
| #140 | (threat? NEAR/5 (reduc* or decreas* or increas* or lower* or fewer OR improv* or enhance* or extend* or develop* or prevalen* or daily or weekly or current or rate or rates or behavio?r* or knowledge or prevent* or attitude* or avoid* or perceive* or percept* or promot* or program* or change*)):ti,ab,kw | 888 | |  |
| #141 | (conduct NEAR/5 (reduc* or decreas* or increas* or lower* or fewer OR improv* or enhance* or extend* or develop* or prevalen* or daily or weekly or current or rate or rates or behavio?r* or knowledge or prevent* or attitude* or avoid* or perceive* or percept* or promot* or program* or change*)):ti,ab,kw | 1677 | |  |
| #142 | (delinquen* NEAR/5 (reduc* or decreas* or increas* or lower* or fewer OR improv* or enhance* or extend* or develop* or prevalen* or daily or weekly or current or rate or rates or behavio?r* or knowledge or prevent* or attitude* or avoid* or perceive* or percept* or promot* or program* or change*)):ti,ab,kw | 371 | |  |
| #143 | ((disrupt* NEXT behavio?r*) NEAR/5 (reduc* or decreas* or increas* or lower* or fewer OR improv* or enhance* or extend* or develop* or prevalen* or daily or weekly or current or rate or rates or behavio?r* or knowledge or prevent* or attitude* or avoid* or perceive* or percept* or promot* or program* or change*)):ti,ab,kw | 945 | |  |
| #144 | ((problem* NEXT behavio?r*) NEAR/5 (reduc* or decreas* or increas* or lower* or fewer OR improv* or enhance* or extend* or develop* or prevalen* or daily or weekly or current or rate or rates or behavio?r* or knowledge or prevent* or attitude* or avoid* or perceive* or percept* or promot* or program* or change*)):ti,ab,kw | 1463 | |  |
| #145 | ((volatile NEXT behavio?r*) NEAR/5 (reduc* or decreas* or increas* or lower* or fewer OR improv* or enhance* or extend* or develop* or prevalen* or daily or weekly or current or rate or rates or behavio?r* or knowledge or prevent* or attitude* or avoid* or perceive* or percept* or promot* or program* or change*)):ti,ab,kw | 0 | |  |
| #146 | ((antisocial or anti-social) NEAR/5 (reduc* or decreas* or increas* or lower* or fewer OR improv* or enhance* or extend* or develop* or prevalen* or daily or weekly or current or rate or rates or behavio?r* or knowledge or prevent* or attitude* or avoid* or perceive* or percept* or promot* or program* or change*)):ti,ab,kw | 469 | |  |
| #147 | (perpetrat* NEAR/5 (reduc* or decreas* or increas* or lower* or fewer OR improv* or enhance* or extend* or develop* or prevalen* or daily or weekly or current or rate or rates or behavio?r* or knowledge or prevent* or attitude* or avoid* or perceive* or percept* or promot* or program* or change*)):ti,ab,kw | 202 | |  |
| #148 | (bully* NEAR/5 (reduc* or decreas* or increas* or lower* or fewer OR improv* or enhance* or extend* or develop* or prevalen* or daily or weekly or current or rate or rates or behavio?r* or knowledge or prevent* or attitude* or avoid* or perceive* or percept* or promot* or program* or change*)):ti,ab,kw | 230 | |  |
| #149 | (victimi* NEAR/5 (reduc* or decreas* or increas* or lower* or fewer OR improv* or enhance* or extend* or develop* or prevalen* or daily or weekly or current or rate or rates or behavio?r* or knowledge or prevent* or attitude* or avoid* or perceive* or percept* or promot* or program* or change*)):ti,ab,kw | 275 | |  |
| #150 | (cyberbully* NEAR/5 (reduc* or decreas* or increas* or lower* or fewer OR improv* or enhance* or extend* or develop* or prevalen* or daily or weekly or current or rate or rates or behavio?r* or knowledge or prevent* or attitude* or avoid* or perceive* or percept* or promot* or program* or change*)):ti,ab,kw | 38 | |  |
| #151 | (conflict* NEAR/5 (reduc* or decreas* or increas* or lower* or fewer OR improv* or enhance* or extend* or develop* or prevalen* or daily or weekly or current or rate or rates or behavio?r* or knowledge or prevent* or attitude* or avoid* or perceive* or percept* or promot* or program* or change*)):ti,ab,kw | 1839 | |  |
| #152 | ((positive NEXT behavio?r*) NEAR/5 (reduc* or decreas* or increas* or lower* or fewer OR improv* or enhance* or extend* or develop* or prevalen* or daily or weekly or current or rate or rates or behavio?r* or knowledge or prevent* or attitude* or avoid* or perceive* or percept* or promot* or program* or change*)):ti,ab,kw | 383 | |  |
| #153 | ((improv* NEXT behavio?r*) NEAR/5 (reduc* or decreas* or increas* or lower* or fewer OR improv* or enhance* or extend* or develop* or prevalen* or daily or weekly or current or rate or rates or behavio?r* or knowledge or prevent* or attitude* or avoid* or perceive* or percept* or promot* or program* or change*)):ti,ab,kw | 484 | |  |
| #154 | ((social NEXT behavio?r*) NEAR/5 (reduc* or decreas* or increas* or lower* or fewer OR improv* or enhance* or extend* or develop* or prevalen* or daily or weekly or current or rate or rates or behavio?r* or knowledge or prevent* or attitude* or avoid* or perceive* or percept* or promot* or program* or change*)):ti,ab,kw | 3468 | |  |
| #155 | ((social-emotion* or socio-emotion*) NEAR/5 (reduc* or decreas* or increas* or lower* or fewer OR improv* or enhance* or extend* or develop* or prevalen* or daily or weekly or current or rate or rates or behavio?r* or knowledge or prevent* or attitude* or avoid* or perceive* or percept* or promot* or program* or change*)):ti,ab,kw | 504 | |  |
| #156 | ((social-charact*) NEAR/5 (reduc* or decreas* or increas* or lower* or fewer OR improv* or enhance* or extend* or develop* or prevalen* or daily or weekly or current or rate or rates or behavio?r* or knowledge or prevent* or attitude* or avoid* or perceive* or percept* or promot* or program* or change*)):ti,ab,kw | 9 | |  |
| #157 | ((social-inclusion) NEAR/5 (reduc* or decreas* or increas* or lower* or fewer OR improv* or enhance* or extend* or develop* or prevalen* or daily or weekly or current or rate or rates or behavio?r* or knowledge or prevent* or attitude* or avoid* or perceive* or percept* or promot* or program* or change*)):ti,ab,kw | 35 | |  |
| #158 | ((social NEXT develop*) NEAR/5 (reduc* or decreas* or increas* or lower* or fewer OR improv* or enhance* or extend* or develop* or prevalen* or daily or weekly or current or rate or rates or behavio?r* or knowledge or prevent* or attitude* or avoid* or perceive* or percept* or promot* or program* or change*)):ti,ab,kw | 199 | |  |
| #159 | ((social NEXT competen*) NEAR/5 (reduc* or decreas* or increas* or lower* or fewer OR improv* or enhance* or extend* or develop* or prevalen* or daily or weekly or current or rate or rates or behavio?r* or knowledge or prevent* or attitude* or avoid* or perceive* or percept* or promot* or program* or change*)):ti,ab,kw | 268 | |  |
| #160 | ((emotion* NEXT develop*) NEAR/5 (reduc* or decreas* or increas* or lower* or fewer OR improv* or enhance* or extend* or develop* or prevalen* or daily or weekly or current or rate or rates or behavio?r* or knowledge or prevent* or attitude* or avoid* or perceive* or percept* or promot* or program* or change*)):ti,ab,kw | 243 | |  |
| #161 | (conduct NEAR/5 (reduc* or decreas* or increas* or lower* or fewer OR improv* or enhance* or extend* or develop* or prevalen* or daily or weekly or current or rate or rates or behavio?r* or knowledge or prevent* or attitude* or avoid* or perceive* or percept* or promot* or program* or change*)):ti,ab,kw | 1677 | |  |
| #162 | (respect NEAR/5 (reduc* or decreas* or increas* or lower* or fewer OR improv* or enhance* or extend* or develop* or prevalen* or daily or weekly or current or rate or rates or behavio?r* or knowledge or prevent* or attitude* or avoid* or perceive* or percept* or promot* or program* or change*)):ti,ab,kw | 4814 | |  |
| #163 | ((safety or safe) NEAR/5 (reduc* or decreas* or increas* or lower* or fewer OR improv* or enhance* or extend* or develop* or prevalen* or daily or weekly or current or rate or rates or behavio?r* or knowledge or prevent* or attitude* or avoid* or perceive* or percept* or promot* or program* or change*)):ti,ab,kw | 38099 | |  |
| #164 | #84 or #85 or #86 or #87 or #88 or #89 or #90 or #91 or #92 or #93 or #94 or #95 or #96 or #97 or #99 or #100 or #101 or #102 or #103 or #104 or #105 | 127654 | |  |
| #165 | #106 or #107 or #108 or #109 or #110 or #111 or #112 or #113 or #114 or #115 or #116 or #117 or #118 or #119 or #120 or #121 or #122 or #123 or #124 or #125 or #126 | 5805 | |  |
| #166 | #127 or #128 or #129 or #130 or #131 or #132 or #133 or #134 or #135 or #136 or #137 or #138 or #139 or #140 or #141 or #142 or #143 or #144 or #145 or #146 or #147 or #148 or #149 or #150 or #151 or #152 or #153 or #154 or #155 or #156 or #157 or #158 or #159 or #160 or #161 or #162 or #163 | 62061 | |  |
| #167 | MeSH descriptor: [Program Evaluation] explode all trees | 6350 | |  |
| #168 | MeSH descriptor: [Evaluation Study] this term only | 1 | |  |
| #169 | MeSH descriptor: [Validation Study] this term only | 0 | |  |
| #170 | MeSH descriptor: [Clinical Trial] explode all trees | 141 | |  |
| #171 | (prevent*):ti,ab,kw | 237179 | |  |
| #172 | (program*):ti,ab,kw | 130402 | |  |
| #173 | (intervent*):ti,ab,kw | 434511 | |  |
| #174 | (restorative NEXT approach*):ti,ab,kw | 27 | |  |
| #175 | (control NEXT school?):ti,ab,kw | 813 | |  |
| #176 | (trial or trials):ti,ab,kw | 929113 | |  |
| #177 | #167 or #168 or #169 or #170 or #171 or #172 or #173 or #174 or #175 or #176 | 1192739 | |  |
| #178 | #43 and #83 and #164 and #177 | 1641 | |  |
| #179 | #43 and #83 and #165 and #177 | 432 | |  |
| #180 | #43 and #83 and #166 and #177 | 1660 | |  |

## EPPI-Centre database of health promotion research (Bibliomap)

| Database name | Database of health promotion research (Bibliomap) |
| --- | --- |
| Database platform | EPPI-Centre |
| Dates of database coverage | Complete database to date searched. |
| Date searched | 13/05/2021 |
| Searched by | JF |
| Number of results | 0 |
| EndNote import order | N/A |
| Number of results once duplicates removed | 0 |
| Search strategy notes | None |

Freetext: “school OR schools OR schoolwide”

## EPPI-Centre Database of promoting health effectiveness reviews (DoPHER)

| Database name | Database of promoting health effectiveness reviews (DoPHER) |
| --- | --- |
| Database platform | EPPI-Centre |
| Dates of database coverage | Complete database to date searched. |
| Date searched | 13/05/2020 |
| Searched by | JF |
| Number of results | 0 |
| EndNote import order | N/A |
| Number of results once duplicates removed | 0 |
| Search strategy notes | None |

Freetext: “school OR schools OR schoolwide”

## OvidSP Econlit

| Database name | Econlit |
| --- | --- |
| Database platform | OvidSP |
| Dates of database coverage | 1886 to April 29, 2021 |
| Date searched | 11/05/2021 |
| Searched by | JF |
| Number of results | 268 |
| EndNote import order | 4 |
| Number of results once duplicates removed | 33 |
| Search strategy notes | Search lines ending in .ti,ab,kw. search in the title, abstract and author keywords only. Search lines ending in .jx. search in the journal title only. or/*x-y* combines search sets in the range *x-y* with Boolean operator OR. * is used for truncation of words. # is used for a compulsory wildcard. ? is used for an optional wildcard. adj*n* searches for terms within *n* words or each other. Words in square brackets [ ] are comments and are not included in the search terms. |

1	child*.ti,ab,kw. (29289_

2	boy?.ti,ab,kw. (1919)

3	girl?.ti,ab,kw. (2461)

4	(schoolage or (school adj1 age)).ti,ab,kw. (401)

5	schoolchild*.ti,ab,kw. (95)

6	youngster?.ti,ab,kw. (99)

7	minor?.ti,ab,kw. (3422)

8	((pupil* or student*) not ((pupil adj5 eye?) or (student* adj medic*))).ti,ab,kw. (22999)

9	prepubescen*.ti,ab,kw. (3)

10	pubescent*.ti,ab,kw. (3)

11	adolescent*.ti,ab,kw. (2058)

12	juvenil*.ti,ab,kw. (408)

13	underage*.ti,ab,kw. (78)

14	(preteen* or pre-teen*).ti,ab,kw. (13)

15	(teen or teens).ti,ab,kw. (607)

16	teenage*.ti,ab,kw. (1091)

17	(youth or youths).ti,ab,kw. (6273)

18	young person*.ti,ab,kw. (100)

19	young people*.ti,ab,kw. (2047)

20	(kid or kids).ti,ab,kw. (362)

21	(transition adj4 adult*).ti,ab,kw. (175)

22	emerging adult*.ti,ab,kw. (23)

23	young adult.ti,ab,kw. (359)

24	parent?.ti,ab,kw. (10082)

25	mother?.ti,ab,kw. (5638)

26	father?.ti,ab,kw. (2448)

27	guardian?.ti,ab,kw. (206)

28	teacher?.ti,ab,kw. (4858)

29	child*.jx. (0)

30	adolescen*.jx. (0)

31	youth*.jx. (0)

32	school*.jx. (2527)

33	(("5" or "6" or "7" or "8" or "9" or "10" or "11" or "12" or "13" or "14" or "15" or "16" or "17" or "18") adj (year* old or year* of age)).ti,ab,kw. (636)

34	((five or six or seven or eight or nine or ten or eleven or twelve or thirteen or fourteen or fifteen or sixteen or seventeen or eighteen) adj (year* old or year* of age)).ti,ab,kw. (229)

35	(age* adj ("5" or "6" or "7" or "8" or "9" or "10" or "11" or "12" or "13" or "14" or "15" or "16" or "17" or "18") adj year*).ti,ab,kw. (72)

36	(age* adj (five or six or seven or eight or nine or ten or eleven or twelve or thirteen or fourteen or fifteen or sixteen or seventeen or eighteen) adj year*).ti,ab,kw.	18

37	((primary or secondary or year) adj1 ("1" or "2" or "3" or "4" or "5" or "6" or "7" or "8" or "9" or "10" or "11" or "12" or "13")).ti,ab,kw. (3855)

38	(grade? adj1 (first or second or third or fourth or fifth or sixth or seventh or eighth or ninth or tenth or eleventh or twelfth)).ti,ab,kw. (495)

39	or/1-38 (75769)

40	(school? adj5 (based or level or wide or led or ethos or environment or organi?ation* or climate or toolkit? or approach* or action group? or practice? or whole or health* or leader* or cultur* or manag* or governance or system? or communit*)).ti,ab. (6231)

41	schoolwide.ti,ab. (7)

42	restor* justice.ti,ab. (40)

43	school?.ti,ab,jx. (36015)

44	"peer-to-peer".ti,ab. (455)

45	(peer adj2 (led or leader* or participat* or mediat* or helper? or resistan* or wide or action? or influen*)).ti,ab. (249)

46	(relationship* adj2 (led or leader* or participat* or mediat* or helper? or resistan* or wide or action? or influen*)).ti,ab. (1439)

47	((community or communities) adj2 (led or leader* or participat* or mediat* or helper? or resistan* or wide or action? or influen* or relationship*)).ti,ab. (1654)

48	(pupil? adj2 (led or leader* or participat* or mediat* or helper? or resistan* or wide or action? or influen* relationship*)).ti,ab. (4)

49	((child or children) adj2 (led or leader* or participat* or mediat* or helper? or resistan* or wide or action? or influen*)).ti,ab. (452)

50	((young people or young person) adj2 (led or leader* or participat* or mediat* or helper? or resistan* or wide or action? or influen*)).ti,ab. (37)

51	(teen* adj2 (led or leader* or participat* or mediat* or helper? or resistan* or wide or action? or influen*)).ti,ab. (33)

52	(adolescent* adj2 (led or leader* or participat* or mediat* or helper? or resistan* or wide or action? or influen*)).ti,ab. (69)

53	(youth? adj2 (led or leader* or participat* or mediat* or helper? or resistan* or wide or action? or influen*)).ti,ab. (151)

54	(student? adj2 (led or leader* or participat* or mediat* or helper? or resistan* or wide or action? or influen*)).ti,ab. (589)

55	(teach* adj2 (led or leader* or participat* or mediat* or helper? or resistan* or wide or action? or influen* or relationship*)).ti,ab. (279)

56	(curricul* adj2 (led or leader* or participat* or mediat* or helper? or resistan* or wide or action? or influen* or relationship*)).ti,ab. (23)

57	(administrat* adj2 (led or leader* or participat* or mediat* or helper? or resistan* or wide or action? or influen* or relationship*)).ti,ab. (327)

58	((personnel or staff) adj2 (led or leader* or participat* or mediat* or helper? or resistan* or wide or action? or influen* or relationship*)).ti,ab. (132)

59	(volunteer* adj2 (led or leader* or participat* or mediat* or helper? or resistan* or wide or action? or influen* or relationship*)).ti,ab. (151)

60	((advocacy or advocate) adj2 (led or leader* or participat* or mediat* or helper? or resistan* or wide or action? or influen* or relationship*)).ti,ab. (63)

61	(parent* adj2 (led or leader* or participat* or mediat* or helper? or resistan* or wide or action? or influen*)).ti,ab. (434)

62	(environment* adj2 (led or leader* or participat* or mediat* or helper? or resistan* or wide or action? or influen* or relationship*)).ti,ab. (1828)

63	(socio-ecolog* adj2 (led or leader* or participat* or mediat* or helper? or resistan* or wide or action? or influen* or relationship*)).ti,ab. (2)

64	(learn* adj2 (led or leader* or participat* or mediat* or helper? or resistan* or wide or action? or influen* or relationship*)).ti,ab. (544)

65	((policy or policies) adj2 (led or leader* or participat* or mediat* or helper? or resistan* or wide or action? or influen* or relationship*)).ti,ab. (5335)

66	or/44-65 (13742)

67	43 and 66 (1226)

68	or/40-42,67 (7167)

69	((smoking or smoked or smoke or smoker) adj5 (reduc* or decreas* or increas* or lower* or fewer or improv* or enhance* or extend* or develop* or prevalen* or daily or weekly or current or rate or rates or behavio?r* or knowledge or prevent* or attitude* or avoid* or perceive* or percept* or promot* or program* or change*)).ti,ab. (1145)

70	(tobacco adj5 (reduc* or decreas* or increas* or lower* or fewer or improv* or enhance* or extend* or develop* or prevalen* or daily or weekly or current or rate or rates or behavio?r* or knowledge or prevent* or attitude* or avoid* or perceive* or percept* or promot* or program* or change*)).ti,ab. (441)

71	(cigarette? adj5 (reduc* or decreas* or increas* or lower* or fewer or improv* or enhance* or extend* or develop* or prevalen* or daily or weekly or current or rate or rates or behavio?r* or knowledge or prevent* or attitude* or avoid* or perceive* or percept* or promot* or program* or change*)).ti,ab. (536)

72	(marijuana adj5 (reduc* or decreas* or increas* or lower* or fewer or improv* or enhance* or extend* or develop* or prevalen* or daily or weekly or current or rate or rates or behavio?r* or knowledge or prevent* or attitude* or avoid* or perceive* or percept* or promot* or program* or change*)).ti,ab. (115)

73	(cannabis adj5 (reduc* or decreas* or increas* or lower* or fewer or improv* or enhance* or extend* or develop* or prevalen* or daily or weekly or current or rate or rates or behavio?r* or knowledge or prevent* or attitude* or avoid* or perceive* or percept* or promot* or program* or change*)).ti,ab. (55)

74	(solvent? adj5 (reduc* or decreas* or increas* or lower* or fewer or improv* or enhance* or extend* or develop* or prevalen* or daily or weekly or current or rate or rates or behavio?r* or knowledge or prevent* or attitude* or avoid* or perceive* or percept* or promot* or program* or change*)).ti,ab. (31)

75	(alcohol adj5 (reduc* or decreas* or increas* or lower* or fewer or improv* or enhance* or extend* or develop* or prevalen* or daily or weekly or current or rate or rates or behavio?r* or knowledge or prevent* or attitude* or avoid* or perceive* or percept* or promot* or program* or change*)).ti,ab. (656)

76	(binge drink* adj5 (reduc* or decreas* or increas* or lower* or fewer or improv* or enhance* or extend* or develop* or prevalen* or daily or weekly or current or rate or rates or behavio?r* or knowledge or prevent* or attitude* or avoid* or perceive* or percept* or promot* or program* or change*)).ti,ab. (53)

77	(underage drink* adj5 (reduc* or decreas* or increas* or lower* or fewer or improv* or enhance* or extend* or develop* or prevalen* or daily or weekly or current or rate or rates or behavio?r* or knowledge or prevent* or attitude* or avoid* or perceive* or percept* or promot* or program* or change*)).ti,ab. (9)

78	(substance? adj5 (reduc* or decreas* or increas* or lower* or fewer or improv* or enhance* or extend* or develop* or prevalen* or daily or weekly or current or rate or rates or behavio?r* or knowledge or prevent* or attitude* or avoid* or perceive* or percept* or promot* or program* or change*)).ti,ab. (283)

79	(drug? adj5 (reduc* or decreas* or increas* or lower* or fewer or improv* or enhance* or extend* or develop* or prevalen* or daily or weekly or current or rate or rates or behavio?r* or knowledge or prevent* or attitude* or avoid* or perceive* or percept* or promot* or program* or change*)).ti,ab. (1735)

80	(education* attain* adj5 (reduc* or decreas* or increas* or lower* or fewer or improv* or enhance* or extend* or develop* or prevalen* or daily or weekly or current or rate or rates or behavio?r* or knowledge or prevent* or attitude* or avoid* or perceive* or percept* or promot* or program* or change*)).ti,ab. (1207)

81	(academic attain* adj5 (reduc* or decreas* or increas* or lower* or fewer or improv* or enhance* or extend* or develop* or prevalen* or daily or weekly or current or rate or rates or behavio?r* or knowledge or prevent* or attitude* or avoid* or perceive* or percept* or promot* or program* or change*)).ti,ab. (8)

82	(student* attain* adj5 (reduc* or decreas* or increas* or lower* or fewer or improv* or enhance* or extend* or develop* or prevalen* or daily or weekly or current or rate or rates or behavio?r* or knowledge or prevent* or attitude* or avoid* or perceive* or percept* or promot* or program* or change*)).ti,ab. (9)

83	(education* achieve* adj5 (reduc* or decreas* or increas* or lower* or fewer or improv* or enhance* or extend* or develop* or prevalen* or daily or weekly or current or rate or rates or behavio?r* or knowledge or prevent* or attitude* or avoid* or perceive* or percept* or promot* or program* or change*)).ti,ab. (108)

84	(academic achieve* adj5 (reduc* or decreas* or increas* or lower* or fewer or improv* or enhance* or extend* or develop* or prevalen* or daily or weekly or current or rate or rates or behavio?r* or knowledge or prevent* or attitude* or avoid* or perceive* or percept* or promot* or program* or change*)).ti,ab. (159)

85	(student* achieve* adj5 (reduc* or decreas* or increas* or lower* or fewer or improv* or enhance* or extend* or develop* or prevalen* or daily or weekly or current or rate or rates or behavio?r* or knowledge or prevent* or attitude* or avoid* or perceive* or percept* or promot* or program* or change*)).ti,ab. (305)

86	(education* perform* adj5 (reduc* or decreas* or increas* or lower* or fewer or improv* or enhance* or extend* or develop* or prevalen* or daily or weekly or current or rate or rates or behavio?r* or knowledge or prevent* or attitude* or avoid* or perceive* or percept* or promot* or program* or change*)).ti,ab. (54)

87	(academic perform* adj5 (reduc* or decreas* or increas* or lower* or fewer or improv* or enhance* or extend* or develop* or prevalen* or daily or weekly or current or rate or rates or behavio?r* or knowledge or prevent* or attitude* or avoid* or perceive* or percept* or promot* or program* or change*)).ti,ab. (190)

88	(student* perform* adj5 (reduc* or decreas* or increas* or lower* or fewer or improv* or enhance* or extend* or develop* or prevalen* or daily or weekly or current or rate or rates or behavio?r* or knowledge or prevent* or attitude* or avoid* or perceive* or percept* or promot* or program* or change*)).ti,ab. (216)

89	(education* outcome? adj5 (reduc* or decreas* or increas* or lower* or fewer or improv* or enhance* or extend* or develop* or prevalen* or daily or weekly or current or rate or rates or behavio?r* or knowledge or prevent* or attitude* or avoid* or perceive* or percept* or promot* or program* or change*)).ti,ab. (349)

90	(academic outcome? adj5 (reduc* or decreas* or increas* or lower* or fewer or improv* or enhance* or extend* or develop* or prevalen* or daily or weekly or current or rate or rates or behavio?r* or knowledge or prevent* or attitude* or avoid* or perceive* or percept* or promot* or program* or change*)).ti,ab. (62)

91	(student* outcome? adj5 (reduc* or decreas* or increas* or lower* or fewer or improv* or enhance* or extend* or develop* or prevalen* or daily or weekly or current or rate or rates or behavio?r* or knowledge or prevent* or attitude* or avoid* or perceive* or percept* or promot* or program* or change*)).ti,ab. (118)

92	(education* commitment adj5 (reduc* or decreas* or increas* or lower* or fewer or improv* or enhance* or extend* or develop* or prevalen* or daily or weekly or current or rate or rates or behavio?r* or knowledge or prevent* or attitude* or avoid* or perceive* or percept* or promot* or program* or change*)).ti,ab. (0)

93	(academic commitment adj5 (reduc* or decreas* or increas* or lower* or fewer or improv* or enhance* or extend* or develop* or prevalen* or daily or weekly or current or rate or rates or behavio?r* or knowledge or prevent* or attitude* or avoid* or perceive* or percept* or promot* or program* or change*)).ti,ab. (2)

94	(student* commitment adj5 (reduc* or decreas* or increas* or lower* or fewer or improv* or enhance* or extend* or develop* or prevalen* or daily or weekly or current or rate or rates or behavio?r* or knowledge or prevent* or attitude* or avoid* or perceive* or percept* or promot* or program* or change*)).ti,ab. (2)

95	(education* engage* adj5 (reduc* or decreas* or increas* or lower* or fewer or improv* or enhance* or extend* or develop* or prevalen* or daily or weekly or current or rate or rates or behavio?r* or knowledge or prevent* or attitude* or avoid* or perceive* or percept* or promot* or program* or change*)).ti,ab. (1)

96	(academic engage* adj5 (reduc* or decreas* or increas* or lower* or fewer or improv* or enhance* or extend* or develop* or prevalen* or daily or weekly or current or rate or rates or behavio?r* or knowledge or prevent* or attitude* or avoid* or perceive* or percept* or promot* or program* or change*)).ti,ab. (7)

97	(student* engage* adj5 (reduc* or decreas* or increas* or lower* or fewer or improv* or enhance* or extend* or develop* or prevalen* or daily or weekly or current or rate or rates or behavio?r* or knowledge or prevent* or attitude* or avoid* or perceive* or percept* or promot* or program* or change*)).ti,ab. (21)

98	(dropout adj5 (reduc* or decreas* or increas* or lower* or fewer or improv* or enhance* or extend* or develop* or prevalen* or daily or weekly or current or rate or rates or behavio?r* or knowledge or prevent* or attitude* or avoid* or perceive* or percept* or promot* or program* or change*)).ti,ab. (447)

99	(absentee* adj5 (reduc* or decreas* or increas* or lower* or fewer or improv* or enhance* or extend* or develop* or prevalen* or daily or weekly or current or rate or rates or behavio?r* or knowledge or prevent* or attitude* or avoid* or perceive* or percept* or promot* or program* or change*)).ti,ab. (251)

100	((violent or violence) adj5 (reduc* or decreas* or increas* or lower* or fewer or improv* or enhance* or extend* or develop* or prevalen* or daily or weekly or current or rate or rates or behavio?r* or knowledge or prevent* or attitude* or avoid* or perceive* or percept* or promot* or program* or change*)).ti,ab. (1707)

101	((aggression or aggressive) adj5 (reduc* or decreas* or increas* or lower* or fewer or improv* or enhance* or extend* or develop* or prevalen* or daily or weekly or current or rate or rates or behavio?r* or knowledge or prevent* or attitude* or avoid* or perceive* or percept* or promot* or program* or change*)).ti,ab. (698)

102	((hostile or hostility) adj5 (reduc* or decreas* or increas* or lower* or fewer or improv* or enhance* or extend* or develop* or prevalen* or daily or weekly or current or rate or rates or behavio?r* or knowledge or prevent* or attitude* or avoid* or perceive* or percept* or promot* or program* or change*)).ti,ab. (209)

103	(assault* adj5 (reduc* or decreas* or increas* or lower* or fewer or improv* or enhance* or extend* or develop* or prevalen* or daily or weekly or current or rate or rates or behavio?r* or knowledge or prevent* or attitude* or avoid* or perceive* or percept* or promot* or program* or change*)).ti,ab. (91)

104	(crime adj5 (reduc* or decreas* or increas* or lower* or fewer or improv* or enhance* or extend* or develop* or prevalen* or daily or weekly or current or rate or rates or behavio?r* or knowledge or prevent* or attitude* or avoid* or perceive* or percept* or promot* or program* or change*)).ti,ab. (2175)

105	((abuse? or abusive) adj5 (reduc* or decreas* or increas* or lower* or fewer or improv* or enhance* or extend* or develop* or prevalen* or daily or weekly or current or rate or rates or behavio?r* or knowledge or prevent* or attitude* or avoid* or perceive* or percept* or promot* or program* or change*)).ti,ab. (593)

106	(misbehav* adj5 (reduc* or decreas* or increas* or lower* or fewer or improv* or enhance* or extend* or develop* or prevalen* or daily or weekly or current or rate or rates or behavio?r* or knowledge or prevent* or attitude* or avoid* or perceive* or percept* or promot* or program* or change*)).ti,ab. (47)

107	(threat? adj5 (reduc* or decreas* or increas* or lower* or fewer or improv* or enhance* or extend* or develop* or prevalen* or daily or weekly or current or rate or rates or behavio?r* or knowledge or prevent* or attitude* or avoid* or perceive* or percept* or promot* or program* or change*)).ti,ab. (1716)

108	(conduct adj5 (reduc* or decreas* or increas* or lower* or fewer or improv* or enhance* or extend* or develop* or prevalen* or daily or weekly or current or rate or rates or behavio?r* or knowledge or prevent* or attitude* or avoid* or perceive* or percept* or promot* or program* or change*)).ti,ab. (1121)

109	(delinquen* adj5 (reduc* or decreas* or increas* or lower* or fewer or improv* or enhance* or extend* or develop* or prevalen* or daily or weekly or current or rate or rates or behavio?r* or knowledge or prevent* or attitude* or avoid* or perceive* or percept* or promot* or program* or change*)).ti,ab. (406)

110	(disrupt* behavio?r* adj5 (reduc* or decreas* or increas* or lower* or fewer or improv* or enhance* or extend* or develop* or prevalen* or daily or weekly or current or rate or rates or behavio?r* or knowledge or prevent* or attitude* or avoid* or perceive* or percept* or promot* or program* or change*)).ti,ab. (18)

111	(problem* behavio?r* adj5 (reduc* or decreas* or increas* or lower* or fewer or improv* or enhance* or extend* or develop* or prevalen* or daily or weekly or current or rate or rates or behavio?r* or knowledge or prevent* or attitude* or avoid* or perceive* or percept* or promot* or program* or change*)).ti,ab. (76)

112	(volatile behavio?r* adj5 (reduc* or decreas* or increas* or lower* or fewer or improv* or enhance* or extend* or develop* or prevalen* or daily or weekly or current or rate or rates or behavio?r* or knowledge or prevent* or attitude* or avoid* or perceive* or percept* or promot* or program* or change*)).ti,ab. (28)

113	((antisocial or anti-social) adj5 (reduc* or decreas* or increas* or lower* or fewer or improv* or enhance* or extend* or develop* or prevalen* or daily or weekly or current or rate or rates or behavio?r* or knowledge or prevent* or attitude* or avoid* or perceive* or percept* or promot* or program* or change*)).ti,ab. (138)

114	(perpetrat* adj5 (reduc* or decreas* or increas* or lower* or fewer or improv* or enhance* or extend* or develop* or prevalen* or daily or weekly or current or rate or rates or behavio?r* or knowledge or prevent* or attitude* or avoid* or perceive* or percept* or promot* or program* or change*)).ti,ab. (35)

115	(bully* adj5 (reduc* or decreas* or increas* or lower* or fewer or improv* or enhance* or extend* or develop* or prevalen* or daily or weekly or current or rate or rates or behavio?r* or knowledge or prevent* or attitude* or avoid* or perceive* or percept* or promot* or program* or change*)).ti,ab. (65)

116	(victim* adj5 (reduc* or decreas* or increas* or lower* or fewer or improv* or enhance* or extend* or develop* or prevalen* or daily or weekly or current or rate or rates or behavio?r* or knowledge or prevent* or attitude* or avoid* or perceive* or percept* or promot* or program* or change*)).ti,ab. (404)

117	(cyberbully* adj5 (reduc* or decreas* or increas* or lower* or fewer or improv* or enhance* or extend* or develop* or prevalen* or daily or weekly or current or rate or rates or behavio?r* or knowledge or prevent* or attitude* or avoid* or perceive* or percept* or promot* or program* or change*)).ti,ab. (4)

118	(conflict* adj5 (reduc* or decreas* or increas* or lower* or fewer or improv* or enhance* or extend* or develop* or prevalen* or daily or weekly or current or rate or rates or behavio?r* or knowledge or prevent* or attitude* or avoid* or perceive* or percept* or promot* or program* or change*)).ti,ab. (4291)

119	(positive behavio?r* adj5 (reduc* or decreas* or increas* or lower* or fewer or improv* or enhance* or extend* or develop* or prevalen* or daily or weekly or current or rate or rates or behavio?r* or knowledge or prevent* or attitude* or avoid* or perceive* or percept* or promot* or program* or change*)).ti,ab. (47)

120	(improv* behavio?r* adj5 (reduc* or decreas* or increas* or lower* or fewer or improv* or enhance* or extend* or develop* or prevalen* or daily or weekly or current or rate or rates or behavio?r* or knowledge or prevent* or attitude* or avoid* or perceive* or percept* or promot* or program* or change*)).ti,ab. (38)

121	(social behavio?r* adj5 (reduc* or decreas* or increas* or lower* or fewer or improv* or enhance* or extend* or develop* or prevalen* or daily or weekly or current or rate or rates or behavio?r* or knowledge or prevent* or attitude* or avoid* or perceive* or percept* or promot* or program* or change*)).ti,ab. (601)

122	((social-emotion* or socio-emotion*) adj5 (reduc* or decreas* or increas* or lower* or fewer or improv* or enhance* or extend* or develop* or prevalen* or daily or weekly or current or rate or rates or behavio?r* or knowledge or prevent* or attitude* or avoid* or perceive* or percept* or promot* or program* or change*)).ti,ab. (78)

123	(social-charact* adj5 (reduc* or decreas* or increas* or lower* or fewer or improv* or enhance* or extend* or develop* or prevalen* or daily or weekly or current or rate or rates or behavio?r* or knowledge or prevent* or attitude* or avoid* or perceive* or percept* or promot* or program* or change*)).ti,ab. (27)

124	(social-inclusion adj5 (reduc* or decreas* or increas* or lower* or fewer or improv* or enhance* or extend* or develop* or prevalen* or daily or weekly or current or rate or rates or behavio?r* or knowledge or prevent* or attitude* or avoid* or perceive* or percept* or promot* or program* or change*)).ti,ab. (165)

125	(social develop* adj5 (reduc* or decreas* or increas* or lower* or fewer or improv* or enhance* or extend* or develop* or prevalen* or daily or weekly or current or rate or rates or behavio?r* or knowledge or prevent* or attitude* or avoid* or perceive* or percept* or promot* or program* or change*)).ti,ab. (1763)

126	(social competen* adj5 (reduc* or decreas* or increas* or lower* or fewer or improv* or enhance* or extend* or develop* or prevalen* or daily or weekly or current or rate or rates or behavio?r* or knowledge or prevent* or attitude* or avoid* or perceive* or percept* or promot* or program* or change*)).ti,ab. (12)

127	(emotion* develop* adj5 (reduc* or decreas* or increas* or lower* or fewer or improv* or enhance* or extend* or develop* or prevalen* or daily or weekly or current or rate or rates or behavio?r* or knowledge or prevent* or attitude* or avoid* or perceive* or percept* or promot* or program* or change*)).ti,ab. (49)

128	(conduct adj5 (reduc* or decreas* or increas* or lower* or fewer or improv* or enhance* or extend* or develop* or prevalen* or daily or weekly or current or rate or rates or behavio?r* or knowledge or prevent* or attitude* or avoid* or perceive* or percept* or promot* or program* or change*)).ti,ab. (1121)

129	(respect adj5 (reduc* or decreas* or increas* or lower* or fewer or improv* or enhance* or extend* or develop* or prevalen* or daily or weekly or current or rate or rates or behavio?r* or knowledge or prevent* or attitude* or avoid* or perceive* or percept* or promot* or program* or change*)).ti,ab. (3613)

130	((safety or safe) adj5 (reduc* or decreas* or increas* or lower* or fewer or improv* or enhance* or extend* or develop* or prevalen* or daily or weekly or current or rate or rates or behavio?r* or knowledge or prevent* or attitude* or avoid* or perceive* or percept* or promot* or program* or change*)).ti,ab. (2989)

131	or/69-130 (28696)

132	prevent*.ti,ab. (17329)

133	program*.ti,ab. (68585)

134	intervent*.ti,ab. (23751)

135	restorative approach*.ti,ab. (3)

136	control school?.ti,ab. (26)

137	(trial or trials).ti,ab. (4376)

138	or/132-137 (106760)

139	39 and 68 and 131 and 138 (268)

## EBSCO Educational Resources Information Center (ERIC)

| Database name | Educational Resources Information Center (ERIC) |
| --- | --- |
| Database platform | EBSCO |
| Dates of database coverage | Complete database to date searched. |
| Date searched | 19/05/2021 |
| Searched by | JF |
| Number of results | 15,414 |
| EndNote import order | 12 |
| Number of results once duplicates removed | 1301 |
| Search strategy notes | * is used for truncation. # is used for an optional wildcard. Search strings starting with TI search the title only. Search strings starting with AB search the abstract only. Search strings starting with SO search the journal title only. N/*n* search for terms within *n* words of each other, in any order. |

| Search number | Query | Results |
| --- | --- | --- |
| S1 | (TI child*) or (AB child*) | 302,072 |
| S2 | (TI boy#) or (AB boy#) | 22,057 |
| S3 | (TI (schoolage or (school N1 age))) or (AB (schoolage or (school N1 age))) | 6,806 |
| S4 | (TI schoolchild*) or (AB schoolchild*) | 1,155 |
| S5 | (TI youngster#) OR (AB youngster#) | 2,203 |
| S6 | (TI minor#) OR (AB minor#) | 4,511 |
| S7 | (TI pupil*) OR (AB pupil*) | 28,242 |
| S8 | ((TI student*) OR (AB student*)) NOT ((TI "medical student*") or (AB "medical student*")) | 714,258 |
| S9 | (TI prepubescen*) OR (AB prepubescen*) | 31 |
| S10 | (TI girl#) or (AB girl#) | 23,515 |
| S11 | (TI pubescent*) or (AB pubescent*) | 26 |
| S12 | (TI adolescent*) OR (AB adolescent*) | 46,248 |
| S13 | (TI juvenil*) OR (AB juvenil*) | 5,373 |
| S14 | (TI underage*) or (AB underage*) | 221 |
| S15 | (TI (preteen* or pre-teen*)) OR (AB (preteen* or pre-teen*)) | 177 |
| S16 | (TI (teen or teens)) OR (AB (teen or teens)) | 5,231 |
| S17 | (TI teenage*) OR (AB teenage*) | 6,320 |
| S18 | (TI (youth or youths)) OR (AB (youth or youths)) | 48,526 |
| S19 | (TI "young person*") OR (AB "young person*") | 812 |
| S20 | (TI "young people*") OR (AB "young people*") | 16,605 |
| S21 | (TI (kid or kids)) OR (AB (kid or kids)) | 6,412 |
| S22 | (TI (transition n4 adult*)) OR (AB (transition N4 adult*)) | 1,825 |
| S23 | (TI "emerging adult*") OR (AB "emerging adult*") | 658 |
| S24 | (TI "young adult*") OR (AB "young adult*") | 9,674 |
| S25 | (TI parent#) OR (AB parent#) | 107,922 |
| S26 | (TI mother#) OR (AB mother#) | 23,351 |
| S27 | (TI father#) or (AB father#) | 7,929 |
| S28 | (TI guardian#) OR (AB guardian#) | 1,356 |
| S29 | (TI teacher#) OR (AB teacher#) | 396,086 |
| S30 | (SO child*) | 69,438 |
| S31 | (SO adolescen*) | 12,110 |
| S32 | (SO youth*) | 6,995 |
| S33 | (SO school*) | 62,926 |
| S34 | (TI ("5" or "6" or "7" or "8" or "9" or "10" or "11" or "12" or "13" or "14" or "15" or "16" or "17" or "18") N1 ("year* old" or "year* of age")) OR (AB ("5" or "6" or "7" or "8" or "9" or "10" or "11" or "12" or "13" or "14" or "15" or "16" or "17" or "18") N1 ("year* old" or "year* of age")) | 6,083 |
| S35 | (TI (five or six or seven or eight or nine or ten or eleven or twelve or thirteen or fourteen or fifteen or sixteen or seventeen or eighteen) N1 ("year* old" or "year* of age")) OR (AB (five or six or seven or eight or nine or ten or eleven or twelve or thirteen or fourteen or fifteen or sixteen or seventeen or eighteen) N1 ("year* old" or "year* of age")) | 9,623 |
| S36 | (TI (age* N1 ("5" or "6" or "7" or "8" or "9" or "10" or "11" or "12" or "13" or "14" or "15" or "16" or "17" or "18") N1 year*)) OR (AB (age* N1 ("5" or "6" or "7" or "8" or "9" or "10" or "11" or "12" or "13" or "14" or "15" or "16" or "17" or "18") N1 year*)) | 2,280 |
| S37 | (TI (age* N1 (five or six or seven or eight or nine or ten or eleven or twelve or thirteen or fourteen or fifteen or sixteen or seventeen or eighteen) N1 year*)) OR (AB (age* N1 (five or six or seven or eight or nine or ten or eleven or twelve or thirteen or fourteen or fifteen or sixteen or seventeen or eighteen) N1 year*)) | 2,694 |
| S38 | (TI (primary or secondary or year) N1 ("1" or "2" or "3" or "4" or "5" or "6" or "7" or "8" or "9" or "10" or "11" or "12" or "13")) OR (AB (primary or secondary or year) N1 ("1" or "2" or "3" or "4" or "5" or "6" or "7" or "8" or "9" or "10" or "11" or "12" or "13")) | 44,227 |
| S39 | (TI grade# N1 (first or second or third or fourth or fifth or sixth or seventh or eighth or ninth or tenth or eleventh or twelfth)) OR (AB grade# N1 (first or second or third or fourth or fifth or sixth or seventh or eighth or ninth or tenth or eleventh or twelfth)) | 59,390 |
| S40 | S1 OR S2 OR S3 OR S4 OR S5 OR S6 OR S7 OR S8 OR S9 OR S10 OR S11 OR S12 OR S13 OR S14 OR S15 OR S16 OR S17 OR S18 OR S19 OR S20 OR S21 OR S22 OR S23 OR S24 OR S25 OR S26 OR S27 OR S28 OR S29 OR S30 OR S31 OR S32 OR S33 OR S34 OR S35 OR S36 OR S37 OR S38 OR S39 | 1,171,151 |
| S41 | (TI (school# N5 (based or level or wide or led or ethos or environment or organi#ation* or climate or toolkit# or approach* or "action group#" or practice# or whole or health* or leader* or cultur* or manag* or governance or system# or communit*))) OR (AB (school# N5 (based or level or wide or led or ethos or environment or organi#ation* or climate or toolkit# or approach* or "action group#" or practice# or whole or health* or leader* or cultur* or manag* or governance or system# or communit*))) | 170,201 |
| S42 | (TI schoolwide) or (AB schoolwide) | 1,945 |
| S43 | (TI "restor* justice") OR (AB "restor* justice") | 210 |
| S44 | (TI school#) or (AB school#) | 529,402 |
| S45 | (TI "peer-to-peer") OR (AB "peer-to-peer") | 759 |
| S46 | (TI (peer N2 (led or leader* or participat* or mediat* or helper# or resistan* or wide or action# or influen*))) OR (AB (peer N2 (led or leader* or participat* or mediat* or helper# or resistan* or wide or action# or influen*))) | 4,132 |
| S47 | (TI (relationship* N2 (led or leader* or participat* or mediat* or helper# or resistan* or wide or action# or influen*))) OR (AB (relationship* N2 (led or leader* or participat* or mediat* or helper# or resistan* or wide or action# or influen*))) | 5,935 |
| S48 | (TI ((community or communities) N2 (led or leader* or participat* or mediat* or helper# or resistan* or wide or action# or influen* or relationship*))) OR (AB ((community or communities) N2 (led or leader* or participat* or mediat* or helper# or resistan* or wide or action# or influen* or relationship*))) | 14,447 |
| S49 | ((TI (pupil# N2 (led or leader* or participat* or mediat* or helper# or resistan* or wide or action# or influen* or relationship*)) OR (AB (pupil# N2 (led or leader* or participat* or mediat* or helper# or resistan* or wide or action# or influen* or relationship*))) | 1,329 |
| S50 | (TI ((child or children) N2 (led or leader* or participat* or mediat* or helper# or resistan* or wide or action# or influen*))) OR (AB (child or children*) N2 (led or leader* or participat* or mediat* or helper# or resistan* or wide or action# or influen*))) | 11,149 |
| S51 | (TI (("young people" or "young person") N2 (led or leader* or participat* or mediat* or helper# or resistan* or wide or action# or influen*))) OR (AB (("young people" or "young person") N2 (led or leader* or participat* or mediat* or helper# or resistan* or wide or action# or influen*))) | 510 |
| S52 | (TI ((teen or teens or teenage*) N2 (led or leader* or participat* or mediat* or helper# or resistan* or wide or action# or influen*))) OR (AB ((teen or teens or teenage*) N2 (led or leader* or participat* or mediat* or helper# or resistan* or wide or action# or influen*))) | 294 |
| S53 | (TI (adolescen* N2 (led or leader* or participat* or mediat* or helper# or resistan* or wide or action# or influen*))) OR (AB (adolescen* N2 (led or leader* or participat* or mediat* or helper# or resistan* or wide or action# or influen*))) | 2,019 |
| S54 | (TI (youth# N2 (led or leader* or participat* or mediat* or helper# or resistan* or wide or action# or influen*))) OR (AB (youth# N2 (led or leader* or participat* or mediat* or helper# or resistan* or wide or action# or influen*))) | 2,667 |
| S55 | (TI (student# N2 (led or leader* or participat* or mediat* or helper# or resistan* or wide or action# or influen* or relationship*))) OR (AB (student# N2 (led or leader* or participat* or mediat* or helper# or resistan* or wide or action# or influen* or relationship*))) | 51,760 |
| S56 | (TI (teach* N2 (led or leader* or participat* or mediat* or helper# or resistan* or wide or action# or influen* or relationship*))) OR (AB (teach* N2 (led or leader* or participat* or mediat* or helper# or resistan* or wide or action# or influen* or relationship*))) | 39,090 |
| S57 | (TI (curricul* N2 (led or leader* or participat* or mediat* or helper# or resistan* or wide or action# or influen* or relationship*))) OR (AB (curricul* N2 (led or leader* or participat* or mediat* or helper# or resistan* or wide or action# or influen* or relationship*))) | 4,434 |
| S58 | (TI (administrat* N2 (led or leader* or participat* or mediat* or helper# or resistan* or wide or action# or influen* or relationship*))) OR (AB (administrat* N2 (led or leader* or participat* or mediat* or helper# or resistan* or wide or action# or influen* or relationship*))) | 5,462 |
| S59 | (TI ((personnel or staff) N2 (led or leader* or participat* or mediat* or helper# or resistan* or wide or action# or influen* or relationship*))) OR (AB ((personnel or staff) N2 (led or leader* or participat* or mediat* or helper# or resistan* or wide or action# or influen* or relationship*))) | 4,762 |
| S60 | (TI (volunteer* N2 (led or leader* or participat* or mediat* or helper# or resistan* or wide or action# or influen* or relationship*))) OR (AB (volulnteer* N2 (led or leader* or participat* or mediat* or helper# or resistan* or wide or action# or influen* or relationship*))) | 113 |
| S61 | (TI ((advocacy or advocate) N2 (led or leader* or participat* or mediat* or helper# or resistan* or wide or action# or influen* or relationship*))) OR (AB ((advocacy or advocate) N2 (led or leader* or participat* or mediat* or helper# or resistan* or wide or action# or influen* or relationship*))) | 1,011 |
| S62 | (TI (parent* N2 (led or leader* or participat* or mediat* or helper# or resistan* or wide or action# or influen*))) OR (AB (parent* N2 (led or leader* or participat* or mediat* or helper# or resistan* or wide or action# or influen*))) | 9,270 |
| S63 | (TI (environment* N2 (led or leader* or participat* or mediat* or helper# or resistan* or wide or action# or influen* or relationship*))) OR (AB (environment* N2 (led or leader* or participat* or mediat* or helper# or resistan* or wide or action# or influen* or relationship*))) | 6,255 |
| S64 | (TI ("socio-ecolog*" N2 (led or leader* or participat* or mediat* or helper# or resistan* or wide or action# or influen* or relationship*))) OR (AB ("socio-ecolog*" N2 (led or leader* or participat* or mediat* or helper# or resistan* or wide or action# or influen* or relationship*))) | 9 |
| S65 | (TI (learn* N2 (led or leader* or participat* or mediat* or helper# or resistan* or wide or action# or influen* or relationship*))) OR (AB (learn* N2 (led or leader* or participat* or mediat* or helper# or resistan* or wide or action# or influen* or relationship*))) | 21,247 |
| S66 | (TI ((policy or policies) N2 (led or leader* or participat* or mediat* or helper# or resistan* or wide or action# or influen* or relationship*))) OR (AB ((policy or policies) N2 (led or leader* or participat* or mediat* or helper# or resistan* or wide or action# or influen* or relationship*))) | 6,852 |
| S67 | S45 OR S46 OR S47 OR S48 OR S49 OR S50 OR S51 OR S52 OR S53 OR S54 OR S55 OR S56 OR S57 OR S58 OR S59 OR S60 OR S61 OR S62 OR S63 OR S64 OR S65 OR S66 | 158,748 |
| S68 | S44 AND S67 | 71,453 |
| S69 | S41 OR S42 OR S43 OR S68 | 210,710 |
| S70 | (TI ((smoking or smoked or smoke or smoker) N5 (reduc* or decreas* or increas* or lower* or fewer or improv* or enhance* or extend* or develop* or prevalen* or daily or weekly or current or rate or rates or behavio#r* or knowledge or prevent* or attitude* or avoid* or perceive* or percept* or promot* or program* or change*))) OR (AB ((smoking or smoked or smoke or smoker) N5 (reduc* or decreas* or increas* or lower* or fewer or improv* or enhance* or extend* or develop* or prevalen* or daily or weekly or current or rate or rates or behavio#r* or knowledge or prevent* or attitude* or avoid* or perceive* or percept* or promot* or program* or change*))) | 1,874 |
| S71 | (TI (tobacco N5 (reduc* or decreas* or increas* or lower* or fewer or improv* or enhance* or extend* or develop* or prevalen* or daily or weekly or current or rate or rates or behavio#r* or knowledge or prevent* or attitude* or avoid* or perceive* or percept* or promot* or program* or change*)) OR (AB (tobacco N5 (reduc* or decreas* or increas* or lower* or fewer or improv* or enhance* or extend* or develop* or prevalen* or daily or weekly or current or rate or rates or behavio#r* or knowledge or prevent* or attitude* or avoid* or perceive* or percept* or promot* or program* or change*)) | 1,011 |
| S72 | (TI (cigarette# N5 (reduc* or decreas* or increas* or lower* or fewer or improv* or enhance* or extend* or develop* or prevalen* or daily or weekly or current or rate or rates or behavio#r* or knowledge or prevent* or attitude* or avoid* or perceive* or percept* or promot* or program* or change*)) OR (AB (cigarette# N5 (reduc* or decreas* or increas* or lower* or fewer or improv* or enhance* or extend* or develop* or prevalen* or daily or weekly or current or rate or rates or behavio#r* or knowledge or prevent* or attitude* or avoid* or perceive* or percept* or promot* or program* or change*)) | 535 |
| S73 | (TI (marijuana N5 (reduc* or decreas* or increas* or lower* or fewer or improv* or enhance* or extend* or develop* or prevalen* or daily or weekly or current or rate or rates or behavio#r* or knowledge or prevent* or attitude* or avoid* or perceive* or percept* or promot* or program* or change*)) OR (AB (marijuana N5 (reduc* or decreas* or increas* or lower* or fewer or improv* or enhance* or extend* or develop* or prevalen* or daily or weekly or current or rate or rates or behavio#r* or knowledge or prevent* or attitude* or avoid* or perceive* or percept* or promot* or program* or change*)) | 451 |
| S74 | (TI (cannabis N5 (reduc* or decreas* or increas* or lower* or fewer or improv* or enhance* or extend* or develop* or prevalen* or daily or weekly or current or rate or rates or behavio#r* or knowledge or prevent* or attitude* or avoid* or perceive* or percept* or promot* or program* or change*)) OR (AB (cannabis N5 (reduc* or decreas* or increas* or lower* or fewer or improv* or enhance* or extend* or develop* or prevalen* or daily or weekly or current or rate or rates or behavio#r* or knowledge or prevent* or attitude* or avoid* or perceive* or percept* or promot* or program* or change*)) | 99 |
| S75 | (TI (solvent# N5 (reduc* or decreas* or increas* or lower* or fewer or improv* or enhance* or extend* or develop* or prevalen* or daily or weekly or current or rate or rates or behavio#r* or knowledge or prevent* or attitude* or avoid* or perceive* or percept* or promot* or program* or change*)) OR (AB (solvent# N5 (reduc* or decreas* or increas* or lower* or fewer or improv* or enhance* or extend* or develop* or prevalen* or daily or weekly or current or rate or rates or behavio#r* or knowledge or prevent* or attitude* or avoid* or perceive* or percept* or promot* or program* or change*)) | 58 |
| S76 | (TI (alcohol N5 (reduc* or decreas* or increas* or lower* or fewer or improv* or enhance* or extend* or develop* or prevalen* or daily or weekly or current or rate or rates or behavio#r* or knowledge or prevent* or attitude* or avoid* or perceive* or percept* or promot* or program* or change*)) OR (AB (alcohol N5 (reduc* or decreas* or increas* or lower* or fewer or improv* or enhance* or extend* or develop* or prevalen* or daily or weekly or current or rate or rates or behavio#r* or knowledge or prevent* or attitude* or avoid* or perceive* or percept* or promot* or program* or change*)) | 4,308 |
| S77 | (TI ("binge drink*" N5 (reduc* or decreas* or increas* or lower* or fewer or improv* or enhance* or extend* or develop* or prevalen* or daily or weekly or current or rate or rates or behavio#r* or knowledge or prevent* or attitude* or avoid* or perceive* or percept* or promot* or program* or change*)) OR (AB ("binge drink*" N5 (reduc* or decreas* or increas* or lower* or fewer or improv* or enhance* or extend* or develop* or prevalen* or daily or weekly or current or rate or rates or behavio#r* or knowledge or prevent* or attitude* or avoid* or perceive* or percept* or promot* or program* or change*)) | 186 |
| S78 | (TI ("underage drink*" N5 (reduc* or decreas* or increas* or lower* or fewer or improv* or enhance* or extend* or develop* or prevalen* or daily or weekly or current or rate or rates or behavio#r* or knowledge or prevent* or attitude* or avoid* or perceive* or percept* or promot* or program* or change*)) OR (AB ("underage drink*" N5 (reduc* or decreas* or increas* or lower* or fewer or improv* or enhance* or extend* or develop* or prevalen* or daily or weekly or current or rate or rates or behavio#r* or knowledge or prevent* or attitude* or avoid* or perceive* or percept* or promot* or program* or change*)) | 65 |
| S79 | (TI ("underage drink*" N5 (reduc* or decreas* or increas* or lower* or fewer or improv* or enhance* or extend* or develop* or prevalen* or daily or weekly or current or rate or rates or behavio#r* or knowledge or prevent* or attitude* or avoid* or perceive* or percept* or promot* or program* or change*)) OR (AB ("underage drink*" N5 (reduc* or decreas* or increas* or lower* or fewer or improv* or enhance* or extend* or develop* or prevalen* or daily or weekly or current or rate or rates or behavio#r* or knowledge or prevent* or attitude* or avoid* or perceive* or percept* or promot* or program* or change*)) | 65 |
| S80 | (TI (substance# N5 (reduc* or decreas* or increas* or lower* or fewer or improv* or enhance* or extend* or develop* or prevalen* or daily or weekly or current or rate or rates or behavio#r* or knowledge or prevent* or attitude* or avoid* or perceive* or percept* or promot* or program* or change*)) OR (AB (substance# N5 (reduc* or decreas* or increas* or lower* or fewer or improv* or enhance* or extend* or develop* or prevalen* or daily or weekly or current or rate or rates or behavio#r* or knowledge or prevent* or attitude* or avoid* or perceive* or percept* or promot* or program* or change*)) | 3,809 |
| S81 | (TI (drug# N5 (reduc* or decreas* or increas* or lower* or fewer or improv* or enhance* or extend* or develop* or prevalen* or daily or weekly or current or rate or rates or behavio#r* or knowledge or prevent* or attitude* or avoid* or perceive* or percept* or promot* or program* or change*)) OR (AB (drug# N5 (reduc* or decreas* or increas* or lower* or fewer or improv* or enhance* or extend* or develop* or prevalen* or daily or weekly or current or rate or rates or behavio#r* or knowledge or prevent* or attitude* or avoid* or perceive* or percept* or promot* or program* or change*)) | 6,003 |
| S82 | (TI ("education* attain*" N5 (reduc* or decreas* or increas* or lower* or fewer or improv* or enhance* or extend* or develop* or prevalen* or daily or weekly or current or rate or rates or behavio#r* or knowledge or prevent* or attitude* or avoid* or perceive* or percept* or promot* or program* or change*)) OR (AB ("education* attain*" N5 (reduc* or decreas* or increas* or lower* or fewer or improv* or enhance* or extend* or develop* or prevalen* or daily or weekly or current or rate or rates or behavio#r* or knowledge or prevent* or attitude* or avoid* or perceive* or percept* or promot* or program* or change*)) | 1,503 |
| S83 | (TI ("academic attain*" N5 (reduc* or decreas* or increas* or lower* or fewer or improv* or enhance* or extend* or develop* or prevalen* or daily or weekly or current or rate or rates or behavio#r* or knowledge or prevent* or attitude* or avoid* or perceive* or percept* or promot* or program* or change*)) OR (AB ("academic attain*" N5 (reduc* or decreas* or increas* or lower* or fewer or improv* or enhance* or extend* or develop* or prevalen* or daily or weekly or current or rate or rates or behavio#r* or knowledge or prevent* or attitude* or avoid* or perceive* or percept* or promot* or program* or change*)) | 97 |
| S84 | (TI ("student* attain*" N5 (reduc* or decreas* or increas* or lower* or fewer or improv* or enhance* or extend* or develop* or prevalen* or daily or weekly or current or rate or rates or behavio#r* or knowledge or prevent* or attitude* or avoid* or perceive* or percept* or promot* or program* or change*)) OR (AB ("student* attain*" N5 (reduc* or decreas* or increas* or lower* or fewer or improv* or enhance* or extend* or develop* or prevalen* or daily or weekly or current or rate or rates or behavio#r* or knowledge or prevent* or attitude* or avoid* or perceive* or percept* or promot* or program* or change*)) | 199 |
| S85 | (TI ("education* achieve*" N5 (reduc* or decreas* or increas* or lower* or fewer or improv* or enhance* or extend* or develop* or prevalen* or daily or weekly or current or rate or rates or behavio#r* or knowledge or prevent* or attitude* or avoid* or perceive* or percept* or promot* or program* or change*)) OR (AB ("education* achieve*" N5 (reduc* or decreas* or increas* or lower* or fewer or improv* or enhance* or extend* or develop* or prevalen* or daily or weekly or current or rate or rates or behavio#r* or knowledge or prevent* or attitude* or avoid* or perceive* or percept* or promot* or program* or change*)) | 652 |
| S86 | (TI ("academic achieve*" N5 (reduc* or decreas* or increas* or lower* or fewer or improv* or enhance* or extend* or develop* or prevalen* or daily or weekly or current or rate or rates or behavio#r* or knowledge or prevent* or attitude* or avoid* or perceive* or percept* or promot* or program* or change*)) OR (AB ("academic achieve*" N5 (reduc* or decreas* or increas* or lower* or fewer or improv* or enhance* or extend* or develop* or prevalen* or daily or weekly or current or rate or rates or behavio#r* or knowledge or prevent* or attitude* or avoid* or perceive* or percept* or promot* or program* or change*)) | 6,220 |
| S87 | (TI ("student* achieve*" N5 (reduc* or decreas* or increas* or lower* or fewer or improv* or enhance* or extend* or develop* or prevalen* or daily or weekly or current or rate or rates or behavio#r* or knowledge or prevent* or attitude* or avoid* or perceive* or percept* or promot* or program* or change*)) OR (AB ("student* achieve*" N5 (reduc* or decreas* or increas* or lower* or fewer or improv* or enhance* or extend* or develop* or prevalen* or daily or weekly or current or rate or rates or behavio#r* or knowledge or prevent* or attitude* or avoid* or perceive* or percept* or promot* or program* or change*)) | 9,519 |
| S88 | (TI ("education* perform*" N5 (reduc* or decreas* or increas* or lower* or fewer or improv* or enhance* or extend* or develop* or prevalen* or daily or weekly or current or rate or rates or behavio#r* or knowledge or prevent* or attitude* or avoid* or perceive* or percept* or promot* or program* or change*)) OR (AB ("education* perform*" N5 (reduc* or decreas* or increas* or lower* or fewer or improv* or enhance* or extend* or develop* or prevalen* or daily or weekly or current or rate or rates or behavio#r* or knowledge or prevent* or attitude* or avoid* or perceive* or percept* or promot* or program* or change*)) | 276 |
| S89 | (TI ("academic perform*" N5 (reduc* or decreas* or increas* or lower* or fewer or improv* or enhance* or extend* or develop* or prevalen* or daily or weekly or current or rate or rates or behavio#r* or knowledge or prevent* or attitude* or avoid* or perceive* or percept* or promot* or program* or change*)) OR (AB ("academic perform*" N5 (reduc* or decreas* or increas* or lower* or fewer or improv* or enhance* or extend* or develop* or prevalen* or daily or weekly or current or rate or rates or behavio#r* or knowledge or prevent* or attitude* or avoid* or perceive* or percept* or promot* or program* or change*)) | 3,794 |
| S90 | (TI ("student* perform*" N5 (reduc* or decreas* or increas* or lower* or fewer or improv* or enhance* or extend* or develop* or prevalen* or daily or weekly or current or rate or rates or behavio#r* or knowledge or prevent* or attitude* or avoid* or perceive* or percept* or promot* or program* or change*)) OR (AB ("student* perform*" N5 (reduc* or decreas* or increas* or lower* or fewer or improv* or enhance* or extend* or develop* or prevalen* or daily or weekly or current or rate or rates or behavio#r* or knowledge or prevent* or attitude* or avoid* or perceive* or percept* or promot* or program* or change*)) | 4,789 |
| S91 | (TI ("education* outcome#" N5 (reduc* or decreas* or increas* or lower* or fewer or improv* or enhance* or extend* or develop* or prevalen* or daily or weekly or current or rate or rates or behavio#r* or knowledge or prevent* or attitude* or avoid* or perceive* or percept* or promot* or program* or change*)) OR (AB ("education* outcome#" N5 (reduc* or decreas* or increas* or lower* or fewer or improv* or enhance* or extend* or develop* or prevalen* or daily or weekly or current or rate or rates or behavio#r* or knowledge or prevent* or attitude* or avoid* or perceive* or percept* or promot* or program* or change*)) | 1,561 |
| S92 | (TI ("academic outcome#" N5 (reduc* or decreas* or increas* or lower* or fewer or improv* or enhance* or extend* or develop* or prevalen* or daily or weekly or current or rate or rates or behavio#r* or knowledge or prevent* or attitude* or avoid* or perceive* or percept* or promot* or program* or change*)) OR (AB ("academic outcome#" N5 (reduc* or decreas* or increas* or lower* or fewer or improv* or enhance* or extend* or develop* or prevalen* or daily or weekly or current or rate or rates or behavio#r* or knowledge or prevent* or attitude* or avoid* or perceive* or percept* or promot* or program* or change*)) | 915 |
| S93 | (TI ("student* outcome#" N5 (reduc* or decreas* or increas* or lower* or fewer or improv* or enhance* or extend* or develop* or prevalen* or daily or weekly or current or rate or rates or behavio#r* or knowledge or prevent* or attitude* or avoid* or perceive* or percept* or promot* or program* or change*)) OR (AB ("student* outcome#" N5 (reduc* or decreas* or increas* or lower* or fewer or improv* or enhance* or extend* or develop* or prevalen* or daily or weekly or current or rate or rates or behavio#r* or knowledge or prevent* or attitude* or avoid* or perceive* or percept* or promot* or program* or change*)) | 2,841 |
| S94 | (TI ("education* commitment" N5 (reduc* or decreas* or increas* or lower* or fewer or improv* or enhance* or extend* or develop* or prevalen* or daily or weekly or current or rate or rates or behavio#r* or knowledge or prevent* or attitude* or avoid* or perceive* or percept* or promot* or program* or change*)) OR (AB ("education* commitment" N5 (reduc* or decreas* or increas* or lower* or fewer or improv* or enhance* or extend* or develop* or prevalen* or daily or weekly or current or rate or rates or behavio#r* or knowledge or prevent* or attitude* or avoid* or perceive* or percept* or promot* or program* or change*)) | 25 |
| S95 | (TI ("education* commitment" N5 (reduc* or decreas* or increas* or lower* or fewer or improv* or enhance* or extend* or develop* or prevalen* or daily or weekly or current or rate or rates or behavio#r* or knowledge or prevent* or attitude* or avoid* or perceive* or percept* or promot* or program* or change*)) OR (AB ("education* commitment" N5 (reduc* or decreas* or increas* or lower* or fewer or improv* or enhance* or extend* or develop* or prevalen* or daily or weekly or current or rate or rates or behavio#r* or knowledge or prevent* or attitude* or avoid* or perceive* or percept* or promot* or program* or change*)) | 25 |
| S96 | (TI ("academic commitment" N5 (reduc* or decreas* or increas* or lower* or fewer or improv* or enhance* or extend* or develop* or prevalen* or daily or weekly or current or rate or rates or behavio#r* or knowledge or prevent* or attitude* or avoid* or perceive* or percept* or promot* or program* or change*)) OR (AB ("academic commitment" N5 (reduc* or decreas* or increas* or lower* or fewer or improv* or enhance* or extend* or develop* or prevalen* or daily or weekly or current or rate or rates or behavio#r* or knowledge or prevent* or attitude* or avoid* or perceive* or percept* or promot* or program* or change*)) | 12 |
| S97 | (TI ("academic commitment" N5 (reduc* or decreas* or increas* or lower* or fewer or improv* or enhance* or extend* or develop* or prevalen* or daily or weekly or current or rate or rates or behavio#r* or knowledge or prevent* or attitude* or avoid* or perceive* or percept* or promot* or program* or change*)) OR (AB ("academic commitment" N5 (reduc* or decreas* or increas* or lower* or fewer or improv* or enhance* or extend* or develop* or prevalen* or daily or weekly or current or rate or rates or behavio#r* or knowledge or prevent* or attitude* or avoid* or perceive* or percept* or promot* or program* or change*)) | 12 |
| S98 | (TI ("student* commitment" N5 (reduc* or decreas* or increas* or lower* or fewer or improv* or enhance* or extend* or develop* or prevalen* or daily or weekly or current or rate or rates or behavio#r* or knowledge or prevent* or attitude* or avoid* or perceive* or percept* or promot* or program* or change*)) OR (AB ("student* commitment" N5 (reduc* or decreas* or increas* or lower* or fewer or improv* or enhance* or extend* or develop* or prevalen* or daily or weekly or current or rate or rates or behavio#r* or knowledge or prevent* or attitude* or avoid* or perceive* or percept* or promot* or program* or change*)) | 70 |
| S99 | (TI ("education* engage*" N5 (reduc* or decreas* or increas* or lower* or fewer or improv* or enhance* or extend* or develop* or prevalen* or daily or weekly or current or rate or rates or behavio#r* or knowledge or prevent* or attitude* or avoid* or perceive* or percept* or promot* or program* or change*)) OR (AB ("education* engage*" N5 (reduc* or decreas* or increas* or lower* or fewer or improv* or enhance* or extend* or develop* or prevalen* or daily or weekly or current or rate or rates or behavio#r* or knowledge or prevent* or attitude* or avoid* or perceive* or percept* or promot* or program* or change*)) | 41 |
| S100 | (TI ("academic engage*" N5 (reduc* or decreas* or increas* or lower* or fewer or improv* or enhance* or extend* or develop* or prevalen* or daily or weekly or current or rate or rates or behavio#r* or knowledge or prevent* or attitude* or avoid* or perceive* or percept* or promot* or program* or change*)) OR (AB ("academic engage*" N5 (reduc* or decreas* or increas* or lower* or fewer or improv* or enhance* or extend* or develop* or prevalen* or daily or weekly or current or rate or rates or behavio#r* or knowledge or prevent* or attitude* or avoid* or perceive* or percept* or promot* or program* or change*)) | 405 |
| S101 | (TI ("student* engage*" N5 (reduc* or decreas* or increas* or lower* or fewer or improv* or enhance* or extend* or develop* or prevalen* or daily or weekly or current or rate or rates or behavio#r* or knowledge or prevent* or attitude* or avoid* or perceive* or percept* or promot* or program* or change*)) OR (AB ("student* engage*" N5 (reduc* or decreas* or increas* or lower* or fewer or improv* or enhance* or extend* or develop* or prevalen* or daily or weekly or current or rate or rates or behavio#r* or knowledge or prevent* or attitude* or avoid* or perceive* or percept* or promot* or program* or change*)) | 3,177 |
| S102 | (TI (dropout N5 (reduc* or decreas* or increas* or lower* or fewer or improv* or enhance* or extend* or develop* or prevalen* or daily or weekly or current or rate or rates or behavio#r* or knowledge or prevent* or attitude* or avoid* or perceive* or percept* or promot* or program* or change*)) OR (AB (dropout N5 (reduc* or decreas* or increas* or lower* or fewer or improv* or enhance* or extend* or develop* or prevalen* or daily or weekly or current or rate or rates or behavio#r* or knowledge or prevent* or attitude* or avoid* or perceive* or percept* or promot* or program* or change*)) | 6,894 |
| S103 | (TI (absentee* N5 (reduc* or decreas* or increas* or lower* or fewer or improv* or enhance* or extend* or develop* or prevalen* or daily or weekly or current or rate or rates or behavio#r* or knowledge or prevent* or attitude* or avoid* or perceive* or percept* or promot* or program* or change*)) OR (AB (absentee* N5 (reduc* or decreas* or increas* or lower* or fewer or improv* or enhance* or extend* or develop* or prevalen* or daily or weekly or current or rate or rates or behavio#r* or knowledge or prevent* or attitude* or avoid* or perceive* or percept* or promot* or program* or change*)) | 1,013 |
| S104 | (TI ((violent or violence) N5 (reduc* or decreas* or increas* or lower* or fewer or improv* or enhance* or extend* or develop* or prevalen* or daily or weekly or current or rate or rates or behavio#r* or knowledge or prevent* or attitude* or avoid* or perceive* or percept* or promot* or program* or change*))) OR (AB ((violent or violence) N5 (reduc* or decreas* or increas* or lower* or fewer or improv* or enhance* or extend* or develop* or prevalen* or daily or weekly or current or rate or rates or behavio#r* or knowledge or prevent* or attitude* or avoid* or perceive* or percept* or promot* or program* or change*))) | 6,016 |
| S105 | (TI ((aggression or aggressive) N5 (reduc* or decreas* or increas* or lower* or fewer or improv* or enhance* or extend* or develop* or prevalen* or daily or weekly or current or rate or rates or behavio#r* or knowledge or prevent* or attitude* or avoid* or perceive* or percept* or promot* or program* or change*))) OR (AB ((aggression or aggressive) N5 (reduc* or decreas* or increas* or lower* or fewer or improv* or enhance* or extend* or develop* or prevalen* or daily or weekly or current or rate or rates or behavio#r* or knowledge or prevent* or attitude* or avoid* or perceive* or percept* or promot* or program* or change*))) | 4,533 |
| S106 | (TI ((hostile or hostility) N5 (reduc* or decreas* or increas* or lower* or fewer or improv* or enhance* or extend* or develop* or prevalen* or daily or weekly or current or rate or rates or behavio#r* or knowledge or prevent* or attitude* or avoid* or perceive* or percept* or promot* or program* or change*))) OR (AB ((hostile or hostility) N5 (reduc* or decreas* or increas* or lower* or fewer or improv* or enhance* or extend* or develop* or prevalen* or daily or weekly or current or rate or rates or behavio#r* or knowledge or prevent* or attitude* or avoid* or perceive* or percept* or promot* or program* or change*))) | 749 |
| S107 | (TI (assault* N5 (reduc* or decreas* or increas* or lower* or fewer or improv* or enhance* or extend* or develop* or prevalen* or daily or weekly or current or rate or rates or behavio#r* or knowledge or prevent* or attitude* or avoid* or perceive* or percept* or promot* or program* or change*)) OR (AB (assault* N5 (reduc* or decreas* or increas* or lower* or fewer or improv* or enhance* or extend* or develop* or prevalen* or daily or weekly or current or rate or rates or behavio#r* or knowledge or prevent* or attitude* or avoid* or perceive* or percept* or promot* or program* or change*)) | 544 |
| S108 | (TI (crime N5 (reduc* or decreas* or increas* or lower* or fewer or improv* or enhance* or extend* or develop* or prevalen* or daily or weekly or current or rate or rates or behavio#r* or knowledge or prevent* or attitude* or avoid* or perceive* or percept* or promot* or program* or change*)) OR (AB (crime N5 (reduc* or decreas* or increas* or lower* or fewer or improv* or enhance* or extend* or develop* or prevalen* or daily or weekly or current or rate or rates or behavio#r* or knowledge or prevent* or attitude* or avoid* or perceive* or percept* or promot* or program* or change*)) | 2,094 |
| S109 | (TI ((abuse# or abusive) N5 (reduc* or decreas* or increas* or lower* or fewer or improv* or enhance* or extend* or develop* or prevalen* or daily or weekly or current or rate or rates or behavio#r* or knowledge or prevent* or attitude* or avoid* or perceive* or percept* or promot* or program* or change*))) OR (AB ((abuse# or abusive) N5 (reduc* or decreas* or increas* or lower* or fewer or improv* or enhance* or extend* or develop* or prevalen* or daily or weekly or current or rate or rates or behavio#r* or knowledge or prevent* or attitude* or avoid* or perceive* or percept* or promot* or program* or change*))) | 6,998 |
| S110 | (TI (misbehav* N5 (reduc* or decreas* or increas* or lower* or fewer or improv* or enhance* or extend* or develop* or prevalen* or daily or weekly or current or rate or rates or behavio#r* or knowledge or prevent* or attitude* or avoid* or perceive* or percept* or promot* or program* or change*)) OR (AB (misbehav* N5 (reduc* or decreas* or increas* or lower* or fewer or improv* or enhance* or extend* or develop* or prevalen* or daily or weekly or current or rate or rates or behavio#r* or knowledge or prevent* or attitude* or avoid* or perceive* or percept* or promot* or program* or change*)) | 428 |
| S111 | (TI (threat# N5 (reduc* or decreas* or increas* or lower* or fewer or improv* or enhance* or extend* or develop* or prevalen* or daily or weekly or current or rate or rates or behavio#r* or knowledge or prevent* or attitude* or avoid* or perceive* or percept* or promot* or program* or change*)) OR (AB (threat# N5 (reduc* or decreas* or increas* or lower* or fewer or improv* or enhance* or extend* or develop* or prevalen* or daily or weekly or current or rate or rates or behavio#r* or knowledge or prevent* or attitude* or avoid* or perceive* or percept* or promot* or program* or change*)) | 1,677 |
| S112 | (TI (conduct N5 (reduc* or decreas* or increas* or lower* or fewer or improv* or enhance* or extend* or develop* or prevalen* or daily or weekly or current or rate or rates or behavio#r* or knowledge or prevent* or attitude* or avoid* or perceive* or percept* or promot* or program* or change*)) OR (AB (conduct N5 (reduc* or decreas* or increas* or lower* or fewer or improv* or enhance* or extend* or develop* or prevalen* or daily or weekly or current or rate or rates or behavio#r* or knowledge or prevent* or attitude* or avoid* or perceive* or percept* or promot* or program* or change*)) | 3,884 |
| S113 | (TI (delinquen* N5 (reduc* or decreas* or increas* or lower* or fewer or improv* or enhance* or extend* or develop* or prevalen* or daily or weekly or current or rate or rates or behavio#r* or knowledge or prevent* or attitude* or avoid* or perceive* or percept* or promot* or program* or change*)) OR (AB (delinquen* N5 (reduc* or decreas* or increas* or lower* or fewer or improv* or enhance* or extend* or develop* or prevalen* or daily or weekly or current or rate or rates or behavio#r* or knowledge or prevent* or attitude* or avoid* or perceive* or percept* or promot* or program* or change*)) | 2,562 |
| S114 | (TI ("disrupt* behavio#r*" N5 (reduc* or decreas* or increas* or lower* or fewer or improv* or enhance* or extend* or develop* or prevalen* or daily or weekly or current or rate or rates or behavio#r* or knowledge or prevent* or attitude* or avoid* or perceive* or percept* or promot* or program* or change*)) OR (AB ("disrupt* behavio#r*" N5 (reduc* or decreas* or increas* or lower* or fewer or improv* or enhance* or extend* or develop* or prevalen* or daily or weekly or current or rate or rates or behavio#r* or knowledge or prevent* or attitude* or avoid* or perceive* or percept* or promot* or program* or change*)) | 1,805 |
| S115 | (TI ("problem* behavio#r*" N5 (reduc* or decreas* or increas* or lower* or fewer or improv* or enhance* or extend* or develop* or prevalen* or daily or weekly or current or rate or rates or behavio#r* or knowledge or prevent* or attitude* or avoid* or perceive* or percept* or promot* or program* or change*)) OR (AB ("problem* behavio#r*" N5 (reduc* or decreas* or increas* or lower* or fewer or improv* or enhance* or extend* or develop* or prevalen* or daily or weekly or current or rate or rates or behavio#r* or knowledge or prevent* or attitude* or avoid* or perceive* or percept* or promot* or program* or change*)) | 3,773 |
| S116 | (TI ("volatile behavio#r*" N5 (reduc* or decreas* or increas* or lower* or fewer or improv* or enhance* or extend* or develop* or prevalen* or daily or weekly or current or rate or rates or behavio#r* or knowledge or prevent* or attitude* or avoid* or perceive* or percept* or promot* or program* or change*)) OR (AB ("volatile behavio#r*" N5 (reduc* or decreas* or increas* or lower* or fewer or improv* or enhance* or extend* or develop* or prevalen* or daily or weekly or current or rate or rates or behavio#r* or knowledge or prevent* or attitude* or avoid* or perceive* or percept* or promot* or program* or change*)) | 1 |
| S117 | (TI ("volatile behavio#r*" N5 (reduc* or decreas* or increas* or lower* or fewer or improv* or enhance* or extend* or develop* or prevalen* or daily or weekly or current or rate or rates or behavio#r* or knowledge or prevent* or attitude* or avoid* or perceive* or percept* or promot* or program* or change*)) OR (AB ("volatile behavio#r*" N5 (reduc* or decreas* or increas* or lower* or fewer or improv* or enhance* or extend* or develop* or prevalen* or daily or weekly or current or rate or rates or behavio#r* or knowledge or prevent* or attitude* or avoid* or perceive* or percept* or promot* or program* or change*)) | 1 |
| S118 | (TI ((antisocial or "anti-social") N5 (reduc* or decreas* or increas* or lower* or fewer or improv* or enhance* or extend* or develop* or prevalen* or daily or weekly or current or rate or rates or behavio#r* or knowledge or prevent* or attitude* or avoid* or perceive* or percept* or promot* or program* or change*))) OR (AB ((antisocial or "anti-social") N5 (reduc* or decreas* or increas* or lower* or fewer or improv* or enhance* or extend* or develop* or prevalen* or daily or weekly or current or rate or rates or behavio#r* or knowledge or prevent* or attitude* or avoid* or perceive* or percept* or promot* or program* or change*))) | 1,661 |
| S119 | (TI (perpetrat* N5 (reduc* or decreas* or increas* or lower* or fewer or improv* or enhance* or extend* or develop* or prevalen* or daily or weekly or current or rate or rates or behavio#r* or knowledge or prevent* or attitude* or avoid* or perceive* or percept* or promot* or program* or change*)) OR (AB (perpetrat* N5 (reduc* or decreas* or increas* or lower* or fewer or improv* or enhance* or extend* or develop* or prevalen* or daily or weekly or current or rate or rates or behavio#r* or knowledge or prevent* or attitude* or avoid* or perceive* or percept* or promot* or program* or change*)) | 423 |
| S120 | (TI (bully* N5 (reduc* or decreas* or increas* or lower* or fewer or improv* or enhance* or extend* or develop* or prevalen* or daily or weekly or current or rate or rates or behavio#r* or knowledge or prevent* or attitude* or avoid* or perceive* or percept* or promot* or program* or change*)) OR (AB (bully* N5 (reduc* or decreas* or increas* or lower* or fewer or improv* or enhance* or extend* or develop* or prevalen* or daily or weekly or current or rate or rates or behavio#r* or knowledge or prevent* or attitude* or avoid* or perceive* or percept* or promot* or program* or change*)) | 2,271 |
| S121 | (TI (victim* N5 (reduc* or decreas* or increas* or lower* or fewer or improv* or enhance* or extend* or develop* or prevalen* or daily or weekly or current or rate or rates or behavio#r* or knowledge or prevent* or attitude* or avoid* or perceive* or percept* or promot* or program* or change*)) OR (AB (victim* N5 (reduc* or decreas* or increas* or lower* or fewer or improv* or enhance* or extend* or develop* or prevalen* or daily or weekly or current or rate or rates or behavio#r* or knowledge or prevent* or attitude* or avoid* or perceive* or percept* or promot* or program* or change*)) | 2,586 |
| S122 | (TI (cyberbully* N5 (reduc* or decreas* or increas* or lower* or fewer or improv* or enhance* or extend* or develop* or prevalen* or daily or weekly or current or rate or rates or behavio#r* or knowledge or prevent* or attitude* or avoid* or perceive* or percept* or promot* or program* or change*)) OR (AB (cyberbully* N5 (reduc* or decreas* or increas* or lower* or fewer or improv* or enhance* or extend* or develop* or prevalen* or daily or weekly or current or rate or rates or behavio#r* or knowledge or prevent* or attitude* or avoid* or perceive* or percept* or promot* or program* or change*)) | 319 |
| S123 | (TI (conflict* N5 (reduc* or decreas* or increas* or lower* or fewer or improv* or enhance* or extend* or develop* or prevalen* or daily or weekly or current or rate or rates or behavio#r* or knowledge or prevent* or attitude* or avoid* or perceive* or percept* or promot* or program* or change*)) OR (AB (conflict* N5 (reduc* or decreas* or increas* or lower* or fewer or improv* or enhance* or extend* or develop* or prevalen* or daily or weekly or current or rate or rates or behavio#r* or knowledge or prevent* or attitude* or avoid* or perceive* or percept* or promot* or program* or change*)) | 7,107 |
| S124 | (TI ("positive behavio#r*" N5 (reduc* or decreas* or increas* or lower* or fewer or improv* or enhance* or extend* or develop* or prevalen* or daily or weekly or current or rate or rates or behavio#r* or knowledge or prevent* or attitude* or avoid* or perceive* or percept* or promot* or program* or change*)) OR (AB ("positive behavio#r*" N5 (reduc* or decreas* or increas* or lower* or fewer or improv* or enhance* or extend* or develop* or prevalen* or daily or weekly or current or rate or rates or behavio#r* or knowledge or prevent* or attitude* or avoid* or perceive* or percept* or promot* or program* or change*)) | 2,113 |
| S125 | (TI ("improv* behavio#r*" N5 (reduc* or decreas* or increas* or lower* or fewer or improv* or enhance* or extend* or develop* or prevalen* or daily or weekly or current or rate or rates or behavio#r* or knowledge or prevent* or attitude* or avoid* or perceive* or percept* or promot* or program* or change*)) OR (AB ("improv* behavio#r*" N5 (reduc* or decreas* or increas* or lower* or fewer or improv* or enhance* or extend* or develop* or prevalen* or daily or weekly or current or rate or rates or behavio#r* or knowledge or prevent* or attitude* or avoid* or perceive* or percept* or promot* or program* or change*)) | 325 |
| S126 | (TI ("social behavio#r*" N5 (reduc* or decreas* or increas* or lower* or fewer or improv* or enhance* or extend* or develop* or prevalen* or daily or weekly or current or rate or rates or behavio#r* or knowledge or prevent* or attitude* or avoid* or perceive* or percept* or promot* or program* or change*)) OR (AB ("social behavio#r*" N5 (reduc* or decreas* or increas* or lower* or fewer or improv* or enhance* or extend* or develop* or prevalen* or daily or weekly or current or rate or rates or behavio#r* or knowledge or prevent* or attitude* or avoid* or perceive* or percept* or promot* or program* or change*)) | 3,696 |
| S127 | (TI (("social-emotion*" or "socio-emotion*") N5 (reduc* or decreas* or increas* or lower* or fewer or improv* or enhance* or extend* or develop* or prevalen* or daily or weekly or current or rate or rates or behavio#r* or knowledge or prevent* or attitude* or avoid* or perceive* or percept* or promot* or program* or change*))) OR (AB (("social-emotion*" or "socio-emotion*") N5 (reduc* or decreas* or increas* or lower* or fewer or improv* or enhance* or extend* or develop* or prevalen* or daily or weekly or current or rate or rates or behavio#r* or knowledge or prevent* or attitude* or avoid* or perceive* or percept* or promot* or program* or change*))) | 2,851 |
| S128 | (TI ("social-charact*" N5 (reduc* or decreas* or increas* or lower* or fewer or improv* or enhance* or extend* or develop* or prevalen* or daily or weekly or current or rate or rates or behavio#r* or knowledge or prevent* or attitude* or avoid* or perceive* or percept* or promot* or program* or change*)) OR (AB ("social-charact*" N5 (reduc* or decreas* or increas* or lower* or fewer or improv* or enhance* or extend* or develop* or prevalen* or daily or weekly or current or rate or rates or behavio#r* or knowledge or prevent* or attitude* or avoid* or perceive* or percept* or promot* or program* or change*)) | 101 |
| S129 | (TI ("social-inclusion" N5 (reduc* or decreas* or increas* or lower* or fewer or improv* or enhance* or extend* or develop* or prevalen* or daily or weekly or current or rate or rates or behavio#r* or knowledge or prevent* or attitude* or avoid* or perceive* or percept* or promot* or program* or change*)) OR (AB ("social-inclusion" N5 (reduc* or decreas* or increas* or lower* or fewer or improv* or enhance* or extend* or develop* or prevalen* or daily or weekly or current or rate or rates or behavio#r* or knowledge or prevent* or attitude* or avoid* or perceive* or percept* or promot* or program* or change*)) | 340 |
| S130 | (TI ("social develop*" N5 (reduc* or decreas* or increas* or lower* or fewer or improv* or enhance* or extend* or develop* or prevalen* or daily or weekly or current or rate or rates or behavio#r* or knowledge or prevent* or attitude* or avoid* or perceive* or percept* or promot* or program* or change*)) OR (AB ("social develop*" N5 (reduc* or decreas* or increas* or lower* or fewer or improv* or enhance* or extend* or develop* or prevalen* or daily or weekly or current or rate or rates or behavio#r* or knowledge or prevent* or attitude* or avoid* or perceive* or percept* or promot* or program* or change*)) | 3,510 |
| S131 | (TI ("social competen*" N5 (reduc* or decreas* or increas* or lower* or fewer or improv* or enhance* or extend* or develop* or prevalen* or daily or weekly or current or rate or rates or behavio#r* or knowledge or prevent* or attitude* or avoid* or perceive* or percept* or promot* or program* or change*)) OR (AB ("social competen*" N5 (reduc* or decreas* or increas* or lower* or fewer or improv* or enhance* or extend* or develop* or prevalen* or daily or weekly or current or rate or rates or behavio#r* or knowledge or prevent* or attitude* or avoid* or perceive* or percept* or promot* or program* or change*)) | 1,268 |
| S132 | (TI ("emotion* develop*" N5 (reduc* or decreas* or increas* or lower* or fewer or improv* or enhance* or extend* or develop* or prevalen* or daily or weekly or current or rate or rates or behavio#r* or knowledge or prevent* or attitude* or avoid* or perceive* or percept* or promot* or program* or change*)) OR (AB ("emotion* develop*" N5 (reduc* or decreas* or increas* or lower* or fewer or improv* or enhance* or extend* or develop* or prevalen* or daily or weekly or current or rate or rates or behavio#r* or knowledge or prevent* or attitude* or avoid* or perceive* or percept* or promot* or program* or change*)) | 1,999 |
| S133 | (TI (conduct N5 (reduc* or decreas* or increas* or lower* or fewer or improv* or enhance* or extend* or develop* or prevalen* or daily or weekly or current or rate or rates or behavio#r* or knowledge or prevent* or attitude* or avoid* or perceive* or percept* or promot* or program* or change*)) OR (AB (conduct N5 (reduc* or decreas* or increas* or lower* or fewer or improv* or enhance* or extend* or develop* or prevalen* or daily or weekly or current or rate or rates or behavio#r* or knowledge or prevent* or attitude* or avoid* or perceive* or percept* or promot* or program* or change*)) | 3,884 |
| S134 | (TI (respect N5 (reduc* or decreas* or increas* or lower* or fewer or improv* or enhance* or extend* or develop* or prevalen* or daily or weekly or current or rate or rates or behavio#r* or knowledge or prevent* or attitude* or avoid* or perceive* or percept* or promot* or program* or change*)) OR (AB (respect N5 (reduc* or decreas* or increas* or lower* or fewer or improv* or enhance* or extend* or develop* or prevalen* or daily or weekly or current or rate or rates or behavio#r* or knowledge or prevent* or attitude* or avoid* or perceive* or percept* or promot* or program* or change*)) | 5,368 |
| S135 | (TI ((safety or safe) N5 (reduc* or decreas* or increas* or lower* or fewer or improv* or enhance* or extend* or develop* or prevalen* or daily or weekly or current or rate or rates or behavio#r* or knowledge or prevent* or attitude* or avoid* or perceive* or percept* or promot* or program* or change*))) OR (AB ((safety or safe) N5 (reduc* or decreas* or increas* or lower* or fewer or improv* or enhance* or extend* or develop* or prevalen* or daily or weekly or current or rate or rates or behavio#r* or knowledge or prevent* or attitude* or avoid* or perceive* or percept* or promot* or program* or change*))) | 6,583 |
| S136 | S70 OR S71 OR S72 OR S73 OR S74 OR S75 OR S76 OR S77 OR S78 OR S79 OR S80 OR S81 OR S82 OR S83 OR S84 OR S85 OR S86 OR S87 OR S88 OR S89 OR S90 OR S91 OR S92 OR S93 OR S94 OR S95 OR S96 OR S97 OR S98 OR S99 OR S100 OR S101 OR S102 OR S103 OR S104 OR S105 OR S106 OR S107 OR S108 OR S109 OR S110 OR S111 OR S112 OR S113 OR S114 OR S115 OR S116 OR S117 OR S118 OR S119 OR S120 OR S121 OR S122 OR S123 OR S124 OR S125 OR S126 OR S127 OR S128 OR S129 OR S130 OR S131 OR S132 OR S133 OR S134 OR S135 | 110,949 |
| S137 | (TI prevent*) OR (AB prevent*) | 37,516 |
| S138 | (TI program*) OR (AB program*) | 437,449 |
| S139 | (TI intervent*) OR (AB intervent*) | 75,653 |
| S140 | (TI "restorative approach*") OR (AB "restorative approach*") | 44 |
| S141 | (TI "control school#") OR (AB "control school#") | 611 |
| S142 | (TI (trial or trials)) OR (AB (trial or trials)) | 14,638 |
| S143 | S137 OR S138 OR S139 OR S140 OR S141 OR S142 | 511,833 |
| S144 | S40 AND S69 AND S136 AND S143 | 15,414 |

## OvidSP Embase

| Database name | Embase Classic + Embase |
| --- | --- |
| Database platform | OvidSP |
| Dates of database coverage | 1947 to 2021 May 10 |
| Date searched | 11/05/2021 |
| Searched by | JF |
| Number of results | 12,630 |
| EndNote import order | 2 |
| Number of results once duplicates removed | 1536 |
| Search strategy notes | Search lines ending in a ‘/’ are subject heading searches. Search lines ending in ‘/*ab*’ are subject headings with subheadings added. The subheading is listed in square brackets at the end of the line. Search lines beginning ‘exp’ are exploded subject heading searches. Search lines ending in .ti,ab,kw. search in the title, abstract and author keywords only. Search lines ending in .jx. search in the journal title only. or/*x-y* combines search sets in the range *x-y* with Boolean operator OR. * is used for truncation of words. # is used for a compulsory wildcard. ? is used for an optional wildcard. adj*n* searches for terms within *n* words or each other. Words in square brackets [ ] are comments and are not included in the search terms. |

1	child/ (2099038)

2	adolescent/ (1743252)

3	juvenile/ (54466)

4	boy/ (46089)

5	girl/ (58760)

6	school child/ (388944)

7	"minor (person)"/ (722)

8	student/ (120755)

9	elementary student/ (1641)

10	high school student/ (8724)

11	middle school student/ (1505)

12	exp teacher/ (46836)

13	exp parent/ (295807)

14	child*.ti,ab,kw. (2055489)

15	boy?.ti,ab,kw. (223363)

16	girl?.ti,ab,kw. (228704)

17	(schoolage or (school adj1 age)).ti,ab,kw. (20923)

18	schoolchild*.ti,ab,kw. (19318)

19	youngster?.ti,ab,kw. (3868)

20	minor?.ti,ab,kw. (322508)

21	(pupil* or student*).ti,ab,kw. not (pupil/ or exp health student/) [removes items about eyes] (353843)

22	prepubescen*.ti,ab,kw. (1287)

23	pubescent*.ti,ab,kw. (1258)

24	adolescent*.ti,ab,kw. (374024)

25	juvenil*.ti,ab,kw. (113671)

26	underage*.ti,ab,kw. (1721)

27	(preteen* or pre-teen*).ti,ab,kw. (670)

28	(teen or teens).ti,ab,kw. (15685)

29	teenage*.ti,ab,kw. (31933)

30	(youth or youths).ti,ab,kw. (103140)

31	young person*.ti,ab,kw. (6306)

32	young people*.ti,ab,kw. (44573)

33	(kid or kids).ti,ab,kw. (12540)

34	(transition adj4 adult*).ti,ab,kw. (7656)

35	emerging adult*.ti,ab,kw. (3523)

36	young adult*.ti,ab,kw. (141303)

37	parent?.ti,ab,kw. (382163)

38	mother?.ti,ab,kw. (320185)

39	father?.ti,ab,kw. (62626)

40	guardian?.ti,ab,kw. (11067)

41	teacher?.ti,ab,kw. (60502)

42	child*.jx. (214273)

43	adolescen*.jx. (63273)

44	youth*.jx. (2968)

45	school*.jx. (26582)

46	(("5" or "6" or "7" or "8" or "9" or "10" or "11" or "12" or "13" or "14" or "15" or "16" or "17" or "18") adj (year* old or year* of age)).ti,ab,kw. (350912)

47	((five or six or seven or eight or nine or ten or eleven or twelve or thirteen or fourteen or fifteen or sixteen or seventeen or eighteen) adj (year* old or year* of age)).ti,ab,kw. (29839)

48	(age* adj ("5" or "6" or "7" or "8" or "9" or "10" or "11" or "12" or "13" or "14" or "15" or "16" or "17" or "18") adj year*).ti,ab,kw. (79869)

49	(age* adj (five or six or seven or eight or nine or ten or eleven or twelve or thirteen or fourteen or fifteen or sixteen or seventeen or eighteen) adj year*).ti,ab,kw. (1332)

50	((primary or secondary or year) adj1 ("1" or "2" or "3" or "4" or "5" or "6" or "7" or "8" or "9" or "10" or "11" or "12" or "13")).ti,ab,kw. (982652)

51	(grade? adj1 (first or second or third or fourth or fifth or sixth or seventh or eighth or ninth or tenth or eleventh or twelfth)).ti,ab,kw. (13329)

52	or/1-51 (5803612)

53	school/ (78860)

54	high school/ (23298)

55	middle school/ (1985)

56	primary school/ (14826)

57	exp school health service/ (23747)

58	or/53-57 (131894)

59	"organization and management"/ (427341)

60	58 and 59 (7694)

61	(school? adj5 (based or level or wide or led or ethos or environment or organi?ation* or climate or toolkit? or approach* or action group? or practice? or whole or health* or leader* or cultur* or manag* or governance or system? or communit*)).ti,ab,kw. (82603)

62	schoolwide.ti,ab,kw. (111)

63	restor* justice.ti,ab,kw. (207)

64	school?.ti,ab,kw. (377665)

65	or/53-56,64 (393074)

66	"peer-to-peer".ti,ab. (2259)

67	peer pressure/ (2770)

68	(peer adj2 (led or leader* or participat* or mediat* or helper? or resistan* or wide or action? or influen*)).ti,ab,kw. (5502)

69	(relationship* adj2 (led or leader* or participat* or mediat* or helper? or resistan* or wide or action? or influen*)).ti,ab,kw. (20046)

70	community care/ (62238)

71	community integration/ (1135)

72	community program/ (3039)

73	community participation/ (2988)

74	((community or communities) adj2 (led or leader* or participat* or mediat* or helper? or resistan* or wide or action? or influen* or relationship*)).ti,ab,kw. (28084)

75	(pupil? adj2 (led or leader* or participat* or mediat* or helper? or resistan* or wide or action? or influen* or relationship*)).ti,ab,kw. not pupil/ [removes items about eyes] (445)

76	((child or children) adj2 (led or leader* or participat* or mediat* or helper? or resistan* or wide or action? or influen*)).ti,ab,kw. (18839)

77	((young people or young person) adj2 (led or leader* or participat* or mediat* or helper? or resistan* or wide or action? or influen*)).ti,ab,kw. (460)

78	(teen* adj2 (led or leader* or participat* or mediat* or helper? or resistan* or wide or action? or influen*)).ti,ab,kw. (539)

79	(adolescent* adj2 (led or leader* or participat* or mediat* or helper? or resistan* or wide or action? or influen*)).ti,ab,kw. (6123)

80	(youth? adj2 (led or leader* or participat* or mediat* or helper? or resistan* or wide or action? or influen*)).ti,ab,kw. (2655)

81	(student? adj2 (led or leader* or participat* or mediat* or helper? or resistan* or wide or action? or influen* or relationship*)).ti,ab,kw. (19494)

82	(teach* adj2 (led or leader* or participat* or mediat* or helper? or resistan* or wide or action? or influen* or relationship*)).ti,ab,kw. (4427)

83	(curricul* adj2 (led or leader* or participat* or mediat* or helper? or resistan* or wide or action? or influen* or relationship*)).ti,ab,kw. (1113)

84	(administrat* adj2 (led or leader* or participat* or mediat* or helper? or resistan* or wide or action? or influen* or relationship*)).ti,ab,kw. (7393)

85	((personnel or staff) adj2 (led or leader* or participat* or mediat* or helper? or resistan* or wide or action? or influen* or relationship*)).ti,ab,kw. (6526)

86	(volunteer* adj2 (led or leader* or participat* or mediat* or helper? or resistan* or wide or action? or influen* or relationship*)).ti,ab,kw. (8153)

87	((advocacy or advocate) adj2 (led or leader* or participat* or mediat* or helper? or resistan* or wide or action? or influen* or relationship*)).ti,ab,kw. (1129)

88	(parent* adj2 (led or leader* or participat* or mediat* or helper? or resistan* or wide or action? or influen*)).ti,ab,kw. (13346)

89	social environment/ (35415)

90	organizational climate/ (385)

91	psychosocial environment/ (4376)

92	social support/ (98434)

93	social learning/ (3162)

94	(environment* adj2 (led or leader* or participat* or mediat* or helper? or resistan* or wide or action? or influen* or relationship*)).ti,ab,kw. (37200)

95	(socio-ecolog* adj2 (led or leader* or participat* or mediat* or helper? or resistan* or wide or action? or influen* or relationship*)).ti,ab,kw. (52)

96	(learn* adj2 (led or leader* or participat* or mediat* or helper? or resistan* or wide or action? or influen* or relationship*)).ti,ab,kw. (9098)

97	((policy or policies) adj2 (led or leader* or participat* or mediat* or helper? or resistan* or wide or action? or influen* or relationship*)).ti,ab,kw. (7624)

98	or/66-97 (382319)

99	65 and 98 (35130)

100	or/60-63,99 (109743)

101	prevention/ (302206)

102	primary prevention/ (43236)

103	pc.fs. (1227268)

104	or/101-103 [prevention] (1544887)

105	smoking prevention/ (820)

106	smoking reduction/ (280)

107	smoking/ (336850)

108	adolescent smoking/ (2053)

109	cigarette smoking/ (61697)

110	smoking habit/ (24910)

111	cannabis smoking/ (3664)

112	vaping/ (3031)

113	or/107-112 (414068)

114	104 and 113 (34374)

115	((smoking or smoked or smoke or smoker) adj5 (reduc* or decreas* or increas* or lower* or fewer or improv* or enhance* or extend* or develop* or prevalen* or daily or weekly or current or rate or rates or behavio?r* or knowledge or prevent* or attitude* or avoid* or perceive* or percept* or promot* or program* or change*)).ti,ab,kw. (118162)

116	(tobacco adj5 (reduc* or decreas* or increas* or lower* or fewer or improv* or enhance* or extend* or develop* or prevalen* or daily or weekly or current or rate or rates or behavio?r* or knowledge or prevent* or attitude* or avoid* or perceive* or percept* or promot* or program* or change*)).ti,ab,kw. (34159)

117	(cigarette? adj5 (reduc* or decreas* or increas* or lower* or fewer or improv* or enhance* or extend* or develop* or prevalen* or daily or weekly or current or rate or rates or behavio?r* or knowledge or prevent* or attitude* or avoid* or perceive* or percept* or promot* or program* or change*)).ti,ab,kw. (29222)

118	(marijuana adj5 (reduc* or decreas* or increas* or lower* or fewer or improv* or enhance* or extend* or develop* or prevalen* or daily or weekly or current or rate or rates or behavio?r* or knowledge or prevent* or attitude* or avoid* or perceive* or percept* or promot* or program* or change*)).ti,ab,kw. (5232)

119	(cannabis adj5 (reduc* or decreas* or increas* or lower* or fewer or improv* or enhance* or extend* or develop* or prevalen* or daily or weekly or current or rate or rates or behavio?r* or knowledge or prevent* or attitude* or avoid* or perceive* or percept* or promot* or program* or change*)).ti,ab,kw. (7957)

120	(solvent? adj5 (reduc* or decreas* or increas* or lower* or fewer or improv* or enhance* or extend* or develop* or prevalen* or daily or weekly or current or rate or rates or behavio?r* or knowledge or prevent* or attitude* or avoid* or perceive* or percept* or promot* or program* or change*)).ti,ab,kw. (24064)

121	or/105-106,114-120 (203645)

122	addiction/ (56400)

123	exp drug dependence/ (266405)

124	drinking behavior/ (52846)

125	alcohol abuse/ (31948)

126	binge drinking/ (6075)

127	underage drinking/ (1352)

128	substance abuse/ (55670)

129	or/122-128 (407273)

130	104 and 129 (30646)

131	(alcohol adj5 (reduc* or decreas* or increas* or lower* or fewer or improv* or enhance* or extend* or develop* or prevalen* or daily or weekly or current or rate or rates or behavio?r* or knowledge or prevent* or attitude* or avoid* or perceive* or percept* or promot* or program* or change*)).ti,ab,kw. (95869)

132	(binge drink* adj5 (reduc* or decreas* or increas* or lower* or fewer or improv* or enhance* or extend* or develop* or prevalen* or daily or weekly or current or rate or rates or behavio?r* or knowledge or prevent* or attitude* or avoid* or perceive* or percept* or promot* or program* or change*)).ti,ab,kw. (3037)

133	(underage drink* adj5 (reduc* or decreas* or increas* or lower* or fewer or improv* or enhance* or extend* or develop* or prevalen* or daily or weekly or current or rate or rates or behavio?r* or knowledge or prevent* or attitude* or avoid* or perceive* or percept* or promot* or program* or change*)).ti,ab,kw. (281)

134	(substance? adj5 (reduc* or decreas* or increas* or lower* or fewer or improv* or enhance* or extend* or develop* or prevalen* or daily or weekly or current or rate or rates or behavio?r* or knowledge or prevent* or attitude* or avoid* or perceive* or percept* or promot* or program* or change*)).ti,ab,kw. (68945)

135	(drug? adj5 (reduc* or decreas* or increas* or lower* or fewer or improv* or enhance* or extend* or develop* or prevalen* or daily or weekly or current or rate or rates or behavio?r* or knowledge or prevent* or attitude* or avoid* or perceive* or percept* or promot* or program* or change*)).ti,ab,kw. (546304)

136	or/130-135 (715060)

137	exp academic achievement/ (40173)

138	(education* attain* adj5 (reduc* or decreas* or increas* or lower* or fewer or improv* or enhance* or extend* or develop* or prevalen* or daily or weekly or current or rate or rates or behavio?r* or knowledge or prevent* or attitude* or avoid* or perceive* or percept* or promot* or program* or change*)).ti,ab,kw. (3226)

139	(academic attain* adj5 (reduc* or decreas* or increas* or lower* or fewer or improv* or enhance* or extend* or develop* or prevalen* or daily or weekly or current or rate or rates or behavio?r* or knowledge or prevent* or attitude* or avoid* or perceive* or percept* or promot* or program* or change*)).ti,ab,kw. (85)

140	(student* attain* adj5 (reduc* or decreas* or increas* or lower* or fewer or improv* or enhance* or extend* or develop* or prevalen* or daily or weekly or current or rate or rates or behavio?r* or knowledge or prevent* or attitude* or avoid* or perceive* or percept* or promot* or program* or change*)).ti,ab,kw. (30)

141	(education* achieve* adj5 (reduc* or decreas* or increas* or lower* or fewer or improv* or enhance* or extend* or develop* or prevalen* or daily or weekly or current or rate or rates or behavio?r* or knowledge or prevent* or attitude* or avoid* or perceive* or percept* or promot* or program* or change*)).ti,ab,kw. (548)

142	(academic achieve* adj5 (reduc* or decreas* or increas* or lower* or fewer or improv* or enhance* or extend* or develop* or prevalen* or daily or weekly or current or rate or rates or behavio?r* or knowledge or prevent* or attitude* or avoid* or perceive* or percept* or promot* or program* or change*)).ti,ab,kw. (1878)

143	(student* achieve* adj5 (reduc* or decreas* or increas* or lower* or fewer or improv* or enhance* or extend* or develop* or prevalen* or daily or weekly or current or rate or rates or behavio?r* or knowledge or prevent* or attitude* or avoid* or perceive* or percept* or promot* or program* or change*)).ti,ab,kw. (250)

144	(education* perform* adj5 (reduc* or decreas* or increas* or lower* or fewer or improv* or enhance* or extend* or develop* or prevalen* or daily or weekly or current or rate or rates or behavio?r* or knowledge or prevent* or attitude* or avoid* or perceive* or percept* or promot* or program* or change*)).ti,ab,kw. (156)

145	(academic perform* adj5 (reduc* or decreas* or increas* or lower* or fewer or improv* or enhance* or extend* or develop* or prevalen* or daily or weekly or current or rate or rates or behavio?r* or knowledge or prevent* or attitude* or avoid* or perceive* or percept* or promot* or program* or change*)).ti,ab,kw. (2659)

146	(student* perform* adj5 (reduc* or decreas* or increas* or lower* or fewer or improv* or enhance* or extend* or develop* or prevalen* or daily or weekly or current or rate or rates or behavio?r* or knowledge or prevent* or attitude* or avoid* or perceive* or percept* or promot* or program* or change*)).ti,ab,kw. (1343)

147	(education* outcome? adj5 (reduc* or decreas* or increas* or lower* or fewer or improv* or enhance* or extend* or develop* or prevalen* or daily or weekly or current or rate or rates or behavio?r* or knowledge or prevent* or attitude* or avoid* or perceive* or percept* or promot* or program* or change*)).ti,ab,kw. (828)

148	(academic outcome? adj5 (reduc* or decreas* or increas* or lower* or fewer or improv* or enhance* or extend* or develop* or prevalen* or daily or weekly or current or rate or rates or behavio?r* or knowledge or prevent* or attitude* or avoid* or perceive* or percept* or promot* or program* or change*)).ti,ab,kw. (387)

149	(student* outcome? adj5 (reduc* or decreas* or increas* or lower* or fewer or improv* or enhance* or extend* or develop* or prevalen* or daily or weekly or current or rate or rates or behavio?r* or knowledge or prevent* or attitude* or avoid* or perceive* or percept* or promot* or program* or change*)).ti,ab,kw. (271)

150	(education* commitment adj5 (reduc* or decreas* or increas* or lower* or fewer or improv* or enhance* or extend* or develop* or prevalen* or daily or weekly or current or rate or rates or behavio?r* or knowledge or prevent* or attitude* or avoid* or perceive* or percept* or promot* or program* or change*)).ti,ab,kw. (6)

151	(academic commitment adj5 (reduc* or decreas* or increas* or lower* or fewer or improv* or enhance* or extend* or develop* or prevalen* or daily or weekly or current or rate or rates or behavio?r* or knowledge or prevent* or attitude* or avoid* or perceive* or percept* or promot* or program* or change*)).ti,ab,kw. (10)

152	(student* commitment adj5 (reduc* or decreas* or increas* or lower* or fewer or improv* or enhance* or extend* or develop* or prevalen* or daily or weekly or current or rate or rates or behavio?r* or knowledge or prevent* or attitude* or avoid* or perceive* or percept* or promot* or program* or change*)).ti,ab,kw. (21)

153	(education* engage* adj5 (reduc* or decreas* or increas* or lower* or fewer or improv* or enhance* or extend* or develop* or prevalen* or daily or weekly or current or rate or rates or behavio?r* or knowledge or prevent* or attitude* or avoid* or perceive* or percept* or promot* or program* or change*)).ti,ab,kw. (29)

154	(academic engage* adj5 (reduc* or decreas* or increas* or lower* or fewer or improv* or enhance* or extend* or develop* or prevalen* or daily or weekly or current or rate or rates or behavio?r* or knowledge or prevent* or attitude* or avoid* or perceive* or percept* or promot* or program* or change*)).ti,ab,kw. (103)

155	(student* engage* adj5 (reduc* or decreas* or increas* or lower* or fewer or improv* or enhance* or extend* or develop* or prevalen* or daily or weekly or current or rate or rates or behavio?r* or knowledge or prevent* or attitude* or avoid* or perceive* or percept* or promot* or program* or change*)).ti,ab,kw. (819)

156	(dropout adj5 (reduc* or decreas* or increas* or lower* or fewer or improv* or enhance* or extend* or develop* or prevalen* or daily or weekly or current or rate or rates or behavio?r* or knowledge or prevent* or attitude* or avoid* or perceive* or percept* or promot* or program* or change*)).ti,ab,kw. (6539)

157	(absentee* adj5 (reduc* or decreas* or increas* or lower* or fewer or improv* or enhance* or extend* or develop* or prevalen* or daily or weekly or current or rate or rates or behavio?r* or knowledge or prevent* or attitude* or avoid* or perceive* or percept* or promot* or program* or change*)).ti,ab,kw. (3512)

158	or/137-157 (58859)

159	violence/ (49807)

160	assault/ (7493)

161	exposure to violence/ (1857)

162	gun violence/ (501)

163	physical violence/ (2223)

164	verbal hostility/ (2276)

165	aggression/ (62721)

166	aggressiveness/ (12544)

167	hostility/ (11868)

168	physical abuse/ (6104)

169	gender based violence/ (1052)

170	exp bullying/ (8385)

171	exp delinquency/ (13254)

172	disruptive behavior/ (3009)

173	antisocial behavior/ (8096)

174	conflict/ (26894)

175	or/159-174 (191798)

176	104 and 175 (11107)

177	((violent or violence) adj5 (reduc* or decreas* or increas* or lower* or fewer or improv* or enhance* or extend* or develop* or prevalen* or daily or weekly or current or rate or rates or behavio?r* or knowledge or prevent* or attitude* or avoid* or perceive* or percept* or promot* or program* or change*)).ti,ab,kw. (26535)

178	((aggression or aggressive) adj5 (reduc* or decreas* or increas* or lower* or fewer or improv* or enhance* or extend* or develop* or prevalen* or daily or weekly or current or rate or rates or behavio?r* or knowledge or prevent* or attitude* or avoid* or perceive* or percept* or promot* or program* or change*)).ti,ab,kw. (69031)

179	((hostile or hostility) adj5 (reduc* or decreas* or increas* or lower* or fewer or improv* or enhance* or extend* or develop* or prevalen* or daily or weekly or current or rate or rates or behavio?r* or knowledge or prevent* or attitude* or avoid* or perceive* or percept* or promot* or program* or change*)).ti,ab,kw. (3881)

180	(assault* adj5 (reduc* or decreas* or increas* or lower* or fewer or improv* or enhance* or extend* or develop* or prevalen* or daily or weekly or current or rate or rates or behavio?r* or knowledge or prevent* or attitude* or avoid* or perceive* or percept* or promot* or program* or change*)).ti,ab,kw. (3637)

181	(crime adj5 (reduc* or decreas* or increas* or lower* or fewer or improv* or enhance* or extend* or develop* or prevalen* or daily or weekly or current or rate or rates or behavio?r* or knowledge or prevent* or attitude* or avoid* or perceive* or percept* or promot* or program* or change*)).ti,ab,kw. (4045)

182	((abuse? or abusive) adj5 (reduc* or decreas* or increas* or lower* or fewer or improv* or enhance* or extend* or develop* or prevalen* or daily or weekly or current or rate or rates or behavio?r* or knowledge or prevent* or attitude* or avoid* or perceive* or percept* or promot* or program* or change*)).ti,ab,kw. (39190)

183	(misbehav* adj5 (reduc* or decreas* or increas* or lower* or fewer or improv* or enhance* or extend* or develop* or prevalen* or daily or weekly or current or rate or rates or behavio?r* or knowledge or prevent* or attitude* or avoid* or perceive* or percept* or promot* or program* or change*)).ti,ab,kw. (175)

184	(threat? adj5 (reduc* or decreas* or increas* or lower* or fewer or improv* or enhance* or extend* or develop* or prevalen* or daily or weekly or current or rate or rates or behavio?r* or knowledge or prevent* or attitude* or avoid* or perceive* or percept* or promot* or program* or change*)).ti,ab,kw. (17833)

185	(conduct adj5 (reduc* or decreas* or increas* or lower* or fewer or improv* or enhance* or extend* or develop* or prevalen* or daily or weekly or current or rate or rates or behavio?r* or knowledge or prevent* or attitude* or avoid* or perceive* or percept* or promot* or program* or change*)).ti,ab,kw. (12622)

186	(delinquen* adj5 (reduc* or decreas* or increas* or lower* or fewer or improv* or enhance* or extend* or develop* or prevalen* or daily or weekly or current or rate or rates or behavio?r* or knowledge or prevent* or attitude* or avoid* or perceive* or percept* or promot* or program* or change*)).ti,ab,kw. (3889)

187	(disrupt* behavio?r* adj5 (reduc* or decreas* or increas* or lower* or fewer or improv* or enhance* or extend* or develop* or prevalen* or daily or weekly or current or rate or rates or behavio?r* or knowledge or prevent* or attitude* or avoid* or perceive* or percept* or promot* or program* or change*)).ti,ab,kw. (5136)

188	(problem* behavio?r* adj5 (reduc* or decreas* or increas* or lower* or fewer or improv* or enhance* or extend* or develop* or prevalen* or daily or weekly or current or rate or rates or behavio?r* or knowledge or prevent* or attitude* or avoid* or perceive* or percept* or promot* or program* or change*)).ti,ab,kw. (7477)

189	(volatile behavio?r* adj5 (reduc* or decreas* or increas* or lower* or fewer or improv* or enhance* or extend* or develop* or prevalen* or daily or weekly or current or rate or rates or behavio?r* or knowledge or prevent* or attitude* or avoid* or perceive* or percept* or promot* or program* or change*)).ti,ab,kw. (32)

190	((antisocial or anti-social) adj5 (reduc* or decreas* or increas* or lower* or fewer or improv* or enhance* or extend* or develop* or prevalen* or daily or weekly or current or rate or rates or behavio?r* or knowledge or prevent* or attitude* or avoid* or perceive* or percept* or promot* or program* or change*)).ti,ab,kw. (7123)

191	(perpetrat* adj5 (reduc* or decreas* or increas* or lower* or fewer or improv* or enhance* or extend* or develop* or prevalen* or daily or weekly or current or rate or rates or behavio?r* or knowledge or prevent* or attitude* or avoid* or perceive* or percept* or promot* or program* or change*)).ti,ab,kw. (2497)

192	(bully* adj5 (reduc* or decreas* or increas* or lower* or fewer or improv* or enhance* or extend* or develop* or prevalen* or daily or weekly or current or rate or rates or behavio?r* or knowledge or prevent* or attitude* or avoid* or perceive* or percept* or promot* or program* or change*)).ti,ab,kw. (3463)

193	(victim* adj5 (reduc* or decreas* or increas* or lower* or fewer or improv* or enhance* or extend* or develop* or prevalen* or daily or weekly or current or rate or rates or behavio?r* or knowledge or prevent* or attitude* or avoid* or perceive* or percept* or promot* or program* or change*)).ti,ab,kw. (11526)

194	(cyberbully* adj5 (reduc* or decreas* or increas* or lower* or fewer or improv* or enhance* or extend* or develop* or prevalen* or daily or weekly or current or rate or rates or behavio?r* or knowledge or prevent* or attitude* or avoid* or perceive* or percept* or promot* or program* or change*)).ti,ab,kw. (476)

195	(conflict* adj5 (reduc* or decreas* or increas* or lower* or fewer or improv* or enhance* or extend* or develop* or prevalen* or daily or weekly or current or rate or rates or behavio?r* or knowledge or prevent* or attitude* or avoid* or perceive* or percept* or promot* or program* or change*)).ti,ab,kw. (23248)

196	(positive behavio?r* adj5 (reduc* or decreas* or increas* or lower* or fewer or improv* or enhance* or extend* or develop* or prevalen* or daily or weekly or current or rate or rates or behavio?r* or knowledge or prevent* or attitude* or avoid* or perceive* or percept* or promot* or program* or change*)).ti,ab,kw. (2275)

197	(improv* behavio?r* adj5 (reduc* or decreas* or increas* or lower* or fewer or improv* or enhance* or extend* or develop* or prevalen* or daily or weekly or current or rate or rates or behavio?r* or knowledge or prevent* or attitude* or avoid* or perceive* or percept* or promot* or program* or change*)).ti,ab,kw. (3248)

198	(social behavio?r* adj5 (reduc* or decreas* or increas* or lower* or fewer or improv* or enhance* or extend* or develop* or prevalen* or daily or weekly or current or rate or rates or behavio?r* or knowledge or prevent* or attitude* or avoid* or perceive* or percept* or promot* or program* or change*)).ti,ab,kw. (19703)

199	((social-emotion* or socio-emotion*) adj5 (reduc* or decreas* or increas* or lower* or fewer or improv* or enhance* or extend* or develop* or prevalen* or daily or weekly or current or rate or rates or behavio?r* or knowledge or prevent* or attitude* or avoid* or perceive* or percept* or promot* or program* or change*)).ti,ab,kw. (2954)

200	(social-charact* adj5 (reduc* or decreas* or increas* or lower* or fewer or improv* or enhance* or extend* or develop* or prevalen* or daily or weekly or current or rate or rates or behavio?r* or knowledge or prevent* or attitude* or avoid* or perceive* or percept* or promot* or program* or change*)).ti,ab,kw. (310)

201	(social-inclusion adj5 (reduc* or decreas* or increas* or lower* or fewer or improv* or enhance* or extend* or develop* or prevalen* or daily or weekly or current or rate or rates or behavio?r* or knowledge or prevent* or attitude* or avoid* or perceive* or percept* or promot* or program* or change*)).ti,ab,kw. (624)

202	(social develop* adj5 (reduc* or decreas* or increas* or lower* or fewer or improv* or enhance* or extend* or develop* or prevalen* or daily or weekly or current or rate or rates or behavio?r* or knowledge or prevent* or attitude* or avoid* or perceive* or percept* or promot* or program* or change*)).ti,ab,kw. (4718)

203	(social competen* adj5 (reduc* or decreas* or increas* or lower* or fewer or improv* or enhance* or extend* or develop* or prevalen* or daily or weekly or current or rate or rates or behavio?r* or knowledge or prevent* or attitude* or avoid* or perceive* or percept* or promot* or program* or change*)).ti,ab,kw. (1602)

204	(emotion* develop* adj5 (reduc* or decreas* or increas* or lower* or fewer or improv* or enhance* or extend* or develop* or prevalen* or daily or weekly or current or rate or rates or behavio?r* or knowledge or prevent* or attitude* or avoid* or perceive* or percept* or promot* or program* or change*)).ti,ab,kw. (2702)

205	(conduct adj5 (reduc* or decreas* or increas* or lower* or fewer or improv* or enhance* or extend* or develop* or prevalen* or daily or weekly or current or rate or rates or behavio?r* or knowledge or prevent* or attitude* or avoid* or perceive* or percept* or promot* or program* or change*)).ti,ab,kw. (12622)

206	(respect adj5 (reduc* or decreas* or increas* or lower* or fewer or improv* or enhance* or extend* or develop* or prevalen* or daily or weekly or current or rate or rates or behavio?r* or knowledge or prevent* or attitude* or avoid* or perceive* or percept* or promot* or program* or change*)).ti,ab,kw. (54165)

207	((safety or safe) adj5 (reduc* or decreas* or increas* or lower* or fewer or improv* or enhance* or extend* or develop* or prevalen* or daily or weekly or current or rate or rates or behavio?r* or knowledge or prevent* or attitude* or avoid* or perceive* or percept* or promot* or program* or change*)).ti,ab,kw. (206993)

208	or/176-207 (511061)

209	exp program evaluation/ (27748)

210	evaluation study/ (49432)

211	validation study/ (89973)

212	exp clinical trial/ (1633754)

213	prevent*.ti,ab,kw. (2088918)

214	program*.ti,ab,kw. (1276861)

215	intervent*.ti,ab,kw. (1566787)

216	restorative approach*.ti,ab,kw. (240)

217	control school?.ti,ab,kw. (1077)

218	(trial or trials).ti,ab,kw. (1620270)

219	or/209-218 (6413007)

220	121 or 136 or 158 or 208 (1404615)

221	52 and 100 and 219 and 220 (12630)

## OvidSP Global Health

| Database name | Global Health |
| --- | --- |
| Database platform | OvidSP |
| Dates of database coverage | 1910 to 2021 Week 18 |
| Date searched | 11/05/2021 |
| Searched by | JF |
| Number of results | 3988 |
| EndNote import order | 5 |
| Number of results once duplicates removed | 455 |
| Search strategy notes | Search lines ending in a ‘/’ are subject heading searches. Search lines ending in ‘/*ab*’ are subject headings with subheadings added. The subheading is listed in square brackets at the end of the line. Search lines beginning ‘exp’ are exploded subject heading searches. Search lines ending in .ti,ab. search in the title and abstract only. Search lines ending in .jx. search in the journal title only. or/*x-y* combines search sets in the range *x-y* with Boolean operator OR. * is used for truncation of words. # is used for a compulsory wildcard. ? is used for an optional wildcard. adj*n* searches for terms within *n* words or each other. Words in square brackets [ ] are comments and are not included in the search terms. |

1	children/ (385429)

2	boys/ (25915)

3	girls/ (31911)

4	school children/ (32085)

5	adolescents/ (69936)

6	youth/ (18927)

7	students/ (24642)

8	high school students/ (4948)

9	junior high school students/ (272)

10	pupils/ (119)

11	teachers/ (3069)

12	parents/ (9920)

13	fathers/ (1653)

14	mothers/ (38506)

15	child*.ti,ab. (388469)

16	boy?.ti,ab. (44580)

17	girl?.ti,ab. (47901)

18	(schoolage or (school adj1 age)).ti,ab. (5157)

19	schoolchild*.ti,ab. (11149)

20	youngster?.ti,ab. (563)

21	minor?.ti,ab. (34959)

22	(pupil* or student*).ti,ab. not (exp eyes/ or students/) (49359)

23	prepubescen*.ti,ab. (154)

24	pubescent*.ti,ab. (244)

25	adolescent*.ti,ab. (65049)

26	juvenil*.ti,ab. (9467)

27	underage*.ti,ab. (566)

28	(preteen* or pre-teen*).ti,ab. (136)

29	(teen or teens).ti,ab. (2912)

30	teenage*.ti,ab. (5649)

31	(youth or youths).ti,ab. (23446)

32	young person*.ti,ab. (1051)

33	young people*.ti,ab. (9956)

34	(kid or kids).ti,ab. (2585)

35	(transition adj4 adult*).ti,ab. (699)

36	emerging adult*.ti,ab. (842)

37	young adult.ti,ab. (6036)

38	parent?.ti,ab. (55025)

39	mother?.ti,ab. (84833)

40	father?.ti,ab. (8944)

41	guardian?.ti,ab. (2267)

42	teacher?.ti,ab. (12662)

43	child*.jx. (25717)

44	adolescen*.jx. (7110)

45	youth.jx. (502)

46	school*.jx. (4007)

47	(("5" or "6" or "7" or "8" or "9" or "10" or "11" or "12" or "13" or "14" or "15" or "16" or "17" or "18") adj (year* old or year* of age)).ti,ab. (68822)

48	((five or six or seven or eight or nine or ten or eleven or twelve or thirteen or fourteen or fifteen or sixteen or seventeen or eighteen) adj (year* old or year* of age)).ti,ab. (5161)

49	(age* adj ("5" or "6" or "7" or "8" or "9" or "10" or "11" or "12" or "13" or "14" or "15" or "16" or "17" or "18") adj year*).ti,ab. (20342)

50	(age* adj (five or six or seven or eight or nine or ten or eleven or twelve or thirteen or fourteen or fifteen or sixteen or seventeen or eighteen) adj year*).ti,ab. (309)

51	((primary or secondary or year) adj1 ("1" or "2" or "3" or "4" or "5" or "6" or "7" or "8" or "9" or "10" or "11" or "12" or "13")).ti,ab. (103129)

52	(grade? adj1 (first or second or third or fourth or fifth or sixth or seventh or eighth or ninth or tenth or eleventh or twelfth)).ti,ab. (3429)

53	or/1-52 [POPULATION] (724488)

54	schools/ (19135)

55	elementary schools/ (920)

56	high schools/ (663)

57	private schools/ (347)

58	public schools/ (1057)

59	school health services/ (265)

60	student teacher relationships/ (107)

61	exp management/ or exp administration/ (73065)

62	or/54-59 (21471)

63	61 and 62 (426)

64	(school? adj5 (based or level or wide or led or ethos or environment or organi?ation* or climate or toolkit? or approach* or action group? or practice? or whole or health* or leader* or cultur* or manag* or governance or system? or communit*)).ti,ab. (26741)

65	schoolwide.ti,ab. (23)

66	restor* justice.ti,ab. (16)

67	school?.ti,ab. (101084)

68	or/54-58,67 (101488)

69	"peer-to-peer".ti,ab. (242)

70	peer relationships/ or peer influence/ (3051)

71	(peer adj2 (led or leader* or participat* or mediat* or helper? or resistan* or wide or action? or influen*)).ti,ab. (1337)

72	(relationship* adj2 (led or leader* or participat* or mediat* or helper? or resistan* or wide or action? or influen*)).ti,ab. (3079)

73	community involvement/ (4385)

74	((community or communities) adj2 (led or leader* or participat* or mediat* or helper? or resistan* or wide or action? or influen* or relationship*)).ti,ab. (9771)

75	(pupil? adj2 (led or leader* or participat* or mediat* or helper? or resistan* or wide or action? or influen* or relationship*)).ti,ab. not exp eyes/ (132)

76	student participation/ (54)

77	((child or children) adj2 (led or leader* or participat* or mediat* or helper? or resistan* or wide or action? or influen*)).ti,ab. (5064)

78	((young people or young person) adj2 (led or leader* or participat* or mediat* or helper? or resistan* or wide or action? or influen*)).ti,ab. (129)

79	(teen* adj2 (led or leader* or participat* or mediat* or helper? or resistan* or wide or action? or influen*)).ti,ab. (135)

80	(adolescent* adj2 (led or leader* or participat* or mediat* or helper? or resistan* or wide or action? or influen*)).ti,ab. (1673)

81	(youth? adj2 (led or leader* or participat* or mediat* or helper? or resistan* or wide or action? or influen*)).ti,ab. (780)

82	(student? adj2 (led or leader* or participat* or mediat* or helper? or resistan* or wide or action? or influen*)).ti,ab. (2720)

83	(teach* adj2 (led or leader* or participat* or mediat* or helper? or resistan* or wide or action? or influen* or relationship*)).ti,ab. (757)

84	(curricul* adj2 (led or leader* or participat* or mediat* or helper? or resistan* or wide or action? or influen* or relationship*)).ti,ab. (101)

85	(administrat* adj2 (led or leader* or participat* or mediat* or helper? or resistan* or wide or action? or influen* or relationship*)).ti,ab. (1017)

86	((personnel or staff) adj2 (led or leader* or participat* or mediat* or helper? or resistan* or wide or action? or influen* or relationship*)).ti,ab. (859)

87	(volunteer* adj2 (led or leader* or participat* or mediat* or helper? or resistan* or wide or action? or influen* or relationship*)).ti,ab. (1180)

88	((advocacy or advocate) adj2 (led or leader* or participat* or mediat* or helper? or resistan* or wide or action? or influen* or relationship*)).ti,ab. (220)

89	(parent* adj2 (led or leader* or participat* or mediat* or helper? or resistan* or wide or action?)).ti,ab. (1245)

90	parent participation/ (72)

91	social environment/ (1704)

92	(environment* adj2 (led or leader* or participat* or mediat* or helper? or resistan* or wide or action? or influen* or relationship*)).ti,ab. (7315)

93	(socio-ecolog* adj2 (led or leader* or participat* or mediat* or helper? or resistan* or wide or action? or influen* or relationship*)).ti,ab. (22)

94	(learn* adj2 (led or leader* or participat* or mediat* or helper? or resistan* or wide or action? or influen* or relationship*)).ti,ab. (664)

95	educational policy/ (221)

96	((policy or policies) adj2 (led or leader* or participat* or mediat* or helper? or resistan* or wide or action? or influen* or relationship*)).ti,ab. (2858)

97	or/69-96 (45509)

98	68 and 97 (7894)

99	or/63-66,98 [SCHOOL WIDE] (31419)

100	exp prevention/ (154042)

101	smoking cessation/ or tobacco control/ (6893)

102	tobacco smoking/ (54293)

103	vaping/ (445)

104	(102 or 103) and 100 (2124)

105	((smoking or smoked or smoke or smoker) adj5 (reduc* or decreas* or increas* or lower* or fewer or improv* or enhance* or extend* or develop* or prevalen* or daily or weekly or current or rate or rates or behavio?r* or knowledge or prevent* or attitude* or avoid* or perceive* or percept* or promot* or program* or change*)).ti,ab. (30853)

106	(tobacco adj5 (reduc* or decreas* or increas* or lower* or fewer or improv* or enhance* or extend* or develop* or prevalen* or daily or weekly or current or rate or rates or behavio?r* or knowledge or prevent* or attitude* or avoid* or perceive* or percept* or promot* or program* or change*)).ti,ab. (11101)

107	(cigarette? adj5 (reduc* or decreas* or increas* or lower* or fewer or improv* or enhance* or extend* or develop* or prevalen* or daily or weekly or current or rate or rates or behavio?r* or knowledge or prevent* or attitude* or avoid* or perceive* or percept* or promot* or program* or change*)).ti,ab. (8477)

108	(marijuana adj5 (reduc* or decreas* or increas* or lower* or fewer or improv* or enhance* or extend* or develop* or prevalen* or daily or weekly or current or rate or rates or behavio?r* or knowledge or prevent* or attitude* or avoid* or perceive* or percept* or promot* or program* or change*)).ti,ab. (1323)

109	(cannabis adj5 (reduc* or decreas* or increas* or lower* or fewer or improv* or enhance* or extend* or develop* or prevalen* or daily or weekly or current or rate or rates or behavio?r* or knowledge or prevent* or attitude* or avoid* or perceive* or percept* or promot* or program* or change*)).ti,ab. (1570)

110	(solvent? adj5 (reduc* or decreas* or increas* or lower* or fewer or improv* or enhance* or extend* or develop* or prevalen* or daily or weekly or current or rate or rates or behavio?r* or knowledge or prevent* or attitude* or avoid* or perceive* or percept* or promot* or program* or change*)).ti,ab. (3057)

111	or/101,104-110 [SMOKING] (45664)

112	exp substance abuse/ (28339)

113	alcohol intake/ (31262)

114	alcoholism/ (11159)

115	exp addiction/ (7859)

116	(or/112-115) and 100 (3630)

117	(alcohol adj5 (reduc* or decreas* or increas* or lower* or fewer or improv* or enhance* or extend* or develop* or prevalen* or daily or weekly or current or rate or rates or behavio?r* or knowledge or prevent* or attitude* or avoid* or perceive* or percept* or promot* or program* or change*)).ti,ab. (21510)

118	(binge drink* adj5 (reduc* or decreas* or increas* or lower* or fewer or improv* or enhance* or extend* or develop* or prevalen* or daily or weekly or current or rate or rates or behavio?r* or knowledge or prevent* or attitude* or avoid* or perceive* or percept* or promot* or program* or change*)).ti,ab. (1019)

119	(underage drink* adj5 (reduc* or decreas* or increas* or lower* or fewer or improv* or enhance* or extend* or develop* or prevalen* or daily or weekly or current or rate or rates or behavio?r* or knowledge or prevent* or attitude* or avoid* or perceive* or percept* or promot* or program* or change*)).ti,ab. (122)

120	(substance? adj5 (reduc* or decreas* or increas* or lower* or fewer or improv* or enhance* or extend* or develop* or prevalen* or daily or weekly or current or rate or rates or behavio?r* or knowledge or prevent* or attitude* or avoid* or perceive* or percept* or promot* or program* or change*)).ti,ab. (14790)

121	(drug? adj5 (reduc* or decreas* or increas* or lower* or fewer or improv* or enhance* or extend* or develop* or prevalen* or daily or weekly or current or rate or rates or behavio?r* or knowledge or prevent* or attitude* or avoid* or perceive* or percept* or promot* or program* or change*)).ti,ab. (66331)

122	or/116-121 [DRUG ABUSE] (101258)

123	exp educational performance/ (4112)

124	educational attendance/ (1407)

125	(education* attain* adj5 (reduc* or decreas* or increas* or lower* or fewer or improv* or enhance* or extend* or develop* or prevalen* or daily or weekly or current or rate or rates or behavio?r* or knowledge or prevent* or attitude* or avoid* or perceive* or percept* or promot* or program* or change*)).ti,ab. (1096)

126	(academic attain* adj5 (reduc* or decreas* or increas* or lower* or fewer or improv* or enhance* or extend* or develop* or prevalen* or daily or weekly or current or rate or rates or behavio?r* or knowledge or prevent* or attitude* or avoid* or perceive* or percept* or promot* or program* or change*)).ti,ab. (16)

127	(student* attain* adj5 (reduc* or decreas* or increas* or lower* or fewer or improv* or enhance* or extend* or develop* or prevalen* or daily or weekly or current or rate or rates or behavio?r* or knowledge or prevent* or attitude* or avoid* or perceive* or percept* or promot* or program* or change*)).ti,ab. (3)

128	(education* achieve* adj5 (reduc* or decreas* or increas* or lower* or fewer or improv* or enhance* or extend* or develop* or prevalen* or daily or weekly or current or rate or rates or behavio?r* or knowledge or prevent* or attitude* or avoid* or perceive* or percept* or promot* or program* or change*)).ti,ab. (123)

129	(academic achieve* adj5 (reduc* or decreas* or increas* or lower* or fewer or improv* or enhance* or extend* or develop* or prevalen* or daily or weekly or current or rate or rates or behavio?r* or knowledge or prevent* or attitude* or avoid* or perceive* or percept* or promot* or program* or change*)).ti,ab. (295)

130	(student* achieve* adj5 (reduc* or decreas* or increas* or lower* or fewer or improv* or enhance* or extend* or develop* or prevalen* or daily or weekly or current or rate or rates or behavio?r* or knowledge or prevent* or attitude* or avoid* or perceive* or percept* or promot* or program* or change*)).ti,ab. (28)

131	(education* perform* adj5 (reduc* or decreas* or increas* or lower* or fewer or improv* or enhance* or extend* or develop* or prevalen* or daily or weekly or current or rate or rates or behavio?r* or knowledge or prevent* or attitude* or avoid* or perceive* or percept* or promot* or program* or change*)).ti,ab. (33)

132	(academic perform* adj5 (reduc* or decreas* or increas* or lower* or fewer or improv* or enhance* or extend* or develop* or prevalen* or daily or weekly or current or rate or rates or behavio?r* or knowledge or prevent* or attitude* or avoid* or perceive* or percept* or promot* or program* or change*)).ti,ab. (420)

133	(student* perform* adj5 (reduc* or decreas* or increas* or lower* or fewer or improv* or enhance* or extend* or develop* or prevalen* or daily or weekly or current or rate or rates or behavio?r* or knowledge or prevent* or attitude* or avoid* or perceive* or percept* or promot* or program* or change*)).ti,ab. (68)

134	(education* outcome? adj5 (reduc* or decreas* or increas* or lower* or fewer or improv* or enhance* or extend* or develop* or prevalen* or daily or weekly or current or rate or rates or behavio?r* or knowledge or prevent* or attitude* or avoid* or perceive* or percept* or promot* or program* or change*)).ti,ab. (137)

135	(academic outcome? adj5 (reduc* or decreas* or increas* or lower* or fewer or improv* or enhance* or extend* or develop* or prevalen* or daily or weekly or current or rate or rates or behavio?r* or knowledge or prevent* or attitude* or avoid* or perceive* or percept* or promot* or program* or change*)).ti,ab. (54)

136	(student* outcome? adj5 (reduc* or decreas* or increas* or lower* or fewer or improv* or enhance* or extend* or develop* or prevalen* or daily or weekly or current or rate or rates or behavio?r* or knowledge or prevent* or attitude* or avoid* or perceive* or percept* or promot* or program* or change*)).ti,ab. (32)

137	(education* commitment adj5 (reduc* or decreas* or increas* or lower* or fewer or improv* or enhance* or extend* or develop* or prevalen* or daily or weekly or current or rate or rates or behavio?r* or knowledge or prevent* or attitude* or avoid* or perceive* or percept* or promot* or program* or change*)).ti,ab. (2)

138	(academic commitment adj5 (reduc* or decreas* or increas* or lower* or fewer or improv* or enhance* or extend* or develop* or prevalen* or daily or weekly or current or rate or rates or behavio?r* or knowledge or prevent* or attitude* or avoid* or perceive* or percept* or promot* or program* or change*)).ti,ab. (1)

139	(student* commitment adj5 (reduc* or decreas* or increas* or lower* or fewer or improv* or enhance* or extend* or develop* or prevalen* or daily or weekly or current or rate or rates or behavio?r* or knowledge or prevent* or attitude* or avoid* or perceive* or percept* or promot* or program* or change*)).ti,ab. (2)

140	(education* engage* adj5 (reduc* or decreas* or increas* or lower* or fewer or improv* or enhance* or extend* or develop* or prevalen* or daily or weekly or current or rate or rates or behavio?r* or knowledge or prevent* or attitude* or avoid* or perceive* or percept* or promot* or program* or change*)).ti,ab. (8)

141	(academic engage* adj5 (reduc* or decreas* or increas* or lower* or fewer or improv* or enhance* or extend* or develop* or prevalen* or daily or weekly or current or rate or rates or behavio?r* or knowledge or prevent* or attitude* or avoid* or perceive* or percept* or promot* or program* or change*)).ti,ab. (11)

142	(student* engage* adj5 (reduc* or decreas* or increas* or lower* or fewer or improv* or enhance* or extend* or develop* or prevalen* or daily or weekly or current or rate or rates or behavio?r* or knowledge or prevent* or attitude* or avoid* or perceive* or percept* or promot* or program* or change*)).ti,ab. (111)

143	(dropout adj5 (reduc* or decreas* or increas* or lower* or fewer or improv* or enhance* or extend* or develop* or prevalen* or daily or weekly or current or rate or rates or behavio?r* or knowledge or prevent* or attitude* or avoid* or perceive* or percept* or promot* or program* or change*)).ti,ab. (710)

144	(absentee* adj5 (reduc* or decreas* or increas* or lower* or fewer or improv* or enhance* or extend* or develop* or prevalen* or daily or weekly or current or rate or rates or behavio?r* or knowledge or prevent* or attitude* or avoid* or perceive* or percept* or promot* or program* or change*)).ti,ab. (756)

145	or/123-144 [ACADEMIC PERFORMANCE] (8297)

146	aggressive behaviour/ (12963)

147	aggression/ (1006)

148	fighting/ (98)

149	(or/146-148) and 100 (1180)

150	((violent or violence) adj5 (reduc* or decreas* or increas* or lower* or fewer or improv* or enhance* or extend* or develop* or prevalen* or daily or weekly or current or rate or rates or behavio?r* or knowledge or prevent* or attitude* or avoid* or perceive* or percept* or promot* or program* or change*)).ti,ab. (5923)

151	((aggression or aggressive) adj5 (reduc* or decreas* or increas* or lower* or fewer or improv* or enhance* or extend* or develop* or prevalen* or daily or weekly or current or rate or rates or behavio?r* or knowledge or prevent* or attitude* or avoid* or perceive* or percept* or promot* or program* or change*)).ti,ab. (3529)

152	((hostile or hostility) adj5 (reduc* or decreas* or increas* or lower* or fewer or improv* or enhance* or extend* or develop* or prevalen* or daily or weekly or current or rate or rates or behavio?r* or knowledge or prevent* or attitude* or avoid* or perceive* or percept* or promot* or program* or change*)).ti,ab. (314)

153	(assault* adj5 (reduc* or decreas* or increas* or lower* or fewer or improv* or enhance* or extend* or develop* or prevalen* or daily or weekly or current or rate or rates or behavio?r* or knowledge or prevent* or attitude* or avoid* or perceive* or percept* or promot* or program* or change*)).ti,ab. (488)

154	(crime adj5 (reduc* or decreas* or increas* or lower* or fewer or improv* or enhance* or extend* or develop* or prevalen* or daily or weekly or current or rate or rates or behavio?r* or knowledge or prevent* or attitude* or avoid* or perceive* or percept* or promot* or program* or change*)).ti,ab. (766)

155	((abuse? or abusive) adj5 (reduc* or decreas* or increas* or lower* or fewer or improv* or enhance* or extend* or develop* or prevalen* or daily or weekly or current or rate or rates or behavio?r* or knowledge or prevent* or attitude* or avoid* or perceive* or percept* or promot* or program* or change*)).ti,ab. (5037)

156	(misbehav* adj5 (reduc* or decreas* or increas* or lower* or fewer or improv* or enhance* or extend* or develop* or prevalen* or daily or weekly or current or rate or rates or behavio?r* or knowledge or prevent* or attitude* or avoid* or perceive* or percept* or promot* or program* or change*)).ti,ab. (21)

157	(threat? adj5 (reduc* or decreas* or increas* or lower* or fewer or improv* or enhance* or extend* or develop* or prevalen* or daily or weekly or current or rate or rates or behavio?r* or knowledge or prevent* or attitude* or avoid* or perceive* or percept* or promot* or program* or change*)).ti,ab. (4259)

158	(conduct adj5 (reduc* or decreas* or increas* or lower* or fewer or improv* or enhance* or extend* or develop* or prevalen* or daily or weekly or current or rate or rates or behavio?r* or knowledge or prevent* or attitude* or avoid* or perceive* or percept* or promot* or program* or change*)).ti,ab. (1940)

159	(delinquen* adj5 (reduc* or decreas* or increas* or lower* or fewer or improv* or enhance* or extend* or develop* or prevalen* or daily or weekly or current or rate or rates or behavio?r* or knowledge or prevent* or attitude* or avoid* or perceive* or percept* or promot* or program* or change*)).ti,ab. (472)

160	(disrupt* behavio?r* adj5 (reduc* or decreas* or increas* or lower* or fewer or improv* or enhance* or extend* or develop* or prevalen* or daily or weekly or current or rate or rates or behavio?r* or knowledge or prevent* or attitude* or avoid* or perceive* or percept* or promot* or program* or change*)).ti,ab. (234)

161	(problem* behavio?r* adj5 (reduc* or decreas* or increas* or lower* or fewer or improv* or enhance* or extend* or develop* or prevalen* or daily or weekly or current or rate or rates or behavio?r* or knowledge or prevent* or attitude* or avoid* or perceive* or percept* or promot* or program* or change*)).ti,ab. (722)

162	(volatile behavio?r* adj5 (reduc* or decreas* or increas* or lower* or fewer or improv* or enhance* or extend* or develop* or prevalen* or daily or weekly or current or rate or rates or behavio?r* or knowledge or prevent* or attitude* or avoid* or perceive* or percept* or promot* or program* or change*)).ti,ab. (4)

163	((antisocial or anti-social) adj5 (reduc* or decreas* or increas* or lower* or fewer or improv* or enhance* or extend* or develop* or prevalen* or daily or weekly or current or rate or rates or behavio?r* or knowledge or prevent* or attitude* or avoid* or perceive* or percept* or promot* or program* or change*)).ti,ab. (566)

164	(perpetrat* adj5 (reduc* or decreas* or increas* or lower* or fewer or improv* or enhance* or extend* or develop* or prevalen* or daily or weekly or current or rate or rates or behavio?r* or knowledge or prevent* or attitude* or avoid* or perceive* or percept* or promot* or program* or change*)).ti,ab. (514)

165	(bully* adj5 (reduc* or decreas* or increas* or lower* or fewer or improv* or enhance* or extend* or develop* or prevalen* or daily or weekly or current or rate or rates or behavio?r* or knowledge or prevent* or attitude* or avoid* or perceive* or percept* or promot* or program* or change*)).ti,ab. (746)

166	(victim* adj5 (reduc* or decreas* or increas* or lower* or fewer or improv* or enhance* or extend* or develop* or prevalen* or daily or weekly or current or rate or rates or behavio?r* or knowledge or prevent* or attitude* or avoid* or perceive* or percept* or promot* or program* or change*)).ti,ab. (1826)

167	(cyberbully* adj5 (reduc* or decreas* or increas* or lower* or fewer or improv* or enhance* or extend* or develop* or prevalen* or daily or weekly or current or rate or rates or behavio?r* or knowledge or prevent* or attitude* or avoid* or perceive* or percept* or promot* or program* or change*)).ti,ab. (86)

168	(conflict* adj5 (reduc* or decreas* or increas* or lower* or fewer or improv* or enhance* or extend* or develop* or prevalen* or daily or weekly or current or rate or rates or behavio?r* or knowledge or prevent* or attitude* or avoid* or perceive* or percept* or promot* or program* or change*)).ti,ab. (2677)

169	(positive behavio?r* adj5 (reduc* or decreas* or increas* or lower* or fewer or improv* or enhance* or extend* or develop* or prevalen* or daily or weekly or current or rate or rates or behavio?r* or knowledge or prevent* or attitude* or avoid* or perceive* or percept* or promot* or program* or change*)).ti,ab. (425)

170	(improv* behavio?r* adj5 (reduc* or decreas* or increas* or lower* or fewer or improv* or enhance* or extend* or develop* or prevalen* or daily or weekly or current or rate or rates or behavio?r* or knowledge or prevent* or attitude* or avoid* or perceive* or percept* or promot* or program* or change*)).ti,ab. (401)

171	(social behavio?r* adj5 (reduc* or decreas* or increas* or lower* or fewer or improv* or enhance* or extend* or develop* or prevalen* or daily or weekly or current or rate or rates or behavio?r* or knowledge or prevent* or attitude* or avoid* or perceive* or percept* or promot* or program* or change*)).ti,ab. (1321)

172	((social-emotion* or socio-emotion*) adj5 (reduc* or decreas* or increas* or lower* or fewer or improv* or enhance* or extend* or develop* or prevalen* or daily or weekly or current or rate or rates or behavio?r* or knowledge or prevent* or attitude* or avoid* or perceive* or percept* or promot* or program* or change*)).ti,ab. (327)

173	(social-charact* adj5 (reduc* or decreas* or increas* or lower* or fewer or improv* or enhance* or extend* or develop* or prevalen* or daily or weekly or current or rate or rates or behavio?r* or knowledge or prevent* or attitude* or avoid* or perceive* or percept* or promot* or program* or change*)).ti,ab. (88)

174	(social-inclusion adj5 (reduc* or decreas* or increas* or lower* or fewer or improv* or enhance* or extend* or develop* or prevalen* or daily or weekly or current or rate or rates or behavio?r* or knowledge or prevent* or attitude* or avoid* or perceive* or percept* or promot* or program* or change*)).ti,ab. (138)

175	(social develop* adj5 (reduc* or decreas* or increas* or lower* or fewer or improv* or enhance* or extend* or develop* or prevalen* or daily or weekly or current or rate or rates or behavio?r* or knowledge or prevent* or attitude* or avoid* or perceive* or percept* or promot* or program* or change*)).ti,ab. (1222)

176	(social competen* adj5 (reduc* or decreas* or increas* or lower* or fewer or improv* or enhance* or extend* or develop* or prevalen* or daily or weekly or current or rate or rates or behavio?r* or knowledge or prevent* or attitude* or avoid* or perceive* or percept* or promot* or program* or change*)).ti,ab. (104)

177	(emotion* develop* adj5 (reduc* or decreas* or increas* or lower* or fewer or improv* or enhance* or extend* or develop* or prevalen* or daily or weekly or current or rate or rates or behavio?r* or knowledge or prevent* or attitude* or avoid* or perceive* or percept* or promot* or program* or change*)).ti,ab. (257)

178	(conduct adj5 (reduc* or decreas* or increas* or lower* or fewer or improv* or enhance* or extend* or develop* or prevalen* or daily or weekly or current or rate or rates or behavio?r* or knowledge or prevent* or attitude* or avoid* or perceive* or percept* or promot* or program* or change*)).ti,ab. (1940)

179	(respect adj5 (reduc* or decreas* or increas* or lower* or fewer or improv* or enhance* or extend* or develop* or prevalen* or daily or weekly or current or rate or rates or behavio?r* or knowledge or prevent* or attitude* or avoid* or perceive* or percept* or promot* or program* or change*)).ti,ab. (6340)

180	((safety or safe) adj5 (reduc* or decreas* or increas* or lower* or fewer or improv* or enhance* or extend* or develop* or prevalen* or daily or weekly or current or rate or rates or behavio?r* or knowledge or prevent* or attitude* or avoid* or perceive* or percept* or promot* or program* or change*)).ti,ab. (35193)

181	or/149-180 [VIOLENCE] (71028)

182	111 or 122 or 145 or 181 (211200)

183	program evaluation/ (659)

184	program effectiveness/ (127)

185	exp clinical trials/ (67664)

186	prevent*.ti,ab. (400284)

187	program*.ti,ab. (219712)

188	intervent*.ti,ab. (212462)

189	restorative approach*.ti,ab. (13)

190	control school?.ti,ab. (650)

191	(trial or trials).ti,ab. (171811)

192	or/183-191 (817391)

193	53 and 99 and 182 and 192 (3988)

## WHO International Clinical Trials Registry Platform

| Database name | International Clinical Trials Registry Platform |
| --- | --- |
| Database platform | <http://apps.who.int/trialsearch/> |

Site not searched. Site was out of order due to increased use due to the COVID-19 pandemic.

## OvidSP PsycINFO

| Database name | PsycINFO |
| --- | --- |
| Database platform | OvidSP |
| Dates of database coverage | 1806 to May week 1 2021 |
| Date searched | 11/05/2021 |
| Searched by | JF |
| Number of results | 19,126 |
| EndNote import order | 6 |
| Number of results once duplicates removed | 1387 |
| Search strategy notes | Search lines ending in a ‘/’ are subject heading searches. Search lines ending in ‘/*ab*’ are subject headings with subheadings added. The subheading is listed in square brackets at the end of the line. Search lines beginning ‘exp’ are exploded subject heading searches. Search lines ending in .ti,ab. search in the title and abstract only. Search lines ending in .jx. search in the journal title only. or/*x-y* combines search sets in the range *x-y* with Boolean operator OR. * is used for truncation of words. # is used for a compulsory wildcard. ? is used for an optional wildcard. adj*n* searches for terms within *n* words or each other. Words in square brackets [ ] are comments and are not included in the search terms. |

1	early adolescence/ (2301)

2	emerging adulthood/ (3848)

3	classmates/ (352)

4	exp elementary school students/ (446480

5	high school students/ (32868)

6	junior high school students/ (11627)

7	middle school students/ (9099)

8	elementary school teachers/ (8850)

9	high school teachers/ (4219)

10	junior high school teachers/ (1630)

11	middle school teachers/ (1122)

12	parents/ (42296)

13	adoptive parents/ (1649)

14	exp fathers/ (11325)

15	homosexual parents/ (939)

16	exp mothers/ (42358)

17	single parents/ (1640)

18	child*.ti,ab. (712788)

19	boy?.ti,ab. (74626)

20	girl?.ti,ab. (74210)

21	(schoolage or (school adj1 age)).ti,ab. (13922)

22	schoolchild*.ti,ab. (3828)

23	youngster?.ti,ab. (3693)

24	minor?.ti,ab. (25888)

25	(pupil* or student*).ti,ab. not ("pupil (eye)"/ or exp students/) (345881)

26	prepubescen*.ti,ab. (394)

27	pubescent*.ti,ab. (355)

28	adolescent*.ti,ab. (221330)

29	juvenil*.ti,ab. (26065)

30	underage*.ti,ab. (1170)

31	(preteen* or pre-teen*).ti,ab. (488)

32	(teen or teens).ti,ab. (10648)

33	teenage*.ti,ab. (13578)

34	(youth or youths).ti,ab. (102725)

35	young person*.ti,ab. (2751)

36	young people*.ti,ab. (31916)

37	(kid or kids).ti,ab. (4404)

38	(transition adj4 adult*).ti,ab. (4397)

39	emerging adult*.ti,ab. (5374)

40	young adult.ti,ab. (13637)

41	parent?.ti,ab. (225963)

42	mother?.ti,ab. (123390)

43	father?.ti,ab. (47220)

44	guardian?.ti,ab. (3753)

45	teacher?.ti,ab. (190753)

46	child*.jx. (129387)

47	adolescen*.jx. (46476)

48	youth*.jx. (15807)

49	school*.jx. (27839)

50	(("5" or "6" or "7" or "8" or "9" or "10" or "11" or "12" or "13" or "14" or "15" or "16" or "17" or "18") adj (year* old or year* of age)).ti,ab. (46654)

51	((five or six or seven or eight or nine or ten or eleven or twelve or thirteen or fourteen or fifteen or sixteen or seventeen or eighteen) adj (year* old or year* of age)).ti,ab. (6574)

52	(age* adj ("5" or "6" or "7" or "8" or "9" or "10" or "11" or "12" or "13" or "14" or "15" or "16" or "17" or "18") adj year*).ti,ab. (8349)

53	(age* adj (five or six or seven or eight or nine or ten or eleven or twelve or thirteen or fourteen or fifteen or sixteen or seventeen or eighteen) adj year*).ti,ab. (201)

54	((primary or secondary or year) adj1 ("1" or "2" or "3" or "4" or "5" or "6" or "7" or "8" or "9" or "10" or "11" or "12" or "13")).ti,ab. (79591)

55	(grade? adj1 (first or second or third or fourth or fifth or sixth or seventh or eighth or ninth or tenth or eleventh or twelfth)).ti,ab. (32382)

56	or/1-55 [POPULATION] (1488543)

57	elementary schools/ (8496)

58	high schools/ (7511)

59	junior high schools/ (630)

60	middle schools/ (4392)

61	or/57-60 (20134)

62	management/ (14172)

63	61 and 62 (25)

64	classroom environment/ (8765)

65	school environment/ (13254)

66	classroom management/ (1616)

67	educational administration/ (5336)

68	parent school relationship/ (3000)

69	(school? adj5 (based or level or wide or led or ethos or environment or organi?ation* or climate or toolkit? or approach* or action group? or practice? or whole or health* or leader* or cultur* or manag* or governance or system? or communit*)).ti,ab. (99351)

70	schoolwide.ti,ab. (714)

71	restorative justice/ (698)

72	school?.ti,ab. (389041)

73	61 or 72 (389632)

74	peer relations/ (16743)

75	"peer-to-peer".ti,ab. (1612)

76	(peer adj2 (led or leader* or participat* or mediat* or helper? or resistan* or wide or action? or influen*)).ti,ab. (6473)

77	(relationship* adj2 (led or leader* or participat* or mediat* or helper? or resistan* or wide or action? or influen*)).ti,ab. (30128)

78	school integration/ (1267)

79	community involvement/ (5303)

80	((community or communities) adj2 (led or leader* or participat* or mediat* or helper? or resistan* or wide or action? or influen* or relationship*)).ti,ab. (14515)

81	(pupil? adj2 (led or leader* or participat* or mediat* or helper? or resistan* or wide or action? or influen* or relationship*)).ti,ab. not "pupil (eye)"/ (882)

82	((child or children) adj2 (led or leader* or participat* or mediat* or helper? or resistan* or wide or action? or influen*)).ti,ab. (12313)

83	((young people or young person) adj2 (led or leader* or participat* or mediat* or helper? or resistan* or wide or action? or influen*)).ti,ab. (506)

84	(teen* adj2 (led or leader* or participat* or mediat* or helper? or resistan* or wide or action? or influen*)).ti,ab. (463)

85	(adolescent* adj2 (led or leader* or participat* or mediat* or helper? or resistan* or wide or action? or influen*)).ti,ab. (5927)

86	(youth? adj2 (led or leader* or participat* or mediat* or helper? or resistan* or wide or action? influen*)).ti,ab. (2719)

87	(student? adj2 (led or leader* or participat* or mediat* or helper? or resistan* or wide or action? or influen* or relationship*)).ti,ab. (31762)

88	(teach* adj2 (led or leader* or participat* or mediat* or helper? or resistan* or wide or action? or influen* or relationship*)).ti,ab. (19460)

89	(curricul* adj2 (led or leader* or participat* or mediat* or helper? or resistan* or wide or action? or influen* or relationship*)).ti,ab. (1290)

90	(administrat* adj2 (led or leader* or participat* or mediat* or helper? or resistan* or wide or action? or influen* or relationship*)).ti,ab. (2619)

91	((personnel or staff) adj2 (led or leader* or participat* or mediat* or helper? or resistan* or wide or action? or influen* or relationship*)).ti,ab. (3557)

92	(volunteer* adj2 (led or leader* or participat* or mediat* or helper? or resistan* or wide or action? or influen* or relationship*)).ti,ab. (3029)

93	((advocacy or advocate) adj2 (led or leader* or participat* or mediat* or helper? or resistan* or wide or action? or relationship*)).ti,ab. (759)

94	(parent* adj2 (led or leader* or participat* or mediat* or helper? or resistan* or wide or action? or influen*)).ti,ab. (14229)

95	social environments/ (7693)

96	organizational climate/ (12282)

97	social support/ (38105)

98	social learning/ (5217)

99	(environment* adj2 (led or leader* or participat* or mediat* or helper? or resistan* or wide or action? or influen* or relationship*)).ti,ab. (15885)

100	(socio-ecolog* adj2 (led or leader* or participat* or mediat* or helper? or resistan* or wide or action? or influen* or relationship*)).ti,ab. (30)

101	(learn* adj2 (led or leader* or participat* or mediat* or helper? or resistan* or wide or action? or influen* or relationship*)).ti,ab. (14053)

102	education policy/ (1735)

103	((policy or policies) adj2 (led or leader* or participat* or mediat* or helper? or resistan* or wide or action? or influen* or relationship*)).ti,ab. (5106)

104	or/74-103 (245853)

105	73 and 104 (54765)

106	or/63-71,105 [SCHOOL-WIDE INTERVENTIONS] (153287)

107	prevention/ (32157)

108	smoking cessation/ (13738)

109	"tobacco use disorder"/ (245)

110	tobacco smoking/ (32898)

111	marijuana usage/ (3079)

112	electronic cigarettes/ (1767)

113	or/109-112 (36363)

114	107 and 113 (1093)

115	((smoking or smoked or smoke or smoker) adj5 (reduc* or decreas* or increas* or lower* or fewer or improv* or enhance* or extend* or develop* or prevalen* or daily or weekly or current or rate or rates or behavio?r* or knowledge or prevent* or attitude* or avoid* or perceive* or percept* or promot* or program* or change*)).ti,ab. (27885)

116	(tobacco adj5 (reduc* or decreas* or increas* or lower* or fewer or improv* or enhance* or extend* or develop* or prevalen* or daily or weekly or current or rate or rates or behavio?r* or knowledge or prevent* or attitude* or avoid* or perceive* or percept* or promot* or program* or change*)).ti,ab. (9654)

117	(cigarette? adj5 (reduc* or decreas* or increas* or lower* or fewer or improv* or enhance* or extend* or develop* or prevalen* or daily or weekly or current or rate or rates or behavio?r* or knowledge or prevent* or attitude* or avoid* or perceive* or percept* or promot* or program* or change*)).ti,ab. (8874)

118	(marijuana adj5 (reduc* or decreas* or increas* or lower* or fewer or improv* or enhance* or extend* or develop* or prevalen* or daily or weekly or current or rate or rates or behavio?r* or knowledge or prevent* or attitude* or avoid* or perceive* or percept* or promot* or program* or change*)).ti,ab. (3193)

119	(cannabis adj5 (reduc* or decreas* or increas* or lower* or fewer or improv* or enhance* or extend* or develop* or prevalen* or daily or weekly or current or rate or rates or behavio?r* or knowledge or prevent* or attitude* or avoid* or perceive* or percept* or promot* or program* or change*)).ti,ab. (4196)

120	(solvent? adj5 (reduc* or decreas* or increas* or lower* or fewer or improv* or enhance* or extend* or develop* or prevalen* or daily or weekly or current or rate or rates or behavio?r* or knowledge or prevent* or attitude* or avoid* or perceive* or percept* or promot* or program* or change*)).ti,ab. (187)

121	or/108,114-120 [SMOKING] (45095)

122	exp "substance use disorder"/ (133904)

123	underage drinking/ (868)

124	illegal drug distribution/ (839)

125	or/122-124 (135034)

126	107 and 125 (2209)

127	(alcohol adj5 (reduc* or decreas* or increas* or lower* or fewer or improv* or enhance* or extend* or develop* or prevalen* or daily or weekly or current or rate or rates or behavio?r* or knowledge or prevent* or attitude* or avoid* or perceive* or percept* or promot* or program* or change*)).ti,ab. (42157)

128	(binge drink* adj5 (reduc* or decreas* or increas* or lower* or fewer or improv* or enhance* or extend* or develop* or prevalen* or daily or weekly or current or rate or rates or behavio?r* or knowledge or prevent* or attitude* or avoid* or perceive* or percept* or promot* or program* or change*)).ti,ab. (1856)

129	(underage drink* adj5 (reduc* or decreas* or increas* or lower* or fewer or improv* or enhance* or extend* or develop* or prevalen* or daily or weekly or current or rate or rates or behavio?r* or knowledge or prevent* or attitude* or avoid* or perceive* or percept* or promot* or program* or change*)).ti,ab. (229)

130	(substance? adj5 (reduc* or decreas* or increas* or lower* or fewer or improv* or enhance* or extend* or develop* or prevalen* or daily or weekly or current or rate or rates or behavio?r* or knowledge or prevent* or attitude* or avoid* or perceive* or percept* or promot* or program* or change*)).ti,ab. (29245)

131	(drug? adj5 (reduc* or decreas* or increas* or lower* or fewer or improv* or enhance* or extend* or develop* or prevalen* or daily or weekly or current or rate or rates or behavio?r* or knowledge or prevent* or attitude* or avoid* or perceive* or percept* or promot* or program* or change*)).ti,ab. (59663)

132	or/126-131 [SUBSTANCE ABUSE] (120466)

133	exp academic achievement/ (79107)

134	academic achievement motivation/ (5866)

135	(education* attain* adj5 (reduc* or decreas* or increas* or lower* or fewer or improv* or enhance* or extend* or develop* or prevalen* or daily or weekly or current or rate or rates or behavio?r* or knowledge or prevent* or attitude* or avoid* or perceive* or percept* or promot* or program* or change*)).ti,ab. (1975)

136	(academic attain* adj5 (reduc* or decreas* or increas* or lower* or fewer or improv* or enhance* or extend* or develop* or prevalen* or daily or weekly or current or rate or rates or behavio?r* or knowledge or prevent* or attitude* or avoid* or perceive* or percept* or promot* or program* or change*)).ti,ab. (129)

137	(student* attain* adj5 (reduc* or decreas* or increas* or lower* or fewer or improv* or enhance* or extend* or develop* or prevalen* or daily or weekly or current or rate or rates or behavio?r* or knowledge or prevent* or attitude* or avoid* or perceive* or percept* or promot* or program* or change*)).ti,ab. (63)

138	(education* achieve* adj5 (reduc* or decreas* or increas* or lower* or fewer or improv* or enhance* or extend* or develop* or prevalen* or daily or weekly or current or rate or rates or behavio?r* or knowledge or prevent* or attitude* or avoid* or perceive* or percept* or promot* or program* or change*)).ti,ab. (586)

139	(academic achieve* adj5 (reduc* or decreas* or increas* or lower* or fewer or improv* or enhance* or extend* or develop* or prevalen* or daily or weekly or current or rate or rates or behavio?r* or knowledge or prevent* or attitude* or avoid* or perceive* or percept* or promot* or program* or change*)).ti,ab. (6227)

140	(student* achieve* adj5 (reduc* or decreas* or increas* or lower* or fewer or improv* or enhance* or extend* or develop* or prevalen* or daily or weekly or current or rate or rates or behavio?r* or knowledge or prevent* or attitude* or avoid* or perceive* or percept* or promot* or program* or change*)).ti,ab. (4899)

141	(education* perform* adj5 (reduc* or decreas* or increas* or lower* or fewer or improv* or enhance* or extend* or develop* or prevalen* or daily or weekly or current or rate or rates or behavio?r* or knowledge or prevent* or attitude* or avoid* or perceive* or percept* or promot* or program* or change*)).ti,ab. (166)

142	(academic perform* adj5 (reduc* or decreas* or increas* or lower* or fewer or improv* or enhance* or extend* or develop* or prevalen* or daily or weekly or current or rate or rates or behavio?r* or knowledge or prevent* or attitude* or avoid* or perceive* or percept* or promot* or program* or change*)).ti,ab. (4490)

143	(student* perform* adj5 (reduc* or decreas* or increas* or lower* or fewer or improv* or enhance* or extend* or develop* or prevalen* or daily or weekly or current or rate or rates or behavio?r* or knowledge or prevent* or attitude* or avoid* or perceive* or percept* or promot* or program* or change*)).ti,ab. (2153)

144	(education* outcome? adj5 (reduc* or decreas* or increas* or lower* or fewer or improv* or enhance* or extend* or develop* or prevalen* or daily or weekly or current or rate or rates or behavio?r* or knowledge or prevent* or attitude* or avoid* or perceive* or percept* or promot* or program* or change*)).ti,ab. (950)

145	(academic outcome? adj5 (reduc* or decreas* or increas* or lower* or fewer or improv* or enhance* or extend* or develop* or prevalen* or daily or weekly or current or rate or rates or behavio?r* or knowledge or prevent* or attitude* or avoid* or perceive* or percept* or promot* or program* or change*)).ti,ab. (894)

146	(student* outcome? adj5 (reduc* or decreas* or increas* or lower* or fewer or improv* or enhance* or extend* or develop* or prevalen* or daily or weekly or current or rate or rates or behavio?r* or knowledge or prevent* or attitude* or avoid* or perceive* or percept* or promot* or program* or change*)).ti,ab. (1291)

147	(education* commitment adj5 (reduc* or decreas* or increas* or lower* or fewer or improv* or enhance* or extend* or develop* or prevalen* or daily or weekly or current or rate or rates or behavio?r* or knowledge or prevent* or attitude* or avoid* or perceive* or percept* or promot* or program* or change*)).ti,ab. (12)

148	(academic commitment adj5 (reduc* or decreas* or increas* or lower* or fewer or improv* or enhance* or extend* or develop* or prevalen* or daily or weekly or current or rate or rates or behavio?r* or knowledge or prevent* or attitude* or avoid* or perceive* or percept* or promot* or program* or change*)).ti,ab. (14)

149	(student* commitment adj5 (reduc* or decreas* or increas* or lower* or fewer or improv* or enhance* or extend* or develop* or prevalen* or daily or weekly or current or rate or rates or behavio?r* or knowledge or prevent* or attitude* or avoid* or perceive* or percept* or promot* or program* or change*)).ti,ab. (37)

150	(education* engage* adj5 (reduc* or decreas* or increas* or lower* or fewer or improv* or enhance* or extend* or develop* or prevalen* or daily or weekly or current or rate or rates or behavio?r* or knowledge or prevent* or attitude* or avoid* or perceive* or percept* or promot* or program* or change*)).ti,ab. (35)

151	(academic engage* adj5 (reduc* or decreas* or increas* or lower* or fewer or improv* or enhance* or extend* or develop* or prevalen* or daily or weekly or current or rate or rates or behavio?r* or knowledge or prevent* or attitude* or avoid* or perceive* or percept* or promot* or program* or change*)).ti,ab. (514)

152	(student* engage* adj5 (reduc* or decreas* or increas* or lower* or fewer or improv* or enhance* or extend* or develop* or prevalen* or daily or weekly or current or rate or rates or behavio?r* or knowledge or prevent* or attitude* or avoid* or perceive* or percept* or promot* or program* or change*)).ti,ab. (2046)

153	(dropout adj5 (reduc* or decreas* or increas* or lower* or fewer or improv* or enhance* or extend* or develop* or prevalen* or daily or weekly or current or rate or rates or behavio?r* or knowledge or prevent* or attitude* or avoid* or perceive* or percept* or promot* or program* or change*)).ti,ab. (4418)

154	(absentee* adj5 (reduc* or decreas* or increas* or lower* or fewer or improv* or enhance* or extend* or develop* or prevalen* or daily or weekly or current or rate or rates or behavio?r* or knowledge or prevent* or attitude* or avoid* or perceive* or percept* or promot* or program* or change*)).ti,ab. (1745)

155	or/133-154 [ACADEMIC ACHIEVEMENT] (100824)

156	violence/ or gun violence/ or school violence/ (35007)

157	violent crime/ or physical abuse/ (8169)

158	aggressive behavior/ or threat/ (37268)

159	dating violence/ (1215)

160	exp bullying/ (10320)

161	exp juvenile delinquency/ (19689)

162	behavior problems/ (30015)

163	antisocial behavior/ (10192)

164	emotional abuse/ (2744)

165	verbal abuse/ (548)

166	conflict/ or arguments/ (25251)

167	or/156-166 (160760)

168	107 and 167 (4270)

169	((violent or violence) adj5 (reduc* or decreas* or increas* or lower* or fewer or improv* or enhance* or extend* or develop* or prevalen* or daily or weekly or current or rate or rates or behavio?r* or knowledge or prevent* or attitude* or avoid* or perceive* or percept* or promot* or program* or change*)).ti,ab. (31037)

170	((aggression or aggressive) adj5 (reduc* or decreas* or increas* or lower* or fewer or improv* or enhance* or extend* or develop* or prevalen* or daily or weekly or current or rate or rates or behavio?r* or knowledge or prevent* or attitude* or avoid* or perceive* or percept* or promot* or program* or change*)).ti,ab. (35725)

171	((hostile or hostility) adj5 (reduc* or decreas* or increas* or lower* or fewer or improv* or enhance* or extend* or develop* or prevalen* or daily or weekly or current or rate or rates or behavio?r* or knowledge or prevent* or attitude* or avoid* or perceive* or percept* or promot* or program* or change*)).ti,ab. (5258)

172	(assault* adj5 (reduc* or decreas* or increas* or lower* or fewer or improv* or enhance* or extend* or develop* or prevalen* or daily or weekly or current or rate or rates or behavio?r* or knowledge or prevent* or attitude* or avoid* or perceive* or percept* or promot* or program* or change*)).ti,ab. (3655)

173	(crime adj5 (reduc* or decreas* or increas* or lower* or fewer or improv* or enhance* or extend* or develop* or prevalen* or daily or weekly or current or rate or rates or behavio?r* or knowledge or prevent* or attitude* or avoid* or perceive* or percept* or promot* or program* or change*)).ti,ab. (9125)

174	((abuse? or abusive) adj5 (reduc* or decreas* or increas* or lower* or fewer or improv* or enhance* or extend* or develop* or prevalen* or daily or weekly or current or rate or rates or behavio?r* or knowledge or prevent* or attitude* or avoid* or perceive* or percept* or promot* or program* or change*)).ti,ab. (33583)

175	(misbehav* adj5 (reduc* or decreas* or increas* or lower* or fewer or improv* or enhance* or extend* or develop* or prevalen* or daily or weekly or current or rate or rates or behavio?r* or knowledge or prevent* or attitude* or avoid* or perceive* or percept* or promot* or program* or change*)).ti,ab. (535)

176	(threat? adj5 (reduc* or decreas* or increas* or lower* or fewer or improv* or enhance* or extend* or develop* or prevalen* or daily or weekly or current or rate or rates or behavio?r* or knowledge or prevent* or attitude* or avoid* or perceive* or percept* or promot* or program* or change*)).ti,ab. (12368)

177	(conduct adj5 (reduc* or decreas* or increas* or lower* or fewer or improv* or enhance* or extend* or develop* or prevalen* or daily or weekly or current or rate or rates or behavio?r* or knowledge or prevent* or attitude* or avoid* or perceive* or percept* or promot* or program* or change*)).ti,ab. (8296)

178	(delinquen* adj5 (reduc* or decreas* or increas* or lower* or fewer or improv* or enhance* or extend* or develop* or prevalen* or daily or weekly or current or rate or rates or behavio?r* or knowledge or prevent* or attitude* or avoid* or perceive* or percept* or promot* or program* or change*)).ti,ab. (9044)

179	(disrupt* behavio?r* adj5 (reduc* or decreas* or increas* or lower* or fewer or improv* or enhance* or extend* or develop* or prevalen* or daily or weekly or current or rate or rates or behavio?r* or knowledge or prevent* or attitude* or avoid* or perceive* or percept* or promot* or program* or change*)).ti,ab. (6502)

180	(problem* behavio?r* adj5 (reduc* or decreas* or increas* or lower* or fewer or improv* or enhance* or extend* or develop* or prevalen* or daily or weekly or current or rate or rates or behavio?r* or knowledge or prevent* or attitude* or avoid* or perceive* or percept* or promot* or program* or change*)).ti,ab. (13113)

181	(volatile behavio?r* adj5 (reduc* or decreas* or increas* or lower* or fewer or improv* or enhance* or extend* or develop* or prevalen* or daily or weekly or current or rate or rates or behavio?r* or knowledge or prevent* or attitude* or avoid* or perceive* or percept* or promot* or program* or change*)).ti,ab. (16)

182	((antisocial or anti-social) adj5 (reduc* or decreas* or increas* or lower* or fewer or improv* or enhance* or extend* or develop* or prevalen* or daily or weekly or current or rate or rates or behavio?r* or knowledge or prevent* or attitude* or avoid* or perceive* or percept* or promot* or program* or change*)).ti,ab. (11205)

183	(perpetrat* adj5 (reduc* or decreas* or increas* or lower* or fewer or improv* or enhance* or extend* or develop* or prevalen* or daily or weekly or current or rate or rates or behavio?r* or knowledge or prevent* or attitude* or avoid* or perceive* or percept* or promot* or program* or change*)).ti,ab. (3496)

184	(bully* adj5 (reduc* or decreas* or increas* or lower* or fewer or improv* or enhance* or extend* or develop* or prevalen* or daily or weekly or current or rate or rates or behavio?r* or knowledge or prevent* or attitude* or avoid* or perceive* or percept* or promot* or program* or change*)).ti,ab. (6146)

185	(victim* adj5 (reduc* or decreas* or increas* or lower* or fewer or improv* or enhance* or extend* or develop* or prevalen* or daily or weekly or current or rate or rates or behavio?r* or knowledge or prevent* or attitude* or avoid* or perceive* or percept* or promot* or program* or change*)).ti,ab. (14596)

186	(cyberbully* adj5 (reduc* or decreas* or increas* or lower* or fewer or improv* or enhance* or extend* or develop* or prevalen* or daily or weekly or current or rate or rates or behavio?r* or knowledge or prevent* or attitude* or avoid* or perceive* or percept* or promot* or program* or change*)).ti,ab. (1030)

187	(conflict* adj5 (reduc* or decreas* or increas* or lower* or fewer or improv* or enhance* or extend* or develop* or prevalen* or daily or weekly or current or rate or rates or behavio?r* or knowledge or prevent* or attitude* or avoid* or perceive* or percept* or promot* or program* or change*)).ti,ab. (26420)

188	(positive behavio?r* adj5 (reduc* or decreas* or increas* or lower* or fewer or improv* or enhance* or extend* or develop* or prevalen* or daily or weekly or current or rate or rates or behavio?r* or knowledge or prevent* or attitude* or avoid* or perceive* or percept* or promot* or program* or change*)).ti,ab. (4187)

189	(improv* behavio?r* adj5 (reduc* or decreas* or increas* or lower* or fewer or improv* or enhance* or extend* or develop* or prevalen* or daily or weekly or current or rate or rates or behavio?r* or knowledge or prevent* or attitude* or avoid* or perceive* or percept* or promot* or program* or change*)).ti,ab. (1507)

190	(social behavio?r* adj5 (reduc* or decreas* or increas* or lower* or fewer or improv* or enhance* or extend* or develop* or prevalen* or daily or weekly or current or rate or rates or behavio?r* or knowledge or prevent* or attitude* or avoid* or perceive* or percept* or promot* or program* or change*)).ti,ab. (21622)

191	((social-emotion* or socio-emotion*) adj5 (reduc* or decreas* or increas* or lower* or fewer or improv* or enhance* or extend* or develop* or prevalen* or daily or weekly or current or rate or rates or behavio?r* or knowledge or prevent* or attitude* or avoid* or perceive* or percept* or promot* or program* or change*)).ti,ab. (5583)

192	(social-charact* adj5 (reduc* or decreas* or increas* or lower* or fewer or improv* or enhance* or extend* or develop* or prevalen* or daily or weekly or current or rate or rates or behavio?r* or knowledge or prevent* or attitude* or avoid* or perceive* or percept* or promot* or program* or change*)).ti,ab. (317)

193	(social-inclusion adj5 (reduc* or decreas* or increas* or lower* or fewer or improv* or enhance* or extend* or develop* or prevalen* or daily or weekly or current or rate or rates or behavio?r* or knowledge or prevent* or attitude* or avoid* or perceive* or percept* or promot* or program* or change*)).ti,ab. (723)

194	(social develop* adj5 (reduc* or decreas* or increas* or lower* or fewer or improv* or enhance* or extend* or develop* or prevalen* or daily or weekly or current or rate or rates or behavio?r* or knowledge or prevent* or attitude* or avoid* or perceive* or percept* or promot* or program* or change*)).ti,ab. (6952)

195	(social competen* adj5 (reduc* or decreas* or increas* or lower* or fewer or improv* or enhance* or extend* or develop* or prevalen* or daily or weekly or current or rate or rates or behavio?r* or knowledge or prevent* or attitude* or avoid* or perceive* or percept* or promot* or program* or change*)).ti,ab. (3528)

196	(emotion* develop* adj5 (reduc* or decreas* or increas* or lower* or fewer or improv* or enhance* or extend* or develop* or prevalen* or daily or weekly or current or rate or rates or behavio?r* or knowledge or prevent* or attitude* or avoid* or perceive* or percept* or promot* or program* or change*)).ti,ab. (5351)

197	(conduct adj5 (reduc* or decreas* or increas* or lower* or fewer or improv* or enhance* or extend* or develop* or prevalen* or daily or weekly or current or rate or rates or behavio?r* or knowledge or prevent* or attitude* or avoid* or perceive* or percept* or promot* or program* or change*)).ti,ab. (8296)

198	(respect adj5 (reduc* or decreas* or increas* or lower* or fewer or improv* or enhance* or extend* or develop* or prevalen* or daily or weekly or current or rate or rates or behavio?r* or knowledge or prevent* or attitude* or avoid* or perceive* or percept* or promot* or program* or change*)).ti,ab. (13490)

199	((safety or safe) adj5 (reduc* or decreas* or increas* or lower* or fewer or improv* or enhance* or extend* or develop* or prevalen* or daily or weekly or current or rate or rates or behavio?r* or knowledge or prevent* or attitude* or avoid* or perceive* or percept* or promot* or program* or change*)).ti,ab. (23748)

200	or/168-199 [VIOLENCE] (267280)

201	121 or 132 or 155 or 200 (491361)

202	exp program evaluation/ (20985)

203	exp clinical trials/ (12819)

204	prevent*.ti,ab. (231370)

205	program*.ti,ab. (407944)

206	intervent*.ti,ab. (404511)

207	restorative approach*.ti,ab. (131)

208	control school?.ti,ab. (647)

209	(trial or trials).ti,ab. (189500)

210	or/202-209 (992095)

211	56 and 106 and 201 and 210 (19126)

## SCOPUS

| Database name | SCOPUS |
| --- | --- |
| Database platform | [www.scopus.com](http://www.scopus.com) |
| Dates of database coverage | Complete database to search date |
| Date searched | 13 May 2021 |
| Searched by | JF |
| Number of results | 19,365 |
| EndNote import order | 14 |
| Number of results once duplicates removed | 1096 |
| Search strategy notes | All terms are searched in the title, abstract and author keywords. * is used for truncation of words. W/*n* searches for terms within *n* words or each other. |

(TITLE-ABS-KEY (child or boy or girl or schoolage or (school w/1 age) or schoolchild or youngster or minor or pupil or (student not "medical student") or prepubescent or pubescent or adolescent or juvenile or underage or preteen or "pre-teen" or teen or teenage or youth or "young person" or "young people" or kid or (transition w/4 adult) or "emerging adult" or "young adult" or parent or mother or father or guardian or teacher)) AND (TITLE-ABS-KEY ((school* W/5 (based OR level OR wide OR led OR ethos OR environment OR organization* OR organisation* OR climate OR toolkit* OR approach* OR "action group*" OR practice* OR whole OR health* OR leader* OR cultur* OR manag* OR governance OR system* OR communit*)) OR schoolwide OR "restor* justice" OR ((((peer or relationship* or child or children or "young people" or "young person" or teen or teens or teenage* or adolescen* or youth* or parent*) W/2 (led OR leader* OR participat* OR mediat* OR helper* OR resistan* OR wide OR action* or influen*)) OR ((community or communities or pupil or student or teach* or curricul* or administrat* or personnel or staff or volunteer* or advocacy or advocate or environment* or "socio-ecolog*" or learn* or policy or policies) W/2 (led OR leader* OR participat* OR mediat* OR helper* OR resistan* OR wide OR action* or influen* or relationship*)) OR "peer-to-peer") AND school*))) AND (TITLE-ABS-KEY (prevent* OR program* OR intervent* OR "restorative approach*" OR "control school*" OR trial OR trials)) AND (TITLE-ABS-KEY ((violent OR violence OR aggression OR aggressive OR hostile OR hostility OR assault* OR crime OR abuse* OR abusive OR misbehav* OR threat* OR conduct OR delinquen* OR disrupt* OR "problem* behavior*" OR "problem behaviour*" OR "volatile behavior*" OR "volatile behaviour*" OR antisocial OR "anti-social" OR perpetrat* OR bully* OR victim* OR cyberbully* OR conflict* OR "positive behavior*" OR "positive behaviour*" OR "improv* behavior*" OR "improv* behaviour*" OR "social behavior*" OR "social behaviour*" OR "social-emotion*" OR "socio-emotion*" OR "social-charact*" OR "social-inclusion" OR "social develop*" OR "social competen*" OR "emotion* develop*" OR conduct OR respect OR safety OR safe OR "education* attain*" OR "academic attain*" OR "student* attain*" OR "education* achieve*" OR "academic achieve*" OR "student achieve*" OR "education* perform*" OR "academic perform*" OR "education* perform*" OR "education* outcome*" OR "academic outcome*" OR "student* outcome*" OR "education* commitment" OR "academic commitment" OR "student* commitment" OR "education* engage*" OR "academic engage*" OR "student* engage*" OR dropout OR absentee* OR alcohol OR "binge drink*" OR "underage drink*" OR substance* OR drug* OR smoking OR smoked OR smoke OR smoker OR tobacco OR cigarette* OR marijuana OR cannabis OR solvent*) W/5 (reduc* OR decreas* OR increas* OR lower* OR fewer OR improv* OR enhance* OR extend* OR develop* OR prevalen* OR daily OR weekly OR current OR rate OR rates OR behavior* OR behaviour* OR knowledge OR prevent* OR attitude* OR avoid* OR perceive* OR percept* OR promot* OR program* OR change*)))

## OvidSP Social Policy and Practice

| Database name | Social Policy & Practice |
| --- | --- |
| Database platform | OvidSP |
| Dates of database coverage | 202101 |
| Date searched | 11/05/2021 |
| Searched by | JF |
| Number of results | 743 |
| EndNote import order | 7 |
| Number of results once duplicates removed | 64 |
| Search strategy notes | Search lines ending in .ti,ab. search in the title and abstract only. Search lines ending in .jx. search in the journal title only. or/*x-y* combines search sets in the range *x-y* with Boolean operator OR. * is used for truncation of words. # is used for a compulsory wildcard. ? is used for an optional wildcard. adj*n* searches for terms within *n* words or each other. Words in square brackets [ ] are comments and are not included in the search terms. |

1	child*.ti,ab. (81550)

2	boy?.ti,ab. (2384)

3	girl?.ti,ab. (2801)

4	(schoolage or (school adj1 age)).ti,ab. (446)

5	schoolchild*.ti,ab. (154)

6	youngster?.ti,ab. (205)

7	minor?.ti,ab. (1009)

8	((pupil* or student*) not ((pupil adj5 eye?) or (student* adj medic*))).ti,ab. (14057)

9	prepubescen*.ti,ab. (17)

10	pubescent*.ti,ab. (19)

11	adolescent*.ti,ab. (7815)

12	juvenil*.ti,ab. (1512)

13	underage*.ti,ab. (88)

14	(preteen* or pre-teen*).ti,ab. (28)

15	(teen or teens).ti,ab. (485)

16	teenage*.ti,ab. (2260)

17	(youth or youths).ti,ab. (11249)

18	young person*.ti,ab. (1544)

19	young people*.ti,ab. (25548)

20	(kid or kids).ti,ab. (489)

21	(transition adj4 adult*).ti,ab. (810)

22	emerging adult*.ti,ab. (113)

23	young adult.ti,ab. (368

24	parent?.ti,ab. (22907)

25	mother?.ti,ab. (6789)

26	father?.ti,ab. (2640)

27	guardian?.ti,ab. (779)

28	teacher?.ti,ab. (4913)

29	child*.jx. (23079)

30	adolescen*.jx. (1323)

31	youth*.jx. (4888)

32	school*.jx. (334)

33	(("5" or "6" or "7" or "8" or "9" or "10" or "11" or "12" or "13" or "14" or "15" or "16" or "17" or "18") adj (year* old or year* of age)).ti,ab. (1886)

34	((five or six or seven or eight or nine or ten or eleven or twelve or thirteen or fourteen or fifteen or sixteen or seventeen or eighteen) adj (year* old or year* of age)).ti,ab. (305)

35	(age* adj ("5" or "6" or "7" or "8" or "9" or "10" or "11" or "12" or "13" or "14" or "15" or "16" or "17" or "18") adj year*).ti,ab. (272)

36	(age* adj (five or six or seven or eight or nine or ten or eleven or twelve or thirteen or fourteen or fifteen or sixteen or seventeen or eighteen) adj year*).ti,ab. (21)

37	((primary or secondary or year) adj1 ("1" or "2" or "3" or "4" or "5" or "6" or "7" or "8" or "9" or "10" or "11" or "12" or "13")).ti,ab. (3612)

38	(grade? adj1 (first or second or third or fourth or fifth or sixth or seventh or eighth or ninth or tenth or eleventh or twelfth)).ti,ab. (104)

39	or/1-38 (120097)

40	(school? adj5 (based or level or wide or led or ethos or environment or organi?ation* or climate or toolkit? or approach* or action group? or practice? or whole or health* or leader* or cultur* or manag* or governance or system? or communit*)).ti,ab. (6313)

41	schoolwide.ti,ab. (4)

42	restor* justice.ti,ab. (342)

43	school?.ti,ab,jx. (22403)

44	"peer-to-peer".ti,ab. (137)

45	(peer adj2 (led or leader* or participat* or mediat* or helper? or resistan* or wide or action? or influen*)).ti,ab. (266)

46	(relationship* adj2 (led or leader* or participat* or mediat* or helper? or resistan* or wide or action? or influen*)).ti,ab. (735)

47	((community or communities) adj2 (led or leader* or participat* or mediat* or helper? or resistan* or wide or action? or influen* or relationship*)).ti,ab. (2384)

48	(pupil? adj2 (led or leader* or participat* or mediat* or helper? or resistan* or wide or action? or influen* or relationship*)).ti,ab. (136)

49	((child or children) adj2 (led or leader* or participat* or mediat* or helper? or resistan* or wide or action? or influen*)).ti,ab. (1339)

50	((young people or young person) adj2 (led or leader* or participat* or mediat* or helper? or resistan* or wide or action? or influen*)).ti,ab. (438)

51	(teen* adj2 (led or leader* or participat* or mediat* or helper? or resistan* or wide or action? or influen*)).ti,ab. (28)

52	(adolescent* adj2 (led or leader* or participat* or mediat* or helper? or resistan* or wide or action? or influen*)).ti,ab. (145)

53	(youth? adj2 (led or leader* or participat* or mediat* or helper? or resistan* or wide or action? or influen*)).ti,ab. (394)

54	(student? adj2 (led or leader* or participat* or mediat* or helper? or resistan* or wide or action? or influen*)).ti,ab. (308)

55	(teach* adj2 (led or leader* or participat* or mediat* or helper? or resistan* or wide or action? or influen* or relationship*)).ti,ab. (287)

56	(curricul* adj2 (led or leader* or participat* or mediat* or helper? or resistan* or wide or action? or influen* or relationship*)).ti,ab. (51)

57	(administrat* adj2 (led or leader* or participat* or mediat* or helper? or resistan* or wide or action? or influen* or relationship*)).ti,ab. (48)

58	((personnel or staff) adj2 (led or leader* or participat* or mediat* or helper? or resistan* or wide or action? or influen* or relationship*)).ti,ab. (551)

59	(volunteer* adj2 (led or leader* or participat* or mediat* or helper? or resistan* or wide or action? or influen* or relationship*)).ti,ab. (258)

60	((advocacy or advocate) adj2 (led or leader* or participat* or mediat* or helper? or resistan* or wide or action? or influen* or relationship*)).ti,ab. (156)

61	(parent* adj2 (led or leader* or participat* or mediat* or helper? or resistan* or wide or action? or influen*)).ti,ab. (751)

62	(environment* adj2 (led or leader* or participat* or mediat* or helper? or resistan* or wide or action? or influen* or relationship*)).ti,ab. (491)

63	(socio-ecolog* adj2 (led or leader* or participat* or mediat* or helper? or resistan* or wide or action? or influen* or relationship*)).ti,ab. (0)

64	(learn* adj2 (led or leader* or participat* or mediat* or helper? or resistan* or wide or action? or influen* or relationship*)).ti,ab. (544)

65	((policy or policies) adj2 (led or leader* or participat* or mediat* or helper? or resistan* or wide or action? or influen* or relationship*)).ti,ab. (1239)

66	or/44-65 (9937)

67	43 and 66 (1338)

68	or/40-42,67 (7446)

69	((smoking or smoked or smoke or smoker) adj5 (reduc* or decreas* or increas* or lower* or fewer or improv* or enhance* or extend* or develop* or prevalen* or daily or weekly or current or rate or rates or behavio?r* or knowledge or prevent* or attitude* or avoid* or perceive* or percept* or promot* or program* or change*)).ti,ab. (527)

70	(tobacco adj5 (reduc* or decreas* or increas* or lower* or fewer or improv* or enhance* or extend* or develop* or prevalen* or daily or weekly or current or rate or rates or behavio?r* or knowledge or prevent* or attitude* or avoid* or perceive* or percept* or promot* or program* or change*)).ti,ab. (110)

71	(cigarette? adj5 (reduc* or decreas* or increas* or lower* or fewer or improv* or enhance* or extend* or develop* or prevalen* or daily or weekly or current or rate or rates or behavio?r* or knowledge or prevent* or attitude* or avoid* or perceive* or percept* or promot* or program* or change*)).ti,ab. (60)

72	(marijuana adj5 (reduc* or decreas* or increas* or lower* or fewer or improv* or enhance* or extend* or develop* or prevalen* or daily or weekly or current or rate or rates or behavio?r* or knowledge or prevent* or attitude* or avoid* or perceive* or percept* or promot* or program* or change*)).ti,ab. (29)

73	(cannabis adj5 (reduc* or decreas* or increas* or lower* or fewer or improv* or enhance* or extend* or develop* or prevalen* or daily or weekly or current or rate or rates or behavio?r* or knowledge or prevent* or attitude* or avoid* or perceive* or percept* or promot* or program* or change*)).ti,ab. (65)

74	(solvent? adj5 (reduc* or decreas* or increas* or lower* or fewer or improv* or enhance* or extend* or develop* or prevalen* or daily or weekly or current or rate or rates or behavio?r* or knowledge or prevent* or attitude* or avoid* or perceive* or percept* or promot* or program* or change*)).ti,ab. (12)

75	(alcohol adj5 (reduc* or decreas* or increas* or lower* or fewer or improv* or enhance* or extend* or develop* or prevalen* or daily or weekly or current or rate or rates or behavio?r* or knowledge or prevent* or attitude* or avoid* or perceive* or percept* or promot* or program* or change*)).ti,ab. (1358)

76	(binge drink* adj5 (reduc* or decreas* or increas* or lower* or fewer or improv* or enhance* or extend* or develop* or prevalen* or daily or weekly or current or rate or rates or behavio?r* or knowledge or prevent* or attitude* or avoid* or perceive* or percept* or promot* or program* or change*)).ti,ab. (48)

77	(underage drink* adj5 (reduc* or decreas* or increas* or lower* or fewer or improv* or enhance* or extend* or develop* or prevalen* or daily or weekly or current or rate or rates or behavio?r* or knowledge or prevent* or attitude* or avoid* or perceive* or percept* or promot* or program* or change*)).ti,ab. (13)

78	(substance? adj5 (reduc* or decreas* or increas* or lower* or fewer or improv* or enhance* or extend* or develop* or prevalen* or daily or weekly or current or rate or rates or behavio?r* or knowledge or prevent* or attitude* or avoid* or perceive* or percept* or promot* or program* or change*)).ti,ab. (1384)

79	(drug? adj5 (reduc* or decreas* or increas* or lower* or fewer or improv* or enhance* or extend* or develop* or prevalen* or daily or weekly or current or rate or rates or behavio?r* or knowledge or prevent* or attitude* or avoid* or perceive* or percept* or promot* or program* or change*)).ti,ab. (2101)

80	(education* attain* adj5 (reduc* or decreas* or increas* or lower* or fewer or improv* or enhance* or extend* or develop* or prevalen* or daily or weekly or current or rate or rates or behavio?r* or knowledge or prevent* or attitude* or avoid* or perceive* or percept* or promot* or program* or change*)).ti,ab. (291)

81	(academic attain* adj5 (reduc* or decreas* or increas* or lower* or fewer or improv* or enhance* or extend* or develop* or prevalen* or daily or weekly or current or rate or rates or behavio?r* or knowledge or prevent* or attitude* or avoid* or perceive* or percept* or promot* or program* or change*)).ti,ab. (18)

82	(student* attain* adj5 (reduc* or decreas* or increas* or lower* or fewer or improv* or enhance* or extend* or develop* or prevalen* or daily or weekly or current or rate or rates or behavio?r* or knowledge or prevent* or attitude* or avoid* or perceive* or percept* or promot* or program* or change*)).ti,ab. (5)

83	(education* achieve* adj5 (reduc* or decreas* or increas* or lower* or fewer or improv* or enhance* or extend* or develop* or prevalen* or daily or weekly or current or rate or rates or behavio?r* or knowledge or prevent* or attitude* or avoid* or perceive* or percept* or promot* or program* or change*)).ti,ab. (117)

84	(academic achieve* adj5 (reduc* or decreas* or increas* or lower* or fewer or improv* or enhance* or extend* or develop* or prevalen* or daily or weekly or current or rate or rates or behavio?r* or knowledge or prevent* or attitude* or avoid* or perceive* or percept* or promot* or program* or change*)).ti,ab. (70)

85	(student* achieve* adj5 (reduc* or decreas* or increas* or lower* or fewer or improv* or enhance* or extend* or develop* or prevalen* or daily or weekly or current or rate or rates or behavio?r* or knowledge or prevent* or attitude* or avoid* or perceive* or percept* or promot* or program* or change*)).ti,ab. (16)

86	(education* perform* adj5 (reduc* or decreas* or increas* or lower* or fewer or improv* or enhance* or extend* or develop* or prevalen* or daily or weekly or current or rate or rates or behavio?r* or knowledge or prevent* or attitude* or avoid* or perceive* or percept* or promot* or program* or change*)).ti,ab. (36)

87	(academic perform* adj5 (reduc* or decreas* or increas* or lower* or fewer or improv* or enhance* or extend* or develop* or prevalen* or daily or weekly or current or rate or rates or behavio?r* or knowledge or prevent* or attitude* or avoid* or perceive* or percept* or promot* or program* or change*)).ti,ab. (60)

88	(student* perform* adj5 (reduc* or decreas* or increas* or lower* or fewer or improv* or enhance* or extend* or develop* or prevalen* or daily or weekly or current or rate or rates or behavio?r* or knowledge or prevent* or attitude* or avoid* or perceive* or percept* or promot* or program* or change*)).ti,ab. (8)

89	(education* outcome? adj5 (reduc* or decreas* or increas* or lower* or fewer or improv* or enhance* or extend* or develop* or prevalen* or daily or weekly or current or rate or rates or behavio?r* or knowledge or prevent* or attitude* or avoid* or perceive* or percept* or promot* or program* or change*)).ti,ab. (136)

90	(academic outcome? adj5 (reduc* or decreas* or increas* or lower* or fewer or improv* or enhance* or extend* or develop* or prevalen* or daily or weekly or current or rate or rates or behavio?r* or knowledge or prevent* or attitude* or avoid* or perceive* or percept* or promot* or program* or change*)).ti,ab. (18)

91	(student* outcome? adj5 (reduc* or decreas* or increas* or lower* or fewer or improv* or enhance* or extend* or develop* or prevalen* or daily or weekly or current or rate or rates or behavio?r* or knowledge or prevent* or attitude* or avoid* or perceive* or percept* or promot* or program* or change*)).ti,ab. (13)

92	(education* commitment adj5 (reduc* or decreas* or increas* or lower* or fewer or improv* or enhance* or extend* or develop* or prevalen* or daily or weekly or current or rate or rates or behavio?r* or knowledge or prevent* or attitude* or avoid* or perceive* or percept* or promot* or program* or change*)).ti,ab. (0)

93	(academic commitment adj5 (reduc* or decreas* or increas* or lower* or fewer or improv* or enhance* or extend* or develop* or prevalen* or daily or weekly or current or rate or rates or behavio?r* or knowledge or prevent* or attitude* or avoid* or perceive* or percept* or promot* or program* or change*)).ti,ab. (0)

94	(student* commitment adj5 (reduc* or decreas* or increas* or lower* or fewer or improv* or enhance* or extend* or develop* or prevalen* or daily or weekly or current or rate or rates or behavio?r* or knowledge or prevent* or attitude* or avoid* or perceive* or percept* or promot* or program* or change*)).ti,ab. (1)

95	(education* engage* adj5 (reduc* or decreas* or increas* or lower* or fewer or improv* or enhance* or extend* or develop* or prevalen* or daily or weekly or current or rate or rates or behavio?r* or knowledge or prevent* or attitude* or avoid* or perceive* or percept* or promot* or program* or change*)).ti,ab. (4)

96	(academic engage* adj5 (reduc* or decreas* or increas* or lower* or fewer or improv* or enhance* or extend* or develop* or prevalen* or daily or weekly or current or rate or rates or behavio?r* or knowledge or prevent* or attitude* or avoid* or perceive* or percept* or promot* or program* or change*)).ti,ab. (1)

97	(student* engage* adj5 (reduc* or decreas* or increas* or lower* or fewer or improv* or enhance* or extend* or develop* or prevalen* or daily or weekly or current or rate or rates or behavio?r* or knowledge or prevent* or attitude* or avoid* or perceive* or percept* or promot* or program* or change*)).ti,ab. (12)

98	(dropout adj5 (reduc* or decreas* or increas* or lower* or fewer or improv* or enhance* or extend* or develop* or prevalen* or daily or weekly or current or rate or rates or behavio?r* or knowledge or prevent* or attitude* or avoid* or perceive* or percept* or promot* or program* or change*)).ti,ab. (87)

99	(absentee* adj5 (reduc* or decreas* or increas* or lower* or fewer or improv* or enhance* or extend* or develop* or prevalen* or daily or weekly or current or rate or rates or behavio?r* or knowledge or prevent* or attitude* or avoid* or perceive* or percept* or promot* or program* or change*)).ti,ab. (61)

100	((violent or violence) adj5 (reduc* or decreas* or increas* or lower* or fewer or improv* or enhance* or extend* or develop* or prevalen* or daily or weekly or current or rate or rates or behavio?r* or knowledge or prevent* or attitude* or avoid* or perceive* or percept* or promot* or program* or change*)).ti,ab. (3202)

101	((aggression or aggressive) adj5 (reduc* or decreas* or increas* or lower* or fewer or improv* or enhance* or extend* or develop* or prevalen* or daily or weekly or current or rate or rates or behavio?r* or knowledge or prevent* or attitude* or avoid* or perceive* or percept* or promot* or program* or change*)).ti,ab. (1108)

102	((hostile or hostility) adj5 (reduc* or decreas* or increas* or lower* or fewer or improv* or enhance* or extend* or develop* or prevalen* or daily or weekly or current or rate or rates or behavio?r* or knowledge or prevent* or attitude* or avoid* or perceive* or percept* or promot* or program* or change*)).ti,ab. (153)

103	(assault* adj5 (reduc* or decreas* or increas* or lower* or fewer or improv* or enhance* or extend* or develop* or prevalen* or daily or weekly or current or rate or rates or behavio?r* or knowledge or prevent* or attitude* or avoid* or perceive* or percept* or promot* or program* or change*)).ti,ab. (229)

104	(crime adj5 (reduc* or decreas* or increas* or lower* or fewer or improv* or enhance* or extend* or develop* or prevalen* or daily or weekly or current or rate or rates or behavio?r* or knowledge or prevent* or attitude* or avoid* or perceive* or percept* or promot* or program* or change*)).ti,ab. (2322)

105	((abuse? or abusive) adj5 (reduc* or decreas* or increas* or lower* or fewer or improv* or enhance* or extend* or develop* or prevalen* or daily or weekly or current or rate or rates or behavio?r* or knowledge or prevent* or attitude* or avoid* or perceive* or percept* or promot* or program* or change*)).ti,ab. (5626)

106	(misbehav* adj5 (reduc* or decreas* or increas* or lower* or fewer or improv* or enhance* or extend* or develop* or prevalen* or daily or weekly or current or rate or rates or behavio?r* or knowledge or prevent* or attitude* or avoid* or perceive* or percept* or promot* or program* or change*)).ti,ab. (22)

107	(threat? adj5 (reduc* or decreas* or increas* or lower* or fewer or improv* or enhance* or extend* or develop* or prevalen* or daily or weekly or current or rate or rates or behavio?r* or knowledge or prevent* or attitude* or avoid* or perceive* or percept* or promot* or program* or change*)).ti,ab. (331)

108	(conduct adj5 (reduc* or decreas* or increas* or lower* or fewer or improv* or enhance* or extend* or develop* or prevalen* or daily or weekly or current or rate or rates or behavio?r* or knowledge or prevent* or attitude* or avoid* or perceive* or percept* or promot* or program* or change*)).ti,ab. (407)

109	(delinquen* adj5 (reduc* or decreas* or increas* or lower* or fewer or improv* or enhance* or extend* or develop* or prevalen* or daily or weekly or current or rate or rates or behavio?r* or knowledge or prevent* or attitude* or avoid* or perceive* or percept* or promot* or program* or change*)).ti,ab. (368)

110	(disrupt* behavio?r* adj5 (reduc* or decreas* or increas* or lower* or fewer or improv* or enhance* or extend* or develop* or prevalen* or daily or weekly or current or rate or rates or behavio?r* or knowledge or prevent* or attitude* or avoid* or perceive* or percept* or promot* or program* or change*)).ti,ab. (301)

111	(problem* behavio?r* adj5 (reduc* or decreas* or increas* or lower* or fewer or improv* or enhance* or extend* or develop* or prevalen* or daily or weekly or current or rate or rates or behavio?r* or knowledge or prevent* or attitude* or avoid* or perceive* or percept* or promot* or program* or change*)).ti,ab. (656)

112	(volatile behavio?r* adj5 (reduc* or decreas* or increas* or lower* or fewer or improv* or enhance* or extend* or develop* or prevalen* or daily or weekly or current or rate or rates or behavio?r* or knowledge or prevent* or attitude* or avoid* or perceive* or percept* or promot* or program* or change*)).ti,ab. (1)

113	((antisocial or anti-social) adj5 (reduc* or decreas* or increas* or lower* or fewer or improv* or enhance* or extend* or develop* or prevalen* or daily or weekly or current or rate or rates or behavio?r* or knowledge or prevent* or attitude* or avoid* or perceive* or percept* or promot* or program* or change*)).ti,ab. (2284)

114	(perpetrat* adj5 (reduc* or decreas* or increas* or lower* or fewer or improv* or enhance* or extend* or develop* or prevalen* or daily or weekly or current or rate or rates or behavio?r* or knowledge or prevent* or attitude* or avoid* or perceive* or percept* or promot* or program* or change*)).ti,ab. (503)

115	(bully* adj5 (reduc* or decreas* or increas* or lower* or fewer or improv* or enhance* or extend* or develop* or prevalen* or daily or weekly or current or rate or rates or behavio?r* or knowledge or prevent* or attitude* or avoid* or perceive* or percept* or promot* or program* or change*)).ti,ab. (601)

116	(victim* adj5 (reduc* or decreas* or increas* or lower* or fewer or improv* or enhance* or extend* or develop* or prevalen* or daily or weekly or current or rate or rates or behavio?r* or knowledge or prevent* or attitude* or avoid* or perceive* or percept* or promot* or program* or change*)).ti,ab. (1462)

117	(cyberbully* adj5 (reduc* or decreas* or increas* or lower* or fewer or improv* or enhance* or extend* or develop* or prevalen* or daily or weekly or current or rate or rates or behavio?r* or knowledge or prevent* or attitude* or avoid* or perceive* or percept* or promot* or program* or change*)).ti,ab. (81)

118	(conflict* adj5 (reduc* or decreas* or increas* or lower* or fewer or improv* or enhance* or extend* or develop* or prevalen* or daily or weekly or current or rate or rates or behavio?r* or knowledge or prevent* or attitude* or avoid* or perceive* or percept* or promot* or program* or change*)).ti,ab. (854)

119	(positive behavio?r* adj5 (reduc* or decreas* or increas* or lower* or fewer or improv* or enhance* or extend* or develop* or prevalen* or daily or weekly or current or rate or rates or behavio?r* or knowledge or prevent* or attitude* or avoid* or perceive* or percept* or promot* or program* or change*)).ti,ab. (260)

120	(improv* behavio?r* adj5 (reduc* or decreas* or increas* or lower* or fewer or improv* or enhance* or extend* or develop* or prevalen* or daily or weekly or current or rate or rates or behavio?r* or knowledge or prevent* or attitude* or avoid* or perceive* or percept* or promot* or program* or change*)).ti,ab. (91)

121	(social behavio?r* adj5 (reduc* or decreas* or increas* or lower* or fewer or improv* or enhance* or extend* or develop* or prevalen* or daily or weekly or current or rate or rates or behavio?r* or knowledge or prevent* or attitude* or avoid* or perceive* or percept* or promot* or program* or change*)).ti,ab. (1714)

122	((social-emotion* or socio-emotion*) adj5 (reduc* or decreas* or increas* or lower* or fewer or improv* or enhance* or extend* or develop* or prevalen* or daily or weekly or current or rate or rates or behavio?r* or knowledge or prevent* or attitude* or avoid* or perceive* or percept* or promot* or program* or change*)).ti,ab. (225)

123	(social-charact* adj5 (reduc* or decreas* or increas* or lower* or fewer or improv* or enhance* or extend* or develop* or prevalen* or daily or weekly or current or rate or rates or behavio?r* or knowledge or prevent* or attitude* or avoid* or perceive* or percept* or promot* or program* or change*)).ti,ab. (24)

124	(social-inclusion adj5 (reduc* or decreas* or increas* or lower* or fewer or improv* or enhance* or extend* or develop* or prevalen* or daily or weekly or current or rate or rates or behavio?r* or knowledge or prevent* or attitude* or avoid* or perceive* or percept* or promot* or program* or change*)).ti,ab. (945)

125	(social develop* adj5 (reduc* or decreas* or increas* or lower* or fewer or improv* or enhance* or extend* or develop* or prevalen* or daily or weekly or current or rate or rates or behavio?r* or knowledge or prevent* or attitude* or avoid* or perceive* or percept* or promot* or program* or change*)).ti,ab. (588)

126	(social competen* adj5 (reduc* or decreas* or increas* or lower* or fewer or improv* or enhance* or extend* or develop* or prevalen* or daily or weekly or current or rate or rates or behavio?r* or knowledge or prevent* or attitude* or avoid* or perceive* or percept* or promot* or program* or change*)).ti,ab. (83)

127	(emotion* develop* adj5 (reduc* or decreas* or increas* or lower* or fewer or improv* or enhance* or extend* or develop* or prevalen* or daily or weekly or current or rate or rates or behavio?r* or knowledge or prevent* or attitude* or avoid* or perceive* or percept* or promot* or program* or change*)).ti,ab. (216)

128	(conduct adj5 (reduc* or decreas* or increas* or lower* or fewer or improv* or enhance* or extend* or develop* or prevalen* or daily or weekly or current or rate or rates or behavio?r* or knowledge or prevent* or attitude* or avoid* or perceive* or percept* or promot* or program* or change*)).ti,ab. (407)

129	(respect adj5 (reduc* or decreas* or increas* or lower* or fewer or improv* or enhance* or extend* or develop* or prevalen* or daily or weekly or current or rate or rates or behavio?r* or knowledge or prevent* or attitude* or avoid* or perceive* or percept* or promot* or program* or change*)).ti,ab. (631)

130	((safety or safe) adj5 (reduc* or decreas* or increas* or lower* or fewer or improv* or enhance* or extend* or develop* or prevalen* or daily or weekly or current or rate or rates or behavio?r* or knowledge or prevent* or attitude* or avoid* or perceive* or percept* or promot* or program* or change*)).ti,ab. (2479)

131	or/69-130 (26167)

132	prevent*.ti,ab. (18340)

133	program*.ti,ab. (34558)

134	intervent*.ti,ab. (26871)

135	restorative approach*.ti,ab. (51)

136	control school?.ti,ab. (14)

137	(trial or trials).ti,ab. (4185)

138	or/132-137 (68640)

139	39 and 68 and 131 and 138 (743)

## Clarivate Analytics Web of Science Social Sciences Citation Index

| Database name | Social Sciences Citation Index |
| --- | --- |
| Database platform | Clarivate Analytics Web of Science |
| Dates of database coverage | 1970 – present, last updated 13 May 2021 |
| Date searched | 14/05/2021 |
| Searched by | JF |
| Number of results | 12,681 |
| EndNote import order | 13 |
| Number of results once duplicates removed | 970 |
| Search strategy notes | All terms are searched in the title, abstract and author keywords. * is used for truncation of words. W/*n* searches for terms within *n* words or each other. |

| # 1 | TS=(child* or boy$ or girl$ or schoolage or (school near/1 age) or schoolchild* or youngster$ or minor$ or pupil* or (student* not "medical student*") or prepubescen* or pubescent* or adolescent* or juvenil* or underage* or preteen* or "pre-teen*" or teen or teens or teenage* or youth or youths or "young person*" or "young people*" or kid or kids or (transition near/4 adult*) or "emerging adult*" or "young adult*" or parent$ or mother$ or father$ or guardian$ or teacher$) | 1,293.520 |  |
| --- | --- | --- | --- |
| # 2 | SO=(child* or adolescen* or youth* or school*) | 55,577 |  |
| # 3 | #2 OR #1 | 1,303,510 |  |
| # 4 | TS=(school$ near/5 (based or level or wide or led or ethos or environment or organi?ation* or climate or toolkit$ or approach* or "action group$" or practice$ or whole or health* or leader* or cultur* or manag* or governance or system$ or communit*)) | 84,816 |  |
| # 5 | TS=(schoolwide or "restor* justice") | 1,401 |  |
| # 6 | TS=(school$) | 329,995 |  |
| # 7 | TS=("peer-to-peer") | 2,315 |  |
| # 8 | TS=((peer or relationship* or child or children or "young people" or "young person" or teen or teens or teenage* or adolescen* or youth$ or parent*) near/2 (led or leader* or participat* or mediat* or helper$ or resistan* or wide or action$ or influen*)) | 84,430 |  |
| # 9 | TS=((community or communities or pupil$ or student$ or teach* or curricul* or administrat* or personnel or staff or volunteer* or advocacy or advocate or environment* or "socio-ecolog*" or learn* or policy or policies) near/2 (led or leader* or participat* or mediat* or helper$ or resistan* or wide or action$ or influen* or relationship*)) | 144,680 |  |
| # 10 | #9 OR #8 OR #7 | 222,340 |  |
| # 11 | #10 AND #6 | 41,220 |  |
| # 12 | #11 OR #5 OR #4 | 112,442 |  |
| # 13 | TS=(((smoking or smoked or smoke or smoker or tobacco or cigarette$ or marijuana or cannabis or solvent$) near/5 (reduc* or decreas* or increas* or lower* or fewer or improv* or enhance* or extend* or develop* or prevalen* or daily or weekly or current or rate or rates or behavio$r* or knowledge or prevent* or attitude* or avoid* or perceive* or percept* or promot* or program* or change*))) | 54,491 |  |
| # 14 | TS=(((alcohol or "binge drink*" or "underage drink*" or substance$ or drug$) near/5 (reduc* or decreas* or increas* or lower* or fewer or improv* or enhance* or extend* or develop* or prevalen* or daily or weekly or current or rate or rates or behavio$r* or knowledge or prevent* or attitude* or avoid* or perceive* or percept* or promot* or program* or change*))) | 100,840 |  |
| # 15 | TS=((("education* attain*" or "academic attain*" or "student* attain*" or "education* achieve*" or "academic achieve*" or "student achieve*" or "education* perform*" or "academic perform*" or "education* perform*" or "education* outcome$" or "academic outcome$" or "student* outcome$" or "education* commitment" or "academic commitment" or "student* commitment" or "education* engage*" or "academic engage*" or "student* engage*" or dropout or absentee*) near/5 (reduc* or decreas* or increas* or lower* or fewer or improv* or enhance* or extend* or develop* or prevalen* or daily or weekly or current or rate or rates or behavio$r* or knowledge or prevent* or attitude* or avoid* or perceive* or percept* or promot* or program* or change*))) | 20,235 |  |
| # 16 | TS=(((violent or violence or aggression or aggressive or hostile or hostility or assault* or crime or abuse$ or abusive or misbehav* or threat$ or conduct or delinquen* or disrupt* or "problem* behavio$r*" or "volatile behavio$r*" or antisocial or "anti-social" or perpetrat* or bully* or victim* or cyberbully* or conflict* or "positive behavio$r*" or "improv* behavio$r*" or "social behavio$r*" or "social-emotion*" or "socio-emotion*" or "social-charact*" or "social-inclusion" or "social develop*" or "social competen*" or "emotion* develop*" or conduct or respect or safety or safe) near/5 (reduc* or decreas* or increas* or lower* or fewer or improv* or enhance* or extend* or develop* or prevalen* or daily or weekly or current or rate or rates or behavio$r* or knowledge or prevent* or attitude* or avoid* or perceive* or percept* or promot* or program* or change*))) | 296,224 |  |
| # 17 | #16 OR #15 OR #14 OR #13 | 431,378 |  |
| # 18 | TS=(prevent* or program* or intervent* or "restorative approach*" or "control school$" or trial or trials) | 1,106,156 |  |
| # 19 | #18 AND #17 AND #12 AND #3 | 12,681 |  |

## Trials Register of Promoting Health Intervention (TRoPHI)

| Database name | Trials Register of Promoting Health Information (TRoPHI) |
| --- | --- |
| Database platform | <http://eppi.ioe.ac.uk/webdatabases4/Search.aspx> |
| Dates of database coverage | Complete database to date searched. |
| Date searched | 11/05/2021 |
| Searched by | JF |
| Number of results | 0 |
| EndNote import order | N/A |
| Number of results once duplicates removed | N/A |
| Search strategy notes | None |

Freetext: school OR schools OR schoolwide
